# Revealing an Iranian Isolate of Tomato Brown Rugose Fruit Virus: Complete Genome Analysis and Mechanical Transmission

**DOI:** 10.3390/microorganisms11102434

**Published:** 2023-09-28

**Authors:** Fereshteh Esmaeilzadeh, Adyatma Irawan Santosa, Ali Çelik, Davoud Koolivand

**Affiliations:** 1 Department of Plant Protection, Faculty of Agriculture, University of Zanjan, Zanjan 45371-38791, Irankoolivand@znu.ac.ir (D.K.); 2 Department of Plant Protection, Faculty of Agriculture, Universitas Gadjah Mada, Yogyakarta 55281, Indonesia; 3 Department of Plant Protection, Faculty of Agriculture, Bolu Abant İzzet Baysal University, Bolu 14030, Turkey

**Keywords:** experimental plant hosts, genetic ancestry, mechanical transmission, phylogeny, population genetics, selection constraint

## Abstract

An analysis of the complete genome sequence of a novel isolate of tomato brown rugose fruit virus (ToBRFV) obtained from tomatoes in Iran and named ToBRFV-Ir is presented in this study. Comprehensive phylogenetic analysis utilizing key viral proteins, including 126 KDa, 183 KDa, movement protein (MP), and coat protein (CP), as well as the complete genome sequence, classified ToBRFV-Ir and 65 isolates from GenBank into three distinct clades. Notably, genetic diversity assessment revealed relatively low variability among the isolates, irrespective of their geographical or clade affiliation. Natural selection analysis based on the complete genome sequence showed that dN/dS values were consistently <1, indicating the prevailing role of negative selection across all populations. Analyses using the Recombination Detection Program and SplitsTree found no evidence of recombination events or signals in the complete genome sequence of the tested isolates. Thus, these results suggest that the genetic composition of ToBRFV remains stable without significant genetic exchange or recombination events occurring. A simple arithmetic comparison of the patristic distances and dates suggested that the time to the most recent common ancestor (TMRCA) of the ToBRFV populations is approximately 0.8 up to 2.7 with the closest tobamoviruses. An evolutionary study of the tested isolates from various countries based on the complete genome suggests Peruvian ancestry. The ToBRF-Ir isolate was successfully transmitted through mechanical inoculations to *Solanum lycopersicum* and *Nicotiana rustica*. These findings shed light on the genetic dynamics and transmission mechanisms of ToBRFV, providing valuable insights into its molecular characteristics and potential spread among susceptible plant species.

## 1. Introduction

Tomato (*Solanum lycopersicum* L.) is a highly cultivated vegetable worldwide, including in Iran, due to its nutritional value and high content of antioxidant-rich phytochemicals. This plant species is a member of the family *Solanaceae* and is commonly used in various processed food products, such as salads, soups, pastes, and sauces [1]. However, tomato production can be negatively affected by several pathogens, including viruses of the genus *Tobamovirus*. One of the *Tobamovirus* species that has caused significant concern in recent years is the tomato brown rugose fruit virus (ToBRFV), which was first identified in Jordan in 2016 [2].

ToBRFV is known to mainly infect tomato and pepper (*Capsicum annuum*) plants, causing a range of symptoms such as mild to severe mosaic with dark green bulges on leaves, leaf deformations, and leaf narrowing. The virus also causes brown and yellow spots and blistering on fruits, leading to reduced yield, quality, and marketability of the crop [2,3,4]. The virus can reduce fruit yield by 15–55%, this significant damage can lead to an increase in the rejection of fruit for certification, making it one of the most devastating diseases of tomatoes [5].

The genome of ToBRFV consists of a single-stranded positive-sense RNA encapsulated into a rod-shaped virion. The genome of ToBRFV is similar in architecture to other tobamoviruses and encodes four open reading frames (ORFs). ORF1 and ORF2 are directly translated from the genomic RNA and encode 126 and 183 KDa proteins, respectively, which are responsible for virus replication. ORF3, located on the large sub-genomic RNA, encodes a 30 KDa movement protein, while ORF4, present on the small sub-genomic RNA, expresses a 17.5 KDa coat protein [3].

The global trade of infected fruits and seeds has contributed to the rapid spread of ToBRFV across continents [6,7]. Once introduced to a new area, the virus can easily be transmitted mechanically through human activities involved in tomato production or farming, as well as through tools that come into direct contact with infected plants. ToBRFV has been reported in many countries, including Jordan [2], Mexico [8], Germany [9], Israel [3], Italy [10], Turkey [11], the United States [12], and the Netherlands [13]. To date, limited research has focused on examining the genetic variability of ToBRFV. However, a recent study conducted by [14] revealed a noteworthy observation of a low level of genetic variability among ToBRFV populations. This finding suggests a relatively stable genetic makeup within ToBRFV populations. In 2021, ToBRFV was also detected in greenhouse-grown tomato plants in Iran [15,16]. Later that year, another isolate was collected in December, which infected pepper [17]. Due to the various modes of ToBRFV transmission and the absence of management measures, the virus has spread to new regions of Iran, including different tomato greenhouses in the northwest, south, and southwest of the country.

As of May 2023, NCBI GenBank had recorded 151 complete genome sequences of ToBRFV. However, the full genome sequence of the isolate from Iran is not yet available. Since ToBRFV has a widespread global distribution and is also causing damage in Iran, obtaining a complete genome sequence of the Iranian isolate is crucial for enhancing our knowledge of the virus’s genetic variation in the country. Additionally, thorough analyses of complete genome sequences of all known isolates will help to better comprehend the virus’ diversity, biology, and evolutionary history [18], which is essential for effective detection methods and management strategies.

We present herein the first complete genome sequence of an Iranian ToBRFV isolate obtained from tomato. Using this sequence, along with other available ToBRFV sequences in GenBank, we investigated the phylogenetic relationships and recombination events among ToBRFV isolates. This study also describes detailed genetic diversity analyses of selected ToBRFV isolates based on different parameters. Our study sheds light on the evolutionary history of this virus and provides insights into its global distribution. The results of this investigation provide important contributions to the knowledge of ToBRFV genetic variability and evolutionary patterns, adding to the existing literature on this economically significant plant virus.

## 2. Materials and Methods

### 2.1. Sampling

In 2022, ten tomato plants exhibiting symptoms similar to those infected by ToBRFV, including severe mosaic and blistering on the leaves and yellow spots on the fruits, were collected from a commercial tomato greenhouse complex in Iran.

### 2.2. Sequencing

Total RNA was extracted from the fresh leaves of the collected samples using a method described before [19]. In brief, total RNA was extracted from 100–150 mg of fresh leaf tissue. The leaves were ground in a mortar with liquid nitrogen. 900 μL of extraction buffer (2% CTAB, 2.5% PVP-40, 2 M NaCl, 100 mM Tris-HCL pH 8.0, 25 mM EDTA pH 8.0, and 2% of β- mercaptoethanol added just before use) heated at 65 °C was then added to the samples, mixed by vertexing, and incubated at 65 °C again for 30 min. Then, an equal volume of chloroform and isoamyl alcohol (24:1 *v*/*v*) was added and mixed well, and the homogenate was centrifuged at 13,000 rpm for 10 min at 4 °C. The aqueous phase was transferred into a new tube, and 0.7 to 1.0 volume of ice-cold isopropanol was added and incubated at −20 °C for overnight, then centrifuged at 15,000 rpm for 15 min. The supernatant was discarded, and the pellet was washed with 70% ethanol, dried, and dissolved in 30 µL of DEPC-treated water.

The cDNA was synthesized from the extracted RNA using the Easy cDNA synthesis kit (Pars Tous Biotech, Mashhad, Iran) as per the manufacturer’s instructions and then subjected to RT-PCR. Briefly, reactions were performed in 10 μL reaction mixtures containing 4 μL of total RNA, 5 μL 5× RT-buffer, and 1 µL of enzyme. The mixtures were incubated at 25 °C for 10 min, 47 °C for 60 min, and 85 °C for 5 min. To specifically detect ToBRFV in these samples, a primer pair targeting the coat protein gene of ToBRFV was used. The complete coat protein gene of ToBRFV was successfully amplified from all 10 collected samples. One of the samples, selected as representative of the monitored greenhouse, was used for the complete genome sequencing. To amplify six overlapping segments corresponding to the complete genome, six specific primer pairs were used based on the complete genome sequence of isolate Tom1-Jo in GenBank (with accession number NC_028478). PCR was performed in 25 μL reactions using 3 μL of the synthesized cDNA, 12.5 μL of 2× master mix (Ampliqon A/S, Stenhuggervej, Odense, Denmark), 1.5 μL of each primer (10 pmol), and RNase-free sterile distilled water up to 25 final volumes. PCR conditions involved an initial step of 95 °C for 5 min, followed by 35 cycles of amplification with 95 °C for 30 s, an appropriate annealing temperature for 30 s, and 72 °C for 90 s, with a final extension at 72 °C for 10 min. The primers and annealing temperature information are provided in Table 1. The PCR products were examined by 1% agarose gel electrophoresis. The resulting amplicons obtained from the sample were subjected to cloning into the pTG19 plasmid vector. Briefly, the PCR products were purified using a DNA purification kit, and then the purified amplicons were ligated into the pTG19 plasmid vector using *T4* DNA ligase, ensuring compatible cohesive ends between the PCR product and the plasmid vector. The ligation reaction was performed according to the manufacturer’s instructions, typically involving incubation at an optimal temperature for DNA ligation. Following ligation, the recombinant plasmids containing the inserted amplicons were transformed into competent *Escherichia coli* cells using the heat shock method. The transformed cells were plated onto selective agar plates containing an appropriate antibiotic for plasmid selection. To confirm the successful cloning and obtain the sequence information, each recombinant plasmid clone was subjected to bidirectional sanger sequencing (BGI Tech. Solutions, Shenzhen, China). The six overlapping segments obtained were analyzed using BLASTn and then assembled using BioEdit version 7.1.3.0 [20] to generate the complete genome sequence of the Iranian isolate. The complete genome sequence of the Iranian isolate was submitted to GenBank.

### 2.3. Phylogenetic Analysis

In order to characterize the relationship between ToBRFV-Ir and other ToBRFV isolates, we compiled a complete genome sequence from various countries. Multiple sequence alignments of an Iranian isolate with selected sequences from GenBank were performed with ClustalW with Transition Weight of 0.5 and a 30% cutoff. The resulting alignments were used to construct neighbor-joining (NJ) and maximum likelihood (ML) trees based on the complete genome and specific gene regions (126 kDa, 183 kDa, MP, and CP) using the MEGA11 software with 1000 bootstrap replicates [21]. The program jModelTest 2 was utilized to identify the suitable nucleotide substitution models for each partition [22]. The detailed information on all used sequences is provided in the Supplementary File. The Sequence Demarcation Tool (SDT v1.2) with ClustalW for alignment was used to calculate the pairwise nucleotide sequence identity matrix [23]. To identify potential recombination events in the complete genome sequence, recombination analyses were performed using Recombination Detection Program (RDP v.4.97) [24] and SplitsTree version 4.13 [25]. For network analysis, FASTA format files generated from MEGA were implemented in SplitsTree with default settings. Additionally, RDP4 was utilized with various recombination detection algorithms, including GENECONV, Bootscan, Chimaera, MaxChi, SiScan, 3Seq, and RDP, to calculate recombinant events. Putative recombinant events based on three or more of the algorithms were calculated as recombinations. In addition, a maximum likelihood tree was constructed based on the complete genomic sequences of ToBRFV-Ir and other ToBRFV isolates from different geographical locations of the world to obtain the ancestral sequence of the virus, whereas tobacco mosaic virus (NC_001367) was used as the outgroup.

**Table 1:** Primers used for the ToBRFV detection by RT-PCR.

Primer Name	Sequence (5′ to 3′)	Ta (°C)	Size (bp)	Reference
ToBRFV-1F	TTTTACAACATATACCAACAACAACAAACAAC	54	1193	[26]
ToBRFV-1R	GAACTATGACCATATCTCTCATTTTTGG			
ToBRFV-2F	AGCAATTTTACAGCGCAATG	54	1338	[26]
ToBRFV-2R	CAACGTGGTACTTCCTAGCATGTG			
ToBRFV-3F	CAAGATCCTAAAGGATACAGCTGCTATAG	56	1352	[26]
ToBRFV-3R	TGACATTAAGAGAAATGTCAGTCAACC			
ToBRFV-4F	AGGTTCTAATCTTTTTGTTGCAGC	54	1349	[26]
ToBRFV-4R	CCAACTGCGTGTAATACGCACA			
ToBRFV-5F	ATTGTTTATTATGACCCTTTGAAGTTG	54	1339	[26]
ToBRFV-5R	CTATAATCCTATTTCTAGTATCGAAAGCTC			
TBRFV-F-5722	CACAATCGCAACTCCATCGC	54	623	[12,26]
ToBRFV-R (6344)	GTGCCTACGGATGTGTATGA			

### 2.4. Population Genetic Parameters

In the phylogenetic analyses of 66 ToBRFV complete genome sequences along with the new Iranian complete genome, all isolates were grouped into three distinct clades. To further examine the genetic characteristics of these clades, various population genetic parameters were analyzed using DnaSP software version 5.10.01. These parameters included the number of haplotypes (*H*), haplotype diversity (*Hd*), number of polymorphic sites (*S*), total number of mutations (η), average pairwise nucleotide diversity (π = Pi), average number of nucleotide differences (*k*), number of synonymous sites (SS), and number of non-synonymous sites (NS). To assess selection pressure on the ToBRFV complete genome sequence, the ratio of nonsynonymous substitution rate (dN) to synonymous substitution rate (dS) (dN/dS = ω) was estimated for clades. Negative selection pressure was considered when dN/dS < 1, neutral selection when dN/dS = 1, and positive selection when dN/dS > 1, following the methodology described previously [27]. Additionally, three types of neutrality tests (Tajima’s *D*, Fu and Li’s D*, and Fu and Li’s F* tests) were performed using DnaSP to assess neutral evolution in the sequence populations [28,29]. To determine genetic differentiation and gene flow among populations, *K*_S_*, *K*_ST_*, Z*, *S*_nn_, and *F*_ST_ values were calculated using DnaSP [30]. These values provide insights into the genetic differentiation and level of gene flow between populations within clades and across continents. Infrequent gene flow was considered significant if *F*_ST_ > 0.33, while frequent gene flow was suggested if *F*_ST_ < 0.33 [31]. Furthermore, the nucleotide sequences of the complete ToBRFV genome were used to calculate the ratio of nonsynonymous (dN) to synonymous (dS) nucleotide substitutions per site (ω = dN/dS) using the single-likelihood ancestor counting (SLAC) method available on the DataMonkey server (www.datamonkey.org) (accessed on 2 August 2023). This analysis provided additional insights into the selective pressures acting on specific sites within the genome. In addition, it is important to note that all the parameters mentioned above were calculated based on the geographical regions from which the isolated plant virus samples originated and different clades. This approach aimed to capture the genetic diversity and evolutionary dynamics of the virus across different continents and regions.

### 2.5. Molecular Dating Analysis of ToBRFV

The time to the most recent common ancestor (TMRCA) of ToBRFV was estimated using the TreeTime framework. A time-scaled phylogenetic tree was reconstructed using the fast-dating RelTime-ML computational method [32] implemented in MEGA11 software. Default calibration settings for TMRCA estimation were employed. The phylogenetic analysis included representative nucleotide sequences from ToBRFV as well as other related viruses such as tomato mosaic virus (ToMV), tomato mottle mosaic virus (ToMMV), pepper mild mottle virus (PMMoV), tobacco mild green mosaic virus (TMGMV), tobacco mosaic virus (TMV), paprika mild mottle virus (PaMMV), rehmannia mosaic virus (RheMV), bell pepper mottle virus (BPMV), yellow tailflower mild mottle virus (YTMMV), and obuda pepper virus (ObPV). The phylogenetic diversities of these viruses were evaluated based on the relative time estimates of each node in the phylogenetic tree. Pepper ringspot virus (PepRSV) was selected as an outgroup for the analysis. 

### 2.6. Mechanical Transmission

To investigate the transmission ability of the Iranian isolate, a ToBRFV-Ir-infected sample was ground in 1 mL of 0.1 M potassium phosphate buffer (pH 7.0) and mechanically inoculated onto tomato (*Solanum lycopersicum*) and tobacco (*Nicotiana rustica*). Inoculated seedlings were maintained in an insect-proof greenhouse with temperatures between 20 and 25 °C and observed daily for symptom development. RT-PCR was performed using ToBRFV coat protein-specific primer pairs at 10 days post-inoculation (dpi).

## 3. Results

### 3.1. Greenhouse Observation and Complete Genome Sequencing of ToBRFV

**Figure 1:**
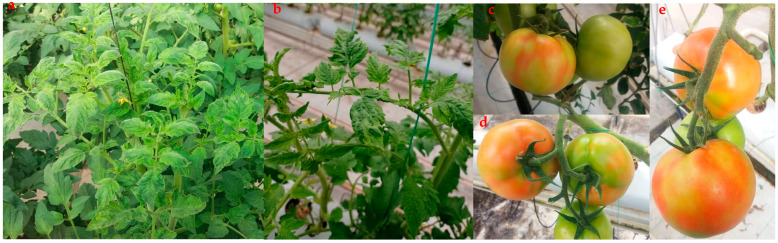
Natural infection of tomato plants with tomato brown rugose fruit virus (ToBRFV) showing (**a**,**b**) severe mosaic and blistering on leaves. (**c**–**e**) discoloration and yellow spots on fruits.

The symptoms of ToBRFV observed in naturally infected tomato plants in the visited greenhouse complex included severe mosaic and blistering on the leaves, as well as yellow spots on the fruit (Figure 1). Notably, the symptoms were more pronounced on almost ripened fruits, where distinct yellow spots or discolorations were observed. These spots appeared as small, irregularly shaped patches on the fruit surface. In some cases, the spots coalesced, forming larger areas of discoloration. The presence of these symptoms on the fruits indicated the systemic spread of the virus within the plant. The severity of the symptoms on young leaves and almost ripened fruits is significant as it highlights the vulnerability of these plant parts to ToBRFV infection. Young leaves are highly susceptible to viral invasion, leading to the development of conspicuous mosaic and blistering symptoms. As the infection progresses, the symptoms intensify, resulting in a more pronounced impact on the fruiting stage.

**Figure 2:**
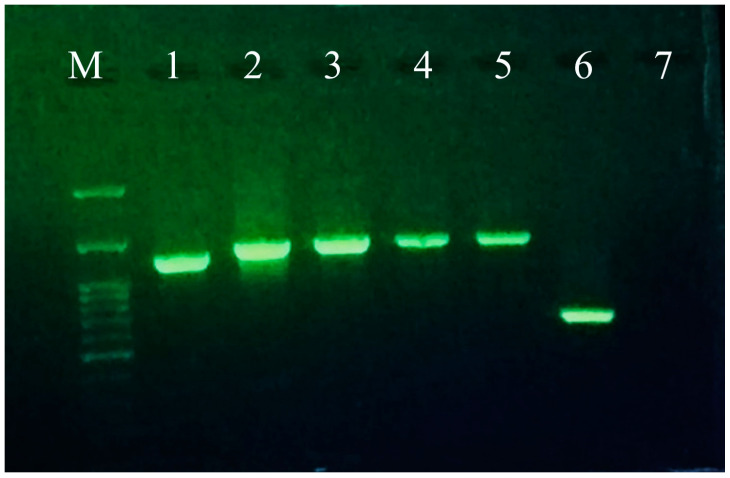
Agarose gel electrophoresis of PCR products of a ToBRFV-Ir-infected sample amplified using six sets of primer pairs: M: 1 kb DNA ladder; lanes 1–6: six overlapping segments corresponding to the complete genome; lane 7: negative control.

Following the amplification of a targeted 623 bp fragment from 10 collected leaves, a positive sample was randomly selected for further analysis, allowing for the generation of the complete genome sequence of ToBRFV. Using six sets of primer pairs, the expected DNA fragments (1193 bp, 1338 bp, 1352 bp, 1349 bp, 1339 bp, and 623 bp) corresponding to four ORFs in the ToBRFV genome were amplified by RT-PCR (Figure 2). After sequence assembly, the consensus sequence of the Iranian ToBRFV isolate was deposited in GenBank under accession number OP557566. The full genome sequence of the Iranian ToBRFV isolate (hereafter ToBRFV-Ir) was 6335 nucleotides (nt) long. The lengths of ORFs I, II, III, and IV were 3351 nt, 4884 nt, 801 nt, and 480 nt, respectively.

BLASTn analysis of ToBRFV-Ir and its four ORFs revealed that ToBRFV-Ir shared high nucleotide sequence identity (98.62–99.81%) with other isolates available in GenBank. Specifically, ToBRFV-Ir showed the highest similarity with the Jordan isolate (KT383474) at the nucleotide sequence level (99.81%). Moreover, the nucleotide sequence similarities of 126 KDa, 183 KDa, MP, and CP of ToBRFV-Ir were found to be 99.67–99.82%, 99.71–99.86%, 99.13–99.5%, and 99.79–100%, respectively, when compared to reported isolates from around the world.

### 3.2. Phylogenetic Relationships of ToBRFV Isolates

The phylogenetic relationship between the ToBRFV-Ir isolate and other complete genome sequences of ToBRFV isolates was obtained from 15 countries, including Jordan, Mexico, China, Italy, the Netherlands, the USA, Israel, Belgium, Germany, the State of Palestine, Peru, Turkey, Canada, Egypt, and the United Kingdom. The final data set included 66 sequences for the 126 KDa, 183 KDa, MP, and CP complete genomes.

**Figure 3:**
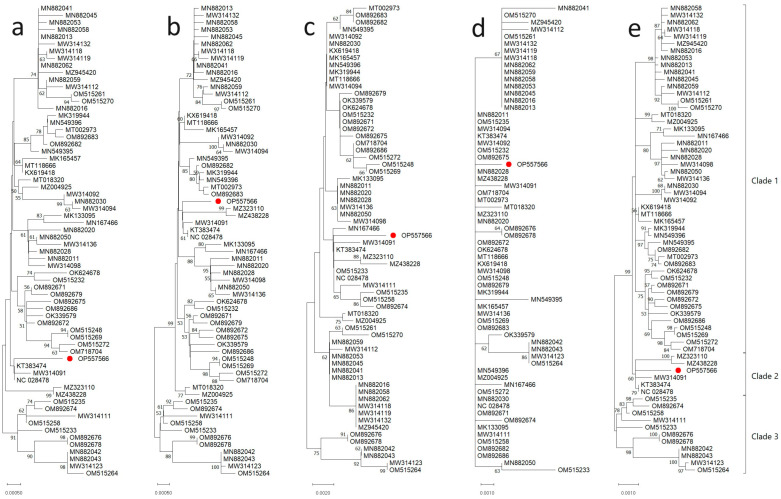
Phylogenetic analysis of the ToBRFV-Ir isolate and other 65 sequences based on (**a**) 126 KDa, (**b**) 183 KDa, (**c**) MP, (**d**) CP, and (**e**) the complete genome sequence Phylogenetic trees were constructed by the neighbor-joining and maximum-likelihood algorithms using MEGA 11 and subjected to 1000 bootstrap replicates. Bootstrap values below 50% are not shown. Red dots indicated locations of ToBRFV-Ir in the constructed tree.

A phylogenetic tree was constructed using the maximum likelihood method and the T92 + G + I model of nucleotide substitution for the complete genome, different nucleotide sequence regions of the ToBRFV-Ir isolate, and comparable sequences from 15 countries. The results showed that the ToBRFV-Ir isolate clustered with isolates from Jordan and China based on all analyses (Figure 3). The phylogenetic analysis based on the complete genome of ToBRFV yielded consistent results that were supported by the analysis conducted on different genomic regions. This convergence of results across multiple genomic segments reinforces the robustness and reliability of the phylogenetic relationships inferred in this study. Based on the complete 126 KDa nucleotide sequence, the ToBRFV-Ir isolate was placed in clade I alongside isolates from various countries (Figure 3a). Further analysis using the neighbor-joining method and the T92 + G model for complete 183 KDa nucleotide sequences showed that the ToBRFV-Ir isolate was clustered with isolates from Jordan and China (Figure 3b). Similar results were obtained when analyzing complete MP nucleotide sequences, where ToBRFV-Ir formed a cluster with isolates from several countries, including Italy, China, Jordan, Peru, and the USA (Figure 3c). A phylogenetic tree was also constructed using the neighbor-joining method and the T92 + G model for complete CP nucleotide sequences, which showed that ToBRFV-Ir grouped with isolates from Mexico, Turkey, Israel, China, Peru, Canada, the USA, Jordan, the Netherlands, Italy, Egypt, the State of Palestine, Germany, and the United Kingdom (Figure 3d). Finally, a phylogenetic tree was constructed using the maximum likelihood method and the HKY + G model based on the complete genome sequence of ToBRFV-Ir and 66 other ToBRFV isolates. The results showed that ToBRFV-Ir formed a cluster with isolates from Jordan and China (Figure 3e).

Overall, the phylogenetic analyses based on nucleotide sequences of the 126 KDa, 183 KDa, MP, and CP, as well as on the complete genome sequence, clustered ToBRFV isolates into three clades, which were further divided into sub-groups. The majority of ToBRFV isolates clustered in clade I, with the exception of complete CP nucleotide sequences. Moreover, based on complete 126 KDa, 183 KDa, and MP nucleotide sequences, the ToBRFV-Ir isolate was placed in clade I, while based on complete CP nucleotide sequences and the complete genome sequence, it was placed in clade II.

**Figure 4:**
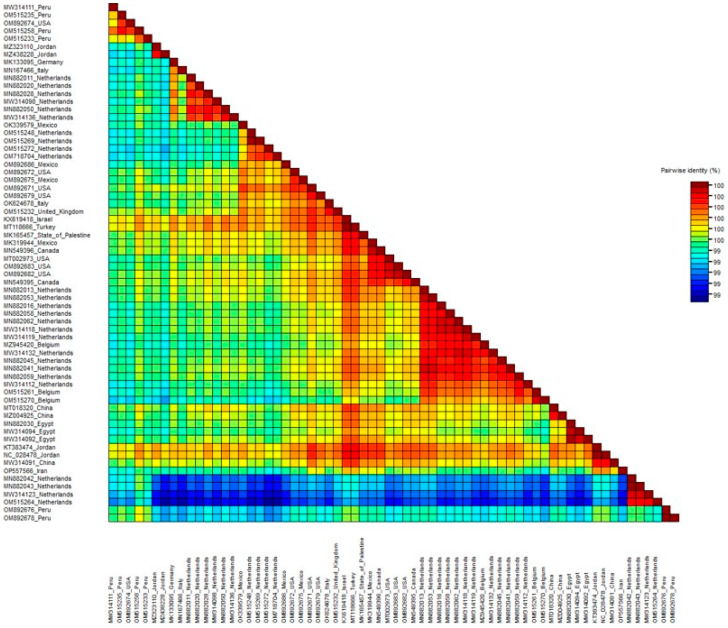
Pairwise sequence comparison of the complete genome sequence of ToBRF-Ir and other ToBRFV isolates using a sequence demarcation tool Pairwise sequence identities (%) between the isolates are represented in different color codes. Each colored key represents a percentage of the identity score between two sequences.

**Figure 5:**
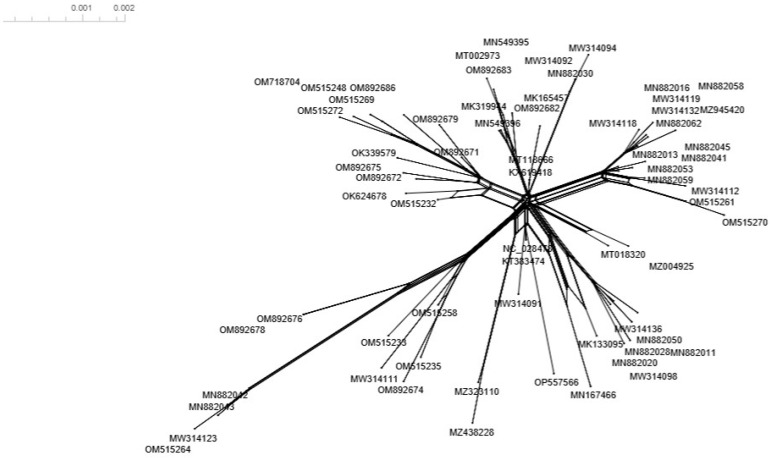
Splits network analysis of the complete genome sequence of 66 ToBRFV isolates from different geographic regions created with the SplitsTree program.

Pairwise comparison of nucleotide sequences using SDT revealed a high sequence similarity ranging from 99% to 100% between the ToBRFV-Ir isolate and other ToBRFV isolates (Figure 4). To investigate the potential recombination events among the examined ToBRFV isolates, we constructed a split network based on the complete sequences using the SplitsTree program (Figure 5). However, our recombination analysis using SplitsTree and RDP4 did not detect any evidence of recombination in the selected ToBRFV isolates for this study. These findings further support the notion that recombination does not play a prominent role in the evolution of ToBRFV isolates. Due to the high similarity of the selected sequences for generating phylogenetic trees, one strain from each country was chosen for ancestral analysis. A maximum likelihood tree was constructed to reconstruct the ancestral sequence of ToBRFV, and it indicated that the Peruvian ToBRFV isolate serves as the ancestor for other isolates from various countries.

### 3.3. Population Genetic Structure 

The present study investigated the genetic diversity and dN/dS ratio of 66 ToBRFV isolates, including the isolate obtained in this study, based on their grouping into three clades formed in the phylogenetic tree (referred to as clades I, II, and III) and their geographic distribution across different continents. The isolates were further categorized based on the continent of origin, including Asia (China, Israel, Jordan, the State of Palestine, Turkey, and Iran), Europe (Belgium, the United Kingdom, Germany, Italy, and the Netherlands), Africa (Egypt), South America (Peru), and North America (Canada, Mexico, and the USA). The number of haplotypes varied across the clades, ranging from 5 in clade II to 48 in clade I. Clade I exhibited the highest haplotype diversity (0.998), while Clade II showed the lowest haplotype diversity (0.933). The number of segregating sites differed among the phylogroups, with Clade II having 33 segregating sites and Clade I having 171. The total number of mutations ranged from 33 in clade II to 172 in clade I. Notably, clade III exhibited the highest nucleotide diversity (Pi = 0.00331), whereas clade II displayed the lowest nucleotide diversity (Pi = 0.00219). Additionally, when considering the continents, the number of haplotypes ranged from 3 in Africa to 32 in Europe. North America showed the highest haplotype diversity (1.000), while Africa exhibited the lowest haplotype diversity (0.833). The number of segregating sites varied among the continents, with Africa having only three segregating sites and Europe having 136. The nucleotide diversity was highest on the European continent (Pi = 0.00348) and lowest on the African continent (Pi = 0.00025). The total number of mutations ranged from 3 in Africa to 138 in Europe (Table 2).

Clade I displayed the highest genetic diversity among the three clades, characterized by 48 haplotypes, a haplotype diversity of 0.964, 171 segregating sites, and a total of 172 mutations (Eta). In contrast, clade II had the lowest genetic diversity with only 5 haplotypes, a haplotype diversity of 0.933, 33 segregating sites, and 33 mutations (Eta). Clade III showed an intermediate level of genetic diversity, with a nucleotide diversity (Pi) of 0.00331. The results revealed that Clade II and the Africa continent exhibited the lowest values for *S* (number of segregating sites), η (total number of mutations), *k* (haplotype diversity), and π (nucleotide diversity), indicating a lower level of genetic variation among their respective ToBRFV isolates. In contrast, the Europe continent showed the highest values for these parameters, indicating a greater genetic variability within its ToBRFV population. To assess the selection pressure acting on the ToBRFV complete genome, we calculated the dN/dS ratios separately for the populations categorized based on clades and continents. The dN/dS ratios for the clades ranged from 0.1162 (clade II) to 0.1736 (clade I). Among the continents categories, the North America continent exhibited the lowest dN/dS ratio (0.1223), while the Europe continent showed the highest dN/dS ratio (0.1738). Overall, both the clades and continents populations displayed dN/dS ratios lower than 1, indicating negative (purifying) selection was predominant in these populations. 

This further supports the notion that the ToBRFV populations are undergoing selective pressure that favors the removal of deleterious genetic variants. In our research, we observed higher ω (dN/dS) ratios in the Europe continent and clade I compared to other continents’ ToBRFV populations and other clades. This could potentially be attributed to the fact that our study included a diverse collection of ToBRFV isolates originating from different geographic regions. It is also worth noting that a majority of the ToBRFV sequences analyzed in our study were affiliated with Clade I and the European continent. These factors may have contributed to the observed differences in ω values. To assess the presence of neutral selection among the populations of ToBRFV, we performed three statistical tests: Tajima’s *D*, Fu and Li’s D*, and Fu and Li’s F*. In general, the statistical tests yielded non-significant negative values across all populations within the clades and continents, indicating the predominance of purifying (negative) selection (Table 3). However, there was one exception that requires further investigation. Notably, the populations belonging to Clade I exhibited significantly negative values in the three tests. Hence, the results indicated a low-frequency polymorphism and suggested the operation of negative selection on those populations. Genetic differentiation and gene flow among populations were assessed using five permutation-based statistical tests: *K*_S_*, *K*_ST_*, Z*, *S*_nn_, and *F*_ST_. The results revealed significant genetic differentiation between ToBRFV populations from different phylogroups and different continents, as indicated by significant results from the *K*_S_*, Z*, and *S*_nn_ tests (*p* < 0.05). The *S*_nn_ values were consistently high (1.000) in all clade comparisons and relatively high (0.947–1.000) in all continent comparisons.

**Figure 6:**
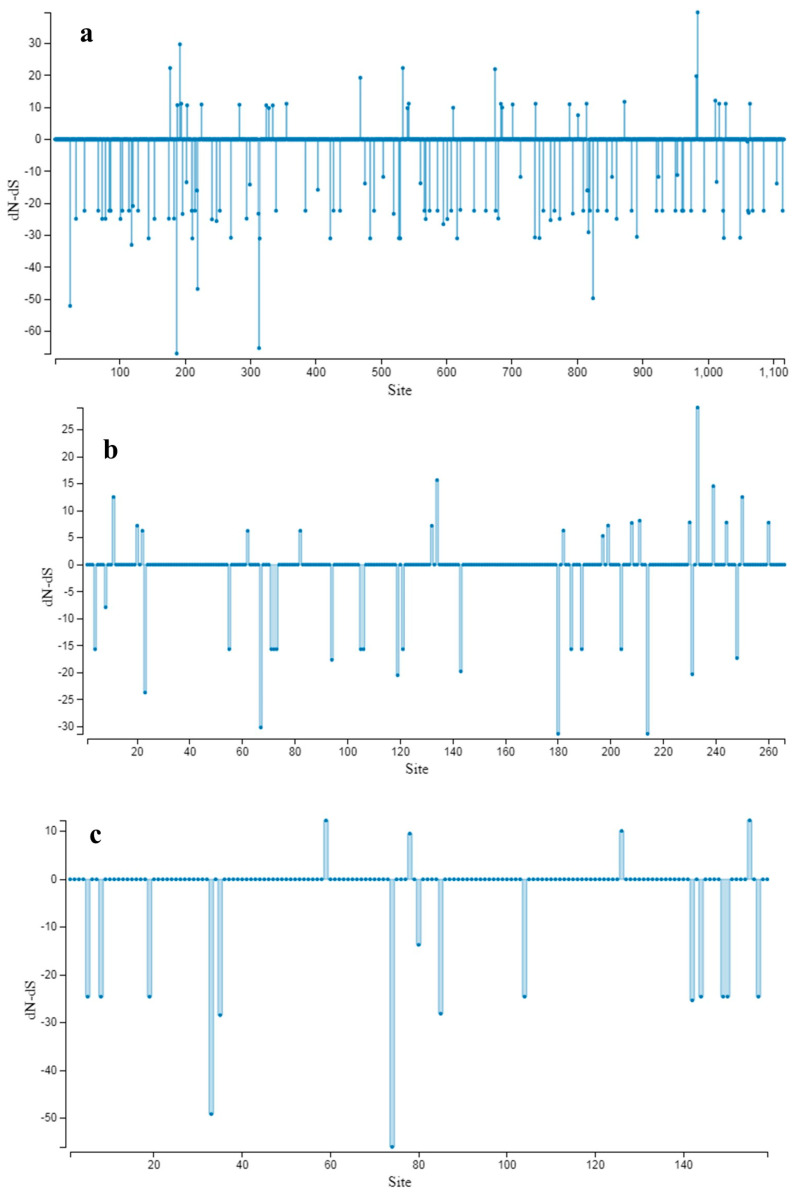
SLAC site graph to identify positive and negative codons/sites for (**a**) 126 KDa, (**b**) MP, and (**c**) CP.

The estimation of gene flow using the *F*_ST_ parameter showed infrequent gene flow between clade I vs. clade III and clade II vs. III in phylogroup comparisons, as well as between Europe vs. Africa, South America vs. Asia, South America vs. Africa, South America vs. North America, Asia vs. Africa, and Africa vs. North America in continent comparisons (*F*_ST_ > 0.33 indicates infrequent gene flow, while *F*_ST_ < 0.33 suggests frequent gene flow). On the other hand, frequent gene flow was observed between clade I vs. clade II in phylogroup comparisons and between Europe vs. Asia, Europe vs. South America, Europe vs. North America, and Asia vs. North America in continent comparisons (Table 4). The lowest *F*_ST_ values in clade comparisons were obtained for clade I vs. II (0.197), while in continent comparisons, the lowest value was observed between Asia vs. North America, indicating low genetic differences between these related populations. No positively selected sites were detected by the more conservative SLAC method (Figure 6). Our findings suggest that the genetic diversity of ToBRFV complete sequences in both groups is low, and the presence of negative selection is evident in these populations. This observation aligns with a previous study indicating a lower degree of variability and strong negative selection on all ORFs of the ToBRFV genome. Furthermore, our results indicate that neither continent categories nor clade-based groupings significantly affect the genetic structure of ToBRFV populations.

### 3.4. Molecular Dating Analysis of ToBRFV

**Figure 7:**
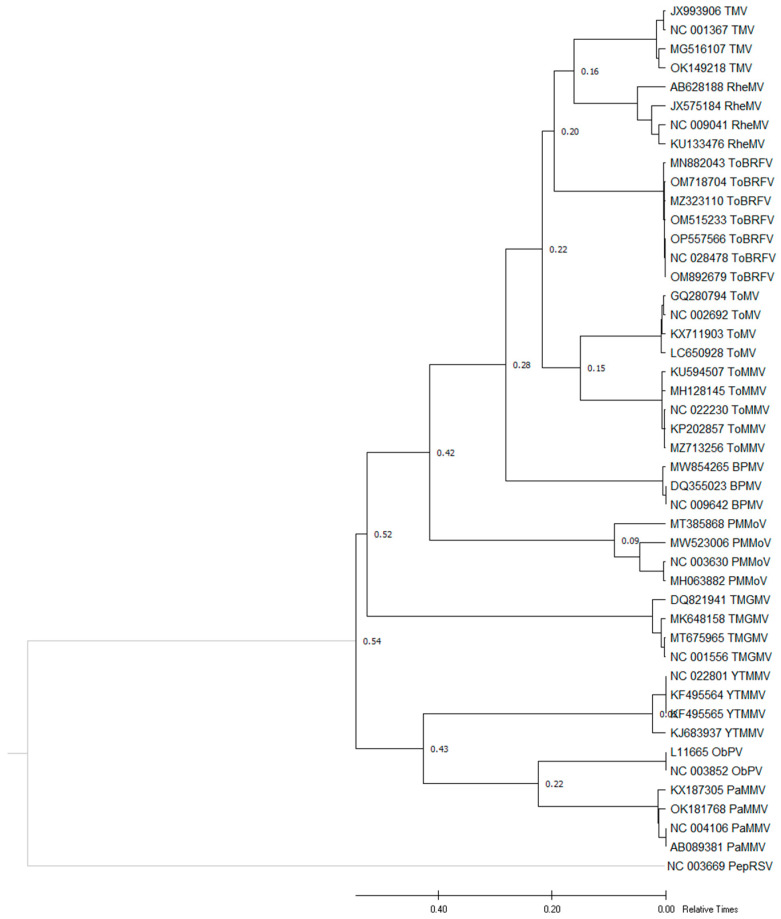
A maximum likelihood phylogeny of the complete genome sequences of representative isolates of tomato brown rugose fruit virus (ToBRFV), tomato mosaic virus (ToMV), tomato mottle mosaic virus (ToMMV), pepper mild mottle virus (PMMoV), tobacco mild green mosaic virus (TMGMV), tobacco mosaic virus (TMV), paprika mild mottle virus (PaMMV), rehmannia mosaic virus (RheMV), bell pepper mottle virus (BPMV), yellow tailflower mild mottle virus (YTMMV), and obuda pepper virus (ObPV) were compared to the divergence time estimation of ToBRFV with ten other tobamoviruses. The pepper ringspot virus (PepRSV) was used as the outgroup.

The estimation of the relative time to TMRCA of ToBRFV was performed using the ratios of ToBRFV and other patristic distances. This approach allowed for the construction of a maximum likelihood phylogenetic tree, which provided insights into the estimated relative time of each node. The analysis involved the comparison of seven ToBRFV isolates, representing two or three isolates from each clade, with ten other tobamovirus isolates obtained from various countries. The inclusion of these isolates in a single phylogeny enabled the estimation of the divergence time between ToBRFV and other tobamoviruses, which was found to be approximately 0.8 to 2.7 (Figure 7, Table 5).

By employing the ratios of patristic distances and constructing a maximum likelihood phylogenetic tree, we were able to estimate the relative time of each node and elucidate the divergence time between ToBRFV and other tobamoviruses. These findings contribute to our understanding of the evolutionary dynamics and relationships within the genus *Tobamovirus*, specifically shedding light on the evolutionary history of ToBRFV. Our study presents compelling evidence regarding the TMRCA of ToBRFV and its divergence from other tobamoviruses, corroborating previous research and expanding our knowledge of this economically significant plant virus. 

### 3.5. Mechanical Transmission

**Figure 8:**
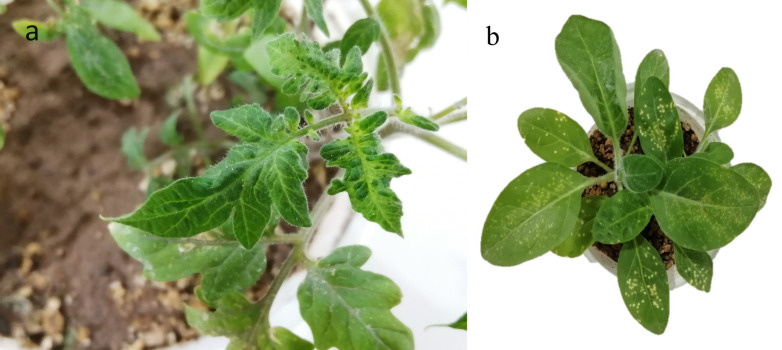
Symptoms on plants inoculated with ToBRFV-Ir. (**a**) mosaic and blistering on tomato leaves at 12 dpi; (**b**) necrotic/chlorotic local lesions on *N*. *rustica* at 3 dpi.

The results of the mechanical inoculation of plants with sap from a positive sample revealed that ToBRFV-Ir can cause symptoms such as mosaic and blistering on tomato plants and chlorotic local lesions in *N. rustica* (as shown in Figure 8). In contrast, plants inoculated with the buffer did not develop any symptoms. *N*. *rustica* showed symptoms at 3 dpi, while symptoms such as mosaic and blistering were observed on tomatoes at 12 dpi. The inoculated plants were then tested using specific primers for ToBRFV, and the infection was confirmed through RT-PCR in these plants.

## 4. Discussion

The seed-borne nature of ToBRFV and, subsequently, the global trade of contaminated seeds (seed movement and infected plant materials) across international borders have had a significant impact on the global economy and have resulted in the emergence or introduction of the disease in new locations [33,34], including Iran [15,16,17], where it has affected the country’s agricultural production of tomatoes. Given that no complete genome sequence of this virus has been reported from Iran, and to ensure the safety of this product against new strains or potential variants, we initially sequenced the complete genome of an isolate obtained from tomato plants. Subsequently, using various genetic analyses, we investigated the genetic diversity of the complete genome and four open reading frames (ORFs) of this isolate compared to other isolates. Based on the results of this study, in line with previous reports, ToBRFV can lead to various typical symptoms ranging from mild to severe mosaic, leaf narrowing, blistering, and yellow areas on the fruit [3,8,9,10,13,26]. The presence of these fruit symptoms, which can reduce fruit marketability, has resulted in economic losses for tomato producers [2,3,4]. Symptoms on plants inoculated with ToBRFV-Ir: (a) mosaic and blistering on tomato leaves at 12 dpi; (b) necrotic/chlorotic local lesions on N. rustica at 3 dpi.

The results of this study also demonstrated that nucleotide diversity among ToBRFV isolates was up to around 98.62–99.81% for the new Iranian isolate. The phylogenetic analysis of ToBRFV based on the complete genome sequences and different genomic regions revealed distinct clustering patterns, indicating the presence of major phylogenetic groups among the viral isolates. These groups often consist of closely related subgroups that share common ancestry. The analysis based on the complete genome sequences provided a comprehensive overview of the evolutionary relationships among ToBRFV strains. It allowed for a thorough examination of the entire viral genome and provided insights into the overall genetic diversity and relatedness among different isolates. The results demonstrated the existence of three major phylogenetic groups, each representing a distinct lineage of ToBRFV. Interestingly, the phylogenetic analysis based on specific replication-associated genes yielded results that were in close agreement with the analysis conducted using complete genome sequences. This indicates a higher level of concordance between the two approaches, suggesting that the RdRp genes capture the evolutionary relationships of ToBRFV strains with greater accuracy and reliability. The findings emphasize the importance of utilizing both complete genome analysis and specific gene-based analyses in phylogenetic studies. While the complete genome analysis provides a comprehensive perspective, the replication-associated gene analysis offers a more targeted examination of specific genetic regions directly involved in viral replication and evolution.

Recombination, which is a fundamental mechanism for generating diversity in RNA viruses, was not detected in ToBRFV sequences using two different approaches. The limited genetic variability among ToBRFV isolates makes it challenging to identify recombinants or distinguish them from parental sequences. This suggests that recombination events may be rare or undetectable due to the low genetic diversity observed among ToBRFV isolates. This study also highlights the genetic relatedness of ToBRFV-Ir to other known isolates, indicating the presence of a conserved genomic background among different strains of ToBRFV. The high nucleotide sequence identities observed in multiple regions of the genome further support the close evolutionary relationships between ToBRFV-Ir and the global population of ToBRFV. The extensive sequence similarities between ToBRFV-Ir and other isolates from diverse geographic locations reinforce the global distribution and spread of this economically significant plant virus. The results demonstrated a high degree of similarity among isolates from different regions, indicating a limited genetic diversity of the virus. Our results indicated that continent categories and clade-based grouping did not impact the genetic structure of ToBRFV populations.

These findings were consistent with those reported by [14], suggesting that geographic regions and grouping based on ORFs, CPs, and the complete genome play a negligible role in the evolution of ToBRFV isolates. The dN/dS ratios, which were consistently below 1, both for continent categories and clade-based categorization, indicate that negative selection is the predominant force shaping the evolution of the ToBRFV genome. This finding aligns with previous reports emphasizing the role of purifying selection in the evolution of ToBRFV. Negative selection is commonly observed during the evolution of plant viruses when analyzing the entire genome, and it is driven by internal and external constraints on the virus [35]. Additionally, selection processes, including the genetic structure of viral populations, can be influenced by various factors such as geographical region, virus structural features, host plants, and insect vectors [36].

In terms of gene flow, frequent gene flow was observed between clade I and clade II in phylogroup comparisons, as well as between Europe and Asia, Europe and South America, Europe and North America, and Asia and North America in continent comparisons. These findings suggest that low gene flow and the absence of recombination contribute to the reduced genetic variability observed in these populations. Alternatively, the low genetic variability in ToBRFV populations may itself contribute to the limited gene flow and absence of recombination. The low level of gene flow and genetic variability, along with the high degree of similarity among isolates from different geographic regions, suggest a common origin, such as contamination through infected seeds or the exchange of infected fruit among countries. These findings are consistent with a previous study [7]. 

Ancestral studies have suggested that Peru may be the center of emergence for ToBRFV [13,14]. Further support for this conclusion is obtained from the study by [7] which suggests the origin of ToBRFV in South America due to the divergence of isolates from Peru. Our findings regarding the TMRCA of the ToBRFV population also align with previous studies’ assessments. However, to better understand and ensure this hypothesis, more isolates from different locations need to be examined.

## 5. Conclusions

Given the global prevalence of ToBRFV, including in Iran, understanding the genetic structure of the virus and the factors or selective forces driving its evolution, such as negative selection, can greatly assist in future surveillance and research efforts, and with continuous monitoring, can prevent the emergence of new isolates or possible variants.

## Figures and Tables

**Table 2:** Genetic diversity analyses of ToBRFV complete sequences based on phylogroups and continents.

Population	*N*	*H*	*Hd*	*S*	η	*k*	π	SS	NS	dS	dN	ω
Phylogroup												
Clade I	49	48	0.998	171	172	16.660	0.00272	1365.22	4745.78	0.00760	0.00132	0.1736
Clade II	6	5	0.933	33	33	13.400	0.00219	1366.94	4750.06	0.00697	0.00081	0.1162
Clade III	11	9	0.964	61	62	20.236	0.00331	1366.94	4750.06	0.00954	0.00152	0.1593
Continent												
Europe	33	32	0.998	136	138	21.303	0.00348	1366.76	4750.24	0.00972	0.00169	0.1738
South_America	6	5	0.933	37	38	16.000	0.00262	1366.44	4750.56	0.00742	0.00123	0.1657
Asia	11	9	0.964	51	51	12.727	0.00208	1365.12	4745.88	0.00583	0.00100	0.1715
Africa	4	3	0.833	3	3	1.500	0.00025	1366.92	4750.08	0.00073	0.00011	0.1506
North America	13	13	1.000	62	63	13.846	0.00226	1366.56	4750.44	0.00711	0.00087	0.1223

*N*: number of isolates; *H*: haplotype number; *Hd*: haplotype diversity; *S*: total number of segregating (polymorphic) sites; Eta (η): total number of mutations; *k*: average number of nucleotide differences between sequences; Pi (π): nucleotide diversity; dS: frequency of synonymous substitution per site; dN: frequency of nonsynonymous substitution per site; ω: dN/dS.

**Table 3:** Neutrality tests of complete genome of ToBRFV isolates in different populations based on phylogroups and continents.

Population	π	Tajima’s *D*	Fu and Li’s D*	Fu and Li’s F*
Phylogroups				
Clade I	0.00272	−2.03856 *	−3.49583 *	−3.52417 *
Clade II	0.00219	−0.46253 ns	−0.36366 ns	−0.42092 ns
Clade III	0.00331	−0.20899 ns	−0.33262 ns	−0.34157 ns
Continents				
Europe	0.00348	−1.41629 ns	−1.85488 ns	−2.02230 ns
South America	0.00262	−0.24596 ns	−0.08919 ns	−0.13431 ns
Asia	0.00208	−1.27138 ns	−1.00970 ns	−1.22239 ns
Africa	0.00025	−0.75445 ns	−0.75445 ns	−0.67466 ns
North America	0.00226	−1.43378 ns	−2.03804 ns	−2.14714 ns

* 0.01 < *p* value > 0.05; ns = not significant.

**Table 4:** Genetic differentiation estimates for lineages of ToBRFV, based on complete genome.

Population	*K*_S_*	*K*_ST_*	*K*_S_*, *K*_ST_* *p*-Value	Z*	*p*-Value	*S*_nn_	*p*-Value	*F*_ST_
Phylogroups								
Clade I (*n* = 50)/Clade II (*n* = 6)	2.77039	0.01671	0.0000 ***	6.25047	0.0010 **	1.000	0.0000 ***	0.197
Clade I (*n* = 50)/Clade III (*n* = 11)	2.80635	0.05540	0.0000 ***	6.21615	0.0000 ***	1.000	0.0000 ***	0.360
Clade II (*n* = 6)/Clade III (*n* = 11)	2.74113	0.09697	0.0000 ***	3.50621	0.0000 ***	1.000	0.0000 ***	0.387
Continents								
Europe (*n* = 33)/Asia (*n* = 11)	2.831	0.032	0.0000 ***	5.678	0.0000 ***	1.000	0.0000 ***	0.140
Europe (*n* = 33)/Africa (*n* = 4)	2.812	0.049	0.0000 ***	5.266	0.0000 ***	1.000	0.0000 ***	0.488
Europe (*n* = 33)/S. America (*n* = 6)	2.908	0.039	0.0000 ***	5.388	0.0000 ***	1.000	0.0000 ***	0.283
Europe (*n* = 33)/N. America (*n* = 13)	2.847	0.035	0.0000 ***	5.744	0.0000 ***	0.978	0.0000 ***	0.144
S. America (*n* = 6)/Asia (*n* = 11)	2.515	0.098	0.0000 ***	3.511	0.0000 ***	1.000	0.0010 **	0.338
S. America (*n* = 6)/Africa (*n* = 4)	2.049	0.253	0.0010 **	2.163	0.0020 **	1.000	0.0100 *	0.657
S. America (*n* = 6)/N. America (*n* = 13)	2.605	0.086	0.0000 ***	3.713	0.0000 ***	0.947	0.0000 ***	0.344
Asia (*n* = 11)/Africa (*n* = 4)	2.156	0.141	0.0000 ***	3.236	0.0000 ***	1.000	0.0020 **	0.564
Asia (*n* = 11)/N. America (*n* = 13)	2.525	0.042	0.0000 ***	4.415	0.0000 ***	0.958	0.0000 ***	0.138
Africa (*n* = 4)/N. America (*n* = 13)	2.315	0.121	0.0000 ***	3.428	0.0010 **	1.000	0.0010 **	0.555

Probability (*p* value) obtained by the permutation test with 1000 replicates * 0.01 < *p* < 0.05, ** 0.001 < *p* < 0.01, *** *p* < 0.001. *F*_ST_ > 0.33 suggests infrequent gene flow; *F*_ST_ < 0.33 suggests frequent gene flow.

**Table 5:** Inferred Time of Most Recent Common Ancestor (TMRCA) of ToBRFV based on the ratio of the patristic distances within the ten other tobamoviruses’ complete genome sequence maximum likelihood tree.

Species	Mean Patristic Distance	Ratios of ToBRFV and Other Patristic Distance
TMV	0.16	0.8
RheMV	0.16	0.8
ToBRFV	0.20	1
ToMV	0.22	1.1
ToMMV	0.22	1.1
BPMV	0.28	1.4
PMMoV	0.42	2.1
TMGMV	0.52	2.6
YTMMV	0.54	2.7
ObPV	0.54	2.7
PaMMV	0.54	2.7

## Data Availability

The complete genome sequences of ToBRFV-Ir, the novel Iranian ToBRFV isolate analyzed in this study, have been made available in GenBank under reference number OP557566.

## Supplementary Materials

The following supporting information can be downloaded at: https://www.mdpi.com/article/10.3390/microorganisms11102434/s1, ToBRFV isolates sequence alignments according to 126 KDa, 183 KDa, MP, CP, and the complete genome sequence.
