# Supplementary material for: Revealing an Iranian Isolate of Tomato Brown Rugose Fruit Virus: Complete Genome Analysis and Mechanical Transmission
Source: Microorganisms. 2023 Sep 28;11(10):2434. doi: 10.3390/microorganisms11102434 (PMC10608917; doi:10.3390/microorganisms11102434)
Supplement: Supplementary file 1 [file microorganisms-11-02434-s001.zip › microorganisms-2602812-Supplementary file/Revealing ToBRFV-Spp material.pdf]

Revealing an Iranian isolate of tomato brown rugose fruit virus: complete genome analysis and mechanical transmission

Fereshteh Esmaeilzadeh[1], Adyatma Irawan Santosa[2,*], Ali Çelik[3], Davoud Koolivand[1]

Affiliations
[1]Department of Plant Protection, Faculty of Agriculture, University of Zanjan, Zanjan, Iran
[2]Department of Plant Protection, Faculty of Agriculture, Universitas Gadjah Mada, Jl. Flora No. 1, Sleman, Yogyakarta, 55281, Indonesia
[3]Department of Plant Protection, Faculty of Agriculture, Bolu Abant İzzet Baysal University, 14030 Bolu, Turkey

Correspondence: adyatma.i.s@ugm.ac.id

Alignments of sequence of ToBRFV isolates tested in this study, based on 126 KDa gene, 183 KDa gene, MP gene, CP gene, and the complete genome, are available as Fasta files. Complete nucleotide sequence of ToBRFV isolates tested in this study can also be found below.

KT383474

ATGGCATACACACAGACAGCTACCACATCCGCTTTGCTCGACACTGTCCGAGGTAACAATACCTTGGTCAACG
ATCTTGCGAAGCGGCGTCTTTATGACACAGCGGTCGACGAGTTCAACGCTCGTGATCGCAGGCCCAAAGTAA
ATTTTTCCAAAGTAATAAGTGAGGAACAGACGCTTATTGCTACTAGGGCATATCCAGAATTCCAGATAACCTTC
TATAATACGCAGAACGCCGTGCATTCGCTTGCCGGTGGACTACGATCCTTAGAACTGGAATATCTAATGATGC
AGATCCCGTACGGATCACTCACATATGATATAGGTGGGAATTTTGCATCTCATCTGTTCAAAGGACGGGCATA
TGTTCACTGCTGTATGCCCAATCTTGATGTCCGCGACATAATGCGGCACGAAGGCCAGAAAGACAGTATAGAA
TTATACCTTTCCAGGCTTGAGCGGGGCAACAAAGTTGTCCCAAATTTCCAAAAGGAAGCTTTTGACAGATACG
CTGAAACGCCAGACGAAGTTGTCTGTCACAGTACCTTCCAAACGTGTACGCACCAGCAGGTGGAAAACACAG
GCAGGGTGTATGCTATTGCATTGCACAGTATATACGATATACCTGCTGATGAATTCGGAGCGGCACTTTTAAG
GAAAAATGTCCATGTTTGTTACGCCGCCTTCCACTTTTCCGAGAATTTACTTCTCGAAGATTCACACGTCAACCT
TGACGAAATCAACGCGTGTTTTTCGCGTGATGGAGACAAGCTGACTTTTTCTTTCGCATCTGAGAGCACTTTAA
ATTATTGTCATAGTTATTCTAATATTTTAAAATACGTGTGCAAAACTTACTTCCCGGCATCTAATAGAGAGGTCT
ACATGAAGGAGTTTTTGGTCACCAGGGTTAACACCTGGTTTTGTAAGTTTTCTAGGATAGATACTTTTTTATTAT
ACAAGGGGGTAGCCCACAAAGGTGTAAATAGTGAGCAATTTTACAGCGCAATGGAAGATGCATGGCACTACA
AAAAGACTCTTGCAATGTGTAACAGCGAGAGGATTCTTCTTGAAGATTCCTCATCGGTCAATTACTGGTTCCCA
AAAATGAGAGATATGGTCATAGTTCCTCTATTCGACATATCTCTCGACACCAGTAAAAGGACCCGCAAAGAAG
TCTTAGTGTCAAAGGATTTTGTATTCACAGTTTTAAATCACATTCGCACTTATCAAGCCAAGGCACTTACATACT
CCAATGTTTTATCCTTTGTCGAATCAATTCGTTCAAGGGTAATTATCAACGGAGTGACTGCCAGGTCTGAGTGG
GATGTTGACAAATCTCTTTTGCAATCCTTGTCCATGACATTTTTCTTGCATACTAAGCTTGCCGTTTTAAAAGAC

GAATTGTTAATCAGCAAGTTTAGTTTGGGGCCAAAATCAGTAAGCCAGCATGTATGGGATGAGATTTCCCTGG
CTTTTGGAAACGCATTTCCATCGATCAAGGAGAGACTGCTAAATCGGAAACTAATTAAAGTGTCGGGAGACGC
ATTAGAAATCAGGGTGCCTGATTTATATGTGACTTTTCACGATAGATTAGTGACTGAGTACAAAACATCGGTG
GATATGCCAGTGCTTGATATCAGAAAGAGAATGGAGGAGACTGAGGTTATGTACAATGCATTGTCTGAGCTA
TCTGTGCTCAAGGAGTCGGACAAGTTCGACGTTGATGTTTTTTCCCGGATGTGCCAGACTTTGGAGGTAGACC
CAATGACTGCAGCAAAGGTTATTGTGGCAGTGATGAGCAACGAGAGCGGACTGACTCTTACATTCGAACAGC
CAACTGAAGCAAATGTCGCATTGGCACTTAAAGATTCAGAAAAAGCCTCTGAGGGTGCACTAGTGGTTACTTC
TAGAGATGTTGAAGAACCATCCATGAAGGGTTCAATGGCAAGAGGAGAGTTACAATTGGCCGGTCTGTCTGG
AGACCAACCAGAGTCTTCCTATACTCGGAACGAGGAAATAGAGTCATTAGAGCAATTCCACATGGCAACGGCT
AGTTCGTTAATTCGGAAACAGATGAGTTCGATTGTGTACACGGGCCCCATTAAAGTTCAGCAAATGAAAAACT
TTATTGATAGCCTGGTAGCATCACTCTCTGCTGCGGTGTCGAACCTAGTCAAGATCCTAAAGGATACAGCTGCT
ATAGATCTCGAAACCCGTCAGAAGTTTGGAGTCTTAGATGTTGCGACCAAAAGATGGTTAATTAAACCTTTAG
CCAAGAATCACGCATGGGGCGTTATTGAAACACATGCTAGGAAGTACCACGTTGCACTTTTGGAGTATGATGA
GCATGGAGTGGTAACTTGCGACAGTTGGAGAAGGGTGGCCGTGAGTTCTGAGTCAATGGTTTATTCTGATAT
GGCAAAGCTCAGAACACTGAGGAGATTATTAAGAGATGGTGAGCCTCATGTCAGCAGTGCTAAAGTCGTCCT
AGTTGACGGTGTCCCGGGTTGTGGAAAGACAAAAGAGATTCTCTCGAAAGTAAATTTTGAGGAAGATCTAAT
CTTAGTACCGGGTAAGCAGGCTGCTGAAATGATAAAGAGGCGTGCTAATGCGTCAGGAATAATTCAAGCCAC
AAGAGATAATGTTCGTACTGTTGATTCATTTATAATGAATTACGGTAAAGGAACACGCTGTCAGTTCAAAAGG
TTATTTATCGACGAAGGTCTGATGTTGCACACTGGTTGTGTGAATTTTCTTGTTTCTATGTCTCTGTGCGAAATT
GCATATGTTTATGGAGACACACAACAAATTCCATACATCAACAGAGTATCCGGTTTTCCGTACCCTGCACATTT
TGCAAAAATAGAGGTTGATGAGGTGGAAACTCGCAGAACTACGCTGCGTTGTCCAGCCGACATTACCCACTAT
CTTAACAGAAGGTACGAAGGATATGTCATGTGTACATCGTCGGTTAAAAAGTCAGTTTCTCAGGAAATGGTGA
GCGGGGCCGCAATGATCAATCCTGTATCTAAGCCATTGAATGGGAAAGTTTTGACTTTCACTCAGTCTGATAA
AGAGGCGCTGCTTTCTCGAGGATATACGGACGTCCATACAGTACATGAGGTACAAGGTGAGACATATGCAGA
TGTGTCGTTGGTCAGATTGACTCCGACACCTGTATCTATCATCGCAGGAGATAGTCCGCACGTTCTCGTAGCTT
TGTCAAGGCATACCCAAACATTGAAGTATTACACCGTAGTGATGGATCCTCTTGTAAGTATAATTAGGGATTTA
GAAAAACTTAGTTCTTACTTGTTAGATATGTATAAAGTAGATGCAGGGACCCAATAGCAATTACAGGTAGACT
CCGTGTTTAAAGGTTCTAATCTTTTTGTTGCAGCACCAAAGACTGGAGATATCTCAGATATGCAATTTTACTAT
GATAAGTGTCTCCCAGGTAATAGCACCATGTTAAATAACTATGATGCTGTTACCATGAGGTTGACTGACATTTC
TCTTAATGTCAAAGATTGCATATTGGATTTCTCTAAGTCTGTGGCTGCACCGAAGGATCCGATCAAACCACTGA
TTCCGATGGTACGAACGGCGGCAGAAATGCCACGCCAGACTGGACTATTGGAAAATTTGGTGGCGATGATCA
AAAGAAACTTTAATTCACCGGAGTTATCAGGAATAATCGACATTGAGAATACTGCATCTTTAGTAGTAGATAA
ATTTTTTGATAGTTACTTGCTTAAAGAAAAAAGAAAACCAAATAAAAATGTTTCTTTATTTTGTAGAGAGTCTCT
CAATAGATGGTTAGAGAAGCAGGAGCAAGTGACCATTGGTCAGCTTGCAGATTTTGATTTTGTGGATCTTCCT
GCCGTTGATCAGTACAGGCATATGATTAAAGCGCAACCTAAGCAGAAGCTGGATACATCAATTCAAAGCGAAT
ATCCGGCCTTGCAGACGATTGTGTATCATTCGAAAAAGATCAACGCAATCTTCGGTCCTTTGTTCAGTGAGCTC
ACAAGGCAAATGCTCGAAAGCATAGACTCAAGTAAGTTTTTGTTCTTTACAAGGAAGACGCCAGCTCAAATTG
AGGATTTCTTCGGAGATCTCGATAGCCATGTCCCTATGGATATCTTGGAGTTGGATATTTCGAAGTATGACAAA
TCTCAGAACGAGTTCCACTGTGCAGTAGAGTATGAAATATGGAGAAGACTTGGATTAGAAGATTTTCTGGGA
GAAGTTTGGAAACAAGGCCACAGGAAAACTACTCTTAAAGATTACACAGCTGGTATTAAAACGTGTTTATGGT
ACCAGAGAAAGAGTGGGGACGTTACAACATTCATCGGTAATACGGTGATTATTGCTGCTTGTTTAGCTTCCAT
GTTGCCCATGGAGAAAATAATCAAAGGTGCATTTTGCGGAGATGACAGTTTACTATACTTCCCAAAAGGTTGT
GAGTTTCCTGACATACAGCATACAGCCAACCTTATGTGGAATTTCGAGGCTAAGCTATTCAGAAAGCAGTATG
GTTATTTCTGTGGAAGGTACGTGATACATCATGACAGAGGGTGTATTGTTTATTATGACCCTTTGAAGTTGATT
TCTAAACTTGGTGCTAAACACATCAAGGATTGGGATCACTTAGAAGAGTTCAGAAGATCCCTTTGTGATGTTG
CAAATTCGTTGAACAACTGTGCGTATTACACGCAGTTGGACGACGCTGTGAGTGAGGTCCATAAAACCGCACC
CCCGGGTTCGTTTGTATATAAAAGTTTAGTTAAATATCTGTCCGATAAGGTTCTTTTTAGAAGTTTGTTTATAGA
TGGCTCTTGTTAAGGGTAAAGTCAATATTAATGAGTTCATAGACTTGTCAAAATCAGAAAAATTTCTTCCGTCT

ATGTTCACACCTGTTAAGAGTGTCATGATCTCCAAGGTTGATAAGATATTGGTTCATGAAGATGAATCTTTGTC
CGAAGTCAATTTACTCAAAGGTGTAAAACTCATTGATGGTGGCTATGTACATCTTGCTGGTCTTGTGGTGACA
GGTGAATGGAATTTGCCAGATAATTGTCGTGGTGGTGTCAGTGTCTGTTTGGTCGATAAGAGAATGGAGAGA
GCGGACGAGGCAACTCTTGCTTCATACTATACCGCAGCGGCTAAGAAAAGGTTTCAGTTCAAAGTCGTTCCAA
ATTACAACATCACTACCAAGGACGCAGAAAAGGCAGTTTGGCAAGTACTAGTTAATATTAGAAATGTTAAAAT
TGCTGCGGGTTACTGTCCGCTGTCATTAGAATTTGTGTCAGTGTGTATTGTTTATAAAAATATTATAAAACTCG
GTTTGAGAGAGAAAATTACGAGCGTCACGGATGGAGGGCCCATGGAACTATCAGAAGAAGTTGTTGATGAG
TTCATGGAAGAAGTCCCGATGTCTGTAAGGCTTGCAAAATTTCGTTCGAAGACCGGAAAAAAGTTTAGTAGTA
AAAGTGAGAATAATAGTGGTAATAATAGGCCGAAACCAGACAAAAACCAAAGGAAGGAAAAGGGTTTAAAA
GTTAGGGTTGAGAAGGATAATTTAATTGATAATGAATTGGAGACTTACGTCGCCGATTCAGATTCGTATTAAA
TATGTCTTACACAATCGCAACTCCATCGCAATTTGTGTTTTTGTCATCAGCATGGGCCGACCCTATAGAATTAAT
AAATTTATGTACTAATTCACTAGGTAATCAGTTCCAAACACAACAAGCTAGAACAACCGTTCAACGGCAATTTA
GCGAAGTGTGGAAACCTGTCCCTCAAGTCACTGTTAGGTTTCCTGACAGTGGTTTTAAGGTGTATAGGTACAA
TGCGGTACTAGATCCTCTAGTTACTGCTTTGTTAGGAGCTTTCGATACTAGAAATAGGATTATAGAAGTCGAAA
ATCAGGCGAACCCGACAACCGCCGAAACGTTAGACGCTACTCGTAGAGTAGATGACGCAACGGTGGCTATAA
GGAGCGCTATAAATAATTTAGTAGTAGAATTGGTCAAAGGAACAGGTTTGTACAATCAGAGCACATTTGAAA
GTGCATCCGGTTTACAATGGTCCTCTGCACCTGCATCTTGA

KX619418

ATGGCATACACACAGACAGCTACCACATCCGCTTTGCTCGACACTGTCCGAGGTAACAATACCTTGGTCAACG
ATCTTGCGAAGCGGCGTCTTTATGACACAGCGGTCGACGAGTTCAACGCTCGTGATCGCAGGCCCAAAGTAA
ATTTTTCCAAAGTAATAAGTGAGGAACAGACGCTTATTGCTACTAGGGCATATCCAGAATTCCAGATAACCTTC
TATAATACGCAGAACGCCGTGCATTCGCTTGCCGGTGGACTACGATCCTTAGAACTGGAATATCTAATGATGC
AGATCCCGTACGGATCACTCACATATGATATAGGTGGGAATTTTGCATCTCATCTGTTCAAAGGACGGGCATA
TGTTCACTGCTGTATGCCCAATCTTGATGTCCGCGACATAATGCGGCACGAAGGCCAGAAAGACAGTATAGAA
TTATACCTTTCCAGGCTTGAGCGGGGCAACAAAGTTGTCCCAAATTTCCAAAAGGAAGCTTTTGACAGATACG
CTGAAACGCCAGACGAAGTTGTCTGTCACAGTACCTTCCAAACGTGTACGCACCAGCAGGTGGAAAACACAG
GCAGGGTGTATGCTATTGCATTGCACAGTATATACGATATACCTGCTGATGAATTCGGAGCGGCACTTTTAAG
GAAAAATGTCCATGTTTGTTACGCCGCCTTCCACTTTTCCGAGAATTTACTTCTCGAAGATTCACACGTCAACCT
TGACGAAATCAACGCGTGTTTTTCGCGTGATGGAGACAAGCTGACTTTTTCTTTCGCATCTGAGAGCACTTTAA
ATTATTGTCATAGTTATTCTAATATTTTAAAATACGTGTGCAAAACTTACTTCCCGGCATCTAATAGAGAGGTCT
ACATGAAGGAGTTTTTGGTCACCAGGGTTAACACCTGGTTTTGTAAGTTTTCTAGGATAGATACTTTTTTATTAT
ACAAGGGGGTAGCCCACAAAGGTGTAAATAGTGAGCAATTTTACAGCGCAATGGAAGATGCATGGCACTACA
AAAAGACTCTTGCAATGTGTAACAGCGAGAGGATTCTTCTTGAAGATTCCTCATCGGTCAATTACTGGTTCCCA
AAAATGAGAGATATGGTCATAGTTCCTCTATTCGACATATCTCTCGACACCAGTAAAAGGACCCGCAAAGAAG
TCTTAGTGTCAAAGGATTTTGTATTCACAGTTTTAAATCACATTCGCACTTATCAAGCCAAGGCACTTACATACT
CCAATGTTTTATCCTTTGTCGAATCAATTCGTTCAAGGGTAATTATCAACGGAGTGACTGCCAGGTCTGAGTGG
GATGTTGACAAATCTCTTTTGCAATCCTTGTCCATGACATTTTTCTTGCATACTAAGCTTGCCGTTTTAAAAGAC
GAATTGTTAATCAGCAAGTTTAGTTTGGGGCCAAAATCAGTAAGCCAGCATGTATGGGATGAGATTTCCCTGG
CTTTTGGAAACGCATTTCCATCGATCAAGGAGAGACTGCTAAATCGGAAACTAATTAAAGTGTCGGGAGACGC
ATTAGAAATCAGGGTGCCTGATTTATATGTGACTTTTCACGATAGATTAGTGACTGAGTACAAAACATCGGTG
GATATGCCAGTGCTTGATATCAGAAAGAGAATGGAGGAGACTGAGGTTATGTACAATGCATTGTCTGAGCTA
TCTGTGCTCAAGGAGTCGGACAAGTTCGACGTTGATGTTTTTTCCCGGATGTGCCAGACTTTGGAGGTAGACC
CAATGACTGCAGCAAAGGTTATTGTGGCAGTGATGAGCAACGAGAGCGGACTGACTCTTACATTCGAACAGC
CAACTGAAGCAAATGTCGCATTGGCACTTAAAGATTCAGAAAAAGCCTCTGAGGGTGCACTAGTGGTTACTTC
TAGAGATGTTGAAGAACCATCCATGAAGGGTTCAATGGCAAGAGGAGAGTTACAATTGGCCGGTCTGTCTGG

AGACCAACCAGAGTCTTCCTATACTCGGAACGAGGAAATAGAGTCATTAGAGCAATTCCACATGGCAACGGCT
AGTTCGTTAATTCGGAAACAGATGAGTTCGATTGTGTACACGGGCCCCATTAAAGTTCAGCAAATGAAAAACT
TTATTGATAGCCTGGTAGCATCACTCTCTGCTGCGGTGTCGAACCTAGTCAAGATCCTAAAGGATACAGCTGCT
ATAGATCTCGAAACCCGTCAGAAGTTTGGAGTCTTAGATGTTGCGACCAAAAGATGGTTAATTAAACCTTTAG
CCAAGAATCACGCATGGGGCGTTATTGAAACACATGCTAGGAAGTACCACGTTGCACTTTTGGAGTATGATGA
GCATGGAGTGGTAACTTGCGACAGTTGGAGAAGGGTGGCCGTGAGTTCTGAGTCAATGGTTTATTCTGATAT
GGCAAAGCTCAGAACACTGAGGAGATTATTAAGAGATGGAGAGCCTCATGTCAGCAGTGCTAAAGTCGTCCT
AGTTGACGGTGTCCCGGGTTGTGGAAAGACAAAAGAGATTCTCTCGAAAGTAAATTTTGAGGAAGATCTAAT
CTTAGTACCGGGTAAGCAGGCTGCTGAAATGATAAAGAGGCGTGCTAATGCGTCAGGAATAATTCAAGCCAC
AAGAGATAATGTTCGTACTGTTGATTCATTTATAATGAATTACGGTAAAGGAACACGCTGTCAGTTCAAAAGG
TTATTTATCGACGAAGGTCTGATGTTGCACACTGGTTGTGTGAATTTTCTTGTTTCTATGTCTCTGTGCGAAATT
GCATATGTTTATGGAGACACACAACAAATTCCATACATCAACAGAGTATCCGGTTTTCCGTACCCTGCACATTT
TGCAAAAATAGAGGTTGATGAGGTGGAAACTCGCAGAACTACGCTGCGTTGTCCAGCCGACATTACCCACTAT
CTTAACAGAAGGTACGAAGGACATGTCATGTGTACATCGTCGGTTAAAAAGTCAGTTTCTCAGGAAATGGTGA
GCGGGGCCGCAATGATCAATCCTGTATCTAAGCCATTGAATGGGAAAGTTTTGACTTTCACTCAGTCTGATAA
AGAGGCGCTGCTTTCTCGAGGATATACGGACGTCCATACAGTACATGAGGTACAAGGTGAGACATATGCAGA
TGTGTCGTTGGTCAGATTGACTCCGACACCTGTATCTATCATCGCAGGAGATAGTCCGCACGTTCTCGTAGCTT
TGTCAAGGCATACCCAAACATTGAAGTATTACACCGTAGTGATGGATCCTCTTGTAAGTATAATTAGGGATTTA
GAAAAACTTAGTTCTTACTTGTTAGATATGTATAAAGTAGATGCAGGGACCCAATAGCAATTACAGGTAGACT
CCGTGTTTAAAGGTTCTAATCTTTTTGTTGCAGCACCAAAGACTGGAGATATCTCAGATATGCAATTTTACTAT
GATAAGTGTCTCCCAGGTAATAGCACCATGTTAAATAACTATGATGCTGTTACCATGAGGTTGACTGACATTTC
TCTTAATGTCAAAGATTGCATATTGGATTTCTCTAAGTCTGTGGCTGCACCGAAGGATCCGATCAAACCACTGA
TTCCAATGGTACGAACGGCGGCAGAAATGCCACGCCAGACTGGACTATTGGAAAATTTGGTGGCGATGATCA
AAAGAAACTTTAATTCACCGGAGTTATCAGGAATAATCGACATTGAGAATACTGCATCTTTAGTAGTAGATAA
ATTTTTTGATAGTTACTTGCTTAAAGAAAAAAGAAAACCAAATAAAAATGTTTCTTTATTTTGTAGAGAGTCTCT
CAATAGATGGTTAGAGAAGCAGGAGCAAGTGACCATTGGTCAGCTTGCAGATTTTGATTTTGTGGATCTTCCT
GCCGTTGATCAGTACAGGCATATGATTAAAGCGCAACCTAAGCAGAAGCTGGATACATCAATTCAAAGCGAAT
ATCCGGCCTTGCAGACGATTGTGTATCATTCGAAAAAGATCAACGCAATCTTCGGTCCTTTGTTCAGTGAGCTC
ACAAGGCAAATGCTCGAAAGCATAGACTCAAGTAAGTTTTTGTTCTTTACAAGGAAGACGCCAGCTCAAATTG
AGGATTTCTTCGGAGATCTCGATAGCCATGTCCCTATGGATATCTTGGAGTTGGATATTTCGAAGTATGACAAA
TCTCAGAACGAGTTCCACTGTGCAGTAGAGTATGAAATATGGAGAAGACTTGGATTAGAAGATTTTCTGGGA
GAAGTTTGGAAACAAGGCCACAGGAAAACTACTCTTAAAGATTACACAGCTGGTATTAAAACGTGTTTATGGT
ACCAGAGAAAGAGTGGGGACGTTACAACATTCATCGGTAATACGGTGATTATTGCTGCTTGTTTAGCTTCCAT
GTTGCCCATGGAGAAAATAATCAAAGGTGCATTTTGCGGAGATGACAGTTTACTATACTTCCCAAAAGGTTGT
GAGTTTCCTGACATACAGCATACAGCCAACCTTATGTGGAATTTCGAGGCTAAGCTATTCAGAAAGCAGTATG
GTTATTTCTGTGGAAGGTACGTGATACATCATGACAGAGGGTGTATTGTTTATTATGACCCTTTGAAGTTGATT
TCTAAACTTGGTGCTAAACACATCAAGGATTGGGATCACTTAGAAGAGTTCAGAAGATCCCTTTGTGATGTTG
CAAATTCGTTGAACAACTGTGCGTATTACACGCAGTTGGACGACGCTGTGAGTGAGGTCCATAAAACCGCACC
CCCGGGTTCGTTTGTATATAAAAGTTTAGTTAAATATCTGTCCGATAAGGTTCTTTTTAGAAGTTTGTTTATAGA
TGGCTCTTGTTAAGGGTAAAGTCAATATTAATGAGTTCATAGACTTGTCAAAATCAGAAAAATTTCTTCCGTCT
ATGTTCACACCTGTTAAGAGTGTCATGATCTCCAAGGTTGATAAGATATTGGTTCATGAAGATGAATCTTTGTC
CGAAGTCAATTTACTCAAAGGTGTAAAACTCATTGATGGTGGCTATGTACATCTTGCTGGTCTTGTGGTGACA
GGTGAATGGAATTTGCCAGATAATTGTCGTGGTGGTGTCAGTGTCTGTTTGGTCGATAAGAGAATGGAGAGA
GCGGACGAGGCAACTCTTGCTTCATACTATACCGCAGCGGCTAAGAAAAGGTTTCAGTTCAAAGTCGTTCCAA
ATTACAACATCACTACCAAGGACGCAGAAAAGGCAGTTTGGCAAGTACTAGTTAATATTAGAAATGTTAAAAT
TGCTGCGGGTTACTGTCCGCTGTCATTAGAATTTGTGTCAGTGTGTATTGTTTATAAAAATATTATAAAACTCG
GTTTGAGAGAGAAAATTACGAGCGTCACGGATGGAGGGCCCATGGAACTATCAGAAGAAGTTGTTGATGAG
TTCATGGAAGAAGTCCCGATGTCTGTAAGGCTTGCAAAATTTCGTTCGAAGACCGGAAAAAAGTTTAGTAGTA

AAAGTGAGAATAATAGTGGTAATAATAGGCCGAAACCAAACAAAAACCAAAGGAAGGAAAAGGGTTTAAAA
GTTAGGGTTGAGAAGGATAATTTAATTGATAATGAATTGGAGACTTACGTCGCCGATTCAGATTCGTATTAAA
TATGTCTTACACAATCGCAACTCCATCGCAATTTGTGTTTTTGTCATCAGCATGGGCCGACCCTATAGAATTAAT
AAATTTATGTACTAATTCACTAGGTAATCAGTTCCAAACACAACAAGCTAGAACAACCGTTCAACGGCAATTTA
GCGAAGTGTGGAAACCTGTCCCTCAAGTCACTGTTAGGTTTCCTGACAGTGGTTTTAAGGTGTATAGGTACAA
TGCGGTACTAGATCCTCTAGTTACTGCTTTGTTAGGAGCTTTCGATACTAGAAATAGGATTATAGAAGTCGAAA
ATCAGGCGAACCCGACAACCGCCGAAACGTTAGACGCTACTCGTAGAGTAGATGACGCAACGGTGGCTATAA
GGAGCGCTATAAATAATTTAGTAGTAGAATTGGTCAAAGGAACAGGTTTGTACAATCAGAGCACATTTGAAA
GTGCATCCGGTTTACAATGGTCCTCTGCACCTGCATCTTGA

MK133095

ATGGCATACACACAGACAGCTACCACATCCGCTTTGCTCGACACTGTCCGAGGTAACAATACCTTGGTCAACG
ATCTTGCGAAGCGGCGTCTTTATGACACAGCGGTCGACGAGTTCAACGCTCGTGATCGCAGGCCCAAAGTAA
ATTTTTCCAAAGTAATAAGTGAGGAACAGACGCTTATTGCTACTAGGGCATATCCAGAATTCCAGATAACCTTC
TATAATACGCAGAACGCCGTGCATTCGCTTGCCGGTGGACTACGATCCTTAGAACTGGAATATCTAATGATGC
AGATCCCGTACGGATCACTCACATATGATATAGGTGGGAATTTTGCATCTCATCTGTTCAAAGGACGGGCATA
TGTTCACTGCTGTATGCCCAATCTTGATGTCCGCGACATAATGCGGCACGAAGGCCAGAAAGACAGTATAGAA
TTATACCTTTCCAGGCTTGAGCGGGGCAACAAAGTTGTCCCAAATTTCCAAAAGGAAGCTTTTGACAGATACG
CTGAAACGCCAGACGAAGTTGTCTGTCACAGTACCTTCCAAACGTGTACTCACCAGCAGGTGGAAAACACAG
GCAGGGTGTATGCTATTGCACTGCACAGTATATACGATATACCTGCTGATGAATTCGGAGCGGCACTTTTAAG
GAAAAATGTCCATGTTTGTTACGCCGCCTTCCACTTTTCCGAGAATTTACTTCTCGAAGATTCACATGTCAACCT
TGACGAAATCAATGCGTGTTTTTCGCGTGATGGAGACAAGCTGACTTTTTCTTTCGCATCTGAGAGCACTTTAA
ATTATTGTCATAGTTATTCTAATATTTTAAAATACGTGTGCAAAACTTACTTCCCGGCATCTAATAGAGAGGTCT
ACATGAAGGAGTTTTTGGTCACCAGGGTTAACACCTGGTTTTGTAAGTTTTCTAGGATAGATACTTTTTTATTAT
ACAAGGGGGTAGCCCACAAAGGTGTAAATAGTGAGCAATTTTACAGCGCAATGGAAGATGCATGGCACTACA
AAAAGACTCTTGCAATGTGTAACAGCGAGAGGATTCTTCTTGAAGATTCCTCATCGGTCAATTACTGGTTCCCA
AAAATGAGAGATATGGTCATAGTTCCTCTATTCGACATATCTCTCGACACCAGTAAAAGGACCCGCAAAGAAG
TCTTAGTGTCAAAGGATTTTGTATTCACAGTTTTAAATCACATTCGCACTTATCAAGCCAAGGCACTTACATACT
CCAATGTTTTATCCTTTGTCGAATCAATTCGTTCAAGGGTAATTATCAACGGAGTGACTGCCAGGTCTGAGTGG
GATGTTGACAAATCTCTTTTGCAATCCTTGTCCATGACATTTTTCTTGCATACTAAGCTTGCCGTTTTAAAAGAC
GAATTGTTAATCAGCAAGTTTAGTTTGGGGCCAAAATCAGTAAGCCAGCATGTATGGGATGAGATTTCCCTGG
CTTTTGGAAACGCATTTCCATCGATCAAGGAGAGACTGCTAAATCGGAAACTAATTAAAGTGTCGGGAGACGC
ATTAGAAATCAGGGTGCCTGATTTATATGTGACTTTTCACGATAGATTAGTGACTGAGTACAAAACATCGGTG
GATATGCCAGTGCTTGATATCAGAAAGAGAATGGAGGAGACTGAGGTTATGTACAATGCATTGTCTGAGCTA
TCTGTGCTCAAGGAGTCGGACAAGTTCGACGTTGATGTTTTTTCCCGGATGTGCCAGACTTTGGAGGTAGACC
CAATGACTGCAGCAAAGGTTATTGTGGCAGTGATGAGCAACGAGAGCGGACTGACTCTTACATTCGAACAGC
CAACTGAAGCAAATGTCGCATTGGCACTTAAAGATTCAGAAAAAGCCTCTGAGGGTGCACTAGTGGTTACTTC
TAGAGATGTTGAAGAACCATCCATGAAGGGTTCAATGGCAAGAGGAGAGTTACAATTGGCCGGTCTGTCTGG
AGACCAACCAGAGTCTTCCTATACTCGGAACGAGGAAATAGAGTCATTAGAGCAATTCCACATGGCAACGGCT
AGTTCGTTAATTCGGAAACAGATGAGTTCGATTGTGTACACGGGCCCCATTAAAGTTCAGCAAATGAAAAACT
TTATTGATAGCCTGGTAGCATCACTCTCTGCTGCGGTGTCGAACCTAGTCAAGATCCTAAAGGATACAGCTGCT

ATAGATTTCGAAACCCGTCAGAAGTTTGGAGTCTTAGATGTTGCGACCAAAAGATGGTTAATTAAACCTTTAG
CCAAGAATCACGCATGGGGCGTTATTGAAACACATGCTAGGAAGTACCACGTTGCACTTTTGGAGTATGATGA
GCATGGAGTGGTAACTTGCGACAGTTGGAGAAGGGTGGCCGTGAGTTCTGAGTCAATGGTTTATTCTGATAT
GGCAAAGCTCAGAACACTGAGGAGATTATTAAGAGATGGTGAGCCTCATGTCAGCAGTGCTAAAGTCGTCCT
AGTTGACGGTGTCCCGGGTTGTGGAAAGACAAAAGAGATTCTCTCGAAAGTAAATTTTGAGGAAGATCTAAT
CTTAGTACCGGGTAAGCAGGCTGCTGAAATGATAAAGAGGCGTGCTAATGCGTTAGGAATAATTCAAGCCAC
AAGAGATAATGTTCGTACTGTTGATTCATTTATAATGAATTACGGTAAAGGAACACGCTGTCAGTTCAAAAGG
TTATTTATCGACGAAGGTCTGATGTTGCACACTGGTTGTGTGAATTTTCTTGTTTCTATGTCTCTGTGCGAAATT
GCATATGTTTATGGAGACACACAACAAATTCCATACATCAACAGAGTATCCGGTTTTCCGTACCCTGCACATTT
TGCAAAAATAGAGGTTGATGAGGTGGAAACTCGCAGAACTACGCTGCGTTGTCCAGCCGACATTACCCACTAT
CTTAACAGAAGGTACGAAGGACATGTCATGTGTACATCGTCGGTTAAAAAGTCAGTTTCTCAGGAAATGGTGA
GCGGGGCCGCAATGATCAATCCTGTATCTAAGCCATTGAATGGGAAAGTTTTGACTTTCACTCAGTCTGATAA
AGAGGCGCTGCTTTCTCGAGGATATACGGACGTCCATACAGTACATGAGGTACAAGGTGAGACATATGCAGA
TGTGTCGTTGGTCAGATTGACTCCGACACCTGTATCTATCATCGCAGGAGATAGTCCGCACGTTCTCGTAGCTT
TGTCAAGGCATACCCAAACATTGAAGTATTACACCGTAGTGATGGATCCTCTTGTAAGTATAATTAGGGATTTA
GAAAAACTTAGTTCTTACTTGTTAGATATGTATAAAGTAGATGCAGGGACCCAATAGCAATTACAGGTAGACT
CCGTGTTTAAAGGTTCTAATCTTTTTGTTGCAGCACCAAAGACTGGAGATATCTCAGATATGCAATTTTACTAT
GATAAGTGTCTCCCAGGTAATAGCACCATGTTAAATAACTATGATGCTGTTACCATGAGGTTGACTGACATTTC
TCTTAATGTCAAAGATTGCATATTGGATTTCTCTAAGTCTGTGTCTGCACCGAAGGATCCGATCAAACCACTGA
TTCCAATGGTACGAACGGCGGCAGAAAAGCCACGCCAGACTGGACTATTGGAAAATTTGGTGGCGATGATCA
AAAGAAACTTTAATTCACCGGAGTTATCAGGAATAATCGACATTGAGAATACTGCATCTTTAGTAGTAGATAA
ATTTTTTGATAGTTACTTGCTTAAAGAAAAAAGAAAACCAAATAAAAATGTTTCTTTATTTTGTAGAGAGTCTCT
CAATAGATGGTTAGAGAAGCAGGAGCAAGTGACCATTGGTCAGCTTGCAGATTTTGATTTTGTGGATCTTCCT
GCCGTTGATCAGTACAGGCATATGATTAAAGCGCAACCTAAGCAGAAGCTGGATACATCAATTCAAAGCGAAT
ATCCGGCCTTGCAGACGATTGTGTATCATTCGAAAAAGATCAACGCAATCTTCGGTCCTTTGTTCAGTGAGCTC
ACAAGGCAAATGCTCGAAAGCATAGACTCAAGTAAGTTTTTGTTCTTTACAAGGAAGACGCCAGCTCAAATTG
AGGATTTCTTCGGAGATCTCGATAGCCATGTCCCTATGGATATCTTGGAGTTGGATATTTCGAAGTATGACAAA
TCTCAGAACGAGTTCCACTGTGCAGTAGAGTATGAAATATGGAGAAGACTTGGATTAGAAGATTTTCTGGGA
GAAGTTTGGAAACAAGGCCACAGGAAAACTACTCTTAAAGATTACACAGCTGGTATTAAAACGTGTTTATGGT
ACCAGAGAAAGAGTGGGGACGTTACAACATTCATCGGTAATACGGTGATTATTGCTGCTTGTTTAGCTTCCAT
GTTGCCCATGGAGAAAATAATCAAAGGTGCATTTTGCGGAGATGACAGTTTACTATACTTCCCAAAAGGTTGT
GAGTTTCCTGACATACAGCATACAGCCAACCTTATGTGGAATTTCGAGGCTAAGCTATTCAGAAAGCAGTATG
GTTATTTCTGTGGAAGGTACGTGATACATCACGACAGAGGGTGTATTGTTTATTATGACCCTTTGAAGTTGATT
TCTAAACTTGGTGCTAAACACATCAAGGATTGGGATCACTTAGAAGAGTTCAGAAGATCCCTTTGTGATGTTG
CAAATTCGTTGAACAACTGTGCGTATTACACGCAGTTGGACGACGCTGTGAGTGAGGTCCATAAAACCGCACC
CCCGGGTTCGTTTGTATATAAAAGTTTAGTTAAATATCTGTCCGATAAGGTTCTTTTTAGAAGTTTGTTTATAGA
TGGCTCTTGTTAAGGGTAAAGTCAATATTAATGAGTTCATAGACTTGTCAAAATCAGAAAAATTTCTTCCGTCT
ATGTTCACACCTGTTAAGAGTGTCATGATCTCCAAGGTTGATAAGATATTGGTTCATGAAGATGAATCTTTGTC
CGAAGTCAATTTACTCAAAGGTGTAAAACTCATTGATGGTGGCTATGTACATCTTGCTGGTCTTGTGGTGACA
GGTGAATGGAATTTGCCAGATAATTGTCGTGGTGGTGTCAGTGTCTGTTTGGTCGATAAGAGAATGGAGAGA
GCGGACGAGGCAACTCTTGCTTCATACTATACCGCAGCGGCTAAGAAAAGGTTTCAGTTCAAAGTCGTTCCAA
ATTACAACATCACTACCAAGGACGCAGAAAAGGCAGTTTGGCAAGTACTAGTTAATATTAGAAATGTTAAAAT
TGCTGCGGGTTACTGTCCGCTGTCATTAGAATTTGTGTCAGTGTGTATTGTTTATAAAAATATTATAAAACTCG
GTTTGAGAGAGAAAATTACGAGCGTCACGGATGGAGGGCCTATGGAACTATCAGAAGAAGTTGTTGATGAGT
TCATGGAAGAAGTCCCGATGTCTGTAAGGCTTGCAAAATTTCGTTCGAAGACCGGAAAAAAGTTTAGTAGTAA
AAGTGAGAATAATAGTGGTAATAATAGGCCGAAACCAAACAAAAACCAAAGGAAGGAAAAGGGTTTAAAAG
TTAGGGTTGAGAAGGATGATTTAATTGATAATGAATTGGAGACTTACGTCGCCGATTCAGATTCGTATTAAAT
ATGTCTTACACAATCGCAACTCCATCGCAATTTGTGTTTTTGTCATCAGCATGGGCCGACCCTATAGAATTAATA

AATTTATGTACTAATTCACTAGGTAATCAGTTCCAAACACAACAAGCTAGAACAACCGTTCAACGGCAATTTAG
CGAAGTGTGGAAACCTGTCCCTCAAGTCACTGTTAGGTTTCCTGACAGTGGTTTTAAGGTGTATAGGTACAAT
GCGGTACTAGATCCTCTAGTTACTGCTTTGTTAGGAGCTTTCGATACTAGAAATAGGATTATAGAAGTCGAAA
ATCAGGCGAACCCGACAACCGCCGAAACGTTAGACGCTACTCGTAGAGTAGATGACGCAACGGTGGCTATAA
GGAGCGCTATAAATAATTTAGTAGTAGAATTGGTCAAAGGAACAGGTTTGTACAATCAGAGCACATTTGAAA
GTGCATCCGGTTTACAATGGTCCTCTGCACCTGCATCTTGA

MK165457

ATGGCATACACACAGACAGCTACCACATCCGCTTTGCTCGACACTGTCCGAGGTAACAATACCTTGGTCAACG
ATCTTGCGAAGCGGCGTCTTTATGACACAGCGGTCGACGAGTTCAACGCTCGTGATCGCAGGCCCAAAGTAA
ATTTTTCCAAAGTAATAAGTGAGGAACAGACGCTTATTGCTACTAGGGCATATCCAGAATTCCAGATAACCTTC
TATAATACGCAGAACGCCGTGCATTCGCTTGCCGGTGGACTACGATCCTTAGAACTGGAATATCTAATGATGC
AGATCCCGTACGGATCACTCACATATGATATAGGTGGGAATTTTGCATCTCATCTGTTCAAAGGACGGGCATA
TGTTCACTGCTGTATGCCCAATCTTGATGTCCGCGACATAATGCGGCACGAAGGCCAGAAAGACAGTATAGAA
TTATACCTTTCCAGGCTTGAGCGGGGCAACAAAGTTGTCCCAAATTTCCAAAAGGAAGCTTTTGACAGATACG
CTGAAACGCCAGACGAAGTTGTCTGTCACAGTACCTTCCAAACGTGTACGCACCAGCAGGTGGAAAACACAG
GCAGGGTGTATGCTATTGCATTGCACAGTATATACGATATACCTGCAGATGAATTCGGAGCGGCACTTTTAAG
GAAAAATGTCCATGTTTGTTACGCCGCCTTCCACTTTTCCGAGAATTTACTTCTCGAAGATTCACACGTCAACCT
TGACGAAATCAACGCGTGTTTTTCGCGTGATGGAGACAAGCTGACTTTTTCTTTCGCATCTGAGAGCACTTTAA
ATTATTGTCATAGTTATTCTAATATTTTAAAATACGTGTGCAAAACTTACTTCCCGGCATCTAATAGAGAGGTCT
ACATGAAGGAGTTTTTGGTCACCAGGGTTAACACCTGGTTTTGTAAGTTTTCTAGGATAGATACTTTTTTATTAT
ACAAGGGGGTAGCCCACAAAGGTGTAAATAGTGAGCAATTTTACAGCGCAATGGAAGATGCATGGCACTACA
AAAAGACTCTTGCAATGTGTAACAGCGAGAGGATTCTTCTTGAAGATTCCTCATCGGTCAATTACTGGTTCCCA
AAAATGAGAGATATGGTCATAGTTCCTCTATTCGACATATCTCTCGACACCAGTAAAAGGACCCGCAAAGAAG
TCTTAGTGTCAAAGGATTTTGTATTCACAGTTTTAAATCACATTCGCACTTATCAAGCCAAGGCACTTACATACT
CCAATGTTTTATCCTTTGTCGAATCAATTCGTTCAAGGGTAATTATCAACGGAGTGACTGCCAGGTCTGAGTGG
GATGTTGACAAATCTCTTTTGCAATCCTTGTCCATGACATTTTTCTTGCATACTAAGCTTGCCGTTTTAAAAGAC
GAATTGTTAATCAGCAAGTTTAGTTTGGGGCCAAAATCAGTAAGCCAGCATGTATGGGATGAGATTTCCCTGG
CTTTTGGAAACGCATTTCCATCGATCAAGGAGAGACTGCTAAATCGGAAACTAATTAAAGTGTCGGGAGACGC
ATTAGAAATCAGGGTGCCTGATTTATATGTGACTTTTCACGATAGATTAGTGACTGAGTACAAAACATCGGTG
GAAATGCCAGTGCTTGATATCAGAAAGAGAATGGAGGAGACTGAGGTTATGTACAATGCATTGTCTGAGCTA
TCTGTGCTCAAGGAGTCGGACAAGTTCGACGTAGATGTTTTTCCCGGATGTGCCAGACTTTGGAGGTAGACC
CAATGACTGCAGCAAAGGTTATTGTGGCAGTGATGAGCAACGAGAGCGGACTGACTCTTACATTCGAACAGC
CAACTGAAGCAAATGTCGCATTGGCACTTAAAGATTCAGAAAAAGCCTCTGAGGGTGCACTAGTGGTTACTTC
TAGAGATGTTGAAGAACCATCCATGAAGGGTTCAATGGCAAGAGGAGAGTTACAATTGGCCGGTCTGTCTGG
AGACCAACCAGAGTCTTCCTATACTCGGAACGAGGAAATAGAGTCATTAGAGCAATTCCACATGGCAACGGCT
AGTTCGTTAATTCGGAAACAGATGAGTTCGATTGTGTACACGGGCCCCATTAAAGTTCAGCAAATGAAAAACT
TTATTGATAGCCTGGTAGCATCACTCTCTGCTGCGGTGTCGAACCTAGTCAAGATCCTAAAGGATACAGCTGCT
ATAGATCTCGAAACCCGTCAGAAGTTTGGAGTCTTAGATGTTGCGACCAAAAGATGGTTAATTAAACCTTTAG
CCAAGAATCACGCATGGGGCGTTATTGAAACACATGC-
AGGAAGTACCACGTTGCACTTTTGGAGTATGATGAGCATGGAGTGGTAACTTGCGACAGTTGGAGAAGGGTG
GCCGTGAGTTCTGAGTCAATGGTTTATTCTGATATGGCAAAGCTCAGAACACTGAGGAGATTATTAAGAGATG
GAGAGCCTCATGTCAGCAGTGCTAAAGTCGTCCTAGTTGACGGTGTCCCGGGTTGTGGAAAGACAAAAGAGA
TTCTCTCGAAAGTAAATTTTGAGGAAGATCTAATCTTAGTACCGGGTAAGCAGGCTGCTGAAATGATAAAGAG
GCGTGCTAATGCGTCAGGAATAATTCAAGCCACAAGAGATAATGTTCGTACTGTTGATTCATTTATAATGAATT
ACGGTAAAGGAACACGCTGTCAGTTCAAAAGGTTATTTATCGACGAAGGTCTGATGTTGCACACTGGTTGTGT

GAATTTTCTTGTTTCTATGTCTCTGTGCGAAATTGCATATGTTTATGGAGACACACAACAAATTCCATACATCAA
CAGAGTATCCGGTTTTCCGTACCCTGCACATTTTGCAAAAATAGAGGTTGATGAGGTGGAAACTCGCAGAACT
ACGCTGCGTTGTCCAGCCGACATTACCCACTATCTTAACAGAAGGTACGAAGGACATGTCATGTGTACATCGT
CGGTTAAAAAGTCAGTTTCTCAGGAAATGGTGAGCGGGGCCGCAATGATCAATCCTGTATCTAAGCCATTGAA
TGGGAAAGTTTTGACTTTCACTCAGTCTGATAAAGAGGCGCTGCTTTCTCGAGGATATACGGACGTCCATACA
GTACATGAGGTACAAGGTGAGACATATGCAGATGTGTCGTTGGTCAGATTGACTCCGACACCTGTATCTATCA
TCGCAGGAGATAGTCCGCACGTTCTCGTAGCTTTGTCAAGGCATACCCAAACATTGAAGTATTACACCGTAGT
GATGGATCCTCTTGTAAGTATAATTAGGGATTTAGAAAAACTTAGTTCTTACTTGTTAGATATGTATAAAGTAG
ATGCAGGGACCCAATAGCAATTACAGGTAGACTCCGTGTTTAAAGGTTCTAATCTTTTTGTTGCAGCACCAAA
GACTGGAGATATCTCAGATATGCAATTTTACTATGATAAGTGTCTCCCAGGTAATAGCACCATGTTAAATAACT
ATGATGCTGTTACCATGAGGTTGACTGACATTTCTCTTAATGTCAAAGATTGCATATTGGATTTCTCTAAGTCTG
TGGCTGCACCGAAGGATCCGATCAAACCACTGATTCCAATGGTACGAACGGCGGCAGAAATGCCACGCCAGA
CTGGACTATTGGAAAATTTGGTGGCGATGATCAAAAGAAACTTTAATTCACCGGAGTTATCGGGAATAATCGA
CATTGAGAATACTGCATCTTTAGTAGTAGATAAATTTTTTGATAGTTACTTGCTTAAAGAAAAAAGAAAACCAA
ATAAAAATGTTTCTTTATTTTGTAGAGAGTCTCTCAATAGATGGTTAGAGAAGCAGGAGCAAGTGACCATTGG
TCAGCTTGCAGATTTTGATTTTGTGGATCTTCCTGCCGTTGATCAGTACAGGCATATGATTAAAGCGCAACCTA
AGCAGAAGCTGGATACATCAATTCAAAGCGAATATCCGGCCTTGCAGACGATTGTGTATCATTCGAAAAAGAT
CAACGCAATCTTCGGTCCTTTGTTCAGTGAGCTCACAAGGCAAATGCTCGAAAGCATAGACTCAAGTAAGTTTT
TGTTCTTTACAAGGAAGACGCCAGCTCAAATTGAGGATTTCTTCGGAGATCTCGATAGCCATGTCCCTATGGAT
ATCTTGGAGTTGGATATTTCGAAGTATGACAAATCTCAGAACGAGTTCCACTGTGCAGTAGAGTATGAAATAT
GGAGAAGACTTGGATTAGAAGATTTTCTGGGAGAAGTTTGGAAACAAGGCCACAGGAAAACTACTCTTAAAG
ATTACACAGCTGGTATTAAAACGTGTTTATGGTACCAGAGAAAGAGTGGGGACGTTACAACATTCATCGGTAA
TACGGTGATTATTGCTGCTTGTTTAGCTTCCATGTTGCCCATGGAGAAAATAATCAAAGGTGCATTTTGCGGAG
ATGACAGTTTACTATACTTCCCAAAAGGTTGTGAGTTTCCTGACATACAGCATACAGCCAACCTTATGTGGAAT
TTCGAGGCTAAGCTATTCAGAAAGCAGTATGGTTATTTCTGTGGAAGGTACGTGATACATCATGACAGAGGGT
GTATTGTTTATTATGACCCTTTGAAGTTGATTTCTAAACTTGGTGCTAAACACATCAAGGATTGGGATCACTTA
GAAGAGTTCAGAAGATCCCTTTGTGATGTTGCAAATTCGTTGAACAACTGTGCGTATTACACGCAGTTGGACG
ACGCTGTGAGTGAGGTCCATAAAACCGCACCCCCGGGTTCGTTTGTATATAAAAGTTTAGTTAAATATCTGTCC
GATAAGGTTCTTTTTAGAAGTTTGTTTATAGATGGCTCTTGTTAAGGGTAAAGTCAATATTAATGAGTTCATAG
ACTTGTCAAAATCAGAAAAATTTCTTCCGTCTATGTTCACACCTGTTAAGAGTGTCATGATCTCCAAGGTTGAT
AAGATATTGGTTCATGAAGATGAATCTTTGTCCGAAGTCAATTTACTCAAAGGTGTAAAACTCATTGATGGTG
GCTATGTACATCTTGCTGGTCTTGTGGTGACAGGTGAATGGAATTTGCCAGATAATTGTCGTGGTGGTGTCAG
TGTCTGTTTGGTCGATAAGAGAATGGAGAGAGCGGACGAGGCAACTCTTGCTTCATACTATACCGCAGCGGC
TAAGAAAAGGTTTCAGTTCAAAGTCGTTCCAAATTACAACATCACTACCAAGGACGCAGAAAAGGCAGTTTGG
CAAGTACTAGTTAATATTAGAAATGTTAAAATTGCTGCGGGTTACTGTCCGCTGTCATTAGAATTTGTGTCAGT
GTGTATTGTTTATAAAAATATTATAAAACTCGGTTTGAGAGAGAAAATTACGAGCGTCACGGATGGAGGGCCC
ATGGAACTATCAGAAGAAGTTGTTGATGAGTTCATGGAAGAAGTCCCGATGTCTGTAAGGCTTGCAAAATTTC
GTTCGAAGACCGGAAAAAAGTTTAGTAGTAAAAGTGAGAATAATAGTGGTAATAATAGGCCGAAACCAAACA
AAA-
CCAAAGGAAGGAAAAGGGTTTAAAAGTTAGGGTTGAGAAGGATAATTTAATTGATAATGAATTGGAGACTTA
CGTCGCCGATTCAGATTCGTATTAAATATGTCTTACACAATCGCAACTCCATCGCAATTTGTGTTTTTGTCATCA
GCATGGGCCGACCCTATAGAATTAATAAATTTATGTACTAATTCACTAGGTAATCAGTTCCAAACACAACAAGC
TAGAACAACCGTTCAACGGCAATTTAGCGAAGTGTGGAAACCTGTCCCTCAAGTCACTGTTAGGTTTCCTGAC
AGTGGTTTTAAGGTGTATAGGTACAATGCGGTACTAGATCCTCTAGTTACTGCTTTGTTAGGAGCTTTCGATAC
TAGAAATAGGATTATAGAAGTCGAAAATCAGGCGAACCCGACAACCGCCGAAACGTTAGACGCTACTCGTAG
AGTAGATGACGCAACGGTGGCTATAAGGAGCGCTATAAATAATTTAGTAGTAGAATTGGTCAAAGGAACAGG
TTTGTACAATCAGAGCACATTTGAAAGTGCATCCGGTTTACAATGGTCCTCTGCACCTGCATCTTGA

MK319944

ATGGCATACACACAGACAGCTACCACATCCGCTTTGCTCGACACTGTCCGAGGTAACAATACCTTGGTCAACG
ATCTTGCGAAGCGGCGTCTTTATGACACAGCGGTCGACGAGTTCAACGCTCGTGATCGCAGGCCCAAAGTAA
ATTTTTCCAAAGTAATAAGTGAGGAACAGACGCTTATTGCTACTAGGGCATATCCAGAATTCCAGATAACCTTC
TATAATACGCAGAACGCCGTGCATTCGCTTGCCGGTGGACTACGATCCTTAGAACTGGAATATCTAATGATGC
AGATCCCGTACGGATCACTCACATATGATATAGGTGGGAATTTTGCATCTCATCTGTTCAAAGGACGGGCATA
TGTTCACTGCTGTATGCCCAATCTTGATGTCCGCGACATAATGCGGCACGAAGGCCAGAAAGACAGTATAGAA
TTATACCTTTCCAGGCTTGAGCGGGGCAACAAAGTTGTCCCAAATTTCCAAAAGGAAGCTTTTGACAGATACG
CTGAAACGCCAGACGAAGTTGTCTGTCACAGTACCTTCCAAACGTGTACGCACCAGCAGGTGGAAAACACAG
GCAGGGTGTATGCTATTGCATTGCACAGTATATACGATATACCTGCTGATGAATTCGGAGCGGCACTTTTAAG
AAAAAATGTCCATGTTTGTTACGCCGCCTTCCACTTTTCCGAGAATTTACTTCTCGAAGATTCACACGTCAACCT
TGACGAAATCAACGCGTGTTTTTCGCGTGATGGAGACAAGCTGACTTTTTCTTTCGCATCTGAGAGCACTTTAA
ATTATTGTCATAGTTATTCTAATATTTTAAAATACGTGTGCAAAACTTACTTCCCGGCATCTAATAGAGAGGTCT
ACATGAAGGAGTTTTTGGTCACCAGGGTTAACACCTGGTTTTGTAAGTTTTCTAGGATAGATACTTTTTTATTAT
ACAAGGGGGTAGCCCACAAAGGTGTAAATAGTGAGCAATTTTACAGCGCAATGGAAGATGCATGGCACTACA
AAAAGACTCTTGCAATGTGTAACAGCGAGAGGATTCTTCTTGAAGATTCCTCATCGGTCAATTACTGGTTCCCA
AAAATGAGAGATATGGTCATAGTTCCTCTATTCGACATATCTCTCGACACCAGTAAAAGGACCCGCAAAGAAG
TCTTAGTGTCAAAGGATTTTGTATTCACAGTTCTAAATCACATTCGCACTTATCAAGCCAAGGCACTTACATACT
CCAATGTTTTATCCTTTGTCGAATCAATTCGTTCAAGGGTAATTATCAACGGAGTGACTGCCAGGTCTGAGTGG
GATGTTGACAAATCTCTTTTGCAATCCTTGTCCATGACATTTTTCTTGCATACTAAGCTTGCCGTTTTAAAAGAC
GAATTGTTAATCAGCAAGTTTAGTTTGGGGCCAAAATCAGTAAGCCAGCATGTATGGGATGAGATTTCCCTGG
CTTTTGGAAACGCATTTCCATCGATCAAGGAGAGACTGCTAAATCGGAAACTAATTAAAGTGTCGGGAGACGC
ATTAGAAATCAGGGTGCCTGATTTATATGTGACTTTTCACGATAGATTAGTGACTGAGTACAAAACATCGGTG
GATATGCCAGTGCTTGATATCAGAAAGAGAATGGAGGAGACTGAGGTTATGTACAATGCATTGTCTGAGCTA
TCTGTGCTCAAGGAGTCGGACAAGTTCGACGTTGATGTTTTTTCCCGGATGTGCCAGACTTTGGAGGTAGACC
CAATGACTGCAGCAAAGGTTATTGTGGCAGTGATGAGCAACGAGAGCGGACTGACTCTCACATTCGGACAGC
CAACTGAAGCAAATGTCGCATTGGCACTTAAAGATTCAGAAAAAGCCTCTGAGGGTGCACTAGTGGTTACTTC
TAGAGATGTTGAAGAACCATCCATGAAGGGTTCAATGGCAAGAGGAGAGTTACAATTGGCCGGTCTGTCTGG
AGACCAACCAGAGTCTTCCTATACTCGGAACGAGGAAATAGAGTCATTAGAGCAATTCCACATGGCAACGGCT
AGTTCGTTAATTCGGAAACAGATGAGTTCGATTGTGTACACGGGCCCCATTAAAGTTCAGCAAATGAAAAACT
TTATTGATAGCCTGGTAGCATCACTCTCTGCTGCGGTGTCGAACCTAGTCAAGATCCTAAAGGATACAGCTGCT
ATAGATCTCGAAACCCGTCAGAAGTTTGGAGTCTTAGATGTTGCGACCAAAAGATGGTTAATTAAACCTTTAG
CCAAGAATCACGCATGGGGCGTTATTGAAACACATGCTAGGAAGTACCACGTTGCACTTTTGGAGTATGATGA
GCATGGAGTGGTAACTTGCGACAGTTGGAGAAGGGTGGCCGTGAGTTCTGAGTCAATGGTTTATTCTGATAT
GGCAAAGCTCAGAACACTGAGGAGATTATTAAGAGATGGTGAGCCTCATGTCAGCAGTGCTAAAGTCGTCCT
AGTTGACGGTGTCCCGGGTTGTGGAAAGACAAAAGAGATTCTCTCGAAAGTAAATTTTGAGGAAGATCTAAT
CTTAGTACCGGGTAAGCAGGCTGCTGAAATGATAAAGAGGCGTGCTAATGCGTCAGGAATAATTCAAGCCAC
AAGAGATAATGTTCGTACTGTTGATTCATTTATAATGAATTACGGTAAAGGAACACGCTGTCAGTTCAAAAGG
TTATTTATCGACGAAGGTCTGATGTTGCACACTGGTTGTGTGAATTTTCTTGTTTCTATGTCTCTGTGCGAAATT
GCATATGTTTATGGAGACACACAACAAATTCCATACATCAACAGAGTATCCGGTTTTCCGTACCCTGCACATTT
TGCAAAAATAGAGGTTGATGAGGTGGAAACTCGCAGAACTACGCTGCGTTGTCCAGCCGACATTACCCACTAT
CTTAACAGAAGGTACGAAGGACATGTCATGTGTACATCGTCGGTTAAAAAGTCAGTTTCTCAGGAAATGGTGA
GCGGGGCCGCAATGATCAATCCTGTATCTAAGCCATTGAATGGGAAAGTTTTGACTTTCACTCAGTCTGATAA
AGAGGCGCTGCTTTCTCGAGGATATACGGACGTCCATACAGTACATGAGGTACAAGGTGAGACATATGCAGA
TGTGTCGTTGGTCAGATTGACTCCGACACCTGTATCTATTATCGCAGGAGATAGTCCGCACGTTCTCGTAGCTT
TGTCAAGGCATACCCAAACATTGAAGTATTACACCGTAGTGATGGATCCTCTTGTAAGTATAATTAGGGATTTA

GAAAAACTTAGTTCTTACTTGTTAGATATGTATAAAGTAGATGCAGGGACCCAATAGCAATTACAGGTAGACT
CCGTGTTTAAAGGTTCTAATCTTTTTGTTGCAGCACCAAAGACTGGAGATATCTCAGATATGCAATTTTACTAT
GATAAGTGTCTCCCAGGTAATAGCACCATGTTAAATAACTATGATGCTGTTACCATGAGGTTGACTGACATTTC
TCTTAATGTCAAAGATTGCATATTGGATTTCTCTAAGTCTGTGGCTGCACCGAAGGATCCGATCAAACCACTGA
TTCCAATGGTACGAACGGCGGCAGAAATGCCACGCCAGACTGGACTATTGGAAAATTTGGTGGCGATGATCA
AAAGAAACTTTAATTCACCGGAGTTATCAGGAATAATCGACATTGAGAATACTGCATCTTTAGTAGTAGATAA
ATTTTTTGATAGTTACTTGCTTAAAGAAAAAAGAAAACCAAATAAAAATGTTTCTTTATTTTGTAGAGAGTCTCT
CAATAGATGGTTAGAGAAGCAGGAGCAAGTGACCATTGGTCAGCTTGCAGATTTTGATTTTGTGGATCTTCCT
GCCGTTGATCAGTACAGGCATATGATTAAAGCGCAACCTAAGCAGAAGCTGGATACATCAATTCAAAGCGAAT
ATCCGGCCTTGCAGACGATTGTGTATCATTCGAAAAAGATCAACGCAATCTTCGGTCCTTTGTTCAGTGAGCTC
ACAAGGCAAATGCTCGAAAGCATAGACTCAAGTAAGTTTTTGTTCTTTACAAGGAAGACGCCAGCTCAAATTG
AGGATTTCTTCGGAGATCTCGATAGCCATGTCCCTATGGATATCTTGGAGTTGGATATTTCGAAGTATGACAAA
TCTCAGAACGAGTTCCACTGTGCAGTAGAGTATGAAATATGGAGAAGACTTGGATTAGAAGATTTTCTGGGA
GAAGTTTGGAAACAAGGCCACAGGAAAACTACTCTTAAAGATTACACAGCTGGTATTAAAACGTGTTTATGGT
ACCAGAGAAAGAGTGGGGACGTTACAACATTCATCGGTAATACGGTGATTATTGCTGCTTGTTTAGCTTCCAT
GTTGCCCATGGAGAAAATAATCAAAGGTGCATTTTGCGGAGATGACAGTTTACTATACTTCCCAAAAGGTTGT
GAGTTTCCTGACATACAGCATACAGCCAACCTTATGTGGAATTTCGAGGCTAAGCTATTCAGAAAGCAGTATG
GTTATTTCTGTGGAAGGTACGTGATACATCATGACAGAGGGTGTATTGTTTATTATGACCCTTTGAAGTTGATT
TCTAAACTTGGTGCTAAACACATCAAGGATTGGGATCACTTAGAAGAGTTCAGAAGATCCCTTTGTGATGTTG
CAAATTCGTTGAACAACTGTGCGTATTACACGCAGTTGGACGACGCTGTGAGTGAGGTCCATAAAACCGCACC
CCCGGGTTCGTTTGTATATAAAAGTTTAGTTAAATATCTGTCCGATAAGGTTCTTTTTAGAAGTTTGTTTATAGA
TGGCTCTTGTTAAGGGTAAAGTCAATATTAATGAGTTCATAGACTTGTCAAAATCAGAAAAATTTCTTCCGTCT
ATGTTCACACCTGTTAAGAGTGTCATGATCTCCAAGGTTGATAAGATATTGGTTCATGAAGATGAATCTTTGTC
CGAAGTCAATTTACTCAAAGGTGTAAAACTCATTGATGGTGGCTATGTACATCTTGCTGGTCTTGTGGTGACA
GGTGAATGGAATTTGCCAGATAATTGTCGTGGTGGTGTCAGTGTCTGTTTGGTCGATAAGAGAATGGAGAGA
GCGGACGAGGCAACTCTTGCTTCATACTATACCGCAGCGGCTAAGAAAAGGTTTCAGTTCAAAGTCGTTCCAA
ATTACAACATCACTACCAAGGACGCAGAAAAGGCAGTTTGGCAAGTACTAGTTAATATTAGAAATGTTAAAAT
TGCTGCGGGTTACTGTCCGCTGTCATTAGAATTTGTGTCAGTGTGTATTGTTTATAAAAATATTATAAAACTCG
GTTTGAGAGAGAAAATTACGAGCGTCACGGATGGAGGGCCCATGGAACTATCAGAAGAAGTTGTTGATGAG
TTCATGGAAGAAGTCCCGATGTCTGTAAGGCTTGCAAAATTTCGTTCGAAGACCGGAAAAAAGTTTAGTAGTA
AAAGTGAGAATAATAGTGGTAATAATAGGCCGAAACCAAACAAAAACCAAAGGAAGGAAAAGGGTTTAAAA
GTTAGGGTTGAGAAGGATAATTTAATTGATAATGAATTGGAGACTTACGTCGCCGATTCAGATTCGTATTAAA
TATGTCTTACACAATCGCAACTCCATCGCAATTTGTGTTTTTGTCATCAGCATGGGCCGACCCTATAGAATTAAT
AAATTTATGTACTAATTCACTAGGTAATCAGTTCCAAACACAACAAGCTAGAACAACCGTTCAACGGCAATTTA
GCGAAGTGTGGAAACCTGTCCCTCAAGTCACTGTTAGGTTTCCTGACAGTGGTTTTAAGGTGTATAGGTACAA
TGCGGTACTAGATCCTCTAGTTACTGCTTTGTTAGGAGCTTTCGATACTAGAAATAGGATTATAGAAGTCGAAA
ATCAGGCGAACCCGACAACCGCCGAAACGTTAGACGCTACTCGTAGAGTAGATGACGCAACGGTGGCTATAA
GGAGCGCTATAAATAATTTAGTAGTAGAATTGGTCAAAGGAACAGGTTTGTACAATCAGAGCACATTTGAAA
GTGCATCCGGTTTACAATGGTCCTCTGCACCTGCATCTTGA

MN167466

ATGGCATACACACACAGACAGCTACCACATCCGCTTTGCTCGACACTGTCCGAGGTAACAATACCTTGGTCAACG
ATCTTGCGAAGCGGCGTCTTTATGACACAGCGGTCGACGAGTTCAACGCTCGTGATCGCAGGCCCAAAGTAA
ATTTTTCCAAAGTAATAAGTGAGGAACAGACGCTTATTGCTACTAGGGCATATCCAGAATTCCAGATAACCTTC
TATAATACGCAGAACGCCGTGCATTCGCTTGCCGGTGGACTACGATCCTTAGAACTGGAATATCTAATGATGC
AGATCCCGTACGGATCACTCACATATGATATAGGTGGGAATTTTGCATCTCATCTGTTCAAAGGACGGGCATA

TGTTCACTGCTGTATGCCCAATCTTGATGTCCGCGACATAATGCGGCACGAAGGCCAGAAAGACAGTATAGAA
TTATACCTTTCCAGGCTTGAGCGGGGCAACAAAGTTGTCCCAAATTTCCAAAAGGAAGCTTTTGACAGATACG
CTGAAACGCCAGACGAAGTTGTCTGTCACAGTACCTTCCAAACGTGTACTCAGCAGCAGGTGGAAAACACAG
GCAGGGTGTATGCTATTGCACTGTACAGTATATACGATATACCTGCTGATGAATTCGGAGCGGCACTTTTAAG
GAAAAATGTCCATGTTTGTTACGCCGCCTTCCACTTTTCCGAGAATTTACTTCTCGAAGATTCACATGTCAACCT
TGACGAAATCAATGCGTGTTTTTCGCGTGATGGAGACAAGCTGACTTTTTCTTTCGCATCTGAGAGCACTTTAA
ATTATTGTCATAGTTATTCTAATATTTTAAAATACGTGTGCAAAACTTACTTCCCGGCATCTAATAGAGAGGTCT
ACATGAAGGAGTTTTTGGTCACCAGGGTTAACACCTGGTTTTGTAAGTTTTCTAGGATAGATACTTTTTTATTAT
ACAAGGGGGTAGCCCACAAAGGTGTAAATAGTGAGCAATTTTACAGCGCAATGGAAGATGCATGGCACTACA
AAAAGACTCTTGCAATGTGTAACAGCGAGAGGATTCTTCTTGAAGATTCCTCATCGGTCAATTACTGGTTCCCA
AAAATGAGAGATATGGTCATAGTTCCTCTATTCGACATATCTCTCGACACCAGTAAAAGGACCCGCAAAGAAG
TCTTAGTGTCAAAGGATTTTGTATTCACAGTTTTAAATCACATTCGCACTTATCAAGCCAAGGCACTTACATACT
CCAATGTTTTATCCTTTGTCGAATCAATTCGATCAAGGGTAATTATCAACGGAGTGACTGCCAGGTCTGAGTGG
GATGTTGACAAATCTCTTTTGCAATCCTTGTCCATGACATTTTTCTTGCATACTAAGCTTGCCGTTTTAAAAGAC
GAATTGTTAATCAGCAAGTTTAGTTTGGGGCCAAAATCAGTAAGCCAGCATGTATGGGATGAGATTTCCCTGG
CTTTTGGAAACGCATTTCCATCGATCAAGGAGAGACTGCTAAATCGGAAACTAATTAAAGTGTCGGGAGACGC
ATTAGAAATCAGGGTGCCTGATTTATATGTGACTTTTCACGATAGATTAGTGACTGAGTACAAAACATCGGTG
GATATGCCAGTGCTTGATATCAGAAAGAGAATGGAGGAGACTGAGGTTATGTACAATGCATTGTCTGAGCTA
TCTGTGCTCAAGGAGTCGGACAAGTTCGACGTTGATGTTTTTTCCCGGATGTGCCAGACTTTGGAGGTAGACC
CAATGACTGCAGCAAAGGTTATTGTGGCAGTGATGAGCAACGAGAGCGGACTGACTCTTACATTCGAACAGC
CAACTGAAGCAAATGTCGCATTGGCACTTAAAGATTCAGAAAAAGCCTCTGAGGGTGCACTAGTGGTTACTTC
TAGAGATGTTGAAGAACCATCCATGAAGGGTTCAATGGCAAGAGGAGAGTTACAATTGGCCGGTCTGTCTGG
AGACCAACCAGAGTCTTCCTATACTCGGAACGAGGAAATAGAGTCATTAGAGCAATTCCACATGGCAACGGCT
AGTTCGTTAATTCGGAAACAGATGAGTTCGATTGTGTACACGGGCCCCATTAAAGTTCAGCAAATGAAAAACT
TTATTGATAGCCTGGTAGCATCACTCTCTGCTGCGGTGTCGAACCTAGTCAAGATCCTAAAGGATACAGCTGCT
ATAGATCTCGAAACCCGTCAGAAGTTTGGAGTCTTAGATGTTGCGACCAAAAGATGGTTAATTAAACCTTTAG
CCAAGAATCACGCATGGGGCGTTATTGAAACACATGCTAGGAAGTACCACGTTGCACTTTTGGAGTATGATGA
GCATGGAGTGGTAACTTGCGACAGTTGGAGAAGGGTGGCCGTGAGTTCTGAGTCAATGGTTTATTCTGATAT
GGCAAAGCTCAGAACACTGAGGAGATTATTAAGAGATGGTGAGCCTCATGTCAGCAGTGCTAAAGTCGTCCT
AGTTGACGGTGTCCCGGGTTGTGGAAAGACAAAAGAGATTCTCTCGAAAGTAAATTTTGAGGAAGATCTAAT
CTTAGTACCGGGTAAGCAGGCTGCTGAAATGATAAAGAGGCGTGCTAATGCGTCAGGAATAATTCAAGCCAC
AAGAGATAATGTTCGTACTGTTGATTCATTTATAATGAATTACGGTAAAGGAACACGCTGTCAGTTCAAAAGG
TTATTTATCGACGAAGGTCTGATGTTGCACACTGGTTGTGTGAATTTTCTTGTTTCTATGTCTCTGTGCGAAATT
GCATATGTTTATGGAGACACACAACAAATTCCATACATCAACAGAGTATCCGGTTTTCCGTACCCTGCACATTT
TGCAAAAATAGAGGTTGATGAGGTGGAAACTCGCAGAACTACGCTGCGTTGTCCAGCCGACATTACCCACTAT
CTTAACAGAAGGTACGAAGGATATGTCATGTGTACATCGTCGGTTAAAAAGTCAGTTTCTCAGGAAATGGTGA
GCGGGGCCGCAATGATCAATCCTGTATCTAAGCCATTGAATGGGAAAGTTTTGACTTTCACTCAGTCTGATAA
AGAGGCGCTGCTTTCTCGAGGATATACGGACGTCCATACAGTACATGAGGTACAAGGTGAGACATATGCAGA
TGTGTCGTTGGTCAGATTGACTCCGACACCTGTATCTATCATCGCAGGAGATAGTCCGCACGTTCTCGTAGCTT
TGTCAAGGCATACCCAAACATTGAAGTATTACACCGTAGTGATGGATCCTCTTGTAAGTATAATTAGGGATTTA
GAAAAACTTAGTTCTTACTTATTAGATATGTATAAAGTAGATGCAGGGACCCAATAGCAATTACAGGTAGACT
CCGTGTTTAAAGGTTCTAATCTTTTTGTTGCAGCACCAAAGACTGGAGATATCTCAGATATGCAATTTTACTAT
GATAAGTGTCTCCCAGGTAATAGCACCATGTTAAATAACTATGATGCTGTTACCATGAGGTTGACTGACATTTC
TCTTAATGTCAAAGATTGCATATTGGATTTCTCTAAGTCTGTGTCTGCACCGAAGGATCCGATCAAACCACTGA
TTCCAATGGTACGAACGGCGGCAGAAATGCCACGCCAGACTGGACTATTGGAAAATTTGGTGGCGATGATCA
AAAGAAACTTTAATTCACCGGAGTTATCAGGAATAATCGACATTGAGAATACTGCATCTTTAGTAGTAGATAA
ATTTTTTGATAGTTACTTGCTTAAAGAAAAAAGAAAACCAAATAAAAATGTTTCTTTATTTTGTAGAGAGTCTCT
CAATAGATGGTTAGAGAAGCAGGAGCAAGTGACCATTGGTCAGCTTGCAGATTTTGATTTTGTGGATCTTCCT

GCCGTTGATCAGTACAGGCATATGATTAAAGCGCAACCTAAGCAGAAGCTGGATACATCAATTCAAAGCGAAT
ATCCGGCCTTGCAGACGATTGTGTATCATTCGAAAAAGATCAACGCAATCTTCGGTCCTTTGTTCAGTGAGCTC
ACAAGGCAAATGCTCGAAAGCATAGACTCAAGTAAGTTTTTGTTCTTTACAAGGAAGACGCCAGCTCAAATTG
AGGATTTCTTCGGAGATCTCGATAGCCATGTCCCTATGGATATCTTGGAGTTGGATATTTCGAAGTATGACAAA
TCTCAGAACGAGTTCCACTGTGCAGTAGAGTATGAAATATGGAGAAGACTTGGATTAGAAGATTTTCTGGGA
GAAGTTTGGAAACAAGGCCACAGGAAAACTACTCTTAAAGATTACACAGCTGGTATTAAAACGTGTTTATGGT
ACCAGAGAAAGAGTGGGGACGTTACAACATTCATCGGTAATACGGTGATTATTGCTGCTTGTTTAGCTTCCAT
GTTACCCATGGAGAAAATAATCAAAGGTGCATTTTGCGGAGATGACAGTTTACTATACTTCCCAAAAGGTTGT
GAGTTTCCTGACATACAGCATACAGCCAACCTTATGTGGAATTTCGAGGCTAAGCTATTCAGAAAGCAGTATG
GTTATTTCTGTGGAAGGTACGTGATACATCACGACAGAGGGTGTATTGTTTATTATGACCCTTTGAAGTTGATT
TCTAAACTTGGTGCTAAACACATCAAGGATTGGGATCACTTAGAAGAGTTCAGAAGATCCCTTTGTGATGTTG
CAAATTCGTTGAACAACTGTGCGTATTACACGCAGTTGGACGACGCTGTGAGTGAGGTCCATAAAACCGCACC
CCCGGGTTCGTTTGTATATAAAAGTTTAGTTAAATATCTGTCCGATAAGGTTCTTTTTAGAAGTTTGTTTATAGA
TGGCTCTTGTTAAGGGTAAAGTCAATATTAATGAGTTCATAGACTTGTCAAAATCAGAAAAATTTCTTCCGTCT
ATGTTCACACCTGTTAAGAGTGTCATGATCTCCAAGGTTGATAAGATATTGGTTCATGAAGATGAATCTTTGTC
CGAAGTCAATTTACTCAAAGGTGTAAAACTCATTGATGGTGGCTATGTACATCTTGCTGGTCTTGTGGTGACA
GGTGAATGGAATTTGCCAGATAATTGTCGTGGTGGTGTCAGTGTCTGTTTGGTCGATAAGAGAATGGAGAGA
GCGGACGAGGCAACTCTTGCTTCATACTATACCGCAGCGGCTAAGAAAAGGTTTCAGTTCAAAGTCGTTCCAA
ATTACAACATCACTACCAAGGACGCAGAAAAGGCAGTTTGGCAAGTACTAGTTAATATTAGAAATGTTAAAAT
TGCTGCGGGTTACTGTCCGCTGTCATTAGAATTTGTGTCAGTGTGTATTGTTTATAAAAATATTATAAAACTCG
GTTTGAGAGAGAAAATTACGAGCGTCACGGATGGAGGGCCCATGGAACTATCAGAAGAAGTTGTTGATGAG
TTCATGGAAGAAGTCCCGATGTCTGTAAGGCTTGCAAAATTTCGTTCGAAGACCGGAAAAAAGTTTAGTAGTA
AAAGTGAGAATAATAGTGGTAATAATAGGCCGAAACCAGACAAAAACCAAAGGAAGGAAAAGGGTTTAAAA
GTTAGGGTTGAGAAGGATGATTTAATTGATAATGAATTGGAGACTTACGTCGCCGATTCAGATTCGTATTAAA
TATGTCTTACACAATCGCAACTCCATCGCAATTTGTGTTTTTGTCATCAGCATGGGCCGACCCTATAGAATTAAT
AAATTTATGTACTAATTCACTAGGTAATCAGTTCCAAACACAACAAGCTAGAACAACCGTTCAACGGCAATTTA
GCGAAGTGTGGAAACCTGTCCCTCAAGACACTGTTAGGTTTCCTGACAGTGGTTTTAAGGTGTATAGGTACAA
TGCGGTACTAGATCCTCTAGTTACTGCTTTGTTAGGAGCTTTCGATACTAGAAATAGGATTATAGAAGTCGAAA
ATCAGGCGAACCCGACAACCGCCGAAACGTTAGACGCTACTCGTAGAGTAGATGACGCAACGGTGGCTATAA
GGAGCGCTATAAATAATTTAGTAGTAGAATTGGTCAAAGGAACAGGTTTGTACAATCAGAGCACATTTGAAA
GTGCATCCGGTTTACAATGGTCCTCTGCACCTGCATCTTGA

MN549395

ATGGCATACACACAGACAGCTACCACATCCGCTTTGCTCGACACTGTCCGAGGTAACAATACCTTGGTCAACG
ATCTTGCGAAGCGGCGTCTTTATGACACAGCGGTCGACGAGTTCAACGCTCGTGATCGCAGGCCCAAAGTAA
ATTTTTCCAAAGTAATAAGTGAGGAACAGACGCTTATTGCTACTAGGGCATATCCAGAATTCCAGATAACCTTC
TATAATACGCAGAACGCCGTGCATTCGCTTGCCGGTGGACTACGATCCTTAGAACTGGAATATCTAATGATGC
AGATCCCGTACGGATCACTCACATATGATATAGGTGGGAATTTTGCATCTCATCTGTTCAAAGGACGGGCATA
TGTTCACTGCTGTATGCCCAATCTTGATGTCCGCGACATAATGCGGCACGAAGGCCAGAAAGACAGTATAGAA
TTATACCTTTCCAGGCTTGAGCGGGGCAACAAAGTTGTCCCAAATTTCCAAAAGGAAGCTTTTGACAGATACG
CTGAAACGCCAGACGAAGTTGTCTGTCACAGTACCTTCCAAACGTGTACGCACCAGCAGGTGGAAAACACAG
GCAGGGTGTATGCTATTGCATTGCACAGTATATACGATATACCTGCTGATGAATTCGGAGCGGCACTTTTAAG
GAAAAATGTCCATGTTTGTTACGCCGCCTTCCACTTTTCCGAGAATTTACTTCTCGAAGATTCACACGTCAACCT
TGACGAAATCAACGCGTGTTTTTCGCGTGATGGAGACAAGCTGACTTTTTCTTTCGCATCTGAGAGCACTTTAA
ATTATTGTCATAGTTATTCTAATATTTTAAAATACGTGTGCAAAACTTACTTCCCGGCATCTAATAGAGAGGTCT
ACATGAAGGAGTTTTTGGTCACCAGGGTTAACACCTGGTTTTGTAAGTTTTCTAGGATAGATACTTTTTTATTAT

ACAAGGGGGTAGCCCACAAAGGTGTAAATAGTGAGCAATTTTACAGCGCAATGGAAGATGCATGGCACTACA
AAAAGACTCTTGCAATGTGTAACAGCGAGAGGATTCTTCTTGAAGATTCCTCATCGGTCAATTACTGGTTCCCA
AAAATGAGAGATATGGTCATAGTTCCTCTATTCGACATATCTCTCGACACCAGTAAAAGGACCCGCAAAGAAG
TCTTAGTGTCAAAGGATTTTGTATTCACAGTTTTAAATCACATTCGCACTTATCAAGCCAAGGCACTTACATACT
CCAATGTTTTATCCTTTGTCGAATCAATTCGTTCAAGGGTAATTATCAACGGAGTGACTGCCAGGTCTGAGTGG
GATGTTGACAAATCTCTTTTGCAATCCTTGTCCATGACATTTTTCTTGCATACTAAGCTTGCCGTTTTAAAAGAC
GAATTGTTAATCAGCAAGTTTAGTTTGGGGCCAAAATCAGTAAGCCAGCATGTATGGGATGAGATTTCCCTGG
CTTTTGGAAACGCATTTCCATCGATCAAGGAGAGACTGCTAAATCGGAAACTAATTAAAGTGTCGGGAGACGC
ATTAGAAATCAGGGTGCCTGATTTATATGTGACTTTTCACGATAGATTAGTGACTGAGTACAAAACATCGGTG
GATATGCCAGTGCTTGATATCAGAAAGAGAATGGAGGAGACTGAGGTTATGTACAATGCATTGTCTGAGCTA
TCTGTGCTCAAGGAGTCGGACAAGTTCGACGTTGATGTTTTTTCCCGGATGTGCCAGACTTTGGAGGTAGACC
CAATGACTGCAGCAAAGGTTATTGTGGCAGTGATGAGCAACGAGAGCGGACTGACTCTCACATTCGAACAGC
CAACTGAAGCAAATGTCGCATTGGCACTTAAAGATTCAGAAAAAGCCTCTGAGGGTGCACTAGTGGTTACTTC
TAGAGATGTTGAAGAACCATCCATGAAGGGTTCAATGGCAAGAGGAGAGTTACAATTGGCCGGTCTGTCTGG
AGACCAACCAGAGTCTTCCTATACTCGGAACGAGGAAATAGAGTCATTAGAGCAATTCCACATGGCAACGGCT
AGTTCGTTAATTCGGAAACAGATGAGTTCGATTGTGTACACGGGCCCCATTAAAGTTCAGCAAATGAAAAACT
TTATTGATAGCCTGGTAGCATCACTCTCTGCTGCGGTGTCGAACCTAGTCAAGATCCTAAAGGATACAGCTGCT
ATAGATCTCGAAACCCGTCAGAAGTTTGGAGTCTTAGATGTTGCGACCAAAAGATGGTTAATTAAACCTTTAG
CCAAGAATCACGCATGGGGCGTTATTGAAACACATGCTAGGAAGTACCACGTTGCACTTTTGGAGTATGATGA
GCATGGAGTGGTAACTTGCGACAGTTGGAGAAGGGTGGCCGTGAGTTCTGAGTCAATGGTTTATTCTGATAT
GGCAAAGCTCAGAACACTGAGGAGATTATTAAGAGATGGTGAGCCTCATGTCAGCAGTGCTAAAGTCGTCCT
AGTTGACGGTGTCCCGGGTTGTGGAAAGACAAAAGAGATTCTCTCGAAAGTAAATTTTGAGGAAGATCTAAT
CTTAGTACCGGGTAAGCAGGCTGCTGAAATGATAAAGAGGCGTGCTAATGCGTCAGGAATAATTCAAGCCAC
AAGAGATAATGTTCGTACTGTTGATTCATTTATAATGAATTACGGTAAAGGAACACGCTGTCAGTTCAAAAGG
TTATTTATCGACGAAGGTCTGATGTTGCACACTGGTTGTGTGAATTTTCTTGTTTCTATGTCTCTATGCGAAATT
GCATATGTTTATGGAGACACACAACAAATTCCATACATCAACAGAGTATCCGGTTTTCCGTACCCTGCACATTT
TGCAAAAATAGAGGTTGATGAGGTGGAAACTCGCAGAACTACGCTGCGTTGTCCAGCCGACATTACCCACTAT
CTTAACAGAAGGTACGAAGGACATGTCATGTGTACATCGTCGGTTAAAAAGTCAGTTTCTCAGGAAATGGTGA
GCGGGGCCGCAATGATCAATCCTGTATCTAAGCCATTGAATGGGAAAGTTTTGACTTTCACTCAGTCTGATAA
AGAGGCGCTGCTTTCTCGAGGATATACGGACGTCCATACAGTACATGAGGTACAAGGTGAGACATATGCAGA
TGTGTCGTTGGTCAGATTGACTCCGACACCTGTATCTATTATCGCAGGAGATAGTCCGCACGTTCTCGTAGCTT
TGTCAAGGCATACCCAAACATTGAAGTATTACACCGTAGTGATGGATCCTCTTGTAAGTATAATTAGGGATTTA
GAAAAACTTAGTTCTTACTTGTTAGATATGTATAAAGTAGATGCAGGGACCCAATAGCAATTACAGGTAGACT
CCGTGTTTAAAGGTTCTAATCTTTTTGTTGCAGCACCAAAGACTGGAGATATCTCAGATATGCAATTTTACTAT
GATAAGTGTCTCCCAGGTAATAGCACCATGTTAAATAACTATGATGCTGTTACCATGAGGTTGACTGACATTTC
TCTTAATGTCAAAGATTGCATATTGGATTTCTCTAAGTCTGTGGCTGCACCGAAGGATCCGATCAAACCACTGA
TTCCAATGGTACGAACGGCGGCAGAAATGCCACGCCAGACTGGACTATTGGAAAATTTGGTGGCGATGATCA
AAAGAAACTTTAATTCACCGGAGTTATCAGGAATAATCGACATTGAGAATACTGCATCTTTAGTAGTAGATAA
ATTTTTTGATAGTTACTTGCTTAAAGAAAAAAGAAAACCAAATAAAAATGTTTCTTTATTTTGTAGAGAGTCTCT
CAATAGATGGTTAGAGAAGCAGGAGCAAGTGACCATTGGTCAGCTTGCAGATTTTGATTTTGTGGATCTTCCT
GCCGTTGATCAGTACAGGCATATGATTAAAGCGCAACCTAAGCAGAAGCTGGATACATCAATTCAAAGCGAAT
ATCCGGCCTTGCAGACGATTGTGTATCATTCGAAAAAGATCAACGCAATCTTCGGTCCTTTGTTCAGTGAGCTC
ACAAGGCAAATGCTCGAAAGCATAGACTCAAGTAAGTTTTTGTTCTTTACAAGGAAGACGCCAGCTCAAATTG
AGGATTTCTTCGGAGATCTCGATAGCCATGTCCCTATGGATATCTTGGAGTTGGATATTTCGAAGTATGACAAA
TCTCAGAACGAGTTCCACTGTGCAGTAGAGTATGAAATATGGAGAAGACTTGGATTAGAAGATTTTCTGGGA
GAAGTTTGGAAACAAGGCCACAGGAAAACTACTCTTAAAGATTACACAGCTGGTATTAAAACGTGTTTATGGT
ACCAGAGAAAGAGTGGGGACGTTACAACATTCATCGGTAATACGGTGATTATTGCTGCTTGTTTAGCTTCCAT
GTTGCCCATGGAGAAAATAATCAAAGGTGCATTTTGCGGAGATGACAGTTTACTATACTTCCCAAAAGGTTGT

GAGTTTCCTGACATACAGCATACAGCCAACCTTATGTGGAATTTCGAGGCTAAGCTATTCAGAAAGCAGTATG
GTTATTTCTGTGGAAGGTACGTGATACATCATGACAGAGGGTGTATTGTTTATTATGACCCTTTGAAGTTGATT
TCTAAACTTGGTGCTAAACACATCAAGGATTGGGATCACTTAGAAGAGTTCAGAAGATCCCTTTGTGATGTTG
CAAATTCGTTGAACAACTGTGCGTATTACACGCAGTTGGACGACGCTGTGAGTGAGGTCCATAAAACCGCACC
CCCGGGTTCGTTTGTATATAAAAGTTTAGTTAAATATCTGTCCGATAAGGTTCTTTTTAGAAGTTTGTTTATAGA
TGGCTCTTGTTAAGGGTAAAGTCAATATTAATGAGTTCATAGACTTGTCAAAATCAGAAAAATTTCTTCCGTCT
ATGTTCACACCTGTTAAGAGTGTCATGATCTCCAAGGTTGATAAGATATTGGTTCATGAAGATGAATCTTTGTC
CGAAGTCAATTTACTCAAAGGTGTAAAACTCATTGATGGTGGCTATGTACATCTTGCTGGTCTTGTGGTGACA
GGTGAATGGAATTTGCCAGATAATTGTCGTGGTGGTGTCAGTGTCTGTTTGGTCGATAAGAGAATGGAGAGA
GCGGACGAGGCAACTCTTGCTTCATACTATACCGCAGCGGCTAAGAAAAGGTTTCAGTTCAAAGTCGTTCCAA
ATTACAACATCACTACCAAGGACGCAGAAAAGGCAGTTTGGCAAGTACTAGTTAATATTAGAAATGTTAAAAT
TGCTGCGGGTTACTGTCCGCTGTCATTAGAATTTGTGTCAGTGTGTATTGTTTATAAAAATATTATAAAACTCG
GTTTGAGAGAGAAAATTACGAGCGTCACGGATGGAGGGCCCATGGAACTATCAGAAGAAGTTGTTGATGAG
TTCATGGAAGAAGTCCCGATGTCTGTAAGGCTTGCAAAATTTCGTTCGAAGACCGGAAAAAAGTTTAGTAGTA
AAAGTGAGAATAATAGTGGTAATAATAGGCCGAAACCAAACAAAAACCAAAGGAAGGAAAAGGGTTTAAAA
ATTAGGGTTGAGAAGGATAATTTAATTGATAATGAATTGGAGACTTACGTCGCCGATTCAGATTCGTATTAAA
TATGTCTTACACAATCGCAACTCCATCGCAATTTGTGTTTTTGTCATCAGCATGGGCCGACCCTATAGAATTAAT
AAATTTATGTACTAATTCACTAGGTAATCAGTTCCAAACACAACAAGCTAGAACAACCGTTCAACGGCAATTTA
GCGAAGTGTGGAAACCTGTCCCTCAAGTCACTGTTAGGTTTCCTGACAGTGGTTTTAAGGTGTATAGGTACAA
TGCGGTACTAGATCCTCTTGTTACTGCTTTGTTAGGAGCTTTCGATACTAGAAATAGGATTATAGAAGTCGAAA
ATCAGGCGAACCCGACGACCGCCGAAACGTTAGACGCTACTCGTAGAGTAGATGACGCAACGGTGGCTATAA
GGAGCGCTATAAATAATTTAGTAGTAGAATTGGTCAAAGGAACAGGTTTGTACAATCAGAGCACATTTGAAA
GTGCATCCGGTTTACAATGGTCCTCTGCACCTGCATCTTGA

MN549396

ATGGCATACACACAGACAGCTACCACATCCGCTTTGCTCGACACTGTCCGAGGTAACAATACCTTGGTCAACG
ATCTTGCGAAGCGGCGTCTTTATGACACAGCGGTCGACGAGTTCAACGCTCGTGATCGCAGGCCCAAAGTAA
ATTTTTCCAAAGTAATAAGTGAGGAACAGACGCTTATTGCTACTAGGGCATATCCAGAATTCCAGATAACCTTC
TATAATACGCAGAACGCCGTGCATTCGCTTGCCGGTGGACTACGATCCTTAGAACTGGAATATCTAATGATGC
AGATCCCGTACGGATCACTCACATATGATATAGGTGGGAATTTTGCATCTCATCTGTTCAAAGGACGGGCATA
TGTTCACTGCTGTATGCCCAATCTTGATGTCCGCGACATAATGCGGCACGAAGGCCAGAAAGACAGTATAGAA
TTATACCTTTCCAGGCTTGAGCGGGGCAACAAAGTTGTCCCAAATTTCCAAAAGGAAGCTTTTGACAGATACG
CTGAAACGCCAGACGAAGTTGTCTGTCACAGTACCTTCCAAACGTGTACGCACCAGCAGGTGGAAAACACAG
GCAGGGTGTATGCTATTGCATTGCACAGTATATACGATATACCTGCTGATGAATTCGGAGCGGCACTTTTAAG
AAAAAATGTCCATGTTTGTTACGCCGCCTTCCACTTTTCCGAGAATTTACTTCTCGAAGATTCACACGTCAACCT
TGACGAAATCAACGCGTGTTTTTCGCGTGATGGAGACAAGCTGACTTTTTCTTTCGCATCTGAGAGCACTTTAA
ATTATTGTCATAGTTATTCTAATATTTTAAAATACGTGTGCAAAACTTACTTCCCGGCATCTAATAGAGAGGTCT
ACATGAAGGAGTTTTTGGTCACCAGGGTTAACACCTGGTTTTGTAAGTTTTCTAGGATAGATACTTTTTTATTAT
ACAAGGGGGTAGCCCACAAAGGTGTAAATAGTGAGCAATTTTACAGCGCAATGGAAGATGCATGGCACTACA
AAAAGACTCTTGCAATGTGTAACAGCGAGAGGATTCTTCTTGAAGATTCCTCATCGGTCAATTACTGGTTCCCA
AAAATGAGAGATATGGTCATAGTTCCTCTATTCGACATATCTCTCGACACCAGTAAAAGGACCCGCAAAGAAG
TCTTAGTGTCAAAGGATTTTGTATTCACAGTTCTAAATCACATTCGCACTTATCAAGCCAAGGCACTTACATACT
CCAATGTTTTATCCTTTGTCGAATCAATTCGTTCAAGGGTAATTATCAACGGAGTGACTGCCAGGTCTGAGTGG
GATGTTGACAAATCTCTTTTGCAATCCTTGTCCATGACATTTTTCTTGCATACTAAGCTTGCCGTTTTAAAAGAC
GAATTGTTAATCAGCAAGTTTAGTTTGGGGCCAAAATCAGTAAGCCAGCATGTATGGGATGAGATTTCCCTGG
CTTTTGGAAACGCATTTCCATCGATCAAGGAGAGACTGCTAAATCGGAAACTAATTAAAGTGTCGGGAGACGC

ATTAGAAATCAGGGTGCCTGATTTATATGTGACTTTTCACGATAGATTAGTGACTGAGTACAAAACATCGGTG
GATATGCCAGTGCTTGATATCAGAAAGAGAATGGAGGAGACTGAGGTTATGTACAATGCATTGTCTGAGCTA
TCTGTGCTCAAGGAGTCGGACAAGTTCGACGTTGATGTTTTTTCCCGGATGTGCCAGACTTTGGAGGTAGACC
CAATGACTGCAGCAAAGGTTATTGTGGCAGTGATGAGCAACGAGAGCGGACTGACTCTCACATTCGAACAGC
CAACTGAAGCAAATGTCGCATTGGCACTTAAAGATTCAGAAAAAGCCTCTGAGGGTGCACTAGTGGTTACTTC
TAGAGATGTTGAAGAACCATCCATGAAGGGTTCAATGGCAAGAGGAGAGTTACAATTGGCCGGTCTGTCTGG
AGACCAACCAGAGTCTTCCTATACTCGGAACGAGGAAATAGAGTCATTAGAGCAATTCCACATGGCAACGGCT
AGTTCGTTAATTCGGAAACAGATGAGTTCGATTGTGTACACGGGCCCCATTAAAGTTCAGCAAATGAAAAACT
TTATTGATAGCCTGGTAGCATCACTCTCTGCTGCGGTGTCGAACCTAGTCAAGATCCTAAAGGATACAGCTGCT
ATAGATCTCGAAACCCGTCAGAAGTTTGGAGTCTTAGATGTTGCGACCAAAAGATGGTTAATTAAACCTTTAG
CCAAGAATCACGCATGGGGCGTTATTGAAACACATGCTAGGAAGTACCACGTTGCACTTTTGGAGTATGATGA
GCATGGAGTGGTAACTTGCGACAGTTGGAGAAGGGTGGCCGTGAGTTCTGAGTCAATGGTTTATTCTGATAT
GGCAAAGCTCAGAACACTGAGGAGATTATTAAGAGATGGTGAGCCTCATGTCAGCAGTGCTAAAGTCGTCCT
AGTTGACGGTGTCCCGGGTTGTGGAAAGACAAAAGAGATTCTCTCGAAAGTAAATTTTGAGGAAGATCTAAT
CTTAGTACCGGGTAAGCAGGCTGCTGAAATGATAAAGAGGCGTGCTAATGCGTCAGGAATAATTCAAGCCAC
AAGAGATAATGTTCGTACTGTTGATTCATTTATAATGAATTACGGTAAAGGAACACGCTGTCAGTTCAAAAGG
TTATTTATCGACGAAGGTCTGATGTTGCACACTGGTTGTGTGAATTTTCTTGTTTCTATGTCTCTGTGCGAAATT
GCATATGTTTATGGAGACACACAACAAATTCCATACATCAACAGAGTATCCGGTTTTCCGTACCCTGCACATTT
TGCAAAAATAGAGGTTGATGAGGTGGAAACTCGCAGAACTACGCTGCGTTGTCCAGCCGACATTACCCACTAT
CTTAACAGAAGGTACGAAGGACATGTCATGTGTACATCGTCGGTTAAAAAGTCAGTTTCTCAGGAAATGGTGA
GCGGGGCCGCAATGATCAATCCTGTATCTAAGCCATTGAATGGGAAAGTTTTGACTTTCACTCAGTCTGATAA
AGAGGCGCTGCTTTCTCGAGGATATACGGACGTCCATACAGTACATGAGGTACAAGGTGAGACATATGCAGA
TGTGTCGTTGGTCAGATTGACTCCGACACCTGTATCTATTATCGCAGGAGATAGTCCGCACGTTCTCGTAGCTT
TGTCAAGGCATACCCAAACATTGAAGTATTACACCGTAGTGATGGATCCTCTTGTAAGTATAATTAGGGATTTA
GAAAAACTTAGTTCTTACTTGTTAGATATGTATAAAGTAGATGCAGGGACCCAATAGCAATTACAGGTAGACT
CCGTGTTTAAAGGTTCTAATCTTTTTGTTGCAGCACCAAAGACTGGAGATATCTCAGATATGCAATTTTACTAT
GATAAGTGTCTCCCAGGTAATAGCACCATGTTAAATAACTATGATGCTGTTACCATGAGGTTGACTGACATTTC
TCTTAATGTCAAAGATTGCATATTGGATTTCTCTAAGTCTGTGGCTGCACCGAAGGATCCGATCAAACCACTGA
TTCCAATGGTACGAACGGCGGCAGAAATGCCACGCCAGACTGGACTATTGGAAAATTTGGTGGCGATGATCA
AAAGAAACTTTAATTCACCGGAGTTATCAGGAATAATCGACATTGAGAATACTGCATCTTTAGTAGTAGATAA
ATTTTTTGATAGTTACTTGCTTAAAGAAAAAAGAAAACCAAATAAAAATGTTTCTTTATTTTGTAGAGAGTCTCT
CAATAGATGGCTAGAGAAGCAGGAGCAAGTGACCATTGGTCAGCTTGCAGATTTTGATTTTGTGGATCTTCCT
GCCGTTGATCAGTACAGGCATATGATTAAAGCGCAACCTAAGCAGAAGCTGGATACATCAATTCAAAGCGAAT
ATCCGGCCTTGCAGACGATTGTGTATCATTCGAAAAAGATCAACGCAATCTTCGGTCCTTTGTTCAGTGAGCTC
ACAAGGCAAATGCTCGAAAGCATAGACTCAAGTAAGTTTTTGTTCTTTACAAGGAAGACGCCAGCTCAAATTG
AGGATTTCTTCGGAGATCTCGATAGCCATGTCCCTATGGATATCTTGGAGTTGGATATTTCGAAGTATGACAAA
TCTCAGAACGAGTTCCACTGTGCAGTAGAGTATGAAATATGGAGAAGACTTGGATTAGAAGATTTTCTGGGA
GAAGTTTGGAAACAAGGCCACAGGAAAACTACTCTTAAAGATTACACAGCTGGTATTAAAACGTGTTTATGGT
ACCAGAGAAAGAGTGGGGACGTTACAACATTCATCGGTAATACGGTGATTATTGCTGCTTGTTTAGCTTCCAT
GTTGCCCATGGAGAAAATAATCAAAGGTGCATTTTGCGGAGATGACAGTTTACTATACTTCCCAAAAGGTTGT
GAGTTTCCTGACATACAGCATACAGCCAACCTTATGTGGAATTTCGAGGCTAAGCTATTCAGAAAGCAGTATG
GTTATTTCTGTGGAAGGTACGTGATACATCATGACAGAGGGTGTATTGTTTATTATGACCCTTTGAAGTTGATT
TCTAAACTTGGTGCTAAACACATCAAGGATTGGGATCACTTAGAAGAGTTCAGAAGATCCCTTTGTGATGTTG
CAAATTCGTTGAACAACTGTGCGTATTACACGCAGTTGGACGACGCTGTGAGTGAGGTCCATAAAACCGCACC
CCCGGGTTCGTTTGTATATAAAAGTTTAGTTAAATATCTGTCCGATAAGGTTCTTTTTAGAAGTTTGTTTATAGA
TGGCTCTTGTTAAGGGTAAAGTCAATATTAATGAGTTCATAGACTTGTCAAAATCAGAAAAAATTTCTTCCGTCT
ATGTTCACACCTGTTAAGAGTGTCATGATCTCCAAGGTTGATAAGATATTGGTTCATGAAGATGAATCTTTGTC
CGAAGTCAATTTACTCAAAGGTGTAAAACTCATTGATGGTGGCTATGTACATCTTGCTGGTCTTGTGGTGACA

GGTGAATGGAATTTGCCAGATAATTGTCGTGGTGGTGTCAGTGTCTGTTTGGTCGATAAGAGAATGGAGAGA
GCGGACGAGGCAACTCTTGCTTCATACTATACCGCAGCGGCTAAGAAAAGGTTTCAGTTCAAAGTCGTTCCAA
ATTACAACATCACTACCAAGGACGCAGAAAAGGCAGTTTGGCAAGTACTAGTTAATATTAGAAATGTTAAAAT
TGCTGCGGGTTACTGTCCGCTGTCATTAGAATTTGTGTCAGTGTGTATTGTTTATAAAAATATTATAAAACTCG
GTTTGAGAGAGAAAATTACGAGCGTCACGGATGGAGGGCCCATGGAACTATCAGAAGAAGTTGTTGATGAG
TTCATGGAAGAAGTCCCGATGTCTGTAAGGCTTGCAAAATTTCGTTCGAAGACCGGAAAAAAGTTTAGTAGTA
AAAGTGAGAATAATAGTGGTAATAATAGGCCGAAACCAAACAAAAACCAAAGGAAGGAAAAGGGTTTAAAA
GTTAGGGTTGAGAAGGATAATTTAATTGATAATGAATTGGAGACTTACGTCGCCGATTCAGATTCGTATTAAA
TATGTCTTACACAATCGCAACTCCATCGCAATTTGTGTTTTTGTCATCAGCATGGGCCGACCCTATAGAATTAAT
AAATTTATGTACTAATTCACTAGGTAATCAGTTCCAAACACAACAAGCTAGAACAACCGTTCAACGGCAATTTA
GCGAAGTGTGGAAACCTGTCCCTCAAGTCACTGTTAGGTTTCCTGACAGTGGTTTTAAGGTGTATAGGTACAA
TGCGGTACTAGATCCTCTAGTTACTGCTTTGTTAGGAGCTTTCGATACTAGAAATAGGATTATAGAAGTCGAAA
ATCAGGCGAACCCGACAACCGCCGAAACGTTAGACGCTACTCGTAGAGTAGATGACGCAACGGTGGCTATAA
GGAGCGCTATAAATAATTTAGTAGTAGAATTGGTCAAAGGAACAGGTTTGTACAATCAGAGCACATTTGAAA
GTGCATCCGGTTTACAATGGTCCTCTGCACCTGCATCTTGA

MN882011

ATGGCATACACACAGACAGCTACCACATCCGCTTTGCTCGACACTGTCCGAGGTAACAATACCTTGGTCAACG
ATCTTGCGAAGCGGCGTCTTTATGACACAGCGGTCGACGAGTTCAACGCTCGTGATCGCAGGCCCAAAGTAA
ATTTTTCCAAAGTAATAAGTGAGGAACAGACGCTTATTGCTACTAGGGCATATCCAGAATTCCAGATAACCTTC
TATAATACGCAGAACGCCGTGCATTCGCTTGCCGGTGGACTACGATCCTTAGAACTGGAATATCTAATGATGC
AGATCCCGTACGGATCACTCACATATGATATAGGTGGGAATTTTGCATCTCATCTGTTCAAAGGACGGGCATA
TGTTCACTGCTGTATGCCCAATCTTGATGTCCGCGACATAATGCGGCACGAAGGCCAGAAAGACAGTATAGAA
TTATACCTTTCCAGGCTTGAGCGGGGCAACAAAGTTGTCCCAAATTTCCAAAAGGAAGCTTTTGACAGATACG
CTGAAACGCCAGACGAAGTTGTCTGTCACAGTACCTTCCAAACGTGTACGCACCAGCAGGTGGAAAACACAG
GCAGGGTGTATGCTATTGCACTGCACAGTATATACGATATACCTGCTGATGAATTCGGAGCGGCACTTTTAAG
GAAAAATGTCCATGTTTGTTACGCCGCCTTCCACTTTTCCGAGAATTTACTTCTCGAAGATTCACACGTCAACCT
TGACGAAATCAATGCGTGTTTTTCGCGTGATGGAGACAAGCTGACTTTTTCTTTCGCATCTGAGAGCACTTTAA
ATTATTGTCATAGTTATTCTAATATTTTAAAATACGTGTGCAAAACTTACTTCCCGGCATCTAATAGAGAGGTCT
ACATGAAGGAGTTTTTGGTCACCAGGGTTAACACCTGGTTTTGTAAGTTTTCTAGGATAGATACTTTTTTATTAT
ACAAGGGGGTAGCCCACAAAGGTGTAAATAGTGAGCAATTTTACAGCGCAATGGAAGATGCATGGCACTACA
AAAAGACTCTTGCAATGTGTAACAGCGAGAGGATTCTTCTTGAAGATTCCTCATCGGTCAATTACTGGTTCCCA
AAAATGAGAGATATGGTCATAGTTCCTCTATTCGACATATCTCTCGACACCAGTAAAAGGACCCGCAAAGAAG
TCTTAGTGTCAAAGGATTTTGTATTCACAGTTTTAAATCACATTCGCACTTATCAAGCCAAGGCACTTACATACT
CCAATGTTTTATCCTTTGTCGAATCAATTCGTTCAAGGGTAATTATCAACGGAGTGACTGCCAGGTCTGAGTGG
GATGTTGACAAATCTCTTTTGCAATCCTTGTCCATGACATTTTTCTTGCATACTAAGCTTGCCGTTTTAAAAGAC
GAATTGTTAATCAGCAAGTTTAGTTTGGGGCCAAAATCAGTAAGCCAGCACGTATGGGATGAGATTTCCCTGG
CTTTTGGAAACGCATTTCCATCGATCAAGGAGAGACTGCTAAATCGGAAACTAATTAAAGTGTCGGGAGACGC
ATTAGAAATCAGGGTGCCTGATTTATATGTGACTTTTCACGATAGATTAGTGACTGAGTACAAAACATCGGTG
GATATGCCAGTGCTTGATATCAGAAAGAGAATGGAGGAGACTGAGGTTATGTACAATGCATTGTCTGAGCTA
TCTGTGCTCAAGGAGTCGGACAAGTTCGACGTTGATGTTTTTTCCCGGATGTGCCAGACTTTGGAGGTAGACC
CAATGACTGCAGCAAAGGTTATTGTGGCAGTGATGAGCAACGAGAGCGGACTGACTCTTACATTCGAACAGC
CAACTGAAGCAAATGTCGCATTGGCACTTAAAGATTCAGAAAAAGCCTCTGAGGGTGCACTAGTGGTTACTTC
TAGAGATGTTGAAGAACCATCCATGAAGGGTTCAATGGCAAGAGGAGAGTTACAATTGGCCGGTCTGTCTGG
CGACCAACCAGAGTCTTCCTATACTCGGAACGAGGAAATAGAGTCATTAGAGCAATTCCACATGGCAACGGCT
AGTTCGTTAATTCGGAAACAGATGAGTTCGATTGTGTACACGGGCCCCATTAAAGTTCAGCAAATGAAAAACT

TTATTGATAGCCTGGTAGCATCACTCTCTGCTGCGGTGTCGAACCTAGTCAAGATCCTAAAGGATACAGCTGCT
ATAGATCTCGAAACCCGTCAGAAGTTTGGAGTCTTAGATGTTGCGACCAAAAGATGGTTAATTAAACCTTTAG
CCAAGAATCACGCATGGGGCGTTATTGAAACACATGCTAGGAAGTACCACGTTGCACTTTTGGAGTATGATGA
GCATGGAGTGGTAACTTGCGACAGTTGGAGAAGGGTGGCCGTGAGTTCTGAGTCAATGGTTTATTCTGATAT
GGCAAAGCTCAGAACACTGAGGAGATTATTAAGAGATGGTGAGCCTCATGTCAGCAGTGCTAAAGTCGTCCT
AGTTGACGGTGTCCCGGGTTGTGGAAAGACAAAAGAGATTCTCTCGAAAGTAAATTTTGAGGAAGATCTAAT
CTTAGTACCGGGTAAGCAGGCTGCTGAAATGATAAAGAGGCGTGCTAATGCGTCAGGAATAATTCAAGCCAC
AAGAGATAATGTTCGTACTGTTGATTCATTTATAATGAATTACGGTAAAGGAACACGCTGTCAGTTCAAAAGG
TTATTTATCGACGAAGGTCTGATGTTGCACACTGGTTGTGTGAATTTTCTTGTTTCTATGTCTCTGTGCGAAATT
GCATATGTTTATGGAGACACACAACAAATTCCATACATCAACAGAGTATCCGGTTTTCCGTACCCTGCACATTT
TGCAAAAATAGAGGTTGATGAGGTGGAAACTCGCAGAACTACGCTGCGTTGTCCAGCCGACATTACCCACTAT
CTTAACAGAAGGTACGAAGGACATGTCATGTGTACATCGTCGGTTAAAAAGTCAGTTTCTCAGGAAATGGTGA
GCGGGGCCGCAATGATCAATCCTGTATCTAAGCCATTGAATGGGAAAGTTTTGACTTTCACTCAGTCTGATAA
AGAGGCGCTGCTTTCTCGAGGATATACGGACGTCCATACAGTACATGAGGTACAAGGTGAGACATATGCAGA
TGTGTCGTTGGTCAGATTGACTCCGACACCTGTATCTATCATCGCAGGAGATAGTCCGCACGTTCTCGTAGCTT
TGTCAAGGCATACCCAAACATTGAAGTATTACACCGTAGTGATGGATCCTCTTGTAAGTATAATTAGGGATTTA
GAAAAACTTAGTTCTTACTTGTTAGATATGTATAAAGTAGATGCAGGGACCCAATAGCAATTACAGGTAGACT
CCGTGTTTAAAGGTTCTAATCTTTTTGTTGCAGCACCAAAGACTGGAGATATCTCAGATATGCAATTTTACTAT
GATAAGTGTCTCCCAGGTAATAGCACCATGTTAAATAACTATGATGCTGTTACCATGAGGTTGACTGACATTTC
TCTTAATGTCAAAGATTGCATATTGGATTTCTCTAAGTCTGTGGCTGCACCGAAGGATCCGATCAAACCACTGA
TTCCAATGGTACGAACGGCGGCAGAAAAGCCACGCCAGACTGGACTATTGGAAAATTTGGTGGCGATGATCA
AAAGAAACTTTAATTCACCGGAGTTATCAGGAATAATCGACATTGAGAATACTGCATCTTTAGTAGTAGATAA
ATTTTTTGATAGTTACTTGCTTAAAGAAAAAAGAAAACCAAATAAAAATGTTTCTTTATTTTGTAGAGAGTCTCT
CAATAGATGGTTAGAGAAGCAGGAGCAAGTGACCATTGGTCAGCTTGCAGATTTTGATTTTGTGGATCTTCCT
GCCGTTGATCAGTACAGGCATATGATTAAAGCGCAACCTAAGCAGAAGCTGGATACATCAATTCAAAGCGAAT
ATCCGGCCTTGCAGACGATTGTGTATCATTCGAAAAAGATCAACGCAATCTTCGGTCCTTTGTTCAGTGAGCTC
ACAAGGCAAATGCTCGAAAGCATAGACTCAAGTAAGTTTTTGTTCTTTACAAGGAAGGCGCCAGCTCAAATTG
AGGATTTCTTCGGAGATCTCGATAGCCATGTCCCTATGGATATCTTGGAGTTGGATATTTCGAAGTATGACAAA
TCTCAGAACGAGTTCCACTGTGCAGTAGAGTATGAAATATGGAGAAGACTTGGATTAGAAGATTTTCTGGGA
GAAGTTTGGAAACAAGGCCACAGAAAAACTACTCTTAAAGATTATACAGCTGGTATTAAAACGTGTTTATGGT
ACCAGAGAAAGAGTGGGGACGTTACAACATTCATCGGTAATACGGTGATTATTGCTGCTTGTTTAGCATCCAT
GTTACCCATGGAGAAAATAATCAAAGGTGCATTTTGCGGAGATGACAGTTTACTATACTTCCCAAAAGGTTGT
GAGTTTCCTGACATACAGCATACAGCCAACCTTATGTGGAATTTCGAGGCTAAGCTATTCAGAAAGCAGTATG
GTTATTTCTGTGGAAGGTACGTGATACATCACGACAGAGGGTGTATTGTTTATTATGACCCTTTGAAGTTGATT
TCTAAACTTGGTGCTAAACACATCAAGGATTGGGATCACTTAGAAGAGTTCAGAAGATCCCTTTGTGATGTTG
CAAATTCGTTGAACAACTGTGCGTATTACACGCAGTTGGACGACGCTGTGAGTGAGGTCCATAAAACCGCACC
CCCGGGTTCGTTTGTATATAAAAGTTTAGTTAAATATCTGTCCGATAAGGTTCTTTTTAGAAGTTTGTTTATAGA
TGGCTCTTGTTAAGGGTAAAGTCAATATTAATGAGTTCATAGACTTGTCAAAATCAGAAAAAATTTCTTCCGTCT
ATGTTCACACCTGTTAAGAGTGTCATGATCTCCAAGGTTGATAAGATATTGGTTCATGAAGATGAATCTTTGTC
CGAAGTCAATTTACTCAAAGGTGTAAAACTCATTGATGGTGGCTATGTACATCTTGCTGGTCTTGTGGTGACA
GGTGAATGGAATTTGCCAGATAATTGTCGTGGTGGTGTCAGTGTCTGTTTGGTCGATAAGAGAATGGAGAGA
GCGGACGAGGCAACTCTTGCTTCATACTATACCGCAGCGGCTAAGAAAAGGTTTCAGTTCAAAGTCGTTCCAA
ATTACAACATCACTACCAAGGACGCAGAAAAGGCAGTTTGGCAAGTACTAGTTAATATTAGAAATGTTAAAAT
TGCTGCGGGTTACTGTCCGCTGTCATTAGAATTTGTGTCAGTGTGTATTGTTTATAAAAATATTATAAAACTCG
GTTTGAGAGAGAAAATTACGAGCGTCACGGATGGAGGGCCTATGGAACTATCAGAAGAAGTTGTTGATGAGT
TCATGGAAGAAGTCCCGATGTCTGTAAGGCTTGCAAAATTTCGTTCGAAGACCGGAAAAAAGTTTAGTAGTAA
AAGTGAGAATAATAGTGGTAATAATAGGCCGAAACCAAACAAAAACCAAAGGAAGGAAAAGGGTTTAAAAG
TTAGGGTTGAGAAGGATAATTTAATTGATAATGAATTGGAGACTTACGTCGCCGATTCAGATTCGTATTAAAT

ATGTCTTACACAATCGCAACTCCATCGCAATTTGTGTTTTTGTCATCAGCATGGGCCGACCCTATAGAATTAATA
AATTTATGTACTAATTCACTAGGTAATCAGTTCCAAACACAACAAGCTAGAACAACCGTTCAACGGCAATTTAG
CGAAGTGTGGAAACCTGTCCCTCAAGTCACTGTTAGGTTTCCTGACAGTGGTTTTAAGGTGTATAGGTACAAT
GCGGTACTAGATCCTCTAGTTACTGCTTTGTTAGGAGCTTTCGATACTAGAAATAGGATTATAGAAGTCGAAA
ATCAGGCGAACCCGACAACCGCCGAAACGTTAGACGCTACTCGTAGAGTAGATGACGCAACGGTGGCTATAA
GGAGCGCTATAAATAATTTAGTAGTAGAATTGGTCAAAGGAACAGGTTTGTACAATCAGAGCACATTTGAAA
GTGCATCCGGTTTACAATGGTCCTCTGCACCTGCATCTTGA

MN882013

ATGGCATACACACAGACAGCTACCACATCCGCTTTGCTCGACACTGTCCGAGGTAACAATACCTTGGTCAACG
ATCTTGCGAAGCGGCGTCTTTATGACACAGCGGTCGACGAGTTCAACGCTCGTGATCGCAGGCCCAAAGTAA
ATTTTTCCAAAGTAATAAGTGAGGAACAGACGCTTATTGCTACTAGGGCATATCCTGAATTCCAGATAACCTTC
TATAATACGCAGAACGCCGTGCATTCGCTTGCCGGTGGACTACGATCCTTAGAACTGGAATATCTAATGATGC
AGATCCCGTACGGATCACTCACATATGATATAGGTGGGAATTTTGCATCTCATCTGTTCAAAGGACGGGCATA
TGTTCACTGCTGTATGCCCAATCTTGATGTCCGCGACATAATGCGGCACGAAGGCCAGAAAGACAGTATAGAA
TTATACCTTTCCAGGCTTGAGCGGGGCAACAAAGTTGTCCCAAATTTCCAAAAGGAAGCTTTTGACAGATACG
CTGAAACGCCAGACGAAGTTGTCTGTCACAGTACCTTCCAAACGTGTACGCACCAGCAGGTGGAAAACACAG
GCAGGGTGTATGCTATTGCATTGCACAGTATATACGATATACCTGCTGATGAATTCGGAGCGGCACTTTTAAG
GAAAAATGTCCATGTTTGTTACGCCGCCTTCCACTTTTCCGAGAATTTACTTCTCGAAGATTCACACGTCAACCT
TGACGAAATCAACGCGTGTTTTTCGCGTGATGGAGACAAGCTGACTTTTTCTTTCGCATCTGAGAGCACTTTAA
ATTATTGTCATAGTTATTCTAATATTTTAAAATACGTGTGCAAAACTTACTTCCCGGCATCTAATAGAGAGGTCT
ACATGAAGGAGTTTTTGGTCACCAGGGTTAACACCTGGTTTTGTAAGTTTTCTAGGATAGATACTTTTTTATTAT
ACAAGGGGGTAGCCCACAAAGGTGTAAATAGTGAGCAATTTTACAGCGCAATGGAAGATGCATGGCACTACA
AAAAGACTCTTGCAATGTGTAACAGCGAGAGGATTCTTCTTGAAGATTCCTCATCGGTCAATTACTGGTTCCCA
AAAATGAGAGATATGGTCATAGTTCCTCTATTCGACATATCTCTCGACACTAGTAAAAGGACCCGCAAAGAAG
TCTTAGTGTCAAAGGATTTTGTATTCACAGTTTTAAATCACATTCGCACTTATCAAGCCAAGGCACTTACATACT
CCAATGTTTTATCCTTTGTCGAATCAATTCGTTCAAGGGTAATTATCAACGGAGTGACTGCCAGGTCTGAGTGG
GATGTTGACAAATCTCTTTTGCAATCCTTGTCCATGACATTTTTCTTGCATACTAAGCTTGCCGTTTTAAAAGAC
GAATTGTTAATCAGCAAGTTTAGTTTGGGGCCAAAATCAGTAAGCCAGCATGTATGGGATGAGATTTCCCTGG
CTTTTGGAAACGCATTTCCATCGATCAAGGAGAGACTGCTAAATCGGAAACTAATTAAAGTGTCGGGAGACGC
ATTAGAAATCAGGGTGCCTGATTTATATGTGACTTTTCACGATAGATTAGTGACTGAGTACAAAACATCGGTG
GATATGCCAGTGCTTGATATCAGAAAGAGAATGGAGGAGACTGAGGTTATGTACAATGCATTGTCTGAGCTA
TCTGTGCTCAAGGAGTCGGACAAGTTCGACGTTGATGTTTTTTCCCGGATGTGCCAGACTTTGGAGGTAGACC
CAATGACTGCAGCAAAGGTTATTGTGGCAGTGATGAGCAACGAGAGCGGACTGACTCTTACATTCGAACAGC
CAACTGAAGCAAATGTCGCATTGGCACTTAAAGATTCAGAAAAAGCCTCTGAGGGTGCACTAGTGGTTACTTC
TAGAGATGTTGAAGAACCATCCATGAAGGGTTCAATGGCAAGAGGAGAGTTACAATTGGCCGGTCTGTCTGG
AGACCAACCAGAGTCTTCCTATACTCGGAACGAGGAAATAGAGTCATTAGAGCAATTCCACATGGCAACGGCT
GGTTCGTTAATTCGGAAACAGATGAGTTCGATTGTGTACACGGGCCCCATTAAAGTTCAGCAAATGAAAAACT
TTATTGATAGCCTGGTAGCATCACTCTCTGCTGCGGTGTCGAACCTAGTCAAGATCCTAAAGGATACAGCTGCT
ATAGATCTCGAAACCCGTCAGAAGTTTGGAGTCTTAGATGTTGCGACCAAAAGATGGTTAATTAAACCTTTAG
CCAAGAATCACGCATGGGGCGTTATTGAAACACATGCTAGGAAGTACCACGTTGCACTTTTGGAGTATGATGA
GCATGGAGTGGTAACTTGCGACAGTTGGAGAAGGGTGGCCGTGAGTTCTGAGTCAATGGTTTATTCTGATAT
GGCAAAGCTCAGAACACTGAGGAGATTATTAAGAGATGGTGAGCCTCATGTCAGCAGTGCTAAAGTCGTCCT
AGTTGACGGTGTCCCGGGTTGTGGAAAGACAAAAGAGATTCTCTCGAAAGTAAATTTTGAGGAAGATCTAAT
CTTAGTACCGGGTAAGCAGGCTGCTGAAATGATAAAGAGGCGTGCTAATGCGTCAGGAATAATTCAAGCCAC
AAGAGATAATGTTCGTACTGTTGATTCATTTATAATGAATTACGGTAAAGGAACACGCTGTCAGTTCAAAAGG

TTATTTATCGACGAAGGTCTGATGTTGCACACTGGTTGTGTGAATTTTCTTGTTTCTATGTCTCTGTGCGAAATT
GCATATGTTTATGGAGACACACAACAAATTCCATACATCAACAGAGTATCCGGTTTTCCGTACCCTGCACATTT
TGCAAAAATAGAGGTTGATGAGGTGGAAACTCGCAGAACTACGCTGCGTTGTCCAGCCGACATTACCCACTAT
CTTAACAGAAGGTACGAAGGACATGTCATGTGTACATCGTCGGTTAAAAAGTCAGTTTCTCAGGAAATGGTGA
GCGGGGCCGCAATGATCAATCCTGTATCTAAGCCATTGAATGGGAAAGTTTTGACTTTCACTCAGTCTGATAA
AGAGGCGCTGCTTTCTCGAGGATATACGGACGTCCATACAGTACATGAGGTACAAGGTGAGACATATGCAGA
TGTGTCGTTGGTCAGATTGACTCCGACACCTGTATCTATCATCGCAGGAGATAGTCCGCACGTTCTCGTAGCTT
TGTCAAGGCATACCCAAACATTGAAGTATTACACCGTAGTGATGGATCCTCTTGTAAGTATAATTAGGGATTTA
GAAAAACTTAGTTCTTACTTGTTAGATATGTATAAAGTAGATGCAGGGACCCAATAGCAATTACAGGTAGACT
CCGTGTTTAAAGGTTCTAACCTTTTTGTTGCAGCACCAAAGACTGGAGATATCTCAGATATGCAATTTTACTAT
GATAAGTGTCTCCCAGGTAATAGCACCATGTTAAATAACTATGATGCTGTTACCATGAGGTTGACTGACATTTC
TCTTAATGTCAAAGATTGCATATTGGATTTCTCTAAGTCTGTGGCTGCACCGAAGGATCCGATCAAACCACTGA
TTCCAATGGTACGAACGGCGGCAGAAATGCCACGCCAGACTGGACTATTGGAAAATTTGGTGGCGATGATCA
AAAGAAACTTTAATTCACCGGAGTTATCAGGAATAATCGACATTGAGAATACTGCATCTTTAGTAGTAGATAA
ATTTTTTGATAGTTACTTGCTTAAAGAAAAAAGAAAACCAAATAAAAATGTTTCTTTATTTTGTAGAGAGTCTCT
CAATAGATGGTTAGAGAAGCAGGAGCAAGTGACCATTGGTCAGCTTGCAGATTTTGATTTTGTGGATCTTCCT
GCCGTTGATCAGTACAGGCATATGATTAAAGCGCAACCTAAGCAGAAGCTGGATACATCAATTCAAAGCGAAT
ATCCGGCCTTGCAGACGATTGTGTATCATTCGAAAAAGATCAACGCAATCTTCGGTCCTTTGTTCAGTGAGCTC
ACAAGGCAAATGCTCGAAAGCATAGACTCAAGTAAGTTTTTGTTCTTTACAAGGAAGACGCCAGCTCAAATTG
AGGATTTCTTCGGAGATCTCGATAGCCATGTCCCTATGGATATCTTGGAGTTGGATATTTCGAAGTATGACAAA
TCTCAGAACGAGTTCCACTGTGCAGTAGAGTATGAAATATGGAGAAGACTTGGATTAGAAGATTTTCTGGGA
GAAGTTTGGAAACAAGGCCACAGGAAAACTACTCTTAAAGATTACACAGCTGGTATTAAAACGTGTTTATGGT
ACCAGAGAAAGAGTGGGGACGTTACAACATTCATCGGTAATACGGTGATTATTGCTGCTTGTTTAGCTTCCAT
GTTGCCCATGGAGAAAATAATCAAAGGTGCATTTTGCGGAGATGACAGTTTACTATACTTCCCAAAAGGTTGT
GAGTTTCCTGACATACAGCATACAGCCAACCTTATGTGGAATTTCGAGGCTAAGCTATTCAGAAAGCAGTATG
GTTATTTCTGTGGAAGGTACGTGATACATCATGACAGAGGGTGTATTGTTTATTATGACCCTTTGAAGTTGATT
TCTAAACTTGGTGCTAAACACATCAAGGATTGGGATCACTTAGAAGAGTTCAGAAGATCCCTTTGTGATGTTG
CAAATTCGTTGAACAACTGTGCGTATTACACGCAGTTGGACGACGCTGTGAGTGAGGTCCATAAAACCGCACC
CCCGGGTTCGTTTGTATATAAAAGTTTAGTTAAATATCTGTCCGATAAGGTTCTTTTTAGAAGTTTGTTTATAGA
TGGCTCTTGTTAAGGGTAAAGTCAATATTAATGAGTTCATAGACTTGTCAAAATCAGAAAAATTTCTTCCGTCT
ATGTTCACACCTGTTAAGAGTGTCATGATCTCCAAGGTTGATAAGATATTGGTTCATGAAGATGAATCTTTGTC
CGAAGTCAATTTACTCAAAGGTGTAAAACTCATTGATGGTGGCTATGTACATCTTGCTGGTCTTGTGGTGACA
GGTGAATGGAATTTGCCAGATAATTGTCGTGGTGGTGTCAGTGTCTGTTTGGTCGATAAAAGAATGGAGAGA
GCGGACGAGGCAACTCTTGCTTCATACTATACCGCAGCGGCTAAGAAAAGGTTTCAGTTCAAAGTCGTTCCAA
ATTACAACATCACTACCAAGGACGCAGAAAAGGCAGTTTGGCAAGTACTAGTTAATATTAGAAATGTTAAAAT
TGCTGCGGGTTACTGTCCGCTGTCATTAGAATTTGTGTCAGTGTGTATTGTTTATAAAAATATTATAAAACTCG
GTTTGAGAGAGAAAATTACGAGCGTCACGGATGGAGGGCCCATGGAACTATCAGAAGAAGTTGTTGATGAG
TTCATGGAAGAAGTCCCGATGTCTGTAAGGCTTGCAAAATTTCGTTCGAAGACCGGGAAAAAGTTTAGTAGTA
AAAGTGAGAATAATAGTGGTAATAATAGGCCGAAACCAAACAAAAACCAAAGGAAGGAAAAGGGTTTAAAA
GTTAGGGTTGAGAAGGATAATTTAATTGATAATGAATTGGAGACTTACGTCGCCGATTCAGATTCGTATTAAA
TATGTCTTACACAATCGCAACTCCATCGCAATTTGTGTTTTTGTCATCAGCATGGGCCGACCCTATAGAATTAAT
AAATTTATGTACTAATTCACTAGGTAATCAATTCCAAACACAACAAGCTAGAACAACCGTTCAACGGCAATTTA
GCGAAGTGTGGAAACCTGTCCCTCAAGTCACTGTTAGGTTTCCTGACAGTGGTTTTAAGGTGTATAGGTACAA
TGCGGTACTAGATCCTCTAGTTACTGCTTTGTTAGGAGCTTTCGATACTAGAAATAGGATTATAGAAGTCGAAA
ATCAGGCGAACCCGACAACCGCCGAAACGTTAGACGCTACTCGTAGAGTAGATGACGCAACGGTGGCTATAA
GGAGCGCTATAAATAATTTAGTAGTAGAATTGGTCAAAGGAACAGGTTTGTACAATCAGAGCACATTTGAAA
GTGCATCCGGTTTACAATGGTCCTCTGCACCTGCATCTTGA

MN882016

ATGGCATACACACAGACAGCTACCACATCCGCTTTGCTCGACACTGTCCGAGGTAACAATACCTTGGTCAACG
ATCTTGCGAAGCGGCGTCTTTATGACACAGCGGTCGACGAGTTCAACGCTCGTGATCGCAGGCCCAAAGTAA
ATTTTTCCAAAGTAATAAGTGAGGAACAGACGCTTATTGCTACTAGGGCATATCCTGAATTCCAGATAACCTTC
TATAATACGCAGAACGCCGTGCATTCGCTTGCCGGTGGACTACGATCCTTAGAACTGGAATATCTAATGATGC
AGATCCCGTACGGATCACTCACATATGATATAGGTGGGAATTTTGCATCTCATCTGTTCAAAGGACGGGCATA
TGTTCACTGCTGTATGCCCAATCTTGATGTCCGCGACATAATGCGGCACGAAGGCCAGAAAGACAGTATAGAA
TTATACCTTTCCAGGCTTGAGCGGGGCAACAAAGTTGTCCCAAATTTCCAAAAGGAAGCTTTTGACAGATACG
CTGAAACGCCAGACGAAGTTGTCTGTCACAGTACCTTCCAAACGTGTACGCACCAGCAGGTGAAAAACACAG
GCAGGGTGTATGCTATTGCATTGCACAGTATATACGATATACCTGCTGATGAATTCGGAGCGGCACTTTTAAG
GAAAAATGTCCATGTTTGTTACGCCGCCTTCCACTTTTCCGAGAATTTACTTCTCGAAGATTCACACGTCAACCT
TGACGAAATCAACGCGTGTTTTTCGCGTGATGGAGACAAGCTGACTTTTTCTTTCGCATCTGAGAGCACTTTAA
ATTATTGTCATAGTTATTCTAATATTTTAAAATACGTGTGCAAAACTTACTTCCCGGCATCTAATAGAGAGGTCT
ACATGAAGGAGTTTTTGGTCACCAGGGTTAACACCTGGTTTTGTAAGTTTTCTAGGATAGATACTTTTTTATTAT
ACAAGGGGGTAGCCCACAAAGGTGTAAATAGTGAGCAATTTTACAGCGCAATGGAAGATGCATGGCACTACA
AAAAGACTCTTGCAATGTGTAACAGCGAGAGGATTCTTCTTGAAGATTCCTCATCGGTCAATTACTGGTTCCCA
AAAATGAGAGATATGGTCATAGTTCCTCTATTCGACATATCTCTCGACACTAGTAAAAGGACCCGCAAAGAAG
TCTTAGTGTCAAAGGATTTTGTATTCACAGTTTTAAATCACATTCGCACTTATCAAGCCAAGGCACTTACATACT
CCAATGTTTTATCCTTTGTCGAATCAATTCGTTCAAGGGTAATTATCAACGGAGTGACTGCCAGGTCTGAGTGG
GATGTTGACAAATCTCTTTTGCAATCCTTGTCCATGACATTTTTCTTGCATACTAAGCTTGCCGTTTTAAAAGAC
GAATTGTTAATCAGCAAGTTTAGTTTGGGGCCAAAATCAGTAAGCCAGCATGTATGGGATGAGATTTCCCTGG
CTTTTGGAAACGCATTTCCATCGATCAAGGAGAGACTGCTAAATCGGAAACTAATTAAAGTGTCGGGAGACGC
ATTAGAAATCAGGGTGCCTGATTTATATGTGACTTTTCACGATAGATTAGTGACTGAGTACAAAACATCGGTG
GATATGCCAGTGCTTGATATCAGAAAGAGAATGGAGGAGACTGAGGTTATGTACAATGCATTGTCTGAGCTA
TCTGTGCTCAAGGAGTCGGACAAGTTCGACGTTGATGTTTTTTCCCGGATGTGCCAGACTTTGGAGGTAGACC
CAATGACTGCAGCAAAGGTTATTGTGGCAGTGATGAGCAACGAGAGCGGACTGACTCTTACATTCGAACAGC
CAACTGAAGCAAATGTCGCATTGGCACTTAAAGATTCAGAAAAAGCCTCTGAGGGTGCACTAGTGGTTACTTC
TAGAGATGTTGAAGAACCATCCATGAAGGGTTCAATGGCAAGAGGAGAGTTACAATTGGCCGGTCTGTCTGG
AGACCAACCAGAGTCTTCCTATACTCGGAACGAGGAAATAGAGTCATTAGAGCAATTCCACATGGCAACGGCT
GGTTCGTTAATTCGGAAACAGATGAGTTCGATTGTGTACACGGGCCCCATTAAAGTTCAGCAAATGAAAAACT
TTATTGATAGCCTGGTAGCATCACTCTCTGCTGCGGTGTCGAACCTAGTCAAGATCCTAAAGGATACAGCTGCT
ATAGATCTCGAAACCCGTCAGAAGTTTGGAGTCTTAGATGTTGCGACCAAAAGATGGTTAATTAAACCTTTAG
CCAAGAATCACGCATGGGGCGTCATTGAAACACATGCTAGGAAGTACCACGTTGCACTTTTGGAGTATGATGA
GCATGGAGTGGTAACTTGCGACAGTTGGAGAAGGGTGGCCGTGAGTTCTGAGTCAATGGTTTATTCTGATAT
GGCAAAGCTCAGAACACTGAGGAGATTATTAAGAGATGGTGAGCCTCATGTCAGCAGTGCTAAAGTCGTCCT
AGTTGACGGTGTCCCGGGTTGTGGAAAGACAAAAGAGATTCTCTCGAAAGTAAATTTTGAGGAAGATCTAAT
CTTAGTACCGGGTAAGCAGGCTGCTGAAATGATAAAGAGGCGTGCTAATGCGTCAGGAATAATTCAAGCCAC
AAGAGATAATGTTCGTACTGTTGATTCATTTATAATGAATTACGGTAAAGGAACACGCTGTCAGTTCAAAAGG
TTATTTATCGACGAAGGTCTGATGTTGCACACTGGTTGTGTGAATTTTCTTGTTTCTATGTCTCTGTGCGAAATT
GCATATGTTTATGGAGACACACAACAAATTCCATACATCAACAGAGTATCCGGTTTTCCGTACCCTGCACATTT
TGCAAAAATAGAGGTTGATGAGGTGGAAACTCGCAGAACTACGCTGCGTTGTCCAGCCGACATTACCCACTAT
CTTAACAGAAGGTACGAAGGACATGTCATGTGTACATCGTCGGTTAAAAAGTCAGTTTCTCAGGAAATGGTGA
GCGGGGCCGCAATGATCAATCCTGTATCTAAGCCATTGAATGGGAAAGTTTTGACTTTCACTCAGTCTGATAA
AGAGGCGCTGCTTTCTCGAGGATATACGGACGTCCATACAGTACATGAGGTACAAGGTGAGACATATGCAGA
TGTGTCGTTGGTCAGATTGACTCCGACACCTGTATCTATCATCGCAGGAGATAGTCCGCACGTTCTCGTAGCTT
TGTCAAGGCATACCCAAACATTGAAGTATTACACCGTAGTGATGGATCCTCTTGTAAGTATAATTAGGGATTTA

GAAAAACTTAGTTCTTACTTGTTAGATATGTATAAAGTAGATGCAGGGACCCAATAGCAATTACAGGTAGACT
CCGTGTTTAAAGGTTCTAATCTTTTTGTTGCAGCACCAAAGACTGGAGATATCTCAGATATGCAATTTTACTAT
GATAAGTGTCTCCCAGGTAATAGCACCATGTTAAATAACTATGATGCTGTTACCATGAGGTTGACTGACATTTC
TCTTAATGTCAAAGATTGCATATTGGATTTCTCTAAGTCTGTGGCTGCACCGAAGGATCCGATCAAACCACTGA
TTCCAATGGTACGAACGGCGGCAGAAATGCCACGCCAGACTGGACTATTGGAAAATTTGGTGGCGATGATCA
AAAGAAACTTTAATTCACCGGAGTTATCAGGAATAATCGACATTGAGAATACTGCATCTTTAGTAGTAGATAA
ATTTTTTGATAGTTACTTGCTTAAAGAAAAAAGAAAACCAAATAAAAATGTTTCTTTATTTTGTAGAGAGTCTCT
CAATAGATGGTTAGAGAAGCAGGAGCAAGTGACCATTGGTCAGCTTGCAGATTTTGATTTTGTGGATCTTCCT
GCCGTTGATCAGTACAGGCATATGATTAAAGCGCAACCTAAGCAGAAGCTGGATACATCAATTCAAAGCGAAT
ATCCGGCCTTGCAGACGATTGTGTATCATTCGAAAAAGATCAACGCAATCTTCGGTCCTTTGTTCAGTGAGCTC
ACAAGGCAAATGCTCGAAAGCATAGACTCAAGTAAGTTTTTGTTCTTTACAAGGAAGACGCCAGCTCAAATTG
AGGATTTCTTCGGAGATCTCGATAGCCATGTCCCTATGGATATCTTGGAGTTGGATATTTCGAAGTATGACAAA
TCTCAGAACGAGTTCCACTGTGCAGTAGAGTATGAAATATGGAGAAGACTTGGATTAGAAGATTTTCTGGGA
GAAGTTTGGAAACAAGGCCACAGGAAAACTACTCTTAAAGATTACACAGCTGGTATTAAAACGTGTTTATGGT
ACCAGAGAAAGAGTGGGGACGTTACAACATTCATCGGTAATACGGTGATTATTGCTGCTTGTTTAGCTTCCAT
GTTGCCCATGGAGAAAATAATCAAAGGTGCATTTTGCGGAGATGACAGTTTACTATACTTCCCAAAAGGTTGT
GAGTTTCCTGACATACAGCATACAGCCAACCTTATGTGGAATTTCGAGGCTAAGCTATTCAGAAAGCAGTATG
GTTATTTCTGTGGAAGGTACGTGATACATCATGACAGAGGGTGTATTGTTTATTATGACCCTTTGAAGTTGATT
TCTAAACTTGGTGCTAAACACATCAAGGATTGGGATCACTTAGAAGAGTTCAGAAGATCCCTTTGTGATGTTG
CAAATTCGTTGAACAACTGTGCGTATTACACGCAGTTGGACGACGCTGTGAGTGAGGTCCATAAAACCGCACC
CCCGGGTTCGTTTGTATATAAAAGTTTAGTTAAATATCTGTCCGATAAGGTTCTTTTTAGAAGTTTGTTTATAGA
TGGCTCTTGTTAAGGGTAAAGTCAATATTAATGAGTTCATAGACTTGTCAAAATCAGAAAAATTTCTTCCGTCT
ATGTTCACACCTGTTAAGAGTGTCATGATCTCCAAGGTTGATAAGATATTGGTTCATGAAGATGAATCTTTGTC
CGAAGTCAATTTACTCAAAGGTGTAAAACTCATTGATGGTGGCTATGTACATCTTGCTGGTCTTGTGGTGACA
GGTGAATGGAATTTGCCAGATAATTGTCGTGGTGGTGTCAGTGTCTGTTTGGTCGATAAAAGAATGGAGAGA
GCGGACGAGGCAACTCTTGCTTCATACTATACCGCAGCGGCTAAGAAAAGGTTTCAGTTCAAAGTCGTTCCAA
ATTACAACATCACTACCAAGGACGCAAAAAAGGCAGTTTGGCAAGTACTAGTTAATATTAGAAATGTTAAAAT
TGCTGCGGGTTACTGTCCGCTGTCATTAGAATTTGTGTCAGTGTGTATTGTTTATAAAAATATTATAAAACTCG
GTTTGAGAGAGAAAATTACGAGCGTAACGGATGGAGGGCCCATGGAACTATCAGAAGAAGTTGTTGATGAG
TTCATGGAAGAAGTCCCGATGTCTGTAAGGCTTGCAAAATTTCGTTCGAAGACCGGGAAAAAGTTTAGTAGTA
AAAGTGAGAATAATAGTGGTAATAATAGGCCGAAACCAAACAAAAACCAAAGGAAGGAAAAGGGTTTAAAA
GTTAGGGTTGAGAAGGATAATTTAATTGATAATGAATTGGAGACTTACGTCGCCGATTCAGATTCGTATTAAA
TATGTCTTACACAATCGCAACTCCATCGCAATTTGTGTTTTTGTCATCAGCATGGGCCGACCCTATAGAATTAAT
AAATTTATGTACTAATTCACTAGGTAATCAATTCCAAACACAACAAGCTAGAACAACCGTTCAACGGCAATTTA
GCGAAGTGTGGAAACCTGTCCCTCAAGTCACTGTTAGGTTTCCTGACAGTGGTTTTAAGGTGTATAGGTACAA
TGCGGTACTAGATCCTCTAGTTACTGCTTTGTTAGGAGCTTTCGATACTAGAAATAGGATTATAGAAGTCGAAA
ATCAGGCGAACCCGACAACCGCCGAAACGTTAGACGCTACTCGTAGAGTAGATGACGCAACGGTGGCTATAA
GGAGCGCTATAAATAATTTAGTAGTAGAATTGGTCAAAGGAACAGGTTTGTACAATCAGAGCACATTTGAAA
GTGCATCCGGTTTACAATGGTCCTCTGCACCTGCATCTTGA

MN882020

ATGGCATACACACAGACAGCTACCACATCCGCTTTGCTCGACACTGTCCGAGGTAACAATACCTTGGTCAACG
ATCTTGCGAAGCGGCGTCTTTATGACACAGCGGTCGACGAGTTCAACGCTCGTGATCGCAGGCCCAAAGTAA
ATTTTTCCAAAGTAATAAGTGAGGAACAGACGCTTATTGCTACTAGGGCATATCCAGAATTCCAGATAACCTTC
TATAATACGCAGAATGCCGTGCATTCGCTTGCCGGTGGACTACGATCCTTAGAACTGGAATATCTAATGATGC
AGATCCCGTACGGATCACTCACATATGATATAGGTGGGAATTTTGCATCTCATCTGTTCAAAGGACGGGCATA

TGTTCACTGCTGTATGCCCAATCTTGATGTCCGCGACATAATGCGGCACGAAGGCCAGAAAGACAGTATAGAA
TTATACCTTTCCAGGCTTGAGCGGGGCAACAAAGTTGTCCCAAATTTCCAAAAGGAAGCTTTTGACAGATACG
CTGAAACGCCAGACGAAGTTGTCTGTCACAGTACCTTCCAAACGTGTACGCACCAGCAGGTGAAAAACACAG
GCAGGGTGTATGCTATTGCACTGCACAGTATATACGATATACCTGCTGATGAATTCGGAGCGGCACTTTTAAG
GAAAAATGTCCATGTTTGTTACGCCGCCTTCCACTTTTCCGAGAATTTACTTCTCGAAGATTCACACGTCAACCT
TGACGAAATCAATGCGTGTTTTTCGCGTGATGGAGACAAGCTGACTTTTTCTTTCGCATCTGAGAGCACTTTAA
ATTATTGTCATAGTTATTCTAATATTTTAAAATACGTGTGCAAAACTTACTTCCCGGCATCTAATAGAGAGGTCT
ACATGAAGGAGTTTTTGGTCACCAGGGTTAACACCTGGTTTTGTAAGTTTTCTAGGATAGATACTTTTTTATTAT
ACAAGGGGGTAGCCCACAAAGGTGTAAATAGTGAGCAATTTTACAGCGCAATGGAAGATGCATGGCACTACA
AAAAGACTCTTGCAATGTGTAACAGCGAGAGGATTCTTCTTGAAGATTCCTCATCGGTCAATTACTGGTTCCCA
AAAATGAGAGATATGGTCATAGTTCCTCTATTCGACATATCTCTCGACACCAGTAAAAGGACCCGCAAAGAAG
TCTTAGTGTCAAAGGATTTTGTATTCACAGTTTTAAATCACATTCGCACTTATCAAGCCAAGGCACTTACATACT
CCAATGTTTTATCCTTTGTCGAATCAATTCGTTCAAGGGTAATTATCAACGGAGTGACTGCCAGGTCTGAGTGG
GATGTTGACAAATCTCTTTTGCAATCCTTGTCCATGACATTTTTCTTGCATACTAAGCTTGCCGTTTTAAAAGAC
GAATTGTTAATCAGCAAGTTTAGTTTGGGGCCAAAATCAGTAAGCCAGCATGTATGGGATGAGATTTCCCTGG
CTTTTGGAAACGCATTTCCATCGATCAAGGAGAGACTGCTAAATCGGAAACTAATTAAAGTGTCGGGAGACGC
ATTAGAAATCAGGGTGCCTGATTTATATGTGACTTTTCACGATAGATTAGTGACTGAGTACAAAACATCGGTG
GATATGCCAGTGCTTGATATCAGAAAGAGAATGGAGGAGACTGAGGTTATGTACAATGCATTGTCTGAGCTA
TCTGTGCTCAAGGAGTCGGACAAGTTCGACGTTGATGTTTTTCCCGGATGTGCCAGACTTTGGAGGTAGACC
CAATGACTGCAGCAAAGGTTATTGTGGCAGTGATGAGCAACGAGAGCGGACTGACTCTTACATTCGAACAGC
CAACTGAAGCAAATGTCGCATTGGCACTTAAAGATTCAGAAAAAGCCTCTGAGGGTGCACTAGTGGTTACTTC
TAGAGATGTTGAAGAACCATCCATGAAGGGTTCAATGGCAAGAGGAGAGTTACAATTGGCCGGTCTGTCTGG
AGACCAACCAGAGTCTTCCTATACTCGGAACGAGGAAATAGAGTCATTAGAGCAATTCCACATGGCAACGGCT
AGTTCGTTAATTCGGAAACAGATGAGTTCGATTGTGTACACGGGCCCCATTAAAGTTCAGCAAATGAAAAACT
TTATTGATAGCCTGGTAGCATCACTCTCTGCTGCGGTGTCGAACCTAGTCAAGATCCTAAAGGATACAGCTGCT
ATAGATCTCGAAACCCGTCAGAAGTTTGGAGTCTTAGATGTTGCGACCAAAAGATGGTTAATTAAACCTTTAG
CCAAGAATCACGCATGGGGCGTTATTGAAACACATGCTAGGAAGTACCACGTTGCACTTTTGGAGTATGATGA
GCATGGAGTGGTAACTTGCGACAGTTGGAGAAGGGTGGCCGTGAGTTCTGAGTCAATGGTTTATTCTGATAT
GGCAAAGCTTAGAACACTGAGGAGATTATTAAGAGATGGTGAGCCTCATGTCAGCAGTGCTAAAGTCGTCCT
AGTTGACGGTGTCCCGGGTTGTGGAAAGACAAAAGAGATTCTCTCGAAAGTAAATTTTGAGGAAGATCTAAT
CTTAGTACCGGGTAAGCAGGCTGCTGAAATGATAAAGAGGCGTGCTAATGCGTCAGGAATAATTCAAGCCAC
AAGAGATAATGTTCGTACTGTTGATTCATTTATAATGAATTACGGTAAAGGAACACGCTGTCAGTTCAAAAGG
TTATTTATCGACGAAGGTCTGATGTTGCACACTGGTTGTGTGAATTTTCTTGTTTCTATGTCTCTGTGCGAAATT
GCATATGTTTATGGAGACACACAACAAATTCCATACATCAACAGAGTATCCGGTTTTCCGTACCCTGCACATTT
TGCAAAAATAGAGGTTGATGAGGTGGAAACTCGCAGAACTACGCTGCGTTGTCCAGCCGACATTACCCACTAT
CTTAACAGAAGGTACGAAGGACATGTCATGTGTACATCGTCGGTTAAAAAGTCGGTTTCTCAGGAAATGGTG
AGCGGGGCCGCAATGATCAATCCTGTATCTAAGCCATTGAATGGGAAAGTTTTGACTTTCACTCAGTCTGATA
AAGAGGCGCTGCTTTCTCGAGGATATACGGACGTCCATACAGTACATGAGGTACAAGGTGAGACATATGCAG
ATGTGTCGTTGGTCAGATTGACTCCGACACCTGTATCTATCATCGCAGGAGATAGTCCGCACGTTCTCGTAGCT
TTGTCAAGGCATACCCAAACATTGAAGTATTACACCGTAGTGATGGATCCTCTTGTAAGTATAATTAGGGATTT
AGAAAAACTTAGTTCTTACTTGTTAGATATGTATAAAGTAGATGCAGGGACCCAATAGCAATTACAGGTAGAC
TCCGTGTTCAAAGGTTCTAATCTTTTTGTTGCAGCACCAAAGACTGGAGATATCTCAGATATGCAATTTTACTAT
GATAAGTGTCTCCCAGGTAATAGCACCATGTTAAATAACTATGATGCTGTTACCATGAGGTTGACTGACATTTC
TCTTAATGTCAAAGATTGCATATTGGATTTCTCTAAGTCTGTGGCTGCACCGAAGGATCCGATCAAACCACTGA
TTCCAATGGTACGAACGGCGGCAGAAAAGCCACGCCAGACTGGACTATTGGAAAATTTGGTGGCGATGATCA
AAAGAAACTTTAATTCACCGGAGTTATCAGGAATAATCGACATTGAGAATACTGCATCTTTAGTAGTAGATAA
ATTTTTTGATAGTTACTTGCTTAAAGAAAAAAGAAAACCAAATAAAAATGTTTCTTTATTTTGTAGAGAGTCTCT
CAATAGATGGTTAGAGAAGCAGGAGCAAGTGACCATTGGTCAGCTTGCAGATTTTGATTTTGTGGATCTTCCT

GCCGTTGATCAGTACAGGCATATGATTAAAGCGCAACCTAAGCAGAAGCTGGATACATCAATTCAAAGCGAAT
ATCCGGCCTTGCAGACGATTGTGTATCATTCGAAAAAGATCAACGCAATCTTCGGTCCTTTGTTCAGTGAGCTC
ACAAGGCAAATGCTCGAAAGCATAGACTCAAGTAAGTTTTTGTTCTTTACAAGGAAGGCGCCAGCTCAAATTG
AGGATTTCTTCGGAGATCTCGATAGCCATGTCCCTATGGATATCTTGGAGTTGGATATTTCGAAGTATGACAAA
TCTCAGAACGAGTTCCACTGTGCAGTAGAGTATGAAATATGGAGAAGACTTGGATTAGAAGATTTTCTGGGA
GAAGTTTGGAAACAAGGCCACAGAAAAACTACTCTTAAAGATTACACAGCTGGTATTAAAACGTGTTTATGGT
ACCAGAGAAAGAGTGGGGACGTTACAACATTCATCGGTAATACGGTGATTATTGCTGCTTGTTTAGCATCCAT
GTTGCCCATGGAGAAAATAATCAAAGGTGCATTTTGCGGAGATGACAGTTTACTATACTTCCCAAAAGGTTGT
GAGTTTCCTGACATACAGCATACAGCCAACCTTATGTGGAATTTCGAGGCTAAGCTATTCAGAAAGCAGTATG
GTTATTTCTGTGGAAGGTACGTGATACATCACGACAGAGGGTGTATTGTTTATTATGACCCTTTGAAGTTGATT
TCTAAACTTGGTGCTAAACACATCAAGGATTGGGATCACTTAGAAGAGTTCAGAAGATCCCTTTGTGATGTTG
CAAATTCGTTGAACAACTGTGCGTATTACACGCAGTTGGACGACGCTGTGAGTGAGGTCCATAAAACCGCACC
CCCGGGTTCGTTTGTATATAAAAGTTTAGTTAAATATCTGTCCGATAAGGTTCTTTTTAGAAGTTTGTTTATAGA
TGGCTCTTGTTAAGGGTAAAGTCAATATTAATGAGTTCATAGACTTGTCAAAATCAGAAAAATTTCTTCCGTCT
ATGTTCACACCTGTTAAGAGTGTCATGATCTCCAAGGTTGATAAGATATTGGTTCATGAAGATGAATCTTTGTC
CGAAGTCAATTTACTCAAAGGTGTAAAACTCATTGATGGTGGCTATGTACATCTTGCTGGTCTTGTGGTGACA
GGTGAATGGAATTTGCCAGATAATTGTCGTGGTGGTGTCAGTGTCTGTTTGGTCGATAAGAGAATGGAGAGA
GCGGACGAGGCAACTCTTGCTTCATACTATACCGCAGCGGCTAAGAAAAGGTTTCAGTTCAAAGTCGTTCCAA
ATTACAACATCACTACCAAGGACGCAGAAAAGGCAGTTTGGCAAGTACTAGTTAATATTAGAAATGTTAAAAT
TGCTGCGGGTTACTGTCCGCTGTCATTAGAATTTGTGTCAGTGTGTATTGTTTATAAAAATATTATAAAACTCG
GTTTGAGAGAGAAAATTACGAGCGTCACGGATGGAGGGCCTATGGAACTATCAGAAGAAGTTGTTGATGAGT
TCATGGAAGAAGTCCCGATGTCTGTAAGGCTTGCAAAATTTCGTTCGAAGACCGGAAAAAAGTTTAGTAGTAA
AAGTGAGAATAATAGTGGTAATAATAGGCCGAAACCAAACAAAAACCAAAGGAAGGAAAAGGGTTTAAAAG
TTAGGGTTGAGAAGGATAATTTAATTGATAATGAATTGGAGACTTACGTCGCCGATTCAGATTCGTATTAAAT
ATGTCTTACACAATCGCAACTCCATCGCAATTTGTGTTTTTGTCATCAGCATGGGCCGACCCTATAGAATTAATA
AATTTATGTACTAATTCACTAGGTAATCAGTTCCAAACACAACAAGCTAGAACAACCGTTCAACGGCAATTTAG
CGAAGTGTGGAAACCTGTCCCTCAAGTCACTGTTAGGTTTCCTGACAGTGGTTTTAAGGTGTATAGGTACAAT
GCGGTACTAGATCCTCTAGTTACTGCTTTGTTAGGAGCTTTCGATACTAGAAATAGGATTATAGAAGTCGAAA
ATCAGGCGAACCCGACAACCGCCGAAACGTTAGACGCTACTCGTAGAGTAGATGACGCAACGGTGGCTATAA
GGAGCGCTATAAATAATTTAGTAGTAGAATTGGTCAAAGGAACAGGTTTGTACAATCAGAGCACATTTGAAA
GTGCATCCGGTTTACAATGGTCCTCTGCACCTGCATCTTGA

MN882028

ATGGCATACACACAGACAGCTACCACATCCGCTTTGCTCGACACTGTCCGAGGTAACAATACCTTGGTCAACG
ATCTTGCGAAGCGGCGTCTTTATGACACAGCGGTCGACGAGTTCAACGCTCGTGATCGCAGGCCCAAAGTAA
ATTTTTCCAAAGTAATAAGTGAGGAACAGACGCTTATTGCTACTAGGGCATATCCAGAATTCCAGATAACCTTC
TATAATACGCAGAACGCCGTGCATTCGCTTGCCGGTGGACTACGATCCTTAGAACTGGAATATCTAATGATGC
AGATCCCGTACGGATCACTCACATATGATATAGGTGGGAATTTTGCATCTCATCTGTTCAAAGGACGAGCATA
TGTTCACTGCTGTATGCCCAATCTTGATGTCCGCGACATAATGCGGCACGAAGGCCAGAAAGACAGTATAGAA
TTATACCTTTCCAGGCTTGAGCGGGGCAACAAAGTTGTCCCAAATTTCCAAAAGGAAGCTTTTGACAGATACG
CTGAAACGCCAGACGAAGTTGTCTGTCACAGTACCTTCCAAACGTGTACGCACCAGCAGGTGGAAAACACAG
GCAGGGTGTATGCTATTGCACTGCACAGTATATACGATATACCTGCTGATGAATTCGGAGCGGCACTTTTAAG
GAAAAATGTCCATGTTTGTTACGCCGCCTTCCACTTTTCCGAGAATTTACTTCTCGAAGATTCACACGTCAACCT
TGACGAAATCAATGCGTGTTTTTCGCGTGATGGAGACAAGCTGACTTTTTCTTTCGCATCTGAGAGCACTTTAA
ATTATTGTCATAGTTATTCTAATATTTTAAAATACGTGTGCAAAACTTACTTCCCGGCATCTAATAGAGAGGTCT
ACATGAAGGAGTTTTTGGTCACCAGGGTTAACACCTGGTTTTGTAAGTTTTCTAGGATAGATACTTTTTTATTAT

ACAAGGGGGTAGCCCACAAAGGTGTAAATAGTGAGCAATTTTACAGCGCAATGGAAGATGCATGGCACTACA
AAAAGACTCTTGCAATGTGTAACAGCGAGAGGATTCTTCTTGAAGATTCCTCATCGGTCAATTACTGGTTCCCA
AAAATGAGAGATATGGTCATAGTTCCTCTATTCGACATATCTCTCGACACCAGTAAAAGGACCCGCAAAGAAG
TCTTAGTGTCAAAGGATTTTGTATTCACAGTTTTAAATCACATTCGCACTTATCAAGCCAAGGCACTTACATACT
CCAATGTTTTATCCTTTGTCGAATCAATTCGTTCAAGGGTAATTATCAACGGAGTGACTGCCAGGTCTGAGTGG
GATGTTGACAAATCTCTTTTGCAATCCTTGTCCATGACATTTTTCTTGCATACTAAGCTTGCCGTTTTAAAAGAC
GAATTGTTAATCAGCAAGTTTAGTTTGGGGCCAAAATCAGTAAGCCAGCATGTATGGGATGAGATTTCCCTGG
CTTTTGGAAACGCATTTCCATCGATCAAGGAGAGACTGCTAAATCGGAAACTAATTAAAGTGTCGGGAGACGC
ATTAGAAATCAGGGTGCCTGATTTATATGTGACTTTTCACGATAGATTAGTGACTGAGTACAAAACATCGGTG
GATATGCCAGTGCTTGATATCAGAAAGAGAATGGAGGAGACTGAGGTTATGTACAATGCATTGTCTGAGCTA
TCTGTGCTCAAGGAGTCGGACAAGTTCGACGTTGATGTTTTTTCCCGGATGTGCCAGACTTTGGAGGTAGACC
CAATGACTGCAGCAAAGGTTATTGTGGCAGTGATGAGCAACGAGAGCGGACTGACTCTTACATTCGAACAGC
CAACTGAAGCAAATGTCGCATTGGCACTTAAAGATTCAGAAAAAGCCTCTGAGGGTGCACTAGTGGTTACTTC
TAGAGATGTTGAAGAACCATCCATGAAGGGTTCAATGGCAAGAGGAGAGTTACAATTGGCCGGTCTGTCTGG
AGACCAACCAGAGTCTTCCTATACTCGGAACGAGGAAATAGAGTCATTAGAGCAATTCCACATGGCAACGGCT
AGTTCGTTAATTCGGAAACAGATGAGTTCGATTGTGTACACGGGCCCCATTAAAGTTCAGCAAATGAAAAACT
TTATTGATAGCCTGGTAGCATCACTCTCTGCTGCGGTGTCGAACCTAGTCAAGATCCTAAAGGATACAGCTGCT
ATAGATCTCGAAACCCGTCAGAAGTTTGGAGTCTTAGATGTTGCGACCAAAAGATGGTTAATTAAACCTTTAG
CCAAGAATCACGCATGGGGCGTTATTGAAACACATGCTAGGAAGTACCACGTTGCACTTTTGGAGTATGATGA
GCATGGAGTGGTAACTTGCGACAGTTGGAGAAGGGTGGCCGTGAGTTCTGAGTCAATGGTTTATTCTGATAT
GGCAAAGCTCAGAACACTGAGGAGATTATTAAGAGATGGTGAGCCTCATGTCAGCAGTGCTAAAGTCGTCCT
AGTTGACGGTGTCCCGGGTTGTGGAAAGACAAAAGAGATTCTCTCGAAAGTAAATTTTGAGGAAGATCTAAT
CTTAGTACCGGGTAAGCAGGCTGCTGAAATGATAAAGAGGCGTGCTAATGCGTCAGGAATAATTCAAGCCAC
AAGAGATAATGTTCGTACTGTTGATTCATTTATAATGAATTACGGTAAAGGAACACGCTGTCAGTTCAAAAGG
TTATTTATCGACGAAGGTCTGATGTTGCACACTGGTTGTGTGAATTTTCTTGTTTCTATGTCTCTGTGCGAAATT
GCATATGTTTATGGAGACACACAACAAATTCCATACATCAACAGAGTATCCGGTTTTCCGTACCCTGCACATTT
TGCAAAAATAGAGGTTGATGAGGTGGAAACTCGCAGAACTACGCTGCGTTGTCCAGCCGACATTACCCACTAT
CTTAACAGAAGGTACGAAGGACATGTCATGTGTACATCGTCGGTTAAAAAGTCAGTTTCTCAGGAAATGGTGA
GCGGGGCCGCAATGATCAATCCTGTATCTAAGCCATTGAATGGGAAAGTTTTGACTTTCACTCAGTCTGATAA
AGAGGCGCTGCTTTCTCGAGGATATACGGACGTCCATACAGTACATGAGGTACAAGGTGAGACATATGCAGA
TGTGTCGTTGGTCAGATTGACTCCGACACCTGTATCTATCATCGCAGGAGATAGTCCGCACGTTCTCGTAGCTT
TGTCAAGGCATACCCAAACATTGAAGTATTACACCGTAGTGATGGATCCTCTTGTAAGTATAATTAGGGATTTA
GAAAAACTTAGTTCTTACTTGTTAGATATGTATAAAGTAGATGCAGGGACCCAATAGCAATTACAGGTAGACT
CCGTGTTTAAAGGTTCTAATCTTTTTGTTGCAGCACCAAAGACTGGAGATATCTCAGATATGCAATTTTACTAT
GATAAGTGTCTCCCAGGTAATAGCACCATGTTAAATAACTATGATGCTGTTACCATGAGGTTGACTGACATTTC
TCTTAATGTCAAAGATTGCATATTGGATTTCTCTAAGTCTGTGGCTGCACCGAAGGATCCGATCAAACCACTGA
TTCCAATGGTACGAACGGCGGCAGAAAAGCCACGCCAGACTGGACTATTGGAAAATTTGGTGGCGATGATCA
AAAGAAACTTTAATTCACCGGAGTTATCAGGAATAATCGACATTGAGAATACTGCATCTTTAGTAGTAGATAA
ATTTTTTGATAGTTACTTGCTTAAAGAAAAAAGAAAACCAAATAAAAATGTTTCTTTATTTTGTAGAGAGTCTCT
CAATAGATGGTTAGAGAAGCAGGAGCAAGTGACCATTGGTCAGCTTGCAGATTTTGATTTTGTGGATCTTCCT
GCCGTTGATCAGTACAGGCATATGATCAAAGCGCAACCTAAGCAGAAGCTGGATACATCAATTCAAAGCGAA
TATCCGGCCTTGCAGACGATTGTGTATCATTCGAAAAAGATCAACGCAATCTTCGGTCCTTTGTTCAGTGAGCT
CACAAGGCAAATGCTCGAAAGCATAGACTCAAGTAAGTTTTTGTTCTTTACAAGGAAGGCGCCAGCTCAAATT
GAGGATTTCTTCGGAGATCTCGATAGCCATGTCCCTATGGATATCTTGGAGTTGGATATTTCGAAGTATGACA
AATCTCAGAACGAGTTCCACTGTGCAGTAGAGTATGAAATATGGAGAAGACTTGGATTAGAAGATTTTCTGG
GAGAAGTTTGGAAACAAGGCCACAGAAAAACTACTCTTAAAGATTACACAGCTGGTATTAAAACGTGTTTATG
GTACCAGAGAAAGAGTGGGGACGTTACAACATTCATCGGTAATACGGTGATTATTGCTGCTTGTTTAGCATCC
ATGTTGCCCATGGAGAAAATAATCAAAGGTGCATTTTGCGGAGATGACAGTTTACTATACTTCCCAAAAGGTT

GTGAGTTTCCTGACATACAGCATACAGCCAACCTTATGTGGAATTTCGAGGCTAAGCTATTCAGAAAGCAGTA
TGGTTATTTCTGTGGAAGGTACGTGATACATCACGACAGAGGGTGTATTGTTTATTATGACCCTTTGAAGTTGA
TTTCTAAACTTGGTGCTAAACACATCAAGGATTGGGATCACTTAGAAGAGTTCAGAAGATCCCTTTGTGATGTT
GCAAATTCGTTGAACAACTGTGCGTATTACACGCAGTTGGACGACGCTGTGAGTGAGGTCCATAAAACCGCAC
CCCCGGGTTCGTTTGTATATAAAAGTTTAGTTAAATATCTGTCCGATAAGGTTCTTTTTAGAAGTTTGTTTATAG
ATGGCTCTTGTTAAGGGTAAAGTCAATATTAATGAGTTCATAGACTTGTCAAAATCAGAAAAATTTCTTCCGTC
TATGTTCACACCTGTTAAGAGTGTCATGATCTCCAAGGTTGATAAGATATTGGTTCATGAAGATGAATCTTTGT
CCGAAGTCAATTTACTCAAAGGTGTAAAACTCATTGATGGTGGCTATGTACATCTTGCTGGTCTTGTGGTGACA
GGTGAATGGAATTTGCCAGATAATTGTCGTGGTGGTGTCAGTGTCTGTTTGGTCGATAAGAGAATGGAGAGA
GCGGACGAGGCAACTCTTGCTTCATACTATACCGCAGCGGCTAAGAAAAGGTTTCAGTTCAAAGTCGTTCCAA
ATTACAACATCACTACCAAGGACGCAGAAAAGGCAGTTTGGCAAGTACTAGTTAATATTAGAAATGTTAAAAT
TGCTGCGGGTTACTGTCCGCTGTCATTAGAATTTGTGTCAGTGTGTATTGTTTATAAAAATATTATAAAACTCG
GTTTGAGAGAGAAAATTACGAGCGTCACGGATGGAGGGCCTATGGAACTATCAGAAGAAGTTGTTGATGAGT
TCATGGAAGAAGTCCCGATGTCTGTAAGGCTTGCAAAATTTCGTTCGAAGACCGGAAAAAAGTTTAGTAGTAA
AAGTGAGAATAATAGTGGTAATAATAGGCCGAAACCAAACAAAAACCAAAGGAAGGAAAAGGGTTTAAAAG
TTAGGGTTGAGAAGGATAATTTAATTGATAATGAATTGGAGACTTACGTCGCCGATTCAGATTCGTATTAAAT
ATGTCTTACACAATCGCAACTCCATCGCAATTTGTGTTTTTGTCATCAGCATGGGCCGACCCTATAGAATTAATA
AATTTATGTACTAATTCACTAGGTAATCAGTTCCAAACACAACAAGCTAGAACAACCGTTCAACGGCAATTTAG
CGAAGTGTGGAAACCTGTCCCTCAAGTCACTGTTAGGTTTCCTGACAGTGGTTTTAAGGTGTATAGGTACAAT
GCGGTACTAGATCCTCTAGTTACTGCTTTGTTAGGAGCTTTCGATACTAGAAATAGGATTATAGAAGTCGAAA
ATCAGGCGAACCCGACAACCGCCGAAACGTTAGACGCTACTCGTAGAGTAGATGACGCAACGGTGGCTATAA
GGAGCGCTATAAATAATTTAGTAGTAGAATTGGTCAAAGGAACAGGTTTGTACAATCAGAGCACATTTGAAA
GTGCATCCGGTTTACAATGGTCCTCTGCACCTGCATCTTGA

MN882030

ATGGCATACACACAGACAGCTACCACATCCGCTTTGCTCGACACTGTCCGAGGTAACAATACCTTGGTCAACG
ATCTTGCGAAGCGGCGTCTTTATGACACAGCGGTCGACGAGTTCAACGCTCGTGATCGCAGGCCCAAAGTAA
ATTTTTCCAAAGTAATAAGTGAGGAACAGACGCTTATTGCTACTAGGGCATATCCAGAATTCCAGATAACCTTC
TATAATACGCAGAACGCCGTGCATTCGCTTGCCGGTGGACTACGATCCTTAGAACTGGAATATCTAATGATGC
AGATCCCGTACGGATCACTCACATATGATATAGGTGGGAATTTTGCATCTCATCTGTTCAAAGGACGGGCATA
TGTTCACTGCTGTATGCCCAATCTTGATGTCCGCGACATAATGCGGCACGAAGGCCAGAAAGACAGTATAGAA
TTATACCTTTCCAGGCTTGAGCGGGGCAACAAAGTTGTCCCAAATTTCCAAAAGGAAGCTTTTGACAGATACG
CTGAAACGCCAGACGAAGTTGTCTGTCACAGTACCTTCCAAACGTGTACGCACCAGCAGGTGGAAAACACAG
GCAGGGTGTATGCTATTGCATTGCACAGTATATACGATATACCTGCTGATGAATTCGGAGCGGCACTTTTAAG
GAAAAATGTCCATGTTTGTTACGCCGCCTTCCACTTTTCCGAGAATTTACTTCTCGAAGATTCACACGTCAACCT
TGACGAAATCAACGCGTGTTTTTCGCGCGATGGAGACAAGCTGACTTTTTCTTTCGCATCTGAGAGCACTTTAA
ATTACTGTCATAGTTATTCTAATATTTTAAAATACGTGTGCAAAACTTACTTCCCGGCATCTAATAGAGAGGTCT
ACATGAAGGAGTTTTTGGTCACCAGGGTTAACACCTGGTTTTGTAAGTTTTCTAGGATAGATACTTTTTTATTAT
ACAAGGGGGTAGCCCACAAAGGTGTAAATAGTGAGCAATTTTACAGCGCAATGGAAGATGCATGGCACTACA
AAAAGACTCTTGCAATGTGTAACAGCGAGAGGATTCTTCTTGAAGATTCCTCATCGGTCAATTACTGGTTCCCA
AAAATGAGAGATATGGTCATAGTTCCTCTATTCGACATATCTCTCGACACCAGTAAAAGGACCCGCAAAGAAG
TCTTAGTGTCAAAGGATTTTGTATTCACAGTTTTAAATCACATTCGCACTTATCAAGCCAAGGCACTTACATACT
CCAATGTTTTATCCTTTGTCGAATCAATTCGTTCAAGGGTAATTATCAACGGAGTGACTGCCAGGTCTGAGTGG
GATGTTGACAAATCTCTTTTGCAATCCTTGTCCATGACATTTTTCTTGCATACTAAGCTTGCCGTTTTAAAAGAC
GAATTGTTAATCAGCAAGTTTAGTTTGGGGCCAAAATCAGTAAGCCAGCATGTATGGGATGAGATTTCCCTGG
CTTTTGGAAACGCATTTCCATCGATCAAGGAGAGACTGCTAAATCGGAAACTAATTAAAGTGTCGGGAGACGC

ATTAGAAATCAGGGTGCCTGATTTATATGTGACTTTTCACGATAGATTAGTGACTGAGTACAAAACATCGGTG
GATATGCCAGTGCTTGATATCAGAAAGAGAATGGAGGAGACTGAGGTTATGTACAATGCATTGTCTGAGCTA
TCTGTGCTCAAGGAGTCGGACAAGTTCGACGTTGATGTTTTTTCCCGGATGTGCCAGACTTTGGAGGTAGACC
CAATGACTGCAGCAAAGGTTATTGTGGCAGTGATGAGCAATGAGAGCGGACTGACTCTTACATTCGAACAGC
CAACTGAAGCAAATGTCGCATTGGCACTTAAAGATTCAGAAAAAGCCTCTGAGGGTGCACTAGTGGTTACTTC
TAGAGATGTTGAAGAACCATCCATGAAGGGTTCAATGGCAAGAGGAGAGTTACAATTGGCCGGTCTGTCTGG
AGACCAACCAGAGTCTTCCTATACTCGGAACGAGGAAATAGTGTCATTAGAGCAATTCCACATGGCAACGGCT
AGTTCGTTAATTCGGAAACAGATGAGTTCGATTGTGTACACGGGCCCCATTAAAGTTCAGCAAATGAAAAACT
TTATTGATAGCCTGGTAGCATCACTCTCTGCTGCGGTGTCGAACCTAGTCAAGATCCTAAAGGATACAGCTGCT
ATAGATCTCGAAACCCGTCAGAAGTTTGGAGTCTTAGATGTTGCGACCAAAAGATGGTTAATTAAACCTTTAG
CCAAGAATCACGCATGGGGCGTTATTGAAACACATGCTAGGAAGTACCACGTTGCACTTTTGGAGTATGATGA
GCATGGAGTGGTAACTTGCGACAGTTGGAGAAGGGTGGCCGTGAGTTCTGAGTCAATGGTTTATTCTGATAT
GGCAAAGCTCAGAACACTGAGGGGATTATTAAGAGATGGTGAGCCTCATGTCAGCAGTGCTAAAGTCGTCCT
AGTTGACGGTGTCCCGGGTTGTGGAAAGACAAAAGAGATTCTCTCGAAAGTAAATTTTGAGGAAGATCTAAT
CTTAGTACCGGGTAAGCAGGCTGCTGAAATGATAAAGAGGCGTGCTAATGCGTCAGGAATAATTCAAGCCAC
AAGAGATAATGTTCGTACTGTTGATTCATTTATAATGAATTACGGTAAAGGAACACGCTGTCAGTTCAAAAGG
TTATTTATCGACGAAGGTCTGATGTTGCACACTGGTTGTGTGAATTTTCTTGTTTCTATGTCTCTGTGCGAAATT
GCATATGTTTATGGAGACACACAACAAATTCCATACATCAACAGAGTATCCGGTTTTCCGTACCCTGCACATTT
TGCAAAAATAGAGGTTGATGAGGTGGAAACTCGCAGAACTACGCTGCGTTGTCCAGCCGACATTACTCACTAT
CTTAACAGAAGGTACGAAGGACATGTCATGTGTACATCGTCGGTTAAAAAGTCAGTTTCTCAGGAAATGGTGA
GCGGGGCCGCAATGATCAATCCTGTATCTAAGCCATTGAATGGGAAAGTTTTGACTTTCACTCAGTCTGATAA
AGAGGCGCTGCTTTCTCGAGGATATACGGACGTCCATACAGTACATGAGGTACAAGGTGAGACATATGCAGA
CGTGTCGTTGGTCAGATTGACTCCGACACCTGTATCTATCATCGCAGGAGATAGTCCGCACGTTCTCGTAGCTT
TGTCAAGGCATACCCAAACATTGAAGTATTACACCGTAGTGATGGATCCTCTTGTAAGTATAATTAGGGATTTA
GAAAAACTTAGTTCTTACTTGTTAGATATGTATAAAGTAGATGCAGGGACCCAATAGCAATTACAGGTAGACT
CCGTGTTTAAAGGTTCTAATCTTTTTGTTGCAGCACCAAAGACTGGAGATATCTCAGATATGCAATTTTACTAT
GATAAGTGTCTCCCAGGTAATAGCACCATGTTAAATAACTATGATGCTGTTACCATGAGGTTGACTGACATTTC
TCTTAATGTCAAAGATTGCATATTGGATTTCTCTAAGTCTGTGGCTGCACCGAAGGATCCGATCAAACCACTGA
TTCCAATGGTACGAACGGCGGCAGAAATGCCACGCCAGACTGGACTATTGGAAAATTTGGTGGCGATGATCA
AAAGAAACTTCAATTCACCGGAGTTATCAGGAATAATCGACATTGAGAATACTGCATCTTTAGTAGTAGATAA
ATTTTTTGATAGTTACTTGCTTAAAGAAAAAAGAAAACCAAATAAAAATGTTTCTTTATTTTGTAGAGAGTCTCT
CAATAGATGGTTAGAGAAGCAGGAGCAAGTGACCATTGGTCAGCTTGCAGATTTTGATTTTGTGGATCTTCCT
GCCGTTGATCAGTACAGGCATATGATTAAAGCGCAACCTAAGCAGAAGCTGGATACATCAATTCAAAGCGAAT
ATCCGGCCTTGCAGACGATTGTGTATCATTCGAAAAAGATCAACGCAATCTTCGGTCCTTTGTTCAGTGAGCTC
ACAAGGCAAATGCTCGAAAGCATAGACTCAAGTAAGTTTTTGTTCTTTACAAGGAAGACGCCAGCTCAAATTG
AGGATTTCTTCGGAGATCTCGATAGCCATGTCCCTATGGATATCTTGGAGTTGGATATTTCGAAGTATGACAAA
TCTCAGAACGAGTTCCACTGTGCAGTAGAGTATGAAATATGGAGAAGACTTGGATTAGAAAATTTTCTGGGA
GAAGTTTGGAAACAAGGCCACAGGAAAACTACTCTTAAAGATTACACAGCTGGTATTAAAACGTGTTTATGGT
ACCAGAGAAAGAGTGGGGACGTTACAACATTCATCGGTAATACGGTGATTATTGCTGCTTGTTTAGCTTCCAT
GTTGCCCATGGAGAAAATAATCAAAGGTGCATTTTGCGGAGATGACAGTTTACTATACTTCCCAAAAGGTTGT
GAGTTTCCTGACATACAGCATACAGCCAACCTTATGTGGAATTTCGAGGCTAAGCTATTCAGAAAGCAGTATG
GTTATTTCTGTGGAAGGTACGTGATACATCATGACAGAGGGTGTATTGTTTATTATGACCCTTTGAAGTTGATT
TCTAAACTTGGTGCTAAACACATCAAGGATTGGGATCACTTAGAAGAGTTCAGAAGATCCCTTTGTGATGTTG
CAAATTCGTTGAACAACTGTGCGTATTACACGCAGTTGGACGACGCTGTGAGTGAGGTCCATAAAACCGCACC
CCCGGGTTCGTTTGTATATAAAAGTTTAGTTAAATATCTGTCCGATAAGGTTCTTTTTAGAAGTTTGTTTATAGA
TGGCTCTTGTTAAGGGTAAAGTCAATATTAATGAGTTCATAGACTTGTCAAAATCAGAAAAAATTTCTTCCGTCT
ATGTTCACACCTGTTAAGAGTGTCATGATCTCCAAGGTTGATAAGATATTGGTTCATGAAGATGAATCTTTGTC
CGAAGTCAATTTACTCAAAGGTGTAAAACTCATTGATGGTGGCTATGTACATCTTGCTGGTCTTGTGGTGACA

GGTGAATGGAATTTGCCAGATAATTGTCGTGGTGGTGTCAGTGTCTGTTTGGTCGATAAGAGAATGGAGAGA
GCGGACGAGGCAACTCTTGCTTCATACTATACCGCAGCGGCTAAGAAAAGGTTTCAGTTCAAAGTCGTTCCAA
ATTACAACATCACTACCAAGGACGCAGAAAAGGCAGTTTGGCAAGTACTAGTTAATATTAGAAATGTTAAAAT
TGCTGCGGGTTACTGTCCGCTGTCATTAGAATTTGTGTCAGTGTGTATTGTTTATAAAAATATTATAAAACTCG
GTTTGAGAGAGAAAATTACGAGCGTCACGGATGGAGGGCCCATGGAACTATCAGAAGAAGTTGTTGATGAG
TTCATGGAAGAAGTCCCGATGTCTGTAAGGCTTGCAAAATTTCGTTCGAAGACCGGAAAAAAGTTTAGTAGTA
AAAGTGAGAATAATAGTGGTAATAATAGGCCGAAACCAAACAAAAACCAAAGGAAGGAAAAGGGTTTAAAA
GTTAGGGTTGAGAAGGATAATTTAATTGATAATGAATTGGAGACTTACGTCGCCGATTCAGATTCGTATTAAA
TATGTCTTACACAATCGCAACTCCATCGCAATTTGTGTTTTTGTCATCAGCATGGGCCGACCCTATAGAATTAAT
AAATTTATGTACTAATTCACTAGGTAATCAGTTCCAAACACAACAAGCTAGAACAACCGTTCAACGGCAATTTA
GCGAAGTGTGGAAACCTGTCCCTCAAGTCACTGTTAGGTTTCCTGACAGTGGTTTTAAGGTGTATAGGTACAA
TGCGGTACTAGATCCTCTAGTTACTGCTTTGTTAGGAGCTTTCGATACTAGAAATAGGATTATAGAAGTCGAAA
ATCAGGCGAACCCGACAACCGCCGAAACGTTAGACGCTACTCGTAGAGTAGATGACGCAACGGTGGCTATAA
GGAGCGCTATAAATAATTTAGTAGTAGAATTGGTCAAAGGAACAGGTTTGTACAATCAGAGCACATTTGAAA
GTGCATCCGGTTTACAATGGTCCTCTGCACCTGCATCTTGA

MN882041

ATGGCATACACACAGACAGCTACCACATCCGCTTTGCTCGACACTGTCCGAGGTAACAATACCTTGGTCAACG
ATCTTGCGAAGCGGCGTCTTTATGACACAGCGGTCGACGAGTTCAACGCTCGTGATCGCAGGCCCAAAGTAA
ATTTTTCCAAAGTAATAAGTGAGGAACAGACGCTTATTGCTACTAGGGCATATCCTGAATTCCAGATAACCTTC
TATAATACGCAGAACGCCGTGCATTCGCTTGCCGGTGGACTACGATCCTTAGAACTGGAATATCTAATGATGC
AGATCCCGTACGGATCACTCACATATGATATAGGTGGGAATTTTGCATCTCATCTGTTCAAAGGACGGGCATA
TGTTCACTGCTGTATGCCCAATCTTGATGTCCGCGACATAATGCGGCACGAAGGCCAGAAAGACAGTATAGAA
TTATACCTTTCCAGGCTTGAGCGGGGCAACAAAGTTGTCCCAAATTTCCAAAAGGAAGCTTTTGACAGATACG
CTGAAACGCCAGACGAAGTTGTCTGTCACAGTACCTTCCAAACGTGTACGCACCAGCAGGTGGAAAACACAG
GCAGGGTGTATGCTATTGCATTGCACAGTATATACGATATACCTGCTGATGAATTCGGAGCGGCACTTTTAAG
GAAAAATGTCCATGTTTGTTACGCCGCCTTCCACTTTTCCGAGAATTTACTTCTCGAAGATTCACACGTCAACCT
TGACGAAATCAACGCGTGTTTTTCGCGTGATGGAGACAAGCTGACTTTTTCTTTCGCATCTGAGAGCACTTTAA
ATTATTGTCATAGTTATTCTAATATTTTAAAATACGTGTGCAAAACTTACTTCCCGGCATCTAATAGAGAGGTCT
ACATGAAGGAGTTTTTGGTCACCAGGGTTAACACCTGGTTTTGTAAGTTTTCTAGGATAGATACTTTTTTATTAT
ACAAGGGGGTAGCCCACAAAGGTGTAAATAGTGAGCAATTTTACAGCGCAATGGAAGATGCATGGCACTACA
AAAAGACTCTTGCAATGTGTAACAGCGAGAGGATTCTTCTTGAAGATTCCTCATCGGTCAATTACTGGTTCCCA
AAAATGAGAGATATGGTCATAGTTCCTCTATTCGACATATCTCTCGACACTAGTAAAAGGACCCGCAAAGAAG
TCTTAGTGTCAAAGGATTTTGTATTCACAGTTTTAAATCACATTCGCACTTATCAAGCCAAGGCACTTACATACT
CCAATGTTTTATCCTTTGTCGAATCAATTCGTTCAAGGGTAATTATCAACGGAGTGACTGCCAGGTCTGAGTGG
GATGTTGACAAATCTCTTTTGCAATCCTTGTCCATGACATTTTTCTTGCATACTAAGCTTGCCGTTTTAAAAGAC
GAATTGTTAATCAGCAAGTTTAGTTTGGGGCCAAAATCAGTAAGCCAGCATGTATGGGATGAGATTTCCCTGG
CTTTTGGAAACGCATTTCCATCGATCAAGGAGAGACTGCTAAATCGGAAACTAATTAAAGTGTCGGGAGACGC
ATTAGAAATCAGGGTGCCTGATTTATATGTGACTTTTCACGATAGATTAGTGACTGAGTACAAAACATCGGTG
GATATGCCAGTGCTTGATATCAGAAAGAGAATGGAGGAGACTGAGGTTATGTACAATGCATTGTCTGAGCTA
TCTGTGCTCAAGGAGTCGGACAAGTTCGACGTTGATGTTTTTTCCCGGATGTGCCAGACTTTGGAGGTAGACC
CAATGACTGCAGCAAAGGTTATTGTGGCAGTGATGAGCAACGAGAGCGGACTGACTCTTACATTCGAACAGC
CAACTGAAGCAAATGTCGCATTGGCACTTAAAGATTCAGAAAAAGCCTCTGAGGGTGCACTAGTGGTTACTTC
TAGAGATGTTGAAGAACCATCCATGAAGGGTTCAATGGCAAGAGGAGAGTTACAATTGGCCGGTCTGTCTGG
AGACCAACCAGAGTCTTCCTATACTCGGAACGAGGAAATAGAGTCATTAGAGCAATTCCACATGGCAACGGCT
GGTTCGTTAATTCGGAAACAGATGAGTTCGATTGTGTACACGGGCCCCATTAAAGTTCAGCAAATGAAAAACT

TTATTGATAGCCTGGTAGCATCACTCTCTGCTGCGGTGTCGAACCTAGTCAAGATCCTAAAGGATACAGCTGCT
ATAGATCTCGAAACCCGTCAGAAGTTTGGAGTCTTAGATGTTGCGACCAAAAGATGGTTAATTAAACCTTTAG
CCAAGAATCACGCATGGGGCGTTATTGAAACACATGCTAGGAAGTACCACGTTGCACTTTTGGAGTATGATGA
GCATGGAGTGGTAACTTGCGACAGTTGGAGAAGGGTGGCCGTGAGTTCTGAGTCAATGGTTTATTCTGATAT
GGCAAAGCTCAGAACACTGAGGAGATTATTAAGAGATGGTGAGCCTCATGTCAGCAGTGCTAAAGTCGTCCT
AGTTGACGGTGTCCCGGGTTGTGGAAAGACAAAAGAGATTCTCTCGAAAGTAAATTTTGAGGAAGATCTAAT
CTTAGTACCGGGTAAGCAGGCTGCTGAAATGATAAAGAGGCGTGCTAATGCGTCAGGAATAATTCAAGCCAC
AAGAGATAATGTTCGTACTGTTGATTCATTTATAATGAATTACGGTAAAGGAACACGCTGTCAGTTCAAAAGG
TTATTTATCGACGAAGGTCTGATGTTGCACACTGGTTGTGTGAATTTTCTTGTTTCTATGTCTCTGTGCGAAATT
GCATATGTTTATGGAGACACACAACAAATTCCATACATCAACAGAGTATCCGGTTTTCCGTACCCTGCACATTT
TGCAAAAATAGAGGTTGATGAGGTGGAAACTCGCAGAACTACGCTGCGTTGTCCAGCCGACATTACCCACTAT
CTTAACAGAAGGTACGAAGGACATGTCATGTGTACATCGTCGGTTAAAAAGTCAGTTTCTCAGGAAATGGTGA
GCGGGGCCGCAATGATCAATCCTGTATCTAAGCCATTGAATGGGAAAGTTTTGACTTTCACTCAGTCTGATAA
AGAGGCGCTGCTTTCTCGAGGATATACGGACGTCCATACAGTACATGAGGTACAAGGTGAGACATATGCAGA
TGTGTCGTTGGTCAGATTGACTCCGACACCTGTATCTATCATCGCAGGAGATAGTCCGCACGTTCTCGTAGCTT
TGTCAAGGCATACCCAAACATTGAAGTATTACACCGTAGTGATGGATCCTCTTGTAAGTATAATTAGGGATTTA
GAAAAACTTAGTTCTTACTTGTTAGATATGTATAAAGTAGATGCAGGGACCCAATAGCAATTACAGGTAGACT
CCGTGTTTAAAGGTTCTAATCTTTTTGTTGCAGCACCAAAGACTGGAGATATCTCAGATATGCAATTTTACTAT
GATAAGTGTCTCCCAGGTAATAGCACCATGTTAAATAACTATGATGCTGTTACCATGAGGTTGACTGACATTTC
TCTTAATGTCAAAGATTGCATATTGGATTTCTCTAAGTCTGTGGCTGCACCGAAGGATCCGATCAAACCACTGA
TTCCAATGGTACGAACGGCGGCAGAAATGCCACGCCAGACTGGACTATTGGAAAATTTGGTGGCGATGATCA
AAAGAAACTTTAATTCACCGGAGTTATCAGGAATAATCGACATTGAGAATACTGCATCTTTAGTAGTAGATAA
ATTTTTTGATAGTTACTTGCTTAAAGAAAAAAGAAAACCAAATAAAAATGTTTCTTTATTTTGTAGAGAGTCTCT
CAATAGATGGTTAGAGAAGCAGGAGCAAGTGACCATTGGTCAGCTTGCAGATTTTGATTTTGTGGATCTTCCT
GCCGTTGATCAGTACAGGCATATGATTAAAGCGCAACCTAAGCAGAAGCTGGATACATCAATTCAAAGCGAAT
ATCCGGCCTTGCAGACGATTGTGTATCATTCGAAAAAGATCAACGCAATCTTCGGTCCTTTGTTCAGTGAGCTC
ACAAGGCAAATGCTCGAAAGCATAGACTCAAGTAAGTTTTTGTTCTTTACAAGGAAGACGCCAGCTCAAATTG
AGGATTTCTTCGGAGATCTCGATAGCCATGTCCCTATGGATATCTTGGAGTTGGATATTTCGAAGTATGACAAA
TCTCAGAACGAGTTCCACTGTGCAGTAGAGTATGAAATATGGAGAAGACTTGGATTAGAAGATTTTCTGGGA
GAAGTTTGGAAACAAGGCCACAGGAAAACTACTCTTAAAGATTACACAGCTGGTATTAAAACGTGTTTATGGT
ACCAGAGAAAGAGTGGGGACGTTACAACATTCATCGGTAATACGGTGATTATTGCTGCTTGTTTAGCTTCCAT
GTTGCCCATGGAGAAAATAATCAAAGGTGCATTTTGCGGAGATGACAGTTTACTATACTTCCCAAAAGGTTGT
GAGTTTCCTGACATACAGCATACAGCCAACCTTATGTGGAATTTCGAGGCTAAGCTATTCAGAAAGCAGTATG
GTTATTTCTGTGGAAGGTACGTGATACATCATGACAGAGGGTGTATTGTTTATTATGACCCTTTGAAGTTGATT
TCTAAACTTGGTGCTAAACACATCAAGGATTGGGATCACTTAGAAGAGTTCAGAAGATCCCTTTGTGATGTTG
CAAATTCGTTGAACAACTGTGCGTATTACACGCAGTTGGACGACGCTGTGAGTGAGGTCCATAAAACCGCACC
CCCGGGTTCGTTTGTATATAAAAGTTTAGTTAAATATCTGTCCGATAAGGTTCTTTTTAGAAGTTTGTTTATAGA
TGGCTCTTGTTAAGGGTAAAGTCAATATTAATGAGTTCATAGACTTGTCAAAATCAGAAAAATTTCTTCCGTCT
ATGTTCACACCTGTTAAGAGTGTCATGATCTCCAAGGTTGATAAGATATTGGTTCATGAAGATGAATCTTTGTC
CGAAGTCAATTTACTCAAAGGTGTAAAACTCATTGATGGTGGCTATGTACATCTTGCTGGTCTTGTGGTGACA
GGTGAATGGAATTTGCCAGATAATTGTCGTGGTGGTGTCAGTGTCTGTTTGGTCGATAAAAGAATGGAGAGA
GCGGACGAGGCAACTCTTGCTTCATACTATACCGCAGCGGCTAAGAAAAGGTTTCAGTTCAAAGTCGTTCCAA
ATTACAACATCACTACCAAGGACGCAGAAAAGGCAGTTTGGCAAGTACTAGTTAATATTAGAAATGTTAAAAT
TGCTGCGGGTTACTGTCCGCTGTCATTAGAATTTGTGTCAGTGTGTATTGTTTATAAAAATATTATAAAACTCG
GTTTGAGAGAGAAAATTACGAGCGTCACGGATGGAGGGCCCATGGAACTATCAGAAGAAGTTGTTGATGAG
TTCATGGAAGAAGTCCCGATGTCTGTAAGGCTTGCAAAATTTCGTTCGAAGACCGGGAAAAAGTTTAGTAGTA
AAAGTGAGAATAATAGTGGTAATAATAGGCCGAAACCAAACAAAAACCAAAGGAAGGAAAAGGGTTTAAAA
GTTAGGGTTGAGAAGGATAATTTAATTGATAATGAATTGGAGACTTACGTCGCCGATTCAGATTCGTATTAAA

TATGTCTTACACAATCGCAACTCCGTCGCAATTTGTGTTTTTGTCATCAGCATGGGCCGACCCTATAGAATTAAT
AAATTTATGTACTAATTCACTAGGTAATCAATTCCAAACACAACAAGCTAGAACAACCGTTCAACGGCAATTTA
GCGAAGTGTGGAAACCTGTCCCTCAAGTCACTGTTAGGTTTCCTGACAGTGGTTTTAAGGTGTATAGGTACAA
TGCGGTACTAGATCCTCTAGTTACTGCTTTGTTAGGAGCTTTCGATACTAGAAATAGGATTATAGAAGTCGAAA
ATCAGGCGAACCCGACAACCGCCGAAACGTTAGACGCTACTCGTAGAGTAGATGACGCAACGGTGGCTATAA
GGAGCGCTATAAATAATTTAGTAGTAGAATTGGTCAAAGGAACAGGTTTGTACAATCAGAGCACATTTGAAA
GTGCATCCGGATTACAATGGTCCTCTGCACCTGCATCTTGA

MN882042

ATGGCATACACACAGACAGCTACCACATCCGCTTTGCTCGACACTGTCCGAGGTAACAATACCTTGGTCAATG
ATCTTGCGAAGCGGCGTCTTTATGACACAGCGGTCGACGAGTTCAACGCTCGTGATCGCAGGCCCAAAGTAA
ATTTTTCCAAAGTAATAAGTGAGGAACAGACGCTTATTGCTACTAGGGCATATCCAGAATTCCAGATAACCTTC
TATAATACGCAGAACGCCGTGCATTCGCTTGCCGGTGGACTACGATCCTTAGAACTGGAATATCTAATGATGC
AGATCCCGTACGGATCACTCACATATGATATAGGTGGGAATTTTGCATCACATCTGTTCAAAGGACGGGCATA
TGTTCACTGCTGTATGCCCAATCTTGATGTCCGCGACATAATGCGGCACGAAGGCCAGAAAGACAGTATAGAA
TTATACCTTTCCAGGCTTGAGCGGGGCAACAAAGTTGTCCCAAATTTCCAAAAGGAAGCTTTTGACAGATACG
CTGAAACGCCAGACGAAGTTGTCTGTCACAGTACCTTCCAAACGTGTACGCACCAGCAGGTGGAAAACACAG
GCAGGGTGTATGCTATTGCATTGCACAGTATATACGATATACCTGCTGATGAATTCGGAGCGGCACTTTTAAG
GAAAAATGTCCATGTTTGTTACGCCGCCTTCCACTTTTCCGAGAATTTACTTCTCGAAGATTCACACGTCAACCT
TGACGAAATCAACGCGTGTTTTTCGCGTGATGGAGACAAGCTGACTTTTTCTTTCGCATCTGAGAGCACTTTAA
ATTATTGTCATAGTTATTCTAATATTTTAAAATACGTGTGCAAAACTTACTTCCCGGCATCTAATAGAGAGGTCT
ACATGAAGGAGTTTTTAGTCACCAGGGTTAACACCTGGTTTTGTAAGTTTTCTAGGATTGATACTTTTTTATTAT
ACAAGGGGGTAGCCCACAAAGGTGTAAATAGTGAGCAATTTTACAGCGCAATGGAAGATGCATGGCACTACA
AAAAGACTCTTGCAATGTGTAACAGCGAGAGGATTCTTCTTGAAGATTCCTCATCGGTCAATTACTGGTTCCCA
AAAATGAGAGATATGGTCATAGTTCCTCTATTCGACATATCTCTCGACACCAGTAAAAGGACCCGCAAAGAAG
TCTTAGTGTCAAAGGATTTTGTATTCACAGTTTTAAATCACATTCGCACTTATCAAGCCAAGGCACTTACATACT
CCAATGTTTTATCCTTTGTCGAATCAATTCGTTCAAGGGTAATTATCAACGGAGTGACTGCTAGGTCTGAGTGG
GATGTTGACAAATCTCTTTTGCAATCCTTGTCCATGACATTTTTCTTGCATACTAAGCTTGCCGTTTTAAAAGAC
GAATTGTTAATCAGCAAGTTTAGTTTGGGGCCAAAATCAGTAAGCCAGCATGTATGGGATGAGATTTCCCTGG
CTTTTGGAAACGCATTTCCATCGATCAAGGAGAGACTGCTAAATCGGAAACTAATTAAAGTGTCGGGAGACGC
ATTAGAAATCAGGGTGCCTGATTTATATGTGACTTTTCACGATAGATTAGTGACTGAGTACAAAACATCGGTG
GATATGCCAGTGCTTGATATCAGAAAGAGAATGGAGGAGACTGAGGTTATGTACAATGCATTGTCTGAGCTA
TCTGTGCTCAAGGAGTCGGACAAGTTCGACGTTGATGTTTTTTCCCGGATGTGCCAGACTTTGGAGGTAGACC
CAATGACTGCAGCAAAGGTTATAGTGGCAGTGATGAGCAACGAGAGCGGACTGACTCTTACATTCGAACAGC
CAACTGAAGCAAATGTCGCATTGGCACTTAAAGATTCAGAAAAAGCCTCTGAGGGTGCACTAGTGGTTACTTC
TAGAGATGTTGAAGAACCATCCATGAAGGGTTCAATGGCAAGAGGAGAGTTACAATTGGCCGGTCTGTCTGG
AGACCAACCAGAGTCTTCCTATACTCGGAACGAGGAAATAGAGTCATTAGAGCAATTCCACATGGCAACGGCT
AGTTCGTTAATTCGGAAACAGATGAGTTCGATTGTGTACACGGGCCCCATTAAAGTTCAGCAAATGAAAAACT
TTATTGATAGCCTGGTAGCATCACTCTCTGCTGCGGTGTCGAACCTAGTCAAGATCCTAAAGGATACAGCTGCT
ATAGATCTCGAAACCCGTCAGAAGTTTGGAGTCTTAGATGTTGCGACCAAAAGATGGTTAATTAAACCTTTAG
CCAAAAATCACGCATGGGGCGTTATTGAAACACATGCTAGGAAGTACCACGTTGCACTTTTGGAGTATGATGA
GCATGGAGTGGTAACTTGCGACAGTTGGAGAAGGGTGGCCGTGAGTTCTGAGTCAATGGTTTATTCTGATAT
GGCAAAGCTCAGAACACTGAGGAGATTATTAAGAGATGGTGAGCCTCATGTCAGCAGTGCTAAAGTCGTCCT
AGTTGACGGTGTCCCGGGTTGTGGAAAGACAAAAGAGATTCTCTCGAAAGTAAATTTTGAGGAAGATCTAAT
CTTAGTACCGGGTAAGCAGGCTGCTGAAATGATAAAGAGGCGTGCTAATGCGTCAGGAATAATTCAAGCCAC
AAGAGATAATGTTCGTACTGTTGATTCATTTATAATGAATTACGGTAAAGGAACACGCTGTCAGTTCAAAAGG

TTATTTATCGACGAAGGTCTGATGTTGCACACTGGTTGTGTGAATTTTCTTGTTTCTATGTCTCTGTGCGAAATT
GCATATGTATATGGAGACACACAACAAATTCCATACATCAACAGAGTATCCGGTTTTCCGTACCCTGCACATTT
TGCAAAAATAGAGGTTGATGAGGTGGAAACTCGCAGAACTACGCTGCGTTGTCCAGCCGACATTACCCACTAT
CTTAACAGAAGGTACGAAGGACATGTCATGTGTACATCGTCGGTTAAAAAGTCAGTTTCTCAGGAAATGGTGA
GCGGGGCCGCAATGATCAATCCTGTATCTAAGCCATTGAATGGGAAAGTTTTGACTTTCACTCAGTCCGATAA
AGAGGCGCTGCTTTCTCGAGGATATACGGACGTCCATACAGTACATGAGGTACAAGGTGAGACATATGCAGA
TGTGTCGTTGGTCAGATTGACTCCGACACCTGTATCTATCATCGCAGGAGATAGTCCGCACGTTCTCGTAGCTT
TGTCAAGGCATACCCAAACATTGAAGTATTACACCGTAGTGATGGATCCTCTTGTAAGTATAATTAGGGATTTA
GAAAAACTTAGTTCTTACTTGTTAGATATGTATAAAGTAGATGCAGGGACCCAATAGCAATTACTGGTAGACT
CCGTGTTTAAAGGTTCTAATCTTTTTGTTGCAGCACCAAAGACTGGAGATATCTCAGATATGCAATTTTACTAT
GATAAGTGTCTCCCAGGTAATAGCACCATGTTAAATAACTATGATGCTGTTACCATGAGGTTGACTGACATTTC
TCTTAATGTCAAAGATTGCATATTGGATTTCTCTAAGTCTGTGGCTGCACCGAAGGATCCGATCAAACCACTGA
TTCCAATGGTACGAACGGCGGCAGAAATGCCACGCCAGACTGGACTATTGGAAAATTTGGTGGCGATGATCA
AAAGAAACTTTAATTCACCGGAGTTATCAGGAATAATCGACATTGAGAATACTGCATCTTTGGTAGTAGATAA
ATTTTTTGATAGTTACTTGCTTAAAGAAAAAAGAAAACCAAATAAAAATGTTTCTTTATTTTGTAGAGAGTCTCT
CAATAGATGGTTAGAGAAGCAGGAGCAAGTGACCATTGGTCAGCTTGCAGATTTTGATTTTGTGGATCTTCCT
GCCGTTGATCAGTACAGGCATATGATTAAAGCGCAACCTAAGCAGAAGCTGGATACATCAATTCAAAGCGAAT
ATCCGGCCTTGCAGACGATTGTGTATCATTCGAAAAAGATCAACGCAATCTTCGGTCCTTTGTTCAGTGAGCTC
ACAAGGCAAATGCTCGAAAGCATAGACTCAAGTAAGTTTTTGTTCTTTACAAGGAAGACGCCAGCTCAAATCG
AGGATTTCTTCGGAGATCTCGATAGCCATGTCCCTATGGATATCTTGGAGTTGGATATTTCGAAGTATGACAAA
TCTCAGAACGAGTTCCACTGTGCAGTAGAGTATGAAATATGGAGAAGACTTGGATTAGAAGATTTTCTGGGA
GAAGTTTGGAAACAAGGCCACAGAAAAACTACTCTTAAAGATTACACAGCTGGTATTAAAACGTGTTTATGGT
ACCAGAGAAAGAGTGGGGACGTTACAACATTCATCGGTAATACGGTGATTATTGCTGCTTGTTTAGCTTCCAT
GTTGCCCATGGAGAAAATAATCAAAGGTGCATTTTGCGGAGATGACAGTTTACTATACTTCCCAAAAGGTTGT
GAGTTTCCCGACATACAGCATACAGCTAACCTTATGTGGAATTTCGAGGCTAAGCTATTCAGAAAGCAGTATG
GTTATTTCTGTGGAAGGTACGTGATACATCATGACAGAGGGTGTATTGTTTATTACGACCCTTTGAAGTTGATT
TCTAAACTTGGTGCTAAACACATCAAGGATTGGGATCACTTAGAAGAGTTCAGAAGATCCCTTTGTGATGTTG
CAATTTCGTTGAACAACTGTGCGTATTACACGCAGTTGGACGACGCTGTGAGTGAGGTCCATAAAACCGCACC
CCCGGGTTCGTTTGTTTATAAAAGTTTAGTTAAATATCTGTCCGATAAGGTTCTTTTTAGAAGTTTGTTTATAGA
TGGCTCTTGTAAAGGGTAAAGTCAATATTAATGAGTTCATAGACTTGTCAAAATCAGAAAAATTTCTTCCGTCT
ATGTTCACACCTGTTAAGAGTGTCATGATCTCCAAGGTTGATAAGATATTGGTTCATGAAGATGAATCTTTGTC
CGAAGTCAATTTACTCAAAGGTGTAAAACTCATTAATGGTGGCTATGTACATCTTGCTGGTCTTGTAGTGACAG
GTGAATGGAATTTGCCAGATAATTGTCGTGGTGGTGTCAGTGTCTGTTTGGTCGATAAGAGAATGGAGAGAG
CGGACGAGGCAACTCTTGCTTCATACTATACCGCAGCGGCTAAGAAAAGGTTTCAGTTCAAAGTCGTTCCAAA
TTACAACATCACTACCAAGGACGCAGAAAAGGCAGTTTGGCAAGTACTAGTTAATATTAGAAATGTTAAAATT
GCTGCGGGTTACTGTCCGCTGTCATTAGAATTTGTGTCAGTGTGTATTGTTTATAAAAATATTATAAAACTCGG
TTTGAGAGAGAAAATTACGAGCGTCACGGAAGGAGGGCCCATGGAACTATCAGAAGAAGTTGTTGATGAGTT
CATGGAAGAAGTCCCGATGTCTGTAAGGCTTGCAAAATTTCGTTCGAAGACCGGGAAAAAGTTTAGTAGTAA
AAGTGAGAATAATAGTGGTAATAATAGGCCGAAACCAGACAAAAACCAAAGGAAGGAAAAGGGTTTAAAAG
TTAGGGTTGAGAAGGATAATTTAATTGATAATGAATTGGAGACTTACGTCGCCGATTCAGATTCGTATTAAAT
ATGTCTTACACAATCGCAACTCCATCGCAATTTGTGTTTTTGTCATCAGCATGGGCCGACCCTATAGAATTAATA
AATTTATGTACTAATTCACTAGGCAATCAGTTCCAAACACAACAAGCTAGAACAACCGTTCAACGGCAATTTAG
CGAAGTGTGGAAACCTGTCCCTCAAGTCACTGTTAGGTTTCCTGACAGTGGTTTTAAGGTGTATAGGTACAAT
GCGGTACTAGATCCTCTAGTTACTGCTTTGTTGGGAGCTTTCGATACTAGAAATAGGATTATAGAAGTCGAAA
ATCAGGCGAACCCGACAACCGCCGAAACGTTAGACGCTACTCGTAGAGTAGATGACGCAACGGTGGCTATAA
GGAGCGCTATAAATAATTTAGTAGTAGAATTGGTCAAAGGAACAGGTTTGTACAATCAGAGCACATTTGAAA
GTGCATCCGGTTTACAATGGTCCTCTGCACCTGCATCTTGA

MN882043

ATGGCATACACACAGACAGCTACCACATCCGCTTTGCTCGACACTGTCCGAGGTAACAATACCTTGGTCAATG
ATCTTGCGAAGCGGCGTCTTTATGACACAGCGGTCGACGAGTTCAACGCTCGTGATCGCAGGCCCAAAGTAA
ATTTTTCCAAAGTAATAAGTGAGGAACAGACGCTTATTGCTACTAGGGCATATCCAGAATTCCAGATAACCTTC
TATAATACGCAGAACGCCGTGCATTCGCTTGCCGGTGGACTACGATCCTTAGAACTGGAATATCTAATGATGC
AGATCCCGTACGGATCACTCACATATGATATAGGTGGGAATTTTGCATCACATCTGTTCAAAGGACGGGCATA
TGTTCACTGCTGTATGCCCAATCTTGATGTCCGCGACATAATGCGGCACGAAGGCCAGAAAGACAGTATAGAA
TTATACCTTTCCAGGCTTGAGCGGGGCAACAAAGTTGTCCCAAATTTCCAAAAGGAAGCTTTTGACAGATACG
CTGAAACGCCAGACGAAGTTGTCTGTCACAGTACCTTCCAAACGTGTACGCACCAGCAGGTGGAAAACACAG
GCAGGGTGTATGCTATTGCATTGCACAGTATATACGATATACCTGCTGATGAATTCGGAGCGGCACTTTTAAG
GAAAAATGTCCATGTTTGTTACGCCGCCTTCCACTTTTCCGAGAATTTACTTCTCGAAGATTCACACGTCAACCT
TGACGAAATCAACGCGTGTTTTTCGCGTGATGGAGACAAGCTGACTTTTTCTTTCGCATCTGAGAGCACTTTAA
ATTATTGTCATAGTTATTCTAATATTTTAAAATACGTGTGCAAAACTTACTTCCCGGCATCTAATAGAGAGGTCT
ACATGAAGGAGTTTTTAGTCACCAGGGTTAACACCTGGTTTTGTAAGTTTTCTAGGATTGATACTTTTTTATTAT
ACAAGGGGGTAGCCCACAAAGGTGTAAATAGTGAGCAATTTTACAGCGCAATGGAAGATGCATGGCACTACA
AAAAGACTCTTGCAATGTGTAACAGCGAGAGGATTCTTCTTGAAGATTCCTCATCGGTCAATTACTGGTTCCCA
AAAATGAGAGATATGGTCATAGTTCCTCTATTCGACATATCTCTCGACACCAGTAAAAGGACCCGCAAAGAAG
TCTTAGTGTCAAAGGATTTTGTATTCACAGTTTTAAATCACATTCGCACTTATCAAGCCAAGGCACTTACATACT
CCAATGTTTTATCCTTTGTCGAATCAATTCGTTCAAGGGTAATTATCAACGGAGTGACTGCTAGGTCTGAGTGG
GATGTTGACAAATCTCTTTTGCAATCCTTGTCCATGACATTTTTCTTGCATACTAAGCTTGCCGTTTTAAAAGAC
GAATTGTTAATCAGCAAGTTTAGTTTGGGGCCAAAATCAGTAAGCCAGCATGTATGGGATGAGATTTCCCTGG
CTTTTGGAAACGCATTTCCATCGATCAAGGAGAGACTGCTAAATCGGAAACTAATTAAAGTGTCGGGAGACGC
ATTAGAAATCAGGGTGCCTGATTTATATGTGACTTTTCACGATAGATTAGTGACTGAGTACAAAACATCGGTG
GATATGCCAGTGCTTGATATCAGAAAGAGAATGGAGGAGACTGAGGTTATGTACAATGCATTGTCTGAGCTA
TCTGTGCTCAAGGAGTCGGACAAGTTCGACGTTGATGTTTTTTCCCGGATGTGCCAGACTTTGGAGGTAGACC
CAATGACTGCAGCAAAGGTTATAGTGGCAGTGATGAGCAACGAGAGCGGACTGACTCTTACATTCGAACAGC
CAACTGAAGCAAATGTCGCATTGGCACTTAAAGATTCAGAAAAAGCCTCTGAGGGTGCACTAGTGGTTACTTC
TAGAGATGTTGAAGAACCATCCATGAAGGGTTCAATGGCAAGAGGAGAGTTACAATTGGCCGGTCTGTCTGG
AGACCAACCAGAGTCTTCCTATACTCGGAACGAGGAAATAGAGTCATTAGAGCAATTCCACATGGCAACGGCT
AGTTCGTTAATTCGGAAACAGATGAGTTCGATTGTGTACACGGGCCCCATTAAAGTTCAGCAAATGAAAAACT
TTATTGATAGCCTGGTAGCATCACTCTCTGCTGCGGTGTCGAACCTAGTCAAGATCCTAAAGGATACAGCTGCT
ATAGATCTCGAAACCCGTCAGAAGTTTGGAGTCTTAGATGTTGCGACCAAAAGATGGTTAATTAAACCTTTAG
CCAAAAATCACGCATGGGGCGTTATTGAAACACATGCTAGGAAGTACCACGTTGCACTTTTGGAGTATGATGA
GCATGGAGTGGTAACTTGCGACAGTTGGAGAAGGGTGGCCGTGAGTTCTGAGTCAATGGTTTATTCTGATAT
GGCAAAGCTCAGAACACTGAGGAGATTATTAAGAGATGGTGAGCCTCATGTCAGCAGTGCTAAAGTCGTCCT
AGTTGACGGTGTCCCGGGTTGTGGAAAGACAAAAGAGATTCTCTCGAAAGTAAATTTTGAGGAAGATCTAAT
CTTAGTACCGGGTAAGCAGGCTGCTGAAATGATAAAGAGGCGTGCTAATGCGTCAGGAATAATTCAAGCCAC
AAGAGATAATGTTCGTACTGTTGATTCATTTATAATGAATTACGGTAAAGGAACACGCTGTCAGTTCAAAAGG
TTATTTATCGACGAAGGTCTGATGTTGCACACTGGTTGTGTGAATTTTCTTGTTTCTATGTCTCTGTGCGAAATT
GCATATGTATATGGAGACACACAACAAATTCCATACATCAACAGAGTATCCGGTTTTCCGTACCCTGCACATTT
TGCAAAAATAGAGGTTGATGAGGTGGAAACTCGCAGAACTACGCTGCGTTGTCCAGCCGACATTACCCACTAT
CTTAACAGAAGGTACGAAGGACATGTCATGTGTACATCGTCGGTTAAAAAGTCAGTTTCTCAGGAAATGGTGA
GCGGGGCCGCAATGATCAATCCTGTATCTAAGCCATTGAATGGGAAAGTTTTGACTTTCACTCAGTCCGATAA
AGAGGCGCTGCTTTCTCGAGGATATACGGACGTCCATACAGTACATGAGGTACAAGGTGAGACATATGCAGA
TGTGTCGTTGGTCAGATTGACTCCGACACCTGTATCTATCATCGCAGGAGATAGTCCGCACGTTCTCGTAGCTT
TGTCAAGGCATACCCAAACATTGAAGTATTACACCGTAGTGATGGATCCTCTTGTAAGTATAATTAGGGATTTA

GAAAAACTTAGTTCTTACTTGTTAGATATGTATAAAGTAGATGCAGGGACCCAATAGCAATTACTGGTAGACT
CCGTGTTTAAAGGTTCTAATCTTTTTGTTGCAGCACCAAAGACTGGAGATATCTCAGATATGCAATTTTACTAT
GATAAGTGTCTCCCAGGTAATAGCACCATGTTAAATAACTATGATGCTGTTACCATGAGGTTGACTGACATTTC
TCTTAATGTCAAAGATTGCATATTGGATTTCTCTAAGTCTGTGGCTGCACCGAAGGATCCGATCAAACCACTGA
TTCCAATGGTACGAACGGCGGCAGAAATGCCACGCCAGACTGGACTATTGGAAAATTTGGTGGCGATGATCA
AAAGAAACTTTAATTCACCGGAGTTATCAGGAATAATCGACATTGAGAATACTGCATCTTTGGTAGTAGATAA
ATTTTTTGATAGTTACTTGCTTAAAGAAAAAAGAAAACCAAATAAAAATGTTTCTTTATTTTGTAGAGAGTCTCT
CAATAGATGGTTAGAGAAGCAGGAGCAAGTGACCATTGGTCAGCTTGCAGATTTTGATTTTGTGGATCTTCCT
GCCGTTGATCAGTACAGGCATATGATTAAAGCGCAACCTAAGCAGAAGCTGGATACATCAATTCAAAGCGAAT
ATCCGGCCTTGCAGACGATTGTGTATCATTCGAAAAAGATCAACGCAATCTTCGGTCCTTTGTTCAGTGAGCTC
ACAAGGCAAATGCTCGAAAGCATAGACTCAAGTAAGTTTTTGTTCTTTACAAGGAAGACGCCAGCTCAAATCG
AGGATTTCTTCGGAGATCTCGATAGCCATGTCCCTATGGATATCTTGGAGTTGGATATTTCGAAGTATGACAAA
TCTCAGAACGAGTTCCACTGTGCAGTAGAGTATGAAATATGGAGAAGACTTGGATTAGAAGATTTTCTGGGA
GAAGTTTGGAAACAAGGCCACAGAAAAACTACTCTTAAAGATTACACAGCTGGTATTAAAACGTGTTTATGGT
ACCAGAGAAAGAGTGGGGACGTTACAACATTCATCGGTAATACGGTGATTATTGCTGCTTGTTTAGCTTCCAT
GTTGCCCATGGAGAAAATAATCAAAGGTGCATTTTGCGGAGATGACAGTTTACTATACTTCCCAAAAGGTTGT
GAGTTTCCCGACATACAGCATACAGCTAACCTTATGTGGAATTTCGAGGCTAAGCTATTCAGAAAGCAGTATG
GTTATTTCTGTGGAAGGTACGTGATACATCATGACAGAGGGTGTATTGTTTATTACGACCCTTTGAAGTTGATT
TCTAAACTTGGTGCTAAACACATCAAGGATTGGGATCACTTAGAAGAGTTCAGAAGATCCCTTTGTGATGTTG
CAATTTCGTTGAACAACTGTGCGTATTACACGCAGTTGGACGACGCTGTGAGTGAGGTCCATAAAACCGCACC
CCCGGGTTCGTTTGTTTATAAAAGTTTAGTTAAATATCTGTCCGATAAGGTTCTTTTTAGAAGTTTGTTTATAGA
TGGCTCTTGTAAAGGGTAAAGTCAATATTAATGAGTTCATAGACTTGTCAAAATCAGAAAAATTTCTTCCGTCT
ATGTTCACACCTGTTAAGAGTGTCATGATCTCCAAGGTTGATAAGATATTGGTTCATGAAGATGAATCTTTGTC
CGAAGTCAATTTACTCAAAGGTGTAAAACTCATTAATGGTGGCTATGTACATCTTGCTGGTCTTGTAGTGACAG
GTGAATGGAATTTGCCAGATAATTGTCGTGGTGGTGTCAGTGTCTGTTTGGTCGATAAGAGAATGGAGAGAG
CGGACGAGGCAACTCTTGCTTCATACTATACCGCAGCGGCTAAGAAAAGGTTTCAGTTCAAAGTCGTTCCAAA
TTACAACATCACTACCAAGGACGCAGAAAAGGCAGTTTGGCAAGTACTAGTTAATATTAGAAATGTTAAAATT
GCTGCGGGTTACTGTCCGCTGTCATTAGAATTTGTGTCAGTGTGTATTGTTTATAAAAATATTATAAAACTCGG
TTTGAGAGAGAAAATTACGAGCGTCACGGAAGGAGGGCCCATGGAACTATCAGAAGAAGTTGTTGATGAGTT
CATGGAAGAAGTCCCGATGTCTGTAAGGCTTGCAAAATTTCGTTCGAAGACCGGGAAAAAGTTTAGTAGTAA
AAGTGAGAATAATAGTGGTAATAATAGGCCGAAACCAGACAAAAACCAAAGGAAGGAAAAGGGTTTAAAAG
TTAGGGTTGAGAAGGATAATTTAATTGATAATGAATTGGAGACTTACGTCGCCGATTCAGATTCGTATTAAAT
ATGTCTTACACAATCGCAACTCCATCGCAATTTGTGTTTTTGTCATCAGCATGGGCCGACCCTATAGAATTAATA
AATTTATGTACTAATTCACTAGGCAATCAGTTCCAAACACAACAAGCTAGAACAACCGTTCAACGGCAATTTAG
CGAAGTGTGGAAACCTGTCCCTCAAGTCACTGTTAGGTTTCCTGACAGTGGTTTTAAGGTGTATAGGTACAAT
GCGGTACTAGATCCTCTAGTTACTGCTTTGTTGGGAGCTTTCGATACTAGAAATAGGATTATAGAAGTCGAAA
ATCAGGCGAACCCGACAACCGCCGAAACGTTAGACGCTACTCGTAGAGTAGATGACGCAACGGTGGCTATAA
GGAGCGCTATAAATAATTTAGTAGTAGAATTGGTCAAAGGAACAGGTTTGTACAATCAGAGCACATTTGAAA
GTGCATCCGGTTTACAATGGTCCTCTGCACCTGCATCTTGA

MN882045

ATGGCATACACACAGACAGCTACCACATCCGCTTTGCTCGACACTGTCCGAGGTAACAATACCTTGGTCAACG
ATCTTGCGAAGCGGCGTCTTTATGACACAGCGGTCGACGAGTTCAACGCTCGTGATCGCAGGCCCAAAGTAA
ATTTTTCCAAAGTAATAAGTGAGGAACAGACGCTTATTGCTACTAGGGCATATCCTGAATTCCAGATAACCTTC
TATAATACGCAGAACGCCGTGCATTCGCTTGCCGGTGGACTACGATCCTTAGAACTGGAATATCTAATGATGC
AGATCCCGTACGGATCACTCACATATGATATAGGTGGGAATTTTGCATCTCATCTGTTCAAAGGACGGGCATA

TGTTCACTGCTGTATGCCCAATCTTGATGTCCGCGACATAATGCGGCACGAAGGCCAGAAAGACAGTATAGAA
TTATACCTTTCCAGGCTTGAGCGGGGCAACAAAGTTGTCCCAAATTTCCAAAAGGAAGCTTTTGACAGATACG
CTGAAACGCCAGACGAAGTTGTCTGTCACAGTACCTTCCAAACGTGTACGCACCAGCAGGTGGAAAACACAG
GCAGGGTGTATGCTATTGCATTGCACAGTATATACGATATACCTGCTGATGAATTCGGAGCGGCACTTTTAAG
GAAAAATGTCCATGTTTGTTACGCCGCCTTCCACTTTTCCGAGAATTTACTTCTCGAAGATTCACACGTCAACCT
TGACGAAATCAACGCGTGTTTTTCGCGTGATGGAGACAAGCTGACTTTTTCTTTCGCATCTGAGAGCACTTTAA
ATTATTGTCATAGTTATTCTAATATTTTAAAATACGTGTGCAAAACTTACTTCCCGGCATCTAATAGAGAGGTCT
ACATGAAGGAGTTTTTGGTCACCAGGGTTAACACCTGGTTTTGTAAGTTTTCTAGGATAGATACTTTTTTATTAT
ACAAGGGGGTAGCCCACAAAGGTGTAAATAGTGAGCAATTTTACAGCGCAATGGAAGATGCATGGCACTACA
AAAAGACTCTTGCAATGTGTAACAGCGAGAGGATTCTTCTTGAAGATTCCTCATCGGTCAATTACTGGTTCCCA
AAAATGAGAGATATGGTCATAGTTCCTCTATTCGACATATCTCTCGACACTAGTAAAAGGACCCGCAAAGAAG
TCTTAGTGTCAAAGGATTTTGTATTCACAGTTTTAAATCACATTCGCACTTATCAAGCCAAGGCACTTACATACT
CCAATGTTTTATCCTTTGTCGAATCAATTCGTTCAAGGGTAATTATCAACGGAGTGACTGCCAGGTCTGAGTGG
GATGTTGACAAATCTCTTTTGCAATCCTTGTCCATGACATTTTTCTTGCATACTAAGCTTGCCGTTTTAAAAGAC
GAATTGTTAATCAGCAAGTTTAGTTTGGGGCCAAAATCAGTAAGCCAGCATGTATGGGATGAGATTTCCCTGG
CTTTTGGAAACGCATTTCCATCGATCAAGGAGAGACTACTAAATCGGAAACTAATTAAAGTGTCGGGAGACGC
ATTAGAAATCAGGGTGCCTGATTTATATGTGACTTTTCACGATAGATTAGTGACTGAGTACAAAACATCGGTG
GATATGCCAGTGCTTGATATCAGAAAGAGAATGGAGGAGACTGAGGTTATGTACAATGCATTGTCTGAGCTA
TCTGTGCTTAAGGAGTCGGACAAGTTCGACGTTGATGTTTTTTCCCGGATGTGCCAGACTTTGGAGGTAGACC
CAATGACTGCAGCAAAGGTTATTGTGGCAGTGATGAGCAACGAGAGCGGACTGACTCTTACATTCGAACAGC
CAACTGAAGCAAATGTCGCATTGGCACTTAAAGATTCAGAAAAAGCCTCTGAGGGTGCACTAGTGGTTACTTC
TAGAGATGTTGAAGAACCATCCATGAAGGGTTCAATGGCAAGAGGAGAGTTACAATTGGCCGGTCTGTCTGG
AGACCAACCAGAGTCTTCCTATACTCGGAACGAGGAAATAGAGTCATTAGAGCAATTCCACATGGCAACGGCT
GGTTCGTTAATTCGGAAACAGATGAGTTCGATTGTGTACACGGGCCCCATTAAAGTTCAGCAAATGAAAAACT
TTATTGATAGCCTGGTAGCATCACTCTCTGCTGCGGTGTCGAACCTAGTCAAGATCCTAAAGGATACAGCTGCT
ATAGATCTCGAAACCCGTCAGAAGTTTGGAGTCTTAGATGTTGCGACCAAAAGATGGTTAATTAAACCTTTAG
CCAAGAATCACGCATGGGGCGTTATTGAAACACATGCTAGGAAGTACCACGTTGCACTTTTGGAGTATGATGA
GCATGGAGTGGTAACTTGCGACAGTTGGAGAAGGGTGGCCGTGAGTTCTGAGTCAATGGTTTATTCTGATAT
GGCAAAGCTCAGAACACTGAGGAGATTATTAAGAGATGGTGAGCCTCATGTCAGCAGTGCTAAAGTCGTCCT
AGTTGACGGTGTCCCGGGTTGTGGAAAGACAAAAGAGATTCTCTCGAAAGTAAATTTTGAGGAAGATCTAAT
CTTAGTACCGGGTAAGCAGGCTGCTGAAATGATAAAGAGGCGTGCTAATGCGTCAGGAATAATTCAAGCCAC
AAGAGATAATGTTCGTACTGTTGATTCATTTATAATGAATTACGGTAAAGGAACACGCTGTCAGTTCAAAAGG
TTATTTATCGACGAAGGTCTGATGTTGCACACTGGTTGTGTGAATTTTCTTGTTTCTATGTCTCTGTGCGAAATT
GCATATGTTTATGGAGACACACAACAAATTCCATACATCAACAGAGTATCCGGTTTTCCGTACCCTGCGCATTT
TGCAAAAATAGAGGTTGATGAGGTGGAAACTCGCAGAACTACGCTGCGTTGTCCAGCCGACATTACCCACTAT
CTTAACAGAAGGTACGAAGGACATGTCATGTGTACATCGTCGGTTAAAAAGTCAGTTTCTCAGGAAATGGTGA
GCGGGGCCGCAATGATCAATCCTGTATCTAAGCCATTGAATGGGAAAGTTTTGACTTTCACTCAGTCTGATAA
AGAGGCGCTGCTTTCTCGAGGATATACGGACGTCCATACAGTACATGAGGTACAAGGTGAGACATATGCAGA
TGTGTCGTTGGTCAGATTGACTCCGACACCTGTATCTATCATCGCAGGAGATAGTCCGCACGTTCTCGTAGCTT
TGTCAAGGCATACCCAAACATTGAAGTATTACACCGTAGTGATGGATCCTCTTGTAAGTATAATTAGGGATTTA
GAAAAACTTAGTTCTTACTTGTTAGATATGTATAAAGTAGATGCAGGGACCCAATAGCAATTACAGGTAGACT
CCGTGTTTAAAGGTTCTAATCTTTTTGTTGCAGCACCAAAGACTGGAGATATCTCAGATATGCAATTTTACTAT
GATAAGTGTCTCCCAGGTAATAGCACCATGTTAAATAACTATGATGCTGTTACCATGAGGTTGACTGACATTTC
TCTTAATGTCAAAGATTGCATATTGGATTTCTCTAAGTCTGTGGCTGCACCGAAGGATCCGATCAAACCACTGA
TTCCAATGGTACGAACGGCGGCAGAAATGCCACGCCAGACTGGACTATTGGAAAATTTGGTGGCGATGATCA
AAAGAAACTTTAATTCACCGGAGTTATCAGGAATAATCGACATTGAGAATACTGCATCTTTAGTAGTAGATAA
ATTTTTTGATAGTTACTTGCTTAAAGAAAAAAGAAAACCAAATAAAAATGTTTCTTTATTTTGTAGAGAGTCTCT
CAATAGATGGTTAGAGAAGCAGGAGCAAGTGACCATTGGTCAGCTTGCAGATTTTGATTTTGTGGATCTTCCT

GCCGTTGATCAGTACAGGCATATGATTAAAGCGCAACCTAAGCAGAAGCTGGATACATCAATTCAAAGCGAAT
ATCCGGCCTTGCAGACGATTGTGTATCATTCGAAAAAGATCAACGCAATCTTCGGTCCTTTGTTCAGTGAGCTC
ACAAGGCAAATGCTCGAAAGCATAGACTCAAGTAAGTTTTTGTTCTTTACAAGGAAGACGCCAGCTCAAATTG
AGGATTTCTTCGGAGATCTCGATAGCCATGTCCCTATGGATATCTTGGAGTTGGATATTTCGAAGTATGACAAA
TCTCAGAACGAGTTCCACTGTGCAGTAGAGTATGAAATATGGAGAAGACTTGGATTAGAAGATTTTCTGGGA
GAAGTTTGGAAACAAGGCCACAGGAAAACTACTCTTAAAGATTACACAGCTGGTATTAAAACGTGTTTATGGT
ACCAGAGAAAGAGTGGGGACGTTACAACATTCATCGGTAATACGGTGATTATTGCTGCTTGTTTAGCTTCCAT
GTTGCCCATGGAGAAAATAATCAAAGGTGCATTTTGCGGAGATGACAGTTTACTATACTTCCCAAAAGGTTGT
GAGTTTCCTGACATACAGCATACAGCCAACCTTATGTGGAATTTCGAGGCTAAGCTATTCAGAAAGCAGTATG
GTTATTTCTGTGGAAGGTACGTGATACATCATGACAGAGGGTGTATTGTTTATTATGACCCTTTGAAGTTGATT
TCTAAACTTGGTGCTAAACACATCAAGGATTGGGATCACTTAGAAGAGTTCAGAAGATCCCTTTGTGATGTTG
CAAATTCGTTGAACAACTGTGCGTATTACACGCAGTTGGACGACGCTGTGAGTGAGGTCCATAAAACCGCACC
CCCGGGTTCGTTTGTATATAAAAGTTTAGTTAAATATCTGTCCGATAAGGTTCTTTTTAGAAGTTTGTTTATAGA
TGGCTCTTGTTAAGGGTAAAGTCAATATTAATGAGTTCATAGACTTGTCAAAATCAGAAAAATTTCTTCCGTCT
ATGTTCACACCTGTTAAGAGTGTCATGATCTCCAAGGTTGATAAGATATTGGTTCATGAAGATGAATCTTTGTC
CGAAGTCAATTTACTCAAAGGTGTAAAACTCATTGATGGTGGCTATGTACATCTTGCTGGTCTTGTGGTGACA
GGTGAATGGAATTTGCCAGATAATTGTCGTGGTGGTGTCAGTGTCTGTTTGGTCGATAAAAGAATGGAGAGA
GCGGACGAGGCAACTCTTGCTTCATACTATACCGCAGCGGCTAAGAAAAGGTTTCAGTTCAAAGTCGTTCCAA
ATTACAACATCACTACCAAGGACGCAGAAAAGGCAGTTTGGCAAGTACTAGTTAATATTAGAAATGTTAAAAT
TGCTGCGGGTTACTGTCCGCTGTCATTAGAATTTGTGTCAGTGTGTATTGTTTATAAAAATATTATAAAACTCG
GTTTGAGAGAGAAAATTACGAGCGTCACGGATGGAGGGCCCATGGAACTATCAGAAGAAGTTGTTGATGAG
TTCATGGAAGAAGTCCCGATGTCTGTAAGGCTTGCAAAATTTCGTTCGAAGACCGGGAAAAAGTTTAGTAGTA
AAAGTGAGAATAATAGTGGTAATAATAGGCCGAAACCAAACAAAAACCAAAGGAAGGAAAAGGGTTTAAAA
GTTAGGGTTGAGAAGGATAATTTAATTGATAATGAATTGGAGACTTACGTCGCCGATTCAGATTCGTATTAAA
TATGTCTTACACAATCGCAACTCCATCGCAATTTGTGTTTTTGTCATCAGCATGGGCCGACCCTATAGAATTAAT
AAATTTATGTACTAATTCACTAGGTAATCAATTCCAAACACAACAAGCTAGAACAACCGTTCAACGGCAATTTA
GCGAAGTGTGGAAACCTGTCCCTCAAGTCACTGTTAGGTTTCCTGACAGTGGTTTTAAGGTGTATAGGTACAA
TGCGGTACTAGATCCTCTAGTTACTGCTTTGTTAGGAGCTTTCGATACTAGAAATAGGATTATAGAAGTCGAAA
ATCAGGCGAACCCGACAACCGCCGAAACGTTAGACGCTACTCGTAGAGTAGATGACGCAACGGTGGCTATAA
GGAGCGCTATAAATAATTTAGTAGTAGAATTGGTCAAAGGAACAGGTTTGTACAATCAGAGCACATTTGAAA
GTGCATCCGGTTTACAATGGTCCTCTGCACCTGCATCTTGA

MN882050

ATGGCATACACACAGACAGCTACCACATCCGCTTTGCTCGACACTGTCCGAGGTAACAATACCTTGGTCAACG
ATCTTGCGAAGCGGCGTCTTTATGACACAGCGGTCGACGAGTTCAACGCTCGTGATCGCAGGCCCAAAGTAA
ATTTTTCCAAAGTAATAAGTGAGGAACAGACGCTTATTGCTACTAGGGCATATCCAGAATTCCAGATAACCTTT
TATAATACGCAGAACGCCGTGCATTCGCTTGCCGGTGGACTACGATCCTTAGAACTGGAATATCTAATGATGC
AGATCCCGTACGGATCACTCACATATGATATAGGTGGGAATTTTGCATCTCATCTGTTCAAAGGACGGGCATA
TGTTCACTGCTGTATGCCCAATCTTGATGTCCGCGACATAATGCGGCACGAAGGCCAGAAAGACAGTATAGAA
TTATACCTTTCCAGGCTTGAGCGGGGCAACAAAGTTGTCCCAAATTTCCAAAAGGAAGCTTTTGACAGATACG
CTGAAACGCCAGACGAAGTTGTCTGTCACAGTACCTTCCAAACGTGTACGCACCAGCAGGTGGAAAACACAG
GCAGGGTGTATGCTATTGCACTGCACAGTATATACGATATACCTGCTGATGAATTCGGAGCGGCACTTTTAAG
GAAAAATGTCCATGTTTGTTACGCCGCCTTCCACTTTTCCGAGAATTTACTTCTCGAAGATTCACACGTCAACCT
TGACGAAATCAATGCGTGTTTTTCGCGTGATGGAGACAAGCTGACTTTTTCTTTCGCATCTGAGAGCACTTTAA
ATTATTGTCATAGTTATTCTAATATTTTAAAATACGTGTGCAAAACTTACTTCCCGGCATCTAATAGAGAGGTCT
ACATGAAGGAGTTTTTGGTCACCAGGGTTAACACCTGGTTTTGTAAGTTTTCTAGGATAGATACTTTTTTATTAT

ACAAGGGGGTAGCCCACAAAGGTGTAAATAGTGAGCAATTTTACAGCGCAATGGAAGATGCATGGCACTACA
AAAAGACTCTTGCAATGTGTAACAGCGAGAGGATTCTTCTTGAAGATTCCTCATCGGTCAATTACTGGTTCCCA
AAAATGAGAGATATGGTCATAGTTCCTCTATTCGACATATCTCTCGACACCAGTAAAAGGACCCGCAAAGAAG
TCTTAGTGTCAAAGGATTTTGTATTCACAGTTTTAAATCACATTCGCACTTATCAAGCCAAGGCACTTACATACT
CCAATGTTTTATCCTTTGTCGAATCAATTCGTTCAAGGGTAATTATCAACGGAGTGACTGCCAGGTCTGAGTGG
GATGTTGACAAATCTCTTTTGCAATCCTTGTCCATGACATTTTTCTTGCATACTAAGCTTGCCGTTTTAAAAGAC
GAATTGTTAATCAGCAAGTTTAGTTTGGGGCCAAAATCAGTAAGCCAGCATGTATGGGATGAGATTTCCCTGG
CTTTTGGAAACGCATTTCCATCGATCAAGGAGAGACTGCTAAATCGGAAACTAATTAAAGTGTCGGGAGACGC
ATTAGAAATCAGGGTGCCTGATTTATATGTGACTTTTCACGATAGATTAGTGACTGAGTACAAAACATCGGTG
GATATGCCAGTGCTTGATATCAGAAAGAGAATGGAGGAGACTGAGGTTATGTACAATGCATTGTCTGAGCTA
TCTGTGCTCAAGGAGTCGGACAAGTTCGACGTTGATGTTTTTTCCCGGATGTGCCAGACTTTGGAGGTAGACC
CAATGACTGCAGCAAAGGTTATTGTGGCAGTGATGAGCAACGAGAGCGGACTGACTCTTACATTCGAACAGC
CAACTGAAGCAAATGTCGCATTGGCACTTAAAGATTCAGAAAAAGCCTCTGAGGGTGCACTAGTGGTTACTTC
TAGAGATGTTGAAGAACCATCCATGAAGGGTTCAATGGCAAGAGGAGAGTTACAATTGGCCGGTCTGTCTGG
AGACCAACCAGAGTCTTCCTATACTCGGAACGAGGAAATAGAGTCATTAGAGCAATTCCACATGGCAACGGCT
AGTTCGTTAATTCGGAAACAGATGAGTTCGATTGTGTACACGGGCCCCATTAAAGTTCAGCAAATGAAAAACT
TTATTGATAGCCTGGTAGCATCACTCTCTGCTGCGGTGTCGAACCTAGTCAAGATCCTAAAGGATACAGCTGCT
ATAGATCTCGAAACCCGTCAGAAGTTTGGAGTCTTAGATGTTGCGACCAAAAGATGGTTAATTAAACCTTTAG
CCAAGAATCACGCATGGGGCGTTATTGAAACACATGCTAGGAAGTACCACGTTGCACTTTTGGAGTATGATGA
GCATGGAGTGGTAACTTGCGACAGTTGGAGAAGGGTGGCCGTGAGTTCTGAGTCAATGGTTTATTCTGATAT
GGCAAAGCTCAGAACACTGAGGAGATTATTAAGAGATGGTGAGCCTCATGTCAGCAGTGCTAAAGTCGTCCT
AGTTGACGGTGTCCCGGGTTGTGGAAAGACAAAAGAGATTCTCTCGAAAGTAAATTTTGAGGAAGATCTAAT
CTTAGTACCGGGTAAGCAGGCTGCTGAAATGATAAAGAGGCGTGCTAATGCGTCAGGAATAATTCAAGCCAC
AAGAGATAATGTTCGTACTGTTGATTCATTTATAATGAATTACGGTAAAGGAACACGCTGTCAGTTCAAAAGG
TTATTTATCGACGAAGGTCTGATGTTGCACACTGGTTGTGTGAATTTTCTTGTTTCTATGTCTCTGTGCGAAATT
GCATATGTTTATGGAGACACACAACAAATTCCATACATCAACAGAGTATCCGGTTTTCCGTACCCTGCACATTT
TGCAAAAATAGAGGTTGATGAGGTGGAAACTCGCAGAACTACGCTGCGTTGTCCAGCCGACATTACCCACTAT
CTTAACAGAAGGTACGAAGGACATGTCATGTGTACATCGTCGGTTAAAAAGTCAGTTTCTCAGGAAATGGTGA
GCGGGGCCGCAATGATCAATCCTGTATCTAAGCCATTGAATGGGAAAGTTTTGACTTTCACTCAGTCTGATAA
AGAGGCGCTGCTTTCTCGAGGATATACGGACGTCCATACAGTACATGAGGTACAAGGTGAGACATATGCAGA
TGTGTCGTTGGTCAGATTGACTCCGACACCTGTATCTATCATCGCAGGAGATAGTCCGCACGTTCTCGTAGCTT
TGTCAAGGCATACCCAAACATTGAAGTATTACACCGTAGTGATGGATCCTCTTGTAAGTATAATTAGGGATTTA
GAAAAACTTAGTTCTTACTTGTTAGATATGTATAAAGTAGATGCAGGGACCCAATAGCAATTACAGGTAGACT
CCGTGTTTAAAGGTTCTAATCTTTTTGTTGCAGCACCAAAGACTGGAGATATCTCAGATATGCAATTTTACTAT
GATAAGTGTCTCCCAGGTAATAGCACCATGTTAAATAACTATGATGCTGTTACCATGAGGTTGACTGACATTTC
TCTTAATGTCAAAGATTGCATATTGGATTTCTCTAAGTCTGTGGCTGCACCGAAGGATCCGATCAAACCACTGA
TTCCAATGGTACGAACGGCGGCAGAAAAGCCACGCCAGACTGGACTATTGGAAAATTTGGTGGCGATGATCA
AAAGAAACTTTAATTCACCGGAGTTATCAGGAATAATCGACATTGAGAATACTGCATCTTTAGTAGTAGATAA
ATTTTTTGATAGTTACTTGCTTAAAGAAAAAAGAAAACCAAATAAAAATGTTTCTTTATTTTGTAGAGAGTCTCT
CAATAGATGGTTAGAGAAGCAGGAGCAAGTGACCATTGGTCAGCTTGCAGATTTTGATTTTGTGGATCTTCCT
GCCGTTGATCAGTACAGGCATATGATTAAAGCGCAACCTAAGCAGAAGCTGGATACATCAATTCAAAGCGAAT
ATCCGGCCTTGCAGACGATTGTGTATCATTCGAAAAAGATCAACGCAATCTTCGGTCCTTTGTTCAGTGAGCTC
ACAAGGCAAATGCTCGAAAGCATAGACTCAAGTAAGTTTTTGTTCTTTACAAGGAAGGCGCCAGCTCAAATTG
AGGATTTCTTCGGAGATCTCGATAGCCATGTCCCTATGGATATCTTGGAGTTGGATATTTCGAAGTATGACAAA
TCTCAGAACGAGTTCCACTGTGCAGTAGAGTATGAAATATGGAGAAGACTTGGATTAGAAGATTTTCTGGGA
GAAGTTTGGAAACAAGGCCACAGAAAAACTACTCTTAAAGATTACACAGCTGGTATTAAAACGTGTTTATGGT
ACCAGAGAAAGAGTGGGGACGTTACAACATTCATCGGTAATACGGTGATTATTGCTGCTTGTTTAGCATCCAT
GTTGCCCATGGAGAAAATAATCAAAGGTGCATTTTGCGGAGATGACAGTTTACTATACTTCCCAAAAGGTTGT

GAGTTTCCTGACATACAGCATACAGCCAACCTTATGTGGAATTTCGAGGCTAAGCTATTCAGAAAGCAGTATG
GTTATTTCTGTGGAAGGTACGTGATACATCACGACAGAGGGTGTATTGTTTATTATGACCCTTTGAAGTTGATT
TCTAAACTTGGTGCTAAACACATCAAGGATTGGGATCACTTAGAAGAGTTCAGAAGATCCCTTTGTGATGTTG
CAAATTCGTTGAACAACTGTGCGTATTACACGCAGTTGGACGACGCTGTGAGTGAGGTCCATAAAACCGCACC
CCCGGGTTCGTTTGTATATAAAAGTTTAGTTAAATATCTGTCCGATAAGGTTCTTTTTAGAAGTTTGTTTATAGA
TGGCTCTTGTTAAGGGTAAAGTCAATATTAATGAGTTCATAGACTTGTCAAAATCAGAAAAATTTCTTCCGTCT
ATGTTCACACCTGTTAAGAGTGTCATGATCTCCAAGGTTGATAAGATATTGGTTCATGAAGATGAATCTTTGTC
CGAAGTCAATTTACTCAAAGGTGTAAAACTCATTGATGGTGGCTATGTACATCTTGCTGGTCTTGTGGTGACA
GGTGAATGGAATTTGCCAGATAATTGTCGTGGTGGTGTCAGTGTCTGTTTGGTCGATAAGAGAATGGAGAGA
GCGGACGAGGCAACTCTTGCTTCATACTATACCGCAGCGGCTAAGAAAAGGTTTCAGTTCAAAGTCGTTCCAA
ATTACAACATCACTACCAAGGACGCAGAAAAGGCAGTTTGGCAAGTACTAGTTAATATTAGAAATGTTAAAAT
TGCTGCGGGTTACTGTCCGCTGTCATTAGAATTTGTGTCAGTGTGTATTGTTTATAAAAATATTATAAAACTCG
GTTTGAGAGAGAAAATTACGAGCGTCACGGATGGAGGGCCTATGGAACTATCAGAAGAAGTTGTTGATGAGT
TCATGGAAGAAGTCCCGATGTCTGTAAGGCTTGCAAAATTTCGTTCGAAGACCGGAAAAAAGTTTAGTAGTAA
AAGTGAGAATAATAGTGGTAATAATAGGCCGAAACCAAACAAAAACCAAAGGAAGGAAAAGGGTTTAAAAG
TTAGGGTTGAGAAGGATAATTTAATTGATAATGAATTGGAGACTTACGTCGCCGATTCAGATTCGTATTAAAT
ATGTCTTACACAATCGCAACTCCATCGCAATTTGTGTTTTTGTCATCAGCATGGGCCGACCCTATAGAATTAATA
AATTTATGTACTAATTCACTAGGTAATCAGTTCCAAACACAACAAGCTAGAACAACCGTTCAACGGCAATTTAG
CGAAGTGTGGAAACCTGTCCCTCAAGTCACTGTTAGGTTTCCTGACAGTGGTTTTAAGGTGTATAGGTACAAT
GCGGTACTAGATCCTCTAGTTACTGCTTTGTTAGGAGCTTTCGATACTAGAAATAGGATTATAGAAGTCGAAA
ATCAGGCGAACCCGACAACCGCCGAAACGTTAGACGCTACTCGTAGAGTAGATGACGCAACGGTGGCTATAA
GGAGCGCTATAAATAATTTAGTAGTAGAATTGGTCAAAGGAACAGGTTTGTACAATCAGAGCACATTTGAAA
GTGCATCTGGTTTACAATGGTCCTCTGCACCTGCATCTTGA

MN882053

ATGGCATACACACAGACAGCTACCACATCCGCTTTGCTCGACACTGTCCGAGGTAACAATACCTTGGTCAACG
ATCTTGCGAAGCGGCGTCTTTATGACACAGCGGTCGACGAGTTCAACGCTCGTGATCGCAGGCCCAAAGTAA
ATTTTTCCAAAGTAATAAGTGAGGAACAGACGCTTATTGCTACTAGGGCATATCCTGAATTCCAGATAACCTTC
TATAATACGCAGAACGCCGTGCATTCGCTTGCCGGTGGACTACGATCCTTAGAACTGGAATATCTAATGATGC
AGATCCCGTACGGATCACTTACATATGATATAGGTGGGAATTTTGCATCTCATCTGTTCAAAGGACGGGCATA
TGTTCACTGCTGTATGCCCAATCTTGATGTCCGCGACATAATGCGGCACGAAGGCCAGAAAGACAGTATAGAA
TTATACCTTTCCAGGCTTGAGCGGGGCAACAAAGTTGTCCCAAATTTCCAAAAGGAAGCTTTTGACAGATACG
CTGAAACGCCAGACGAAGTTGTCTGTCACAGTACCTTCCAAACGTGTACGCACCAGCAGGTGGAAAACACAG
GCAGGGTGTATGCTATTGCATTGCACAGTATATACGATATACCTGCTGATGAATTCGGAGCGGCACTTTTAAG
GAAAAATGTCCATGTTTGTTACGCCGCCTTCCACTTTTCCGAGAATTTACTTCTCGAAGATTCACACGTCAACCT
TGACGAAATCAACGCGTGTTTTTCGCGTGATGGAGACAAGCTGACTTTTTCTTTCGCATCTGAGAGCACTTTAA
ATTATTGTCATAGTTATTCTAATATTTTAAAATACGTGTGCAAAACTTACTTCCCGGCATCTAATAGAGAGGTCT
ACATGAAGGAGTTTTTGGTCACCAGGGTTAACACCTGGTTTTGTAAGTTTTCTAGGATAGATACTTTTTTATTAT
ACAAGGGGGTAGCCCACAAAGGTGTAAATAGTGAGCAATTTTACAGCGCAATGGAAGATGCATGGCACTACA
AAAAGACTCTTGCAATGTGTAACAGCGAGAGGATTCTTCTTGAAGATTCCTCATCGGTCAATTACTGGTTCCCA
AAAATGAGAGATATGGTCATAGTTCCTCTATTCGACATATCTCTCGACACTAGTAAAAGGACCCGCAAAGAAG
TCTTAGTGTCAAAGGATTTTGTATTCACAGTTTTAAATCACATTCGCACTTATCAAGCCAAGGCACTTACATACT
CCAATGTTTTATCCTTTGTCGAATCAATTCGTTCAAGGGTAATTATCAACGGAGTGACTGCCAGGTCTGAGTGG
GATGTTGACAAATCTCTTTTGCAATCCTTGTCCATGACATTTTTCTTGCATACTAAGCTTGCCGTTTTAAAAGAC
GAATTGTTAATCAGCAAGTTTAGTTTGGGGCCAAAATCAGTAAGCCAGCATGTATGGGATGAGATTTCCCTGG
CTTTTGGAAACGCATTTCCATCGATCAAGGAGAGACTGCTAAATCGGAAACTAATTAAAGTGTCGGGAGACGC

ATTAGAAATCAGGGTGCCTGATTTATATGTGACTTTTCACGATAGATTAGTGACTGAGTACAAAACATCGGTG
GATATGCCAGTGCTTGATATCAGAAAGAGAATGGAGGAGACTGAGGTTATGTACAATGCATTGTCTGAGCTA
TCTGTGCTCAAGGAGTCGGACAAGTTCGACGTTGATGTTTTTTCCCGGATGTGCCAGACTTTGGAGGTAGACC
CAATGACTGCAGCAAAGGTTATTGTGGCAGTGATGAGCAACGAGAGCGGACTGACTCTTACATTCGAACAGC
CAACTGAAGCAAATGTCGCATTGGCACTTAAAGATTCAGAAAAAGCCTCTGAGGGTGCACTAGTGGTTACTTC
TAGAGATGTTGAAGAACCATCCATGAAGGGTTCAATGGCAAGAGGAGAGTTACAATTGGCCGGTCTGTCTGG
AGACCAACCAGAGTCTTCCTATACTCGGAACGAGGAAATAGAGTCATTAGAGCAATTCCACATGGCAACGGCT
GGTTCGTTAATTCGGAAACAGATGAGTTCGATTGTGTACACGGGCCCCATTAAAGTTCAGCAAATGAAAAACT
TTATTGATAGCCTGGTAGCATCACTCTCTGCTGCGGTGTCGAACCTAGTCAAGATCCTAAAGGATACAGCTGCT
ATAGATCTCGAAACCCGTCAGAAGTTTGGAGTCTTAGATGTTGCGACCAAAAGATGGTTAATTAAACCTTTAG
CCAAGAATCACGCATGGGGCGTTATTGAAACACATGCTAGGAAGTACCACGTTGCACTTTTGGAGTATGATGA
GCATGGAGTGGTAACTTGCGACAGTTGGAGAAGGGTGGCCGTGAGTTCTGAGTCAATGGTTTATTCTGATAT
GGCAAAGCTCAGAACACTGAGGAGATTATTAAGAGATGGTGAGCCTCATGTCAGCAGTGCTAAAGTCGTCCT
AGTTGACGGTGTCCCGGGTTGTGGAAAGACAAAAGAGATTCTCTCGAAAGTAAATTTTGAGGAAGATCTAAT
CTTAGTACCGGGTAAGCAGGCTGCTGAAATGATAAAGAGGCGTGCTAATGCGTCAGGAATAATTCAAGCCAC
AAGAGATAATGTTCGTACTGTTGATTCATTTATAATGAATTACGGTAAAGGAACACGCTGTCAGTTCAAAAGG
TTATTTATCGACGAAGGTCTGATGTTGCACACTGGTTGTGTGAATTTTCTTGTTTCTATGTCTCTGTGCGAAATT
GCATATGTTTATGGAGACACACAACAAATTCCATACATCAACAGAGTATCCGGTTTTCCGTACCCTGCACATTT
TGCAAAAATAGAGGTTGATGAGGTGGAAACTCGCAGAACTACGCTGCGTTGTCCAGCCGACATTACCCACTAT
CTTAACAGAAGGTACGAAGGACATGTCATGTGTACATCGTCGGTTAAAAAGTCAGTTTCTCAGGAAATGGTGA
GCGGGGCCGCAATGATCAATCCTGTATCTAAGCCATTGAATGGGAAAGTTTTGACTTTCACTCAGTCTGATAA
AGAGGCGCTGCTTTCTCGAGGATATACGGACGTCCATACAGTACATGAGGTACAAGGTGAGACATATGCAGA
TGTGTCGTTGGTCAGATTGACTCCGACACCTGTATCTATCATCGCAGGAGATAGTCCGCACGTTCTCGTAGCTT
TGTCAAGGCATACCCAAACATTGAAGTATTACACCGTAGTGATGGATCCTCTTGTAAGTATAATTAGGGATTTA
GAAAAACTTAGTTCTTACTTGTTAGATATGTATAAAGTAGATGCAGGGACCCAATAGCAATTACAGGTAGACT
CCGTGTTTAAAGGTTCTAATCTTTTTGTTGCAGCACCAAAGACTGGAGATATCTCAGATATGCAATTTTACTAT
GATAAGTGTCTCCCAGGTAATAGCACCATGTTAAATAACTATGATGCTGTTACCATGAGGTTGACTGACATTTC
TCTTAATGTCAAAGATTGCATATTGGATTTCTCTAAGTCTGTGGCTGCACCGAAGGATCCGATCAAACCACTGA
TTCCAATGGTACGAACGGCGGCAGAAATGCCACGCCAGACTGGACTATTGGAAAATTTGGTGGCGATGATCA
AAAGAAACTTTAATTCACCGGAGTTATCAGGAATAATCGACATTGAGAATACTGCATCTTTAGTAGTAGATAA
ATTTTTTGATAGTTACTTGCTTAAAGAAAAAAGAAAACCAAATAAAAATGTTTCTTTATTTTGTAGAGAGTCTCT
CAATAGATGGTTAGAGAAGCAGGAGCAAGTGACCATTGGTCAGCTTGCAGATTTTGATTTTGTGGATCTTCCT
GCCGTTGATCAGTACAGGCATATGATTAAAGCGCAACCTAAGCAGAAGCTGGATACATCAATTCAAAGCGAAT
ATCCGGCCTTGCAGACGATTGTGTATCATTCGAAAAAGATCAACGCAATCTTCGGTCCTTTGTTCAGTGAGCTC
ACAAGGCAAATGCTCGAAAGCATAGACTCAAGTAAGTTTTTGTTCTTTACAAGGAAGACGCCAGCTCAAATTG
AGGATTTCTTCGGAGATCTCGATAGCCATGTCCCTATGGATATCTTGGAGTTGGATATTTCGAAGTATGACAAA
TCTCAGAACGAGTTCCACTGTGCAGTAGAGTATGAAATATGGAGAAGACTTGGATTAGAAGATTTTCTGGGA
GAAGTTTGGAAACAAGGCCACAGGAAAACTACTCTTAAAGATTACACAGCTGGTATTAAAACGTGTTTATGGT
ACCAGAGAAAGAGTGGGGACGTTACAACATTCATCGGTAATACGGTGATTATTGCTGCTTGTTTAGCTTCCAT
GTTGCCCATGGAGAAAATAATCAAAGGTGCATTTTGCGGAGATGACAGTTTACTATACTTCCCAAAAGGTTGT
GAGTTTCCTGACATACAGCATACAGCCAACCTTATGTGGAATTTCGAGGCTAAGCTATTCAGAAAGCAGTATG
GTTATTTCTGTGGAAGGTACGTGATACATCATGACAGAGGGTGTATTGTTTATTATGACCCTTTGAAGTTGATT
TCTAAACTTGGTGCTAAACACATCAAGGATTGGGATCACTTAGAAGAGTTCAGAAGATCCCTTTGTGATGTTG
CAAATTCGTTGAACAACTGTGCGTATTACACGCAGTTGGACGACGCTGTGAGTGAGGTCCATAAAACCGCACC
CCCGGGTTCGTTTGTATATAAAAGTTTAGTTAAATATCTGTCCGATAAGGTTCTTTTTAGAAGTTTGTTTATAGA
TGGCTCTTGTTAAGGGTAAAGTCAATATTAATGAGTTCATAGACTTGTCAAAATCAGAAAAAATTTCTTCCGTCT
ATGTTCACACCTGTTAAGAGTGTCATGATCTCCAAGGTTGATAAGATATTGGTTCATGAAGATGAATCTTTGTC
CGAAGTCAATTTACTCAAAGGTGTAAAACTCATTGATGGTGGCTATGTACATCTTGCTGGTCTTGTGGTGACA

GGTGAATGGAATTTGCCAGATAATTGTCGTGGTGGTGTCAGTGTCTGTTTGGTCGATAAAAGAATGGAGAGA
GCGGACGAGGCAACTCTTGCTTCATACTATACCGCAGCGGCTAAGAAAAGGTTTCAGTTCAAAGTCGTTCCAA
ATTACAACATCACTACCAAGGACGCAGAAAAGGCAGTTTGGCAAGTACTAGTTAATATTAGAAATGTTAAAAT
TGCTGCGGGTTACTGTCCGCTGTCATTAGAATTTGTGTCAGTGTGTATTGTTTATAAAAATATTATAAAACTCG
GTTTGAGAGAGAAAATTACGAGCGTCACGGATGGAGGGCCCATGGAACTATCAGAAGAAGTTGTTGATGAG
TTCATGGAAGAAGTCCCGATGTCTGTAAGGCTTGCAAAATTTCGTTCGAAGACCGGGAAAAAGTTTAGTAGTA
AAAGTGAGAATAATAGTGGTAATAATAGGCCGAAACCAAACAAAAACCAAAGGAAGGAAAAGGGTTTAAAA
GTTAGGGTTGAGAAGGATAATTTAATTGATAATGAATTGGAGACTTACGTCGCCGATTCAGATTCGTATTAAA
TATGTCTTACACAATCGCAACTCCATCGCAATTTGTGTTTTTGTCATCAGCATGGGCCGACCCTATAGAATTAAT
AAATTTATGTACTAATTCACTAGGTAATCAATTCCAAACACAACAAGCTAGAACAACCGTTCAACGGCAATTTA
GCGAAGTGTGGAAACCTGTCCCTCAAGTCACTGTTAGGTTTCCTGACAGTGGTTTTAAGGTGTATAGGTACAA
TGCGGTACTAGATCCTCTAGTTACTGCTTTGTTAGGAGCTTTCGATACTAGAAATAGGATTATAGAAGTCGAAA
ATCAGGCGAACCCGACAACCGCCGAAACGTTAGACGCTACTCGTAGAGTAGATGACGCAACGGTGGCTATAA
GGAGCGCTATAAATAATTTAGTAGTAGAATTGGTCAAAGGAACAGGTTTGTACAATCAGAGCACATTTGAAA
GTGCATCCGGTTTACAATGGTCCTCTGCACCTGCATCTTGA

MN882058

ATGGCATACACACAGACAGCTACCACATCCGCTTTGCTCGACACTGTCCGAGGTAACAATACCTTGGTCAACG
ATCTTGCGAAGCGGCGTCTTTATGACACAGCGGTCGACGAGTTCAACGCTCGTGATCGCAGGCCCAAAGTAA
ATTTTTCCAAAGTAATAAGTGAGGAACAGACGCTTATTGCTACTAGGGCATATCCTGAATTCCAGATAACCTTC
TATAATACGCAGAACGCCGTGCATTCGCTTGCCGGTGGACTACGATCCTTAGAACTGGAATATCTAATGATGC
AGATCCCGTACGGATCACTCACATATGATATAGGTGGGAATTTTGCATCTCATCTGTTCAAAGGACGGGCATA
TGTTCACTGCTGTATGCCCAATCTTGATGTCCGCGACATAATGCGGCACGAAGGCCAGAAAGACAGTATAGAA
TTATACCTTTCCAGGCTTGAGCGGGGCAACAAAGTTGTCCCAAATTTCCAAAAGGAAGCTTTTGACAGATACG
CTGAAACGCCAGACGAAGTTGTCTGTCACAGTACCTTCCAAACGTGTACGCACCAGCAGGTGGAAAACACAG
GCAGGGTGTATGCTATTGCATTGCACAGTATATACGATATACCTGCTGATGAATTCGGAGCGGCACTTTTGAG
GAAAAATGTCCATGTTTGTTACGCCGCCTTCCACTTTTCCGAGAATTTACTTCTCGAAGATTCACACGTCAACCT
TGACGAAATCAACGCGTGTTTTTCGCGTGATGGAGACAAGCTGACTTTTTCTTTCGCATCTGAGAGCACTTTAA
ATTATTGTCATAGTTATTCTAATATTTTAAAATACGTGTGCAAAACTTACTTCCCGGCATCTAATAGAGAGGTCT
ACATGAAGGAGTTTTTGGTCACCAGGGTTAACACCTGGTTTTGTAAGTTTTCTAGGATAGATACTTTTTTATTAT
ACAAGGGGGTAGCCCACAAAGGTGTAAATAGTGAGCAATTTTACAGCGCAATGGAAGATGCATGGCACTACA
AAAAGACTCTTGCAATGTGTAACAGCGAGAGGATTCTTCTTGAAGATTCCTCATCGGTCAATTACTGGTTCCCA
AAAATGAGAGATATGGTCATAGTTCCTCTATTCGACATATCTCTCGACACTAGTAAAAGGACCCGCAAAGAAG
TCTTAGTGTCAAAGGATTTTGTATTCACAGTTTTAAATCACATTCGCACTTATCAAGCCAAGGCACTTACATACT
CCAATGTTTTATCCTTTGTCGAATCAATTCGTTCAAGGGTAATTATCAACGGAGTGACTGCCAGGTCTGAGTGG
GATGTTGACAAATCTCTTTTGCAATCCTTGTCCATGACATTTTTCTTGCATACTAAGCTTGCCGTTTTAAAAGAC
GAATTGTTAATCAGCAAGTTTAGTTTGGGGCCAAAATCAGTAAGCCAGCATGTATGGGATGAGATTTCCCTGG
CTTTTGGAAACGCATTTCCATCGATCAAGGAGAGACTGCTAAATCGGAAACTAATTAAAGTGTCGGGAGACGC
ATTAGAAATCAGGGTGCCTGATTTATATGTGACTTTTCACGATAGATTAGTGACTGAGTACAAAACATCGGTG
GATATGCCAGTGCTTGATATCAGAAAGAGAATGGAGGAGACTGAGGTTATGTACAATGCATTGTCTGAGCTA
TCTGTGCTCAAGGAGTCGGACAAGTTCGACGTTGATGTTTTTTCCCGGATGTGCCAGACTTTGGAGGTAGACC
CAATGACTGCAGCAAAGGTTATTGTGGCAGTGATGAGCAACGAGAGCGGACTGACTCTTACATTCGAACAGC
CAACTGAAGCAAATGTCGCATTGGCACTTAAAGATTCAGAAAAAGCCTCTGAGGGTGCACTAGTGGTTACTTC
TAGAGATGTTGAAGAACCATCCATGAAGGGTTCAATGGCAAGAGGAGAGTTACAATTGGCCGGTCTGTCTGG
AGACCAACCAGAGTCTTCCTATACTCGGAACGAGGAAATAGAGTCATTAGAGCAATTCCACATGGCAACGGCT
GGTTCGTTAATTCGGAAACAGATGAGTTCGATTGTGTACACGGGCCCCATTAAAGTTCAGCAAATGAAAAACT

TTATTGATAGCCTGGTAGCATCACTCTCTGCTGCGGTGTCGAACCTAGTCAAGATCCTAAAGGATACAGCTGCT
ATAGATCTCGAAACCCGTCAGAAGTTTGGAGTCTTAGATGTTGCGACCAAAAGATGGTTAATTAAACCTTTAG
CCAAGAATCACGCATGGGGCGTTATTGAAACACATGCTAGGAAGTACCACGTTGCACTTTTGGAGTATGATGA
GCATGGAGTGGTAACTTGCGACAGTTGGAGAAGGGTGGCCGTGAGTTCTGAGTCAATGGTTTATTCTGATAT
GGCAAAGCTCAGAACACTGAGGAGATTATTAAGAGATGGTGAGCCTCATGTCAGCAGTGCTAAAGTCGTCCT
AGTTGACGGTGTCCCGGGTTGTGGAAAGACAAAAGAGATTCTCTCGAAAGTAAATTTTGAGGAAGATCTAAT
CTTAGTACCGGGTAAGCAGGCTGCTGAAATGATAAAGAGGCGTGCTAATGCGTCAGGAATAATTCAAGCCAC
AAGAGATAATGTTCGTACTGTTGATTCATTTATAATGAATTACGGTAAAGGAACACGCTGTCAGTTCAAAAGG
TTATTTATCGACGAAGGTCTGATGTTGCACACTGGTTGTGTGAATTTTCTTGTTTCTATGTCTCTGTGCGAAATT
GCATATGTTTATGGAGACACACAACAAATTCCATACATCAACAGAGTATCCGGTTTTCCGTACCCTGCACATTT
TGCCAAAATAGAGGTTGATGAGGTGGAAACTCGCAGAACTACGCTGCGTTGTCCAGCCGACATTACCCACTAT
CTTAACAGAAGGTACGAAGGACATGTCATGTGTACATCGTCGGTTAAAAAGTCAGTTTCTCAGGAAATGGTGA
GCGGGGCCGCAATGATCAATCCTGTATCTAAGCCATTGAATGGGAAAGTTTTGACTTTCACTCAGTCTGATAA
AGAGGCGCTGCTTTCTCGAGGATATACGGACGTCCATACAGTACATGAGGTACAAGGTGAGACATATGCAGA
TGTGTCGTTGGTCAGATTGACTCCGACACCTGTATCTATCATCGCAGGAGATAGTCCGCACGTTCTCGTAGCTT
TGTCAAGGCATACCCAAACATTGAAGTATTACACCGTAGTGATGGATCCTCTTGTAAGTATAATTAGGGATTTA
GAAAAACTTAGTTCTTACTTGTTAGATATGTATAAAGTAGATGCAGGGACCCAATAGCAATTACAGGTAGACT
CCGTGTTTAAAGGTTCTAATCTTTTTGTTGCAGCACCAAAGACTGGAGATATCTCAGATATGCAATTTTACTAT
GATAAGTGTCTCCCAGGTAATAGCACCATGTTAAATAACTATGATGCTGTTACCATGAGGTTGACTGACATTTC
TCTTAATGTCAAAGATTGCATATTGGATTTCTCTAAGTCTGTGGCTGCACCGAAGGATCCGATCAAACCACTGA
TTCCAATGGTACGAACGGCGGCAGAAATGCCACGCCAGACTGGACTATTGGAAAATTTGGTGGCGATGATCA
AAAGAAACTTTAATTCACCGGAGTTATCAGGAATAATCGACATTGAGAATACTGCATCTTTAGTAGTAGATAA
ATTTTTTGATAGTTACTTGCTTAAAGAAAAAAGAAAACCAAATAAAAATGTTTCTTTATTTTGTAGAGAGTCTCT
CAATAGATGGTTAGAGAAGCAGGAGCAAGTGACCATTGGTCAGCTTGCAGATTTTGATTTTGTGGATCTTCCT
GCCGTTGATCAGTACAGGCATATGATTAAAGCGCAACCTAAGCAGAAGCTGGATACATCAATTCAAAGCGAAT
ATCCGGCCTTGCAGACGATTGTGTATCATTCGAAAAAGATCAACGCAATCTTCGGTCCTTTGTTCAGTGAGCTC
ACAAGGCAAATGCTCGAAAGCATAGACTCAAGTAAGTTTTTGTTCTTTACAAGGAAGACGCCAGCTCAAATTG
AGGATTTCTTCGGAGATCTCGATAGCCATGTCCCTATGGATATCTTGGAGTTGGATATTTCGAAGTATGACAAA
TCTCAGAACGAGTTCCACTGTGCAGTAGAGTATGAAATATGGAGAAGACTTGGATTAGAAGATTTTCTGGGA
GAAGTTTGGAAACAAGGCCACAGGAAAACTACTCTTAAAGATTACACAGCTGGTATTAAAACGTGTTTATGGT
ACCAGAGAAAGAGTGGGGACGTTACAACATTCATCGGTAATACGGTGATTATTGCTGCTTGTTTAGCTTCCAT
GTTGCCCATGGAGAAAATAATCAAAGGTGCATTTTGCGGAGATGACAGTTTACTATACTTCCCAAAAGGTTGT
GAGTTTCCTGACATACAGCATACAGCCAACCTTATGTGGAATTTCGAGGCTAAGCTATTCAGAAAGCAGTATG
GTTATTTCTGTGGAAGGTACGTGATACATCATGACAGAGGGTGTATTGTTTATTATGACCCTTTGAAGTTGATT
TCTAAACTTGGTGCTAAACACATCAAGGATTGGGATCACTTAGAAGAGTTCAGAAGATCCCTTTGTGATGTTG
CAAATTCGTTGAACAACTGTGCGTATTACACGCAGTTGGACGACGCTGTGAGTGAGGTCCATAAAACCGCACC
CCCGGGTTCGTTTGTATATAAAAGTTTAGTTAAATATCTGTCCGATAAGGTTCTTTTTAGAAGTTTGTTTATAGA
TGGCTCTTGTTAAGGGTAAAGTCAATATTAATGAGTTCATAGACTTGTCAAAATCAGAAAAATTTCTTCCGTCT
ATGTTCACACCTGTTAAGAGTGTCATGATCTCCAAGGTTGATAAGATATTGGTTCATGAAGATGAATCTTTGTC
CGAAGTCAATTTACTCAAAGGTGTAAAACTCATTGATGGTGGCTATGTACATCTTGCTGGTCTTGTGGTGACA
GGTGAATGGAATTTGCCAGATAATTGTCGTGGTGGTGTCAGTGTCTGTTTGGTCGATAAAAGAATGGAGAGA
GCGGACGAGGCAACTCTTGCTTCATACTATACCGCAGCGGCTAAGAAAAGGTTTCAGTTCAAAGTCGTTCCAA
ATTACAACATCACTACCAAGGACGCAAAAAAGGCAGTTTGGCAAGTACTAGTTAATATTAGAAATGTTAAAAT
TGCTGCGGGTTACTGTCCGCTGTCATTAGAATTTGTGTCAGTGTGTATTGTTTATAAAAATATTATAAAACTCG
GTTTGAGAGAGAAAATTACGAGCGTAACGGATGGAGGGCCCATGGAACTATCAGAAGAAGTTGTTGATGAG
TTCATGGAAGAAGTCCCGATGTCTGTAAGGCTTGCAAAATTTCGTTCGAAGACCGGGAAAAAGTTTAGTAGTA
AAAGTGAGAATAATAGTGGTAATAATAGGCCGAAACCAAACAAAAACCAAAGGAAGGAAAAGGGTTTAAAA
GTTAGGGTTGAGAAGGATAATTTAATTGATAATGAATTGGAGACTTACGTCGCCGATTCAGATTCGTATTAAA

TATGTCTTACACAATCGCAACTCCATCGCAATTTGTGTTTTTGTCATCAGCATGGGCCGACCCTATAGAATTAAT
AAATTTATGTACTAATTCACTAGGTAATCAATTCCAAACACAACAAGCTAGAACAACCGTTCAACGGCAATTTA
GCGAAGTGTGGAAACCTGTCCCTCAAGTCACTGTTAGGTTTCCTGACAGTGGTTTTAAGGTGTATAGGTACAA
TGCGGTACTAGATCCTCTAGTTACTGCTTTGTTAGGAGCTTTCGATACTAGAAATAGGATTATAGAAGTCGAAA
ATCAGGCGAACCCGACAACCGCCGAAACGTTAGACGCTACTCGTAGAGTAGATGACGCAACGGTGGCTATAA
GGAGCGCTATAAATAATTTAGTAGTAGAATTGGTCAAAGGAACAGGTTTGTACAATCAGAGCACATTTGAAA
GTGCATCCGGTTTACAATGGTCCTCTGCACCTGCATCTTGA

MN882059

ATGGCATACACACAGACAGCTACCACATCCGCTTTGCTCGACACTGTCCGAGGTAACAATACCTTGGTCAACG
ATCTTGCGAAGCGGCGTCTTTATGACACAGCGGTCGACGAGTTCAACGCTCGTGATCGCAGGCCCAAAGTAA
ATTTTTCCAAAGTAATAAGTGAGGAACAGACGCTTATTGCTACTAGGGCATATCCTGAATTCCAGATAACCTTC
TATAATACGCAGAACGCCGTGCATTCGCTTGCCGGTGGACTACGATCCTTAGAACTGGAATATCTAATGATGC
AGATCCCGTACGGATCACTCACATATGATATAGGTGGGAATTTTGCATCTCATCTGTTCAAAGGACGGGCATA
TGTTCACTGCTGTATGCCCAATCTTGATGTCCGCGACATAATGCGGCACGAAGGCCAGAAAGACAGTATAGAA
TTATACCTTTCCAGGCTTGAGCGGGGCAACAAAGTTGTCCCAAATTTCCAAAAGGAAGCTTTTGACAGATACG
CTGAAACGCCAGACGAAGTTGTCTGTCACAGTACCTTCCAAACGTGTACGCACCAGCAGGTGGAAAACACAG
GCAGGGTGTATGCTATTGCATTGCACAGTATATACGATATACCTGCTGATGAATTCGGAGCGGCACTTTTAAG
GAAAAATGTCCATGTTTGTTACGCCGCCTTCCACTTTTCCGAGAATTTACTTCTCGAAGATTCACACGTCAACCT
TGACGAAATCAACGCGTGTTTTTCGCGTGATGGAGACAAGCTGACTTTTTCTTTCGCATCTGAGAGCACTTTAA
ATTATTGTCATAGTTATTCTAATATTTTAAAATACGTGTGCAAAACTTACTTCCCGGCATCTAATAGAGAGGTCT
ACATGAAGGAGTTTTTGGTCACCAGGGTTAACACCTGGTTTTGTAAGTTTTCTAGGATAGATACTTTTTTATTAT
ACAAGGGGGTAGCCCACAAAGGTGTAAATAGTGAGCAATTTTACAGCGCAATGGAAGATGCATGGCACTACA
AAAAGACTCTTGCAATGTGTAACAGCGAGAGGATTCTTCTTGAAGATTCCTCATCGGTCAATTACTGGTTCCCA
AAAATGAGAGATATGGTCATAGTTCCTCTATTCGACATATCTCTCGACACTAGTAAAAGGACCCGCAAAGAAG
TCTTAGTGTCAAAGGATTTTGTATTCACAGTTTTAAATCACATTCGCACTTATCAAGCCAAGGCACTTACATACT
CCAATGTTTTATCCTTTGTCGAATCAATTCGTTCAAGGGTAATTATCAACGGAGTGACTGCCAGGTCTGAGTGG
GATGTTGACAAATCTCTTTTGCAATCCTTGTCCATGACATTTTTCTTGCATACTAAGCTTGCCGTTTTAAAAGAC
GAATTGTTAATCAGCAAGTTTAGTTTGGGGCCAAAATCAGTAAGCCAGCATGTATGGGATGAGATTTCCCTGG
CTTTTGGAAACGCATTTCCATCGATCAAGGAGAGACTGCTAAATCGGAAACTAATTAAAGTGTCGGGAGACGC
ATTAGAAATCAGGGTGCCTGATTTATATGTGACTTTTCACGATAGATTAGTGACTGAGTACAAAACATCGGTG
GATATGCCAGTGCTTGATATCAGAAAGAGAATGGAGGAGACTGAGGTTATGTACAATGCATTGTCTGAGCTA
TCTGTGCTCAAGGAGTCGGACAAGTTCGACGTTGATGTTTTTTCCCGGATGTGCCAGACTTTGGAGGTAGACC
CAATGACTGCAGCAAAGGTTATTGTGGCAGTGATGAGCAACGAGAGCGGACTGACTCTTACATTCGAACAGC
CAACTGAAGCAAATGTCGCATTGGCACTTAAAGATTCAGAAAAAGCCTCTGAGGGTGCACTAGTGGTTACTTC
TAGAGATGTTGAAGAACCATCCATGAAGGGTTCAATGGCAAGAGGAGAGTTACAATTGGCCGGTCTGTCTGG
AGACCAACCAGAGTCTTCCTATACTCGGAACGAGGAAATAGAGTCATTAGAGCAATTCCACATGGCAACGGCT
GGTTCGTTAATTCGGAAACAGATGAGTTCGATTGTGTACACGGGCCCCATTAAAGTTCAGCAAATGAAAAACT
TTATTGATAGCCTGGTAGCATCACTCTCTGCTGCGGTGTCGAACCTAGTCAAGATCCTAAAGGATACAGCTGCT
ATAGATCTCGAAACCCGTCAGAAGTTTGGAGTCTTAGATGTTGCGACCAAAAGATGGTTAATTAAACCTTTAG
CCAAGAATCACGCATGGGGCGTTATTGAAACACATGCTAGGAAGTACCACGTTGCACTTTTGGAGTATGATGA
GCATGGAGTGGTAACTAGCGACAGTTGGAGAAGGGTGGCCGTGAGTTCTGAGTCAATGGTTTATTCTGATAT
GGCAAAGCTCAGAACACTGAGGAGATTATTAAGAGATGGTGAGCCTCATGTCAGCAGTGCTAAAGTCGTCCT
AGTTGACGGTGTCCCGGGTTGTGGAAAGACAAAAGAGATTCTCTCGAAAGTAAATTTTGAGGAAGATCTAAT
CTTAGTACCGGGTAAGCAGGCTGCTGAAATGATAAAGAGGCGTGCTAATGCGTCAGGAATAATTCAAGCCAC
AAGAGATAATGTTCGTACTGTTGATTCATTTATAATGAATTACGGTAAAGGAACACGCTGTCAGTTCAAAAGG

TTATTTATCGACGAAGGTCTGATGTTGCACACTGGTTGTGTGAATTTTCTTGTTTCTATGTCTCTGTGCGAAATT
GCATATGTTTATGGAGACACACAACAAATTCCATACATCAACAGAGTATCCGGTTTTCCGTACCCTGCACATTT
TGCAAAAATAGAGGTTGATGAGGTGGAAACTCGCAGAACTACGCTGCGTTGTCCAGCCGACATTACCCACTAT
CTTAACAGAAGGTACGAAGGACATGTCATGTGTACATCGTCGGTTAAAAAGTCAGTTTCTCAGGAAATGGTGA
GCGGGGCCGCAATGATCAATCCTGTATCTAAGCCATTGAATGGGAAAGTTTTGACTTTCACTCAGTCTGATAA
AGAGGCGCTGCTTTCTCGAGGATATACGGACGTCCATACAGTACATGAGGTACAAGGTGAGACATATGCAGA
TGTGTCGTTGGTCAGATTGACTCCGACACCTGTATCTATCATCGCAGGAGATAGTCCGCACGTTCTCGTAGCTT
TGTCAAGGCATACCCAAACATTGAAGTATTACACCGTAGTGATGGATCCTCTTGTAAGTATAATTAGGGATTTA
GAAAAACTTAGTTCTTACTTGTTAGATATGTATAAAGTAGATGCAGGGACCCAATAGCAATTACAGGTAGACT
CCGTGTTTAAAGGTTCTAATCTTTTTGTTGCAGCACCAAAGACTGGAGATATCTCAGATATGCAATTTTACTAT
GATAAGTGTCTCCCAGGTAATAGCACCATGTTAAATAACTATGATGCTGTTACCATGAGGTTGACTGACATTTC
TCTTAATGTCAAAGATTGCATATTGGATTTCTCTAAGTCTGTGGCTGCACCGAAGGATCCGATCAAACCACTGA
TTCCAATGGTACGAACGGCGGCAGAAATGCCACGCCAGACTGGACTATTGGAAAATTTGGTGGCGATGATCA
AAAGAAACTTTAATTCACCGGAGTTATCAGGAATAATCGACATTGAGAATACTGCATCTTTAGTAGTAGATAA
ATTTTTTGATAGTTACTTGCTTAAAGAAAAAAGAAAACCAAATAAAAATGTTTCTTTATTTTGTAGAGAGTCTCT
CAATAGATGGTTAGAGAAGCAGGAGCAAGTGACCATTGGTCAGCTTGCAGATTTTGATTTTGTGGATCTTCCT
GCCGTTGATCAGTACAGGCATATGATTAAAGCGCAACCTAAGCAGAAGCTGGATACATCAATTCAAAGCGAAT
ATCCGGCCTTGCAGACGATTGTGTATCATTCGAAAAAGATCAACGCAATCTTCGGTCCTTTGTTCAGTGAGCTC
ACAAGGCAAATGCTCGAAAGCATAGACTCAAGTAAGTTTTTGTTCTTTACAAGGAAGACGCCAGCTCAAATTG
AGGATTTCTTCGGAGATCTCGATAGCCATGTCCCTATGGATATCTTGGAGTTGGATATTTCGAAGTATGACAAA
TCTCAGAACGAGTTCCACTGTGCAGTAGAGTATGAAATATGGAGAAGACTTGGATTAGAAGATTTTCTGGGA
GAAGTTTGGAAACAAGGCCACAGGAAAACTACTCTTAAAGATTACACAGCTGGTATTAAAACGTGTTTATGGT
ACCAGAGAAAGAGTGGGGACGTTACAACATTCATCGGGAATACGGTGATTATTGCTGCTTGTTTAGCTTCCAT
GTTACCCATGGAGAAAATAATCAAAGGTGCATTTTGCGGAGATGACAGTTTACTATACTTCCCAAAAGGTTGT
GAGTTTCCTGACATACAGCATACAGCCAACCTTATGTGGAATTTCGAGGCTAAGCTATTCAGAAAGCAGTATG
GTTATTTCTGTGGAAGGTACGTGATACATCATGACAGAGGGTGTATTGTTTATTATGACCCTTTGAAGTTGATT
TCTAAACTTGGTGCTAAACACATCAAGGATTGGGATCACTTAGAAGAGTTCAGAAGATCCCTTTGTGATGTTG
CAAATTCGTTGAACAACTGTGCGTATTACACGCAGTTGGACGACGCTGTGAGTGAGGTCCATAAAACCGCACC
CCCGGGTTCGTTTGTATATAAAAGTTTAGTTAAATATCTGTCCGATAAGGTTCTTTTTAGAAGTTTGTTTATAGA
TGGCTCTTGTTAAGGGTAAAGTCAATATTAATGAGTTCATAGACTTGTCAAAATCAGAAAAATTTCTTCCGTCT
ATGTTCACACCTGTTAAGAGTGTCATGATCTCCAAGGTTGATAAGATATTGGTTCATGAAGATGAATCTTTGTC
CGAAGTCAATTTACTCAAAGGTGTAAAACTCATTGATGGTGGCTATGTACATCTTGCTGGTCTTGTGGTGACA
GGTGAATGGAATTTGCCAGATAATTGTCGTGGTGGTGTCAGTGTCTGTTTGGTCGATAAAAGAATGGAGAGA
GCGGACGAGGCAACTCTTGCTTCATACTATACCGCAGCGGCTAAGAAAAGGTTTCAGTTCAAAGTCGTTCCAA
ATTACAACATCACTACCAAGGACGCAGAAAAGGCAGTTTGGCAAGTACTAGTTAATATTAGAAATGTTAAAAT
TGCTGCGGGTTACTGTCCGCTGTCATTAGAATTTGTGTCAGTGTGTATTGTTTATAAAAATATTATAAAACTCG
GTTTGAGAGAGAAAATTACGAGCGTCACGGATGGAGGGCCCATGGAACTATCAGAAGAAGTTGTTGATGAG
TTCATGGAAGAAGTCCCGATGTCTGTAAGGCTTGCAAAATTTCGTTCGAAGACCGGGAAAAAGTTTAGTAGTA
AAAGTGAGAATAATAGTGGTAATAATAGGCCGAAACCAAACAAAAACCAAAGGAAGGAAAAGGGTTTAAAA
GTTAGGGTTGAGAAGGATAATTTAATTGATAATGAATTGGAGACTTACGTCGCCGATTCAGATTCGTATTAAA
TATGTCTTACACAATCGCAACTCCATCGCAATTTGTGTTTTTGTCATCAGCATGGGCCGACCCTATAGAATTAAT
AAATTTATGTACTAATTCACTAGGTAATCAATTCCAAACACAACAAGCTAGAACAACCGTTCAACGGCAATTTA
GCGAAGTGTGGAAACCTGTCCCTCAAGTCACTGTTAGGTTTCCTGACAGTGGTTTTAAGGTGTATAGGTACAA
TGCGGTACTAGATCCTCTAGTTACTGCTTTGTTAGGAGCTTTCGATACTAGAAATAGGATTATAGAAGTCGAAA
ATCAGGCGAACCCGACAACCGCCGAAACGTTAGACGCTACTCGTAGAGTAGATGACGCAACGGTGGCTATAA
GGAGCGCTATAAATAATTTAGTAGTAGAATTGGTCAAAGGAACAGGTTTGTACAATCAGAGCACATTTGAAA
GTGCATCCGGTTTACAATGGTCCTCTGCACCTGCATCTTGA

MN882062

ATGGCATACACACAGACAGCTACCACATCCGCTTTGCTCGACACTGTCCGAGGTAACAATACCTTGGTCAACG
ATCTTGCGAAGCGGCGTCTTTATGACACAGCGGTCGACGAGTTCAACGCTCGTGATCGCAGGCCCAAAGTAA
ATTTTTCCAAAGTAATAAGTGAGGAACAGACGCTTATTGCTACTAGGGCATATCCTGAATTCCAGATAACCTTC
TATAATACGCAGAACGCCGTGCATTCGCTTGCCGGTGGACTACGATCCTTAGAACTGGAATATCTAATGATGC
AGATCCCGTACGGATCACTCACATATGATATAGGTGGGAATTTTGCATCTCATCTGTTCAAAGGACGGGCATA
TGTTCACTGCTGTATGCCCAATCTTGATGTCCGCGACATAATGCGGCACGAAGGCCAGAAAGACAGTATAGAA
TTATACCTTTCCAGGCTTGAGCGGGGCAACAAAGTTGTCCCAAATTTCCAAAAGGAAGCTTTTGACAGATACG
CTGAAACGCCAGACGAAGTTGTCTGTCACAGTACCTTCCAAACGTGTACGCACCAGCAGGTGGAAAACACAG
GCAGGGTGTATGCTATTGCATTGCACAGTATATACGATATACCTGCTGATGAATTCGGAGCGGCACTTTTAAG
GAAAAATGTCCATGTTTGTTACGCCGCCTTCCACTTTTCCGAGAATTTACTTCTCGAAGATTCACACGTCAACCT
TGACGAAATCAACGCGTGTTTTTCGCGTGATGGAGACAAGCTGACTTTTTCTTTCGCATCTGAGAGCACTTTAA
ATTATTGTCATAGTTATTCTAATATTTTAAAATACGTGTGCAAAACTTACTTCCCGGCATCTAATAGAGAGGTCT
ACATGAAGGAGTTTTTGGTCACCAGGGTTAACACCTGGTTTTGTAAGTTTTCTAGGATAGATACTTTTTTATTAT
ACAAGGGGGTAGCCCACAAAGGTGTAAATAGTGAGCAATTTTACAGCGCAATGGAAGATGCATGGCACTACA
AAAAGACTCTTGCAATGTGTAACAGCGAGAGGATTCTTCTTGAAGATTCCTCATCGGTCAATTACTGGTTCCCA
AAAATGAGAGATATGGTCATAGTTCCTCTATTCGACATATCTCTCGACACTAGTAAAAGGACCCGCAAAGAAG
TCTTAGTGTCAAAGGATTTTGTATTCACAGTTTTAAATCACATTCGCACTTATCAAGCCAAGGCACTTACATACT
CCAATGTTTTATCCTTTGTCGAATCAATTCGTTCAAGGGTAATTATCAACGGAGTGACTGCCAGGTCTGAGTGG
GATGTTGACAAATCTCTTTTGCAATCCTTGTCCATGACATTTTTCTTGCATACTAAGCTTGCCGTTTTAAAAGAC
GAATTGTTAATCAGCAAGTTTAGTTTGGGGCCAAAATCAGTAAGCCAGCATGTATGGGATGAGATTTCCCTGG
CTTTTGGAAACGCATTTCCATCGATCAAGGAGAGACTGCTAAATCGGAAACTAATTAAAGTGTCGGGAGACGC
ATTAGAAATCAGGGTGCCTGATTTATATGTGACTTTTCACGATAGATTAGTGACTGAGTACAAAACATCGGTG
GATATGCCAGTGCTTGATATCAGAAAGAGAATGGAGGAGACTGAGGTTATGTACAATGCATTGTCTGAGCTA
TCTGTGCTCAAGGAGTCGGACAAGTTCGACGTTGATGTTTTTTCCCGGATGTGCCAGACTTTGGAGGTAGACC
CAATGACTGCAGCAAAGGTTATTGTGGCAGTGATGAGCAACGAGAGCGGACTGACTCTTACATTCGAACAGC
CAACTGAAGCAAATGTCGCATTGGCACTTAAAGATTCAGAAAAAGCCTCTGAGGGTGCACTAGTGGTTACTTC
TAGAGATGTTGAAGAACCATCCATGAAGGGTTCAATGGCAAGAGGAGAGTTACAATTGGCCGGTCTGTCTGG
AGACCAACCAGAGTCTTCCTATACTCGGAACGAGGAAATAGAGTCATTAGAGCAATTCCACATGGCAACGGCT
GGTTCGTTAATTCGGAAACAGATGAGTTCGATTGTGTACACGGGCCCCATTAAAGTTCAGCAAATGAAAAACT
TTATTGATAGCCTGGTAGCATCACTCTCTGCTGCGGTGTCGAACCTAGTCAAGATCCTAAAGGATACAGCTGCT
ATAGATCTCGAAACCCGTCAGAAGTTTGGAGTCTTAGATGTTGCGACCAAAAGATGGTTAATTAAACCTTTAG
CCAAGAATCACGCATGGGGCGTTATTGAAACACATGCTAGGAAGTACCACGTTGCACTTTTGGAGTATGATGA
GCATGGAGTGGTAACTTGCGACAGTTGGAGAAGGGTGGCCGTGAGTTCTGAGTCAATGGTTTATTCTGATAT
GGCAAAGCTCAGAACACTGAGGAGATTATTAAGAGATGGTGAGCCTCATGTCAGCAGTGCTAAAGTCGTCCT
AGTTGACGGTGTCCCGGGTTGTGGAAAGACAAAAGAGATTCTCTCGAAAGTAAATTTTGAGGAAGATCTAAT
CTTAGTACCGGGTAAGCAGGCTGCTGAAATGATAAAGAGGCGTGCTAATGCGTCAGGAATAATTCAAGCCAC
AAGAGATAATGTTCGTACTGTTGATTCATTTATAATGAATTACGGTAAAGGAACACGCTGTCAGTTCAAAAGG
TTATTTATCGACGAAGGTCTGATGTTGCACACTGGTTGTGTGAATTTTCTTGTTTCTATGTCTCTGTGCGAAATT
GCATATGTTTATGGAGACACACAACAAATTCCATACATCAACAGAGTATCCGGTTTTCCGTACCCTGCACATTT
TGCAAAAATAGAGGTTGATGAGGTGGAAACTCGCAGAACTACGCTGCGTTGTCCAGCCGACATTACCCACTAT
CTTAACAGAAGGTACGAAGGACATGTCATGTGTACATCGTCGGTTAAAAAGTCAGTTTCTCAGGAAATGGTGA
GCGGGGCCGCAATGATCAATCCTGTATCTAAGCCATTGAATGGGAAAGTTTTGACTTTCACTCAGTCTGATAA
AGAGGCGCTGCTTTCTCGAGGATATACGGACGTCCATACAGTACATGAGGTACAAGGTGAGACATATGCAGA
TGTGTCGTTGGTCAGATTGACTCCGACACCTGTATCTATCATCGCAGGAGATAGTCCGCACGTTCTCGTAGCTT
TGTCAAGGCATACCCAAACATTGAAGTATTACACCGTAGTGATGGATCCTCTTGTAAGTATAATTAGGGATTTA

GAAAAACTTAGTTCTTACTTGTTAGATATGTATAAAGTAGATGCAGGGACCCAATAGCAATTACAGGTAGACT
CCGTGTTTAAAGGTTCTAATCTTTTTGTTGCAGCACCAAAGACTGGAGATATCTCAGATATGCAATTTTACTAT
GATAAGTGTCTCCCAGGTAATAGCACCATGTTAAATAACTATGATGCTGTTACCATGAGGTTGACTGACATTTC
TCTTAATGTCAAAGATTGCATATTGGATTTCTCTAAGTCTGTGGCTGCACCGAAGGATCCGATCAAACCACTGA
TTCCAATGGTACGAACGGCGGCAGAAATGCCACGCCAGACTGGACTATTGGAAAATTTGGTGGCGATGATCA
AAAGAAACTTTAATTCACCGGAGTTATCAGGAATAATCGACATTGAGAATACTGCATCTTTAGTAGTAGATAA
ATTTTTTGATAGTTACTTGCTTAAAGAAAAAAGAAAACCAAATAAAAATGTTTCTTTATTTTGTAGAGAGTCTCT
CAATAGATGGTTAGAGAAGCAGGAGCAAGTGACCATTGGTCAGCTTGCAGATTTTGATTTTGTGGATCTTCCT
GCCGTTGATCAGTACAGGCATATGATTAAAGCGCAACCTAAGCAGAAGCTGGATACATCAATTCAAAGCGAAT
ATCCAGCCTTGCAGACGATTGTGTATCATTCGAAAAAGATCAACGCAATCTTCGGTCCTTTGTTCAGTGAGCTC
ACAAGGCAAATGCTCGAAAGCATAGACTCAAGTAAGTTTTTGTTCTTTACAAGGAAGACGCCAGCTCAAATTG
AGGATTTCTTCGGAGATCTCGATAGCCATGTCCCTATGGATATCTTGGAGTTGGATATTTCGAAGTATGACAAA
TCTCAGAACGAGTTCCACTGTGCAGTAGAGTATGAAATATGGAGAAGACTTGGATTAGAAGATTTTCTGGGA
GAAGTTTGGAAACAAGGCCACAGGAAAACTACTCTTAAAGATTACACAGCTGGTATTAAAACGTGTTTATGGT
ACCAGAGAAAGAGTGGGGACGTTACAACATTCATCGGTAATACGGTGATTATTGCTGCTTGTTTAGCTTCCAT
GTTGCCCATGGAGAAAATAATCAAAGGTGCATTTTGCGGAGATGACAGTTTACTATACTTCCCAAAAGGTTGT
GAGTTTCCTGACATACAGCATACAGCCAACCTTATGTGGAATTTCGAGGCTAAGCTATTCAGAAAGCAGTATG
GTTATTTCTGTGGAAGGTACGTGATACATCATGACAGAGGGTGTATTGTTTATTATGACCCTTTGAAGTTGATT
TCTAAACTTGGTGCTAAACACATCAAGGATTGGGATCACTTAGAAGAGTTCAGAAGATCCCTTTGTGATGTTG
CAAATTCGTTGAACAACTGTGCGTATTACACGCAGTTGGACGACGCTGTGAGTGAGGTCCATAAAACCGCACC
CCCGGGTTCGTTTGTATATAAAAGTTTAGTTAAATATCTGTCCGATAAGGTTCTTTTTAGAAGTTTGTTTATAGA
TGGCTCTTGTTAAGGGTAAAGTCAATATTAATGAGTTCATAGACTTGTCAAAATCAGAAAAATTTCTTCCGTCT
ATGTTCACACCTGTTAAGAGTGTCATGATCTCCAAGGTTGATAAGATATTGGTTCATGAAGATGAATCTTTGTC
CGAAGTCAATTTACTCAAAGGTGTAAAACTCATTGATGGTGGCTATGTACATCTTGCTGGTCTTGTGGTGACA
GGTGAATGGAATTTGCCAGATAATTGTCGTGGTGGTGTCAGTGTCTGTTTGGTCGATAAAAGAATGGAGAGA
GCGGACGAGGCAACTCTTGCTTCATACTATACCGCAGCGGCTAAGAAAAGGTTTCAGTTCAAAGTCGTTCCAA
ATTACAACATCACTACCAAGGACGCAAAAAAGGCAGTTTGGCAAGTACTAGTTAATATTAGAAATGTTAAAAT
TGCTGCGGGTTACTGTCCGCTGTCATTAGAATTTGTGTCAGTGTGTATTGTTTATAAAAATATTATAAAACTCG
GTTTGAGAGAGAAAATTACGAGCGTAACGGATGGAGGGCCCATGGAACTATCAGAAGAAGTTGTTGATGAG
TTCATGGAAGAAGTCCCGATGTCTGTAAGGCTTGCAAAATTTCGTTCGAAGACCGGGAAAAAGTTTAGTAGTA
AAAGTGAGAATAATAGTGGTAATAATAGGCCGAAACCAAACAAAAACCAAAGGAAGGAAAAGGGTTTAAAA
GTTAGGGTTGAGAAGGATAATTTAATTGATAATGAATTGGAGACTTACGTCGCCGATTCAGATTCGTATTAAA
TATGTCTTACACAATCGCAACTCCATCGCAATTTGTGTTTTTGTCATCAGCATGGGCCGACCCTATAGAATTAAT
AAATTTATGTACTAATTCACTAGGTAATCAATTCCAAACACAACAAGCTAGAACAACCGTTCAACGGCAATTTA
GCGAAGTGTGGAAACCTGTCCCTCAAGTCACTGTTAGGTTTCCTGACAGTGGTTTTAAGGTGTATAGGTACAA
TGCGGTACTAGATCCTCTAGTTACTGCTTTGTTAGGAGCTTTCGATACTAGAAATAGGATTATAGAAGTCGAAA
ATCAGGCGAACCCGACAACCGCCGAAACGTTAGACGCTACTCGTAGAGTAGATGACGCAACGGTGGCTATAA
GGAGCGCTATAAATAATTTAGTAGTAGAATTGGTCAAAGGAACAGGTTTGTACAATCAGAGCACATTTGAAA
GTGCATCCGGTTTACAATGGTCCTCTGCACCTGCATCTTGA

#MT002973

1

ATGGCATACACACAGACAGCTACCACATCCGCTTTGCTCGACACTGTCCGAGGTAACAATACCTTGGTCAACG
ATCTTGCGAAGCGGCGTCTTTATGACACAGCGGTCGACGAGTTCAACGCTCGTGATCGCAGGCCCAAAGTAA

ATTTTTCCAAAGTAATAAGTGAGGAACAGACGCTTATTGCTACTAGGGCATATCCAGAATTCCAGATAACCTTC
TATAATACGCAGAACGCCGTGCATTCGCTTGCCGGTGGACTACGATCCTTAGAACTGGAATATCTAATGATGC
AGATCCCGTACGGATCACTCACATATGATATAGGTGGGAATTTTGCATCTCATCTGTTCAAAGGACGGGCATA
TGTTCACTGCTGTATGCCCAATCTTGATGTCCGCGACATAATGCGGCACGAAGGCCAGAAAGACAGTATAGAA
TTATACCTTTCCAGGCTTGAGCGGGGCAACAAAGTTGTCCCAAATTTCCAAAAGGAAGCTTTTGACAGATACG
CTGAAACGCCAGACGAAGTTGTCTGTCACAGTACCTTCCAAACGTGTACTCACCAGCAGGTGGAAAACACAG
GCAGGGTGTATGCTATTGCATTGCACAGTATATACGATATACCTGCTGATGAATTCGGAGCGGCACTTTTAAG
AAAAAATGTCCATGTTTGTTACGCCGCCTTCCACTTTTCCGAGAATTTACTTCTCGAAGATTCACACGTCAACCT
TGACGAAATCAACGCGTGTTTTTCGCGTGATGGAGACAAGCTGACTTTTTCTTTCGCATCTGAGAGCACTTTAA
ATTATTGTCATAGTTATTCTAATATTTTAAAATACGTGTGCAAAACTTACTTCCCGGCATCTAATAGAGAGGTCT
ACATGAAGGAGTTTTTGGTCACCAGGGTTAACACCTGGTTTTGTAAGTTTTCTAGGATAGATACTTTTTTATTAT
ACAAGGGGGTAGCCCACAAAGGTGTAAATAGTGAGCAATTTTACAGCGCAATGGAAGATGCATGGCACTACA
AAAAGACTCTTGCAATGTGTAACAGCGAGAGGATTCTTCTTGAAGATTCCTCATCGGTCAATTACTGGTTCCCA
AAAATGAGAGATATGGTCATAGTTCCTCTATTCGACATATCTCTCGACACCAGTAAAAGGACCCGCAAAGAAG
TCTTAGTGTCAAAGGATTTTGTATTCACAGTTCTAAATCACATTCGCACTTATCAAGCCAAGGCACTTACATACT
CCAATGTTTTATCCTTTGTCGAATCAATTCGTTCAAGGGTAATTATCAACGGAGTGACTGCCAGGTCTGAGTGG
GATGTTGACAAATCTCTTTTGCAATCCTTGTCCATGACATTTTTCTTGCATACTAAGCTTGCCGTTTTAAAAGAC
GAATTGTTAATCAGCAAGTTTAGTTTGGGGCCAAAATCAGTAAGCCAGCATGTATGGGATGAGATTTCCCTGG
CTTTTGGAAACGCATTTCCATCGATCAAGGAGAGACTGCTAAATCGGAAACTAATTAAAGTGTCGGGAGACGC
ATTAGAAATCAGGGTGCCTGATTTATATGTGACTTTTCACGATAGATTAGTGACTGAGTACAAAACATCGGTG
GATATGCCAGTGCTTGATATCAGAAAGAGAATGGAGGAGACTGAGGTTATGTACAATGCATTGTCTGAGCTA
TCTGTGCTCAAGGAGTCGGACAAGTTCGACGTTGATGTTTTTTCCCGGATGTGCCAGACTTTGGAGGTAGACC
CAATGACTGCAGCAAAGGTTATTGTGGCAGTGATGAGCAACGAGAGCGGACTGACTCTCACATTCGAACAGC
CAACTGAAGCAAATGTCGCATTGGCACTTAAAGATTCAGAAAAAGCCTCTGAGGGTGCACTAGTGGTTACTTC
TAGAGATGTTGAAGAACCATCCATGAAGGGTTCAATGGCAAGAGGAGAGTTACAATTGGCCGGTCTGTCTGG
AGACCAACCAGAGTCTTCCTATACTCGGAACGAGGAAATAGAGTCATTAGAGCAATTCCACATGGCAACGGCT
AGTTCGTTAATTCGGAAACAGATGAGTTCGATTGTGTACACGGGCCCCATTAAAGTTCAGCAAATGAAAAACT
TTATTGATAGCCTGGTAGCATCACTCTCTGCTGCGGTGTCGAACCTAGTCAAGATCCTAAAGGATACAGCTGCT
ATAGATCTCGAAACCCGTCAGAAGTTTGGAGTCTTAGATGTTGCGACCAAAAGATGGTTAATTAAACCTTTAG
CCAAGAATCACGCATGGGGCGTTATTGAAACACATGCTAGGAAGTACCACGTTGCACTTTTGGAGTATGATGA
GCATGGAGTGGTAACTTGCGACAGTTGGAGAAGGGTGGCCGTGAGTTCTGAGTCAATGGTTTATTCTGATAT
GGCAAAGCTCAGAACACTGAGGAGATTATTAAGAGATGGTGAGCCTCATGTCAGCAGTGCTAAAGTCGTCCT
AGTTGACGGTGTCCCGGGTTGTGGAAAGACAAAAGAGATTCTCTCGAAAGTAAATTTTGAGGAAGATCTAAT
CTTAGTACCGGGTAAGCAGGCTGCTGAAATGATAAAGAGGCGTGCTAATGCGTCAGGAATAATTCAAGCCAC
AAGAGATAATGTTCGTACTGTTGATTCATTTATAATGAATTACGGTAAAGGAACACGCTGTCAGTTCAAAAGG
TTATTTATCGACGAAGGTCTGATGTTGCACACTGGTTGTGTGAATTTTCTTGTTTCTATGTCTCTGTGCGAAATT
GCATATGTTTATGGAGACACACAACAAATTCCATACATCAACAGAGTATCCGGTTTTCCGTACCCTGCACATTT
TGCAAAAATAGAGGTTGATGAGGTGGAAACTCGCAGAACTACGCTGCGTTGTCCAGCCGACATTACCCACTAT
CTTAACAGAAGGTACGAAGGACATGTCATGTGTACATCGTCGGTTAAAAAGTCAGTTTCTCAGGAAATGGTGA
GCGGGGCCGCAATGATCAATCCTGTATCTAAGCCATTGAATGGGAAAGTTTTGACTTTCACTCAGTCTGACAA
AGAGGCGCTGCTTTCTCGAGGATATACGGACGTCCATACAGTACATGAGGTACAAGGTGAGACATATGCAGA
TGTGTCGTTGGTCAGATTGACTCCGACACCTGTATCTATTATCGCAGGAGATAGTCCGCACGTTCTCGTAGCTT
TGTCAAGGCATACCCAAACATTGAAGTATTACACCGTAGTGATGGATCCTCTTGTAAGTATAATTAGGGATTTA
GAAAAACTTAGTTCTTACTTGTTAGATATGTATAAAGTAGATGCAGGGACCCAATAGCAATTACAGGTAGACT
CCGTGTTTAAAGGTTCTAATCTTTTTGTTGCAGCACCAAAGACTGGAGATATCTCAGATATGCAATTTTACTAT
GATAAGTGTCTCCCAGGTAATAGCACCATGTTAAATAACTATGATGCTGTTACCATGAGGTTGACTGACATTTC
TCTTAATGTCAAAGATTGCATATTGGATTTCTCTAAGTCTGTGGCTGCACCGAAGGATCCGATCAAACCACTGA
TTCCAATGGTACGAACGGCGGCAGAAATGCCACGCCAGACTGGACTATTGGAAAATTTGGTGGCGATGATCA

AAAGAAACTTTAATTCACCGGAGTTATCAGGAATAATCGACATTGAGAATACTGCATCTTTAGTAGTAGATAA
ATTTTTTGATAGTTACTTGCTTAAAGAAAAAAGAAAACCAAATAAAAATGTTTCTTTATTTTGTAGAGAGTCTCT
CAATAGATGGTTAGAGAAGCAGGAGCAAGTGACCATTGGTCAGCTTGCAGATTTTGATTTTGTGGATCTTCCT
GCCGTTGATCAGTACAGGCATATGATTAAAGCGCAACCTAAGCAGAAGCTGGATACATCAATTCAAAGCGAAT
ATCCGGCCTTGCAGACGATTGTGTATCATTCGAAAAAGATCAACGCAATCTTCGGTCCTTTGTTCAGTGAGCTC
ACAAGGCAAATGCTCGAAAGCATAGACTCAAGTAAGTTTTTGTTCTTTACAAGGAAGACGCCAGCTCAAATTG
AGGATTTCTTCGGAGATCTCGATAGCCATGTCCCTATGGATATCTTGGAGTTGGATATTTCGAAGTATGACAAA
TCTCAGAACGAGTTCCACTGTGCAGTAGAGTATGAAATATGGAGAAGACTTGGATTAGAAGATTTTCTGGGA
GAAGTTTGGAAACAAGGCCACAGGAAAACTACTCTTAAAGATTACACAGCTGGTATTAAAACGTGTTTATGGT
ACCAGAGAAAGAGTGGGGACGTTACAACATTCATCGGTAATACGGTGATTATTGCTGCTTGTTTAGCTTCCAT
GTTGCCCATGGAGAAAATAATCAAAGGTGCATTTTGCGGAGATGACAGTTTACTATACTTCCCAAAAGGTTGT
GAGTTTCCTGACATACAGCATACAGCCAACCTTATGTGGAATTTCGAGGCTAAGCTATTCAGAAAGCAGTATG
GTTATTTCTGTGGAAGGTACGTGATACATCATGACAGAGGGTGTATTGTTTATTATGACCCTTTGAAGTTGATT
TCTAAACTTGGTGCTAAACACATCAAGGATTGGGATCACTTAGAAGAGTTCAGAAGATCCCTTTGTGATGTTG
CAAATTCGTTGAACAACTGTGCGTATTACACGCAGTTGGACGACGCTGTGAGTGAGGTCCATAAAACCGCACC
CCCGGGTTCGTTTGTATATAAAAGTTTAGTTAAATATCTGTCCGATAAGGTTCTTTTTAGAAGTTTGTTTATAGA
TGGCTCTTGTTAAGGGTAAAGTCAATATTAATGAGTTCATAGACTTGTCAAAATCAGAAAAATTTCTTCCGTCT
ATGTTCACACCTGTTAAGAGTGTCATGATCTCCAAGGTTGATAAGATATTGGTTCATGAAGATGAATCTTTGTC
CGAAGTCAATTTACTCAAAGGTGTAAAACTCATTGATGGTGGCTATGTACATCTTGCTGGTCTTGTGGTGACA
GGTGAATGGAATTTGCCAGATAATTGTCGTGGTGGTGTCAGTGTCTGTTTGGTCGATAAGAGAATGGAGAGA
GCGGACGAGGCAACTCTTGCTTCGTACTATACCGCAGCGGCTAAGAAAAGGTTTCAGTTCAAAGTCGTTCCAA
ATTACAACATCACTACCAAGGACGCAGAAAAGGCAGTTTGGCAAGTACTAGTTAATATTAGAAATGTTAAAAT
TGCTGCGGGTTACTGTCCGCTGTCATTAGAATTTGTGTCAGTGTGTATTGTTTATAAAAATATTATAAAACTCG
GTTTGAGAGAGAAAATTACGAGCGTCACGGATGGAGGGCCCATGGAACTATCAGAAGAAGTTGTTGATGAG
TTCATGGAAGAAGTCCCGATGTCTGTAAGGCTTGCAAAATTTCGTGCGAAGACCGGAAAAAAGTTTAGTAGTA
AAAGTGAGAATAATAGTGGTAATAATAGGCCGAAACCAAACAAAAACCAAAGGAAGGAAAAGGGTTTAAAA
ATTAGGGTTGAGAAGGATAATTTAATTGATAATGAATTGGAGACTTACGTCGCCGATTCAGATTCGTATTAAA
TATGTCTTACACAATCGCAACTCCATCGCAATTTGTGTTTTTGTCATCAGCATGGGCCGACCCTATAGAATTAAT
AAATTTATGTACTAATTCACTAGGTAATCAGTTCCAAACACAACAAGCTAGAACAACCGTTCAACGGCAATTTA
GCGAAGTGTGGAAACCTGTCCCTCAAGTCACTGTTAGGTTTCCTGACAGTGGTTTTAAGGTGTATAGGTACAA
TGCGGTACTAGATCCTCTAGTTACTGCTTTGTTAGGAGCTTTCGATACTAGAAATAGGATTATAGAAGTCGAAA
ATCAGGCGAACCCGACAACCGCCGAAACGTTAGACGCTACTCGTAGAGTAGATGACGCAACGGTGGCTATAA
GGAGCGCTATAAATAATTTAGTAGTAGAATTGGTCAAAGGAACAGGTTTGTACAATCAGAGCACATTTGAAA
GTGCATCCGGTTTACAATGGTCCTCTGCACCTGCATCTTGA

#MT018320

1

ATGGCATACACACAGACAGCTACCACATCCGCTTTGCTCGACACTGTCCGAGGTAACAATACCTTGGTCAACG
ATCTTGCGAAGCGGCGTCTTTATGACACAGCGGTCGACGAGTTCAACGCTCGTGATCGCAGGCCCAAAGTAA
ATTTTTCCAAAGTAATAAGTGAGGAACAGACGCTTATTGCTACTAGGGCATATCCAGAATTCCAGATAACCTTC
TATAATACGCAGAACGCCGTGCATTCGCTTGCCGGTGGGCTACGATCCTTAGAACTGGAATATCTAATGATGC
AGATCCCGTACGGATCACTCACATATGATATAGGTGGGAATTTTGCATCTCATCTGTTCAAAGGACGGGCATA
TGTTCACTGCTGTATGCCCAATCTTGATGTCCGCGACATAATGCGGCACGAAGGCCAGAAAGACAGTATAGAA
TTATACCTTTCCAGGCTTGAGCGGGGCAACAAAGTTGTCCCAAATTTCCAAAAGGAAGCTTTTGACAGATACG

CTGAAACGCCAGACGAAGTTGTCTGTCACAGTACCTTCCAAACGTGTACGCACCAGCAGGTGGAAAACACAG
GCAGGGTGTATGCTATTGCATTGCACAGTATATACGATATACCTGCTGATGAATTCGGAGCGGCACTTTTAAG
GAAAAATGTCCATGTTTGTTACGCCGCCTTCCACTTTTCCGAGAATTTACTTCTCGAAGATTCACACGTCAACCT
TGACGAAATCAACGCGTGTTTTTCGCGTGATGGAGACAAGCTGACTTTTTCTTTCGCATCTGAGAGCACTTTAA
ATTATTGTCATAGTTATTCTAATATTTTAAAATACGTGTGCAAAACTTACTTCCCGGCATCTAATAGAGAGGTCT
ACATGAAGGAGTTTTTGGTCACCAGGGTTAACACCTGGTTTTGTAAGTTTTCTAGGATAGATACTTTTTTATTAT
ACAAGGGGGTAGCCCACAAAGGTGTAAATAGTGAGCAATTTTACAGCGCAATGGAAGATGCATGGCACTACA
AAAAGACTCTTGCAATGTGTAACAGCGAGAGGATTCTTCTTGAAGATTCCTCATCGGTCAATTACTGGTTCCCA
AAAATGAGAGATATGGTCATAGTTCCTCTATTCGACATATCTCTCGACACCAGTAAAAGGACCCGCAAAGAAG
TCTTAGTGTCAAAGGATTTTGTATTCACAGTTTTAAATCACATTCGCACTTATCAAGCCAAGGCACTTACATACT
CCAATGTTTTATCCTTTGTCGAATCAATTCGTTCAAGGGTAATTATCAACGGAGTGACTGCCAGGTCTGAGTGG
GATGTTGACAAATCTCTTTTGCAATCCTTGTCCATGACATTTTTCTTGCATACTAAGCTTGCCGTTTTAAAAGAC
GAATTGTTAATCAGCAAGTTTAGTTTGGGGCCAAAATCAGTAAGCCAGCATGTATGGGATGAGATTTCCCTGG
CTTTTGGAAACGCATTTCCATCGATCAAGGAGAGACTGCTAAATCGGAAACTAATTAAAGTGTCGGGAGACGC
ATTAGAAATCAGGGTGCCTGATTTATATGTGACTTTTCACGATAGATTAGTGACTGAGTACAAAACATCGGTG
GATATGCCAGTGCTTGATATCAGAAAGAGAATGGAGGAGACTGAGGTTATGTACAATGCATTGTCTGAGCTA
TCTGTGCTCAAGGAGTCGGACAAGTTCGACGTTGATGTTTTTTCCCGGATGTGCCAGACTTTGGAGGTAGACC
CAATGACTGCAGCAAAGGTTATTGTGGCAGTGATGAGCAACGAGAGCGGACTGACTCTTACATTCGAACAGC
CAACTGAAGCAAATGTCGCATTGGCACTTAAAGATTCAGAAAAAGCCTCTGAGGGTGCACTAGTGGTTACTTC
TAGAGATGTTGAAGAACCATCCATGAAGGGTTCAATGGCAAGAGGAGAGTTACAATTGGCCGGTCTGTCTGG
AGACCAACCAGAGTCTTCCTATACTCGGAACGAGGAAATAGAGTCATTAGAGCAATTCCACATGGCAACGGCT
AGTTCGTTAATTCGGAAACAGATGAGTTCGATTGTGTACACGGGCCCCATTAAAGTTCAGCAAATGAAAAACT
TTATTGATAGCCTGGTAGCATCACTCTCTGCTGCGGTGTCGAACCTAGTCAAGATCCTAAAGGATACAGCTGCT
ATAGATCTCGAAACCCGTCAGAAGTTTGGAGTCTTAGATGTTGCGACCAAAAGATGGTTAATTAAACCTTTAG
CCAAGAATCACGCATGGGGCGTTATTGAAACACATGCTAGGAAGTACCACGTTGCACTTTTGGAGTATGATGA
GCATGGAGTGGTAACTTGCGACAGTTGGAGAAGGGTGGCCGTGAGTTCTGAGTCAATGGTTTATTCTGATAT
GGCAAAGCTCAGAACACTGAGGAGATTATTAAGAGATGGTGAGCCTCATGTCAGCAGTGCTAAAGTCGTCCT
AGTTGACGGTGTCCCGGGTTGTGGAAAGACAAAAGAGATTCTCTCGAAAGTAAATTTTGAGGAAGATCTAAT
CTTAGTACCGGGTAAGCAGGCTGCTGAAATGATAAAGAGGCGTGCTAATGCGTCAGGAATAATTCAAGCCAC
AAGAGATAATGTTCGTACTGTTGATTCATTTATAATGAATTACGGTAAAGGAACACGCTGTCAGTTCAAAAGG
TTATTTATCGACGAAGGTCTGATGTTGCACACTGGTTGTGTGAATTTTCTTGTTTCTATGTCTCTGTGCGAAATT
GCATATGTTTATGGAGACACACAACAAATTCCATACATCAACAGAGTATCCGGTTTTCCGTACCCTGCACATTT
TGCAAAAATAGAGGTTGATGAGGTGGAAACTCGCAGAACTACGCTGCGTTGTCCAGCCGACATTACCCACTAT
CTTAACAGAAGGTACGATGGACATGTCATGTGTACATCGTCGGTTAAAAAGTCAGTTTCTCAGGAAATGGTGA
GCGGGGCCGCAATGATCAATCCTGTATCTAAGCCATTGAATGGGAAAGTTTTGACTTTCACTCAGTCTGATAA
AGAGGCGCTGCTTTCTCGAGGATATACGGACGTCCATACAGTACATGAGGTACAAGGTGAGACATATGCAGA
TGTGTCGTTGGTCAGATTGACTCCGACACCTGTATCTATCATCGCAGGAGATAGTCCGCACGTTCTCGTAGCTT
TGTCAAGGCATACCCAAACATTGAAGTATTACACCGTAGTGATGGATCCTCTTGTAAGTATAATTAGGGATTTA
GAAAAACTTAGTTCTTACTTGTTAGATATGTATAAAGTAGATGCAGGGACCCAATAGCAATTACAGGTAGACT
CCGTGTTTAAAGGTTCTAATCTTTTTGTTGCAGCACCAAAGACTGGAGATATCTCAGATATGCAATTTTACTAT
GATAAGTGTCTCCCAGGTAATAGCACCATGTTAAATAACTATGATGCTGTTACCATGAGGTTGACTGACATTTC
TCTTAATGTCAAAGATTGCATATTGGATTTCTCTAAGTCTGTGGCTGCACCGAAGGATCCGATCAAACCACTGA
TTCCAATGGTACGAACGGCGGCAGAAATGCCACGCCAGACTGGACTATTGGAAAATTTGGTGGCGATGATCA
AAAGAAACTTTAATTCACCGGAGTTATCAGGAATAATCGACATTGAGAATACTGCATCTTTAGTAGTAGATAA
ATTTTTTGATAGTTACTTGCTTAAAGAAAAAAGAAAACCAAATAAAAATGTTTCTTTATTTTGTAGAGAGTCTCT
CAATAGATGGTTAGAGAAGCAGGAGCAAGTGACCATTGGTCAGCTTGCAGATTTTGATTTTGTGGATCTTCCT
GCCGTTGATCAGTACAGGCATATGATTAAAGCGCAACCTAAGCAGAAGCTGGATACATCAATTCAAAGCGAAT
ATCCGGCCTTGCAGACGATTGTGTATCATTCGAAAAAGATCAACGCAATCTTCGGTCCTTTGTTCAGTGAGCTC

ACAAGGCAAATGCTCGAAAGCATAGACTCAAGTAAGTTTTTGTTCTTTACAAGGAAGACGCCAGCTCAAATTG
AGGATTTCTTCGGAGATCTCGATAGCCATGTCCCTATGGATATCTTGGAGTTGGATATTTCGAAGTATGACAAA
TCTCAGAACGAGTTCCACTGTGCAGTAGAGTATGAAATATGGAGAAGACTTGGATTAGAAGATTTTCTGGGA
GAAGTTTGGAAACAAGGCCACAGAAAAACTACTCTTAAAGATTACACAGCTGGTATTAAAACGTGTTTATGGT
ACCAGAGAAAGAGTGGGGACGTTACAACATTCATCGGTAATACGGTGATTATTGCTGCTTGTTTAGCTTCCAT
GTTGCCCATGGAGAAAATAATCAAAGGTGCATTTTGCGGAGATGACAGTTTACTATACTTCCCAAAAGGTTGT
GAGTTTCCTGACATACAGCATACAGCCAACCTTATGTGGAATTTCGAGGCTAAGCTATTCAGAAAGCAGTATG
GTTATTTCTGTGGAAGGTACGTGATACATCATGACAGAGGGTGTATTGTTTATTATGACCCTTTGAAGTTGATT
TCTAAACTTGGTGCTAAACATATCAAGGATTGGGATCACTTAGAAGAGTTCAGAAGATCCCTTTGTGATGTTG
CAAATTCGTTGAACAACTGTGCGTATTACACGCAGTTGGACGACGCTGTGAGTGAGGTCCATAAAACCGCACC
CCCGGGTTCGTTTGTATATAAAAGTTTAGTTAAATATCTGTCCGATAAGGTTCTTTTTAGAAGTTTGTTTATAGA
TGGCTCTTGTTAAGGGTAAAGTTAATATTAATGAGTTCATAGACTTGTCAAAATCAGAAAAATTTCTTCCGTCT
ATGTTCACACCTGTTAAGAGTGTCATGATCTCCAAGGTTGATAAGATATTGGTTCATGAAGATGAATCTTTGTC
CGAAGTCAATTTACTTAAAGGTGTAAAACTCATTGATGGTGGCTATGTACATCTTGCTGGTCTTGTGGTGACA
GGTGAATGGAATTTGCCAGATAATTGTCGTGGTGGTGTCAGTGTCTGTTTGGTCGATAAGAGAATGGAGAGA
GCGGACGAGGCAACTCTTGCTTCATACTATACCGCAGCGGCTAAGAAAAGGTTTCAGTTCAAAGTCGTTCCAA
ATTACAACATCACTACCAAGGACGCAGAAAAGGCAGTTTGGCAAGTACTAGTTAATATTAGAAATGTTAAAAT
TGCTGCGGGTTACTGTCCGCTGTCATTAGAATTTGTGTCAGTGTGTATTGTTTATAAAAATATTATAAAACTCG
GTTTGAGAGAGAAAATTACGAGCGTCACGGATGGAGGGCCCATGGAACTATCAGAAGAAGTTGTTGATGAG
TTCATGGAAGAAGTCCCGATGTCTGTAAGGCTTGCAAAATTTCGTTCGAAGACCGGAAAAAAGTTTAGTAGTA
AAAGTGAGAATAATAGTGGTAATAATAGGCCGAAACCAAACAAAAACCAAAGGAAGGAAAAGGGTTTAAAA
GTTAGGGTTGAGAAGGATAATTTAATTGATAATGAATTGGAGACTTACGTCGCCGATTCAGATTCGTATTAAA
TATGTCTTACACAATCGCAACTCCATCGCAATTTGTGTTTTTGTCATCAGCATGGGCCGACCCTATAGAATTAAT
AAATTTATGTACTAATTCACTAGGTAATCAGTTCCAAACACAACAAGCTAGAACAACCGTTCAACGGCAATTTA
GCGAAGTGTGGAAACCTGTCCCTCAAGTCACTGTTAGGTTTCCTGACAGTGGTTTTAAGGTGTATAGGTACAA
TGCGGTACTAGATCCTCTAGTTACTGCTTTGTTAGGAGCTTTCGATACTAGAAATAGGATTATAGAAGTCGAAA
ATCAGGCGAACCCGACAACCGCCGAAACGTTAGACGCTACTCGTAGAGTAGATGACGCAACGGTGGCTATAA
GGAGCGCTATAAATAATTTAGTAGTAGAATTGGTCAAAGGAACAGGTTTGTACAATCAGAGCACTTTTGAAAG
TGCATCCGGTTTACAATGGTCCTCTGCACCTGCATCTTGA

#MT118666

1

ATGGCATACACACAGACAGCTACCACATCCGCTTTGCTCGACACTGTCCGAGGTAACAATACCTTGGTCAACG
ATCTTGCGAAGCGGCGTCTTTATGACACAGCGGTCGACGAGTTCAACGCTCGTGATCGCAGGCCCAAAGTAA
ATTTTTCCAAAGTAATAAGTGAGGAACAGACGCTTATTGCTACTAGGGCATATCCAGAATTCCAGATAACCTTC
TATAATACGCAGAACGCCGTGCATTCGCTTGCCGGTGGACTACGATCCTTAGAACTGGAATATCTAATGATGC
AGATCCCGTACGGATCACTCACATATGATATAGGTGGGAATTTTGCATCTCATCTGTTCAAAGGACGGGCATA
TGTTCACTGCTGTATGCCCAATCTTGATGTCCGCGACATAATGCGGCACGAAGGCCAGAAAGACAGTATAGAA
TTATACCTTTCCAGGCTTGAGCGGGGCAACAAAGTTGTCCCAAATTTCCAAAAGGAAGCTTTTGACAGATACG
CTGAAACGCCAGACGAAGTTGTCTGTCACAGTACCTTCCAAACGTGTACGCACCAGCAGGTGGAAAACACAG
GCAGGGTGTATGCTATTGCATTGCACAGTATATACGATATACCTGCTGATGAATTCGGAGCGGCACTTTTAAG
GAAAAATGTCCATGTTTGTTACGCCGCCTTCCACTTTTCCGAGAATTTACTTCTCGAAGATTCACACGTCAACCT
TGACGAAATCAACGCGTGTTTTTCGCGTGATGGAGACAAGCTGACTTTTTCTTTCGCATCTGAGAGCACTTTAA
ATTATTGTCATAGTTATTCTAATATTTTAAAATACGTGTGCAAAACTTACTTCCCGGCATCTAATAGAGAGGTCT

ACATGAAGGAGTTTTTGGTCACCAGGGTTAACACCTGGTTTTGTAAGTTTTCTAGGATAGATACTTTTTTATTAT
ACAAGGGGGTAGCCCACAAAGGTGTAAATAGTGAGCAATTTTACAGCGCAATGGAAGATGCATGGCACTACA
AAAAGACTCTTGCAATGTGTAACAGCGAGAGGATTCTTCTTGAAGATTCCTCATCGGTCAATTACTGGTTCCCA
AAAATGAGAGATATGGTCATAGTTCCTCTATTCGACATATCTCTCGACACCAGTAAAAGGACCCGCAAAGAAG
TCTTAGTGTCAAAGGATTTTGTATTCACAGTTTTAAATCACATTCGCACTTATCAAGCCAAGGCACTTACATACT
CCAATGTTTTATCCTTTGTCGAATCAATTCGTTCAAGGGTAATTATCAACGGAGTGACTGCCAGGTCTGAGTGG
GATGTTGACAAATCTCTTTTGCAATCCTTGTCCATGACATTTTTCTTGCATACTAAGCTTGCCGTTTTAAAAGAC
GAATTGTTAATCAGCAAGTTTAGTTTGGGGCCAAAATCAGTAAGCCAGCATGTATGGGATGAGATTTCCCTGG
CTTTTGGAAACGCATTTCCATCGATCAAGGAGAGACTGCTAAATCGGAAACTAATTAAAGTGTCGGGAGACGC
ATTAGAAATCAGGGTGCCTGATTTATATGTGACTTTTCACGATAGATTAGTGACTGAGTACAAAACATCGGTG
GATATGCCAGTGCTTGATATCAGAAAGAGAATGGAGGAGACTGAGGTTATGTACAATGCATTGTCTGAGCTA
TCTGTGCTCAAGGAGTCGGACAAGTTCGACGTTGATGTTTTTTCCCGGATGTGCCAGACTTTGGAGGTAGACC
CAATGACTGCAGCAAAGGTTATTGTGGCAGTGATGAGCAACGAGAGCGGACTGACTCTTACATTCGAACAGC
CAACTGAAGCAAATGTCGCATTGGCACTTAAAGATTCAGAAAAAGCCTCTGAGGGTGCACTAGTGGTTACTTC
TAGAGATGTTGAAGAACCATCCATGAAGGGTTCAATGGCAAGAGGAGAGTTACAATTGGCCGGTCTGTCTGG
AGACCAACCAGAGTCTTCCTATACTCGGAACGAGGAAATAGAGTCATTAGAGCAATTCCACATGGCAACGGCT
AGTTCGTTAATTCGGAAACAGATGAGTTCGATTGTGTACACGGGCCCCATTAAAGTTCAGCAAATGAAAAACT
TTATTGATAGCCTGGTAGCATCACTCTCTGCTGCGGTGTCGAACCTAGTCAAGATCCTAAAGGATACAGCTGCT
ATAGATCTCGAAACCCGTCAGAAGTTTGGAGTCTTAGATGTTGCGACCAAAAGATGGTTAATTAAACCTTTAG
CCAAGAATCACGCATGGGGCGTTATTGAAACACATGCTAGGAAGTACCACGTTGCACTTTTGGAGTATGATGA
GCATGGAGTGGTAACTTGCGACAGTTGGAGAAGGGTGGCCGTGAGTTCTGAGTCAATGGTTTATTCTGATAT
GGCAAAGCTCAGAACACTGAGGAGATTATTAAGAGATGGAGAGCCTCATGTCAGCAGTGCTAAAGTCGTCCT
AGTTGACGGTGTCCCGGGTTGTGGAAAGACAAAAGAGATTCTCTCGAAAGTAAATTTTGAGGAAGATCTAAT
CTTAGTACCGGGTAAGCAGGCTGCTGAAATGATAAAGAGGCGTGCTAATGCGTCAGGAATAATTCAAGCCAC
AAGAGATAATGTTCGTACTGTTGATTCATTTATAATGAATTACGGTAAAGGAACACGCTGTCAGTTCAAAAGG
TTATTTATCGACGAAGGTCTGATGTTGCACACTGGTTGTGTGAATTTTCTTGTTTCTATGTCTCTGTGCGAAATT
GCATATGTTTATGGAGACACACAACAAATTCCATACATCAACAGAGTATCCGGTTTTCCGTACCCTGCACATTT
TGCAAAAATAGAGGTTGATGAGGTGGAAACTCGCAGAACTACGCTGCGTTGTCCAGCCGACATTACCCACTAT
CTTAACAGAAGGTACGAAGGACATGTCATGTGTACATCGTCGGTTAAAAAGTCAGTTTCTCAGGAAATGGTGA
GCGGGGCCGCAATGATCAATCCTGTATCTAAGCCATTGAATGGGAAAGTTTTGACTTTCACTCAGTCTGATAA
AGAGGCGCTGCTTTCTCGAGGATATACGGACGTCCATACAGTACATGAGGTACAAGGTGAGACATATGCAGA
TGTGTCGTTGGTCAGATTGACTCCGACACCTGTATCTATCATCGCAGGAGATAGTCCGCACGTTCTCGTAGCTT
TGTCAAGGCATACCCAAACATTGAAGTATTACACCGTAGTGATGGATCCTCTTGTAAGTATAATTAGGGATTTA
GAAAAACTTAGTTCTTACTTGTTAGATATGTATAAAGTAGATGCAGGGACCCAATAGCAATTACAGGTAGACT
CCGTGTTTAAAGGTTCTAATCTTTTTGTTGCAGCACCAAAGACTGGAGATATCTCAGATATGCAATTTTACTAT
GATAAGTGTCTCCCAGGTAATAGCACCATGTTAAATAACTATGATGCTGTTACCATGAGGTTGACTGACATTTC
TCTTAATGTCAAAGATTGCATATTGGATTTCTCTAAGTCTGTGGCTGCACCGAAGGATCCGATCAAACCACTGA
TTCCAATGGTACGAACGGCGGCAGAAATGCCACGCCAGACTGGACTATTGGAAAATTTGGTGGCGATGATCA
AAAGAAACTTTAATTCACCGGAGTTATCAGGAATAATCGACATTGAGAATACTGCATCTTTAGTAGTAGATAA
ATTTTTTGATAGTTACTTGCTTAAAGAAAAAAGAAAACCAAATAAAAATGTTTCTTTATTTTGTAGAGAGTCTCT
CAATAGATGGTTAGAGAAGCAGGAGCAAGTGACCATTGGTCAGCTTGCAGATTTTGATTTTGTGGATCTTCCT
GCCGTTGATCAGTACAGGCATATGATTAAAGCGCAACCTAAGCAGAAGCTGGATACATCAATTCAAAGCGAAT
ATCCGGCCTTGCAGACGATTGTGTATCATTCGAAAAAGATCAACGCAATCTTCGGTCCTTTGTTCAGTGAGCTC
ACAAGGCAAATGCTCGAAAGCATAGACTCAAGTAAGTTTTTGTTCTTTACAAGGAAGACGCCAGCTCAAATTG
AGGATTTCTTCGGAGATCTCGATAGCCATGTCCCTATGGATATCTTGGAGTTGGATATTTCGAAGTATGACAAA
TCTCAGAACGAGTTCCACTGTGCAGTAGAGTATGAAATATGGAGAAGACTTGGATTAGAAGATTTTCTGGGA
GAAGTTTGGAAACAAGGCCACAGGAAAACTACTCTTAAAGATTACACAGCTGGTATTAAAACGTGTTTATGGT
ACCAGAGAAAGAGTGGGGACGTTACAACATTCATCGGTAATACGGTGATTATTGCTGCTTGTTTAGCTTCCAT

GTTGCCCATGGAGAAAATAATCAAAGGTGCATTTTGCGGAGATGACAGTTTACTATACTTCCCAAAAGGTTGT
GAGTTTCCTGACATACAGCATACAGCCAACCTTATGTGGAATTTCGAGGCTAAGCTATTCAGAAAGCAGTATG
GTTATTTCTGTGGAAGGTACGTGATACATCATGACAGAGGGTGTATTGTTTATTATGACCCTTTGAAGTTGATT
TCTAAACTTGGTGCTAAACACATCAAGGATTGGGATCACTTAGAAGAGTTCAGAAGATCCCTTTGTGATGTTG
CAAATTCGTTGAACAACTGTGCGTATTACACGCAGTTGGACGACGCTGTGAGTGAGGTCCATAAAACCGCACC
CCCGGGTTCGTTTGTATATAAAAGTTTAGTTAAATATCTGTCCGATAAGGTTCTTTTTAGAAGTTTGTTTATAGA
TGGCTCTTGTTAAGGGTAAAGTCAATATTAATGAGTTCATAGACTTGTCAAAATCAGAAAAATTTCTTCCGTCT
ATGTTCACACCTGTTAAGAGTGTCATGATCTCCAAGGTTGATAAGATATTGGTTCATGAAGATGAATCTTTGTC
CGAAGTCAATTTACTCAAAGGTGTAAAACTCATTGATGGTGGCTATGTACATCTTGCTGGTCTTGTGGTGACA
GGTGAATGGAATTTGCCAGATAATTGTCGTGGTGGTGTCAGTGTCTGTTTGGTCGATAAGAGAATGGAGAGA
GCGGACGAGGCAACTCTTGCTTCATACTATACCGCAGCGGCTAAGAAAAGGTTTCAGTTCAAAGTCGTTCCAA
ATTACAACATCACTACCAAGGACGCAGAAAAGGCAGTTTGGCAAGTACTAGTTAATATTAGAAATGTTAAAAT
TGCTGCGGGTTACTGTCCGCTGTCATTAGAATTTGTGTCAGTGTGTATTGTTTATAAAAATATTATAAAACTCG
GTTTGAGAGAGAAAATTACGAGCGTCACGGATGGAGGGCCCATGGAACTATCAGAAGAAGTTGTTGATGAG
TTCATGGAAGAAGTCCCGATGTCTGTAAGGCTTGCAAAATTTCGTTCGAAGACCGGAAAAAAGTTTAGTAGTA
AAAGTGAGAATAATAGTGGTAATAATAGGCCGAAACCAAACAAAAACCAAAGGAAGGAAAAGGGTTTAAAA
GTTAGGGTTGAGAAGGATAATTTAATTGATAATGAATTGGAGACTTACGTCGCCGATTCAGATTCGTATTAAA
TATGTCTTACACAATCGCAACTCCATCGCAATTTGTGTTTTTGTCATCAGCATGGGCCGACCCTATAGAATTAAT
AAATTTATGTACTAATTCACTAGGTAATCAGTTCCAAACACAACAAGCTAGAACAACCGTTCAACGGCAATTTA
GCGAAGTGTGGAAACCTGTCCCTCAAGTCACTGTTAGGTTTCCTGACAGTGGTTTTAAGGTGTATAGGTACAA
TGCGGTACTAGATCCTCTAGTTACTGCTTTGTTAGGAGCTTTCGATACTAGAAATAGGATTATAGAAGTCGAAA
ATCAGGCGAACCCGACAACCGCCGAAACGTTAGACGCTACTCGTAGAGTAGATGACGCAACGGTGGCTATAA
GGAGCGCTATAAATAATTTAGTAGTAGAATTGGTCAAAGGAACAGGTTTGTACAATCAGAGCACATTTGAAA
GTGCATCCGGTTTACAATGGTCCTCTGCACCTGCATCTTGA

#MW314091

1

ATGGCATACACACAGACAGCTACCACATCCGCTTTGCTCGACACTGTCCGAGGTAACAATACCTTGGTCAACG
ATCTTGCGAAGCGGCGTCTTTATGACACAGCGGTCGACGAGTTCAACGCTCGTGATCGCAGGCCCAAAGTAA
ATTTTTCCAAAGTAATAAGTGAGGAACAGACGCTTATTGCTACTAGGGCATATCCAGAATTCCAGATAACCTTC
TATAATACGCAGAACGCCGTGCATTCGCTTGCCGGTGGACTACGATCCTTAGAACTGGAATATCTAATGATGC
AGATCCCGTACGGATCACTCACATATGATATAGGTGGGAATTTTGCATCTCATCTGTTCAAAGGACGGGCATA
TGTTCACTGCTGTATGCCCAATCTTGATGTCCGCGACATAATGCGGCACGAAGGCCAGAAAGACAGCATAGAA
TTATACCTTTCCAGGCTTGAGCGGGGCAACAAAGTTGTCCCAAATTTCCAAAAGGAAGCTTTTGACAGATACG
CTGAAACGCCAGACGAAGTTGTCTGTCACAGTACCTTCCAAACGTGTACGCACCAGCAGGTGGAAAACACAG
GCAGGGTGTATGCTATTGCATTGCACAGTATATACGATATACCTGCTGATGAATTCGGAGCGGCACTTTTAAG
GAAAAATGTCCATGTTTGTTACGCCGCCTTCCACTTTTCCGAGAATTTACTTCTCGAAGATTCACACGTCAACCT
TGACGAAATCAACGCGTGTTTTTCGCGTGATGGAGACAAGCTGACTTTTTCTTTCGCATCTGAGAGCACTTTAA
ATTATTGTCATAGTTATTCTAATATTTTAAAATACGTGTGCAAAACTTACTTCCCGGCATCTAATAGAGAGGTCT
ATATGAAGGAGTTTTTGGTCACCAGGGTTAACACCTGGTTTTGTAAGTTTTCTAGGATAGATACTTTTTTATTAT
ACAAGGGGGTAGCCCACAAAGGTGTAAATAGTGAGCAATTTTACAGCGCAATGGAAGATGCATGGCACTACA
AAAAGACTCTTGCAATGTGTAACAGCGAGAGGATTCTTCTTGAAGATTCCTCATCGGTCAATTACTGGTTCCCA
AAAATGAGAGATATGGTCATAGTTCCTCTATTCGACATATCTCTCGACACCAGTAAAAGGACCCGCAAAGAAG
TCTTAGTGTCAAAGGATTTTGTATTCACAGTTTTAAATCACATTCGCACTTATCAAGCCAAGGCACTTACATACT

CCAATGTTTTATCCTTTGTCGAATCAATTCGTTCAAGGGTAATTATCAACGGAGTGACTGCCAGGTCTGAGTGG
GATGTTGACAAATCTCTTTTGCAATCCTTGTCCATGACATTTTTCTTGCATACTAAGCTTGCCGTTTTAAAAGAC
GAATTGTTAATCAGCAAGTTTAGTTTGGGGCCAAAATCAGTAAGCCAGCATGTATGGGATGAGATTTCCCTGG
CTTTTGGAAACGCATTTCCATCGATCAAGGAGAGACTGCTAAATCGGAAACTAATTAAAGTGTCGGGAGACGC
ATTAGAAATCAGGGTGCCTGATTTATATGTGACTTTTCACGATAGATTAGTGACTGAGTACAAAACATCGGTG
GATATGCCAGTGCTTGATATCAGAAAGAGAATGGAGGAGACTGAGGTTATGTACAATGCATTGTCTGAGCTA
TCTGTGCTCAAGGAGTCGGACAAGTTCGACGTTGATGTTTTTTCCCGGATGTGCCAGACTTTGGAGGTAGACC
CAATGACTGCAGCAAAGGTTATTGTGGCAGTGATGAGCAACGAGAGCGGACTGACTCTTACATTCGAACAGC
CAACTGAAGCAAATGTCGCATTGGCACTTAAAGATTCAGAAAAAGCCTCTGAGGGTGCACTAGTGGTTACTTC
TAGAGATGTTGAAGAACCATCCATGAAGGGTTCAATGGCAAGAGGAGAGTTACAATTGGCCGGTCTGTCTGG
AGACCAACCAGAGTCTTCCTATACTCGGAACGAGGAAATAGAGTCATTAGAGCAATTCCACATGGCAACGGCT
AGTTCGTTAATTCGGAAACAGATGAGTTCGATTGTGTACACGGGCCCCATTAAAGTTCAGCAAATGAAAAACT
TTATTGATAGCCTGGTAGCATCACTCTCTGCTGCGGTGTCGAACCTAGTCAAGATCCTAAAGGATACAGCTGCT
ATAGATCTCGAAACCCGTCAGAAGTTTGGAGTCTTAGATGTTGCGACCAAAAGATGGTTAATTAAACCTTTAG
CCAAGAATCACGCATGGGGCGTTATTGAAACACATGCTAGGAAGTACCACGTTGCACTTTTGGAGTATGATGA
GCATGGAGTGGTAACTTGCGACAGTTGGAGAAGGGTGGCCGTGAGTTCTGAGTCAATGGTTTATTCTGATAT
GGCAAAGCTCAGAACACTGAGGAGATTATTAAGAGATGGTGAGCCTCATGTCAGCAGTGCTAAAGTCGTCCT
AGTTGACGGTGTCCCGGGTTGTGGAAAGACAAAAGAGATTCTCTCGAAAGTAAATTTTGAGGAAGATCTAAT
CTTAGTACCGGGTAAGCAGGCTGCTGAAATGATAAAGAGGCGTGCTAATGCGTCAGGAATAATTCAAGCCAC
AAGAGATAATGTTCGTACTGTTGATTCATTTATAATGAATTACGGTAAAGGAACACGCTGTCAGTTCAAAAGG
TTATTTATCGACGAAGGTCTGATGTTGCACACTGGTTGTGTGAATTTTCTTGTTTCTATGTCTCTGTGCGAAATT
GCATATGTTTATGGAGACACACAACAAATTCCATACATCAACAGAGTATCCGGTTTTCCGTACCCTGCACATTT
TGCAAAAATAGAGGTTGATGAGGTGGAAACTCGCAGAACTACGCTGCGTTGTCCAGCCGACATTACCCACTAT
CTTAACAGAAGGTACGAAGGATATGTCATGTGTACATCGTCGGTTAAAAAGTCAGTTTCTCAGGAAATGGTGA
GCGGGGCCGCAATGATCAATCCTGTATCTAAGCCATTGAATGGGAAAGTTTTGACTTTCACTCAGTCTGATAA
AGAGGCGCTGCTTTCTCGAGGATATACGGACGTCCATACAGTACATGAGGTACAAGGTGAGACATATGCAGA
TGTGTCGTTGGTCAGATTGACTCCGACACCTGTATCTATCATCGCAGGAGATAGTCCGCACGTTCTCGTAGCTT
TGTCAAGGCATACCCAAACATTGAAGTATTACACCGTAGTGATGGATCCTCTTGTAAGTATAATTAGGGATTTA
GAAAAACTTAGTTCTTACTTGTTAGATATGTATAAAGTAGATGCAGGGACCCAATAGCAATTACAGGTAGACT
CCGTGTTTAAAGGTTCTAATCTTTTTGTTGCAGCACCAAAGACTGGAGATATCTCAGATATGCAATTTTACTAT
GATAAGTGTCTCCCAGGTAATAGCACCATGTTAAATAACTATGATGCTGTTACCATGAGGTTGACTGACATTTC
TCTTAATGTCAAAGATTGCATATTGGATTTCTCTAAGTCTGTGGCTGCACCGAAGGATCCGATCAAACCACTGA
TTCCAATGGTACGAACGGCGGCAGAAATGCCACGCCAGACTGGACTATTGGAAAATTTGGTGGCGATGATCA
AAAGAAACTTTAATTCACCGGAGTTATCAGGAATAATCGACATTGAGAATACTGCATCTTTAGTAGTAGATAA
ATTTTTTGATAGTTACTTGCTTAAAGAAAAAAGAAAACCAAATAAAAATGTTTCTTTATTTTGTAGAGAGTCTCT
CAATAGATGGTTAGAGAAGCAGGAGCAAGTGACCATTGGTCAGCTTGCAGATTTTGATTTTGTGGATCTTCCT
GCCGTTGATCAGTACAGGCATATGATTAAAGCGCAACCTAAGCAGAAGCTGGATACATCAATTCAAAGCGAAT
ATCCGGCCTTGCAGACGATTGTGTATCATTCGAAAAAGATCAACGCAATCTTCGGTCCTTTGTTCAGTGAGCTC
ACAAGGCAAATGCTCGAAAGCATAGACTCAAGTAAGTTTTTGTTCTTTACAAGGAAGACGCCAGCTCAAATTG
AGGATTTCTTCGGAGATCTCGATAGCCATGTCCCTATGGATATCTTGGAGTTGGATATTTCGAAGTATGACAAA
TCTCAGAACGAGTTCCACTGTGCAGTAGAGTATGAAATATGGAGAAGACTTGGATTAGAAGATTTTCTGGGA
GAAGTTTGGAAACAAGGCCACAGGAAAACTACTCTTAAAGATTACACAGCTGGTATTAAAACGTGTTTATGGT
ACCAGAGAAAGAGTGGGGACGTTACAACATTCATCGGTAATACGGTGATTATTGCTGCTTGTTTAGCTTCCAT
GTTGCCCATGGAGAAAATAATCAAAGGTGCATTTTGCGGAGATGACAGTTTACTATACTTCCCAAAAGGTTGT
GAGTTTCCTGACATACAGCATACAGCCAACCTTATGTGGAATTTCGAGGCTAAGCTATTCAGAAAGCAGTATG
GTTATTTCTGTGGAAGGTACGTGATACATCATGACAGAGGGTGTATTGTTTATTATGACCCTTTGAAGTTGATT
TCTAAACTTGGTGCTAAACACATCAAGGATTGGGATCACTTAGAAGAGTTCAGAAGATCCCTTTGTGATGTTG
CAAATTCGTTGAACAACTGTGCGTATTACACGCAGTTGGACGACGCTGTGAGTGAGGTCCATAAAACCGCACC

CCCGGGTTCGTTTGTATATAAAAGTTTAGTTAAATATCTGTCCGATAAGGTTCTTTTTAGAAGTTTGTTTATAGA
TGGCTCTTGTTAAGGGTAAAGTCAATATTAGTGAGTTCATAGACTTGTCAAAATCAGAAAAATTTCTTCCGTCT
ATGTTCACACCTGTTAAGAGTGTCATGATCTCCAAGGTTGATAAGATATTGGTTCATGAAGATGAATCTTTGTC
CGAAGTCAATTTACTCAAAGGTGTAAAACTCATTGATGGTGGCTATGTACATCTTGCTGGTCTTGTGGTGACA
GGTGAATGGAATTTGCCAGATAATTGTCGTGGTGGTGTCAGTGTCTGTTTGGTCGATAAGAGAATGGAGAGA
GCGGACGAGGCAACTCTTGCATCATACTATACCGCAGCGGCTAAGAAAAGGTTTCAGTTCAAAGTCGTTCCAA
ATTACAACATCACTACCAAGGACGCAGAAAAGGCAGTTTGGCAAGTACTAGTTAATATTAGAAATGTTAAAAT
TGCTGCGGGTTACTGTCCGCTGTCATTAGAATTTGTGTCAGTGTGTATTGTTTATAAAAATATTATAAAACTCG
GTTTGAGAGAGAAAATTACGAGCGTCACGGATGGAGGGCCCATGGAACTATCAGAAGAAGTTGTTGATGAG
TTCATGGAAGAAGTCCCGATGTCTGTAAGGCTTGCAAAATTTCGTTCGAAGACCGGAAAAAAGTTTAGTAGTA
AAAGTGAGAATAATAGTGGTAATAATAGGCCGAAACCAGACAAAAACCAAAGGAAGGAAAAGGGTTTAAAA
GTTAGGGTTGAGAAGGATAATTTAATTGATAATGAATTGGAGACTTACGTCGCCGATTCAGATTCGTATTAAA
TATGTCTTACACAATTGCAACTCCATCGCAATTTGTGTTTTTGTCATCAGCATGGGCCGACCCTATAGAATTAAT
AAATTTATGTACTAATTCACTAGGTAATCAGTTCCAAACACAACAAGCTAGAACAACCGTTCAACGGCAATTTA
GCGAAGTGTGGAAACCTGTCCCTCAAGTCACTGTTAGGTTTCCTGACAGTGGTTTTAAGGTGTATAGGTACAA
TGCGGTACTAGATCCTCTAGTTACTGCTTTGTTAGGAGCTTTCGATACTAGAAATAGGATTATAGAAGTCGAAA
ATCAGGCGAACCCGACAACCGCCGAAACGTTAGACGCTACTCGTAGAGTAGATGACGCAACGGTGGCTATAA
GGAGCGCTATAAATAATTTAGTAGTAGAATTGGTCAAAGGAACAGGTTTGTACAATCAGAGCACATTTGAAA
GTGCATCCGGTTTACAATGGTCCTCTGCACCTGCATCTTGA

#MW314092

1

ATGGCATACACACAGACAGCTACCACATCCGCTTTGCTCGACACTGTCCGAGGTAACAATACCTTGGTCAACG
ATCTTGCGAAGCGGCGTCTTTATGACACAGCGGTCGACGAGTTCAACGCTCGTGATCGCAGGCCCAAAGTAA
ATTTTTCCAAAGTAATAAGTGAGGAACAGACGCTTATTGCTACTAGGGCATATCCAGAATTCCAGATAACCTTC
TATAATACGCAGAACGCCGTGCATTCGCTTGCCGGTGGACTACGATCCTTAGAACTGGAATATCTAATGATGC
AGATCCCGTACGGATCACTCACATATGATATAGGTGGGAATTTTGCATCTCATCTGTTCAAAGGACGGGCATA
TGTTCACTGCTGTATGCCCAATCTTGATGTCCGCGACATAATGCGGCACGAAGGCCAGAAAGACAGTATAGAA
TTATACCTTTCCAGGCTTGAGCGGGGCAACAAAGTTGTCCCAAATTTCCAAAAGGAAGCTTTTGACAGATACG
CTGAAACGCCAGACGAAGTTGTCTGTCACAGTACCTTCCAAACGTGTACGCACCAGCAGGTGGAAAACACAG
GCAGGGTGTATGCTATTGCATTGCACAGTATATACGATATACCTGCTGATGAATTCGGAGCGGCACTTTTAAG
GAAAAATGTCCATGTTTGTTACGCCGCCTTCCACTTTTCCGAGAATTTACTTCTCGAAGATTCACACGTCAACCT
TGACGAAATCAACGCGTGTTTTTCGCGTGATGGAGACAAGCTGACTTTTTCTTTCGCATCTGAGAGCACTTTAA
ATTACTGTCATAGTTATTCTAATATTTTAAAATACGTGTGCAAAACTTACTTCCCGGCATCTAATAGAGAGGTCT
ACATGAAGGAGTTTTTGGTCACCAGGGTTAACACCTGGTTTTGTAAGTTTTCTAGGATAGATACTTTTTTATTAT
ACAAGGGGGTAGCCCACAAAGGTGTAAATAGTGAGCAATTTTACAGCGCAATGGAAGATGCATGGCACTACA
AAAAGACTCTTGCAATGTGTAACAGCGAGAGGATTCTTCTTGAAGATTCCTCATCGGTCAATTACTGGTTCCCA
AAAATGAGAGATATGGTCATAGTTCCTCTATTCGACATATCTCTCGACACCAGTAAAAGGACCCGCAAAGAAG
TCTTAGTGTCAAAGGATTTTGTATTCACAGTTTTAAATCACATTCGCACTTATCAAGCCAAGGCACTTACATACT
CCAATGTTTTATCCTTTGTCGAATCAATTCGTTCAAGGGTAATTATCAACGGAGTGACTGCCAGGTCTGAGTGG
GATGTTGACAAATCTCTTTTGCAATCCTTGTCCATGACATTTTTCTTGCATACTAAGCTTGCCGTTTTAAAAGAC
GAATTGTTAATCAGCAAGTTTAGTTTGGGGCCAAAATCAGTAAGCCAGCATGTATGGGATGAGATTTCCCTGG
CTTTTGGAAACGCATTTCCATCGATCAAGGAGAGACTGCTAAATCGGAAACTAATTAAAGTGTCGGGAGACGC
ATTAGAAATCAGGGTGCCTGATTTATATGTGACTTTTCACGATAGATTAGTGACTGAGTACAAAACATCGGTG

GATATGCCAGTGCTTGATATCAGAAAGAGAATGGAGGAGACTGAGGTTATGTACAATGCATTGTCTGAGCTA
TCTGTGCTCAAGGAGTCGGACAAGTTCGACGTTGATGTTTTTTCCCGGATGTGCCAGACTTTGGAGGTAGACC
CAATGACTGCAGCAAAGGTTATTGTGGCAGTGATGAGCAATGAGAGCGGACTGACTCTTACATTCGAACAGC
CAACTGAAGCAAATGTCGCATTGGCACTTAAAGATTCAGAAAAAGCCTCTGAGGGTGCACTAGTGGTTACTTC
TAGAGATGTTGAAGAACCATCCATGAAGGGTTCAATGGCAAGAGGAGAGTTACAATTGGCCGGTCTGTCTGG
AGACCAACCAGAGTCTTCCTATACTCGGAACGAGGAAATAGTGTCATTAGAGCAATTCCACATGGCAACGGCT
AGTTCGTTAATTCGGAAACAGATGAGTTCGATTGTGTACACGGGCCCCATTAAAGTTCAGCAAATGAAAAACT
TTATTGATAGCCTGGTAGCATCACTCTCTGCTGCGGTGTCGAACCTAGTCAAGATCCTAAAGGATACAGCTGCT
ATAGATCTCGAAACCCGTCAGAAGTTTGGAGTCTTAGATGTTGCGACCAAAAGATGGTTAATTAAACCTTTAG
CCAAGAATCACGCATGGGGCGTTATTGAAACACATGCTAGGAAGTACCACGTTGCACTTTTGGAGTATGATGA
GCATGGAGTGGTAACTTGCGACAGTTGGAGAAGGGTGGCCGTGAGTTCTGAGTCAATGGTTTATTCTGATAT
GGCAAAGCTCAGAACACTGAGGGGATTATTAAGAGATGGTGAGCCTCATGTCAGCAGTGCTAAAGTCGTCCT
AGTTGACGGTGTCCCGGGTTGTGGAAAGACAAAAGAGATTCTCTCGAAAGTAAATTTTGAGGAAGATCTAAT
CTTAGTACCGGGTAAGCAGGCTGCTGAAATGATAAAGAGGCGTGCTAATGCGTCAGGAATAATTCAAGCCAC
AAGAGATAATGTTCGTACTGTTGATTCATTTATAATGAATTACGGTAAAGGAACACGCTGTCAGTTCAAAAGG
TTATTTATCGACGAAGGTCTGATGTTGCACACTGGTTGTGTGAATTTTCTTGTTTCTATGTCTCTGTGCGAAATT
GCATATGTTTATGGAGACACACAACAAATTCCATACATCAACAGAGTATCCGGTTTTCCGTACCCTGCACATTT
TGCAAAAATAGAGGTTGATGAGGTGGAAACTCGCAGAACTACGCTGCGTTGTCCAGCCGACATTACTCACTAT
CTTAACAGAAGGTACGAAGGACATGTCATGTGTACATCGTCGGTTAAAAAGTCAGTTTCTCAGGAAATGGTGA
GCGGGGCCGCAATGATCAATCCTGTATCTAAGCCATTGAATGGGAAAGTTTTGACTTTCACTCAGTCTGATAA
AGAGGCGCTGCTTTCTCGAGGATATACGGACGTCCATACAGTACATGAGGTACAAGGTGAGACATATGCAGA
CGTGTCGTTGGTCAGATTGACTCCGACACCTGTATCTATCATCGCAGGAGATAGTCCGCACGTTCTCGTAGCTT
TGTCAAGGCATACCCAAACATTGAAGTATTACACCGTAGTGATGGATCCTCTTGTAAGTATAATTAGGGATTTA
GAAAAACTTAGTTCTTACTTGTTAGATATGTATAAAGTAGATGCAGGGACCCAATAGCAATTACAGGTAGACT
CCGTGTTTAAAGGTTCTAATCTTTTTGTTGCAGCACCAAAGACTGGAGATATCTCAGATATGCAATTTTACTAT
GATAAGTGTCTCCCAGGTAATAGCACCATGTTAAATAACTATGATGCTGTTACCATGAGGTTGACTGACATTTC
TCTTAATGTCAAAGATTGCATATTGGATTTCTCTAAGTCTGTGGCTGCACCGAAGGATCCGATCAAACCACTGA
TTCCAATGGTACGAACGGCGGCAGAAATGCCACGCCAGACTGGACTATTGGAAAATTTGGTGGCGATGATCA
AAAGAAACTTCAATTCACCGGAGTTATCAGGAATAATCGACATTGAGAATACTGCATCTTTAGTAGTAGATAA
ATTTTTTGATAGTTACTTGCTTAAAGAAAAAAGAAAACCAAATAAAAATGTTTCTTTATTTTGTAGAGAGTCTCT
CAATAGATGGTTAGAGAAGCAGGAGCAAGTGACCATTGGTCAGCTTGCAGATTTTGATTTTGTGGATCTTCCT
GCCGTTGATCAGTACAGGCATATGATTAAAGCGCAACCTAAGCAGAAGCTGGATACATCAATTCAAAGCGAAT
ATCCGGCCTTGCAGACGATTGTGTATCATTCGAAAAAGATCAACGCAATCTTCGGTCCTTTGTTCAGTGAGCTC
ACAAGGCAAATGCTCGAAAGCATAGACTCAAGTAAGTTTTTGTTCTTTACAAGGAAGACGCCAGCTCAAATTG
AGGATTTCTTCGGAGATCTCGATAGCCATGTCCCTATGGATATCTTGGAGTTGGATATTTCGAAGTATGACAAA
TCTCAGAACGAGTTCCACTGTGCAGTAGAGTATGAAATATGGAGAAGACTTGGATTAGAAAATTTTCTGGGA
GAAGTTTGGAAACAAGGCCACAGGAAAACTACTCTTAAAGATTACACAGCTGGTATTAAAACGTGTTTATGGT
ACCAGAGAAAGAGTGGGGACGTTACAACATTCATCGGTAATACGGTGATTATTGCTGCTTGTTTAGCTTCCAT
GTTGCCCATGGAGAAAATAATCAAAGGTGCATTTTGCGGAGATGACAGTTTACTATACTTCCCAAAAGGTTGT
GAGTTTCCTGACATACAGCATACAGCCAACCTTATGTGGAATTTCGAGGCTAAGCTATTCAGAAAGCAGTATG
GTTATTTCTGTGGAAGGTACGTGATACATCATGACAGAGGGTGTATTGTTTATTATGACCCTTTGAAGTTGATT
TCTAAACTTGGTGCTAAACACATCAAGGATTGGGATCACTTAGAAGAGTTCAGAAGATCCCTTTGTGATGTTG
CAAATTCGTTGAACAACTGTGCGTATTACACGCAGTTGGACGACGCTGTGAGTGAGGTCCATAAAACCGCACC
CCCGGGTTCGTTTGTATATAAAAGTTTAGTTAAATATCTGTCCGATAAGGTTCTTTTTAGAAGTTTGTTTATAGA
TGGCTCTTGTTAAGGGTAAAGTCAATATTAATGAGTTCATAGACTTGTCAAAATCAGAAAAATTTCTTCCGTCT
ATGTTCACACCTGTTAAGAGTGTCATGATCTCCAAGGTTGATAAGATATTGGTTCATGAAGATGAATCTTTGTC
CGAAGTCAATTTACTCAAAGGTGTAAAACTCATTGATGGTGGCTATGTACATCTTGCTGGTCTTGTGGTGACA
GGTGAATGGAATTTGCCAGATAATTGTCGTGGTGGTGTCAGTGTCTGTTTGGTCGATAAGAGAATGGAGAGA

GCGGACGAGGCAACTCTTGCTTCATACTATACCGCAGCGGCTAAGAAAAGGTTTCAGTTCAAAGTCGTTCCAA
ATTACAACATCACTACCAAGGACGCAGAAAAGGCAGTTTGGCAAGTACTAGTTAATATTAGAAATGTTAAAAT
TGCTGCGGGTTACTGTCCGCTGTCATTAGAATTTGTGTCAGTGTGTATTGTTTATAAAAATATTATAAAACTCG
GTTTGAGAGAGAAAATTACGAGCGTCACGGATGGAGGGCCCATGGAACTATCAGAAGAAGTTGTTGATGAG
TTCATGGAAGAAGTCCCGATGTCTGTAAGGCTTGCAAAATTTCGTTCGAAGACCGGAAAAAAGTTTAGTAGTA
AAAGTGAGAATAATAGTGGTAATAATAGGCCGAAACCAAACAAAAACCAAAGGAAGGAAAAGGGTTTAAAA
GTTAGGGTTGAGAAGGATAATTTAATTGATAATGAATTGGAGACTTACGTCGCCGATTCAGATTCGTATTAAA
TATGTCTTACACAATCGCAACTCCATCGCAATTTGTGTTTTTGTCATCAGCATGGGCCGACCCTATAGAATTAAT
AAATTTATGTACTAATTCACTAGGTAATCAGTTCCAAACACAACAAGCTAGAACAACCGTTCAACGGCAATTTA
GCGAAGTGTGGAAACCTGTCCCTCAAGTCACTGTTAGGTTTCCTGACAGTGGTTTTAAGGTGTATAGGTACAA
TGCGGTACTAGATCCTCTAGTTACTGCTTTGTTAGGAGCTTTCGATACTAGAAATAGGATTATAGAAGTCGAAA
ATCAGGCGAACCCGACAACCGCCGAAACGTTAGACGCTACTCGTAGAGTAGATGACGCAACGGTGGCTATAA
GGAGCGCTATAAATAATTTAGTAGTAGAATTGGTCAAAGGAACAGGTTTGTACAATCAGAGCACATTTGAAA
GTGCATCCGGTTTACAATGGTCCTCTGCACCTGCATCTTGA

#MW314094

1

ATGGCATACACACAGACAGCTACCACATCCGCTTTGCTCGACACTGTCCGAGGTAACAATACCTTGGTCAACG
ATCTTGCGAAGCGGCGTCTTTATGACACAGCGGTCGACGAGTTCAACGCTCGTGATCGCAGGCCCAAAGTAA
ATTTTTCCAAAGTAATAAGTGAGGAACAGACGCTTATTGCTACTAGGGCATATCCAGAATTCCAGATAACCTTC
TATAATACGCAGAACGCCGTGCATTCGCTTGCCGGTGGACTACGATCCTTAGAACTGGAATATCTAATGATGC
AGATCCCGTACGGATCACTCACATATGATATAGGTGGGAATTTTGCATCTCATCTGTTCAAAGGACGGGCATA
TGTTCACTGCTGTATGCCCAATCTTGATGTCCGCGACATAATGCGGCACGAAGGCCAGAAAGACAGTATAGAA
TTATACCTTTCCAGGCTTGAGCGGGGCAACAAAGTTGTCCCAAATTTCCAAAAGGAAGCTTTTGACAGATACG
CTGAAACGCCAGACGAAGTTGTCTGTCACAGTACCTTCCAAACGTGTACGCACCAGCAGGTGGAAAACACAG
GCAGGGTGTATGCTATTGCATTGCACAGTATATACGATATACCTGCTGATGAATTCGGAGCGGCACTTTTAAG
GAAAAATGTCCATGTTTGTTACGCCGCCTTCCACTTTTCCGAGAATTTACTTCTCGAAGATTCACACGTCAACCT
TGACGAAATCAACGCGTGTTTTTCGCGCGATGGAGACAAGCTGACTTTTTCTTTCGCATCTGAGAGCACTTTAA
ATTACTGTCATAGTTATTCTAATATTTTAAAATACGTGTGCAAAACTTACTTCCCGGCATCTAATAGAGAGGTCT
ACATGAAGGAGTTTTTGGTCACCAGGGTTAACACCTGGTTTTGTAAGTTTTCTAGGATAGATACTTTTTTATTAT
ACAAGGGGGTAGCCCACAAAGGTGTAAATAGTGAGCAATTTTACAGCGCAATGGAAGATGCATGGCACTACA
AAAAGACTCTTGCAATGTGTAACAGCGAGAGGATTCTTCTTGAAGATTCCTCATCGGTCAATTACTGGTTCCCA
AAAATGAGAGATATGGTCATAGTTCCTCTATTCGACATATCTCTCGACACCAGTAAAAGGACCCGCAAAGAAG
TCTTAGTGTCAAAGGATTTTGTATTCACAGTTTTAAATCACATTCGCACTTATCAAGCCAAGGCACTTACATACT
CCAATGTTTTATCCTTTGTCGAATCAATTCGTTCAAGGGTAATTATCAACGGAGTGACTGCCAGGTCTGAGTGG
GATGTTGACAAATCTCTTTTGCAATCCTTGTCCATGACATTTTTCTTGCATACTAAGCTTGCCGTTTTAAAAGAC
GAATTGTTAATCAGCAAGTTTAGTTTGGGGCCAAAATCAGTAAGCCAGCATGTATGGGATGAGATTTCCCTGG
CTTTTGGAAACGCATTTCCATCGATCAAGGAGAGACTGCTAAATCGGAAACTAATTAAAGTGTCGGGAGACGC
ATTAGAAATCAGGGTGCCTGATTTATATGTGACTTTTCACGATAGATTAGTGACTGAGTACAAAACATCGGTG
GATATGCCAGTGCTTGATATCAGAAAGAGAATGGAGGAGACTGAGGTTATGTACAATGCATTGTCTGAGCTA
TCTGTGCTCAAGGAGTCGGACAAGTTCGACGTTGATGTTTTTTCCCGGATGTGCCAGACTTTGGAGGTAGACC
CAATGACTGCAGCAAAGGTTATTGTGGCAGTGATGAGCAATGAGAGCGGACTGACTCTTACATTCGAACAGC
CAACTGAAGCAAATGTCGCATTGGCACTTAAAGATTCAGAAAAAGCCTCTGAGGGTGCACTAGTGGTTACTTC
TAGAGATGTTGAAGAACCATCCATGAAGGGTTCAATGGCAAGAGGAGAGTTACAATTGGCCGGTCTGTCTGG

AGACCAACCAGAGTCTTCCTATACTCGGAACGAGGAAATAGTGTCATTAGAGCAATTCCACATGGCAACGGCT
AGTTCGTTAATTCGGAAACAGATGAGTTCGATTGTGTACACGGGCCCCATTAAAGTTCAGCAAATGAAAAACT
TTATTGATAGCCTGGTAGCATCACTCTCTGCTGCGGTGTCGAACCTAGTCAAGATCCTAAAGGATACAGCTGCT
ATAGATCTCGAAACCCGTCAGAAGTTTGGAGTCTTAGATGTTGCGACCAAAAGATGGTTAATTAAACCTTTAG
CCAAGAATCACGCATGGGGCGTTATTGAAACACATGCTAGGAAGTACCACGTTGCACTTTTGGAGTATGATGA
GCATGGAGTGGTAACTTGCGACAGTTGGAGAAGGGTGGCCGTGAGTTCTGAGTCAATGGTTTATTCTGATAT
GGCAAAGCTCAGAACACTGAGGGGATTATTAAGAGATGGTGAGCCTCATGTCAGCAGTGCTAAAGTCGTCCT
AGTTGACGGTGTCCCGGGTTGTGGAAAGACAAAAGAGATTCTCTCGAAAGTAAATTTTGAGGAAGATCTAAT
CTTAGTACCGGGTAAGCAGGCTGCTGAAATGATAAAGAGGCGTGCTAATGCGTCAGGAATAATTCAAGCCAC
AAGAGATAATGTTCGTACTGTTGATTCATTTATAATGAATTACGGTAAAGGAACACGCTGTCAGTTCAAAAGG
TTATTTATCGACGAAGGTCTGATGTTGCACACTGGTTGTGTGAATTTTCTTGTTTCTATGTCTCTGTGCGAAATT
GCATATGTTTATGGAGACACACAACAAATTCCATACATCAACAGAGTATCCGGTTTTCCGTACCCTGCACATTT
TGCAAAAATAGAGGTTGATGAGGTGGAAACTCGCAGAACTACGCTGCGTTGTCCAGCCGACATTACTCACTAT
CTTAACAGAAGGTACGAAGGACATGTCATGTGTACATCGTCGGTTAAAAAGTCAGTTTCTCAGGAAATGGTGA
GCGGGGCCGCAATGATCAATCCTGTATCTAAGCCATTGAATGGGAAAGTTTTGACTTTCACTCAGTCTGATAA
AGAGGCGCTGCTTTCTCGAGGATATACGGACGTCCATACAGTACATGAGGTACAAGGTGAGACATATGCAGA
CGTGTCGTTGGTCAGATTGACTCCGACACCTGTATCAATCATCGCAGGAGATAGTCCGCACGTTCTCGTAGCTT
TGTCAAGGCATACCCAAACATTGAAGTATTACACCGTAGTGATGGATCCTCTTGTAAGTATAATTAGGGATTTA
GAAAAACTTAGTTCTTACTTGTTAGATATGTATAAAGTAGATGCAGGGACCCAATAGCAATTACAGGTAGACT
CCGTGTTTAAAGGTTCTAATCTTTTTGTTGCAGCACCAAAGACTGGAGATATCTCAGATATGCAATTTTACTAT
GATAAGTGTCTCCCAGGTAATAGCACCATGTTAAATAACTATGATGCTGTTACCATGAGGTTGACTGACATTTC
TCTTAATGTCAAAGATTGCATATTGGATTTCTCTAAGTCTGTGGCTGCACCGAAGGATCCGATCAAACCACTGA
TTCCAATGGTACGAACGGCGGCAGAAATGCCACGCCAGACTGGACTATTGGAAAATTTGGTGGCGATGATCA
AAAGAAACTTCAATTCACCGGAGTTATCAGGAACAATCGACATTGAGAATACTGCATCTTTAGTAGTAGATAA
ATTTTTTGATAGTTACTTGCTTAAAGAAAAAAGAAAACCAAATAAAAATGTTTCTTTATTTTGTAGAGAGTCTCT
CAATAGATGGTTAGAGAAGCAGGAGCAAGTGACCATTGGTCAGCTTGCAGATTTTGATTTTGTGGATCTTCCT
GCCGTTGATCAGTACAGGCATATGATTAAAGCGCAACCTAAGCAGAAGCTGGATACATCAATTCAAAGCGAAT
ATCCGGCCTTGCAGACGATTGTGTATCATTCGAAAAAGATCAACGCAATCTTCGGTCCTTTGTTCAGTGAGCTC
ACAAGGCAAATGCTCGAAAGCATAGACTCAAGTAAGTTTTTGTTCTTTACAAGGAAGACGCCAGCTCAAATTG
AGGATTTCTTCGGAGATCTCGATAGCCATGTCCCTATGGATATCTTGGAGTTGGATATTTCGAAGTATGACAAA
TCTCAGAACGAGTTCCACTGTGCAGTAGAGTATGAAATATGGAGAAGACTTGGATTAGAAAATTTTCTGGGA
GAAGTTTGGAAACAAGGCCACAGGAAAACTACTCTTAAAGATTACACAGCTGGTATTAAAACGTGTTTATGGT
ACCAGAGAAAGAGTGGGGACGTTACAACATTCATCGGTAATACGGTGATTATTGCTGCTTGTTTAGCTTCCAT
GTTGCCCATGGAGAAAATAATCAAAGGTGCATTTTGCGGAGATGACAGTTTACTATACTTCCCAAAAGGTTGT
GAGTTTCCTGACATACAGCATACAGCCAACCTTATGTGGAATTTCGAGGCTAAGCTATTCAGAAAGCAGTATG
GTTATTTCTGTGGAAGGTACGTGATACATCATGACAGAGGGTGTATTGTTTATTATGACCCTTTGAAGTTGATT
TCTAAACTTGGTGCTAAACACATCAAGGATTGGGATCACTTAGAAGAGTTCAGAAGATCCCTTTGTGATGTTG
CAAATTCGTTGAACAACTGTGCGTATTACACGCAGTTGGACGACGCTGTGAGTGAGGTCCATAAAACCGCACC
CCCGGGTTCGTTTGTATATAAAAGTTTAGTTAAATATCTGTCCGATAAGGTTCTTTTTAGAAGTTTGTTTATAGA
TGGCTCTTGTTAAGGGTAAAGTCAATATTAATGAGTTCATAGACTTGTCAAAATCAGAAAAATTTCTTCCGTCT
ATGTTCACACCTGTTAAGAGTGTCATGATCTCCAAGGTTGATAAGATATTGGTTCATGAAGATGAATCTTTGTC
CGAAGTCAATTTACTCAAAGGTGTAAAACTCATTGATGGTGGCTATGTACATCTTGCTGGTCTTGTGGTGACA
GGTGAATGGAATTTGCCAGATAATTGTCGTGGTGGTGTCAGTGTCTGTTTGGTCGATAAGAGAATGGAGAGA
GCGGACGAGGCAACTCTTGCTTCATACTATACCGCAGCGGCTAAGAAAAGGTTTCAGTTCAAAGTCGTTCCAA
ATTACAACATCACTACCAAGGACGCAGAAAAGGCAGTTTGGCAAGTACTAGTTAATATTAGAAATGTTAAAAT
TGCTGCGGGTTACTGTCCGCTGTCATTAGAATTTGTGTCAGTGTGTATTGTTTATAAAAATATTATAAAACTCG
GTTTGAGAGAGAAAATTACGAGCGTCACGGATGGAGGGCCCATGGAACTATCAGAAGAAGTTGTTGATGAG
TTCATGGAAGAAGTCCCGATGTCTGTAAGGCTTGCAAAATTTCGTTCGAAGACCGGAAAAAAGTTTAGTAGTA

AAAGTGAGAATAATAGTGGTAATAATAGGCCGAAACCAAACAAAAACCAAAGGAAGGAAAAGGGTTTAAAA
GTTAGGGTTGAGAAGGATAATTTAATTGATAATGAATTGGAGACTTACGTCGCCGATTCAGATTCGTATTAAA
TATGTCTTACACAATCGCAACTCCATCGCAATTTGTGTTTTTGTCATCAGCATGGGCCGACCCTATAGAATTAAT
AAATTTATGTACTAATTCACTAGGTAATCAGTTCCAAACACAACAAGCTAGAACAACCGTTCAACGGCAATTTA
GCGAAGTGTGGAAACCTGTCCCTCAAGTCACTGTTAGGTTTCCTGACAGTGGTTTTAAGGTGTATAGGTACAA
TGCGGTACTAGATCCTCTAGTTACTGCTTTGTTAGGAGCTTTCGATACTAGAAATAGGATTATAGAAGTCGAAA
ATCAGGCGAACCCGACAACCGCCGAAACGTTAGACGCTACTCGTAGAGTAGATGACGCAACGGTGGCTATAA
GGAGCGCTATAAATAATTTAGTAGTAGAATTGGTCAAAGGAACAGGTTTGTACAATCAGAGCACATTTGAAA
GTGCATCCGGTTTACAATGGTCCTCTGCACCTGCATCTTGA

#MW314098

1

ATGGCATACACACAGACAGCTACCACATCCGCTTTGCTCGACACTGTCCGAGGTAACAATACCTTGGTCAACG
ATCTTGCGAAGCGGCGTCTTTATGACACAGCGGTCGACGAGTTCAACGCTCGTGATCGCAGGCCCAAAGTAA
ATTTTTCCAAAGTAATAAGTGAGGAACAGACGCTTATTGCTACTAGGGCATATCCAGAATTCCAGATAACCTTC
TATAATACGCAGAACGCCGTGCATTCGCTTGCCGGTGGACTACGATCCTTAGAACTGGAATATCTAATGATGC
AGATCCCGTACGGATCACTCACATATGATATAGGTGGGAATTTTGCATCTCATCTGTTCAAAGGACGGGCATA
TGTTCACTGCTGTATGCCCAATCTTGATGTCCGCGACATAATGCGGCACGAAGGCCAGAAAGACAGTATAGAA
TTATACCTTTCCAGGCTTGAGCGGGGCAACAAAGTTGTCCCAAATTTCCAAAAGGAAGCTTTTGACAGATACG
CTGAAACGCCAGACGAAGTTGTCTGTCACAGTACCTTCCAAACGTGTACGCACCAGCAGGTGGAAAACACAG
GCAGGGTGTATGCTATTGCACTGCACAGTATATACGATATACCTGCTGACGAATTCGGAGCGGCACTTTTAAG
GAAAAATGTCCATGTTTGTTACGCCGCCTTCCACTTTTCCGAGAATTTACTTCTCGAAGATTCACACGTCAACCT
TGACGAAATCAATGCGTGTTTTTCGCGTGATGGAGACAAGCTGACTTTTTCTTTCGCATCTGAGAGCACTTTAA
ATTATTGTCATAGTTATTCTAATATTTTAAAATACGTGTGCAAAACTTACTTCCCGGCATCTAATAGAGAGGTCT
ACATGAAGGAGTTTTTGGTCACCAGGGTTAACACCTGGTTTTGTAAGTTTTCTAGGATAGATACTTTTTTATTAT
ACAAGGGGGTAGCCCACAAAGGTGTAAATAGTGAGCAATTTTACAGCGCAATGGAAGATGCATGGCACTACA
AAAAGACTCTTGCAATGTGTAACAGCGAGAGGATTCTTCTTGAAGATTCCTCATCGGTCAATTACTGGTTCCCA
AAAATGAGAGATATGGTCATAGTTCCTCTATTCGACATATCTCTCGACACCAGTAAAAGGACCCGCAAAGAAG
TCTTAGTGTCAAAGGATTTTGTATTCACAGTTTTAAATCACATTCGCACTTATCAAGCCAAGGCACTTACATACT
CCAATGTTTTATCCTTTGTCGAATCAATTCGTTCAAGGGTAATTATCAACGGAGTGACTGCCAGGTCTGAGTGG
GATGTTGACAAATCTCTTTTGCAATCCTTGTCCATGACATTTTTCTTGCATACTAAGCTTGCCGTTTTAAAAGAC
GAATTGTTAATCAGCAAGTTTAGTTTGGGGCCAAAATCAGTAAGCCAGCATGTATGGGATGAGATTTCCCTGG
CTTTTGGAAACGCATTTCCATCGATCAAGGAGAGACTGCTAAATCGGAAACTAATTAAAGTGTCGGGAGACGC
ATTAGAAATCAGGGTGCCTGATTTATATGTGACTTTTCACGATAGATTAGTGACTGAGTACAAAACATCGGTG
GATATGCCAGTGCTTGATATCAGAAAGAGAATGGAGGAGACTGAGGTTATGTACAATGCATTGTCTGAGCTA
TCTGTGCTCAAGGAGTCGGACAAGTTCGACGTTGATGTTTTTTCCCGGATGTGCCAGACTTTGGAGGTAGACC
CAATGACTGCAGCAAAGGTTATTGTGGCAGTGATGAGCAACGAGAGCGGACTGACTCTTACATTCGAACAGC
CAACTGAAGCAAATGTCGCATTGGCACTTAAAGATTCAGAAAAAGCCTCTGAGGGTGCACTAGTGGTTACTTC
TAGAGATGTTGAAGAACCATCCATGAAGGGTTCAATGGCAAGAGGAGAGTTACAATTGGCCGGTCTGTCTGG
AGACCAACCAGAGTCTTCCTATACTCGGAACGAGGAAATAGAGTCATTAGAGCAATTCCACATGGCAACGGCT
AGTTCGTTAATTCGGAAACAGATGAGTTCGATTGTGTACACGGGCCCCATTAAAGTTCAGCAAATGAAAAACT
TTATTGATAGCCTGGTAGCATCACTCTCTGCTGCGGTGTCGAACCTAGTCAAGATCCTAAAGGATACAGCTGCT
ATAGATCTCGAAACCCGTCAGAAGTTTGGAGTCTTAGATGTTGCGACCAAAAGATGGTTAATTAAACCTTTAG
CCAAGAATCACGCATGGGGCGTTATTGAAACACATGCTAGGAAGTACCACGTTGCACTTTTGGAGTATGATGA

GCATGGAGTGGTAACTTGCGACAGTTGGAGAAGGGTGGCCGTGAGTTCTGAGTCAATGGTTTATTCTGATAT
GGCAAAGCTCAGAACACTGAGGAGATTATTAAGAGATGGTGAGCCTCATGTCAGCAGTGCTAAAGTCGTCCT
AGTTGACGGTGTCCCGGGTTGTGGAAAGACAAAAGAGATTCTCTCGAAAGTAAATTTTGAGGAAGATCTGAT
CTTAGTACCGGGTAAGCAGGCTGCTGAAATGATAAAGAGGCGTGCTAATGCGTCAGGAATAATTCAAGCCAC
AAGAGATAATGTTCGTACTGTTGATTCATTTATAATGAATTACGGTAAAGGAACACGCTGTCAGTTCAAAAGG
TTATTTATCGACGAAGGTCTGATGTTGCACACTGGTTGTGTGAATTTTCTTGTTTCTATGTCTCTGTGCGAAATT
GCATATGTTTATGGAGACACACAACAAATTCCATACATCAACAGAGTATCCGGTTTTCCGTACCCTGCACATTT
TGCAAAAATAGAGGTTGATGAGGTGGAAACTCGCAGAACTACGCTGCGTTGTCCAGCCGACATTACCCACTAT
CTTAACAGAAGGTACGAAGGACATGTCATGTGTACATCGTCGGTTAAAAAGTCAGTTTCTCAGGAAATGGTGA
GCGGGGCCGCAATGATCAATCCTGTATCTAAGCCATTGAATGGGAAAGTTTTGACTTTCACTCAGTCTGATAA
AGAGGCGCTGCTTTCTCGAGGATATACGGACGTCCATACAGTACATGAGGTACAAGGTGAGACATATGCAGA
TGTGTCGTTGGTCAGATTGACTCCGACACCTGTATCTATCATCGCAGGAGATAGTCCGCACGTTCTCGTAGCTT
TGTCAAGGCATACCCAAACATTGAAGTATTACACCGTAGTGATGGATCCTCTTGTAAGTATAATTAGGGATTTA
GAAAAACTTAGTTCTTACTTGTTAGATATGTATAAAGTAGATGCAGGGACCCAATAGCAATTACAGGTAGACT
CCGTGTTTAAAGGTTCTAATCTTTTTGTTGCAGCACCAAAGACTGGAGATATCTCAGATATGCAATTTTACTAT
GATAAGTGTCTCCCAGGTAATAGCACCATGTTAAATAACTATGATGCTGTTACCATGAGGTTGACTGACATTTC
TCTTAATGTCAAAGATTGCATATTGGATTTCTCTAAGTCTGTGGCTGCACCGAAGGATCCGATCAAACCACTGA
TTCCAATGGTACGAACGGCGGCAGAAAAGCCACGCCAGACTGGACTATTGGAAAATTTGGTGGCGATGATCA
AAAGAAACTTTAATTCACCGGAGTTATCAGGAATAATCGACATTGAGAATACTGCATCTTTAGTAGTAGATAA
ATTTTTTGATAGTTACTTACTTAAAGAAAAAAGAAAACCAAATAAAAATGTTTCTTTATTTTGTAGAGAGTCTCT
CAATAGATGGTTAGAGAAGCAGGAGCAAGTGACCATTGGTCAGCTTGCAGATTTTGATTTTGTGGATCTTCCT
GCCGTTGATCAGTACAGGCATATGATTAAAGCGCAACCTAAGCAGAAGCTGGATACATCAATTCAAAGCGAAT
ATCCGGCCTTGCAGACGATTGTGTATCATTCGAAAAAGATCAACGCAATCTTCGGTCCTTTGTTCAGTGAGCTC
ACAAGGCAAATGCTCGAAAGCATAGACTCAAGTAAGTTTTTGTTCTTTACAAGGAAGGCGCCAGCTCAAATTG
AGGATTTCTTCGGAGATCTCGATAGCCATGTCCCTATGGATATCTTGGAGTTGGATATTTCGAAGTATGACAAA
TCTCAGAACGAGTTCCACTGTGCAGTAGAGTATGAAATATGGAGAAGACTTGGATTAGAAGATTTTCTGGGA
GAAGTTTGGAAACAAGGCCACAGAAAAACTACTCTTAAAGATTACACAGCTGGTATTAAAACGTGTTTATGGT
ACCAGAGAAAGAGTGGGGACGTTACAACATTCATCGGTAATACGGTGATTATTGCTGCTTGTTTAGCATCCAT
GTTGCCCATGGAGAAAATAATCAAAGGTGCATTTTGCGGAGATGACAGTTTGCTATACTTCCCAAAAGGTTGT
GAGTTTCCTGACATACAGCATACAGCCAACCTTATGTGGAATTTCGAGGCTAAGCTATTCAGAAAGCAGTATG
GTTATTTCTGTGGAAGGTACGTGATACATCACGACAGAGGGTGTATTGTTTATTATGACCCTTTGAAGTTGATT
TCTAAACTTGGTGCTAAACACATCAAGGATTGGGATCACTTAGAAGAGTTCAGAAGATCCCTTTGTGATGTTG
CAAATTCGTTGAACAACTGTGCGTATTACACGCAGTTGGACGACGCTGTGAGTGAGGTCCATAAAACCGCACC
CCCGGGTTCGTTTGTATATAAAAGTTTAGTTAAATATCTGTCCGATAAGGTTCTTTTTAGAAGTTTGTTTATAGA
TGGCTCTTGTTAAGGGTAAAGTCAATATTAATGAGTTCATAGACTTGTCAAAATCAGAAAAATTTCTTCCGTCT
ATGTTCACACCTGTTAAGAGTGTCATGATCTCCAAGGTTGATAAGATATTGGTTCATGAAGATGAATCTTTGTC
CGAAGTCAATTTACTCAAAGGTGTAAAACTCATTGATGGTGGCTATGTACATCTTGCTGGTCTTGTGGTGACA
GGTGAATGGAATTTGCCAGATAATTGTCGTGGTGGTGTCAGTGTCTGTTTGGTCGATAAGAGAATGGAGAGA
GCGGACGAGGCAACTCTTGCTTCATACTATACCGCAGCGGCTAAGAAAAGGTTTCAGTTCAAAGTCGTTCCAA
ATTACAACATCACTACCAAGGACGCAGAAAAGGCAGTTTGGCAAGTACTAGTTAATATTAGAAATGTTAAAAT
TGCTGCGGGTTACTGTCCGCTGTCATTAGAATTTGTGTCAGTGTGTATTGTTTATAAAAATATTATAAAACTCG
GTTTGAGAGAGAAAATTACGAGCGTCACGGATGGAGGGCCTATGGAACTATCTGAAGAAGTTGTTGATGAGT
TCATGGAAGAAGTCCCGATGTCTGTAAGGCTTGCAAAATTTCGTTCGAAGACCGGAAAAAAGTTTAGTAGTAA
AAGTGAGAATAATAGTGGTAATAATAGGCCGAAACCAAACAAAAACCAAAGGAAGGAAAAGGGTTTAAAAG
TTAGGGTTGAGAAGGATAATTTAATTGATAATGAATTGGAGACTTACGTCGCCGATTCAGATTCGTATTAAAT
ATGTCTTACACAATCGCAACTCCATCGCAATTTGTGTTTTTGTCATCAGCATGGGCCGACCCTATAGAATTAATA
AATTTATGTACTAATTCACTAGGTAATCAGTTCCAAACACAACAAGCTAGAACAACCGTTCAACGGCAATTTAG
CGAAGTGTGGAAACCTGTCCCTCAAGTCACTGTTAGGTTTCCTGACAGTGGTTTTAAGGTGTATAGGTACAAT

GCGGTACTAGATCCTCTAGTTACTGCTTTGTTAGGAGCTTTCGATACTAGAAATAGGATTATAGAAGTCGAAA
ATCAGGCGAACCCGACAACCGCCGAAACGTTAGACGCTACTCGTAGAGTAGATGACGCAACGGTGGCTATAA
GGAGCGCTATAAATAATTTAGTAGTAGAATTGGTCAAAGGAACAGGTTTGTACAATCAGAGCACATTTGAAA
GTGCATCCGGTTTACAATGGTCCTCTGCACCTGCATCTTGA

#MW314111

1

ATGGCATACACACAGACAGCTACCACATCCGCTTTGCTCGACACTGTCCGAGGTAACAATACCTTGGTCAATG
ATCTTGCGAAGCGGCGTCTTTATGACACAGCGGTCGACGAGTTCAACGCTCGTGATCGCAGGCCCAAAGTAA
ATTTTTCCAAAGTAATAAGTGAGGAACAGACGCTTATTGCTACTAGGGCATATCCAGAATTCCAGATAACCTTC
TATAATACGCAGAACGCCGTGCATTCGCTTGCCGGTGGACTACGATCCTTAGAACTGGAATATCTAATGATGC
AGATCCCGTACGGATCACTCACATATGATATAGGTGGGAATTTTGCATCTCATCTGTTCAAAGGACGGGCATA
TGTTCACTGCTGTATGCCCAATCTTGATGTCCGCGACATAATGCGGCACGAAGGCCAGAAAGACAGTATAGAA
TTATACCTTTCCAGGCTTGAGCGGGGCAACAAAGTTGTCCCAAATTTCCAAAAGGAAGCTTTTGACAGATACG
CTGAAACGCCAGACGAAATTGTCTGTCACAGTACCTTCCAAACGTGTACGCACCAGCAGGTGGAAAACACAG
GCAGGGTGTATGCTATTGCATTGCACAGTATATACGATATACCTGCTGATGAATTCGGAGCGGCACTTTTAAG
GAAAAATGTCCATGTTTGTTACGCCGCCTTCCACTTTTCCGAGAATTTACTTCTCGAAGATTCACACGTCAACCT
TGACGAAATCAACGCGTGTTTTTCGCGTGATGGAGACAAGCTGACTTTTTCTTTCGCATCTGAGAGCACTTTAA
ATTATTGTCATAGTTATTCTAATATTTTAAAATACGTGTGCAAAACTTACTTCCCGGCATCTAATAGAGAGGTCT
ACATGAAGGAGTTTTTAGTCACCAGGGTTAACACCTGGTTTTGTAAGTTTTCTAGGATAGATACTTTTTTATTAT
ACAAGGGGGTAGCCCACAAAGGTGTAAATAGTGAGCAATTTTACAGCGCAATGGAAGATGCGTGGCACTACA
AAAAGACTCTTGCAATGTGTAACAGCGAGAGGATTCTTCTTGAAGATTCCTCATCGGTCAATTACTGGTTCCCA
AAAATGAGAGATATGGTCATAGTTCCTCTATTCGACATATCTCTCGACACCAGTAAAAGGACCCGCAAAGAAG
TCTTAGTGTCAAAGGATTTTGTATTCACAGTTTTAAATCACATTCGCACTTATCAAGCCAAGGCACTTACATACT
CCAATGTTTTATCCTTTGTCGAATCAATTCGTTCAAGGGTAATTATCAACGGAGTGACTGCCAGGTCTGAGTGG
GATGTTGACAAATCTCTTTTGCAATCCTTGTCCATGACATTTTTCTTGCATACTAAGCTTGCCGTTTTAAAAGAC
GAATTGTTAATCAGCAAGTTTAGTTTGGGGCCAAAATCAGTAAGCCAGCATGTATGGGATGAGATTTCCCTGG
CTTTTGGAAACGCATTTCCATCGATCAAGGAGAGACTGCTAAATCGGAAACTAATTAAAGTGTCGGGAGACGC
ATTAGAAATCAGGGTGCCTGATTTATATGTGACTTTTCACGATAGATTAGTGACTGAGTACAAAACATCGGTG
GATATGCCAGTGCTTGATATCAGAAAGAGAATGGAGGAGACTGAGGTTATGTACAATGCATTGTCTGAGCTA
TCTGTGCTCAAGGAGTCGGACAAGTTCGACGTTGATGTTTTTTCCCGGATGTGCCAGACTTTGGAGGTAGACC
CAATGACTGCAGCAAAGGTTATAGTGGCAGTGATGAGCAACGAGAGCGGACTGACTCTTACATTCGAACAGC
CAACTGAAGCAAATGTCGCATTGGCACTTAAAGATTCAGAAAAAGCCTCTGAGGGTGCACTAGTGGTTACTTC
TAGAGATGTTGAAGAACCATCCATGAAGGGTTCAATGGCAAGAGGAGAGTTACAATTGGCCGGTCTGTCTGG
AGACCAACCAGAGTCTTCCTATACTCGGAACGAGGAAATAGAGTCATTAGAGCAATTCCACATGGCAACGGCT
AGTTCGTTAATTCGGAAACAGATGAGTTCGATTGTGTACACGGGCCCCATTAAAGTTCAGCAAATGAAAAACT
TTATTGATAGCCTAGTAGCATCACTCTCTGCTGCGGTGTCGAACCTAGTCAAGATCCTAAAGGATACAGCTGCT
ATAGATCTCGAAACCCGTCAGAAGTTCGGAGTCTTAGATGTTGCGACCAAAAGATGGTTAATTAAACCTTTAG
CCAAGAATCACGCATGGGGCGTTATTGAAACACATGCTAGGAAGTATCACGTTGCACTTTTGGAGTATGATGA
GCATGGAGTGGTAACTTGCGACAGTTGGAGAAGGGTGGCCGTGAGTTCTGAGTCAATGGTTTATTCTGATAT
GGCAAAGCTCAGAACACTGAGGAGATTATTAAGAGATGGTGAGCCTCATGTCAGCAGTGCTAAAGTCGTCCT
AGTTGACGGTGTCCCGGGTTGTGGAAAGACAAAAGAGATTCTCTCGAAAGTAAATTTTGAGGAAGATCTAAT
CTTAGTACCGGGTAAGCAGGCTGCTGAAATGATAAAGAGGCGTGCTAATGCGTCAGGAATAATTCAAGCCAC
AAGAGATAATGTTCGTACTGTTGATTCATTTATAATGAACTACGGTAAAGGAACACGCTGTCAGTTCAAAAGG

TTATTTATCGACGAAGGTCTGATGTTGCACACTGGTTGTGTGAATTTTCTTGTTTCTATGTCTCTGTGCGAAATT
GCATATGTTTATGGAGACACACAACAAATTCCATACATCAACAGAGTATCCGGTTTTCCGTACCCTGCACATTT
TGCAAAAATAGAGGTTGATGAGGTGGAAACTCGCAGAACTACGCTGCGTTGTCCAGCCGACATTACCCACTAT
CTTAACAGAAGGTACGAAGGACATGTCATGTGTACATCGTCGGTTAAAAAGTCAGTTTCTCAGGAAATGGTGA
GCGGGGCCGCAATGATCAATCCTGTATCTAAGCCATTGAATGGGAAAGTTTTGACTTTCACTCAGTCTGATAA
AGAGGCGCTGCTTTCTCGAGGATATACGGACGTCCATACAGTACATGAGGTACAAGGTGAGACATATGCAGA
TGTGTCGTTGGTCAGATTGACTCCGACACCTGTGTCTATCATCGCAGGAGATAGTCCGCACGTTCTCGTAGCTT
TGTCAAGGCATACCCAAACATTGAAGTATTACACCGTAGTGATGGATCCTCTTGTAAGTATAATTAGGGATTTA
GAAAAACTTAGTTCTTACTTGTTAGATATGTATAAAGTAGATGCAGGGACCCAATAGCAATTACAGGTAGACT
CCGTGTTTAAAGGTTCTAATCTTTTTGTTGCAGCACCAAAGACTGGAGATATCTCAGATATGCAATTTTACTAT
GATAAGTGTCTCCCAGGTAATAGCACCATGTTAAATAACTATGATGCTGTTACCATGAGGTTGACTGACATTTC
TCTTAATGTCAAAGATTGCATATTGGATTTCTCTAAGTCTGTGGCTGCACCGAAGGATCCGATCAAACCACTGA
TTCCAATGGTACGAACGGCGGCAGAAATGCCACGCCAGACTGGACTATTGGAAAATTTGGTGGCGATGATCA
AAAGAAACTTTAATTCACCGGAGTTATCAGGAATAATCGACATTGAGAATACTGCATCTTTAGTAGTAGATAA
ATTTTTTGATAGTTACTTGCTTAAAGAAAAAAGAAAACCAAATAAAAATGTTTCTTTATTTTGTAGAGAGTCTCT
CAATAGATGGTTAGAGAAGCAGGAGCAAGTGACCATTGGTCAGCTTGCAGATTTTGATTTTGTGGATCTTCCT
GCCGTTGATCAGTACAGGCATATGATTAAAGCGCAACCTAAGCAGAAGCTGGATACATCGATTCAAAGCGAA
TATCCGGCCTTGCAGACGATTGTGTATCATTCGAAAAAGATCAACGCAATCTTCGGTCCTTTGTTCAGTGAGCT
CACAAGGCAAATGCTCGAAAGCATAGACTCAAGTAAGTTTTTGTTCTTTACAAGGAAGACGCCAGCTCAAATT
GAGGATTTCTTCGGAGATCTCGATAGCCATGTCCCTATGGATATCTTGGAGTTGGATATTTCGAAGTATGACA
AATCTCAGAACGAGTTCCACTGTGCAGTAGAGTATGAAATATGGAGAAGACTTGGATTAGAAGATTTTCTGG
GAGAAGTTTGGAAACAAGGCCACAGAAAAACTACTCTTAAAGATTACACAGCTGGTATTAAAACGTGTTTATG
GTACCAGAGAAAGAGTGGGGACGTTACAACATTCATCGGTAATACGGTGATTATTGCTGCTTGTTTAGCTTCC
ATGTTGCCCATGGAGAAAATAATCAAAGGTGCATTTTGCGGAGATGACAGTTTACTATACTTCCCAAAAGGTT
GTGAGTTTCCTGACATACAGCATACAGCCAACCTTATGTGGAATTTCGAGGCTAAGCTATTCAGAAAGCAGTA
TGGTTATTTCTGTGGAAGGTACGTGATACATCATGACAGAGGGTGTATTGTTTATTATGACCCTTTGAAGTTGA
TTTCTAAACTTGGTGCTAAACACATCAAGGATTGGGATCACTTAGAAGAGTTCAGAAGATCCCTTTGTGATGTT
GCAATTTCGTTGAACAACTGTGCGTATTACACGCAGTTGGACGACGCTGTGAGTGAGGTCCATAAAACCGCAC
CCCCGGGTTCGTTTGTTTATAAAAGTTTAGTTAAATATCTGTCCGATAAGGTTCTTTTTAGAAGTTTGTTTATAG
ATGGCTCTTGTTAAGGGTAAAGTCAATATTAATGAGTTCATAGACTTGTCAAAATCAGAAAAATTTCTTCCGTC
TATGTTCACACCTGTTAAGAGTGTCATGATCTCCAAGGTTGATAAGATATTGGTTCATGAAGATGAATCTTTGT
CCGAAGTCAATTTACTCAAAGGTGTAAAACTCATTGATGGTGGCTATGTACATCTTGCTGGTCTTGTGGTAACA
GGTGAATGGAATTTGCCAGATAATTGTCGTGGTGGTGTCAGTGTCTGTTTGGTCGATAAGAGAATGGAGAGA
GCGGACGAGGCAACTCTTGCTTCATACTATACCGCAGCGGCTAAGAAAAGGTTTCAGTTCAAAGTCGTTCCAA
ATTACAACATCACTACCAAGGACGCAGAAAAGGCAGTTTGGCAAGTACTAGTTAATATTAGAAATGTTAAAAT
TGCTGCGGGTTACTGTCCGCTGTCATTAGAATTTGTGTCAGTGTGTATTGTTTATAAAAATATTATAAAACTCG
GTTTGAGAGAGAAAATTACGAGCGTCACGGATGGAGGGCCCATGGAACTATCAGAAGAAGTTGTTGATGAG
TTCATGGAAGAAGTCCCGATGTCTGTAAGGCTTGCAAAATTTCGTTCGAAGACCGGAAAAAAGTTTAGTAGTA
AAAGTGAGAATAATAGTGGTAATAATAGGCCGAAACCAGTCAAAAACCAAAGGAAGGAAAAGGGTTTAAAA
GTTAGGGTTGAGAAGGATAATTTAATTGATAATGAATTGGAGACTTACGTCGCCGATTCAGATTCGTATTAAA
TATGTCTTACACAATCGCAACTCCATCGCAATTTGTGTTTTTGTCATCAGCATGGGCCGACCCTATAGAATTAAT
AAATTTATGTACTAATTCACTAGGTAATCAGTTCCAAACACAACAAGCTAGAACAACCGTTCAACGGCAATTTA
GCGAAGTGTGGAAACCTGTCCCTCAAGTCACTGTTAGGTTTCCTGACAGTGGTTTTAAGGTGTATAGGTACAA
TGCGGTACTAGATCCTCTAGTTACTGCTTTGTTAGGAGCTTTCGATACTAGAAATAGGATTATAGAAGTCGAAA
ATCAGGCGAACCCGACAACCGCCGAAACGTTAGACGCTACTCGTAGAGTAGATGACGCAACGGTGGCTATAA
GGAGCGCTATAAATAATTTAGTAGTAGAATTGGTCAAAGGAACAGGTTTGTACAATCAGAGCACATTTGAAA
GTGCATCCGGTTTACAATGGTCCTCTGCACCTGCATCTTGA

#MW314112

1

ATGGCATACACACAGACAGCTACCACATCCGCTTTGCTCGACACTGTCCGAGGTAACAATACCTTGGTCAACG
ATCTTGCGAAGCGGCGTCTTTATGACACAGCGGTCGACGAGTTCAACGCTCGTGATCGCAGGCCCAAAGTAA
ATTTTTCCAAAGTAATAAGTGAGGAACAGACGCTTATTGCTACTAGGGCATATCCTGAATTCCAGATAACCTTC
TATAATACGCAGAACGCCGTGCATTCGCTTGCCGGTGGACTACGATCCTTAGAACTGGAATATCTAATGATGC
AGATCCCGTACGGATCACTCACATATGATATAGGTGGGAATTTTGCATCTCATCTGTTCAAAGGACGGGCATA
TGTTCACTGCTGTATGCCCAATCTTGATGTCCGCGACATAATGCGGCACGAAGGCCAGAAAGACAGTATAGAA
TTATACCTTTCCAGGCTTGAGCGGGGCAACAAAGTTGTCCCAAATTTCCAAAAGGAAGCTTTTGACAGATACG
CTGAAACGCCAGACGAAGTTGTCTGTCACAGTACCTTCCAAACGTGTACGCACCAGCAGGTGGAAAACACAG
GCAGGGTGTATGCTATTGCATTGCACAGTATATACGATATACCTGCTGATGAATTCGGAGCGGCACTTTTAAG
GAAAAATGTCCATGTTTTTTACGCCGCCTTCCACTTTTCCGAGAATTTACTTCTCGAAGATTCACACGTCAACCT
TGACGAAATCAACGCGTGTTTTTCGCGTGATGGAGACAAGCTGACTTTTTCTTTCGCATCTGAGAGCACTTTAA
ATTATTGTCATAGTTATTCTAATATTTTAAAATACGTGTGCAAAACTTACTTCCCGGCATCTAATAGAGAGGTCT
ACATGAAGGAGTTTTTGGTCACCAGGGTTAACACCTGGTTTTGTAAGTTTTCTAGGATAGATACTTTTTTATTAT
ACAAGGGGGTAGCCCACAAAGGTGTAAATAGTGAGCAATTTTACAGCGCAATGGAAGATGCATGGCACTACA
AAAAGACTCTTGCAATGTGTAACAGCGAGAGGATTCTTCTTGAAGATTCCTCATCGGTCAATTACTGGTTCCCA
AAAATGAGAGATATGGTCATAGTTCCTCTATTCGACATATCTCTCGACACTAGTAAAAGGACCCGCAAAGAAG
TCTTAGTGTCAAAGGATTTTGTATTCACAGTTTTAAATCACATTCGCACTTATCAAGCCAAGGCACTTACATACT
CCAATGTTTTATCCTTTGTCGAATCAATTCGTTCAAGGGTAATTATCAACGGAGTGACTGCCAGGTCTGAGTGG
GATGTTGACAAATCTCTTTTGCAATCCTTGTCCATGACATTTTTCTTGCATACTAAGCTTGCCGTTTTAAAAGAC
GAATTGTTAATCAGCAAGTTTAGTTTGGGGCCAAAATCAGTAAGCCAGCATGTATGGGATGAGATTTCCCTGG
CTTTTGGAAACGCATTTCCATCGATCAAGGAGAGACTGCTAAATCGGAAACTAATTAAAGTGTCGGGAGACGC
ATTAGAAATCAGGGTGCCTGATTTATATGTGACTTTTCACGACAGATTAGTGACTGAGTACAAAACATCGGTG
GATATGCCAGTGCTTGATATCAGAAAGAGAATGGAGGAGACTGAGGTTATGTACAATGCATTGTCTGAGCTA
TCTGTGCTCAAGGAGTCGGACAAGTTCGACGTTGATGTTTTTTCCCGGATGTGCCAGACTTTGGAGGTAGACC
CAATGACTGCAGCAAAGGTTATTGTGGCAGTGATGAGCAACGAGAGCGGACTGACTCTTACATTCGAACAGC
CAACTGAAGCAAATGTCGCATTGGCACTTAAAGATTCAGAAAAAGCCTCTGAGGGTGCACTAGTGGTTACTTC
TAGAGATGTTGAAGAACCATCCATGAAGGGTTCAATGGCAAGAGGAGAGTTACAATTGGCCGGTCTGTCTGG
AGACCAACCAGAGTCTTCCTATACTCGGAACGAGGAAATAGAGTCATTAGAGCAATTCCACATGGCAACGGCT
GGTTCGTTAATTCGGAAACAGATGAGTTCGATTGTGTACACGGGCCCCATTAAAGTTCAGCAAATGAAAAACT
TTATTGATAGCCTGGTAGCATCACTCTCTGCTGCGGTGTCGAACCTAGTCAAGATCCTAAAGGATACAGCTGCT
ATAGATCTCGAAACCCGTCAGAAGTTTGGAGTCTTAGATGTTGCGACCAAAAGATGGTTAATTAAACCTTTAG
CCAAGAATCACGCATGGGGCGTTATTGAAACACATGCTAGGAAGTACCACGTTGCACTTTTGGAGTATGATGA
GCATGGAGTGGTAACTAGCGACAGTTGGAGAAGGGTGGCCGTGAGTTCTGAGTCAATGGTTTATTCTGATAT
GGCAAAGCTCAGAACACTGAGGAGATTATTAAGAGATGGTGAGCCTCATGTCAGCAGTGCTAAAGTCGTCCT
AGTTGACGGTGTCCCGGGTTGTGGAAAGACAAAAGAGATTCTCTCGAAAGTAAATTTTGAGGAAGATCTAAT
CTTAGTACCGGGTAAGCAGGCTGCTGAAATGATAAAGAGGCGTGCTAATGCGTCAGGAATAATTCAAGCCAC
AAGAGATAATGTTCGTACTGTTGATTCATTTATAATGAATTACGGTAAAGGAACACGCTGTCAGTTCAAAAGG
TTATTTATCGACGAAGGTCTGATGTTGCACACTGGTTGTGTGAATTTTCTTGTTTCTATGTCTCTGTGCGAAATT
GCATATGTTTATGGAGACACACAACAAATTCCATACATCAACAGAGTATCCGGTTTTCCGTACCCTGCACATTT
TGCAAAAATAGAGGTTGATGAGGTGGAAACTCGCAGAACTACGCTGCGTTGTCCAGCCGACATTACCCACTAT
CTTAACAGAAGGTACGAAGGACATGTCATGTGTACATCGTCGGTTAAAAAGTCAGTTTCTCAGGAAATGGTGA
GCGGGGCCGCAATGATCAATCCTGTATCTAAGCCATTGAATGGGAAAGTTTTGACTTTCACTCAGTCTGATAA

AGAGGCGCTGCTTTCTCGAGGATATACGGACGTCCATACAGTACATGAGGTACAAGGTGAGACATATGCAGA
TGTGTCGTTGGTCAGATTGACTCCGACACCTGTATCTATCATCGCAGGAGATAGTCCGCACGTTCTCGTAGCTT
TGTCAAGGCATACCCAAACATTGAAGTATTACACCGTAGTGATGGATCCTCTTGTAAGTATAATTAGGGATTTA
GAAAAACTTAGTTCTTACTTGTTAGATATGTATAAAGTAGATGCAGGGACCCAATAGCAATTACAGGTAGACT
CCGTGTTTAAAGGTTCTAATCTTTTTGTTGCAGCACCAAAGACTGGAGATATCTCAGATATGCAATTTTACTAT
GATAAGTGTCTCCCAGGTAATAGCACCATGTTAAATAACTATGATGCTGTTACCATGAGGTTGACTGACATTTC
TCTTAATGTCAAAGATTGCATATTGGATTTCTCTAAGTCTGTGGCTGCACCGAAGGATCCGATCAAACCACTGA
TTCCAATGGTACGAACGGCGGCAGAAATGCCACGCCAGACTGGACTATTGGAAAATTTGGTGGCGATGATCA
AAAGAAACTTTAATTCACCGGAGTTATCAGGAATAATCGACATTGAGAATACTGCATCTTTAGTAGTAGATAA
ATTTTTTGATAGTTACTTGCTTAAAGAAAAAAGAAAACCAAATAAAAATGTTTCTTTATTTTGTAGAGAGTCTCT
CAATAGATGGTTAGAGAAGCAGGAGCAAGTGACCATTGGTCAGCTTGCAGATTTTGATTTTGTGGATCTTCCT
GCCGTTGATCAGTACAGGCATATGATTAAAGCGCAACCTAAGCAGAAGCTGGATACATCAATTCAAAGCGAAT
ATCCGGCCTTGCAGACGATTGTGTATCATTCGAAAAAGATCAACGCAATCTTCGGTCCTTTGTTCAGTGAGCTC
ACAAGGCAAATGCTCGAAAGCATAGACTCAAGTAAGTTTTTGTTCTTTACAAGGAAGACGCCAGCTCAAATTG
AGGATTTCTTCGGAGATCTCGATAGCCATGTCCCTATGGATATCTTGGAGTTGGATATTTCGAAGTATGACAAA
TCTCAGAACGAGTTCCACTGTGCAGTAGAGTATGAAATATGGAGAAGACTTGGATTAGAAGATTTTCTGGGA
GAAGTTTGGAAACAAGGCCACAGGAAAACTACTCTTAAAGATTACACAGCTGGTATTAAAACGTGTTTATGGT
ACCAGAGAAAGAGTGGGGACGTTACAACATTCATCGGTAATACGGTGATTATTGCTGCTTGTTTAGCTTCCAT
GTTACCCATGGAGAAAATAATCAAAGGTGCATTTTGCGGAGATGACAGTTTACTATACTTCCCAAAAGGTTGT
GAGTTTCCTGACATACAGCATACAGCCAACCTTATGTGGAATTTCGAGGCTAAGCTATTCAGAAAGCAGTATG
GTTATTTCTGTGGAAGGTACGTGATACATCATGACAGAGGGTGTATTGTTTATTATGACCCTTTGAAGTTGATT
TCTAAACTTGGTGCTAAACACATCAAGGATTGGGATCACTTAGAAGAGTTCAGAAGATCCCTTTGTGATGTTG
CAAATTCGTTGAACAACTGTGCGTATTACACGCAGTTGGACGACGCTGTGAGTGAGGTCCATAAAACCGCACC
CCCGGGTTCGTTTGTATATAAAAGTTTAGTTAAATATCTGTCCGATAAGGTTCTTTTTAGAAGTTTGTTTATAGA
TGGCTCTTGTTAAGGGTAAAGTCAATATTAATGAGTTCATAGACTTGTCAAAATCAGAAAAATTTCTTCCGTCT
ATGTTCACACCTGTTAAGAGTGTCATGATCTCCAAGGTTGATAAGATATTGGTTCATGAAGATGAATCTTTGTC
CGAAGTCAATTTACTCAAAGGTGTAAAACTCATTGATGGTGGCTATGTACATCTTGCTGGTCTTGTGGTGACA
GGTGAATGGAATTTGCCAGATAATTGTCGTGGTGGTGTCAGTGTCTGTTTGGTCGATAAAAGAATGGAGAGA
GCGGACGAGGCAACTCTTGCTTCATACTATACCGCAGCGGCTAAGAAAAGGTTTCAGTTCAAGGTCGTTCCAA
ATTACAACATCACTACCAAGGACGCAGAAAAGGCAGTTTGGCAAGTACTAGTTAATATTAGAAATGTTAAAAT
TGCTGCGGGTTACTGTCCGCTGTCATTAGAATTTGTGTCAGTGTGTATTGTTTATAAAAATATTATAAAACTCG
GTTTGAGAGAGAAAATTACGAGCGTCACGGATGGAGGGCCCATGGAACTATCAGAAGAAGTTGTTGATGAG
TTCATGGAAGAAGTCCCGATGTCTGTAAGGCTTGCAAAATTTCGTTCGAAGACCGGGAAAAAGTTTAGTAGTA
AAAGTGAGAATAATAGTGGTAATAATAGGCCGAAACCAAACAAAAACCAAAGGAAGGAAAAGGGTTTAAAA
GTTAGGGTTGAGAAGGATAATTTAATTGATAATGAATTGGAGACTTACGTCGCCGATTCAGATTCGTATTAAA
TATGTCTTACACAATCGCAACTCCATCGCAATTTGTGTTTTTGTCATCAGCATGGGCCGACCCTATAGAATTAAT
AAATTTATGTACTAATTCACTAGGTAATCAATTCCAAACACAACAAGCTAGAACAACCGTTCAACGGCAATTTA
GCGAAGTGTGGAAACCTGTCCCTCAAGTCACTGTTAGGTTTCCTGACAGTGGTTTTAAGGTGTATAGGTACAA
TGCGGTACTAGAACCTCTAGTTACTGCTTTGTTAGGAGCTTTCGATACTAGAAATAGGATTATAGAAGTCGAA
AATCAGGCGAACCCGACAACCGCCGAAACGTTAGACGCTACTCGTAGAGTAGATGACGCAACGGTGGCTATA
AGGAGCGCTATAAATAATTTAGTAGTAGAATTGGTCAAAGGAACAGGTTTGTACAATCAGAGCACATTTGAA
AGTGCATCCGGTTTACAATGGTCCTCTGCACCTGCATCTTGA

#MW314118

ATGGCATACACACAGACAGCTACCACATCCGCTTTGCTCGACACTGTCCGAGGTAACAATACCTTGGTCAACG
ATCTTGCGAAGCGGCGTCTTTATGACACAGCGGTCGACGAGTTCAACGCTCGTGATCGCAGGCCCAAAGTAA
ATTTTTCCAAAGTAATAAGTGAGGAACAGACGCTTATTGCTACTAGGGCATATCCTGAATTCCAGATAACCTTC
TATAATACGCAGAACGCCGTGCATTCGCTTGCCGGTGGACTACGATCCTTAGAACTGGAATATCTAATGATGC
AGATCCCGTACGGATCACTCACATATGATATAGGTGGGAATTTTGCATCTCATCTGTTCAAAGGACGGGCATA
TGTTCACTGCTGTATGCCCAATCTTGATGTCCGCGACATAATGCGGCACGAAGGCCAGAAAGACAGTATAGAA
TTATACCTTTCCAGGCTTGAGCGGGGCAACAAAGTTGTCCCAAATTTCCAAAAGGAAGCTTTTGACAGATACG
CTGAAACGCCAGACGAAGTTGTCTGTCACAGTACCTTCCAAACGTGTACGCACCAGCAGGTGGAAAACACAG
GCAGGGTGTATGCTATTGCATTGCACAGTATATACGATATACCTGCTGATGAATTCGGAGCGGCACTTTTAAG
GAAAAATGTCCATGTTTGTTACGCCGCCTTCCACTTTTCCGAGAATTTACTTCTCGAAGATTCACACGTCAACCT
TGACGAAATCAACGCGTGTTTTTCGCGTGATGGAGACAAGCTGACTTTTTCTTTCGCATCTGAGAGCACTTTAA
ATTATTGTCATAGTTATTCTAATATTTTAAAATACGTGTGCAAAACTTACTTCCCGGCATCTAATAGAGAGGTCT
ACATGAAGGAGTTTTTGGTCACCAGGGTTAACACCTGGTTTTGTAAGTTTTCTAGGATAGATACTTTTTTATTAT
ACAAGGGGGTAGCCCACAAAGGTGTAAATAGTGAGCAATTTTACAGCGCAATGGAAGATGCATGGCACTACA
AAAAGACTCTTGCAATGTGTAACAGCGAGAGGATTCTTCTTGAAGATTCCTCATCGGTCAATTACTGGTTCCCA
AAAATGAGAGATATGGTCATAGTTCCTCTATTCGACATATCTCTCGACACTAGTAAAAGGACCCGCAAAGAAG
TCTTAGTGTCAAAGGATTTTGTATTCACAGTTTTAAATCACATTCGCACTTATCAAGCCAAGGCACTTACATACT
CCAATGTTTTATCCTTTGTCGAATCAATTCGTTCAAGGGTAATTATCAACGGAGTGACTGCCAGGTCTGAGTGG
GATGTTGACAAATCTCTTTTGCAATCCTTGTCCATGACATTTTTCTTGCATACTAAGCTTGCCGTTTTAAAAGAC
GAATTGTTAATCAGCAAGTTTAGTTTGGGGCCAAAATCAGTAAGCCAGCATGTATGGGATGAGATTTCCCTGG
CTTTTGGAAACGCATTTCCATCGATCAAGGAGAGACTGCTAAATCGGAAACTAATTAAAGTGTCGGGAGACGC
ATTAGAAATCAGGGTGCCTGATTTATATGTGACTTTTCACGATAGATTAGTGACTGAGTACAAAACATCGGTG
GATATGCCAGTGCTTGATATCAGAAAGAGAATGGAGGAGACTGAGGTTATGTACAATGCATTGTCTGAGCTA
TCTGTGCTCAAGGAGTCGGACAAGTTCGACGTTGATGTTTTTTCCCGGATGTGCCAGACTTTGGAGGTAGACC
CAATGACTGCAGCAAAGGTTATTGTGGCAGTGATGAGCAACGAGAGCGGACTGACTCTTACATTCGAACAGC
CAACTGAAGCAAATGTCGCATTGGCACTTAAAGATTCAGAAAAAGCCTCTGAGGGTGCACTAGTGGTTACTTC
TAGAGATGTTGAAGAACCATCCATGAAGGGTTCAATGGCAAGAGGAGAGTTACAATTGGCCGGTCTGTCTGG
AGACCAACCAGAGTCTTCCTATACTCGGAACGAGGAAATAGAGTCATTAGAGCAATTCCACATGGCAACGGCT
GGTTCGTTAATTCGGAAACAGATGAGTTCGATTGTGTACACGGGCCCCATTAAAGTTCAGCAAATGAAAAACT
TTATTGATAGCCTGGTAGCATCACTCTCTGCTGCGGTGTCGAACCTAGTCAAGATCCTAAAGGATACAGCTGCT
ATAGATCTCGAAACCCGTCAGAAGTTTGGAGTCTTAGATGTTGCGACCAAAAGATGGTTAATTAAACCTTTAG
CCAAGAATCACGCATGGGGCGTTATTGAAACACATGCTAGGAAGTACCACGTTGCACTTTTGGAGTATGATGA
GCATGGAGTGGTAACTTGCGACAGTTGGAGAAGGGTGGCCGTGAGTTCTGAGTCAATGGTTTATTCTGATAT
GGCAAAGCTCAGAACACTGAGGAGATTATTAAGAGATGGTGAGCCTCATGTCAGTAGTGCTAAAGTCGTCCT
AGTTGACGGTGTCCCGGGTTGTGGAAAGACAAAAGAGATTCTCTCGAAAGTAAATTTTGAGGAAGATCTAAT
CTTAGTACCGGGTAAGCAGGCTGCTGAAATGATAAAGAGGCGTGCTAATGCGTCAGGAATAATTCAAGCCAC
AAGAGATAATGTTCGTACTGTTGATTCATTTATAATGAATTACGGTAAAGGAACACGCTGTCAGTTCAAAAGG
TTATTTATCGACGAAGGTCTGATGTTGCACACTGGTTGTGTGAATTTTCTTGTTTCTATGTCTCTGTGCGAAATT
GCATATGTTTATGGAGACACACAACAAATTCCATACATCAACAGAGTATCCGGTTTTCCGTACCCTGCACATTT
TGCAAAAATAGAGGTTGATGAGGTGGAAACTCGCAGAACTACGCTGCGTTGTCCAGCCGACATTACCCACTAT
CTTAACAGAAGGTACGAAGGACATGTCATGTGTACATCGTCGGTTAAAAAGTCAGTTTCTCAGGAAATGGTGA
GCGGGGCCGCAATGATCAATCCTGTATCTAAGCCATTGAATGGGAAAGTTTTGACTTTCACTCAGTCTGATAA
AGAGGCGCTGCTTTCTCGAGGATATACGGACGTCCATACAGTACATGAGGTACAAGGTGAGACATATGCAGA
TGTGTCGTTGGTCAGATTGACTCCGACACCTGTATCTATCATCGCAGGAGATAGTCCGCACGTTCTCGTAGCTT
TGTCAAGGCATACCCAAACATTGAAGTATTACACCGTAGTGATGGATCCTCTTGTAAGTATAATTAGGGATTTA
GAAAAACTTAGTTCTTACTTGTTAGATATGTATAAAGTAGATGCAGGGACCCAATAGCAATTACAGGTAGACT
CCGTGTTTAAAGGTTCTAATCTTTTTGTTGCAGCACCAAAGACTGGAGATATCTCAGATATGCAATTTTACTAT
GATAAGTGTCTCCCAGGTAATAGCACCATGTTAAATAACTATGATGCTGTTACCATGAGGTTGACTGACATTTC

TCTTAATGTCAAAGATTGCATATTGGATTTCTCTAAGTCTGTGGCTGCACCGAAGGATCCGATCAAACCACTGA
TTCCAATGGTACGAACGGCGGCAGAAATGCCACGCCAGACTGGACTATTGGAAAATTTGGTGGCGATGATCA
AAAGAAACTTTAATTCACCGGAGTTATCAGGAATAATCGACATTGAGAATACTGCATCTTTAGTAGTAGATAA
ATTTTTTGATAGTTACTTGCTTAAAGAAAAAAGAAAACCAAATAAAAATGTTTCTTTATTTTGTAGAGAGTCTCT
CAATAGATGGTTAGAGAAGCAGGAGCAAGTGACCATTGGTCAGCTTGCAGATTTTGATTTTGTGGATCTTCCT
GCCGTTGATCAGTACAGGCATATGATTAAAGCGCAACCTAAGCAGAAGCTGGATACATCAATTCAAAGCGAAT
ATCCGGCCTTGCAGACGATTGTGTATCATTCGAAAAAGATCAACGCAATCTTCGGTCCTTTGTTCAGTGAGCTC
ACAAGGCAAATGCTCGAAAGCATAGACTCAAGTAAGTTTTTGTTCTTTACAAGGAAGACGCCAGCTCAAATTG
AGGATTTCTTCGGAGATCTCGATAGCCATGTCCCTATGGATATCTTGGAGTTGGATATTTCGAAGTATGACAAA
TCTCAGAACGAGTTCCACTGTGCAGTAGAGTATGAAATATGGAGAAGACTTGGATTAGAAGATTTTCTGGGA
GAAGTTTGGAAACAAGGCCACAGGAAAACTACTCTTAAAGATTACACAGCTGGTATTAAAACGTGTTTATGGT
ACCAGAGAAAGAGTGGGGACGTTACAACATTCATCGGTAATACGGTGATTATTGCTGCTTGTTTAGCTTCCAT
GTTGCCCATGGAGAAAATAATCAAAGGTGCATTTTGCGGAGATGACAGTTTACTATACTTCCCAAAAGGTTGT
GAGTTTCCTGACATACAGCATACAGCCAACCTTATGTGGAATTTCGAGGCTAAGCTATTCAGAAAGCAGTATG
GTTATTTCTGTGGAAGGTACGTGATACATCATGACAGAGGGTGTATTGTTTATTATGACCCTTTGAAGTTGATT
TCTAAACTTGGTGCTAAACACATCAAGGATTGGGATCACTTAGAAGAGTTCAGAAGATCCCTTTGTGATGTTG
CAAATTCGTTGAACAACTGTGCGTATTACACGCAGTTGGACGACGCTGTGAGTGAGGTCCATAAAACCGCACC
CCCGGGTTCGTTTGTATATAAAAGTTTAGTTAAATATCTGTCCGATAAGGTTCTTTTTAGAAGTTTGTTTATAGA
TGGCTCTTGTTAAGGGTAAAGTCAATATTAATGAGTTCATAGACTTGTCAAAATCAGAAAAATTTCTTCCGTCT
ATGTTCACACCTGTTAAGAGTGTCATGATCTCCAAGGTTGATAAGATATTGGTTCATGAAGATGAATCTTTGTC
CGAAGTCAATTTACTCAAAGGTGTAAAACTCATTGATGGTGGCTATGTACATCTTGCTGGTCTTGTGGTGACA
GGTGAATGGAATTTGCCAGATAATTGTCGTGGTGGTGTCAGTGTCTGTTTGGTCGATAAAAGAATGGAGAGA
GCGGACGAGGCAACTCTTGCTTCATACTATACCGCAGCGGCTAAGAAAAGGTTTCAGTTCAAAGTCGTTCCAA
ATTACAACATCACTACCAAGGACGCAAAAAAGGCAGTTTGGCAAGTACTAGTTAATATTAGAAATGTTAAAAT
TGCTGCGGGTTACTGTCCGCTGTCATTAGAATTTGTGTCAGTGTGTATTGTTTATAAAAATATTATAAAACTCG
GTTTGAGAGAGAAAATTACGAGCGTAACGGATGGAGGGCCCATGGAACTATCAGAAGAAGTTGTTGATGAG
TTCATGGAAGAAGTCCCGATGTCTGTAAGGCTTGCAAAATTTCGTTCGAAGACCGGGAAAAAGTTTAGTAGTA
AAAGTGAGAATAATAGTGGTAATAATAGGCCGAAACCAAACAAAAACCAAAGGAAGGAAAAGGGTTTAAAA
GTTAGGGTTGAGAAGGATAATTTAATTGATAATGAATTGGAGACTTACGTCGCCGATTCAGATTCGTATTAAA
TATGTCTTACACAATCGCAACTCCATCGCAATTTGTGTTTTTGTCATCAGCATGGGCCGACCCTATAGAATTAAT
AAATTTATGTACTAATTCACTAGGTAATCAATTCCAAACACAACAAGCTAGAACAACCGTTCAACGGCAATTTA
GCGAAGTGTGGAAACCTGTCCCTCAAGTCACTGTTAGGTTTCCTGACAGTGGTTTTAAGGTGTATAGGTACAA
TGCGGTACTAGATCCTCTAGTTACTGCTTTGTTAGGAGCTTTCGATACTAGAAATAGGATTATAGAAGTCGAAA
ATCAGGCGAACCCGACAACCGCCGAAACGTTAGACGCTACTCGTAGAGTAGATGACGCAACGGTGGCTATAA
GGAGCGCTATAAATAATTTAGTAGTAGAATTGGTCAAAGGAACAGGTTTGTACAATCAGAGCACATTTGAAA
GTGCATCCGGTTTACAATGGTCCTCTGCACCTGCATCTTGA

#MW314119

1

ATGGCATACACACAGACAGCTACCACATCCGCTTTGCTCGACACTGTCCGAGGTAACAATACCTTGGTCAACG
ATCTTGCGAAGCGGCGTCTTTATGACACAGCGGTCGACGAGTTCAACGCTCGTGATCGCAGGCCCAAAGTAA
ATTTTTCCAAAGTAATAAGTGAGGAACAGACGCTTATTGCTACTAGGGCATATCCTGAATTCCAGATAACCTTC
TATAATACGCAGAACGCCGTGCATTCGCTTGCCGGTGGACTACGATCCTTAGAACTGGAATATCTAATGATGC
AGATCCCGTACGGATCACTCACATATGATATAGGTGGGAATTTTGCATCTCATCTGTTCAAAGGACGGGCATA

TGTTCACTGCTGTATGCCCAATCTTGATGTCCGCGACATAATGCGGCACGAAGGCCAGAAAGACAGTATAGAA
TTATACCTTTCCAGGCTTGAGCGGGGCAACAAAGTTGTCCCAAATTTCCAAAAGGAAGCTTTTGACAGATACG
CTGAAACGCCAGACGAAGTTGTCTGTCACAGTACCTTCCAAACGTGTACGCACCAGCAGGTGGAAAACACAG
GCAGGGTGTATGCTATTGCATTGCACAGTATATACGATATACCTGCTGATGAATTCGGAGCAGCACTTTTAAG
GAAAAATGTCCATGTTTGTTACGCCGCCTTCCACTTTTCCGAGAATTTACTTCTCGAAGATTCACACGTCAACCT
TGACGAAATCAACGCGTGTTTTTCGCGTGATGGAGACAAGCTGACTTTTTCTTTCGCATCTGAGAGCACTTTAA
ATTATTGTCATAGTTATTCTAATATTTTAAAATACGTGTGCAAAACTTACTTCCCGGCATCTAATAGAGAGGTCT
ACATGAAGGAGTTTTTGGTCACCAGGGTTAACACCTGGTTTTGTAAGTTTTCTAGGATAGATACTTTTTTATTAT
ACAAGGGGGTAGCCCACAAAGGTGTAAATAGTGAGCAATTTTACAGCGCAATGGAAGATGCATGGCACTACA
AAAAGACTCTTGCAATGTGTAACAGCGAGAGGATTCTTCTTGAAGATTCCTCATCGGTCAATTACTGGTTCCCA
AAAATGAGAGATATGGTCATAGTTCCTCTATTCGACATATCTCTCGACACTAGTAAAGGACCCGCAAAGAAG
TCTTAGTGTCAAAGGATTTTGTATTCACAGTTTTAAATCACATTCGCACTTATCAAGCCAAGGCACTTACATACT
CCAATGTTTTATCCTTTGTCGAATCAATTCGTTCAAGGGTAATTATCAACGGAGTGACTGCCAGGTCTGAGTGG
GATGTTGACAAATCTCTTTTGCAATCCTTGTCCATGACATTTTTCTTGCATACTAAGCTTGCCGTTTTAAAAGAC
GAATTGTTAATCAGCAAGTTTAGTTTGGGGCCAAAATCAGTAAGCCAGCATGTATGGGATGAGATTTCCCTGG
CTTTTGGAAACGCATTTCCATCGATCAAGGAGAGACTGCTAAATCGGAAACTAATTAAAGTGTCGGGAGACGC
ATTAGAAATCAGGGTGCCTGATTTATATGTGACTTTTCACGATAGATTAGTGACTGAGTACAAAACATCGGTG
GATATGCCAGTGCTTGATATCAGAAAGAGAATGGAGGAGACTGAGGTTATGTACAATGCATTGTCTGAGCTA
TCTGTGCTCAAGGAGTCGGACAAGTTCGACGTTGATGTTTTTTCCCGGATGTGCCAGACTTTGGAGGTAGACC
CAATGACTGCAGCAAAGGTTATTGTGGCAGTGATGAGCAACGAGAGCGGACTGACTCTTACATTCGAACAGC
CAACTGAAGCAAATGTCGCATTGGCACTTAAAGATTCAGAAAAAGCCTCTGAGGGTGCACTAGTGGTTACTTC
TAGAGATGTTGAAGAACCATCCATGAAGGGTTCAATGGCAAGAGGAGAGTTACAATTGGCCGGTCTGTCTGG
AGACCAACCAGAGTCTTCCTATACTCGGAACGAGGAAATAGAGTCATTAGAGCAATTCCACATGGCAACGGCT
GGTTCGTTAATTCGGAAACAGATGAGTTCGATTGTGTACACGGGCCCCATTAAAGTTCAGCAAATGAAAAACT
TTATTGATAGCCTGGTAGCATCACTCTCTGCTGCGGTGTCGAACCTAGTCAAGATCCTAAAGGATACAGCTGCT
ATAGATCTCGAAACCCGTCAGAAGTTTGGAGTCTTAGATGTTGCGACCAAAAGATGGTTAATTAAACCTTTAG
CCAAGAATCACGCATGGGGCGTTATTGAAACACATGCTAGGAAGTACCACGTTGCACTTTTGGAGTATGATGA
GCATGGAGTGGTAACTTGCGACAGTTGGAGAAGGGTGGCCGTGAGTTCTGAGTCAATGGTTTATTCTGATAT
GGCAAAGCTCAGAACACTGAGGAGATTATTAAGAGATGGTGAGCCTCATGTCAGTAGTGCTAAAGTCGTCCT
AGTTGACGGTGTCCCGGGTTGTGGAAAGACAAAAGAGATTCTCTCGAAAGTAAATTTTGAGGAAGATCTAAT
CTTAGTACCGGGTAAGCAGGCTGCTGAAATGATAAAGAGGCGTGCTAATGCGTCAGGAATAATTCAAGCCAC
AAGAGATAATGTTCGTACTGTTGATTCATTTATAATGAATTACGGTAAAGGAACACGCTGTCAGTTCAAAAGG
TTATTTATCGACGAAGGTCTGATGTTGCACACTGGTTGTGTGAATTTTCTTGTTTCTATGTCTCTGTGCGAAATT
GCATATGTTTATGGAGACACACAACAAATTCCATACATCAACAGAGTATCCGGTTTTCCGTACCCTGCACATTT
TGCAAAAATAGAGGTTGATGAGGTGGAAACTCGCAGAACTACGCTGCGTTGTCCAGCCGACATTACCCACTAT
CTTAACAGAAGGTACGAAGGACATGTCATGTGTACATCGTCGGTTAAAAAGTCAGTTTCTCAGGAAATGGTGA
GCGGGGCCGCAATGATCAATCCTGTATCTAAGCCATTGAATGGGAAAGTTTTGACTTTCACTCAGTCTGATAA
AGAGGCGCTGCTTTCTCGAGGATATACGGACGTCCATACAGTACATGAGGTACAAGGTGAGACATATGCAGA
TGTGTCGTTGGTCAGATTGACTCCGACACCTGTATCTATCATCGCAGGAGATAGTCCGCACGTTCTCGTAGCTT
TGTCAAGGCATACCCAAACATTGAAGTATTACACCGTAGTGATGGATCCTCTTGTAAGTATAATTAGGGATTTA
GAAAAACTTAGTTCTTACTTGTTAGATATGTATAAAGTAGATGCAGGGACCCAATAGCAATTACAGGTAGACT
CCGTGTTTAAAGGTTCTAATCTTTTTGTTGCAGCACCAAAGACTGGAGATATCTCAGATATGCAATTTTACTAT
GATAAGTGTCTCCCAGGTAATAGCACCATGTTAAATAACTATGATGCTGTTACCATGAGGTTGACTGACATTTC
TCTTAATGTCAAAGATTGCATATTGGATTTCTCTAAGTCTGTGGCTGCACCGAAGGATCCGATCAAACCACTGA
TTCCAATGGTACGAACGGCGGCAGAAATGCCACGCCAGACTGGACTATTGGAAAATTTGGTGGCGATGATCA
AAAGAAACTTTAATTCACCGGAGTTATCAGGAATAATCGACATTGAGAATACTGCATCTTTAGTAGTAGATAA
ATTTTTTGATAGTTACTTGCTTAAAGAAAAAAGAAAACCAAATAAAAATGTTTCTTTATTTTGTAGAGAGTCTCT
CAATAGATGGTTAGAGAAGCAGGAGCAAGTGACCATTGGTCAGCTTGCAGATTTTGATTTTGTGGATCTTCCT

GCCGTTGATCAGTACAGGCATATGATTAAAGCGCAACCTAAGCAGAAGCTGGATACATCAATTCAAAGCGAAT
ATCCGGCCTTGCAGACGATTGTGTATCATTCGAAAAAGATCAACGCAATCTTCGGTCCTTTGTTCAGTGAGCTC
ACAAGGCAAATGCTCGAAAGCATAGACTCAAGTAAGTTTTTGTTCTTTACAAGGAAGACGCCAGCTCAAATTG
AGGATTTCTTCGGAGATCTCGATAGCCATGTCCCTATGGATATCTTGGAGTTGGATATTTCGAAGTATGACAAA
TCTCAGAACGAGTTCCACTGTGCAGTAGAGTATGAAATATGGAGAAGACTTGGATTAGAAGATTTTCTGGGA
GAAGTTTGGAAACAAGGCCACAGGAAAACTACTCTTAAAGATTACACAGCTGGTATTAAAACGTGTTTATGGT
ACCAGAGAAAGAGTGGGGACGTTACAACATTCATCGGTAATACGGTGATTATTGCTGCTTGTTTAGCTTCCAT
GTTGCCCATGGAGAAAATAATCAAAGGTGCATTTTGCGGAGATGACAGTTTACTATACTTCCCAAAAGGTTGT
GAGTTTCCTGACATACAGCATACAGCCAACCTTATGTGGAATTTCGAGGCTAAGCTATTCAGAAAGCAGTATG
GTTATTTCTGTGGAAGGTACGTGATACATCATGACAGAGGGTGTATTGTTTATTATGACCCTTTGAAGTTGATT
TCTAAACTTGGTGCTAAACACATCAAGGATTGGGATCACTTAGAAGAGTTCAGAAGATCCCTTTGTGATGTTG
CAAATTCGTTGAACAACTGTGCGTATTACACGCAGTTGGACGACGCTGTGAGTGAGGTCCATAAAACCGCACC
CCCGGGTTCGTTTGTATATAAAAGTTTAGTTAAATATCTGTCCGATAAGGTTCTTTTTAGAAATTTGTTTATAGA
TGGCTCTTGTTAAGGGTAAAGTCAATATTAATGAGTTCATAGACTTGTCAAAATCAGAAAAATTTCTTCCGTCT
ATGTTCACACCTGTTAAGAGTGTCATGATCTCCAAGGTTGATAAGATATTGGTTCATGAAGATGAATCTTTGTC
CGAAGTCAATTTACTCAAAGGTGTAAAACTCATTGATGGTGGCTATGTACATCTTGCTGGTCTTGTGGTGACA
GGTGAATGGAATTTGCCAGATAATTGTCGTGGTGGTGTCAGTGTCTGTTTGGTCGATAAAAGAATGGAGAGA
GCGGACGAGGCAACTCTTGCTTCATACTATACCGCAGCGGCTAAGAAAAGGTTTCAGTTCAAAGTCGTTCCAA
ATTACAACATCACTACCAAGGACGCAAAAAAGGCAGTTTGGCAAGTACTAGTTAATATTAGAAATGTTAAAAT
TGCTGCGGGTTACTGTCCGCTGTCATTAGAATTTGTGTCAGTGTGTATTGTTTATAAAAATATTATAAAACTCG
GTTTGAGAGAGAAAATTACGAGCGTAACGGATGGAGGGCCCATGGAACTATCAGAAGAAGTTGTTGATGAG
TTCATGGAAGAAGTCCCGATGTCTGTAAGGCTTGCAAAATTTCGTTCGAAGACCGGGAAAAAGTTTAGTAGTA
AAAGTGAGAATAATAGTGGTAATAATAGGCCGAAACCAAACAAAAACCAAAGGAAGGAAAAGGGTTTAAAA
GTTAGGGTTGAGAAGGATAATTTAATTGATAATGAATTGGAGACTTACGTCGCCGATTCAGATTCGTATTAAA
TATGTCTTACACAATCGCAACTCCATCGCAATTTGTGTTTTTGTCATCAGCATGGGCCGACCCTATAGAATTAAT
AAATTTATGTACTAATTCACTAGGTAATCAATTCCAAACACAACAAGCTAGAACAACCGTTCAACGGCAATTTA
GCGAAGTGTGGAAACCTGTCCCTCAAGTCACTGTTAGGTTTCCTGACAGTGGTTTTAAGGTGTATAGGTACAA
TGCGGTACTAGATCCTCTAGTTACTGCTTTGTTAGGAGCTTTCGATACTAGAAATAGGATTATAGAAGTCGAAA
ATCAGGCGAACCCGACAACCGCCGAAACGTTAGACGCTACTCGTAGAGTAGATGACGCAACGGTGGCTATAA
GGAGCGCTATAAATAATTTAGTAGTAGAATTGGTCAAAGGAACAGGTTTGTACAATCAGAGCACATTTGAAA
GTGCATCCGGTTTACAATGGTCCTCTGCACCTGCATCTTGA

#MW314123

1

ATGGCATACACACACAGACAGCTACCACATCCGCTTTGCTCGACACTGTCCGAGGTAACAATACCTTGGTCAATG
ATCTTGCGAAGCGGCGTCTTTATGACACAGCGGTCGACGAGTTCAACGCTCGTGATCGCAGGCCCAAAGTAA
ATTTTTCCAAAGTAATAAGTGAGGAACAGACGCTTATTGCTACTAGGGCATATCCAGAATTCCAGATAACCTTC
TATAATACGCAGAACGCCGTGCATTCGCTTGCCGGTGGACTACGATCCTTAGAACTGGAATATCTAATGATGC
AGATCCCGTACGGATCACTCACATATGATATAGGTGGGAATTTTGCATCACATCTGTTCAAAGGACGGGCATA
TGTTCACTGCTGTATGCCCAATCTTGATGTCCGCGACATAATGCGGCACGAAGGCCAGAAAGACAGTATAGAA
TTATACCTTTCCAGGCTTGAGCGGGGCAACAAAGTTGTCCCAAATTTCCAAAAGGAAGCTTTTGACAGATACG
CTGAAACGCCAGACGAAGTTGTCTGTCACAGTACCTTCCAAACGTGTACGCACCAGCAGGTGGAAAACACAG
GCAGGGTGTATGCTATTGCATTGCACAGTATATACGATATACCTGCTGATGAATTCGGAGCGGCACTTTTAAG
GAAAAATGTCCATGTTTGTTACGCCGCCTTCCACTTTTCCGAGAATTTACTTCTCGAAGATTCACACGTCAACCT

TGACGAAATCAACGCGTGTTTTTCGCGTGATGGAGACAAGCTGACTTTTTCTTTCGCATCTGAGAGCACTTTAA
ATTATTGTCATAGTTATTCTAATATTTTAAAATACGTGTGCAAAACTTACTTCCCGGCATCTAATAGAGAGGTCT
ACATGAAGGAGTTTTTAGTCACCAGGGTTAACACCTGGTTTTGTAAGTTTTCTAGGATTGATACTTTTTTATTAT
ACAAGGGGGTAGCCCACAAAGGTGTAAATAGTGAGCAATTTTACAGCGCAATGGAAGATGCATGGCACTACA
AAAAGACTCTTGCAATGTGTAACAGCGAGAGGATTCTTCTTGAAGATTCCTCATCGGTCAATTACTGGTTCCCA
AAAATGAGAGATATGGTCATAGTTCCTCTATTCGACATATCTCTCGACACCAGTAAAAGGACCCGCAAAGAAG
TCTTAGTGTCAAAGGATTTTGTATTCACAGTTTTAAATCACATTCGCACTTATCAAGCCAAGGCACTTACATACT
CCAATGTTTTATCCTTTGTCGAATCAATTCGTTCAAGGGTAATTATCAACGGAGTGACTGCTAGGTCTGAGTGG
GATGTTGACAAATCTCTTTTGCAATCCTTGTCCATGACATTTTTCTTGCATACTAAGCTTGCCGTTTTAAAAGAC
GAATTGTTAATCAGCAAGTTTAGTTTGGGGCCAAAATCAGTAAGCCAGCATGTATGGGATGAGATTTCCCTGG
CTTTTGGAAACGCATTTCCATCGATCAAGGAGAGACTGCTAAATCGGAAACTAATTAAAGTGTCGGGAGACGC
ATTAGAAATCAGGGTGCCTGATTTATATGTGACTTTTCACGATAGATTAGTGACTGAGTACAAAACATCGGTG
GATATGCCAGTGCTTGATATCAGAAAGAGAATGGAGGAGACTGAGGTTATGTACAATGCATTGTCTGAGCTA
TCTGTGCTCAAGGAGTCGGACAAGTTCGACGTTGATGTTTTTTCCCGGATGTGCCAGACTTTGGAGGTAGACC
CAATGACTGCAGCAAAGGTTATAGTGGCAGTGATGAGCAACGAGAGCGGACTGACTCTTACATTCGAACAGC
CAACTGAAGCAAATGTCGCATTGGCACTTAAAGATTCAGAAAAAGCCTCTGAGGGTGCACTAGTGGTTACTTC
TAGAGATGTTGAAGAACCATCCATGAAGGGTTCAATGGCAAGAGGAGAGTTACAATTGGCCGGTCTGTCTGG
AGACCAACCAGAGTCTTCCTATACTCGGAACGAGGAAATAGAGTCATTAGAGCAATTCCACATGGCAACGGCT
AGTTCGTTAATTCGGAAACAGATGAGTTCGATTGTGTACACGGGCCCCATTAAAGTTCAGCAAATGAAAAACT
TTATTGATAGCCTGGTAGCATCACTCTCTGCTGCGGTGTCGAACCTAGTCAAGATCCTAAAGGATACAGCTGCT
ATAGATCTCGAAACCCGTCAGAAGTTTGGAGTCTTAGATGTTGCGACCAAAAGATGGTTAATTAAACCTTTAG
CCAAAAATCACGCATGGGGCGTTATTGAAACACATGCTAGGAAGTACCACGTTGCACTTTTGGAGTATGATGA
GCATGGAGTGGTAACTTGCGACAGTTGGAGAAGGGTGGCCGTGAGTTCTGAGTCAATGGTTTATTCTGATAT
GGCAAAGCTCAGAACACTGAGGAGATTATTAAGAGATGGTGAGCCTCATGTCAGCAGTGCTAAAGTCGTCCT
AGTTGACGGTGTCCCGGGTTGTGGAAAGACAAAAGAGATTCTCTCGAAAGTAAATTTTGAGGAAGATCTAAT
CTTAGTACCGGGTAAGCAGGCTGCTGAAATGATAAAGAGGCGTGCTAATGCGTCAGGAATAATTCAAGCCAC
AAGAGATAATGTTCGTACTGTTGATTCATTTATAATGAATTACGGTAAAGGAACACGCTGTCAGTTCAAAAGG
TTATTTATCGACGAAGGTCTGATGTTGCACACTGGTTGTGTGAATTTTCTTGTTTCTATGTCTCTGTGCGAAATT
GCATATGTATATGGAGACACACAACAAATTCCATACATCAACAGAGTATCCGGTTTTCCGTACCCTGCACATTT
TGCAAAAATAGAGGTTGATGAGGTGGAAACTCGCAGAACTACGCTGCGTTGTCCAGCCGACATTACCCACTAT
CTTAACAGAAGGTACGAAGGACATGTCATGTGTACATCGTCGGTTAAAAAGTCAGTTTCTCAGGAAATGGTGA
GCGGGGCCGCAATGATCAATCCTGTATCTAAGCCATTGAATGGGAAAGTTTTGACTTTCACTCAGTCCGATAA
AGAGGCGCTGCTTTCTCGAGGATATACGGACGTCCATACAGTACATGAGGTACAAGGTGAGACATATGCAGA
TGTGTCGTTGGTCAGATTGACTCCGACACCTGTATCTATCATCGCAGGAGATAGTCCGCACGTTCTCGTAGCTT
TGTCAAGGCATACCCAAACATTGAAGTATTACACCGTAGTGATGGATCCTCTTGTAAGTATAATTAGGGATTTA
GAAAAACTTAGTTCTTACTTGTTAGATATGTATAAAGTAGATGCAGGGACCCAATAGCAATTACTGGTAGACT
CCGTGTTTAAAGGTTCTAATCTTTTTGTTGCAGCACCAAAGACTGGAGATATCTCAGATATGCAATTTTACTAT
GATAAGTGTCTCCCAGGTAATAGCACCATGTTAAATAACTATGATGCTGTTACCATGAGGTTGACTGACATTTC
TCTTAATGTCAAAGATTGCATATTGGATTTCTCTAAGTCTGTGGCTGCACCGAAGGATCCGATCAAACCACTGA
TTCCAATGGTACGAACGGCGGCAGAAATGCCACGCCAGACTGGACTATTGGAAAATTTGGTGGCGATGATCA
AAAGAAACTTTAATTCACCGGAGTTATCAGGAATAATCGACATTGAGAATACTGCATCTTTGGTAGTAGATAA
ATTTTTTGATAGTTACTTGCTTAAAGAAAAAAGAAAACCAAATAAAAATGTTTCTTTATTTTGTAGAGAGTCTCT
CAATAGATGGTTAGAGAAGCAGGAGCAAGTGACCATTGGTCAGCTTGCAGATTTTGATTTTGTGGATCTTCCT
GCCGTTGATCAGTACAGGCATATGATTAAAGCGCAACCTAAGCAGAAGCTGGATACATCAATTCAAAGCGAAT
ATCCGGCCTTGCAGACGATTGTGTATCATTCAAAAAAGATCAACGCAATCTTCGGTCCTTTGTTCAGTGAGCTC
ACAAGGCAAATGCTCGAAAGCATAGACTCAAGTAAGTTTTTGTTCTTTACAAGGAAGACGCCAGCTCAAATCG
AGGATTTCTTCGGAGATCTCGATAGCCATGTCCCTATGGATATCTTGGAGTTGGATATTTCGAAGTATGACAAA
TCTCAGAACGAGTTCCACTGTGCAGTAGAGTATGAAATATGGAGAAGACTTGGATTAGAAGATTTTCTGGGA

GAAGTTTGGAAACAAGGCCACAGAAAAACTACTCTTAAAGATTACACAGCTGGTATTAAAACGTGTTTATGGT
ACCAGAGAAAGAGTGGGGACGTTACAACATTCATCGGTAATACGGTGATTATTGCTGCTTGTTTAGCTTCCAT
GTTGCCCATGGAGAAAATAATCAAAGGTGCATTTTGCGGAGATGACAGTTTACTATACTTCCCAAAAGGTTGT
GAGTTTCCCGACATACAGCATACAGCTAACCTTATGTGGAATTTCGAGGCTAAGCTATTCAGAAAGCAGTATG
GTTATTTCTGTGGAAGGTACGTGATACATCATGACAGAGGGTGTATTGTTTATTACGACCCTTTGAAGTTGATT
TCTAAACTTGGTGCTAAACACATCAAGGATTGGGATCACTTAGAAGAGTTCAGAAGATCCCTTTGTGATGTTG
CAATTTCGTTGAACAACTGTGCGTATTACACGCAGTTGGACGACGCTGTGAGTGAGGTCCATAAAACCGCACC
CCCGGGTTCGTTTGTTTATAAAAGTTTAGTTAAATATCTGTCCGATAAGGTTCTTTTTAGAAGTTTGTTTATAGA
TGGCTCTTGTAAAGGGTAAAGTCAATATTAATGAGTTCATAGACTTGTCAAAATCAGAAAAATTTTTCCCGTCT
ATGTTCACACCTGTTAAGAGTGTCATGATCTCCAAGGTTGATAAGATATTGGTTCATGAAGATGAATCTTTGTC
CGAAGTCAATTTACTCAAAGGTGTAAAACTCATTAATGGTGGCTATGTACATCTTGCTGGTCTTGTAGTGACAG
GTGAATGGAATTTGCCAGATAATTGTCGTGGTGGTGTCAGTGTCTGTTTGGTCGATAAGAGAATGGAGAGAG
CGGACGAGGCAACTCTTGCTTCATACTATACCGCAGCGGCTAAGAAAAGGTTTCAGTTCAAAGTCGTTCCAAA
TTACAACATCACTACCAAGGACGCAGAAAAGGCAGTTTGGCAAGTACTAGTTAATATTAGAAATGTTAAAATT
GCTGCGGGTTACTGTCCGCTGTCATTAGAATTTGTGTCAGTGTGTATTGTTTATAAAAATATTATAAAACTCGG
TTTGAGAGAGAAAATTACGAGCGTCACGGAAGGAGGGCCCATGGAACTATCAGAAGAAGTTGTTGATGAGTT
CATGGAAGAAGTCCCGATGTCTGTAAGGCTTGCAAAATTTCGTTCGAAGACCGGGAAAAAGTTTAGTAGTAA
AAGTGAGAATAATAGTGGTAATAATAGGCCGAAACCAGACAAAAACCAAAGGAAGGAAAAGGGTTTAAAAG
TTAGGGTTGAGAAGGATAATTTAATTGATAATGAATTGGAGACTTACGTCGCCGATTCAGATTCGTATTAAAT
ATGTCTTACACAATCGCAACTCCATCGCAATTTGTGTTTTTGTCATCAGCATGGGCCGACCCTATAGAATTAATA
AATTTATGTACTAATTCACTAGGCAATCAGTTCCAAACACAACAAGCTAGAACAACCGTTCAACGGCAATTTAG
CGAAGTGTGGAAACCTGTCCCTCAAGTCACTGTTAGGTTTCCTGACAGTGGTTTTAAGGTGTATAGGTACAAT
GCGGTACTAGATCCTCTAGTTACTGCTTTGTTGGGAGCTTTCGATACTAGAAATAGGATTATAGAAGTCGAAA
ATCAGGCGAACCCGACAACCGCCGAAACGTTAGACGCTACTCGTAGAGTAGATGACGCAACGGTGGCTATAA
GGAGCGCTATAAATAATTTAGTAGTAGAATTGGTCAAAGGAACAGGTTTGTACAATCAGAGCACATTTGAAA
GTGCATCCGGTTTACAATGGTCCTCTGCACCTGCATCTTGA

#MW314132

1

ATGGCATACACACAGACAGCTACCACATCCGCTTTGCTCGACACTGTCCGAGGTAACAATACCTTGGTCAACG
ATCTTGCGAAGCGGCGTCTTTATGACACAGCGGTCGACGAGTTCAACGCTCGTGATCGCAGGCCCAAAGTAA
ATTTTTCCAAAGTAATAAGTGAGGAACAGACGCTTATTGCTACTAGGGCATATCCTGAATTCCAGATAACCTTC
TATAATACGCAGAACGCCGTGCATTCGCTTGCCGGTGGACTACGATCCTTAGAACTGGAATATCTAATGATGC
AGATCCCGTACGGATCACTCACATATGATATAGGTGGGAATTTTGCATCTCATCTGTTCAAGGGACGGGCATA
TGTTCACTGCTGTATGCCCAATCTTGATGTCCGCGACATAATGCGGCACGAAGGCCAGAAAGACAGTATAGAA
TTATACCTTTCCAGGCTTGAGCGGGGCAACAAAGTTGTCCCAAATTTCCAAAAGGAAGCTTTTGACAGATACG
CTGAAACGCCAGACGAAGTTGTCTGTCACAGTACCTTCCAAACGTGTACGCACCAGCAGGTGGAAAACACAG
GCAGGGTGTATGCTATTGCATTGCACAGTATATACGATATACCTGCTGATGAATTCGGAGCGGCACTTTTAAG
GAAAAATGTCCATGTTTGTTACGCCGCCTTCCACTTTTCCGAGAATTTACTTCTCGAAGATTCACACGTCAACCT
TGACGAAATCAACGCGTGTTTTTCGCGTGATGGAGACAAGCTGACTTTTTCTTTCGCATCTGAGAGCACTTTAA
ATTATTGTCATAGTTATTCTAATATTTTAAAATACGTGTGCAAAACTTACTTCCCGGCATCTAATAGAGAGGTCT
ACATGAAGGAGTTTTTGGTCACCAGGGTTAACACCTGGTTTTGTAAGTTTTCTAGGATAGATACTTTTTTATTAT
ACAAGGGGGTAGCCCACAAAGGTGTAAATAGTGAGCAATTTTACAGCGCAATGGAAGATGCATGGCACTACA
AAAAGACTCTTGCAATGTGTAACAGCGAGAGGATTCTTCTTGAAGATTCCTCATCGGTCAATTACTGGTTCCCA

AAAATGAGAGATATGGTCATAGTTCCTCTATTCGACATATCTCTCGACACTAGTAAAAGGACCCGCAAAGAAG
TCTTAGTGTCAAAGGATTTTGTATTCACAGTTTTAAATCACATTCGCACTTATCAAGCCAAGGCACTTACATACT
CCAATGTTTTATCCTTTGTCGAATCAATTCGTTCAAGGGTAATTATCAACGGAGTGACTGCCAGGTCTGAGTGG
GATGTTGACAAATCTCTTTTGCAATCCTTGTCCATGACATTTTTCTTGCATACTAAGCTTGCCGTTTTAAAAGAC
GAATTGTTAATCAGCAAGTTTAGTTTGGGGCCAAAATCAGTAAGCCAGCATGTATGGGATGAGATTTCCCTGG
CTTTTGGAAACGCATTTCCATCGATCAAGGAGAGACTGCTAAATCGGAAACTAATTAAAGTGTCGGGAGACGC
ATTAGAAATCAGGGTGCCTGATTTATATGTGACTTTTCACGATAGATTAGTGACTGAGTACAAAACATCGGTG
GATATGCCAGTGCTTGATATCAGAAAGAGAATGGAGGAGACTGAGGTTATGTACAATGCATTGTCTGAGCTA
TCTGTGCTCAAGGAGTCGGACAAGTTCGACGTTGATGTTTTTTCCCGGATGTGCCAGACTTTGGAGGTAGACC
CAATGACTGCAGCAAAGGTTATTGTGGCAGTGATGAGCAACGAGAGCGGACTGACTCTTACATTCGAACAGC
CAACTGAAGCAAATGTCGCATTGGCACTTAAAGATTCAGAAAAAGCCTCTGAGGGTGCACTAGTGGTTACTTC
TAGAGATGTTGAAGAACCATCCATGAAGGGTTCAATGGCAAGAGGAGAGTTACAATTGGCCGGTCTGTCTGG
AGACCAACCAGAGTCTTCCTATACTCGGAACGAGGAAATAGAGTCATTAGAGCAATTCCACATGGCAACGGCT
GGTTCGTTAATTCGGAAACAGATGAGTTCGATTGTGTACACGGGCCCCATTAAAGTTCAGCAAATGAAAAACT
TTATTGATAGCCTGGTAGCATCACTCTCTGCTGCGGTGTCGAACCTAGTCAAGATCCTAAAGGATACAGCTGCT
ATAGATCTCGAAACCCGTCAGAAGTTTGGAGTCTTAGATGTTGCGACCAAAAGATGGTTAATTAAACCTTTAG
CCAAGAATCACGCATGGGGCGTTATTGAAACACATGCTAGGAAGTACCACGTTGCACTTTTGGAGTATGATGA
GCATGGAGTGGTAACTTGCGACAGTTGGAGAAGGGTGGCCGTGAGTTCTGAGTCAATGGTTTATTCTGATAT
GGCAAAGCTCAGAACACTGAGGAGATTGTTAAGAGATGGTGAGCCTCATGTCAGCAGTGCTAAAGTCGTCCT
AGTTGACGGTGTCCCGGGTTGTGGAAAGACAAAAGAGATTCTCTCGAAAGTAAATTTTGAGGAAGATCTAAT
CTTAGTACCGGGTAAGCAGGCTGCTGAAATGATAAAGAGGCGTGCTAATGCGTCAGGAATAATTCAAGCCAC
AAGAGATAATGTTCGTACTGTTGATTCATTTATAATGAATTACGGTAAAGGAACACGCTGTCAGTTCAAAAGG
TTATTTATCGACGAAGGTCTGATGTTGCACACTGGTTGTGTGAATTTTCTTGTTTCTATGTCTCTGTGCGAAATT
GCATATGTTTATGGAGACACACAACAAATTCCATACATCAACAGAGTATCCGGTTTTCCGTACCCTGCACATTT
TGCAAAAATAGAGGTTGATGAGGTGGAAACTCGCAGAACTACGCTGCGTTGTCCAGCCGACATTACCCACTAT
CTTAACAGAAGGTACGAAGGACATGTCATGTGTACATCGTCGGTTAAAAAGTCAGTTTCTCAGGAAATGGTGA
GCGGGGCCGCAATGATCAATCCTGTATCTAAGCCATTGAATGGGAAAGTTTTGACTTTCACTCAGTCTGATAA
AGAGGCGCTGCTTTCTCGAGGATATACGGACGTCCATACAGTACATGAGGTACAAGGTGAGACATATGCAGA
TGTGTCGTTGGTCAGATTGACTCCGACACCTGTATCTATCATCGCAGGAGATAGTCCGCACGTTCTCGTAGCTT
TGTCAAGGCATACCCAAACATTGAAGTATTACACCGTAGTGATGGATCCTCTTGTAAGTATAATTAGGGATTTA
GAAAAACTTAGTTCTTACTTGTTAGATATGTATAAAGTAGATGCAGGGACCCAATAGCAATTACAGGTAGACT
CCGTGTTTAAAGGTTCTAATCTTTTTGTTGCAGCACCAAAGACTGGAGATATCTCAGATATGCAATTTTACTAT
GATAAGTGTCTCCCAGGTAATAGCACCATGTTAAATAACTATGATGCTGTTACCATGAGGTTGACTGACATTTC
TCTTAATGTCAAAGATTGCATATTGGATTTCTCTAAGTCTGTGGCTGCACCGAAGGATCCGATCAAACCACTGA
TTCCAATGGTACGAACGGCGGCAGAAATGCCACGCCAGACTGGACTATTGGAAAATTTGGTGGCGATGATCA
AAAGAAACTTTAATTCACCGGAGTTATCAGGAATAATCGACATTGAGAATACTGCATCTTTAGTAGTAGATAA
ATTTTTTGATAGTTACTTGCTTAAAGAAAAAAGAAAACCAAATAAAAATGTTTCTTTATTTTGTAGAGAGTCTCT
CAATAGATGGTTAGAGAAGCAGGAGCAAGTGACCATTGGTCAGCTTGCAGATTTTGATTTTGTGGATCTTCCT
GCCGTTGATCAGTACAGGCATATGATTAAAGCGCAACCTAAGCAGAAGCTGGATACATCAATTCAAAGCGAAT
ATCCGGCCTTGCAGACGATTGTGTATCATTCGAAAAAGATCAACGCAATCTTCGGTCCTTTGTTCAGTGAGCTC
ACAAGGCAAATGCTCGAAAGCATAGACTCAAGTAAGTTTTTGTTCTTTACAAGGAAGACGCCAGCTCAAATTG
AGGATTTCTTCGGAGATCTCGATAGCCATGTCCCTATGGATATCTTGGAGTTGGATATTTCGAAGTATGACAAA
TCTCAGAACGAGTTCCACTGTGCAGTAGAGTATGAAATATGGAGAAGACTTGGATTAGAAGATTTTCTGGGA
GAAGTTTGGAAACAAGGCCACAGGAAAACTACTCTTAAAGATTACACAGCTGGTATTAAAACGTGTTTATGGT
ACCAGAGAAAGAGTGGGGACGTTACAACATTCATCGGTAATACGGTGATTATTGCTGCTTGTTTAGCTTCCAT
GTTGCCCATGGAGAAAATAATCAAAGGTGCATTTTGCGGAGATGACAGTTTACTATACTTCCCAAAAGGTTGT
GAGTTTCCTGACATACAGCATACAGCCAACCTTATGTGGAATTTCGAGGCTAAGCTATTCAGAAAGCAGTATG
GTTATTTCTGTGGAAGGTACGTGATACATCATGACAGAGGGTGTATTGTTTATTATGACCCTTTGAAGTTGATT

TCTAAACTTGGTGCTAAACACATCAAGGATTGGGATCACTTAGAAGAGTTCAGAAGATCCCTTTGTGATGTTG
CAAATTCGTTGAACAACTGTGCGTATTACACGCAGTTGGACGACGCTGTGAGTGAGGTCCATAAAACCGCACC
CCCGGGTTCGTTTGTATATAAAAGTTTAGTTAAATATCTGTCCGATAAGGTTCTTTTTAGAAGTTTGTTTATAGA
TGGCTCTTGTTAAGGGTAAAGTCAATATTAATGAGTTCATAGACTTGTCAAAATCAGAAAAATTTCTTCCGTCT
ATGTTCACACCTGTTAAGAGTGTCATGATCTCCAAGGTTGATAAGATATTGGTTCATGAAGATGAATCTTTGTC
CGAAGTCAATTTACTCAAAGGTGTAAAACTCATTGATGGTGGCTATGTACATCTTGCTGGTCTTGTGGTGACA
GGTGAATGGAATTTGCCAGATAATTGTCGTGGTGGTGTCAGTGTCTGTTTGGTCGATAAAAGAATGGAGAGA
GCGGACGAGGCAACTCTTGCTTCATACTATACCGCAGCGGCTAAGAAAAGGTTTCAGTTCAAAGTCGTTCCAA
ATTACAACATCACTACCAAGGACGCAAAAAAGGCAGTTTGGCAAGTACTAGTTAATATTAGAAATGTTAAAAT
TGCTGCGGGTTACTGTCCGCTGTCATTAGAATTTGTGTCAGTGTGTATTGTTTATAAAAATATTATAAAACTCG
GTTTGAGAGAGAAAATTACGAGCGTAACGGATGGAGGGCCCATGGAACTATCAGAAGAAGTTGTTGATGAG
TTCATGGAAGAAGTCCCGATGTCTGTAAGGCTTGCAAAATTTCGTTCGAAGACCGGGAAAAAGTTTAGTAGTA
AAAGTGAGAATAATAGTGGTAATAATAGGCCGAAACCAAACAAAAACCAAAGGAAGGAAAAGGGTTTAAAA
GTTAGGGTTGAGAAGGATAATTTAATTGATAATGAATTGGAGACTTACGTCGCCGATTCAGATTCGTATTAAA
TATGTCTTACACAATCGCAACTCCATCGCAATTTGTGTTTTTGTCATCAGCATGGGCCGACCCTATAGAATTAAT
AAATTTATGTACTAATTCACTAGGTAATCAATTCCAAACACAACAAGCTAGAACAACCGTTCAACGGCAATTTA
GCGAAGTGTGGAAACCTGTCCCTCAAGTCACTGTTAGGTTTCCTGACAGTGGTTTTAAGGTGTATAGGTACAA
TGCGGTACTAGATCCTCTAGTTACTGCTTTGTTAGGAGCTTTCGATACTAGAAATAGGATTATAGAAGTCGAAA
ATCAGGCGAACCCGACAACCGCCGAAACGTTAGACGCTACTCGTAGAGTAGATGACGCAACGGTGGCTATAA
GGAGCGCTATAAATAATTTAGTAGTAGAATTGGTCAAAGGAACAGGTTTGTACAATCAGAGCACATTTGAAA
GTGCATCCGGTTTACAATGGTCCTCTGCACCTGCATCTTGA

#MW314136

1

ATGGCATACACACAGACAGCTACCACATCCGCTTTGCTCGACACTGTCCGAGGTAACAATACCTTGGTCAACG
ATCTTGCGAAGCGGCGTCTTTATGACACAGCGGTCGACGAGTTCAACGCTCGTGATCGCAGGCCCAAAGTAA
ATTTTTCCAAAGTAATAAGTGAGGAACAGACGCTTATTGCTACTAGGGCATATCCAGAATTCCAGATAACCTTT
TATAATACGCAGAACGCCGTGCATTCGCTTGCCGGTGGACTACGATCCTTAGAACTGGAATATCTAATGATGC
AGATCCCGTACGGATCACTCACATATGATATAGGTGGGAATTTTGCATCTCATCTGTTCAAAGGACGGGCATA
TGTTCACTGCTGTATGCCCAATCTTGATGTCCGCGACATAATGCGGCACGAAGGCCAGAAAGACAGTATAGAA
TTATACCTTTCCAGGCTTGAGCGGGGCAACAAAGTTGTCCCAAATTTCCAAAAGGAAGCTTTTGACAGATACG
CTGAAACGCCAGACGAAGTTGTCTGTCACAGTACCTTCCAAACGTGTACGCACCAGCAGGTGGAAAACACAG
GCAGGGTGTATGCTATTGCACTGCACAGTATATACGATATACCTGCTGATGAATTCGGAGCGGCACTTTTAAG
GAAAAATGTCCATGTTTGTTACGCCGCCTTCCACTTTTCCGAGAATTTACTTCTCGAAGATTCACACGTCAACCT
TGACGAAATCAATGCGTGTTTTTCGCGTGATGGAGACAAGCTGACTTTTTCTTTCGCATCTGAGAGCACTTTAA
ATTATTGTCATAGTTATTCTAATATTTTAAAATACGTGTGCAAAACTTACTTCCCGGCATCTAATAGAGAGGTCT
ACATGAAGGAGTTTTTGGTCACCAGGGTTAACACCTGGTTTTGTAAGTTTTCTAGGATAGATACTTTTTTATTAT
ACAAGGGGGTAGCCTACAAAGGTGTAAATAGTGAGCAATTTTACAGCGCAATGGAAGATGCATGGCACTACA
AAAAGACTCTTGCAATGTGTAACAGCGAGAGGATTCTTCTTGAAGATTCCTCATCGGTCAATTACTGGTTCCCA
AAAATGAGAGATATGGTCATAGTTCCTCTATTCGACATATCTCTCGACACCAGTAAAAGGACCCGCAAAGAAG
TCTTAGTGTCAAAGGATTTTGTATTCACAGTTTTAAATCACATTCGCACTTATCAAGCCAAGGCACTTACATACT
CCAATGTTTTATCCTTTGTCGAATCAATTCGTTCAAGGGTAATTATCAACGGAGTGACTGCCAGGTCTGAGTGG
GATGTTGACAAATCTCTTTTGCAATCCTTGTCCATGACATTTTTCTTGCATACTAAGCTTGCCGTTTTAAAAGAC
GAATTGTTAATCAGCAAGTTTAGTTTGGGGCCAAAATCAGTAAGCCAGCATGTATGGGATGAGATTTCCCTGG

CTTTTGGAAACGCATTTCCATCGATCAAGGAGAGACTGCTAAATCGGAAACTAATTAAAGTGTCGGGAGACGC
ATTAGAAATCAGGGTGCCTGATTTATATGTGACTTTTCACGATAGATTAGTGGCTGAGTACAAAACATCGGTG
GATATGCCAGTGCTTGATATCAGAAAGAGAATGGAGGAGACTGAGGTTATGTACAATGCATTGTCTGAGCTA
TCTGTGCTCAAGGAGTCGGACAAGTTCGACGTTGATGTTTTTTCCCGGATGTGCCAGACTTTGGAGGTAGACC
CAATGACTGCAGCAAAGGTTATTGTGGCAGTGATGAGCAACGAGAGCGGACTGACTCTTACATTCGAACAGC
CAACTGAAGCAAATGTCGCATTGGCACTTAAAGATTCAGAAAAAGCCTCTGAGGGTGCACTAGTGGTTACTTC
TAGAGATGTTGAAGAACCATCCATGAAGGGTTCAATGGCAAGAGGAGAGTTACAATTGGCCGGTCTGTCTGG
AGACCAACCAGAGTCTTCCTATACTCGGAACGAGGAAATAGAGTCATTAGAGCAATTCCACATGGCAACGGCT
AGTTCGTTAATTCGGAAACAGATGAGTTCGATTGTGTACACGGGCCCCATTAAAGTTCAGCAAATGAAAAACT
TTATTGATAGCCTGGTAGCATCACTCTCTGCTGCGGTGTCGAACCTAGTCAAGATCCTAAAGGATACAGCTGCT
ATAGATCTCGAAACCCGTCAGAAGTTTGGAGTCTTAGATGTTGCGACCAAAAGATGGTTAATTAAACCTTTAG
CCAAGAATCACGCATGGGGCGTTATTGAAACACATGCTAGGAAGTACCACGTTGCACTTTTGGAGTATGATGA
GCATGGAGTGGTAACTTGCGACAGTTGGAGAAGGGTGGCCGTGAGTTCTGAGTCAATGGTTTATTCTGATAT
GGCAAAGCTCAGAACACTGAGGAGATTATTAAGAGATGGTGAGCCTCATGTCAGCAGTGCTAAAGTCGTCCT
AGTTGACGGTGTCCCGGGTTGTGGAAAGACAAAAGAGATTCTCTCGAAAGTAAATTTTGAGGAAGATCTAAT
CTTAGTACCGGGTAAGCAGGCTGCTGAAATGATAAAGAGGCGTGCTAATGCGTCAGGAATAATTCAAGCCAC
AAGAGATAATGTTCGCACTGTTGATTCATTTATAATGAATTACGGTAAAGGAACACGCTGTCAGTTCAAAAGG
TTATTTATCGACGAAGGTCTGATGTTGCACACTGGTTGTGTGAATTTTCTTGTTTCTATGTCTCTGTGCGAAATT
GCATATGTTTATGGAGACACACAACAAATTCCATACATCAACAGAGTATCCGGTTTTCCGTACCCTGCACATTT
TGCAAAAATAGAGGTTGATGAGGTGGAAACTCGCAGAACTACGCTGCGTTGTCCAGCCGACATTACCCACTAT
CTTAACAGAAGGTACGAAGGACATGTCATGTGTACATCGTCGGTTAAAAAGTCAGTTTCTCAGGAAATGGTGA
GCGGGGCCGCAATGATCAATCCTGTATCTAAGCCATTGAATGGGAAAGTTTTGACTTTCACTCAGTCTGATAA
AGAGGCGCTGCTTTCTCGAGGATATACGGACGTCCATACAGTACATGAGGTACAAGGTGAGACATATGCAGA
TGTGTCGTTGGTCAGATTGACTCCGACACCTGTATCTATCATCGCAGGAGATAGTCCGCACGTTCTCGTAGCTT
TGTCAAGGCATACCCAAACATTGAAGTATTACACCGTAGTGATGGATCCTCTTGTAAGTATAATTAGGGATTTA
GAAAAACTTAGTTCTTACTTGTTAGATATGTATAAAGTAGATGCAGGGACCCAATAGCAATTACAGGTAGACT
CCGTGTTTAAAGGTTCTAATCTTTTTGTTGCAGCACCAAAGACTGGAGATATCTCAGATATGCAATTTTACTAT
GATAAGTGTCTCCCAGGTAATAGCACCATGTTAAATAACTATGATGCTGTTACCATGAGGTTGACTGACATTTC
TCTTAATGTCAAAGATTGCATATTGGATTTCTCTAAGTCTGTGGCTGCACCGAAGGATCCGATCAAACCACTGA
TTCCAATGGTACGAACGGCGGCAGAAAAGCCACGCCAGACTGGACTATTGGAAAATTTGGTGGCGATGATCA
AAAGAAACTTTAATTCACCGGAGTTATCAGGAATAATCGACATTGAGAATACTGCATCTTTAGTAGTAGATAA
ATTTTTTGATAGTTACTTGCTTAAAGAAAAAAGAAAACCAAATAAAAATGTTTCTTTATTTTGTAGAGAGTCTCT
CAATAGATGGTTAGAGAAGCAGGAGCAAGTGACCATTGGTCAGCTTGCAGATTTTGATTTTGTGGATCTTCCT
GCCGTTGATCAGTACAGGCATATGATTAAAGCGCAACCTAAGCAGAAGCTGGATACATCAATTCAAAGCGAAT
ATCCGGCCTTGCAGACGATTGTGTATCATTCGAAAAAGATCAACGCAATCTTCGGTCCTTTGTTCAGTGAGCTC
ACAAGGCAAATGCTCGAAAGCATAGACTCAAGTAAGTTTTTGTTCTTTACAAGGAAGGCGCCAGCTCAAATTG
AGGATTTCTTCGGAGATCTCGATAGCCATGTCCCTATGGATATCTTGGAGTTGGATATTTCGAAGTATGACAAA
TCTCAGAACGAGTTCCACTGTGCAGTAGAGTATGAAATATGGAGAAGACTTGGATTAGAAGATTTTCTGGGA
GAAGTTTGGAAACAAGGCCACAGAAAAACTACTCTTAAAGATTACACAGCTGGTATTAAAACGTGTTTATGGT
ACCAGAGAAAGAGTGGGGACGTTACAACATTCATCGGTAATACGGTGATTATTGCTGCTTGTTTAGCATCCAT
GTTGCCCATGGAGAAAATAATCAAAGGTGCATTTTGCGGAGATGACAGTTTACTATACTTCCCAAAAGGTTGT
GAGTTTCCTGACATACAGCATACAGCCAACCTTATGTGGAATTTCGAGGCTAAGCTATTCAGAAAGCAGTATG
GTTATTTCTGTGGAAGGTACGTGATACATCACGACAGAGGGTGTATTGTTTATTATGACCCTTTGAAGTTGATT
TCTAAACTTGGTGCTAAACACATCAAGGATTGGGATCACTTAGAAGAGTTCAGAAGATCCCTTTGTGATGTTG
CAAATTCGTTGAACAACTGTGCGTATTACACGCAGTTGGACGACGCTGTGAGTGAGGTCCATAAAACCGCACC
CCCGGGTTCGTTTGTATATAAAAGTTTAGTTAAATATCTGTCCGATAAGGTTCTTTTTAGAAGTTTGTTTATAGA
TGGCTCTTGTTAAGGGTAAAGTCAATATTAATGAGTTCATAGACTTGTCAAAATCAGAAAAATTTCTTCCGTCT
ATGTTCACACCTGTTAAGAGTGTCATGATCTCCAAGGTTGATAAGATATTGGTTCATGAAGATGAATCTTTGTC

CGAAGTCAATTTACTCAAAGGTGTAAAACTCATTGATGGTGGCTATGTACATCTTGCTGGTCTTGTGGTGACA
GGTGAATGGAATTTGCCAGATAATTGTCGTGGTGGTGTCAGTGTCTGTTTGGTCGATAAGAGAATGGAGAGA
GCGGACGAGGCAACTCTTGCTTCATACTATACCGCAGCGGCTAAGAAAAGGTTTCAGTTCAAAGTCGTTCCAA
ATTACAACATCACTACCAAGGACGCAGAAAAGGCAGTTTGGCAAGTACTAGTTAATATTAGAAATGTTAAAAT
TGCTGCGGGTTACTGTCCGCTGTCATTAGAATTTGTGTCAGTGTGTATTGTTTATAAAAATATTATAAAACTCG
GTTTGAGAGAGAAAATTACGAGCGTCACGGATGGAGGGCCTATGGAACTATCAGAAGAAGTTGTTGATGAGT
TCATGGAAGAAGTCCCGATGTCTGTAAGGCTTGCAAAATTTCGTTCGAAGACCGGAAAAAAGTTTAGTAGTAA
AAGTGAGAATAATAGTGGTAATAATAGGCCGAAACCAAACAAAAACCAAAGGAAGGAAAAGGGTTTAAAAG
TTAGGGTTGAGAAGGATAATTTAATTGATAATGAATTGGAGACTTACGTCGCCGATTCAGATTCGTATTAAAT
ATGTCTTACACAATCGCAACTCCATCGCAATTTGTGTTTTTGTCATCAGCATGGGCCGACCCTATAGAATTAATA
AATTTATGTACTAATTCACTAGGTAATCAGTTCCAAACACAACAAGCTAGAACAACCGTTCAACGGCAATTTAG
CGAAGTGTGGAAACCTGTCCCTCAAGTCACTGTTAGGTTTCCTGACAGTGGTTTTAAGGTGTATAGGTACAAT
GCGGTACTAGATCCTCTAGTTACTGCTTTGTTAGGAGCTTTCGATACTAGAAATAGGATTATAGAAGTCGAAA
ATCAGGCGAACCCGACAACCGCCGAAACGTTAGACGCTACTCGTAGAGTAGATGACGCAACGGTGGCTATAA
GGAGCGCTATAAATAATTTAGTAGTAGAATTGGTCAAAGGAACAGGTTTGTACAATCAGAGCACATTTGAAA
GTGCATCCGGTTTACAATGGTCCTCTGCACCTGCATCTTGA

#MZ004925

1

ATGGCATACACACAGACAGCTACCACATCCGCTTTGCTCGACACTGTCCGAGGTAACAATACCTTGGTCAACG
ATCTTGCGAAGCGGCGTCTTTATGACACAGCGGTCGACGAGTTCAACGCTCGTGATCGCAGGCCCAAAGTAA
ATTTTTCCAAAGTAATAAGTGAGGAACAGACGCTTATTGCTACTAGGGCATATCCAGAATTCCAGATAACCTTC
TATAATACGCAGAACGCCGTGCATTCGCTTGCCGGTGGACTACGATCCTTAGAACTGGAATATCTAATGATGC
AGATCCCGTACGGATCACTCACATATGATATAGGTGGGAATTTTGCATCTCATCTGTTCAAAGGACGGGCATA
TGTTCACTGCTGTATGCCCAATCTTGATGTCCGCGACATAATGCGGCACGAAGGCCAGAAAGACAGTATAGAA
TTATACCTTTCCAGGCTTGAGCGGGGCAACAAAGTTGTCCCAAATTTCCAAAAGGAAGCTTTTGACAGATACG
CTGAAACGCCAGACGAAGTTGTCTGTCACAGTACCTTCCAAACGTGTACGCACCAGCAGGTGGAAAACACAG
GCAGGGTGTATGCTATTGCATTGCACAGTATATACGATATACCTGCTGATGAATTCGGAGCGGCACTTTTAAG
GAAAAATGTCCATGTTTGTTACGCCGCCTTCCACTTTTCCGAGAATTTACTTCTCGAAGATTCACACGTCAACCT
TGACGAAATCAACGCGTGTTTTTCGCGTGATGGAGACAAGCTGACTTTTTCTTTCGCATCTGAGAGCACTTTAA
ATTATTGTCATAGTTATTCTAATATTTTAAAATACGTGTGCAAAACTTACTTCCCGGCATCTAATAGAGAGGTCT
ACATGAAGGAGTTTTTGGTCACCAGGGTTAACACCTGGTTTTGTAAGTTTTCTAGGATAGATACTTTTTTATTAT
ACAAGGGGGTAGCCCACAAAGGTGTAAATAGTGAGCAATTTTACAGCGCAATGGAAGATGCATGGCACTACA
AAAAGACTCTTGCAATGTGTAACAGCGAGAGGATTCTTCTTGAAGATTCCTCATCGGTCAATTACTGGTTCCCA
AAAATGAGAGATATGGTCATAGTTCCTCTATTCGACATATCTCTCGACACCAGTAAAAGGACCCGCAAAGAAG
TCTTAGTGTCAAAGGATTTTGTATTCACAGTTTTAAATCACATTCGCACTTATCAAGCCAAGGCACTTACATACT
CCAATGTTTTATCCTTTGTCGAATCAATTCGTTCAAGGGTAATTATCAACGGAGTGACTGCCAGGTCTGAGTGG
GATGTTGACAAATCTCTTTTGCAATCCTTGTCCATGACATTTTTCTTGCATACTAAGCTTGCCGTTTTAAAAGAC
GAATTGTTAATCAGCAAGTTTAGTTTGGGGCCAAAATCAGTAAGCCAGCATGTATGGGATGAGATTTCCCTGG
CTTTTGGAAACGCATTTCCATCGATCAAGGAGAGACTGCTAAATCGGAAACTAATTAAAGTGTCGGGAGACGC
ATTAGAAATCAGGGTGCCTGATTTATATGTGACTTTTCACGATAGATTAGTGACTGAGTACAAAACATCGGTG
GATATGCCAGTGCTTGATATCAGAAAGAGAATGGAGGAGACTGAGGTTATGTACAATGCATTGTCTGAGCTA
TCTGTGCTCAAGGAGTCGGACAAGTTCGACGTTGATGTTTTTTCCCGGATGTGCCAGACTTTGGAGGTAGACC
CAATGACTGCAGCAAAGGTTATTGTGGCAGTGATGAGCAACGAGAGCGGACTGACTCTTACATTCGAACAGC

CAACTGAAGCAAATGTCGCATTGGCACTTAAAGATTCAGAAAAAGCCTCTGAGGGTGCACTAGTGGTTACTTC
TAGAGATGTTGAAGAACCATCCATGAAGGGTTCAATGGCAAGAGGAGAGTTACAATTGGCCGGTCTGTCTGG
AGACCAACCAGAGTCTTCCTATACTCGGAACGAGGAAATAGAGTCATTAGAGCAATTCCACATGGCAACGGCT
AGTTCGTTAATTCGGAAACAGATGAGTTCGATTGTGTACACGGGCCCCATTAAAGTTCAGCAAATGAAAAACT
TTATTGATAGCCTGGTAGCATCACTCTCTGCTGCGGTGTCGAACCTAGTCAAGATCCTAAAGGATACAGCTGCT
ATAGATCTCGAAACCCGTCAGAAGTTTGGAGTCTTAGATGTTGCGACCAAAAGATGGTTAATTAAACCTTTAG
CCAAGAATCACGCATGGGGCGTTATTGAAACACATGCTAGGAAGTACCACGTTGCACTTTTGGAGTATGATGA
GCATGGAGTGGTAACTTGCGACAGTTGGAGAAGGGTGGCCGTGAGTTCTGAGTCAATGGTTTATTCTGATAT
GGCAAAGCTCAGAACACTGAGGAGATTATTAAGAGATGGTGAGCCTCATGTCAGCAGTGCTAAAGTCGTCCT
AGTTGACGGTGTCCCGGGTTGTGGAAAGACAAAAGAGATTCTCTCGAAAGTAAATTTTGAGGAAGATCTAAT
CTTAGTACCGGGTAAGCAGGCTGCTGAAATGATAAAGAGGCGTGCTAATGCGTCAGGAATAATTCAAGCCAC
AAGAGATAATGTTCGTACTGTTGATTCATTTATAATGAATTACGGTAAAGGAACACGCTGTCAGTTCAAAAGG
TTATTTATCGACGAAGGTCTGATGTTGCACACTGGTTGTGTGAATTTTCTTGTTTCTATGTCTCTGTGCGAAATT
GCATATGTTTATGGAGACACACAACAAATTCCATACATCAACAGAGTATCCGGTTTTCCGTACCCTGCACATTT
TGCAAAAATAGAGGTTGATGAGGTGGAAACTCGCAGAACTACGCTGCGTTGTCCAGCCGACATTACTCACTAT
CTTAACAGAAGGTACGATGGACATGTCATGTGTACATCGTCGGTTAAAAAGTCAGTTTCTCAGGAAATGGTGA
GCGGGGCCGCAATGATCAATCCTGTATCTAAGCCATTGAATGGGAAAGTTTTGACTTTCACTCAGTCTGATAA
AGAGGCGCTGCTTTCTCGAGGATATACGGACGTCCATACAGTACATGAGGTACAAGGTGAGACATATGCAGA
TGTGTCGTTGGTCAGATTGACTCCGACACCTGTATCTATCATCGTAGGAGATAGTCCGCACGTTCTCGTAGCTT
TGTCAAGGCATACCCAAACATTGAAGTATTACACCGTAGTGATGGATCCTCTTGTAAGTATAATTAGGGATTTA
GAAAAACTTAGTTCTTACTTGTTAGATATGTATAAAGTAGATGCAGGGACCCAATAGCAATTACAGGTAGACT
CCGTGTTTAAAGGTTCTAATCTTTTTGTTGCAGCACCAAAGACTGGAGATATCTCAGATATGCAATTTTACTAT
GATAAGTGTCTCCCAGGTAATAGCACCATGTTAAATAACTATGATGCTGTTACCATGAGGTTGACTGACATTTC
TCTTAATGTCAAAGATTGCATATTGGATTTCTCTAAGTCTGTGGCTGCACCGAAGGATCCGATCAAACCACTGA
TTCCAATGGTACGAACGGCGGCAGAAATGCCACGCCAGACTGGACTATTGGAAAATTTGGTGGCGATGATCA
AAAGAAACTTTAATTCACCGGAGTTATCAGGAATAATCGACATTGAGAATACTGCATCTTTAGTAGTAGATAA
ATTTTTTGATAGTTACTTGCTAAAAGAAAAAAGAAAACCAAATAAAAATGTTTCTTTATTTTGTAGAGAGTCTC
TCAATAGATGGTTAGAGAAGCAGGAGCAAGTGACCATTGGTCAGCTTGCAGATTTTGATTTTGTGGATCTTCC
TGCCGTTGATCAGTACAGGCATATGATTAAAGCGCAACCTAAGCAGAAGCTGGATACATCAATTCAAAGCGAA
TATCCGGCCTTGCAGACGATTGTGTATCATTCGAAAAAGATCAACGCAATCTTCGGTCCTTTGTTCAGTGAGCT
CACAAGGCAAATGCTCGAAAGCATAGACTCAAGTAAGTTTTTGTTCTTTACAAGGAAGACGCCAGCTCAAATT
GAGGATTTCTTCGGAGATCTCGATAGCCATGTCCCTATGGATATCTTGGAGTTGGATATTTCGAAGTATGACA
AATCTCAGAACGAGTTCCACTGTGCAGTAGAGTATGAAATATGGAGAAGACTTGGATTAGAAGATTTTCTGG
GAGAAGTTTGGAAACAAGGCCACAGAAAAACTACTCTTAAAGATTACACAGCTGGTATTAAAACGTGTTTATG
GTACCAGAGAAAGAGTGGGGACGTTACAACATTCATCGGTAATACGGTGATTATTGCTGCTTGTTTAGCTTCC
ATGTTGCCCATGGAGAAAATAATCAAAGGTGCATTTTGCGGAGATGACAGTTTACTATACTTCCCAAAAGGTT
GTGAGTTTCCTGACATACAGCATACAGCCAACCTTATGTGGAATTTCGAGGCTAAGCTATTCAGAAAGCAGTA
TGGTTATTTCTGTGGAAGGTACGTGATACATCATGACAGAGGGTGTATTGTTTATTATGACCCTTTGAAGTTGA
TTTCTAAACTTGGTGCTAAACATATCAAGGATTGGGATCACTTAGAAGAGTTCAGAAGATCCCTTTGTGATGTT
GCAAATTCGTTGAACAACTGTGCGTATTACACGCAGTTGGACGACGCTGTGAGTGAGGTCCATAAAACCGCAC
CCCCGGGTTCGTTTGTATATAAAAGTTTAGTTAAATATCTGTCCGATAAGGTTCTTTTTAGAAGTTTGTTTATAG
ATGGCTCTTGTTAAGGGTAAAGTTAATATTAATGAGTTCATAGACTTGTCAAAATCAGAAAAAATTTCTTCCGTC
TATGTTCACACCTGTTAAGAGTGTCATGATCTCCAAGGTTGATAAGATATTGGTTCATGAAGATGAATCTTTGT
CCGAAGTCAATTTACTTAAAGGTGTAAAACTCATTGATGGTGGCTATGTACATCTTGCTGGTCTTGTGGTGACA
GGTGAATGGAATTTGCCAGATAATTGTCGTGGTGGTGTCAGTGTCTGTTTGGTCGATAAGAGAATGGAGAGA
GCGGACGAGGCAACTCTTGCTTCATACTATACCGCAGCGGCTAAGAAAAGGTTTCAGTTCAAAGTCGTTCCAA
ATTACAACATCACTACCAAGGACGCAGAAAAGGCAGTTTGGCAAGTACTAGTTAATATTAGAAATGTTAAAAT
TGCTGCGGGTTACTGTCCGCTGTCATTAGAATTTGTGTCAGTGTGTATTGTTTATAAAAATATTATAAAACTCG

GTTTGAGAGAGAAAATTACGAGCGTCACGGATGGAGGGCCCATGGAACTATCAGAAGAAGTTGTTGATGAG
TTCATGGAAGAAGTCCCGATGTCTGTAAGGCTTGCAAAATTTCGTTCGAAGACCGGGAAAAAGTTTAGTAGTA
AAAGTGAGAATAATAGTGGTAATAATAGGCCGAAACCAAACAAAAACCAAAGGAAGGAAAAGGGTTTAAAA
GTTAGGGTTGAGAAGGATAATTTAATTGATAATGAATTGGAGACTTACGTCGCCGATTCAGATTCGTATTAAA
TATGTCTTACACAATCGCAACTCCATCGCAATTTGTGTTTTTGTCATCAGCATGGGCCGACCCTATAGAATTAAT
AAATTTATGTACTAATTCACTAGGTAATCAGTTCCAAACACAACAAGCTAGAACAACCGTTCAACGGCAATTTA
GCGAAGTGTGGAAACCTGTCCCTCAAGTCACTGTTAGGTTTCCTGACAGTGGTTTTAAGGTGTATAGGTACAA
TGCGGTACTAGATCCTCTAGTTACTGCTTTGTTAGGAGCTTTCGATACTAGAAATAGGATTATAGAAGTCGAAA
ATCAGGCGAACCCGACAACCGCCGAAACGTTAGACGCTACTCGTAGAGTAGATGACGCAACGGTGGCTATAA
GGAGCGCTATAAATAATTTAGTAGTAGAATTGGTCAAAGGAACAGGTTTGTACAATCAGAGCACATTTGAAA
GTGCATCCGGTTTACAATGGTCCTCTGCACCTGCATCTTGA

#MZ323110

1

ATGGCATACACACAGACAGCTACCACATCCGCTTTGCTCGACACTGTCCGAGGTAACAATACCTTGGTCAATG
ATCTTGCGAAGCGGCGTCTTTATGACACAGCGGTCGACGAGTTCAACGCTCGTGATCGCAGGCCCAAAGTAA
ATTTTTCCAAAGTAATAAGTGAGGAACAGACGCTTATTGCTACTAGGGCATATCCAGAATTCCAGATAACCTTC
TATAATACGCAGAACGCCGTGCATTCGCTTGCCGGTGGACTACGATCCTTAGAACTGGAATATCTAATGATGC
AGATCCCGTATGGATCACTCACATATGATATAGGTGGGAATTTTGCATCTCATCTGTTCAAAGGACGGGCATA
TGTTCACTGCTGTATGCCCAATCTTGATGTCCGCGACATAATGCGGCACGAAGGCCAGAAAGACAGTATAGAA
TTATACCTTTCCAGGCTTGAGCGGGGCAACAAAGTTGTCCCAAATTTCCAAAAGGAAGCTTTTGACAGATACG
CTGAAACGCCAGACGAAGTTGTCTGTCACAGTACCTTCCAAACGTGTACGCACCAGCAGGTGGAAAACACAG
GCAGAGTGTATGCTATTGCATTGCACAGTATATACGATATACCTGCTGATGAATTCGGAGCGGCACTTTTAAG
GAAAAATGTCCATGTTTGTTACGCCGCCTTCCACTTTTCCGAGAATTTACTTCTCGAAGATTCACACGTCAACCT
TGACGAAATCAACGCGTGTTTTTCGCGTGATGGAGACAAGCTGACTTTTTCTTTCGCATCTGAGAGCACTTTAA
ATTATTGTCATAGTTATTCTAATATTTTAAAATACGTGTGCAAAACTTACTTCCCGGCATCTAATAGAGAGGTCT
ACATGAAGGAGTTTTTGGTCACCAGGGTTAACACCTGGTTTTGTAAGTTTTCTAGGATAGACACTTTTTTATTA
TACAAGGGGGTAGCCCACAAAGGTGTAAATAGTGAGCAATTTTACAGCGCAATGGAAGATGCATGGCACTAC
AAAAAGACTCTTGCAATGTGTAACAGCGAGAGGATTCTTCTTGAAGATTCCTCATCGGTCAATTACTGGTTCCC
AAAAATGAGAGATATGGTCATAGTTCCTCTATTCGACATATCTCTCGACACCAGTAAAAGGACCCGCAAAGAA
GTCTTAGTGTCAAAGGATTTTGTATTCACAGTTTTAAATCACATTCGCACTTATCAAGCCAAGGCACTTACATAC
TCCAATGTTTTATCCTTTGTCGAATCAATTCGTTCAAGGGTAATTATCAACGGAGTGACTGCCAGGTCTGAGTG
GGATGTTGACAAATCTCTTTTGCAATCCTTGTCCATGACATTTTTCTTGCATACTAAGCTTGCCGTTTTAAAAGA
CGAATTGTTAATCAGCAAGTTTAGTTTGGGGCCAAAATCAGTAAGCCAGCATGTATGGGATGAGATTTCCCTG
GCTTTTGGAAACGCATTTCCATCGATCAAGGAGAGACTGCTAAATCGGAAACTAATTAAAGTGTCGGGAGAC
GCATTAGAAATCAGGGTGCCTGATTTATATGTGACTTTTCACGATAGATTAGTGACTGAGTACAAAACATCGG
TGGATATGCCAGTGCTTGATATCAGAAAGAGAATGGAGGAGACTGAGGTTATGTACAATGCATTGTCTGAGC
TATCTGTGCTCAAGGAGTCGGACAAGTTCGACGTTGATGTTTTTTCCCGGATGTGCCAGACTTTGGAGGTTGA
CCCAATGACTGCAGCAAAGGTTATTGTGGCAGTGATGAGCAACGAGAGCGGACTGACTCTTACATTCGAACA
GCCAACTGAAGCAAATGTCGCATTGGCACTTAAAGATTCAGAAAAAGCCTCTGAGGGTGCACTAGTGGTTACT
TCTAGAGATGTTGAAGAACCATCCATGAAGGGTTCAATGGCAAGAGGAGAGTTACAATTGGCCGGTCTGTCT
GGAGACCAACCAGAGTCTTCCTATACTCGGAACGAGGAAATAGAGTCATTAGAGCAATTCCACATGGCAACG
GCTAGTTCGTTAATTCGGAAACAGATGAGTTCGATTGTGTACACGGGCCCCATTAAAGTTCAGCAAATGAAAA
ACTTTATTGATAGCCTGGTAGCATCACTCTCTGCTGCGGTGTCGAACCTAGTCAAGATCCTAAAGGATACAGCT

GCTATAGATCTCGAAACCCGTCAGAAGTTTGGAGTCTTAGATGTTGCAACCAAAAGATGGTTAATTAAACCTTT
AGCCAAGAATCACGCATGGGGCGTTATTGAAACACATGCTAGGAAGTACCACGTTGCACTTTTGGAGTATGAT
GAGCATGGAGTGGTAACTTGCGACAGTTGGAGAAGGGTGGCCGTGAGTTCTGAGTCAATGGTTTATTCTGAT
ATGGCAAAGCTCAGAACACTGAGGAGATTATTAAGAGATGGTGAGCCTCATGTCAGCAGTGCTAAAGTCGTC
CTAGTCGACGGTGTCCCGGGTTGTGGAAAGACAAAAGAGATTCTCTCGAAAGTAAATTTTGAGGAAGATCTA
ATCTTAGTACCGGGTAAGCAGGCTGCTGAAATGATAAAGAGGCGTGCTAATGCGTCAGGAATAATTCAAGCC
ACAAGAGATAATGTTCGTACTGTTGATTCATTTATAATGAATTACGGTAAAGGAACACGCTGTCAGTTCAAAA
GGTTATTTATCGACGAAGGTCTGATGTTGCACACTGGTTGTGTGAATTTTCTTGTTTCTATGTCTCTGTGCGAA
ATTGCATATGTTTATGGAGACACACAACAAATTCCATACATCAACAGAGTATCCGGTTTTCCGTACCCTGCACA
TTTTGCAAAAATAGAGGTTGATGAGGTGGAAACTCGCAGAACTACGCTGCGTTGTCCAGCCGACATTACCCAC
TATCTTAACAGAAGGTACGAAGGATATGTCATGTGTACATCGTCGGTTAAAAAGTCAGTTTCTCAGGAAATGG
TGAGCGGGGCCGCAATGATCAATCCTGTATCTAAGCCATTGAATGGGAAAGTTTTGACTTTCACTCAGTCTGA
TAAAGAGGCGCTGCTTTCTCGAGGATATACGGACGTCCATACAGTACATGAGGTACAAGGTGAGACATATGC
AGATGTGTCGTTGGTCAGATTGACTCCGACACCTGTATCTATCATCGCAGGAGATAGTCCGCACGTTCTCGTA
GCTTTGTCAAGGCATACCCAAACATTGAAGTATTACACCGTAGTGATGGATCCTCTTGTAAGTATAATTAGGG
ATTTAGAAAAACTTAGTTCTTACTTGTTAGATATGTATAAAGTAGATGCAGGGACCCAATAGCAATTACAGGT
AGACTCCGTGTTTAAAGGTTCTAATCTTTTTGTTGCAGCACCAAAGACTGGAGATATCTCAGATATGCAATTTT
ACTATGATAAGTGTCTCCCAGGTAATAGCACCATGTTAAATAACTATGATGCTGTTACCATGAGGTTGACTGAC
ATTTCTCTTAATGTCAAAGATTGCATATTGGATTTCTCTAAGTCTGTGGCTGCACCGAAGGATCCGATCAAACC
ACTGATTCCAATGGTACGAACAGCGGCAGAAATGCCACGCCAGACTGGACTATTGGAAAATTTGGTGGCGAT
GATCAAAAGAAACTTTAATTCACCGGAGTTATCAGGAATAATCGACATTGAAAATACTGCATCTTTAGTAGTA
GATAAATTTTTTGATAGTTACTTGCTTAAAGAAAAAAGAAAACCAAATAAAAATGTTTCTTTATTTTGTAGAGA
GTCTCTCAATAGATGGTTAGAGAAGCAGGAGCAAGTGACCATTGGTCAGCTTGCAGATTTTGATTTTGTGGAT
CTTCCAGCCGTTGATCAGTACAGGCATATGATTAAAGCGCAACCTAAGCAGAAGCTGGATACATCAATTCAAA
GCGAATATCCGGCCTTGCAGACGATTGTGTATCATTCGAAAAAGATCAACGCAATCTTCGGTCCTTTGTTCAGT
GAGCTCACAAGGCAAATGCTCGAAAGCATAGACTCAAGTAAGTTTTTGTTCTTTACAAGGAAGACGCCAGCTC
AAATTGAGGATTTCTTCGGAGATCTCGATAGCCATGTCCCTATGGATATCTTGGAGTTGGATATTTCGAAGTAT
GACAAATCTCAGAACGAGTTCCACTGTGCAGTAGAGTATGAAATATGGAGAAGACTTGGATTAGAAGATTTTC
TGGGAGAAGTTTGGAAACAAGGCCACAGGAAAACTACTCTTAAAGATTACACAGCTGGTATTAAAACGTGTTT
ATGGTACCAGAGAAAGAGTGGGGACGTTACAACATTCATCGGTAATACGGTGATTATTGCTGCTTGTTTAGCT
TCCATGTTGCCCATGGAGAAAATAATCAAAGGTGCATTTTGCGGAGATGACAGTTTACTATACTTCCCAAAAG
GTTGTGAGTTTCCTGACATACAGCATACAGCCAACCTTATGTGGAATTTCGAGGCTAAGCTATTCAGAAAGCA
GTATGGTTATTTCTGTGGAAGGTACGTGATACATCATGACAGAGGGTGTATTGTTTATTATGACCCTTTGAAGT
TGATTTCTAAACTTGGTGCTAAACACATCAAGGATTGGGATCACTTAGAAGAGTTCAGAAGATCCCTTTGTGAT
GTTGCAAATTCGTTGAACAACTGTGCGTATTACACGCAGTTGGACGACGCTGTGAGTGAGGTCCATAAAACCG
CACCCCCGGGTTCGTTTGTATATAAAAGTTTAGTTAAATATCTGTCCGATAAGGTTCTTTTTAGAAGTTTGTTTA
TAGATGGCTCTTGTTAAGGGTAAAGTCAATATTAATGAGTTCATAGACTTGTCAAAATCAGAAAAATTTCTTCC
GTCTATGTTCACACCTGTTAAGAGTGTCATGATCTCCAAGGTTGATAAGATATTGGTTCATGAAGATGAATCTT
TGTCCGAAGTCAATTTACTCAAAGGTGTAAAACTCATTGATGGTGGCTATGTACACCTTGCTGGTCTTGTGGTG
ACAGGTGAATGGAATTTGCCAGATAATTGTCGTGGTGGTGTCAGTGTCTGTTTGGTCGATAAGAGAATGGAG
AGAGCGGACGAGGCAACTCTTGCTTCATACTATACCGCAGCGGCTAAGAAAAGGTTTCAGTTCAAAGTCGTTC
CAAATTACAACATCACTACCAAGGACGCAGAAAAGGCAGTTTGGCAAGTACTAGTTAATATTAGAAATGTTAA
AATTGCTGCGGGTTACTGTCCGCTGTCATTAGAATTTGTGTCAGTGTGTATTGTTTATAAAAATATTATAAAAC
TCGGTTTGAGAGAGAAAATTACGAGCGTCACGGATGGAGGGCCCATGGAACTATCAGAAGAAGTTGTTGATG
AGTTCATGGAAGAAGTCCCGATGTCTGTAAGGCTTGCAAAATTTCGTTCGAAGACCGGAAAAAAGTTTAGTAG
TAAAAGTGAGAATAATAGTGGTAATAATAGGCCGAAACCAGACAAAAACCAAAGGAAGGGAAAGGGTTTAA
AAGTTAGGGTTGAGAAGGATAATTTAATTGATAATGAATTGGAGACTTACGTCGCCGATTCAGATTCGTATTA
AATATGTCTTACACAATCGCAACTCCATCGCAATTTGTGTTTTTGTCATCAGCATGGGCCGACCCTATAGAATTA

ATAAATTTATGTACTAATTCACTAGGTAATCAGTTCCAAACACAACAAGCTAGAACAACCGTTCAACGGCAATT
TAGCGAAGTGTGGAAACCTGTCCCTCAAGTCACTGTTAGGTTTCCTGACAGTGGTTTTAAGGTGTATAGGTAC
AATGCGGTACTAGATCCTCTAGTTACTGCTTTGTTAGGAGCTTTCGATACTAGAAATAGGATTATAGAAGTCGA
AAATCAGGCGAACCCGACAACCGCCGAAACGTTAGACGCTACTCGTAGAGTAGATGACGCAACGGTGGCTAT
AAGGAGCGCTATAAATAATTTAGTAGTAGAATTGGTCAAAGGAACAGGTTTGTACAATCAGAGCACATTTGA
AAGTGCATCCGGTTTACAATGGTCCTCTGCACCTGCATCTTGA

#MZ438228

1

ATGGCATACACACAGACAGCTACCACATCCGCTTTGCTCGACACTGTCCGAGGTAACAATACCTTGGTCAATG
ATCTTGCGAAGCGGCGTCTTTATGACACAGCGGTCGACGAGTTCAACGCTCGTGATCGCAGGCCCAAAGTAA
ATTTTTCCAAAGTAATAAGTGAGGAACAGACGCTTATTGCTACTAGGGCATATCCAGAATTCCAGATAACCTTC
TATAATACGCAGAACGCCGTGCATTCGCTTGCCGGTGGACTACGATCCTTAGAACTGGAATATCTAATGATGC
AGATCCCGTATGGATCACTCACATATGATATAGGTGGGAATTTTGCATCTCATCTGTTCAAAGGACGGGCATA
TGTTCACTGCTGTATGCCCAATCTTGATGTCCGCGACATAATGCGGCACGAAGGCCAGAAAGACAGTATAGAA
TTATACCTTTCCAGGCTTGAGCGGGGCAACAAAGTTGTCCCAAATTTCCAAAAGGAAGCTTTTGACAGATACG
CTGAAACGCCAGACGAAGTTGTCTGTCACAGTACCTTCCAAACGTGTACGCACCAGCAGGTGGAAAACACAG
GCAGAGTGTATGCTATTGCATTGCACAGTATATACGATATACCTGCTGATGAATTCGGAGCGGCACTTTTAAG
GAAAAATGTCCATGTTTGTTACGCCGCCTTCCACTTTTCCGAGAATTTACTTCTCGAAGATTCACACGTCAACCT
TGACGAAATCAACGCGTGTTTTTCGCGTGATGGAGACAAGCTGACTTTTTCTTTCGCATCTGAGAGCACTTTAA
ATTATTGTCATAGTTATTCTAATATTTTAAAATACGTGTGCAAAACTTACTTCCCGGCATCTAATAGAGAGGTCT
ACATGAAGGAGTTTTTGGTCACCAGGGTTAACACCTGGTTTTGTAAGTTTTCTAGGATAGATACTTTTTTATTAT
ACAAGGGGGTAGCCCACAAAGGTGTAAATAGTGAGCAATTTTACAGCGCAATGGAAGATGCATGGCACTACA
AAAAGACTCTTGCAATGTGTAACAGCGAGAGGATTCTTCTTGAAGATTCCTCATCGGTCAATTACTGGTTCCCA
AAAATGAGAGATATGGTCATAGTTCCTCTATTCGACATATCTCTCGACACCAGTAAAAGGACCCGCAAAGAAG
TCTTAGTGTCAAAGGATTTTGTATTCACAGTTTTAAATCACATTCGCACTTATCAAGCCAAGGCACTTACATACT
CCAATGTTTTATCCTTTGTCGAATCAATTCGTTCAAGGGTAATTATCAACGGAGTGACTGCCAGGTCTGAGTGG
GATGTTGACAAATCTCTTTTGCAATCCTTGTCCATGACATTTTTCTTGCATACTAAGCTTGCCGTTTTAAAAGAC
GAATTGTTAATCAGCAAGTTTAGTTTGGGGCCAAAATCAGTAAGCCAGCATGTATGGGATGAGATTTCCCTGG
CTTTTGGAAACGCATTTCCATCGATCAAGGAGAGACTGCTAAATCGGAAACTAATTAAAGTGTCGGGAGACGC
ATTAGAAATCAGGGTGCCTGATTTATATGTGACTTTTCACGATAGATTAGTGACTGAGTACAAAACATCGGTG
GATATGCCAGTGCTTGATATCAGAAAGAGAATGGAGGAGACTGAGGTTATGTACAATGCATTGTCTGAGCTA
TCTGTGCTCAAGGAGTCGGACAAGTTCGACGTTGATGTTTTTTCCCGGATGTGCCAGACTTTGGAGGTTGACC
CAATGACTGCAGCAAAGGTTATTGTGGCAGTGATGAGCAACGAGAGCGGACTGACTCTTACATTCGAACAGC
CAACTGAAGCAAATGTCGCATTGGCACTTAAAGATTCAGAAAAAGCCTCTGAGGGTGCACTAGTGGTTACTTC
TAGAGATGTTGAAGAACCATCCATGAAGGGTTCAATGGCAAGAGGAGAGTTACAATTGGCCGGTCTGTCTGG
AGACCAACCAGAGTCTTCCTATACTCGGAACGAGGAAATAGAGTCATTAGAGCAATTCCACATGGCAACGGCT
AGTTCGTTAATTCGGAAACAGATGAGTTCGATTGTGTACACGGGCCCCATTAAAGTTCAGCAAATGAAAAACT
TTATTGATAGCCTGGTAGCATCACTCTCTGCTGCGGTGTCGAACCTAGTCAAGATCCTAAAGGATACAGCTGCT
ATAGATCTCGAAACCCGTCAGAAGTTTGGAGTCTTAGATGTTGCAACCAAAAGATGGTTAATTAAACCTTTAG
CCAAGAATCACGCATGGGGCGTTATTGAAACACATGCTAGGAAGTACCACGTTGCACTTTTGGAGTATGATGA
GCATGGAGTGGTAACTTGCGACAGTTGGAGAAGGGTGGCCGTGAGTTCTGAGTCAATGGTTTATTCTGATAT
GGCAAAGCTCAGAACACTGAGGAGATTATTAAGAGATGGTGAGCCTCATGTCAGCAGTGCTAAAGTCGTCCT
AGTCGACGGTGTCCCGGGTTGTGGAAAGACAAAAGAGATTCTCTCGAAAGTAAATTTTGAGGAAGATCTAAT

CTTAGTACCGGGTAAGCAGGCTGCTGAAATGATAAAGAGGCGTGCTAATGCGTCAGGAATAATTCAAGCCAC
AAGAGATAATGTTCGTACTGTTGATTCATTTATAATGAATTACGGTAAAGGAACACGCTGTCAGTTCAAAAGG
TTATTTATCGACGAAGGTCTGATGTTGCACACTGGTTGTGTGAATTTTCTTGTTTCTATGTCTCTGTGCGAAATT
GCATATGTTTATGGAGACACACAACAAATTCCATACATCAACAGAGTATCCGGTTTTCCGTACCCTGCACATTT
TGCAAAAATAGAGGTTGATGAGGTGGAAACTCGCAGAACTACGCTGCGTTGTCCAGCCGACATTACCCACTAT
CTTAACAGAAGGTACGAAGGATATGTCATGTGTACATCGTCGGTTAAAAAGTCAGTTTCTCAGGAAATGGTGA
GCGGGGCCGCAATGATCAATCCTGTATCTAAGCCATTGAATGGGAAAGTTTTGACTTTCACTCAGTCTGATAA
AGAGGCGCTGCTTTCTCGAGGATATACGGACGTCCATACAGTACATGAGGTACAAGGTGAGACATATGCAGA
TGTGTCGTTGGTCAGATTGACTCCGACACCTGTATCTATCATCGCAGGAGATAGTCCGCACGTTCTCGTAGCTT
TGTCAAGGCATACCCAAACATTGAAGTATTACACCGTAGTGATGGATCCTCTTGTAAGTATAATTAGGGATTTA
GAAAAACTTAGTTCTTACTTGTTAGATATGTATAAAGTAGATGCAGGGACCCAATAGCAATTACAGGTAGACT
CCGTGTTTAAAGGTTCTAATCTTTTTGTTGCAGCACCAAAGACTGGAGATATCTCAGATATGCAATTTTACTAT
GATAAGTGTCTCCCAGGTAATAGCACCATGTTAAATAACTATGATGCTGTTACCATGAGGTTGACTGACATTTC
TCTTAATGTCAAAGATTGCATATTGGATTTTTCTAAGTCTGTGGCTGCACCGAAGGATCCGATCAAACCACTGA
TTCCAATGGTACGAACAGCGGCAGAAATGCCACGCCAGACTGGACTATTGGAAAATTTGGTGGCGATGATCA
AAAGAAACTTTAATTCACCGGAGTTATCAGGAATAATCGACATTGAAAATACTGCATCTTTAGTAGTAGATAA
ATTTTTTGATAGTTACTTGCTTAAAGAAAAAAGAAAACCAAATAAAAATGTTTCTTTATTTTGTAGAGAGTCTCT
CAATAGATGGTTAGAGAAGCAGGAGCAAGTGACCATTGGTCAGCTTGCAGATTTTGATTTTGTGGATCTTCCA
GCCGTTGATCAGTACAGGCATATGATTAAAGCGCAACCAAAGCAGAAGCTGGATACATCAATTCAAAGCGAA
TATCCGGCCTTGCAGACGATTGTGTATCATTCGAAAAAGATCAACGCAATCTTCGGTCCTTTGTTCAGTGAGCT
CACAAGGCAAATGCTCGAAAGCATAGACTCAAGTAAGTTTTTGTTCTTTACAAGGAAGACGCCAGCTCAAATT
GAGGATTTCTTCGGAGATCTCGATAGCCATGTCCCTATGGATATCTTGGAGTTGGATATTTCGAAGTATGACA
AATCTCAGAACGAGTTCCACTGTGCAGTAGAGTATGAAATATGGAGAAGACTTGGATTAGAAGATTTTCTGG
GAGAAGTTTGGAAACAAGGCCACAGGAAAACTACTCTTAAAGATTACACAGCTGGTATTAAAACGTGTTTATG
GTACCAGAGAAAGAGTGGGGACGTTACAACATTCATCGGTAATACGGTGATTATTGCTGCTTGTTTAGCTTCC
ATGTTGCCCATGGAGAAAATAATCAAAGGTGCATTTTGCGGAGATGACAGTTTACTATACTTCCCAAAAGGTT
GTGAGTTTCCTGACATACAGCATACAGCCAACCTTATGTGGAATTTCGAGGCTAAGCTATTCAGAAAGCAGTA
TGGTTATTTCTGTGGAAGGTACGTGATACATCATGACAGAGGGTGTATTGTTTATTATGACCCTTTGAAGTTGA
TTTCTAAACTTGGTGCTAAACACATCAAGGATTGGGATCACTTAGAAGAGTTCAGAAGATCCCTTTGTGATGTT
GCAAATTCGTTGAACAACTGTGCGTATTACACGCAGTTGGACGACGCTGTGAGTGAGGTCCATAAAACCGCAC
CCCCGGGTTCGTTTGTATATAAAAGTTTAGTTAAATATCTGTCCGATAAGGTTCTTTTTAGAAGTTTGTTTATAG
ATGGCTCTTGTTAAGGGTAAAGTCAATATTAATGAGTTCATAGACTTGTCAAAATCAGAAAAAATATCTTCCGTC
TATGTTCACACCTGTTAAGAGTGTCATGATCTCCAAGGTTGATAAGATATTGGTTCATGAAGATGAATCTTTGT
CCGAAGTCAATTTACTCAAAGGTGTAAAACTCATTGATGGTGGCTATGTACACCTTGCTGGTCTTGTGGTGACA
GGTGAATGGAATTTGCCAGATAAATGTCGTGGTGGTGTCAGTGTCTGTTTGGTCGATAAGAGAATGGAGAGA
GCGGACGAGGCAACTCTTGCTTCATACTATACCGCAGCGGCTAAGAAAAGGTTTCAGTTCAAAGTCGTTCCAA
ATTACAACATCACTACCAAGGACGCAGAAAAGGCAGTTTGGCAAGTACTAGTTAATATTAGAAATGTTAAAAT
TGCTGCGGGTTACTGTCCGCTGTCATTAGAATTTGTGTCAGTGTGTATTGTTTATAAAAATATTATAAAACTCG
GTTTGAGAGAGAAAATTACGAGCGTCACGGATGGAGGGCCCATGGAACTATCAGAAGAAGTTGTTGATGAG
TTCATGGAAGAAGTCCCGATGTCTGTAAGGCTTGCAAAATTTCGTTCGAAGACCGGAAAAAAGTTTAGTAGTA
AAAGTGAGAATAATAGTGGTAATAATAGGCCGAAACCAGACAAAAACCAAAGGAAGGGAAAGGGTTTAAAA
GTTAGGGTTGAGAAGGATAATTTAATTGATAATGAATTGGAGACTTACGTCGCCGATTCAGATTCGTATTAAA
TATGTCTTACACAATCGCAACTCCATCGCAATTTGTGTTTTTGTCATCAGCATGGGCCGACCCTATAGAATTAAT
AAATTTATGTACTAATTCACTAGGTAATCAGTTCCAAACACAACAAGCTAGAACAACCGTTCAACGGCAATTTA
GCGAAGTGTGGAAACCTGTCCCTCAAGTCACTGTTAGGTTTCCTGACAGTGGTTTTAAGGTGTATAGGTACAA
TGCGGTACTAGATCCTCTAGTTACTGCTTTGTTAGGAGCTTTCGATACTAGAAATAGGATTATAGAAGTCGAAA
ATCAGGCGAACCCGACAACCGCCGAAACGTTAGACGCTACTCGTAGAGTAGATGACGCAACGGTGGCTATAA

GGAGCGCTATAAATAATTTAGTAGTAGAATTGGTCAAAGGAACAGGTTTGTACAATCAGAGCACATTTGAAA
GTGCATCCGGTTTACAATGGTCCTCTGCACCTGCATCTTGA

#MZ945420

1

ATGGCATACACACAGACAGCTACCACATCCGCTTTGCTCGACACTGTCCGAGGTAACAATACCTTGGTCAACG
ATCTTGCGAAGCGGCGTCTTTATGACACAGCGGTCGACGAGTTCAACGCTCGTGATCGCAGGCCCAAAGTAA
ATTTTTCCAAAGTAATAAGTGAGGAACAGACGCTTATTGCTACTAGGGCATATCCTGAATTCCAGATAACCTTC
TATAATACGCAGAACGCCGTGCATTCGCTTGCTGGTGGACTACGATCCTTAGAACTGGAATATCTAATGATGC
AGATCCCGTACGGATCACTCACATATGATATAGGTGGGAATTTTGCATCTCATCTGTTCAAAGGACGGGCATA
TGTTCACTGCTGTATGCCCAATCTTGATGTCCGCGACATAATGCGGCACGAAGGCCAGAAAGACAGTATAGAA
TTATACCTTTCCAGGCTTGAGCGGGGCAACAAAGTTGTCCCAAATTTCCAAAAGGAAGCTTTTGACAGATACG
CTGAAACGCCAGACGAAGTTGTCTGTCACAGTACCTTCCAAACGTGTACGCACCAGCAGGTGGAAAACACAG
GCAGGGTGTATGCTATTGCATTGCACAGTATATACGATATACCTGCTGATGAATTCGGAGCGGCACTTTTAAG
GAAAAATGTCCATGTTTGTTACGCCGCCTTCCACTTTTCCGAGAATTTACTTCTCGAAGATTCACACGTCAACCT
TGACGAAATCAACGCGTGTTTTTCGCGTGATGGAGACAAGCTGACTTTTTCTTTCGCATCTGAGAGCACTTTAA
ATTATTGTCATAGTTATTCTAATATTTTAAAATACGTGTGCAAAACTTACTTCCCGGCATCTAATAGAGAGGTCT
ACATGAAGGAGTTTTTGGTCACCAGGGTTAACACCTGGTTTTGTAAGTTTTCTAGGATAGATACTTTTTTATTAT
ACAAGGGGGTAGCCCACAAAGGTGTAAATAGTGAGCAATTTTACAGCGCAATGGAAGATGCATGGCACTACA
AAAAGACTCTTGCAATGTGTAACAGCGAGAGGATTCTTCTTGAAGATTCCTCATCGGTCAATTACTGGTTCCCA
AAAATGAGAGATATGGTCATAGTTCCTCTATTCGACATATCTCTCGACACTAGTAAAAGGACCCGCAAAGAAG
TCTTAGTGTCAAAGGATTTTGTATTCACAGTTTTAAATCACATTCGCACTTATCAAGCCAAGGCACTTACATACT
CCAATGTTTTATCCTTTGTCGAATCAATTCGTTCAAGGGTAATTATCAACGGAGTGACTGCCAGGTCTGAGTGG
GATGTTGACAAATCTCTTTTGCAATCCTTGTCCATGACATTTTTCTTGCATACTAAGCTTGCCGTTTTAAAAGAC
GAATTGTTAATCAGCAAGTTTAGTTTGGGGCCAAAATCAGTAAGCCAGCATGTATGGGATGAGATTTCCCTGG
CTTTTGGAAACGCATTTCCATCGATCAAGGAGAGACTGCTAAATCGGAAACTAATTAAAGTGTCGGGAGACGC
ATTAGAAATCAGGGTGCCTGATTTATATGTGACTTTTCACGATAGATTAGTGACTGAGTACAAAACATCGGTG
GATATGCCAGTGCTTGATATCAGAAAGAGAATGGAGGAGACTGAGGTTATGTACAATGCATTGTCTGAGCTA
TCTGTGCTCAAGGAGTCGGACAAGTTCGACGTTGATGTTTTTTCCCGGATGTGCCAGACTTTGGAGGTAGACC
CAATGACTGCAGCAAAGGTTATTGTGGCAGTGATGAGCAACGAGAGCGGACTGACTCTTACATTCGAACAGC
CAACTGAAGCAAATGTCGCATTGGCACTTAAAGATTCAGAAAAAGCCTCTGAGGGTGCACTAGTGGTTACTTC
TAGAGATGTTGAAGAACCATCCATGAAGGGTTCAATGGCAAGAGGAGAGTTACAATTGGCCGGTCTGTCTGG
AGACCAACCAGAGTCTTCCTATACTCGGAACGAGGAAATAGAGTCGTTAGAGCAATTCCACATGGCAACGGC
TGGTTCGTTAATTCGGAAACAGATGAGTTCGATTGTGTACACGGGCCCCATTAAAGTTCAGCAAATGAAAAAC
TTTATTGATAGCCTGGTAGCATCACTCTCTGCTGCGGTGTCGAACCTAGTCAAGATCCTAAAGGATACAGCTGC
TATAGATCTCGAAACCCGTCAGAAGTTTGGAGTCTTAGATGTTGCGACCAAAAGATGGTTAATTAAACCTTTA
GCCAAGAATCACGCATGGGGCGTTATTGAAACACATGCTAGGAAGTACCACGTTGCACTTTTGGAGTATGATG
AGCATGGAGTGGTAACTTGCGACAGTTGGAGAAGGGTGGCCGTGAGTTCTGAGTCAATGGTTTATTCTGATA
TGGCAAAGCTCAGAACACTGAGGAGATTATTAAGAGATGGTGAGCCTCATGTCAGCAGTGCTAAAGTCGTCC
TAGTTGACGGTGTCCCGGGTTGTGGAAAGACAAAAGAGATTCTCTCGAAAGTAAATTTTGAGGAAGATCTAA
TCTTAGTACCGGGTAAGCAGGCTGCTGAAATGATAAAGAGGCGTGCTAATGCGTCAGGAATAATTCAAGCCA
CAAGAGATAATGTTCGTACTGTTGATTCATTTATAATGAATTACGGTAAAGGAACACGCTGTCAGTTCAAAAG
GTTATTTATCGACGAAGGTCTGATGTTGCACACTGGTTGTGTGAATTTTCTTGTTTCTATGTCTCTGTGCGAAAT
TGCATATGTTTATGGAGACACACAACAAATTCCATACATCAACAGAGTATCCGGTTTTCCGTACCCTGCACATT

TTGCAAAAATAGAGGTTGATGAGGTGGAAACTCGCAGAACTACGCTGCGTTGTCCAGCCGACATTACCCACTA
TCTTAACAGAAGGTACGAAGGACATGTCATGTGTACATCGTCGGTTAAAAAGTCAGTTTCTCAGGAAATGGTG
AGCGGGGCCGCAATGATCAATCCTGTATCTAAGCCATTGAATGGGAAAGTTTTGACTTTCACTCAGTCTGATA
AAGAGGCGCTGCTTTCTCGAGGATATACGGACGTCCATACAGTACATGAGGTACAAGGTGAGACATATGCAG
ATGTGTCGTTGGTCAGATTGACTCCGACACCTATATCTATCATCGCAGGAGATAGTCCGCACGTTCTCGTAGCT
TTGTCAAGGCATACCCAAACATTGAAGTATTACACCGTAGTGATGGATCCTCTTGTAAGTATAATTAGGGATTT
AGAAAAACTTAGTTCTTACTTGTTAGATATGTATAAAGTAGATGCAGGGACCCAATAGCAATTACAGGTAGAC
TCCGTGTTTAAAGGTTCTAATCTTTTTGTTGCAGCACCAAAGACTGGAGATATCTCAGATATGCAATTTTACTAT
GATAAGTGTCTCCCAGGTAATAGCACCATGTTAAATAACTATGATGCTGTTACCATGAGGTTGACTGACATTTC
TCTTAATGTCAAAGATTGCATATTGGATTTCTCTAAGTCTGTGGCTGCACCGAAGGATCCGATCAAACCACTGA
TTCCAATGGTACGAACGGCGGCAGAAATGCCACGCCAGACTGGACTATTGGAAAATTTGGTGGCGATGATCA
AAAGAAACTTTAATTCACCGGAGTTATCAGGAATAATCGACATTGAGAATACTGCATCTTTAGTAGTAGATAA
ATTTTTTGATAGTTACTTGCTTAAAGAAAAAAGAAAACCAAATAAAAATGTTTCTTTATTTTGTAGAGAGTCTCT
CAATAGATGGTTAGAGAAGCAGGAGCAAGTGACCATTGGTCAGCTTGCAGATTTTGATTTTGTGGATCTTCCT
GCCGTTGATCAGTACAGGCATATGATTAAAGCGCAACCTAAGCAGAAGCTGGATACATCAATTCAAAGCGAAT
ATCCGGCCTTGCAGACGATTGTGTATCATTCGAAAAAGATCAACGCAATCTTCGGTCCTTTGTTCAGTGAGCTC
ACAAGGCAAATGCTCGAAAGCATAGACTCAAGTAAGTTTTTGTTCTTTACAAGGAAGACGCCAGCTCAAATTG
AGGATTTCTTCGGAGATCTCGATAGCCATGTCCCTATGGATATCTTGGAGTTGGATATTTCGAAGTATGACAAA
TCTCAGAACGAGTTCCACTGTGCAGTAGAGTATGAAATATGGAGAAGACTTGGATTAGAAGATTTTCTGGGA
GAAGTTTGGAAACAAGGCCACAGGAAAACTACTCTTAAAGATTACACAGCTGGTATTAAAACGTGTTTATGGT
ACCAGAGAAAGAGTGGGGACGTTACAACATTCATCGGTAATACGGTGATTATTGCTGCTTGTTTAGCTTCCAT
GTTGCCCATGGAGAAAATAATCAAAGGTGCATTTTGCGGAGATGACAGTTTACTATACTTCCCAAAAGGTTGT
GAGTTTCCTGACATACAGCATACAGCCAACCTTATGTGGAATTTCGAGGCTAAGCTATTCAGAAAGCAGTATG
GTTATTTCTGTGGAAGGTACGTGATACATCATGACAGAGGGTGTATTGTTTATTATGACCCTTTGAAGTTGATT
TCTAAACTTGGTGCTAAACACATCAAGGATTGGGATCACTTAGAAGAGTTCAGAAGATCCCTTTGTGATGTTG
CAAATTCGTTGAACAACTGTGCGTATTACACGCAGTTGGACGACGCTGTGAGTGAGGTCCATAAAACCGCACC
CCCGGGTTCGTTTGTATATAAAAGTTTAGTTAAATATCTGTCCGATAAGGTTCTTTTTAGAAGTTTGTTTATAGA
TGGCTCTTGTTAAGGGTAAAGTCAATATTAATGAGTTCATAGACTTGTCAAAATCAGAAAAATTTCTTCCGTCT
ATGTTCACACCTGTTAAGAGTGTCATGATCTCCAAGGTTGATAAGATATTGGTTCATGAAGATGAATCTTTGTC
CGAAGTCAATTTACTCAAAGGTGTAAAACTCATTGATGGTGGCTATGTACATCTTGCTGGTCTTGTGGTGACA
GGTGAATGGAATTTGCCAGATAATTGTCGTGGTGGTGTCAGTGTCTGTTTGGTCGATAAAAGAATGGAGAGA
GCGGACGAGGCAACTCTTGCTTCATACTATACCGCAGCGGCTAAGAAAAGGTTTCAGTTCAAAGTCGTTCCAA
ATTACAACATCACTACCAAGGACGCAAAAAAGGCAGTTTGGCAAGTACTAGTTAATATTAGAAATGTTAAAAT
TGCTGCGGGTTACTGTCCGCTGTCATTAGAATTTGTGTCAGTGTGTATTGTTTATAAAAATATTATAAAACTCG
GTTTGAGAGAGAAAATTACGAGCGTAACGGATGGAGGGCCCATGGAACTATCAGAAGAAGTTGTTGATGAG
TTCATGGAAGAAGTCCCGATGTCTGTAAGGCTTGCAAAATTTCGTTCGAAGACCGGGAAAAAGTTTAGTAGTA
AAAGTGAGAATAATAGTGGTAATAATAGGCCGAAACCAAACAAAAACCAAAGGAAGGAAAAGGGTTTAAAA
GTTAGGGTTGAGAAGGATAATTTAATTGATAATGAATTGGAGACTTACGTCGCCGATTCAGATTCGTATTAAA
TATGTCTTACACAATCGCAACTCCATCGCAATTTGTGTTTTTGTCATCAGCATGGGCCGACCCTATAGAATTAAT
AAATTTATGTACTAATTCACTAGGTAATCAATTCCAAACACAACAAGCTAGAACAACCGTTCAACGGCAATTTA
GCGAAGTGTGGAAACCTGTCCCTCAAGTCACTGTTAGGTTTCCTGACAGTGGTTTTAAGGTGTATAGGTACAA
TGCGGTACTAGATCCTCTAGTTACTGCTTTGTTAGGAGCTTTCGATACTAGAAATAGGATTATAGAAGTCGAAA
ATCAGGCGAACCCGACAACCGCCGAAACGTTAGACGCTACTCGTAGAGTAGATGACGCAACGGTGGCTATAA
GGAGCGCTATAAATAATTTAGTAGTAGAATTGGTCAAAGGAACAGGTTTGTACAATCAGAGCACATTTGAAA
GTGCATCCGGTTTACAATGGTCCTCTGCACCAGCATCTTGA

#NC 028478

1

ATGGCATACACACAGACAGCTACCACATCCGCTTTGCTCGACACTGTCCGAGGTAACAATACCTTGGTCAACG
ATCTTGCGAAGCGGCGTCTTTATGACACAGCGGTCGACGAGTTCAACGCTCGTGATCGCAGGCCCAAAGTAA
ATTTTTCCAAAGTAATAAGTGAGGAACAGACGCTTATTGCTACTAGGGCATATCCAGAATTCCAGATAACCTTC
TATAATACGCAGAACGCCGTGCATTCGCTTGCCGGTGGACTACGATCCTTAGAACTGGAATATCTAATGATGC
AGATCCCGTACGGATCACTCACATATGATATAGGTGGGAATTTTGCATCTCATCTGTTCAAAGGACGGGCATA
TGTTCACTGCTGTATGCCCAATCTTGATGTCCGCGACATAATGCGGCACGAAGGCCAGAAAGACAGTATAGAA
TTATACCTTTCCAGGCTTGAGCGGGGCAACAAAGTTGTCCCAAATTTCCAAAAGGAAGCTTTTGACAGATACG
CTGAAACGCCAGACGAAGTTGTCTGTCACAGTACCTTCCAAACGTGTACGCACCAGCAGGTGGAAAACACAG
GCAGGGTGTATGCTATTGCATTGCACAGTATATACGATATACCTGCTGATGAATTCGGAGCGGCACTTTTAAG
GAAAAATGTCCATGTTTGTTACGCCGCCTTCCACTTTTCCGAGAATTTACTTCTCGAAGATTCACACGTCAACCT
TGACGAAATCAACGCGTGTTTTTCGCGTGATGGAGACAAGCTGACTTTTTCTTTCGCATCTGAGAGCACTTTAA
ATTATTGTCATAGTTATTCTAATATTTTAAAATACGTGTGCAAAACTTACTTCCCGGCATCTAATAGAGAGGTCT
ACATGAAGGAGTTTTTGGTCACCAGGGTTAACACCTGGTTTTGTAAGTTTTCTAGGATAGATACTTTTTTATTAT
ACAAGGGGGTAGCCCACAAAGGTGTAAATAGTGAGCAATTTTACAGCGCAATGGAAGATGCATGGCACTACA
AAAAGACTCTTGCAATGTGTAACAGCGAGAGGATTCTTCTTGAAGATTCCTCATCGGTCAATTACTGGTTCCCA
AAAATGAGAGATATGGTCATAGTTCCTCTATTCGACATATCTCTCGACACCAGTAAAAGGACCCGCAAAGAAG
TCTTAGTGTCAAAGGATTTTGTATTCACAGTTTTAAATCACATTCGCACTTATCAAGCCAAGGCACTTACATACT
CCAATGTTTTATCCTTTGTCGAATCAATTCGTTCAAGGGTAATTATCAACGGAGTGACTGCCAGGTCTGAGTGG
GATGTTGACAAATCTCTTTTGCAATCCTTGTCCATGACATTTTTCTTGCATACTAAGCTTGCCGTTTTAAAAGAC
GAATTGTTAATCAGCAAGTTTAGTTTGGGGCCAAAATCAGTAAGCCAGCATGTATGGGATGAGATTTCCCTGG
CTTTTGGAAACGCATTTCCATCGATCAAGGAGAGACTGCTAAATCGGAAACTAATTAAAGTGTCGGGAGACGC
ATTAGAAATCAGGGTGCCTGATTTATATGTGACTTTTCACGATAGATTAGTGACTGAGTACAAAACATCGGTG
GATATGCCAGTGCTTGATATCAGAAAGAGAATGGAGGAGACTGAGGTTATGTACAATGCATTGTCTGAGCTA
TCTGTGCTCAAGGAGTCGGACAAGTTCGACGTTGATGTTTTTTCCCGGATGTGCCAGACTTTGGAGGTAGACC
CAATGACTGCAGCAAAGGTTATTGTGGCAGTGATGAGCAACGAGAGCGGACTGACTCTTACATTCGAACAGC
CAACTGAAGCAAATGTCGCATTGGCACTTAAAGATTCAGAAAAAGCCTCTGAGGGTGCACTAGTGGTTACTTC
TAGAGATGTTGAAGAACCATCCATGAAGGGTTCAATGGCAAGAGGAGAGTTACAATTGGCCGGTCTGTCTGG
AGACCAACCAGAGTCTTCCTATACTCGGAACGAGGAAATAGAGTCATTAGAGCAATTCCACATGGCAACGGCT
AGTTCGTTAATTCGGAAACAGATGAGTTCGATTGTGTACACGGGCCCCATTAAAGTTCAGCAAATGAAAAACT
TTATTGATAGCCTGGTAGCATCACTCTCTGCTGCGGTGTCGAACCTAGTCAAGATCCTAAAGGATACAGCTGCT
ATAGATCTCGAAACCCGTCAGAAGTTTGGAGTCTTAGATGTTGCGACCAAAAGATGGTTAATTAAACCTTTAG
CCAAGAATCACGCATGGGGCGTTATTGAAACACATGCTAGGAAGTACCACGTTGCACTTTTGGAGTATGATGA
GCATGGAGTGGTAACTTGCGACAGTTGGAGAAGGGTGGCCGTGAGTTCTGAGTCAATGGTTTATTCTGATAT
GGCAAAGCTCAGAACACTGAGGAGATTATTAAGAGATGGTGAGCCTCATGTCAGCAGTGCTAAAGTCGTCCT
AGTTGACGGTGTCCCGGGTTGTGGAAAGACAAAAGAGATTCTCTCGAAAGTAAATTTTGAGGAAGATCTAAT
CTTAGTACCGGGTAAGCAGGCTGCTGAAATGATAAAGAGGCGTGCTAATGCGTCAGGAATAATTCAAGCCAC
AAGAGATAATGTTCGTACTGTTGATTCATTTATAATGAATTACGGTAAAGGAACACGCTGTCAGTTCAAAAGG
TTATTTATCGACGAAGGTCTGATGTTGCACACTGGTTGTGTGAATTTTCTTGTTTCTATGTCTCTGTGCGAAATT
GCATATGTTTATGGAGACACACAACAAATTCCATACATCAACAGAGTATCCGGTTTTCCGTACCCTGCACATTT
TGCAAAAATAGAGGTTGATGAGGTGGAAACTCGCAGAACTACGCTGCGTTGTCCAGCCGACATTACCCACTAT
CTTAACAGAAGGTACGAAGGATATGTCATGTGTACATCGTCGGTTAAAAAGTCAGTTTCTCAGGAAATGGTGA
GCGGGGCCGCAATGATCAATCCTGTATCTAAGCCATTGAATGGGAAAGTTTTGACTTTCACTCAGTCTGATAA
AGAGGCGCTGCTTTCTCGAGGATATACGGACGTCCATACAGTACATGAGGTACAAGGTGAGACATATGCAGA
TGTGTCGTTGGTCAGATTGACTCCGACACCTGTATCTATCATCGCAGGAGATAGTCCGCACGTTCTCGTAGCTT
TGTCAAGGCATACCCAAACATTGAAGTATTACACCGTAGTGATGGATCCTCTTGTAAGTATAATTAGGGATTTA

GAAAAACTTAGTTCTTACTTGTTAGATATGTATAAAGTAGATGCAGGGACCCAATAGCAATTACAGGTAGACT
CCGTGTTTAAAGGTTCTAATCTTTTTGTTGCAGCACCAAAGACTGGAGATATCTCAGATATGCAATTTTACTAT
GATAAGTGTCTCCCAGGTAATAGCACCATGTTAAATAACTATGATGCTGTTACCATGAGGTTGACTGACATTTC
TCTTAATGTCAAAGATTGCATATTGGATTTCTCTAAGTCTGTGGCTGCACCGAAGGATCCGATCAAACCACTGA
TTCCGATGGTACGAACGGCGGCAGAAATGCCACGCCAGACTGGACTATTGGAAAATTTGGTGGCGATGATCA
AAAGAAACTTTAATTCACCGGAGTTATCAGGAATAATCGACATTGAGAATACTGCATCTTTAGTAGTAGATAA
ATTTTTTGATAGTTACTTGCTTAAAGAAAAAAGAAAACCAAATAAAAATGTTTCTTTATTTTGTAGAGAGTCTCT
CAATAGATGGTTAGAGAAGCAGGAGCAAGTGACCATTGGTCAGCTTGCAGATTTTGATTTTGTGGATCTTCCT
GCCGTTGATCAGTACAGGCATATGATTAAAGCGCAACCTAAGCAGAAGCTGGATACATCAATTCAAAGCGAAT
ATCCGGCCTTGCAGACGATTGTGTATCATTCGAAAAAGATCAACGCAATCTTCGGTCCTTTGTTCAGTGAGCTC
ACAAGGCAAATGCTCGAAAGCATAGACTCAAGTAAGTTTTTGTTCTTTACAAGGAAGACGCCAGCTCAAATTG
AGGATTTCTTCGGAGATCTCGATAGCCATGTCCCTATGGATATCTTGGAGTTGGATATTTCGAAGTATGACAAA
TCTCAGAACGAGTTCCACTGTGCAGTAGAGTATGAAATATGGAGAAGACTTGGATTAGAAGATTTTCTGGGA
GAAGTTTGGAAACAAGGCCACAGGAAAACTACTCTTAAAGATTACACAGCTGGTATTAAAACGTGTTTATGGT
ACCAGAGAAAGAGTGGGGACGTTACAACATTCATCGGTAATACGGTGATTATTGCTGCTTGTTTAGCTTCCAT
GTTGCCCATGGAGAAAATAATCAAAGGTGCATTTTGCGGAGATGACAGTTTACTATACTTCCCAAAAGGTTGT
GAGTTTCCTGACATACAGCATACAGCCAACCTTATGTGGAATTTCGAGGCTAAGCTATTCAGAAAGCAGTATG
GTTATTTCTGTGGAAGGTACGTGATACATCATGACAGAGGGTGTATTGTTTATTATGACCCTTTGAAGTTGATT
TCTAAACTTGGTGCTAAACACATCAAGGATTGGGATCACTTAGAAGAGTTCAGAAGATCCCTTTGTGATGTTG
CAAATTCGTTGAACAACTGTGCGTATTACACGCAGTTGGACGACGCTGTGAGTGAGGTCCATAAAACCGCACC
CCCGGGTTCGTTTGTATATAAAAGTTTAGTTAAATATCTGTCCGATAAGGTTCTTTTTAGAAGTTTGTTTATAGA
TGGCTCTTGTTAAGGGTAAAGTCAATATTAATGAGTTCATAGACTTGTCAAAATCAGAAAAATTTCTTCCGTCT
ATGTTCACACCTGTTAAGAGTGTCATGATCTCCAAGGTTGATAAGATATTGGTTCATGAAGATGAATCTTTGTC
CGAAGTCAATTTACTCAAAGGTGTAAAACTCATTGATGGTGGCTATGTACATCTTGCTGGTCTTGTGGTGACA
GGTGAATGGAATTTGCCAGATAATTGTCGTGGTGGTGTCAGTGTCTGTTTGGTCGATAAGAGAATGGAGAGA
GCGGACGAGGCAACTCTTGCTTCATACTATACCGCAGCGGCTAAGAAAAGGTTTCAGTTCAAAGTCGTTCCAA
ATTACAACATCACTACCAAGGACGCAGAAAAGGCAGTTTGGCAAGTACTAGTTAATATTAGAAATGTTAAAAT
TGCTGCGGGTTACTGTCCGCTGTCATTAGAATTTGTGTCAGTGTGTATTGTTTATAAAAATATTATAAAACTCG
GTTTGAGAGAGAAAATTACGAGCGTCACGGATGGAGGGCCCATGGAACTATCAGAAGAAGTTGTTGATGAG
TTCATGGAAGAAGTCCCGATGTCTGTAAGGCTTGCAAAATTTCGTTCGAAGACCGGAAAAAAGTTTAGTAGTA
AAAGTGAGAATAATAGTGGTAATAATAGGCCGAAACCAGACAAAAACCAAAGGAAGGAAAAGGGTTTAAAA
GTTAGGGTTGAGAAGGATAATTTAATTGATAATGAATTGGAGACTTACGTCGCCGATTCAGATTCGTATTAAA
TATGTCTTACACAATCGCAACTCCATCGCAATTTGTGTTTTTGTCATCAGCATGGGCCGACCCTATAGAATTAAT
AAATTTATGTACTAATTCACTAGGTAATCAGTTCCAAACACAACAAGCTAGAACAACCGTTCAACGGCAATTTA
GCGAAGTGTGGAAACCTGTCCCTCAAGTCACTGTTAGGTTTCCTGACAGTGGTTTTAAGGTGTATAGGTACAA
TGCGGTACTAGATCCTCTAGTTACTGCTTTGTTAGGAGCTTTCGATACTAGAAATAGGATTATAGAAGTCGAAA
ATCAGGCGAACCCGACAACCGCCGAAACGTTAGACGCTACTCGTAGAGTAGATGACGCAACGGTGGCTATAA
GGAGCGCTATAAATAATTTAGTAGTAGAATTGGTCAAAGGAACAGGTTTGTACAATCAGAGCACATTTGAAA
GTGCATCCGGTTTACAATGGTCCTCTGCACCTGCATCTTGA

#OK339579

1

ATGGCATACACACAGACAGCTACCACATCCGCTTTGCTCGACACTGTCCGAGGTAACAATACCTTGGTCAACG
ATCTTGCGAAGCGGCGTCTTTATGACACAGCGGTCGACGAGTTCAACGCTCGTGATCGCAGGCCCAAAGTAA

ATTTTTCCAAAGTAATAAGTGAGGAACAGACGCTTATTGCTACTAGGGCATATCCAGAATTCCAGATAACCTTC
TATAATACGCAGAACGCCGTGCATTCGCTTGCCGGTGGACTACGATCCTTAGAACTGGAATATCTAATGATGC
AGATCCCGTACGGATCACTCACATATGATATAGGTGGGAATTTTGCATCTCATCTGTTCAAAGGACGGGCATA
TGTTCACTGCTGTATGCCCAATCTTGATGTCCGCGACATAATGCGGCACGAAGGCCAGAAAGACAGTATAGAA
TTATACCTTTCCAGGCTTGAGCGGGGCAACAAAGTTGTCCCAAATTTCCAAAAGGAAGCTTTTGACAGATACG
CTGAAACGCCAGACGAAGTTGTCTGTCACAGTACCTTCCAAACGTGTACGCACCAGCAGGTGGAAAACACAG
GCAGGGTGTATGCTATTGCATTGCACAGTATATACGATATACCTGCTGATGAATTCGGAGCGGCACTTTTAAG
GAAAAATGTCCATGTTTGTTACGCCGCCTTCCACTTTTCCGAGAATTTACTTCTCGAAGATTCACACGTCAACCT
TGACGAAATCAACGCGTGTTTTTCGCGTGATGGAGACAAGCTGACTTTTTCTTTCGCATCTGAGAGCACTTTAA
ATTATTGTCATAGTTATTCTAATATTTTAAAATACGTGTGCAAAACTTACTTCCCGGCATCTAATAGAGAGGTCT
ACATGAAGGAGTTTTTGGTCACCAGGGTTAACACCTGGTTTTGTAAGTTTTCTAGGATAGATACTTTTTTATTAT
ACAAGGGGGTAGCCCACAAAGGTGTAAATAGTGAGCAATTTTACAGCGCAATGGAAGATGCATGGCACTACA
AAAAGACTCTTGCAATGTGTAACAGCGAGAGGATTCTTCTTGAAGATTCCTCATCGGTCAATTACTGGTTCCCA
AAAATGAGAGATATGGTCATAGTTCCTCTATTCGACATATCTCTCGACACCAGTAAAAGGACCCGCAAAGAAG
TCTTAGTGTCAAAGGATTTTGTATTCACAGTTTTAAATCACATTCGCACTTATCAAGCCAAGGCACTTACATACT
CCAATGTTTTATCCTTTGTCGAATCAATTCGTTCAAGGGTAATTATCAACGGAGTGACTGCCAGGTCTGAGTGG
GATGTTGACAAATCTCTTTTGCAATCCTTGTCCATGACATTTTTCTTGCATACTAAGCTTGCCGTTTTAAAAGAC
GAATTGTTAATCAGCAAGTTTAGTTTAGGGCCAAAATCAGTAAGCCAGCATGTATGGGATGAGATTTCCCTGG
CTTTTGGAAACGCATTTCCATCGATCAAGGAGAGACTGCTAAATCGGAAACTAATTAAAGTGTCGGGAGACGC
ATTAGAAATCAGGGTGCCTGATTTATATGTGACTTTTCACGATAGATTAGTGACTGAGTACAAAACATCGGTG
GATATGCCAGTGCTTGATATCAGAAAGAGAATGGAGGAGACTGAGGTTATGTACAATGCATTGTCTGAGCTA
TCTGTGCTCAAGGAGTCGGACAAGTTCGACGTTGATGTTTTTTCCCGGATGTGCCAGACTTTGGAGGTAGACC
CAATGACTGCAGCAAAGGTTATTGTGGCAGTGATGAGCAACGAGAGCGGACTGACTCTTACATTCGAACAGC
CAACTGAAGCAAATGTCGCATTGGCACTTAAAGATTCAGAAAAAGCCTCTGAGGGTGCACTAGTGGTTACTTC
TAGAGATGTTGAAGAACCATCCATGAAGGGTTCAATGGCAAGAGGAGAGTTACAATTGGCCGGTCTGTCTGG
AGACCAACCAGAGTCTTCCTATACTCGGAACGAGGAAATAGAGTCATTAGAGCAATTCCACATGGCAACGGCT
AGTTCGTTAATTCGGAAACAGATGAGTTCGATTGTGTACACGGGCCCCATTAAAGTTCAGCAAATGAAAAACT
TTATTGATAGCCTGGTAGCATCACTCTCTGCTGCGGTGTCGAACCTAGTCAAGATCCTAAAGGATACAGCTGCT
ATAGATCTCGAAACCCGTCAGAAGTTTGGAGTCTTAGATGTTGCGACCAAAAGATGGTTAATTAAACCTTTAG
CCAAGAATCACGCATGGGGCGTTATTGAAACACATGCTAGGAAGTACCACGTTGCACTTTTGGAGTATGATGA
GCATGGAGTGGTAACTTGCGACAGTTGGAGAAGGGTGGCCGTGAGTTCTGAGTCAATGGTTTATTCTGATAT
GGCAAAGCTCAGAACACTGAGGAGATTATTAAGGGATGGTGAGCCTCATGTCAGCAGTGCTAAAGTCGTCCT
AGTTGACGGTGTCCCGGGTTGTGGAAAGACAAAAGAGATTCTCTCGAAAGTAAATTTTGAGGAAGATCTAAT
CTTAGTACCGGGTAAGCAGGCTGCTGAAATGATAAAGAGGCGTGCTAATGCGTCAGGAATAATTCAAGCCAC
AAGAGATAATGTTCGTACTGTTGATTCATTTATAATGAATTACGGTAAAGGAACACGCTGTCAGTTCAAAAGG
TTATTTATCGACGAAGGTCTGATGTTGCACACTGGTTGTGTGAATTTTCTTGTTTCTATGTCTCTGTGCGAAATT
GCATATGTTTATGGAGACACACAACAAATTCCATACATCAACAGAGTATCCGGTTTTCCGTACCCTGCACATTT
TGCAAAAATAGAGGTTGATGAGGTGGAAACTCGCAGAACTACGCTGCGTTGTCCAGCCGACATTACCCACTAT
CTTAACAGAAGGTACGAAGGACATGTCATGTGTACATCGTCGGTTAAAAAGTCAGTTTCTCAGGAAATGGTGA
GCGGGGCCGCAATGATCAATCCTGTATCTAAGCCACTGAATGGGAAAGTTTTGACTTTCACTCAGTCTGATAA
AGAGGCGCTGCTTTCTCGAGGATATACGGACGTCCATACAGTACATGAGGTACAAGGTGAGACATATGCAGA
TGTGTCGTTGGTCAGATTGACTCCGACACCTGTATCTATCATCGCAGGAGATAGTCCGCACGTTCTCGTAGCTT
TGTCAAGGCATACCCAAACATTGAAGTATTACACCGTAGTGATGGATCCTCTTGTAAGTATAATTAGGGATTTA
GAAAAACTTAGTTCTTACTTGTTAGATATGTATAAAGTAGATGCAGGAACCCAATAGCAATTACAGGTAGACT
CCGTGTTTAAAGGTTCTAATCTTTTTGTTGCAGCACCAAAGACTGGAGATATCTCAGATATGCAATTTTACTAT
GATAAGTGTCTCCCAGGTAATAGCACCATGTTAAATAACTATGATGCTGTTACCATGAGGTTGACTGACATTTC
TCTTAATGTCAAAGATTGCATATTGGATTTCTCTAAGTCTGTGGCTGCACCGAAGGATCCGATCAAACCACTGA
TTCCAATGGTACGAACGGCGGCAGAAATGCCACGCCAGACTGGACTATTGGAAAATTTGGTGGCGATGATCA

AAAGAAACTTTAATTCACCGGAGTTATCAGGAATAATCGACATTGAGAATACTGCATCTTTAGTAGTAGATAA
ATTTTTTGATAGTTACTTGCTTAAAGAAAAAAGAAAACCAAATAAAAATGTTTCTTTATTTTGTAGAGAGTCTCT
CAATAGATGGTTAGAGAAGCAGGAGCAAGTGACCATTGGTCAGCTTGCAGATTTTGATTTTGTGGATCTTCCT
GCCGTTGATCAGTACAGGCATATGATTAAAGCGCAACCTAAGCAGAAGCTGGATACATCAATTCAAAGCGAAT
ATCCGGCCTTGCAGACGATTGTGTATCATTCGAAAAAGATCAACGCAATCTTCGGTCCTTTGTTCAGTGAGCTC
ACAAGGCAAATGCTCGAAAGCATAGACTCAAGTAAGTTTTTGTTCTTTACAAGGAAGACGCCAGCTCAAATTG
AGGATTTCTTCGGAGATCTCGATAGCCATGTCCCTATGGATATCTTGGAGTTGGATATTTCGAAGTATGACAAA
TCTCAGAACGAGTTCCACTGTGCAGTAGAGTATGAAATATGGAGAAGACTTGGATTAGAAGATTTTCTGGGA
GAAGTTTGGAAACAAGGCCACAGGAAAACTACTCTTAAAGATTACACAGCTGGTATAAAAACGTGTTTGTGGT
ACCAGAGAAAGAGTGGGGACGTTACAACATTCATCGGTAATACGGTGATTATTGCTGCTTGTTTAGCTTCCAT
GTTGCCCATGGAGAAAATAATCAAAGGTGCATTTTGCGGAGATGACAGTTTACTATACTTCCCAAAAGGTTGT
GAGTTTCCTGACATACAGCATACAGCCAACCTTATGTGGAATTTCGAGGCTAAGCTATTCAGAAAGCAGTATG
GTTATTTCTGTGGAAGGTACGTGATACATCATGACAGAGGGTGTATTGTTTATTATGACCCTTTGAAGTTGATT
TCTAAACTTGGTGCTAAACACATCAAGGATTGGGATCACTTAGAAGAGTTCAGAAGATCCCTTTGTGATGTTG
CAAATTCGTTGAACAACTGTGCGTATTACATGCAGTTGGACGACGCTGTGAGTGAGGTCCATAAAACCGCACC
CCCGGGTTCGTTTGTGTATAAAAGTTTAGTTAAATATCTGTCCGATAAGGTTCTTTTTAGAAGTTTGTTTATAGA
TGGCTCTTGTTAAGGGTAAAGTCAATATTAATGAGTTCATAGACTTGTCAAAATCAGAAAAATTTCTTCCGTCT
ATGTTCACACCTGTTAAGAGTGTCATGATCTCCAAGGTTGATAAGATATTGGTTCATGAAGATGAATCTTTGTC
CGAAGTCAATTTACTCAAAGGTGTAAAACTCATTGATGGTGGCTATGTACATCTTGCTGGTCTTGTGGTGACA
GGTGAATGGAATTTGCCAGATAATTGTCGTGGTGGTGTCAGTGTCTGTTTGGTCGATAAGAGAATGGAGAGA
GCGGACGAGGCAACTCTTGCTTCATACTATACCGCAGCGGCTAAGAAAAGGTTTCAGTTCAAAGTCGTTCCAA
ATTACAACATCACTACCAAGGACGCAGAAAAGGCAGTTTGGCAAGTACTAGTTAATATTAGAAATGTTAAAAT
TGCTGCGGGTTACTGTCCGCTGTCATTAGAATTTGTGTCAGTGTGTATTGTTTATAAAAATATTATAAAACTCG
GTTTGAGAGAGAAAATTACGAGCGTCACGGATGGAGGGCCCATGGAACTATCAGAAGAAGTTGTTGATGAG
TTCATGGAAGAAGTCCCGATGTCTGTAAGGCTTGCAAAATTTCGTTCGAAGACCGGAAAAAAGTTTAGTAGTA
AAAGTGAGAATAATAGTGGTAATAATAGGCCGAAACCAAACAAAAACCAAAGGAAGGAAAAGGGTTTAAAA
GTTAGGGTTGAGAAGGATAATTTAATTGATAATGAATTGGAGACTTACATCGCCGATTCAGATTCGTATTAAA
TATGTCTTACACAATCGCAACTCCATCGCAATTTGTGTTTTTGTCATCAGCATGGGCCGACCCTATAGAATTAAT
AAATTTATGTACTAATTCACTAGGAAATCAGTTCCAAACACAACAAGCTAGAACAACCGTTCAACGGCAATTTA
GCGAAGTGTGGAAACCTGTCCCTCAAGTCACTGTTAGGTTTCCTGACAGTGGTTTTAAGGTGTATAGGTACAA
TGCGGTACTAGATCCTCTAGTTACTGCTTTGTTAGGAGCTTTCGATACTAGAAATAGGATTATAGAAGTCGAAA
ATCAGGCGAACCCGACAACCGCCGAAACGTTAGACGCTACTCGTAGAGTAGATGACGCAACGGTGGCTATAA
GGAGCGCTATAAATAATTTAGTAGTAGAATTGGTCAAAGGAACAGGTTTGTACAATCAGAGCACATTTGAAA
GTGCATCCGGTTTACAATGGTCCTCTGCACCTGCATCTTGA

#OK624678

1

ATGGCATACACACAGACAGCTACCACATCCGCTTTGCTCGACACTGTCCGAGGTAACAATACCTTGGTCAACG
ATCTTGCGAAGCGGCGTCTTTATGACACAGCGGTCGACGAGTTCAACGCTCGTGATCGCAGGCCCAAAGTAA
ATTTTTCCAAAGTAATAAGTGAGGAACAGACGCTTATTGCTACTAGGGCATATCCAGAATTCCAGATAACCTTC
TATAATACGCAGAACGCCGTGCATTCGCTTGCCGGTGGACTACGATCCTTAGAACTGGAATATCTAATGATGC
AGATCCCGTACGGATCACTCACATATGATATAGGTGGGAATTTTGCATCTCATCTGTTCAAAGGACGGGCATA
TGTTCACTGCTGTATGCCCAATCTTGATGTCCGCGACATAATGCGGCACGAAGGCCAGAAAGACAGTATAGAA
TTATACCTTTCCAGGCTTGAGCGGGGCAACAAAGTTGTCCCAAATTTCCAAAAGGAAGCTTTTGACAGATACG

CTGAAACGCCAGACGAAGTTGTCTGTCACAGTACCTTCCAAACGTGTACGCACCAGCAGGTGAAAAACACAG
GCAGGGTGTATGCTATTGCATTGCACAGTATATACGATATACCTGCTGATGAATTCGGAGCGGCACTTTTAAG
GAAAAATGTCCATGTTTGTTACGCCGCCTTCCACTTTTCCGAGAATTTACTTCTCGAAGATTCACACGTCAACCT
TGACGAAATCAACGCGTGTTTTTCGCGTGATGGAGACAAGCTGACTTTTTCTTTCGCATCTGAGAGCACTTTAA
ATTATTGTCATAGTTATTCTAATATTTTAAAATACGTGTGCAAAACTTACTTCCCGGCATCTAATAGAGAGGTCT
ACATGAAGGAGTTTTTGGTCACCAGGGTTAACACCTGGTTTTGTAAGTTTTCTAGGATAGATACTTTTTTATTAT
ACAAGGGGGTAGCCCACAAAGGTGTAAATAGTGAGCAATTTTACAACGCAATGGAAGATGCATGGCACTACA
AAAAGACTCTTGCAATGTGTAACAGCGAGAGGATTCTTCTTGAAGATTCCTCATCGGTCAATTACTGGTTCCCA
AAAATGAGAGATATGGTCATAGTTCCTCTATTCGACATATCTCTCGACACCAGTAAAAGGACCCGCAAAGAAG
TCTTAGTGTCAAAGGATTTTGTATTCACAGTTTTAAATCACATTCGCACTTATCAAGCCAAGGCACTTACATACT
CCAATGTTTTATCCTTTGTCGAATCAATTCGTTCAAGGGTAATTATCAACGGAGTGACTGCCAGGTCTGAGTGG
GATGTTGACAAATCTCTTTTGCAATCCTTGTCCATGACATTTTTCTTGCATACTAAGCTTGCCGTTTTAAAAGAC
GAATTGTTAATCAGCAAGTTTAGTTTGGGGCCAAAATCAGTAAGCCAGCATGTATGGGATGAGATTTCCCTGG
CTTTTGGAAACGCATTTCCATCGATCAAGGAGAGACTGCTAAATCGGAAACTAATTAAAGTGTCGGGAGACGC
ATTAGAAATCAGGGTGCCTGATTTATATGTGACTTTCCACGATAGATTAGTGACTGAGTACAAAACATCGGTG
GATATGCCAGTGCTTGATATCAGAAAGAGAATGGAGGAGACTGAGGTTATGTACAATGCATTGTCTGAGCTA
TCTGTGCTCAAGGAGTCGGACAAGTTCGACGTTGATGTTTTTCCCGGATGTGCCAGACTTTGGAGGTAGACC
CAATGACTGCAGCAAAGGTTATTGTGGCAGTGATGAGCAACGAGAGCGGACTGACTCTTACATTCGAACAGC
CAACTGAAGCAAATGTCGCATTGGCACTTAAAGATTCAGAAAAAGCCTCTGAGGGTGCACTAGTGGTTACTTC
TAGAGATGTTGAAGAACCATCCATGAAGGGTTCAATGGCAAGAGGAGAGTTACAATTGGCCGGTCTGTCTGG
AGACCAACCAGAGTCTTCCTATACTCGGAACGAGGAAATAGAGTCATTAGAGCAATTCCACATGGCAACGGCT
AGTTCGTTAATTCGGAAACAGATGAGTTCGATTGTGTACACGGGCCCCATTAAAGTTCAGCAAATGAAAAACT
TTATTGATAGCCTGGTAGCATCACTCTCTGCTGCGGTGTCGAACCTAGTCAAGATCCTAAAGGATACAGCTGCT
ATAGACCTCGAAACCCGTCAGAAGTTTGGAGTCTTAGATGTTGCGACCAAAAGATGGTTAATTAAACCTTTAG
CCAAGAATCACGCATGGGGCGTTATTGAAACACATGCTAGGAAGTACCACGTTGCACTTTTGGAGTATGATGA
GCATGGAGTGGTAACTTGCGACAGTTGGAGAAGGGTGGCCGTGAGTTCTGAGTCAGTGGTTTATTCTGATAT
GGCAAAGCTCAGAACACTGAGGAGATTATTAAGAGATGGTGAGCCTCATGTCAGCAGTGCTAAAGTCGTCCT
AGTTGACGGTGTCCCGGGTTGTGGAAAGACAAAAGAGATTCTCTCGAAAGTAAATTTTGAGGAAGATCTAAT
CTTAGTACCGGGTAAGCAGGCTGCTGAAATGATAAAGAGGCGTGCTAATGCGTCAGGAATAATTCAAGCCAC
AAGAGATAATGTTCGTACTGTTGATTCATTTATAATGAATTACGGTAAAGGAACACGCTGTCAGTTCAAAAGG
TTATTTATCGACGAAGGTCTGATGTTGCACACTGGTTGTGTGAATTTTCTTGTTTCTATGTCTCTGTGCGAAATT
GCATATGTTTATGGAGACACACAACAAATTCCATACATCAACAGAGTATCCGGTTTTCCGTACCCTGCACATTT
TGCAAAAATAGAGGTTGATGAGGTGGAAACTCGCAGAACTACGCTGCGTTGTCCAGCCGACATTACCCACTAT
CTTAACAGAAGGTACGAAGGACATGTCATGTGTACATCGTCGGTTAAAAAGTCAGTTTCTCAGGAAATGGTGA
GCGGGGCCGCAATGATCAATCCTGTATCTAAGCCACTGAATGGGAAAGTTTTGACTTTCACTCAGTCTGATAA
AGAGGCGCTGCTTTCTCGAGGATATACGGACGTCCATACAGTACATGAGGTACAAGGTGAGACATATGCAGA
TGTGTCGTTGGTCAGATTGACTCCGACACCTGTATCTATCATCGCAGGAGATAGTCCACACGTTCTCGTAGCTT
TGTCAAGGCATACCCAAACATTGAAGTATTACACCGTAGTGATGGATCCTCTTGTAAGTATAATTAGGGATTTA
GAAAAACTTAGTTCTTACTTGTTAGATATGTATAAAGTAGATGCAGGGACCCAATAGCAATTACAGGTAGACT
CCGTGTTTAAAGGTTCTAATCTTTTTGTTGCAGCACCAAAGACTGGAGATATCTCAGATATGCAATTTTACTAT
GATAAGTGTCTCCCAGGTAATAGCACCATGTTAAATAACTATGATGCTGTTACCATGAGGTTGACTGACATTTC
TCTTAATGTCAAAGATTGCATATTGGATTTCTCTAAGTCTGTGGCTGCACCGAAGGATCCGATCAAACCACTGA
TTCCAATGGTACGAACGGCGGCAGAAATGCCACGCCAGACTGGACTATTGGAAAATTTGGTGGCGATGATCA
AAAGAAACTTTAATTCACCGGAGTTATCAGGAATAATCGACATTGAGAATACTGCATCTTTAGTAGTAGATAA
ATTTTTTGATAGTTACTTGCTTAAAGAAAAAAGAAAACCAAATAAAAATGTTTCTTTATTTTGTAGAGAGTCTCT
CAATAGATGGTTAGAGAAGCAGGAGCAAGTGACCATTGGTCAGCTTGCAGATTTTGATTTTGTGGATCTTCCT
GCCGTTGATCAGTACAGGCATATGATTAAAGCGCAACCTAAGCAGAAGCTGGATACATCAATTCAAAGCGAAT
ATCCGGCCTTGCAGACGATTGTGTATCATTCGAAAAAGATCAACGCAATCTTCGGTCCTTTGTTCAGTGAGCTC

ACAAGGCAAATGCTCGAAAGCATAGACTCAAGTAAGTTTTTGTTCTTTACAAGGAAGACGCCAGCTCAAATTG
AGGATTTCTTCGGAGATCTCGATAGCCATGTCCCTATGGATATCTTGGAGTTGGATATTTCGAAGTATGACAAA
TCTCAGAACGAGTTCCACTGTGCAGTAGAGTATGAAATATGGAGAAGACTTGGATTAGAAGATTTTCTGGGA
GAAGTTTGGAAACAAGGCCACAGGAAAACTACTCTTAAAGATTACACAGCTGGTATTAAAACGTGTTTATGGT
ACCAGAGAAAGAGTGGGGACGTTACAACATTCATCGGTAATACGGTGATTATTGCTGCTTGTTTAGCTTCCAT
GTTGCCCATGGAGAAAATAATCAAAGGTGCATTTTGCGGAGATGACAGTTTACTATACTTCCCAAAAGGTTGT
GAGTTTCCTGACATACAGCATACAGCCAACCTTATGTGGAATTTCGAGGCTAAGCTATTCAGAAAGCAGTATG
GTTATTTCTGTGGAAGGTACGTGATACATCATGACAGAGGGTGTATTGTTTATTATGACCCTTTGAAGTTGATT
TCTAAACTTGGTGCTAAACACATCAAGGATTGGGATCACTTAGAAGAGTTCAGAAGATCCCTTTGTGATGTTG
CAAATTCGTTGAACAACTGTGCGTATTACACGCAGTTGGACGACGCTGTGAGTGAGGTCCATAAAACCGCACC
CCCGGGTTCGTTTGTGTATAAAAGTTTAGTTAAATATCTGTCCGATAAGGTTCTTTTTAGAAGTTTGTTTATAGA
TGGCTCTTGTTAAGGGTAAAGTCAATATTAATGAGTTCATAGACTTGTCAAAATCAGAAAAATTTCTTCCGTCT
ATGTTCACACCTGTTAAGAGTGTCATGATCTCCAAGGTTGATAAGATATTGGTTCATGAAGATGAATCTTTGTC
CGAAGTCAATTTACTCAAAGGTGTAAAACTCATTGATGGTGGCTATGTACATCTTGCTGGTCTTGTGGTGACA
GGTGAATGGAATTTGCCAGATAATTGTCGTGGTGGTGTCAGTGTCTGTTTGGTCGATAAGAGAATGGAGAGA
GCGGACGAGGCAACTCTTGCTTCATACTATACCGCAGCGGCTAAGAAAAGGTTTCAGTTCAAAGTCGTTCCAA
ATTACAACATCACTACCAAGGACGCAGAAAAGGCAGTTTGGCAAGTACTAGTTAATATTAGAAATGTTAAAAT
TGCTGCGGGTTACTGTCCGCTGTCATTAGAATTTGTGTCAGTGTGTATTGTTTATAAAAATATTATAAAACTCG
GTTTGAGAGAGAAAATTACGAGCGTCACGGATGGAGGGCCCATGGAACTATCAGAAGAAGTTGTTGATGAG
TTCATGGAAGAAGTCCCGATGTCTGTAAGGCTTGCAAAATTTCGTTCGAAGACCGGAAAAAAGTTTAGTAGTA
AAAGTGAGAATAATAGTGGTAATAATAGGCCGAAACCAAACAAAAACCAAAGGAAGGAAAAGGGTTTAAAA
GTTAGGGTTGAGAAGGATAATTTAATTGATAATGAATTGGAGACTTACATCGCCGATTCAGATTCGTATTAAA
TATGTCTTACACAATCGCAACTCCATCGCAATTTGTGTTTTTGTCATCAGCATGGGCCGACCCTATAGAATTAAT
AAATTTATGTACTAATTCACTAGGTAATCAGTTCCAAACACAACAAGCTAGAACAACCGTTCAACGGCAATTTA
GCGAAGTGTGGAAACCTGTCCCTCAAGTCACTGTTAGGTTTCCTGACAGTGGTTTTAAGGTGTATAGGTACAA
TGCGGTACTAGATCCTCTAGTTACTGCTTTGTTAGGAGCTTTCGATACTAGAAATAGGATTATAGAAGTCGAAA
ATCAGGCGAACCCGACAACCGCCGAAACGTTAGACGCTACTCGTAGAGTAGATGACGCAACGGTGGCTATAA
GGAGCGCTATAAATAATTTAGTAGTAGAATTGGTCAAAGGAACAGGTTTGTACAATCAGAGCACATTTGAAA
GTGCATCCGGTTTACAATGGTCCTCTGCACCTGCATCTTGA

#OM515232

1

ATGGCATACACACAGACAGCTACCACATCCGCTTTGCTCGACACTGTCCGAGGTAACAATACCTTGGTCAATG
ATCTTGCGAAGCGGCGTCTTTATGACACAGCGGTCGACGAGTTCAACGCTCGTGATCGCAGGCCAAAAGTAA
ATTTTTCCAAAGTAATAAGTGAGGAACAGACGCTTATTGCTACTAGGGCATATCCAGAATTCCAGATAACCTTC
TATAATACGCAGAACGCCGTGCATTCGCTTGCCGGTGGACTACGATCCTTAGAACTGGAATATCTAATGATGC
AGATCCCGTACGGATCACTCACATATGATATAGGTGGGAATTTTGCATCTCATCTGTTCAAAGGACGGGCATA
TGTTCACTGCTGTATGCCCAATCTTGATGTCCGCGACATAATGCGGCACGAAGGCCAGAAAGACAGTATAGAA
TTATACCTTTCCAGGCTTGAGCGGGGCAACAAAGTTGTCCCAAATTTCCAAAAGGAAGCTTTTGACAGATACG
CTGAAACGCCAGACGAAGTTGTCTGTCACAGTACCTTCCAAACGTGTACGCACCAGCAGGTGGAAAACACAG
GCAGGGTGTATGCTATTGCATTGCACAGTATATACGATATACCTGCTGATGAATTCGGAGCGGCACTTTTAAG
GAAAAATGTCCATGTTTGTTACGCCGCCTTCCACTTTTCCGAGAATTTACTTCTCGAAGATTCACACGTCAACCT
TGACGAAATCAACGCGTGTTTTTCGCGTGATGGAGACAAGCTGACTTTTTCTTTCGCATCTGAGAGCACTTTAA
ATTATTGTCATAGTTATTCTAATATTTTAAAATACGTGTGCAAAACTTACTTCCCGGCATCTAATAGAGAGGTCT

ACATGAAGGAGTTTTTGGTCACCAGGGTTAACACCTGGTTTTGTAAGTTTTCTAGGATAGATACTTTTTTATTAT
ACAAGGGGGTAGCCCACAAAGGTGTAAATAGTGAGCAATTTTACAGCGCAATGGAAGATGCATGGCACTACA
AAAAGACTCTTGCAATGTGTAACAGCGAGAGGATTCTTCTTGAAGATTCCTCATCGGTCAATTACTGGTTCCCA
AAAATGAGAGATATGGTCATAGTTCCTCTATTCGACATATCTCTCGACACCAGTAAAAGGACCCGCAAAGAAG
TCTTAGTGTCAAAGGATTTTGTATTCACAGTTTTAAATCACATTCGCACTTATCAAGCCAAGGCACTTACATACT
CCAATGTTTTATCCTTTGTCGAATCAATTCGTTCAAGGGTAATTATCAACGGAGTGACTGCCAGGTCTGAGTGG
GATGTTGACAAATCTCTTTTGCAATCCTTGTCCATGACATTTTTCTTGCATACTAAGCTTGCCGTTTTAAAAGAC
GAATTGTTAATCAGCAAGTTTAGTTTGGGGCCAAAATCAGTAAGCCAGCATGTATGGGATGAGATTTCCCTGG
CTTTTGGAAACGCATTTCCATCGATCAAGGAGAGACTGCTAAATCGGAAACTAATTAAAGTGTCGGGAGACGC
ATTAGAAATCAGGGTGCCTGATTTATATGTGACTTTTCACGATAGATTAGTGACTGAGTACAAAACATCGGTG
GATATGCCAGTGCTTGATATCAGAAAGAGAATGGAGGAGACTGAGGTTATGTACAATGCATTGTCTGAGCTA
TCTGTGCTCAAGGAGTCGGACAAGTTCGACGTTGATGTTTTTTCCCGGATGTGCCAGACTTTGGAGGTAGACC
CAATGACTGCAGCAAAGGTTATTGTGGCAGTGATGAGCAACGAGAGCGGACTGACTCTTACATTCGAACAGC
CAACTGAAGCAAATGTCGCATTGGCACTTAAAGATTCAGAAAAAGCCTCTGAGGGTGCACTAGTGGTTACTTC
TAGAGATGTTGAAGAACCATCCATGAAGGGTTCAATGGCAAGAGGAGAGTTACAATTGGCCGGTCTGTCTGG
AGACCAACCAGAGTCTTCCTATACTCGGAACGAGGAAATAGAGTCATTAGAGCAATTCCACATGGCAACGGCT
AGTTCGTTAATTCGGAAACAGATGAGTTCGATTGTGTACACGGGCCCCATTAAAGTTCAGCAAATGAAAAACT
TTATTGATAGCCTGGTAGCATCACTCTCTGCTGCGGTGTCGAACCTAGTCAAGATCCTAAAGGATACAGCTGCT
ATAGACCTCGAAACCCGTCAGAAGTTTGGAGTCTTAGATGTTGCGACCAAAAGATGGTTAATTAAACCTTTAG
CCAAGAATCACGCATGGGGCGTTATTGAAACACATGCTAGGAAGTACCACGTTGCACTTTTGGAGTATGATGA
GCATGGAGTGGTAACTTGCGACAGTTGGAGAAGGGTGGCCGTGAGTTCTGAGTCAGTGGTTTATTCTGATAT
GGCAAAGCTCAGAACACTGAGGAGATTATTAAGAGATGGTGAGCCTCATGTCAGCAGTGCTAAAGTCGTCCT
AGTTGACGGTGTCCCGGGTTGTGGAAAGACAAAAGAGATTCTCTCGAAAGTAAATTTTGAGGAAGATCTAAT
CTTAGTACCGGGTAAGCAGGCTGCTGAAATGATAAAGAGGCGTGCTAATGCGTCAGGAATAATTCAAGCCAC
AAGAGATAATGTTCGTACTGTTGATTCATTTATAATGAATTACGGTAAAGGAACACGCTGTCAGTTCAAAAGG
TTATTTATCGACGAAGGTCTGATGTTGCACACTGGTTGTGTGAATTTTCTTGTTTCTATGTCTCTGTGCGAAATT
GCATATGTTTATGGAGACACACAACAAATTCCATACATCAACAGAGTATCCGGTTTTCCGTACCCTGCACATTT
TGCAAAAATAGAGGTTGATGAGGTGGAAACTCGCAGAACTACGCTGCGTTGTCCAGCCGACATTACCCACTAT
CTTAACAGAAGGTACGAAGGACATGTCATGTGTACATCGTCGGTTAAAAAGTCAGTTTCTCAGGAAATGGTGA
GCGGGGCCGCAATGATCAATCCTGTATCTAAGCCACTGAATGGGAAAGTTTTGACTTTCACTCAGTCTGATAA
AGAGGCGCTGCTTTCTCGAGGATATACGGACGTCCATACAGTACATGAGGTACAAGGTGAGACATATGCAGA
TGTGTCGTTGGTCAGATTGACTCCGACACCTGTATCTATCATCGCAGGAGATAGTCCGCACGTTCTCGTAGCTT
TGTCAAGGCATACCCAAACATTGAAGTATTACACCGTAGTGATGGATCCTCTTGTAAGTATAATTAGGGATTTA
GAAAAACTTAGTTCTTACTTGTTAGATATGTATAAAGTAGATGCAGGGACCCAATAGCAATTACAGGTAGACT
CCGTGTTTAAAGGTTCTAATCTTTTTGTTGCAGCACCAAAGACTGGAGATATCTCAGATATGCAATTTTACTAT
GATAAGTGTCTCCCAGGTAATAGCACCATGTTAAATAACTATGATGCTGTTACCATGAGGTTGACTGACATTTC
TCTTAATGTCAAAGATTGCATATTGGATTTCTCTAAGTCTGTGGCTGCACCGAAGGATCCGATCAAACCACTGA
TTCCAATGGTACGAACGGCGGCAGAAATGCCACGCCAGACTGGACTATTGGAAAATTTGGTGGCGATGATCA
AAAGAAACTTTAATTCACCGGAGTTATCAGGAATAATCGACATTGAGAATACTGCATCTTTAGTAGTAGATAA
ATTTTTTGATAGTTACTTGCTTAAAGAAAAAAGAAAACCAAATAAAAATGTTTCTTTATTTTGTAGAGAGTCTCT
CAATAGATGGTTAGAGAAGCAGGAGCAAGTGACCATTGGTCAGCTTGCAGATTTTGATTTTGTGGATCTTCCT
GCCGTTGATCAGTACAGGCATATGATTAAAGCGCAACCTAAGCAGAAGCTGGATACATCAATTCAAAGCGAAT
ATCCGGCCTTGCAGACGATTGTGTATCATTCGAAAAAGATCAACGCAATCTTCGGTCCTTTGTTCAGTGAGCTC
ACAAGGCAAATGCTCGAAAGCATAGACTCAAGTAAGTTTTTGTTCTTTACAAGGAAGACGCCAGCTCAAATTG
AGGATTTCTTCGGAGATCTCGATAGCCATGTCCCTATGGATATCTTGGAGTTGGATATTTCGAAGTATGACAAA
TCTCAGAACGAGTTCCACTGTGCAGTAGAGTATGAAATATGGAGAAGACTTGGATTAGAAGATTTTCTGGGA
GAAGTTTGGAAACAAGGCCACAGGAAAACTACTCTTAAAGATTACACAGCTGGTATTAAAACGTGTTTATGGT
ACCAGAGAAAGAGTGGGGACGTTACAACATTCATCGGTAATACGGTGATTATTGCTGCTTGTTTAGCTTCCAT

GTTGCCCATGGAGAAAATAATCAAAGGTGCATTTTGCGGAGATGACAGTTTACTATACTTCCCAAAAGGTTGT
GAGTTTCCTGACATACAGCATACAGCCAACCTTATGTGGAATTTCGAGGCTAAGCTATTCAGAAAGCAGTATG
GTTATTTCTGTGGAAGGTACGTGATACATCATGACAGAGGGTGTATTGTTTATTATGACCCTTTGAAGTTGATT
TCTAAACTTGGTGCTAAACACATCAAGGATTGGGATCACTTAGAAGAGTTCAGAAGATCCCTTTGTGATGTTG
CAAATTCGTTGAACAACTGTGCGTATTACACGCAGTTGGACGACGCTGTGAGTGAGGTCCATAAAACCGCACC
CCCGGGTTCGTTTGTGTATAAAAGTTTAGTTAAATATCTGTCCGATAAGGTTCTTTTTAGAAGTTTGTTTATAGA
TGGCTCTTGTTAAGGGTAAAGTCAATATTAATGAGTTCATAGACTTGTCAAAATCAGAAAAATTTCTTCCGTCT
ATGTTCACACCTGTTAAGAGTGTCATGATCTCCAAGGTTGATAAGATATTGGTTCATGAAGATGAATCTTTGTC
CGAAGTCAATTTACTCAAAGGTGTAAAACTCATTGATGGTGGCTATGTACATCTTGCTGGTCTTGTGGTGACA
GGTGAATGGAATTTGCCAGATAATTGTCGTGGTGGTGTCAGTGTCTGTTTGGTCGATAAGAGAATGGAGAGA
GCGGACGAGGCAACTCTTGCTTCATACTATACCGCAGCGGCTAAGAAAAGGTTTCAGTTCAAAGTCGTTCCAA
ATTACAACATCACTACCAAGGACGCAGAAAAGGCAGTTTGGCAAGTACTAGTTAATATTAGAAATGTTAAAAT
TGCTGCGGGTTACTGTCCGCTGTCATTAGAATTTGTGTCAGTGTGTATTGTTTATAAAAATATTATAAAACTCG
GTTTGAGAGAGAAAATTACGAGCGTCACGGATGGAGGGCCCATGGAACTATCAGAAGAAGTTGTTGATGAG
TTCATGGAAGAAGTCCCGATGTCTGTAAGGCTTGCAAAATTTCGTTCGAAGACCGGAAAAAAGTTTAGTAGTA
AAAGTGAGAATAATAGTGGTAATAATAGGCCGAAACCAAACAAAAACCAAAGGAAGGAAAAGGGTTTAAAA
GTTAGGGTTGAGAAGGATAATTTAATTGATAATGAATTGGAGACTTACATCGCCGATTCAGATTCGTATTAAA
TATGTCTTACACAATCGCAACTCCATCGCAATTTGTGTTTTTGTCATCAGCATGGGCCGACCCTATAGAATTAAT
AAATTTATGTACTAATTCACTAGGTAATCAGTTCCAAACACAACAAGCTAGAACAACCGTTCAACGGCAATTTA
GCGAAGTGTGGAAACCTGTCCCTCAAGTCACTGTTAGGTTTCCTGACAGTGGTTTTAAGGTGTATAGGTACAA
TGCGGTACTAGATCCTCTAGTTACTGCTTTGTTAGGAGCTTTCGATACTAGAAATAGGATTATAGAAGTCGAAA
ATCAGGCGAACCCGACAACCGCCGAAACGTTAGACGCTACTCGTAGAGTAGATGACGCAACGGTGGCTATAA
GGAGCGCTATAAATAATTTAGTAGTAGAATTGGTCAAAGGAACAGGTTTGTACAATCAGAGCACATTTGAAA
GTGCATCCGGTTTACAATGGTCCTCTGCACCTGCATCTTGA

#OM515233

1

ATGGCATACACACAGACAGCTACCACATCCGCTTTGCTCGACACTGTCCGAGGTAACAATACCTTGGTCAATG
ATCTTGCGAAGCGGCGTCTTTATGACACAGCGGTCGACGAGTTCAACGCTCGTGATCGCAGGCCCAAAGTAA
ATTTTTCCAAAGTAATAAGTGAGGAACAGACGCTTATTGCTACTAGGGCATATCCAGAATTCCAGATAACCTTC
TATAATACGCAGAACGCCGTGCATTCGCTTGCCGGTGGACTACGATCCTTAGAACTGGAATATCTAATGATGC
AGATCCCGTACGGATCACTCACATATGATATAGGTGGGAATTTTGCATCTCATCTGTTCAAAGGACGGGCATA
TGTTCACTGCTGTATGCCCAATCTTGATGTCCGCGACATAATGCGGCACGAAGGCCAGAAAGACAGTATAGAA
TTATACCTTTCCAGGCTTGAGCGGGGCAACAAAGTTGTCCCAAATTTCCAAAAGGAAGCTTTTGACAGATACG
CTGAAACGCCAGATGAAGTTGTCTGTCACAGTACCTTCCAAACGTGTACGCACCAGCAGGTGGAAAACACAG
GCAGGGTGTATGCTATTGCATTGCACAGTATATACGATATACCTGCTGATGAATTCGGAGCGGCACTTTTAAG
GAAAAATGTCCATGTTTGTTACGCCGCCTTCCACTTTTCCGAGAATTTACTTCTCGAAGATTCACACGTCAACCT
TGACGAAATCAACGCGTGTTTTTCGCGTGATGGAGACAAGCTGACTTTTTCTTTCGCATCTGAGAGCACTTTAA
ATTATTGTCATAGTTATTCTAATATTTTAAAATACGTGTGCAAAACTTACTTCCCGGCATCTAATAGAGAGGTCT
ACATGAAGGAGTTTTTAGTCACCAGGGTTAACACCTGGTTTTGTAAGTTTTCTAGGATAGATACTTTTTTATTAT
ACAAGGGGGTAGCCCACAAAGGTGTAAATAGTGAGCAATTTTACAGCGCAATGGAAGATGCATGGCACTACA
AAAAGACTCTTGCAATGTGTAACAGCGAGAGGATTCTTCTTGAAGATTCCTCATCGGTCAATTACTGGTTCCCA
AAAATGAGAGATATGGTCATAGTTCCTCTATTCGACATATCTCTCGACACCAGTAAAAGGACCCGCAAAGAAG
TCTTAGTGTCAAAGGATTTTGTATTCACAGTTTTAAATCACATTCGCACTTATCAAGCCAAGGCACTTACATACT

CCAATGTTTTATCCTTTGTCGAATCAATTCGTTCAAGGGTAATTATCAACGGAGTGACTGCCAGGTCTGAGTGG
GATGTTGACAAATCTCTTTTGCAATCCTTGTCCATGACATTTTTCTTGCATACTAAGCTTGCCGTTTTAAAAGAC
GAATTGTTAATCAGCAAGTTTAGTTTGGGGCCAAAATCAGTAAGCCAGCATGTATGGGATGAGATTTCCCTGG
CTTTTGGAAACGCATTTCCATCGATCAAGGAGAGACTGCTAAATCGGAAACTAATTAAAGTGTCGGGAGACGC
ATTAGAAATCAGGGTGCCTGATTTATATGTGACTTTTCACGATAGATTAGTGACTGAGTACAAAACATCGGTG
GATATGCCAGTGCTTGATATCAGAAAGAGAATGGAGGAGACTGAGGTTATGTACAATGCATTGTCTGAGCTA
TCTGTGCTCAAGGAGTCGGACAAGTTCGACGTTGATGTTTTTTCCCGGATGTGCCAGACTTTGGAGGTAGACC
CAATGACTGCAGCAAAGGTTATAGTGGCAGTGATGAGCAACGAGAGCGGACTGACTCTTACATTCGAACAGC
CAACTGAAGCAAATGTCGCATTGGCACTTAAAGATTCAGAAAAAGCCTCTGAGGGTGCACTAGTGGTTACTTC
TAGAGATGTTGAAGAACCATCCATGAAGGGTTCAATGGCAAGAGGAGAGTTACAATTGGCCGGTCTGTCTGG
AGACCAACCAGAGTCTTCCTATACTCGGAACGAGGAAATAGAGTCATTAGAGCAATTCCACATGGCAATGGCT
AGTTCGTTAATTCGGAAACAGATGAGTTCGATTGTGTACACGGGCCCCATTAAAGTTCAGCAAATGAAAAACT
TTATTGATAGCCTGGTAGCATCACTCTCTGCTGCGGTGTCGAACCTAGTCAAGATCCTAAAGGATACAGCTGCT
ATAGATCTCGAAACCCGTCAGAAGTTTGGAGTCTTAGATGTTGCGACCAAAAGATGGTTAATTAAACCTTTAG
CCAAGAATCACGCATGGGGCGTTATTGAAACACATGCTAGGAAGTACCACGTTGCACTTTTGGAGTATGATGA
GCATGGAGTGGTAACTTGCGACAGTTGGAGAAGAGTGGCCGTGAGTTCTGAGTCAATGGTTTATTCTGATAT
GGCAAAGCTCAGAACACTGAGGAGATTATTAAGAGATGGTGAGCCTCATGTCAGCAGTGCTAAAGTCGTCCT
AGTTGACGGTGTCCCGGGTTGTGGAAAGACAAAAGAGATTCTCTCGAAAGTAAATTTTGAGGAAGATCTAAT
CTTAGTACCGGGTAAGCAGGCTGCTGAAATGATAAAGAGGCGTGCTAATGCGTCAGGAATAATTCAAGCCAC
AAGAGATAATGTTCGTACTGTTGATTCATTTATAATGAATTACGGTAAAGGAACACGCTGTCAGTTCAAAAGG
TTATTTATCGACGAAGGTCTGATGTTGCACACTGGTTGTGTGAATTTTCTTGTTTCTATGTCTCTGTGCGAAATT
GCATATGTTTATGGAGACACACAACAAATTCCATACATCAACAGAGTATCCGGTTTTCCGTACCCTGCACATTT
TGCAAAAATAGAGGTTGATGAGGTGGAAACTCGCAGAACTACGCTGCGTTGTCCAGCCGACATTACCCACTAT
CTTAACAGAAGGTACGAAGGACATGTCATGTGTACATCGTCGGTTAAAAAGTCAGTTTCTCAGGAAATGGTGA
GCGGGGCCGCAATGATCAATCCTGTATCTAAGCCATTGAATGGGAAAGTTTTGACTTTCACTCAGTCTGATAA
AGAGGCGCTGCTTTCTCGAGGATATACGGACGTCCATACAGTACATGAGGTACAAGGTGAGACATATGCAGA
TGTGTCGTTGGTCAGATTGACTCCGACACCTGTATCTATCATCGCAGGAGATAGTCCGCACGTTCTCGTAGCTT
TGTCAAGGCATACCCAAACATTGAAGTATTACACCGTAGTGATGGATCCTCTTGTAAGTATAATTAGGGATTTA
GAAAAACTTAGTTCTTACTTGTTAGATATGTATAAAGTAGATGCAGGGACCCAATAGCAATTACAGGTAGACT
CCGTGTTTAAAGGTTCTAATCTTTTTGTTGCAGCACCAAAGACTGGAGATATCTCAGATATGCAATTTTACTAT
GATAAGTGTCTCCCAGGTAATAGCACCATGTTAAATAACTATGATGCTGTTACCATGAGGTTGACTGACATTTC
TCTTAATGTCAAAGATTGCATATTGGATTTCTCTAAGTCTGTGGCTGCACCGAAGGATCCGATCAAACCACTGA
TTCCAATGGTACGAACGGCGGCAGAAATGCCACGCCAGACTGGACTATTGGAAAATTTGGTGGCGATGATCA
AAAGAAACTTTAATTCACCGGAGTTATCAGGAATAATCGACATTGAGAATACTGCATCTTTAGTAGTAGATAA
ATTTTTTGATAGTTACTTGCTTAAAGAAAAAAGAAAACCAAATAAAAATGTTTCTTTATTTTGTAGAGAGTCTCT
CAATAGATGGTTAGAGAAGCAGGAGCAAGTGACCATTGGTCAGCTTGCAGATTTTGATTTTGTGGATCTTCCT
GCCGTTGATCAGTACAGGCATATGATTAAAGCGCAACCTAAGCAGAAGCTGGATACATCAATTCAAAGCGAAT
ATCCGGCCTTGCAGACGATTGTGTATCATTCGAAAAAGATCAACGCAATCTTCGGTCCTTTGTTCAGTGAGCTC
ACAAGGCAAATGCTCGAAAGCATAGACTCAAGTAAGTTTTTGTTCTTTACAAGGAAGACGCCAGCTCAAATTG
AGGATTTCTTCGGAGATCTCGATAGCCATGTCCCTATGGATATCTTGGAGTTGGATATTTCGAAGTATGACAAA
TCTCAGAACGAGTTCCACTGTGCAGTAGAGTATGAAATATGGAGAAGACTTGGATTAGAAGATTTTCTGGGA
GAAGTTTGGAAACAAGGCCACAGAAAAACTACTCTTAAAGATTACACAGCTGGTATTAAAACGTGTTTATGGT
ACCAGAGAAAGAGTGGGGACGTTACAACATTCATCGGTAATACGGTGATTATTGCTGCTTGTTTAGCTTCCAT
GTTGCCCATGGAGAAAATAATCAAAGGTGCATTTTGCGGAGATGACAGTTTGCTATACTTCCCAAAAGGTTGT
GAGTTTCCTGACATACAGCATACAGCCAACCTTATGTGGAATTTCGAGGCTAAGCTATTCAGAAAGCAGTATG
GTTATTTCTGTGGAAGGTACGTGATACATCATGACAGAGGGTGTATTGTTTATTACGACCCTTTGAAGTTGATT
TCTAAACTTGGTGCTAAACACATCAAGGATTGGGATCACTTAGAAGAGTTCAGAAGATCCCTTTGTGATGTTG
CAATTTCGTTGAACAACTGTGCGTATTACACGCAGTTGGACGACGCTGTGAGTGAGGTCCATAAAACCGCACC

CCCGGGTTCGTTTGTTTATAAAAGTTTAGTTAAATATCTGTCCGATAAGGTTCTTTTTAGAAGTTTGTTTATAGA
TGGCTCTTGTTAAGGGTAAAGTCAATATTAATGAGTTCATAGACTTGTCAAAATCAGAAAAATTTCTTCCGTCT
ATGTTCACACCTGTTAAGAGTGTCATGATCTCCAAGGTTGATAAGATATTGGTTCATGAAGATGAATCTTTGTC
CGAAGTCAATTTACTCAAAGGTGTAAAACTCATTGATGGTGGCTATGTACATCTTGCTGGTCTTGTGGTGACA
GGTGAATGGAATTTGCCAGATAATTGTCGTGGTGGTGTCAGTGTCTGTTTGGTCGATAAGAGAATGGAGAGA
GCGGACGAGGCAACTCTTGCTTCATACTATACCGCAGCGGCTAAGAAAAGGTTTCAGTTCAAAGTCGTTCCAA
ATTACAACATCACTACCAAGGACGCAGAAAAGGCAGTTTGGCAAGTACTAGTTAATATTAGAAATGTTAAAAT
TGCTGCGGGTTACTGTCCGCTGTCATTAGAATTTGTGTCAGTGTGTATTGTTTATAAAAATATTATAAAACTCG
GTTTGAGAGAGAAAATTACGAGCGTCACGGATGGAGGGCCCATGGAACTATCAGAAGAAGTTGTTGATGAG
TTCATGGAAGAAGTCCCGATGTCTGTAAGGCTTGCAAAATTTCGTTCGAAGACCGGAAAAAAGTTTAGTAGTA
AAAGTGAGAATAATAGTGGTAATAATAGGCCGAAACCAGACAAAAACCAAAGGAAGGAAAAGGGTTTAAAA
GTTAGGGTTGAGAAGGATAATTTAATTGATAATGAATTGGAGACTTACGTCGCCGATTCAGATTCGTATTAAA
TATGTCTTACACAATCGCAACTCCATCGCAATTTGTGTTTTTGTCATCAGCATGGGCCGACCCTATAGAATTAAT
AAATTTATGTACTAATTCACTAGGTAATCAGTTCCAAACACAACAAGCTAGAACAACCGTTCAACGGCAATTTA
GCGAAGTGTGGAAACCTGTCCCTCAAGTCACTGTTAGGTTTCCTGACAGTGGTTTTAAGGTGTATAGGTACAA
CGCGGTACTAGATCCTCTAGTTACTGCTTTGTTAGGAGCTTTCGATACTAGAAATAGGATTATAGAAGTCGAA
AATCAGGCGAACCCGACAACCGCCGAAACGTTAGACGCTACTCGTAGAGTAGATGACGCAACGGTGGCTATA
AGGAGCGCTATAAATAATTTAGTAGTAGAATTGGTCAAAGGAACAGGTTTGTACAATCAAAGCACATTTGAAA
GTGCATCTGGTTTACAATGGTCCTCTGCACCTGCATCTTGA

#OM515235

1

ATGGCATACACACAGACAGCTACCACATCCGCTTTGCTCGACACTGTCCGAGGTAACAATACCTTGGTCAATG
ATCTTGCGAAGCGGCGTCTTTATGACACAGCGGTCGACGAGTTCAACGCTCGTGATCGCAGGCCCAAAGTAA
ATTTTTCCAAAGTAATAAGTGAGGAACAGACGCTTATTGCTACTAGGGCATATCCAGAATTCCAGATAACCTTC
TATAATACGCAGAACGCCGTGCATTCGCTTGCCGGTGGACTACGATCCTTAGAACTGGAATATCTAATGATGC
AGATCCCGTACGGATCACTCACATATGATATAGGTGGGAATTTTGCATCTCATCTGTTCAAAGGACGGGCATA
TGTTCACTGCTGTATGCCCAATCTTGATGTCCGCGACATAATGCGGCACGAAGGCCAGAAAGACAGTATAGAA
TTATACCTTTCCAGGCTTGAGCGGGGCAACAAAGTTGTCCCAAATTTCCAAAAGGAAGCTTTTGACAGATACG
CTGAAACGCCAGACGAAGTTGTCTGTCACAGTACCTTCCAAACGTGTACGCACCAGCAGGTGGAAAACTCAG
GCAGGGTGTATGCTATTGCATTGCACAGTATATACGATATACCTGCTGATGAATTCGGAGCGGCACTTTTAAG
GAAAAATGTCCATGTTTGTTACGCCGCCTTCCACTTTTCCGAGAATTTACTTCTCGAAGATTCACACGTCAACCT
TGACGAAATCAACGCGTGTTTTTCGCGTGATGGAGACAAGCTGACTTTTTCTTTCGCATCTGAGAGCACTTTAA
ATTATTGTCATAGTTATTCTAATATTTTAAAATACGTGTGCAAAACTTACTTCCCGGCATCTAATAGAGAGGTCT
ACATGAAGGAGTTTTTAGTCACCAGGGTTAACACCTGGTTTTGTAAGTTTTCTAGGATAGATACTTTTTTATTAT
ACAAGGGGGTAGCCCACAAAGGTGTAAATAGTGAGCAATTTTACAGCGCAATGGAAGATGCATGGCACTACA
AAAAGACTCTTGCAATGTGTAACAGCGAGAGGATTCTTCTTGAAGATTCCTCATCGGTCAATTACTGGTTCCCA
AAAATGAGAGATATGGTCATAGTTCCTCTATTCGACATATCTCTCGACACCAGTAAAAGGACCCGCAAAGAAG
TCTTAGTGTCAAAGGATTTTGTATTCACAGTTTTAAATCACATTCGCACTTATCAAGCCAAGGCACTTACATACT
CCAATGTTTTATCCTTTGTCGAATCAATTCGTTCAAGGGTAATTATCAACGGAGTGACTGCCAGGTCTGAGTGG
GATGTTGACAAATCTCTTTTGCAATCCTTGTCCATGACATTTTTCTTGCATACTAAGCTTGCCGTTTTAAAAGAC
GAATTGTTAATCAGCAAGTTTAGTTTGGGGCCAAAATCAGTAAGCCAGCATGTATGGGATGAGATTTCCCTGG
CTTTTGGAAACGCATTTCCATCGATCAAGGAGAGACTGCTAAATCGGAAACTAATTAAAGTGTCGGGAGACGC
ATTAGAAATCAGGGTGCCTGATTTATATGTGACTTTTCACGATAGATTAGTGACTGAGTACAAAACATCGGTG

GATATGTCAGTGCTTGATATCAGAAAGAGAATGGAGGAGACTGAGGTTATGTACAATGCATTGTCTGAGCTA
TCTGTGCTCAAGGAGTCGGACAAGTTCGACGTTGATGTTTTTTCCCGGATGTGCCAGACTTTGGAGGTAGACC
CAATGACTGCAGCAAAGGTTATAGTGGCAGTGATGAGCAACGAGAGCGGACTGACTCTTACATTCGAACAGC
CAACTGAAGCAAATGTCGCATTGGCACTTAAAGATTCAGAAAAAGCCTCTGAGGGTGCACTAGTGGTTACTTC
TAGAGATGTTGAAGAACCATCCATGAAGGGTTCAATGGCAAGAGGAGAGTTACAATTGGCCGGTCTGTCTGG
AGACCAACCAGAGTCTTCCTATACTCGGAACGAGGAAATAGAGTCATTAGAGCAATTCCACATGGCAACGGCT
AGTTCGTTAATTCGGAAACAGATGAGTTCGATTGTGTACACGGGCCCCATTAAAGTTCAGCAAATGAAAAACT
TTATTGATAGCCTGGTAGCATCACTCTCTGCTGCGGTGTCGAACCTAGTCAAGATCCTAAAGGATACAGCTGCT
ATAGATCTCGAAACCCGTCAGAAGTTTGGAGTCTTAGATGTTGCGACCAAAAGATGGTTAATTAAACCTTTAG
CCAAGAATCACGCATGGGGCGTTATTGAAACACATGCTAGGAAGTACCACGTTGCACTTTTGGAGTATGATGA
GCATGGAGTGGTAACTTGCGACAGTTGGAGAAGGGTGGCCGTGAGTTCTGAGTCAATGGTTTATTCTGATAT
GGCAAAGCTCAGAACACTGAGGAGATTATTAAGAGATGGTGAGCCTCATGTCAGCAGTGCTAAAGTCGTCCT
AGTTGACGGTGTCCCGGGTTGTGGAAAGACAAAAGAGATTCTCTCGAAAGTAAATTTTGAGGAAGATCTAAT
CTTAGTACCGGGTAAGCAGGCTGCTGAAATGATAAAGAGGCGTGCTAATGCGTCAGGAATAATTCAAGCCAC
AAGAGATAATGTTCGTACTGTTGATTCATTTATAATGAACTACGGTAAAGGAACACGCTGTCAGTTCAAAAGG
TTATTTATCGACGAAGGTCTGATGTTGCACACTGGTTGTGTGAATTTTCTTGTTTCTATGTCTCTGTGCGAAATT
GCATATGTTTATGGAGACACACAACAAATTCCATACATCAACAGAGTATCCGGTTTTCCGTACCCTGCACATTT
TGCAAAAATAGAGGTTGATGAGGTGGAAACTCGCAGAACTACGCTGCGTTGTCCAGCCGACATTACCCACTAT
CTTAACAGAAGGTACGAAGGACATGTCATGTGTACATCGTCGGTTAAAAAGTCAGTTTCTCAGGAAATGGTGA
GCGGGGCCGCAATGATCAATCCTGTATCTAAGCCATTGAATGGGAAAGTTTTGACTTTCACTCAGTCTGATAA
AGAGGTGCTGCTTTCTCGAGGATATACGGACGTCCATACAGTACATGAGGTACAAGGTGAGACATATGCAGA
TGTGTCGTTGGTCAGATTGACTCCGACACCTGTATCTATCATCGCAGGAGATAGTCCGCACGTTCTCGTAGCTT
TGTCAAGGCATACCCAAACATTGAAGTATTACACCGTAGTGATGGATCCTCTTGTAAGTATAATTAGGGATTTA
GAAAAACTTAGTTCTTACTTGTTAGATATGTATAAAGTAGATGCAGGGACCCAATAGCAATTACAGGTAGACT
CCGTGTTTAAAGGTTCTAATCTTTTTGTTGCAGCACCAAAGACTGGAGATATCTCAGATATGCAATTTTACTAT
GATAAGTGTCTCCCAGGTAATAGCACCATGTTAAATAACTATGATGCTGTTACCATGAGGTTGACTGACATTTC
TCTTAATGTCAAAGATTGCATATTGGATTTCTCTAAGTCTGTGGCTGCACCGAAGGATCCGATCAAACCACTGA
TTCCAATGGTACGAACGGCGGCAGAAATGCCACGCCAGACTGGACTATTGGAAAATTTGGTGGCGATGATCA
AAAGAAACTTTAATTCACCGGAGTTATCAGGAATAATCGACATTGAGAATACTGCATCTTTAGTAGTAGATAA
ATTTTTTGATAGTTACTTGCTTAAAGAAAAAAGAAAACCAAATAAAAATGTTTCTTTATTTTGTAGAGAGTCTCT
CAATAGATGGTTAGAGAAGCAGGAGCAAGTGACCATTGGTCAGCTTGCGGATTTTGATTTTGTGGATCTTCCT
GCCGTTGATCAGTACAGGCATATGATTAAAGCGCAACCTAAGCAGAAGCTGGATACATCAATTCAAAGCGAAT
ATCCGGCCTTGCAGACGATTGTGTATCATTCGAAAAAGATCAACGCAATCTTCGGTCCTTTGTTCAGTGAGCTC
ACAAGGCAAATGCTCGAAAGCATAGACTCAAGTAAGTTTTTGTTCTTTACAAGGAAGACGCCAGCTCAAATTG
AGGATTTCTTCGGAGATCTCGATAGCCATGTCCCTATGGATATCTTGGAGTTGGATATTTCGAAGTATGACAAA
TCTCAGAACGAGTTCCACTGTGCAGTAGAGTATGAAATATGGAGAAGACTTGGATTAGAAGATTTTCTGGGA
GAAGTTTGGAAACAAGGCCACAGAAAAACTACTCTTAAAGATTACACAGCTGGTATTAAAACGTGTTTATGGT
ACCAGAGAAAGAGTGGGGACGTTACAACATTCATCGGTAATACGGTGATTATTGCTGCTTGTTTAGCTTCCAT
GTTGCCCATGGAGAAAATAATCAAAGGTGCATTTTGCGGAGATGACAGTTTACTATACTTCCCAAAAGGTTGT
GAGTTTCCTGACATACAGCATACAGCCAACCTTATGTGGAATTTCGAGGCTAAGCTATTCAGAAAGCAGTATG
GTTATTTCTGTGGAAGGTACGTGATACATCATGACAGAGGGTGTATTGTTTATTATGACCCTTTGAAGTTGATT
TCTAAACTTGGTGCTAAACACATCAAGGATTGGGATCACTTAGAAGAGTTCAGAAGATCCCTTTGTGATGTTG
CAATTTCGTTGAACAACTGTGCGTATTACACGCAGTTGGACGACGCTGTGAGTGAGGTCCATAAAACCGCACC
CCCGGGTTCGTTTGTTTATAAAAGTTTAGTTAAATATCTGTCCGATAAGGTTCTTTTTAGAAGTTTGTTTATAGA
TGGCTCTTGTTAAGGGTAAAGTCAATATTAATGAGTTCATAGACTTGTCAAAATCAGAAAAATTTCTTCCGTCT
ATGTTCACACCTGTTAAGAGTGTCATGATCTCCAAGGTTGATAAGATATTGGTTCATGAAGATGAATCTTTGTC
CGAAGTCAATTTACTCAAAGGTGTAAAACTCATTGATGGTGGCTATGTACATCTTGCTGGTCTTGTGGTGACA
GGTGAATGGAATTTGCCAGATAATTGTCGTGGTGGTGTCAGTGTCTGTTTGGTCGATAAGAGAATGGAGAGA

GCGGACGAGGCAACTCTTGCTTCATACTATACCGCAGCGGCTAAGAAAAGGTTTCAGTTCAAAGTCGTTCCAA
ATTACAACATCACTACCAAGGACGCAGAAAAGGCAGTTTGGCAAGTACTAGTTAATATTAGAAATGTTAAAAT
TGCTGCGGGTTACTGTCCGCTGTCATTAGAATTTGTGTCAGTGTGTATTGTTTATAAAAATATTATAAAACTCG
GTTTGAGAGAGAAAATTACGAGCGTTACGGATGGAGGGCCCATGGAACTATCAGAAGAAGTTGTTGATGAGT
TCATGGAAGAAGTCCCGATGTCTGTAAGGCTTGCAAAATTTCGTTCGAAGACCGGAAAAAAGTTTAGTAGTAA
AAGTGAGAATAATAGTGGTAATAATAGGCCGAAGCCAGGCAAAAACCAAAGGAAGGAAAAGGGTTTAAAAG
TTAGGGTTGAGAAGGATAATTTAATTGATAATGAATTGGAGACTTACGTCGCCGATTCAGATTCGTATTAAAT
ATGTCTTACACAATCGCAACTCCATCGCAATTTGTGTTTTTGTCATCAGCATGGGCCGACCCTATAGAATTAATA
AATTTATGTACTAATTCACTAGGTAATCAGTTCCAAACACAACAAGCTAGAACAACCGTTCAACGGCAATTTAG
CGAAGTGTGGAAACCTGTCCCTCAAGTCACTGTTAGGTTTCCTGACAGTGGTTTTAAGGTGTATAGGTACAAT
GCGGTACTAGATCCTCTAGTTACTGCTTTGTTAGGAGCTTTCGATACTAGAAATAGGATTATAGAAGTCGAAA
ATCAGGCGAACCCGACAACCGCCGAAACGTTAGACGCTACTCGTAGAGTAGATGACGCAACGGTGGCTATAA
GGAGCGCTATAAATAATTTAGTAGTAGAATTGGTCAAAGGAACAGGTTTGTACAATCAGAGCACATTTGAAA
GTGCATCCGGTTTACAATGGTCCTCTGCACCTGCATCTTGA

#OM515248

1

ATGGCATACACACACAGACAGCTACCACATCCGCTTTGCTCGACACTGTCCGAGGTAACAATACCTTGGTCAACG
ATCTTGCGAAGCGGCGTCTTTATGACACAGCGGTCGACGAGTTCAACGCTCGTGATCGCAGGCCCAAAGTAA
ATTTTTCCAAAGTAATAAGTGAGGAACAGACGCTTATTGCTACTAGGGCATATCCAGAATTCCAGATAACCTTC
TATAATACGCAGAACGCCGTGCATTCGCTTGCCGGTGGACTACGATCCTTAGAACTGGAATATCTAATGATGC
AGATCCCGTACGGATCACTCACATATGATATAGGTGGGAATTTTGCATCTCATCTGTTCAAAGGACGGGCATA
TGTTCACTGCTGTATGCCCAATCTTGATGTCCGCGACATAATGCGGCACGAAGGCCAGAAAGACAGTATAGAA
TTATACCTTTCCAGGCTTGAGCGGGGCAACAAAGTTGTCCCAAATTTCCAAAAGGAAGCTTTTGACAGATACG
CTGAAACGCCAGACGAAGTTGTCTGTCACAGTACCTTTCAAACGTGTACGCACCAGCAGGTGGAAAACACAG
GCAGGGTGTATGCTATTGCATTGCACAGTATATACGATATACCTGCTGATGAATTCGGAGCGGCACTTTTAAG
GAAAAATGTCCATGTTTGTTACGCCGCCTTCCACTTTTCCGAGAATTTACTTCTCGAAGATTCACACGTCAACCT
TGACGAAATCAACGCGTGTTTTTCGCGTGATGGAGACAAGCTGACTTTTTCTTTCGCATCTGAGAGCACTTTAA
ATTATTGTCATAGTTATTCTAATATTTTAAAATACGTGTGCAGAACTTACTTCCCGGCATCTAATAGAGAGGTCT
ACATGAAGGAGTTTTTGGTCACCAGGGTTAACACCTGGTTTTGTAAGTTTTCTAGGATAGATACTTTTTTATTAT
ACAAGGGGGTAGCCCACAAAGGTGTAAATAGTGAGCAATTTTACAGCGCAATGGAAGATGCATGGCACTACA
AAAAGACTCTTGCAATGTGTAACAGCGAGAGGATTCTTCTTGAAGATTCCTCATCGGTCAATTACTGGTTCCCA
AAAATGAGAGATATGGTCATAGTTCCTCTATTCGACATATCTCTCGACACCAGTAAAAGGACCCGCAAAGAAG
TCTTAGTGTCAAAGGATTTTGTATTCACAGTTTTAAATCACATTCGCACTTATCAAGCCAAGGCACTTACATACT
CCAATGTTTTATCCTTTGTCGAATCAATTCGTTCAAGGGTAATTATCAACGGAGTGACTGCCAGGTCTGAGTGG
GATGTTGACAAATCTCTTTTGCAATCCTTGTCCATGACATTTTTCTTGCATACTAAGCTTGCCGTTTTAAAAGAC
GAATTGTTAATCAGCAAGTTTAGTTTGGGGCCAAAATCAGTAAGCCAGCATGTATGGGATGAGATTTCCCTGG
CTTTTGGAAACGCATTTCCATCGATCAAGGAGAGACTGCTAAATCGGAAACTAATTAAAGTGTCGGGAGACGC
ATTAGAAATCAGGGTGCCTGATTTATATGTGACTTTTCACGATAGATTAGTGACTGAGTACAAAACATCGGTG
GATATGCCAGTGCTTGATATCAGAAAGAGAATGGAGGAGACTGAGGTTATGTACAATGCACTGTCTGAGCTA
TCTGTGCTCAAGGAGTCGGACAAGTTCGACGTTGATGTTTTTTCCCGGATGTGCCAGACTTTGGAGGTAGACC
CAATGACTGCAGCAAAGGTTATTGTGGCAGTGATGAGCAACGAGAGCGGACTGACTCTTACATTCGAACAGC
CAACTGAAGCAAATGTCGCATTGGCACTTAAAGATTCAGAAAAAGCCTCTGAGGGTGCACTAGTGGTTACTTC
TAGAGATGTTGAAGAACCGTCCATGAAGGGTTCAATGGCAAGAGGAGAGTTACAATTGGCCGGTCTGTCTGG

AGACCAACCAGAGTCTTCCTATACTCGGAACGAGGAAATAGAGTCATTAGAGCAATTCCACATGGCAACGGCT
AGTTCGTTAATTCGGAAACAGATGAGTTCGATTGTGTACACGGGCCCCATTAAAGTTCAGCAAATGAAAAACT
TTATTGATAGCCTGGTAGCATCACTCTCTGCTGCGGTGTCGAACCTAGTCAAGATCCTAAAGGATACAGCTGCT
ATAGATCTCGAAACCCGTCAGAAGTTTGGAGTCTTAGATGTTGCGACCAAAAGATGGTTAATTAAACCTTTAG
CCAAGAATCACGCATGGGGCGTTATTGAAACACATGCTAGGAAGTACCACGTTGCACTTTTGGAGTATGATGA
GCATGGAGTGGTAACTTGCGACAGTTGGAGAAGGGTGGCCGTGAGTTCTGAGTCAATGGTTTATTCTGATAT
GGCAAAGCTCAGAACACTGAGGAGATTATTAAGGGATGGTGAGCCTCATGTCAGCAGTGCTAAAGTCGTCCT
AGTTGACGGTGTCCCGGGTTGTGGAAAGACAAAAGAGATTCTCTCAAAAGTAAATTTTGAGGAAGATCTAAT
CTTAGTACCGGGTAAGCAGGCTGCTGAAATGATAAAGAGGCGTGCTAATGCGTCAGGAATAATTCAAGCCAC
AAGAGATAATGTTCGTACTGTTGATTCATTTATAATGAATTACGGTAAAGGAACACGCTGTCAGTTCAAAAGG
TTATTTATCGACGAAGGTCTGATGTTGCACACTGGTTGTGTGAATTTTCTTGTTTCTATGTCTCTGTGCGAAATT
GCATATGTTTATGGAGACACACAACAAATTCCATACATCAACAGAGTATCCGGTTTTCCGTACCCTGCACATTT
TGCAAAAATAGAGGTTGATGAGGTGGAAACTCGCAGAACTACGCTGCGTTGTCCAGCCGACATTACCCACTAT
CTTAACAGAAGGTACGAAGGACATGTCATGTGTACATCGTCGGTTAAAAAGTCAGTTTCTCAGGAAATGGTGA
GCGGGGCCGCAATGATCAATCCTGTATCTAAGCCACTGAATGGGAAAGTTTTGACTTTCACTCAGTCTGATAA
AGAGGCGCTGCTTTCTCGAGGATATACGGACGTCCATACAGTACATGAGGTACAAGGTGAGACATATGCAGA
TGTGTCGTTGGTCAGATTGACTCCGACACCTGTATCTATCATCGCAGGAGATAGTCCGCACGTTCTCGTAGCTT
TGTCAAGGCATACCCAAACATTGAAGTATTACACCGTAGTGATGGATCCTCTTGTAAGTATAATTAGGGATTTA
GAAAAACTTAGTTCTTACTTGTTAGATATGTATAAAGTAGATGCAGGGACCCAATAGCAATTACAGGTAGACT
CCGTGTTTAAAGGTTCTAATCTTTTTGTTGCAGCACCAAAGACTGGAGATATCTCAGATATGCAATTTTACTAT
GATAAGTGTCTCCCAGGTAATAGCACCATGTTAAATAACTATGATGCTGTTACCATGAGGTTGACTGACATTTC
TCTTAATGTCAAAGATTGCATATTGGATTTCTCTAAGTCTGTGGCTGCACCGAAGGATCCGATCAAACCACTGA
TTCCAATGGTACGAACGGCGGCAGAAATGCCACGCCAGACTGGACTATTGGAAAATTTGGTGGCGATGATCA
AAAGAAACTTTAATTCACCGGAGTTATCAGGAATAATCGACATTGAGAATACTGCATCTTTAGTAGTAGATAA
ATTTTTTGATAGTTACTTGCTTAAAGAAAAAAGAAAACCAAATAAAAATGTTTCTTTATTTAGTAGAGAGTCTC
TCAATAGATGGTTAGAGAAGCAGGAGCAAGTGACCATTGGTCAGCTTGCAGATTTTGATTTTGTGGATCTTCC
TGCCGTTGATCAGTACAGGCATATGATTAAAGCGCAACCTAAGCAGAAGCTGGATACATCAATTCAAAGCGAA
TATCCGGCCTTGCAGACGATTGTGTATCATTCGAAAAAGATCAACGCAATCTTCGGTCCTTTGTTCAGTGAGCT
CACAAGGCAAATGCTCGAAAGCATAGACTCAAGTAAGTTTTTGTTCTTTACAAGGAAGACGCCAGCTCAAATT
GAGGATTTCTTCGGAGATCTCGATAGCCATGTCCCTATGGATATCTTGGAGTTGGATATTTCGAAGTATGACA
AATCTCAGAACGAGTTCCACTGTGCAGTAGAGTATGAAATATGGAGAAGACTTGGATTAGAAGATTTTCTGG
GAGAAGTTTGGAAACAAGGCCACAGGAAAACTACTCTTAAAGATTACACAGCTGGTATTAAAACGTGTTTATG
GTACCAGAGAAAGAGTGGGGACGTTACAACATTCATCGGTAATACGGTGATTATTGCTGCTTGTTTAGCTTCC
ATGTTGCCCATGGAGAAAATAATCAAAGGTGCATTTTGCGGAGATGACAGTTTACTATACTTCCCAAAAGGTT
GTGAGTTTCCTGACATACAGCATACAGCCAACCTTATGTGGAATTTCGAGGCTAAGCTATTCAGAAAGCAGTA
TGGTTATTTCTGTGGAAGGTACGTGATACATCATGACAGAGGGTGTATTGTTTATTATGACCCTTTGAAGTTGA
TTTCTAAACTTGGTGCTAAACACATCAAGGATTGGGATCACTTAGAAGAGTTCAGAAGATCCCTTTGTGATGTT
GCAAATTCGTTGAACAACTGTGCGTATTACACGCAGTTGGACGACGCTGTGAGTGAGGTCCATAAAACCGCAC
CCCCGGGTTCGTTTGTGTATAAAAGTTTAGTTAAATATCTGTCCGATAAGGTTCTTTTTAGAAGTTTGTTTATAG
ATGGCTCTTGTTAAGGGTAAAGTCAATATTAATGAGTTCATAGACTTGTCAAAATCAGAAAAATTTCTTCCGTC
TATGTTCACACCTGTTAAGAGTGTCATGATCTCCAAGGTTGATAAGATATTGGTTCATGAAGATGAATCTTTGT
CCGAAGTCAATTTACTCAAAGGTGTAAAACTCATTGATGGTGGCTATGTACATCTTGCTGGTCTTGTGGTGACA
GGTGAATGGAATTTGCCAGATAATTGTCGTGGTGGTGTCAGTGTCTGTTTGGTCGATAAGAGAATGGAGAGA
GCGGACGAGGCAACTCTTGCTTCATACTATACCGCAGCGGCTAAGAAAAGGTTTCAGTTCAAAGTCGTTCCAA
ATTACAACATCACTACCAAGGACGCAGAAAAGGCAGTTTGGCAAGTACTAGTTAATATTAGAAATGTTAAAAT
TGCTGCGGGTTACTGTCCGCTGTCATTAGAATTTGTGTCAGTGTGTATTGTTTATAAAAATATTATAAAACTCG
GTTTGAGAGAGAAAATTACGAGCGTCACGGATGGAGGGCCCATGGAACTATCAGAAGAAGTTGTTGATGAG
TTCATTGAAGAAGTCCCGATGTCTGTGAGGCTTGCAAAATTTCGTTCGAAGACCGGAAAAAAGTTTAGTAGTA

AAAGTGAGAATAATAGTGGTAATAATAGGTCGAAACCAAACAAAAACCAAAGGAAGGAAAAGGGTTTAAAA
GTTAGGGTTGAGAAGGATAATTTAATTGATAATGAATTGGAGACTTACATCGCCGATTCAGATTCGTATTAAA
TATGTCTTACACAATCGCAACTCCATCGCAATTTGTGTTTTTGTCATCAGCATGGGCCGACCCTATAGAATTAAT
AAATTTATGTACTAATTCACTAGGTAATCAGTTCCAAACACAACAAGCTAGAACAACCGTTCAACGGCAATTTA
GCGAAGTGTGGAAACCTGTCCCTCAAGTCACTGTTAGGTTTCCTGACAGTGGTTTTAAGGTGTATAGGTACAA
TGCGGTACTAGATCCTCTAGTTACTGCTTTGTTAGGAGCTTTCGATACTAGAAATAGGATTATAGAAGTCGAAA
ATCAGGCGAACCCGACAACCGCCGAAACGTTAGACGCTACTCGTAGAGTAGATGACGCAACGGTGGCTATAA
GGAGCGCTATAAATAATTTAGTAGTAGAATTGGTCAAAGGAACAGGTTTGTACAATCAGAGCACATTTGAAA
GTGCATCCGGTTTACAATGGTCCTCTGCACCTGCATCTTGA

#OM515258

1

ATGGCATACACACAGACAGCTACCACATCCGCTTTGCTCGACACTGTCCGAGGTAACAATACCTTGGTCAATG
ATCTTGCGAAGCGGCGTCTTTATGACACAGCGGTCGACGAGTTCAACGCTCGTGATCGCAGGCCCAAAGTAA
ATTTTTCCAAAGTAATAAGTGAGGAACAGACGCTTATTGCTACTAGGGCATATCCAGAATTCCAGATAACCTTC
TATAATACGCAGAACGCCGTGCATTCGCTTGCCGGTGGACTACGATCCTTAGAACTGGAATATCTAATGATGC
AGATCCCGTACGGATCACTCACATATGATATAGGTGGGAATTTTGCATCTCATCTGTTCAAAGGACGGGCATA
TGTTCACTGCTGTATGCCCAATCTTGATGTCCGCGACATAATGCGGCACGAAGGCCAGAAAGACAGTATAGAA
TTATACCTTTCCAGGCTTGAGCGGGGCAACAAAGTTGTCCCAAATTTCCAAAAGGAAGCTTTTGACAGATACG
CTGAAACGCCAGACGAAGTTGTCTGTCACAGTACCTTCCAAACGTGTACGCACCAGCAGGTGGAAAACACAG
GCAGGGTGTATGCTATTGCATTGCACAGTATATACGATATACCTGCTGATGAATTCGGAGCGGCACTTTTAAG
GAAAAATGTCCATGTTTGTTACGCCGCCTTCCACTTTTCCGAGAATTTACTTCTCGAAGATTCACACGTCAACCT
TGACGAAATCAACGCGTGTTTTTCGCGTGATGGAGACAAGCTGACTTTTTCTTTCGCATCTGAGAGCACTTTAA
ATTATTGTCATAGTTATTCTAATATTTTAAAATACGTGTGCAAAACTTACTTCCCGGCATCTAATAGAGAGGTCT
ACATGAAGGAGTTTTTAGTCACCAGGGTTAACACCTGGTTTTGTAAGTTTTCTAGGATAGATACTTTTTTATTAT
ACAAGGGGGTAGCCCACAAAGGTGTAAATAGTGAGCAATTTTACAGCGCAATGGAAGATGCATGGCACTACA
AAAAGACTCTTGCAATGTGTAACAGCGAGAGGATTCTTCTTGAAGATTCCTCATCGGTCAATTACTGGTTCCCA
AAAATGAGAGATATGGTCATAGTTCCTCTATTCGACATATCTCTCGACACCAGTAAAAGGACCCGCAAAGAAG
TCTTAGTGTCAAAGGATTTTGTATTCACAGTTTTAAATCACATTCGCACTTATCAAGCCAAGGCACTTACATACT
CCAATGTTTTATCCTTTGTCGAATCAATTCGTTCAAGGGTAATTATCAACGGAGTGACTGCCAGGTCTGAGTGG
GATGTTGACAAATCTCTTTTGCAATCCTTGTCCATGACATTTTTCTTGCATACTAAGCTTGCCGTTTTAAAAGAC
GAATTGTTAATCAGCAAGTTTAGTTTGGGGCCAAAATCAGTAAGCCAGCATGTATGGGATGAGATTTCCCTGG
CTTTTGGAAACGCATTTCCATCGATCAAGGAGAGACTGCTAAATCGGAAACTAATTAAAGTGTCGGGAGACGC
ATTAGAAATCAGGGTGCCTGATTTATATGTGACTTTTCACGATAGATTAGTGACTGAGTACAAAACATCGGTG
GATATGCCAGTGCTTGATATCAGAAAGAGAATGGAGGAGACTGAGGTTATGTACAATGCATTGTCTGAGCTA
TCTGTGCTCAAGGAGTCGGACAAGTTCGACGTTGATGTTTTTTCCCGGATGTGCCAGACTTTGGAGGTAGACC
CAATGACTGCAGCAAAGGTTATAGTGGCAGTGATGAGCAACGAGAGCGGACTGACTCTTACATTCGAACAGC
CAACTGAAGCAAATGTCGCATTGGCACTTAAAGATTCAGAAAAAGCCTCTGAGGGTGCACTAGTGGTTACTTC
TAGAGATGTTGAAGAACCATCCATGAAGGGTTCAATGGCAAGAGGAGAGTTACAATTGGCCGGTCTGTCTGG
AGACCAACCAGAGTCTTCCTATACTCGGAACGAGGAAATAGAGTCATTAGAGCAATTCCACATGGCAACGGCT
AGTTCGTTAATTCGGAAACAGATGAGTTCGATTGTGTACACGGGCCCCATTAAAGTTCAGCAAATGAAAAACT
TTATTGATAGCCTGGTAGCATCACTCTCTGCTGCGGTGTCGAACCTAGTCAAGATCCTAAAGGATACAGCTGCT
ATAGATCTCGAAACCCGTCAGAAGTTTGGAGTCTTAGATGTTGCGACCAAAAGATGGTTAATTAAACCTTTAG
CCAAGAATCACGCATGGGGCGTTATTGAAACACATGCTAGGAAGTACCACGTTGCACTTTTGGAGTATGATGA

GCATGGAGTGGTAACTTGCGACAGTTGGAGAAGGGTGGCCGTGAGTTCTGAGTCAATGGTTTATTCTGATAT
GGCAAAGCTCAGAACACTGAGGAGATTATTAAGAGATGGTGAGCCTCATGTCAGCAGTGCTAAAGTCGTCCT
AGTTGACGGTGTCCCGGGTTGTGGAAAGACAAAAGAGATTCTCTCGAAAGTAAATTTTGAGGAAGATCTAAT
CTTAGTACCGGGTAAGCAGGCTGCTGAAATGATAAAGAGGCGTGCTAATGCGTCAGGAATAATTCAAGCCAC
AAGAGATAATGTTCGTACTGTTGATTCATTTATAATGAACTACGGTAAAGGAACACGCTGTCAGTTCAAAAGG
TTATTTATCGACGAAGGTCTGATGTTGCACACTGGTTGTGTGAATTTTCTTGTTTCTATGTCTCTGTGCGAAATT
GCATATGTTTATGGAGACACACAACAAATTCCATACATCAACAGAGTATCCGGTTTTCCGTACCCTGCACATTT
TGCAAAAATAGAGGTTGATGAGGTGGAAACTCGCAGAACTACGCTGCGTTGTCCAGCCGACATTACCCACTAT
CTTAACAGAAGGTACGAAGGACATGTCATGTGTACATCGTCGGTTAAAAAGTCAGTTTCTCAGGAAATGGTGA
GCGGGGCCGCAATGATCAATCCTGTATCTAAGCCATTGAATGGGAAAGTTTTGACTTTCACTCAGTCTGATAA
AGAGGCGCTGCTTTCTCGAGGATATACGGACGTCCATACAGTACATGAGGTACAAGGTGAGACATATGCAGA
TGTGTCGTTGGTCAGATTGACTCCGACACCTGTATCTATCATCGCAGGAGATAGTCCGCACGTTCTCGTAGCTT
TGTCAAGGCATACCCAAACATTGAAGTATTACACCGTAGTGATGGATCCTCTTGTAAGTATAATTAGGGATTTA
GAAAAACTTAGTTCTTACTTGTTAGATATGTATAAAGTAGATGCAGGGACCCAATAGCAATTACAGGTAGACT
CCGTGTTTAAAGGTTCTAATCTTTTTGTTGCAGCACCAAAGACTGGAGATATCTCAGATATGCAATTTTACTAT
GATAAGTGTCTCCCAGGTAATAGCACCATGTTAAATAACTATGATGCTGTTACCATGAGGTTGACTGACATTTC
TCTTAATGTCAAAGATTGCATATTGGATTTCTCTAAGTCTGTGGCTGCACCGAAGGATCCGATCAAACCACTGA
TTCCAATGGTACGAACGGCGGCAGAAATGCCACGCCAGACTGGACTATTGGAAAATTTGGTGGCGATGATCA
AAAGAAACTTTAATTCACCGGAGTTATCAGGAATAATCGACATTGAGAATACTGCATCTTTAGTAGTAGATAA
ATTTTTTGATAGTTACTTGCTTAAAGAAAAAAGAAAACCAAATAAAAATGTTTCTTTATTTTGTAGAGAGTCTCT
CAATAGATGGTTAGAGAAGCAGGAGCAAGTGACCATTGGTCAGCTTGCAGATTTTGATTTTGTGGATCTTCCT
GCCGTTGATCAGTACAGGCATATGATTAAAGCGCAACCTAAGCAGAAGCTGGATACATCAATTCAAAGCGAAT
ATCCGGCCTTGCAGACGATTGTGTATCATTCGAAAAAGATCAACGCAATCTTCGGTCCTTTGTTCAGTGAGCTC
ACAAGGCAAATGCTCGAAAGCATAGACTCAAGTAAGTTTTTGTTCTTTACAAGGAAGACGCCAGCTCAAATTG
AGGATTTCTTCGGAGATCTCGATAGCCATGTCCCTATGGATATCTTGGAGTTGGATATTTCGAAGTATGACAAA
TCTCAGAACGAGTTCCACTGTGCAGTAGAGTATGAAATATGGAGAAGACTTGGATTAGAAGATTTTCTGGGA
GAAGTTTGGAAACAAGGCCACAGAAAAACTACTCTTAAAGATTACACAGCTGGTATTAAAACGTGTTTATGGT
ACCAGAGAAAGAGTGGGGACGTTACAACATTCATCGGTAATACGGTGATTATTGCTGCTTGTTTAGCTTCCAT
GTTGCCCATGGAGAAAATAATCAAAGGTGCATTTTGCGGAGATGACAGTTTACTATACTTCCCAAAAGGTTGT
GAGTTTCCTGACATACAGCATACAGCCAACCTTATGTGGAATTTCGAGGCTAAGCTATTCAGAAAGCAGTATG
GTTATTTCTGTGGAAGGTACGTGATACATCATGACAGAGGGTGTATTGTTTATTACGACCCTTTGAAGTTGATT
TCTAAACTTGGTGCTAAACACATCAAGGATTGGGATCACTTAGAAGAGTTCAGAAGATCCCTTTGTGATGTTG
CAATTTCGTTGAACAACTGTGCGTATTACACGCAGTTGGACGACGCTGTGAGTGAGGTCCATAAAACCGCACC
CCCGGGTTCGTTTGTTTATAAAAGTTTAGTTAAATATCTGTCCGATAAGGTTCTTTTTAGAAGTTTGTTTATAGA
TGGCTCTTGTTAAGGGTAAAGTCAATATTAATGAGTTCATAGACTTGTCAAAATCAGAAAAATTTCTTCCGTCT
ATGTTCACACCTGTTAAGAGTGTCATGATCTCCAAGGTTGATAAGATATTGGTTCATGAAGATGAATCTTTGTC
CGAAGTCAATTTACTCAAAGGTGTAAAACTCATTGATGGTGGCTATGTACATCTTGCTGGTCTTGTGGTGACA
GGTGAATGGAATTTGCCAGATAATTGTCGTGGTGGTGTCAGTGTCTGTTTGGTCGATAAGAGAATGGAGAGA
GCGGACGAGGCAACTCTTGCTTCATACTATACCGCAGCGGCTAAGAAAAGGTTTCAGTTCAAAGTCGTTCCAA
ATTACAACATCACTACCAAGGACGCAGAAAAGGCAGTTTGGCAAGTACTAGTTAATATTAGAAATGTTAAAAT
TGCTGCGGGTTACTGTCCGCTGTCATTAGAATTTGTGTCAGTGTGTATTGTTTATAAAAATATTATAAAACTCG
GTTTGAGAGAGAAAATTACGAGCGTCACGGATGGAGGGCCCATGGAACTATCAGAAGAAGTTGTTGATGAG
TTCATGGAAGAAGTCCCGATGTCTGTAAGGCTTGCAAAATTTCGTTCGAAGACCGGAAAAAAGTTTAGTAGTA
AAAGTGAGAATAATAGTGGTAATAATAGGCCGAAGCCAGGCAAAAACCAAAGGAAGGAAAAGGGTTTAAAA
GTTAGGGTTGAGAAGGATAATTTAATTGATAATGAATTGGAGACTTACGTCGCCGATTCAGATTCGTATTAAA
TATGTCTTACACAATCGCAACTCCATCGCAATTTGTGTTTTTGTCATCAGCATGGGCCGACCCTATAGAATTAAT
AAATTTATGTACTAATTCACTAGGTAATCAGTTCCAAACACAACAAGCTAGAACAACCGTTCAACGGCAATTTA
GCGAAGTGTGGAAACCTGTCCCTCAAGTCACTGTTAGGTTTCCTGACAGTGGTTTTAAGGTGTATAGGTACAA

TGCGGTACTAGATCCTCTAGTTACTGCTTTGTTAGGAGCTTTCGATACTAGAAATAGGATTATAGAAGTCGAAA
ATCAGGCGAACCCGACAACCGCCGAAACGTTAGACGCTACTCGTAGAGTAGATGACGCAACGGTGGCTATAA
GGAGCGCTATAAATAATTTAGTAGTAGAATTGGTCAAAGGAACAGGTTTGTACAATCAGAGCACATTTGAAA
GTGCATCCGGTTTACAATGGTCCTCTGCACCTGCATCTTGA

#OM515261

1

ATGGCATACACACAGACAGCTACCACATCCGCTTTGCTCGACACTGTCCGAGGTAACAATACCTTGGTCAACG
ATCTTGCGAAGCGGCGTCTTTATGATACAGCGGTCGACGAGTTCAACGCTCGTGATCGCAGGCCCAAAGTAAA
TTTTTCCAAAGTAATAAGTGAGGAACAGACGCTTATTGCTACTAGGGCATATCCTGAATTCCAGATAACCTTCT
ATAATACGCAGAACGCCGTGCATTCGCTTGCCGGTGGACTACGATCCTTAGAACTGGAATATCTAATGATGCA
GATCCCGTACGGATCACTCACATATGATATAGGTGGGAATTTTGCATCTCATCTGTTCAAAGGACGGGCATAT
GTTCACTGCTGTATGCCCAATCTTGATGTCCGCGACATAATGCGGCACGAAGGCCAGAAAGACAGTATAGAAT
TATACCTTTCCAGGCTTGAGCGGGGCAACAAAGTTGTCCCAAATTTCCAAAAGGAAGCTTTTGACAGATACGC
TGAAACGCCAGACGAAGTTGTCTGTCACAGTACCTTCCAAACGTGTACACACCAGCAGGTGGAAAACACAGG
CAGGGTGTATGCTATTGCATTGCACAGTATATACGATATACCTGCTGATGAATTCGGAGCGGCACTTTTAAGG
AAAAATGTCCATGTTTGTTACGCCGCCTTCCACTTTTCCGAGAATTTACTTCTCGAAGATTCACACGTCAACCTT
GACGAAATCAACGCGTGTTTTTCGCGTGATGGAGACAAGCTGACTTTTTCTTTCGCATCTGAGAGCACTTTAAA
TTATTGTCATAGTTATTCTAATATTTTAAAATACGTGTGCAAAACTTACTTCCCGGCATCTAATAGAGAGGTCTA
CATGAAGGAGTTTTTGGTCACCAGGGTTAACACCTGGTTTTGTAAGTTTTCTAGGATAGATACTTTTTTATTATA
CAAGGGGGTAGCCCACAAAGGTGTAAATAGTGAGCAATTTTACAGCGCAATGGAAGATGCATGGCACTACAA
AAAGACTCTTGCAATGTGTAACAGCGAGAGGATTCTTCTTGAAGATTCCTCATCGGTCAATTACTGGTTCCCAA
AAATGAGAGATATGGTCATAGTTCCTCTATTCGACATATCTCTCGACACTAGTAAAAGGACCCGCAAAGAAGT
CTTAGTGTCAAAGGATTTTGTATTCACAGTTTTAAATCACATTCGCACTTATCAAGCCAAGGCACTTACATACTC
CAATGTTTTATCCTTTGTCGAATCAATTCGTTCAAGGGTAATTATCAACGGAGTGACTGCCAGGTCTGAGTGG
GATGTTGACAAATCTCTTTTGCAATCCTTGTCCATGACATTTTTCTTGCATACTAAGCTTGCCGTTTTAAAAGAC
GAATTGTTAATCAGCAAGTTTAGTTTGGGGCCAAAATCAGTAAGCCAGCATGTATGGGATGAGATTTCCCTGG
CTTTTGGAAACGCATTTCCATCGATCAAGGAGAGACTGCTAAATCGGAAACTAATTAAAGTGTCGGGAGACGC
ATTAGAAATCAGGGTGCCTGATTTATATGTGACTTTTCACGATAGATTAGTGACTGAGTACAAAACATCGGTG
GATATGCCAGTGCTTGATATCAGAAAGAGAATGGAGGAGACTGAGGTTATGTACAATGCATTGTCTGAGCTA
TCTGTGCTCAAGGAGTCGGACAAGTTCGACGTTGATGTTTTTTCCCGGATGTGCCAGACTTTGGAGGTAGACC
CAATGACTGCAGCAAAGGTTATTGTGGCAGTGATGAGCAACGAGAGCGGACTGACTCTTACATTCGAACAGC
CAACTGAAGCAAATGTCGCATTGGCACTTAAAGATTCAGAAAAAGCCTCTGAGGGTGCACTAGTGGTTACTTC
TAGAGATGTTGAAGAACCATCCATGAAGGGTTCAATGGCAAGAGGAGAGTTACAATTGGCCGGTCTGTCTGG
AGACCAACCAGAGTCTTCCTATACTCGGAACGAGGAAATAGAGTCATTAGAGCAATTCCACATGGCAACGGCT
GGTTCGTTAATTCGGAAACAGATGAGTTCGATTGTGTACACGGGCCCCATTAAAGTTCAGCAAATGAAAAACT
TTATTGATAGCCTGGTAGCATCACTCTCTGCTGCGGTGTCGAACCTAGTCAAGATCCTAAAGGATACAGCTGCT
ATAGATCTCGAAACCCGTCAGAAGTTTGGAGTCTTAGATGTTGCGACCAAAAGATGGTTAATTAAACCTTTAG
CCAAGAATCACGCATGGGGCGTTATTGAAACACATGCTAGGAAGTACCACGTTGCACTTTTGGAGTATGATGA
GCATGGAGTGGTAACTAGCGACAGTTGGAGAAGGGTGGCCGTGAGTTCTGAGTCAATGGTTTATTCTGATAT
GGCAAAGCTCAGAACACTGAGGAGATTATTAAGAGATGGTGAGCCTCATGTCAGCAGTGCTAAAGTCGTCCT
AGTTGACGGTGTCCCGGGTTGTGGAAAGACAAAAGAGATTCTCTCGAAAGTAAATTTTGAGGAAGATCTAAT
CTTAGTACCGGGTAAGCAGGCTGCTGAAATGATAAAGAGGCGTGCTAATGCGTCAGGAATAATTCAAGCCAC
AAGAGATAATGTTCGTACTGTTGATTCATTTATAATGAATTACGGTAAAGGAACACGCTGTCAGTTCAAAAGG

TTATTTATCGACGAAGGTCTGATGTTGCACACTGGTTGTGTGAATTTTCTTGTTTCAATGTCTCTGTGCGAAATT
GCATATGTTTATGGAGACACACAACAAATTCCATACATCAACAGAGTATCCGGTTTTCCGTACCCTGCACATTT
TGCAAAAATAGAGGTTGATGAGGTGGAAACTCGCAGAACTACGCTGCGTTGTCCAGCCGACATTACCCACTAT
CTTAACAGAAGGTACGAAGGACATGTCATGTGTACATCGTCGGTTAAAAAGTCAGTTTCTCAGGAAATGGTGA
GCGGGGCCGCAATGATCAATCCTGTATCTAAGCCATTGAATGGGAAAGTTTTGACTTTCACTCAGTCTGATAA
AGAGGCGCTGCTTTCTCGAGGATATACGGACGTCCATACAGTACATGAGGTACAAGGTGAGACATATGCAGA
TGTGTCGTTGGTCAGATTGACTCCGACACCTGTATCTATCATCGCAGGAGATAGTCCGCACGTTCTCGTAGCTT
TGTCAAGGCATACCCAAACATTGAAGTATTACACCGTAGTGATGGATCCTCTTGTAAGTATAATTAGGGATTTA
GAAAAACTTAGTTCTTACTTGTTAGATATGTATAAAGTAGATGCAGGGACCCAATAGCAATTACAGGTAGACT
CCGTGTTTAAAGGTTCTAATCTTTTTGTTGCAGCACCAAAGACTGGAGATATCTCAGATATGCAATTTTACTAT
GATAAGTGTCTCCCAGGTAATAGCACCATGTTAAATAACTATGATGCTGTTACCATGAGGTTGACTGACATTTC
TCTTAATGTCAAAGATTGCATATTGGATTTCTCTAAGTCTGTGGCTGCACCGAAGGATCCGATCAAACCACTGA
TTCCAATGGTACGAACGGCGGCAGAAATGCCACGCCAGACTGGACTATTGGAAAATTTGGTGGCGATGATCA
AAAGAAACTTTAATTCACCGGAGTTATCAGGAATAATCGACATTGAGAATACTGCATCTTTAGTAGTAGATAA
ATTTTTTGATAGTTACTTGCTTAAAGAAAAAAGAAAACCAAATAAAAATGTTTCTTTATTTTGTAGAGAGTCTCT
CAATAGATGGTTAGAGAAGCAGGAGCAAGTGACCATTGGTCAGCTTGCAGATTTTGATTTTGTGGATCTTCCT
GCCGTTGATCAGTACAGGCATATGATTAAAGCGCAACCTAAGCAGAAGCTGGATACATCAATTCAAAGCGAAT
ATCCGGCCTTGCAGACGATTGTGTATCATTCGAAAAAGATCAACGCAATCTTCGGTCCTTTGTTCAGTGAGCTC
ACAAGGCAAATGCTCGAAAGCATAGACTCAAGTAAGTTTTTGTTCTTTACAAGGAAGACGCCAGCTCAAATTG
AGGATTTCTTCGGAGATCTCGATAGCCATGTCCCTATGGATATCTTGGAGTTGGATATTTCGAAGTATGACAAA
TCTCAGAACGAGTTCCACTGTGCAGTAGAGTATGAAATATGGAGAAGACTTGGATTAGAAGATTTTCTGGGA
GAAGTTTGGAAACAAGGCCACAGAAAAACTACTCTTAAAGATTACACAGCTGGTATTAAAACGTGTTTATGGT
ACCAGAGAAAGAGTGGGGACGTTACAACATTCATCGGTAATACGGTGATTATTGCTGCTTGTTTAGCTTCCAT
GTTACCCATGGAGAAAATAATCAAAGGTGCATTTTGCGGAGATGACAGTTTACTATACTTCCCAAAAGGTTGT
GAGTTTCCTGACATACAGCATACAGCCAACCTTATGTGGAATTTCGAGGCTAAGCTATTCAGAAAGCAGTATG
GTTATTTCTGTGGAAGGTACGTGATACATCATGACAGAGGGTGTATTGTTTATTATGACCCTTTGAAGTTGATT
TCTAAACTTGGTGCTAAACACATCAAGGATTGGGATCACTTAGAAGAGTTCAGAAGATCCCTTTGTGATGTTG
CAAATTCGTTGAACAACTGTGCGTATTACACGCAGTTGGACGACGCTGTGAGTGAGGTCCATAAAACCGCACC
CCCGGGTTCGTTTGTATATAAAAGTTTAGTTAAATATCTGTCCGATAAGGTTCTTTTTAGAAGTTTGTTTATAGA
TGGCTCTTGTTAAGGGTAAAGTCAATATTAATGAGTTCATAGACTTGTCAAAATCAGAAAAATTTCTCCCGTCT
ATGTTCACACCTGTTAAGAGTGTCATGATCTCCAAGGTTGATAAGATATTGGTTCATGAAGATGAATCTTTGTC
CGAAGTCAATTTACTCAAAGGTGTAAAACTCATTGATGGTGGCTATGTACATCTTGCTGGTCTTGTGGTGACA
GGTGAATGGAATTTGCCAGATAATTGTCGTGGTGGTGTCAGTGTCTGTTTGGTCGATAAAAGAATGGAGAGA
GCGGACGAGGCAACTCTTGCTTCATACTATACCGCAGCGGCTAAGAAAAGGTTTCAGTTCAAAGTCGTTCCAA
ATTACAACATCACTACCAAGGACGCAGAAAAGGCAGTTTGGCAAGTACTAGTTAATATTAGAAATGTTAAAAT
TGCTGCGGGTTACTGTCCGCTGTCATTAGAATTTGTGTCAGTGTGTATTGTTTATAAAAATATTATAAAACTCG
GTTTGAGAGAGAAAATTACGAGCGTCACGGATGGAGGGCCCATGGAACTATCAGAAGAAGTTGTTGATGAG
TTCATGGAAGAAGTCCCGATGTCTGTAAGGCTTGCAAAATTTCGTTCGAAGACCGGGAAAAAGTTTAGTAGTA
AAAGTGAGAATAATAGTGGTAATAATAGGCCGAAACCAAACAAAAACCAAAGGAAGGAAAAGGGTTTAAAA
GTTAGGGTTGAGAAGGATAATTTAATTGATAATGAATTGGAGACTTACGTCGCCGATTCAGATTCGTATTAAA
TATGTCTTACACAATCGCAACTCCATCGCAATTTGTGTTTTTGTCATCAGCATGGGCCGACCCTATAGAATTAAT
AAATTTATGTACTAATTCACTAGGTAATCAATTCCAAACACAACAAGCTAGAACAACCGTTCAACGGCAATTTA
GCGAAGTGTGGAAACCTGTCCCTCAAGTCACTGTTAGGTTTCCTGACAGTGGTTTTAAGGTGTATAGGTACAA
TGCGGTACTAGATCCTCTAGTTACTGCTTTGTTAGGAGCTTTCGATACTAGAAATAGGATTATAGAAGTCGAAA
ATCAGGCGAACCCGACAACCGCCGAAACGTTAGACGCTACTCGTAGAGTAGATGACGCAACGGTGGCTATAA
GGAGCGCTATAAATAATTTAGTAGTAGAATTGGTCAAAGGAACAGGTTTGTACAATCAGAGCACATTTGAAA
GTGCATCCGGTTTACAATGGTCCTCTGCACCTGCATCTTGA

#OM515264

1

ATGGCATACACACAGACAGCTACCACATCCGCTTTGCTCGACACTGTCCGAGGTAACAATACCTTGGTCAATG
ATCTTGCGAAGCGGCGTCTTTATGACACAGCGGTCGACGAGTTCAACGCTCGTGATCGCAGGCCCAAAGTAA
ATTTTTCCAAAGTAATAAGTGAGGAACAGACGCTTATTGCTACTAGGGCATATCCAGAATTCCAGATAACCTTC
TATAATACGCAGAACGCCGTGCATTCGCTTGCCGGTGGACTACGATCCTTAGAACTGGAATATCTAATGATGC
AGATCCCGTACGGATCACTCACATATGATATAGGTGGGAATTTTGCATCACATCTGTTCAAAGGACGGGCATA
TGTTCACTGCTGTATGCCCAATCTTGATGTCCGCGACATAATGCGGCACGAAGGCCAGAAAGACAGTATAGAA
TTATACCTTTCCAGGCTTGAGCGGGGCAACAAAGTTGTCCCAAATTTCCAAAAGGAAGCTTTTGACAGATACG
CTGAAACGCCAGACGAAGTTGTCTGTCACAGTACCTTCCAAACGTGTACGCACCAGCAGGTGGAAAACACAG
GCAGGGTGTATGCTATTGCATTGCACAGTATATACGATATACCTGCTGATGAATTCGGAGCGGCACTTTTAAG
GAAAAATGTCCATGTTTGTTACGCCGCCTTCCACTTTTCCGAGAATTTACTTCTCGAAGATTCACACGTCAACCT
TGACGAAATCAACGCGTGTTTTTCGCGTGATGGAGACAAGCTGACTTTTTCTTTCGCATCTGAGAGCACTTTAA
ATTATTGTCATAGTTATTCTAATATTTTAAAATACGTGTGCAAAACTTACTTCCCGGCATCTAATAGAGAGGTCT
ACATGAAGGAGTTTTTAGTCACCAGGGTTAACACCTGGTTTTGTAAGTTTTCTAGGATTGATACTTTTTTATTAT
ACAAGGGGGTAGCCCACAAAGGTGTAAATAGTGAGCAATTTTACAGCGCAATGGAAGATGCATGGCACTACA
AAAAGACTCTTGCAATGTGTAACAGCGAGAGGATTCTTCTTGAAGATTCCTCATCGGTCAATTACTGGTTCCCA
AAAATGAGAGATATGGTCATAGTTCCTCTATTCGACATATCTCTCGACACCAGTAAAAGGACCCGCAAAGAAG
TCTTAGTGTCAAAGGATTTTGTATTCACAGTTTTAAATCACATTCGCACTTATCAAGCCAAGGCACTTACATACT
CCAATGTTTTATCCTTTGTCGAATCAATTCGTTCAAGGGTAATTATCAACGGAGTGACTGCTAGGTCTGAGTGG
GATGTTGACAAATCTCTTTTGCAATCCTTGTCCATGACATTTTTCTTGCATACTAAGCTTGCCGTTTTAAAAGAC
GAAATGTTAATCAGCAAGTTTAGTTTGGGGCCAAAATCAGTAAGCCAGCATGTATGGGATGAGATTTCCCTGG
CTTTTGGAAACGCATTTCCATCGATCAAGGAGAGACTGCTAAATCGGAAACTAATTAAAGTGTCGGGAGACGC
ATTAGAAATCAGGGTGCCTGATTTATATGTGACTTTTCACGATAGATTAGTGACTGAGTACAAAACATCGGTG
GATATGCCAGTGCTTGATATCAGAAAGAGAATGGAGGAGACTGAGGTTATGTACAATGCATTGTCTGAGCTA
TCTGTGCTCAAGGAGTCGGACAAGTTCGACGTTGATGTTTTTTCCCGGATGTGCCAGACTTTGGAGGTAGACC
CAATGACTGCAGCAAAGGTTATAGTGGCAGTGATGAGCAACGAGAGCGGACTGACTCTTACATTCGAACAGC
CAACTGAAGCAAATGTCGCATTGGCACTTAAAGATTCAGAAAAAGCCTCTGAGGGTGCACTAGTGGTTACTTC
TAGAGATGTTGAAGAACCATCCATGAAGGGTTCAATGGCAAGAGGAGAGTTACAATTGGCCGGTCTGTCTGG
AGACCAACCAGAGTCTTCCTATACTCGGAACGAGGAAATAGAGTCATTAGAGCAATTCCACATGGCAACGGCT
AGTTCGTTAATTCGGAAACAGATGAGTTCGATTGTGTACACGGGCCCCATTAAAGTTCAGCAAATGAAAAACT
TTATTGATAGCCTGGTAGCATCACTCTCTGCTGCGGTGTCGAACCTAGTCAAGATCCTAAAGGATACAGCTGCT
ATAGATCTCGAAACCCGTCAGAAGTTTGGAGTCTTAGATGTTGCGACCAAAAGATGGTTAATTAAACCTTTAG
CCAAAAATCACGCATGGGGCGTTATTGAAACACATGCTAGGAAGTACCACGTTGCACTTTTGGAGTATGATGA
GCATGGAGTGGTAACTTGCGACAGTTGGAGAAGGGTGGCCGTGAGTTCTGAGTCAATGGTTTATTCTGATAT
GGCAAAGCTCAGAACACTGAGGAGATTATTAAGAGATGGTGAGCCTCATGTCAGCAGTGCTAAAGTCGTCCT
AGTTGACGGTGTCCCGGGTTGTGGAAAGACAAAAGAGATTCTCTCGAAAGTAAATTTTGAGGAAGATCTAAT
CTTAGTACCGGGTAAGCAGGCTGCTGAAATGATAAAGAGGCGTGCTAATGCGTCAGGAATAATTCAAGCCAC
AAGAGATAATGTTCGTACTGTTGATTCATTTATAATGAATTACGGTAAAGGAACACGCTGTCAGTTCAAAAGG
TTATTTATCGACGAAGGTCTGATGTTGCACACTGGTTGTGTGAATTTTCTTGTTTCTATGTCTCTGTGCGAAATT
GCATATGTATATGGAGACACACAACAAATTCCATACATCAACAGAGTATCCGGTTTTCCGTACCCTGCACATTT
TGCAAAAATAGAGGTTGATGAGGTGGAAACTCGCAGAACTACGCTGCGTTGTCCAGCCGACATTACCCACTAT
CTTAACAGAAGGTACGAAGGACATGTCATGTGTACATCGTCGGTTAAAAAGTCAGTTTCTCAGGAAATGGTGA
GCGGGGCCGCAATGATCAATCCTGTATCTAAGCCATTGAATGGGAAAATTTTGACTTTCACTCAGTCCGATAA

AGAGGCGCTGCTTTCTCGAGGATATACGGACGTCCATACAGTACATGAGGTACAAGGTGAGACATATGCAGA
TGTGTCGTTGGTCAGATTGACTCCGACACCTGTATCTATCATCGCAGGAGATAGTCCGCACGTTCTCGTAGCTT
TGTCAAGGCATACCCAAACATTGAAGTATTACACCGTAGTGATGGATCCTCTTGTAAGTATAATTAGGGATTTA
GAAAAACTTAGTTCTTACTTGTTAGATATGTATAAAGTAGATGCAGGGACCCAATAGCAATTACTGGTAGACT
CCGTGTTTAAAGGTTCTAATCTTTTTGTTGCAGCACCAAAGACTGGAGATATCTCAGATATGCAATTTTACTAT
GATAAGTGTCTCCCAGGTAATAGCACCATGTTAAATAACTATGATGCTGTTACCATGAGGTTGACTGACATTTC
TCTTAATGTCAAAGATTGCATATTGGATTTCTCTAAGTCTGTGGCTGCACCGAAGGATCCGATCAAACCACTGA
TTCCAATGGTACGAACGGCGGCAGAAATGCCACGCCAGACTGGACTATTGGAAAATTTGGTGGCAATGATCA
AAAGAAACTTTAATTCACCGGAGTTATCAGGAATAATCGACATTGAGAATACTGCATCTTTGGTAGTAGATAA
ATTTTTTGATAGTTACTTGCTTAAAGAAAAAAGAAAACCAAATAAAAATGTTTCTTTATTTTGTAGAGAGTCTCT
CAATAGATGGTTAGAGAAGCAGGAGCAAGTGACCATTGGTCAGCTTGCAGATTTTGATTTTGTGGATCTTCCT
GCCGTTGATCAGTACAGGCATATGATTAAAGCGCAACCTAAGCAGAAGCTGGATACATCAATTCAAAGCGAAT
ATCCGGCCTTGCAGACGATTGTGTATCATTCGAAAAAGATCAACGCAATCTTCGGTCCTTTGTTCAGTGAGCTC
ACAAGGCAAATGCTCGAAAGCATAGACTCAAGTAAGTTTTTGTTCTTTACAAGGAAGACGCCAGCTCAAATCG
AGGATTTCTTCGGAGATCTCGATAGCCATGTCCCTATGGATATCTTGGAGTTGGATATTTCGAAGTATGACAAA
TCTCAGAACGAGTTCCACTGTGCAGTAGAGTATGAAATATGGAGAAGACTTGGATTAGAAGATTTTCTGGGA
GAAGTTTGGAAACAAGGCCACAGAAAAACTACTCTTAAAGATTACACAGCTGGTATTAAAACGTGTTTATGGT
ACCAGAGAAAGAGTGGGGACGTTACAACATTCATCGGTAATACGGTGATTATTGCTGCTTGTTTAGCTTCCAT
GTTGCCCATGGAGAAAATAATCAAAGGTGCATTTTGCGGAGATGACAGTTTACTATACTTCCCAAAAGGTTGT
GAGTTTCCCGACATACAGCATACAGCTAACCTTATGTGGAATTTCGAGGCTAAGCTATTCAGAAAGCAGTATG
GTTATTTCTGTGGAAGGTACGTGATACATCATGACAGAGGGTGTATTGTTTATTACGACCCTTTGAAGTTGATT
TCTAAACTTGGTGCTAAACACATCAAGGATTGGGATCACTTAGAAGAGTTCAGAAGATCCCTTTGTGATGTTG
CAATTTCGTTGAACAACTGTGCGTATTACACGCAGTTGGACGACGCTGTGAGTGAGGTCCATAAAACCGCACC
CCCGGGTTCGTTTGTTTATAAAAGTTTAGTTAAATATCTGTCCGATAAGGTTCTTTTTAGAAGTTTGTTTATAGA
TGGCTCTTGTAAAGGGTAAAGTCAATATTAATGAGTTCATAGACTTGTCAAAATCAGAAAAATTTTTCCCGTCT
ATGTTCACACCTGTTAAGAGTGTCATGATCTCCAAGGTTGATAAGATATTGGTTCATGAAGATGAATCTTTGTC
CGAAGTCAATTTACTCAAAGGTGTAAAACTCATTAATGGTGGCTATGTACATCTTGCTGGTCTTGTAGTGACAG
GTGAATGGAATTTGCCAGATAATTGTCGTGGTGGTGTCAGTGTCTGTTTGGTCGATAAGAGAATGGAGAGAG
CGGACGAGGCAACTCTTGCTTCATACTATACCGCAGCGGCTAAGAAAAGGTTTCAGTTCAAAGTCGTTCCAAA
TTACAACATCACTACCAAGGACGCAGAAAAGGCAGTTTGGCAAGTACTAGTTAATATTAGAAATGTTAAAATT
GCTGCGGGTTACTGTCCGCTGTCATTAGAATTTGTGTCAGTGTGTATTGTTTATAAAAATATTATAAAACTCGG
TTTGAGAGAGAAAATTACGAGCGTCACGGAAGGAGGGCCCATGGAACTATCAGAAGAAGTTGTTGATGAGTT
CATGGAAGAAGTCCCGATGTCTGTAAGGCTTGCAAAATTTCGTTCGAAGACCGGGAAAAAGTTTAGTAGTAA
AAGTGAGAATAATAGTGGTAATAATAGGCCGAAACCAGACAAAAACCAAAGGAAGGAAAAGGGTTTAAAAG
TTAGGGTTGAGAAGGATAATTTAATTGATAATGAATTGGAGACTTACGTCGCCGATTCAGATTCGTATTAAAT
ATGTCTTACACAATCGCAACTCCATCGCAATTTGTGTTTTTGTCATCAGCATGGGCCGACCCTATAGAATTAATA
AATTTATGTACTAATTCACTAGGCAATCAGTTCCAAACACAACAAGCTAGAACAACCGTTCAACGGCAATTTAG
CGAAGTGTGGAAACCTGTCCCTCAAGTCACTGTTAGGTTTCCTGACAGTGGTTTTAAGGTGTATAGGTACAAT
GCGGTACTAGATCCTCTAGTTACTGCTTTGTTGGGAGCTTTCGATACTAGAAATAGGATTATAGAAGTCGAAA
ATCAGGCGAACCCGACAACCGCCGAAACGTTAGACGCTACTCGTAGAGTAGATGACGCAACGGTGGCTATAA
GGAGCGCTATAAATAATTTAGTAGTAGAATTGGTCAAAGGAACAGGTTTGTACAATCAGAGCACATTTGAAA
GTGCATCCGGTTTACAATGGTCCTCTGCACCTGCATCTTGA

#OM515269

ATGGCATACACACAGACAGCTACCACATCCGCTTTGCTCGACACTGTCCGAGGTAACAATACCTTGGTCAACG
ATCTTGCGAAGCGGCGTCTTTATGACACAGCGGTCGACGAGTTCAACGCTCGTGATCGCAGGCCCAAAGTAA
ATTTTTCCAAAGTAATAAGTGAGGAACAGACGCTTATTGCTACTAGGGCATATCCAGAATTCCAGATAACCTTC
TATAATACGCAGAACGCCGTGCATTCGCTTGCCGGTGGACTACGATCCTTAGAACTGGAATATCTAATGATGC
AGATCCCGTACGGATCACTCACATATGATATAGGTGGGAATTTTGCATCTCATCTGTTCAAAGGACGGGCATA
TGTTCACTGCTGTATGCCCAATCTTGATGTCCGCGACATAATGCGGCACGAAGGCCAGAAAGACAGTATAGAA
TTATACCTTTCCAGGCTTGAGCGGGGCAACAAAGTTGTCCCAAATTTCCAAAAGGAAGCTTTTGACAGATACG
CTGAAACGCCAGACGAAGTTGTCTGTCACAGTACCTTTCAAACGTGTACGCACCAGCAGGTGGAAAACACAG
GCAGGGTGTATGCTATTGCATTGCACAGTATATACGATATACCTGCTGATGAATTCGGAGCGGCACTTTTAAG
GAAAAATGTCCATGTTTGTTACGCCGCCTTCCACTTTTCCGAGAATTTACTTCTCGAAGATTCACACGTCAACCT
TGACGAAATCAACGCGTGTTTTTCGCGTGATGGAGACAAGCTGACTTTTTCTTTCGCATCTGAGAGCACTTTAA
ATTATTGTCATAGTTATTCTAATATTTTAAAATACGTGTGCAGAACTTACTTCCCGGCATCTAATAGAGAGGTCT
ACATGAAGGAGTTTTTGGTCACCAGGGTTAACACCTGGTTTTGTAAGTTTTCTAGGATAGATACTTTTTTATTAT
ACAAGGGGGTAGCCCACAAAGGTGTAAATAGTGAGCAATTTTACAGCGCAATGGAAGATGCATGGCACTACA
AAAAGACTCTTGCAATGTGTAACAGCGAGAGGATTCTTCTTGAAGATTCCTCATCGGTCAATTACTGGTTCCCA
AAAATGAGAGATATGGTCATAGTTCCTCTATTCGACATATCTCTCGACACCAGTAAAAGGACCCGCAAAGAAG
TCTTAGTGTCAAAGGATTTTGTATTCACAGTTTTAAATCACATTCGCACTTATCAAGCCAAGGCACTTACATACT
CCAATGTTTTATCCTTTGTCGAATCAATTCGTTCAAGGGTAATTATCAACGGAGTGACTGCCAGGTCTGAGTGG
GATGTTGACAAATCTCTTTTGCAATCCTTGTCCATGACATTTTTCTTGCATACTAAGCTTGCCGTTTTAAAAGAC
GAATTGTTAATCAGCAAGTTTAGTTTGGGGCCAAAATCAGTAAGCCAGCATGTATGGGATGAGATTTCCCTGG
CTTTTGGAAACGCATTTCCATCGATCAAGGAGAGACTGCTAAATCGGAAACTAATTAAAGTGTCGGGAGACGC
ATTAGAAATCAGGGTGCCTGATTTATATGTGACTTTTCACGATAGATTAGTGACTGAGTACAAAACATCGGTG
GATATGCCAGTGCTTGATATCAGAAAGAGAATGGAGGAGACTGAGGTTATGTACAATGCACTGTCTGAGCTA
TCTGTGCTCAAGGAGTCGGACAAGTTCGACGTTGATGTTTTTTCCCGGATGTGCCAGACTTTGGAGGTAGACC
CAATGACTGCAGCAAAGGTTATTGTGGCAGTGATGAGCAACGAGAGCGGACTGACTCTTACATTCGAACAGC
CAACTGAAGCAAATGTCGCATTGGCACTTAAAGATTCAGAAAAAGCCTCTGAGGGTGCACTAGTGGTTACTTC
TAGAGATGTTGAAGAACCGTCCATGAAGGGTTCAATGGCAAGAGGAGAGTTACAATTGGCCGGTCTGTCTGG
AGACCAACCAGAGTCTTCCTATACTCGGAACGAGGAAATAGAGTCATTAGAGCAATTCCACATGGCAACGGCT
AGTTCGTTAATTCGGAAACAGATGAGTTCGATTGTGTACACGGGCCCCATTAAAGTTCAGCAAATGAAAAACT
TTATTGATAGCCTGGTAGCATCACTCTCTGCTGCGGTGTCGAACCTAGTCAAGATCCTAAAGGATACAGCTGCT
ATAGATCTCGAAACCCGTCAGAAGTTTGGAGTCTTAGATGTTGCGACCAAAAGATGGTTAATTAAACCTTTAG
CCAAGAATCACGCATGGGGCGTTATTGAAACACATGCTAGGAAGTACCACGTTGCACTTTTGGAGTATGATGA
GCATGGAGTGGTAACTTGCGACAGTTGGAGAAGGGTGGCCGTGAGTTCTGAGTCAATGGTTTATTCTGATAT
GGCAAAGCTCAGAACACTGAGGAGATTATTAAGGGATGGTGAGCCTCATGTCAGCAGTGCTAAAGTCGTCCT
AGTTGACGGTGTCCCGGGTTGTGGAAAGACAAAAGAGATTCTCTCAAAAGTAAATTTTGAGGAAGATCTAAT
CTTAGTACCGGGTAAGCAGGCTGCTGAAATGATAAAGAGGCGTGCTAATGCGTCAGGAATAATTCAAGCCAC
AAGAGATAATGTTCGTACTGTTGATTCATTTATAATGAATTACGGTAAAGGAACACGCTGTCAGTTCAAAAGG
TTATTTATCGACGAAGGTCTGATGTTGCACACTGGTTGTGTGAATTTTCTTGTTTCTATGTCTCTGTGCGAAATT
GCATATGTTTATGGAGACACACAACAAATTCCATACATCAACAGAGTATCCGGTTTTCCGTACCCTGCACATTT
TGCAAAAATAGAGGTTGATGAGGTGGAAACTCGCAGAACTACGCTGCGTTGTCCAGCCGACATTACCCACTAT
CTTAACAGAAGGTACGAAGGACATGTCATGTGTACATCGTCGGTTAAAAAGTCAGTTTCTCAGGAAATGGTGA
GCGGGGCCGCAATGATCAATCCTGTATCTAAGCCACTGAATGGGAAAGTTTTGACTTTCACTCAGTCTGATAA
AGAGGCGCTGCTTTCTCGAGGATATACGGACGTCCATACAGTACATGAGGTACAAGGTGAGACATATGCAGA
TGTGTCGTTGGTCAGATTGACTCCGACACCTGTATCTATCATCGCAGGAGATAGTCCGCACGTTCTCGTAGCTT
TGTCAAGGCATACCCAAACATTGAAGTATTACACCGTAGTGATGGATCCTCTTGTAAGTATAATTAGGGATTTA
GAAAAACTTAGTTCTTACTTGTTAGATATGTATAAAGTAGATGCAGGGACCCAATAGCAATTACAGGTAGACT
CCGTGTTTAAAGGTTCTAATCTTTTTGTTGCAGCACCAAAGACTGGAGATATCTCAGATATGCAATTTTACTAT
GATAAGTGTCTCCCAGGTAATAGCACCATGTTAAATAACTATGATGCTGTTACCATGAGGTTGACTGACATTTC

TCTTAATGTCAAAGATTGCATATTGGATTTCTCTAAGTCTGTGGCTGCACCGAAGGATCCGATCAAACCACTGA
TTCCAATGGTACGAACGGCGGCAGAAATGCCACGCCAGACTGGACTATTGGAAAATTTGGTGGCGATGATCA
AAAGAAACTTTAATTCACCGGAGTTATCAGGAATAATCGACATTGAGAATACTGCATCTTTAGTAGTAGATAA
ATTTTTTGATAGTTACTTGCTTAAAGAAAAAAGAAAACCAAATAAAAATGTTTCTTTATTTAGTAGAGAGTCTC
TCAATAGATGGTTAGAGAAGCAGGAGCAAGTGACCATTGGTCAGCTTGCAGATTTTGATTTTGTGGATCTTCC
TGCCGTTGATCAGTACAGGCATATGATTAAAGCGCAACCTAAGCAGAAGCTGGATACATCAATTCAAAGCGAA
TATCCGGCCTTGCAGACGATTGTGTATCATTCGAAAAAGATCAACGCAATCTTCGGTCCTTTGTTCAGTGAGCT
CACAAGGCAAATGCTCGAAAGCATAGACTCAAGTAAGTTTTTGTTCTTTACAAGGAAGACGCCAGCTCAAATT
GAGGATTTCTTCGGAGATCTCGATAGCCATGTCCCTATGGATATCTTGGAGTTGGATATTTCGAAGTATGACA
AATCTCAGAACGAGTTCCACTGTGCAGTAGAGTATGAAATATGGAGAAGACTTGGATTAGAAGATTTTCTGG
GAGAAGTTTGGAAACAAGGCCACAGGAAAACTACTCTTAAAGATTACACAGCTGGTATTAAAACGTGTTTATG
GTACCAGAGAAAGAGTGGGGACGTTACAACATTCATCGGTAATACGGTGATTATTGCTGCTTGTTTAGCTTCC
ATGTTGCCCATGGAGAAAATAATCAAAGGTGCATTTTGCGGAGATGACAGTTTACTATACTTCCCAAAAGGTT
GTGAGTTTCCTGACATACAGCATACAGCCAACCTTATGTGGAATTTCGAGGCTAAGCTATTCAGAAAGCAGTA
TGGTTATTTCTGTGGAAGGTACGTGATACATCATGACAGAGGGTGTATTGTTTATTATGACCCTTTGAAGTTGA
TTTCTAAACTTGGTGCTAAACACATCAAGGATTGGGATCACTTAGAAGAGTTCAGAAGATCCCTTTGTGATGTT
GCAAATTCGTTGAACAACTGTGCGTATTACACGCAGTTGGACGACGCTGTGAGTGAGGTCCATAAAACCGCAC
CCCCGGGTTCGTTTGTGTATAAAAGTTTAGTTAAATATCTGTCCGATAAGGTTCTTTTTAGAAGTTTGTTTATAG
ATGGCTCTTGTTAAGGGTAAAGTCAATATTAATGAGTTCATAGACTTGTCAAAATCAGAAAAAATTTCTTCCGTC
TATGTTCACACCTGTTAAGAGTGTCATGATCTCCAAGGTTGATAAGATATTGGTTCATGAAGATGAATCTTTGT
CCGAAGTCAATTTACTCAAAGGTGTAAAACTCATTGATGGTGGCTATGTACATCTTGCTGGTCTTGTGGTGACA
GGTGAATGGAATTTGCCAGATAATTGTCGTGGTGGTGTCAGTGTCTGTTTGGTCGATAAGAGAATGGAGAGA
GCGGACGAGGCAACTCTTGCTTCATACTATACCGCAGCGGCTAAGAAAAGGTTTCAGTTCAAAGTCGTTCCAA
ATTACAACATCACTACCAAGGACGCAGAAAAGGCAGTTTGGCAAGTACTAGTTAATATTAGAAATGTTAAAAT
TGCTGCGGGTTACTGTCCGCTGTCATTAGAATTTGTGTCAGTGTGTATTGTTTATAAAAATATTATAAAACTCG
GTTTGAGAGAGAAAATTACGAGCGTCACGGATGGAGGGCCCATGGAACTATCAGAAGAAGTTGTTGATGAG
TTCATTGAAGAAGTCCCGATGTCTGTGAGGCTTGCAAAATTTCGTTCGAAGACCGGAAAAAAGTTTAGTAGTA
AAAGTGAGAATAATAGTGGTAATAATAGGCCGAAACCAAACAAAAACCAAAGGAAGGAAAAGGGTTTAAAA
GTTAGGGTTGAGAAGGATAATTTAATTGATAATGAATTGGAGACTTACATCGCCGATTCAGATTCGTATTAAA
TATGTCTTACACAATCGCAACTCCATCGCAATTTGTGTTTTTGTCATCAGCATGGGCCGACCCTATAGAATTAAT
AAATTTATGTACTAATTCACTAGGTAATCAGTTCCAAACACAACAAGCTAGAACAACCGTTCAACGGCAATTTA
GCGAAGTGTGGAAACCTGTCCCTCAAGTCACTGTTAGGTTTCCTGACAGTGGTTTTAAGGTGTATAGGTACAA
TGCGGTACTAGATCCTCTAGTTACTGCTTTGTTAGGAGCTTTCGATACTAGAAATAGGATTATAGAAGTCGAAA
ATCAGGCGAACCCGACAACCGCCGAAACGTTAGACGCTACTCGTAGAGTAGATGACGCAACGGTGGCTATAA
GGAGCGCTATAAATAATTTAGTAGTAGAATTGGTCAAAGGAACAGGTTTGTACAATCAGAGCACATTTGAAA
GTGCATCCGGTTTACAATGGTCCTCTGCACCTGCATCTTGA

#OM515270

1

ATGGCATACACACAGACAGCTACCACATCCGCTTTGCTCGACACTGTCCGAGGTAACAATACCTTGGTCAACG
ATCTTGCGAAGCGGCGTCTTTATGATACAGCGGTCGACGAGTTCAACGCTCGTGATCGCAGGCCCAAAGTAAA
TTTTTCCAAAGTAATAAGTGAGGAACAGACGCTTATTGCTACTAGGGCATATCCTGAATTCCAGATAACCTTCT
ATAATACGCAGAACGCCGTGCATTCGCTTGCCGGTGGACTACGATCCTTAGAACTGGAATATCTAATGATGCA
GATCCCGTACGGATCACTCACATATGATATAGGTGGGAATTTTGCATCTCATCTGTTCAAAGGACGGCATAT

GTTCACTGCTGTATGCCCAATCTTGATGTCCGCGACATAATGCGGCACGAAGGCCAGAAAGACAGTATAGAAT
TATACCTTTCCAGGCTTGAGCGGGGCAACAAAGTTGTCCCAAATTTCCAAAAGGAAGCTTTTGACAGATACGC
TGAAACGCCAGACGAAGTTGTCTGTCACAGTACCTTCCAAACGTGTACACACCAGCAGGTGGAAAACACAGG
CAGGGTGTATGCTATTGCATTGCACAGTATATACGATATACCTGCTGATGAATTCGGAGCGGCACTTTTAAGG
AAAAATGTCCATGTTTGTTACGCCGCCTTCCACTTTTCCGAGAATTTACTTCTCGAAGATTCACACGTCAACCTT
GACGAAATCAACGCGTGTTTTTCGCGTGATGGAGACAAGCTGACTTTTTCTTTCGCATCTGAGAGCACTTTAAA
TTATTGTCATAGTTATTCTAATATTTTAAAATACGTGTGCAAAACTTACTTCCCGGCATCTAATAGAGAGGTCTA
CATGAAGGAGTTTTTGGTCACCAGGGTTAACACCTGGTTTTGTAAGTTTTCTAGGATAGATACTTTTTTATTATA
CAAGGGGGTAGCCCACAAAGGTGTAAATAGTGAGCAATTTTACAGCGCAATGGAAGATGCATGGCACTACAA
AAAGACTCTTGCAATGTGTAACAGCGAGAGGATTCTTCTTGAAGATTCCTCATCGGTCAATTACTGGTTCCCAA
AAATGAGAGATATGGTCATAGTTCCTCTATTCGACATATCTCTCGACACTAGTAAAAGGACCCGCAAAGAAGT
CTTAGTGTCAAAGGATTTTGTATTCACAGTTTTAAATCACATTCGCACTTATCAAGCCAAGGCACTTACATACTC
CAATGTTTTATCCTTTGTCGAATCAATTCGTTCAAGGGTAATTATCAACGGAGTGACTGCCAGGTCTGAGTGG
GATGTTGACAAATCTCTTTTGCAATCCTTGTCCATGACATTTTTCTTGCATACTAAGCTTGCCGTTTTAAAAGAC
GAATTGTTAATCAGCAAGTTTAGTTTGGGGCCAAAATCAGTAAGCCAGCATGTATGGGATGAGATTTCCCTGG
CTTTTGGAAACGCATTTCCATCGATCAAGGAGAGACTGCTAAATCGGAAACTAATTAAAGTGTCGGGAGACGC
ATTAGAAATCAGGGTGCCTGATTTATATGTGACTTTTCACGATAGATTAGTGACTGAGTACAAAACATCGGTG
GATATGCCAGTGCTTGATATCAGAAAGAGAATGGAGGAGACTGAGGTTATGTACAATGCATTGTCTGAGCTA
TCTGTGCTCAAGGAGTCGGACAAGTTCGACGTTGATGTTTTTTCCCGGATGTGCCAGACTTTGGAGGTAGACC
CAATGACTGCAGCAAAGGTTATTGTGGCAGTGATGAGCAACGAGAGCGGACTGACTCTTACATTCGAACAGC
CAACTGAAGCAAATGTCGCATTGGCACTTAAAGATTCAGAAAAAGCCTCTGAGGGTGCACTAGTGGTTACTTC
TAGAGATGTTGAAGAACCATCCATGAAGGGTTCAATGGCAAGAGGAGAGTTACAATTGGCCGGTCTGTCTGG
AGACCAACCAGAGTCTTCCTATACTCGGAACGAGGAAATAGAGTCATTAGAGCAATTTCACATGGCAACGGCT
GGTTCGTTAATTCGGAAACAGATGAGTTCGATTGTGTACACGGGCCCCATTAAAGTTCAGCAAATGAAAAACT
TTATTGATAGCCTGGTAGCATCACTCTCTGCTGCGGTGTCGAACCTAGTCAAGATCCTAAAGGATACAGCTGCT
ATAGATCTCGAAACCCGTCAGAAGTTTGGAGTCTTAGATGTTGCGACCAAAAGATGGTTAATTAAACCTTTAG
CCAAGAATCACGCATGGGGCGTTATTGAAACACATGCTAGGAAGTACCACGTTGCACTTTTGGAGTATGATGA
GCATGGAGTGGTAACTAGCGACAGTTGGAGAAGGGTGGCCGTGAGTTCTGAGTCAATGGTTTATTCTGATAT
GGCAAAGCTCAGAACACTGAGGAGATTATTAAGAGATGGTGAGCCTCATGTCAGCAGTGCTAAAGTCGTCCT
AGTTGACGGTGTCCCGGGTTGTGGAAAGACAAAAGAGATTCTCTCGAAAGTAAATTTTGAGGAAGATCTAAT
CTTAGTACCGGGTAAGCAGGCTGCTGAAATGATAAAGAGGCGTGCTAATGCGTCAGGAATAATTCAAGCCAC
AAGAGATAATGTTCGTACTGTTGATTCATTTATAATGAATTACGGTAAAGGAACACGCTGTCAGTTCAAAAGG
TTATTTATCGACGAAGGTCTGATGTTGCACACTGGTTGTGTGAATTTTCTTGTTTCAATGTCTCTGTGCGAAATT
GCATATGTTTATGGAGACACACAACAAATTCCATACATCAACAGAGTATCCGGTTTTCCGTACCCTGCACATTT
TGCAAAAATAGAGGTTGATGAGGTGGAAACTCGCAGAACTACGCTGCGTTGTCCAGCCGACATTACCCACTAT
CTTAACAGAAGGTACGAAGGACATGTCATGTGTACATCGTCGGTTAAAAAGTCAGTTTCTCAGGAAATGGTGA
GCGGGGCCGCAATGATCAATCCTGTATCTAAGCCATTGAATGGGAAAGTTTTGACTTTCACTCAGTCTGATAA
AGAGGCGCTGCTTTCTCGAGGATATACGGACGTCCATACAGTACATGAGGTACAAGGTGAGACATATGCAGA
TGTGTCGTTGGTCAGATTGACTCCGACACCTGTATCTATCATCGCAGGAGATAGTCCGCACGTTCTCGTAGCTT
TGTCAAGGCATACCCAAACATTGAAGTATTACACCGTAGTGATGGATCCTCTTGTAAGTATAATTAGGGATTTA
GAAAAACTTAGTTCTTACTTGTTAGATATGTATAAAGTAGATGCAGGGACCCAATAGCAATTACAGGTAGACT
CCGTGTTTAAAGGTTCTAATCTTTTTGTTGCAGCACCAAAGACTGGAGATATCTCAGATATGCAATTTTACTAT
GATAAGTGTCTCCCAGGTAATAGCACCATGTTAAATAACTATGATGCTGTTACCATGAGGTTGACTGACATTTC
TCTTAATGTCAAAGATTGCATATTGGATTTCTCTAAGTCTGTGGCTGCACCGAAGGATCCGATCAAACCACTGA
TTCCAATGGTACGAACGGCGGCAGAAATGCCACGCCAGACTGGACTATTGGAAAATTTGGTGGCGATGATCA
AAAGAAACTTTAATTCACCGGAGTTATCAGGAATAATCGACATTGAGAATACTGCATCTTTAGTAGTAGATAA
ATTTTTTGATAGTTACTTGCTTAAAGAAAAAAGAAAACCAAATAAAAATGTTTCTTTATTTTGTAGAGAGTCTCT
CAATAGATGGTTAGAGAAGCAGGAGCAAGTGACCATTGGTCAGCTTGCAGATTTTGATTTTGTGGATCTTCCT

GCCGTTGATCAGTACAGGCATATGATTAAAGCGCAACCTAAGCAGAAGCTGGATACATCAATTCAAAGCGAAT
ATCCGGCCTTGCAGACGATTGTGTATCATTCGAAAAAGATCAACGCAATCTTCGGTCCTTTGTTCAGTGAGCTC
ACAAGGCAAATGCTCGAAAGCATAGACTCAAGTAAGTTTTTGTTCTTTACAAGGAAGACGCCAGCTCAAATTG
AGGATTTCTTCGGAGATCTCGATAGCCATGTCCCTATGGATATCTTGGAGTTGGATATTTCGAAGTATGACAAA
TCTCAGAACGAGTTCCACTGTGCAGTAGAGTATGAAATATGGAGAAGACTTGGATTAGAAGATTTTCTGGGA
GAAGTTTGGAAACAAGGCCACAGAAAAACTACTCTTAAAGATTACACAGCTGGTATTAAAACGTGTTTATGGT
ACCAGAGAAAGAGTGGGGACGTTACAACATTCATCGGTAATACGGTGATTATTGCTGCTTGTTTAGCTTCCAT
GTTACCCATGGAGAAAATAATCAAAGGTGCATTTTGCGGAGATGACAGTTTACTATACTTCCCAAAAGGTTGT
GAGTTTCCTGACATACAGCATACAGCCAACCTTATGTGGAATTTCGAGGCTAAGCTATTCAGAAAGCAGTATG
GTTATTTCTGTGGAAGGTACGTGATACATCATGACAGAGGGTGTATTGTTTATTATGACCCTTTGAAGTTGATT
TCTAAACTTGGTGCTAAACACATCAAGGATTGGGATCACTTAGAAGAGTTCAGAAGATCCCTTTGTGATGTTG
CAAATTCGTTGAACAACTGTGCGTATTACACGCAGTTGGACGACGCTGTGAGTGAGGTCCATAAAACCGCACC
CCCGGGTTCGTTTGTATATAAAAGTTTAGTTAAATATCTGTCCGATAAGGTTCTTTTTAGAAGTTTGTTTATAGA
TGGCTCTTGTTAAGGGTAAAGTCAATATTAGTGAGTTCATAGACTTGTCAAAATCAGAAAAATTTCTCCCGTCT
ATGTTCACACCTGTTAAGAGTGTCATGATCTCCAAGGTTGATAAGATATTGGTTCATGAAGATGAATCTTTGTC
CGAAGTCAATTTACTCAAAGGTGTAAAACTCATTGATGGTGGCTATGTACATCTTGCTGGTCTTGTGGTGACA
GGTGAATGGAATTTGCCAGATAATTGTCGTGGTGGTGTCAGTGTCTGTTTGGTCGATAAAAGAATGGAGAGA
GCGGACGAGGCAACTCTTGCTTCATACTATACCGCAGCGGCTAAGAAAAGGTTTCAGTTCAAAGTCGTTCCAA
ATTACAACATCACTACCAAGGACGCAGAAAAGGCAGTTTGGCAAGTACTAGTTAATATTAGAAATGTTAAAAT
TGCTGCGGGTTACTGTCCGCTGTCATTAGAATTTGTGTCAGTGTGTATTGTTTATAAAAATATTATAAAACTCG
GTTTGAGAGAGAAAATTACGAGCGTCACGGATGGAGGGCCCATGGAACTATCAGAAGAAGTTGTTGATGAG
TTCATGGAACAAGTCCCGATGTCTGTAAGGCTTGCAAAATTTCGTTCGAAGACCGGGAAAAAGTTTAGTAGTA
AAAGTGAGAATAATAGTGGTAATAATAGGCCGAAACCAAACAAAAACCAAAGGAAGGAAAAGGGTTTAAAA
GTTAGGGTTGAGAAGGATAATTTAATTGATAATGAATTGGAGACTTACGTCGCCGATTCAGATTCGTATTAAA
TATGTCTTACACAATCGCAACTCCATCGCAATTTGTGTTTTTGTCATCAGCATGGGCCGACCCTATAGAATTAAT
AAATTTATGTACTAATTCACTAGGTAATCAATTCCAAACACAACAAGCTAGAACAACCGTTCAACGGCAATTTA
GCGAAGTGTGGAAACCTGTCCCTCAAGTCACTGTTAGGTTTCCTGACAGTGGTTTTAAGGTGTATAGGTACAA
TGCGGTACTAGATCCTCTAGTTACTGCTTTGTTAGGAGCTTTCGATACTAGAAATAGGATTATAGAAGTCGAAA
ATCAGGCGAACCCGACAACCGCCGAAACGTTAGACGCTACTCGTAGAGTAGATGACGCAACGGTGGCTATAA
GGAGCGCTATAAATAATTTAGTAGTAGAATTGGTCAAAGGAACAGGTTTGTACAATCAGAGCACATTTGAAA
GTGCATCCGGTTTACAATGGTCCTCTGCACCTGCATCTTGA

#OM515272

1

ATGGCATACACACACAGACAGCTACCACATCCGCTTTGCTCGACACTGTCCGAGGTAACAATACCTTGGTCAACG
ATCTTGCGAAGCGGCGTCTTTATGACACAGCGGTCGACGAGTTCAACGCTCGTGATCGCAGGCCCAAAGTAA
ATTTTTCCAAAGTAATAAGTGAGGAACAGACGCTTATTGCTACTAGGGCATATCCAGAATTCCAGATAACCTTC
TATAATACGCAGAACGCCGTGCATTCGCTTGCCGGTGGACTACGATCCTTAGAACTGGAATATCTAATGATGC
AGATCCCGTACGGATCACTCACATATGATATAGGTGGGAATTTTGCATCTCATCTGTTCAAAGGACGGGCATA
TGTTCACTGCTGTATGCCCAATCTTGATGTCCGCGACATAATGCGGCACGAAGGCCAGAAAGACAGTATAGAA
TTATACCTTTCCAGGCTTGAGCGGGGCAACAAAGTTGTCCCAAATTTCCAAAAGGAAGCTTTTGACAGATACG
CTGAAACGCCAGACGAAATTGTCTGTCACAGTACCTTCCAAACGTGTACGCACCAGCAGGTGGAAAACACAG
GCAGGGTGTATGCTATTGCATTGCACAGTATATACGATATACCTGCTGATGAATTCGGAGCGGCACTTTTAAG
GAAAAATGTCCATGTTTGTTACGCCGCCTTCCACTTTTCCGAGAATTTACTTCTCGAAGATTCACACGTCAACCT

TGACGAAATCAACGCGTGTTTTTCGCGTGATGGAGACAAGCTGACTTTTTCTTTCGCATCTGAGAGCACTTTAA
ATTATTGTCATAGTTATTCTAATATTTTAAAATACGTGTGCAGAACTTACTTCCCGGCATCTAATAGAGAGGTCT
ACATGAAGGAGTTTTTGGTCACCAGGGTTAACACCTGGTTTTGTAAGTTTTCTAGGATAGATACTTTTTTATTAT
ACAAGGGGGTAGCCCACAAAGGTGTAAATAGTGAGCAATTTTACAGCGCAATGGAAGATGCATGGCACTACA
AAAAGACTCTTGCAATGTGTAACAGCGAGAGGATTATTCTTGAAGATTCCTCATCGGTCAATTACTGGTTCCCA
AAAATGAGAGATATGGTCATAGTTCCTCTATTCGACATATCTCTCGACACCAGTAAAAGGACCCGCAAAGAAG
TCTTAGTGTCAAAGGATTTTGTATTCACAGTTTTAAATCACATTCGCACTTATCAAGCCAAGGCACTTACATACT
CCAATGTTTTATCCTTTGTCGAATCAATTCGTTCAAGGGTAATTATCAACGGAGTGACTGCCAGGTCTGAGTGG
GATGTTGACAAATCTCTTTTGCAATCCTTGTCCATGACATTTTTCTTGCATACTAAGCTTGCCGTTTTAAAAGAC
GAATTGTTAATCAGCAAGTTTAGTTTGGGGCCAAAATCAGTAAGCCAGCATGTATGGGATGAGATTTCCCTGG
CTTTTGGAAACGCATTTCCATCGATCAAGGAGAGACTGCTAAATCGGAAACTAATTAAAGTGTCGGGAGACGC
ATTAGAAATCAGGGTGCCTGATTTATATGTGACTTTTCACGATAGATTAGTGACTGAGTACAAAACATCGGTG
GATATGCCAGTGCTTGATATCAGAAAGAGAATGGAGGAGACTGAGGTTATGTACAATGCATTGTCTGAGCTA
TCTGTGCTCAAGGAGTCGGACAAGTTCGACGTTGATGTTTTTTCCCGGATGTGCCAGACTTTGGAGGTAGACC
CAATGACTGCAGCAAAGGTTATTGTGGCAGTGATGAGCAACGAGAGCGGACTGACTCTTACATTCGAACAGC
CAACTGAAGCAAATGTCGCATTGGCACTTAAAGATTCAGAAAAAGCCTCTGAGGGTGCACTAGTGGTTACTTC
TAGAGATGTTGAAGAACCGTCCATGAAGGGTTCAATGGCAAGAGGAGAGTTACAATTGGCCGGTCTGTCTGG
AGACCAACCAGAGTCTTCCTATACTCGGAACGAGGAAATAGAGTCATTAGAGCAATTCCACATGGCAACGGCT
AGTTCGTTAATTCGGAAACAGATGAGTTCGATTGTGTACACGGGCCCCATTAAAGTTCAGCAAATGAAAAACT
TTATTGATAGCCTGGTAGCATCACTCTCTGCTGCGGTGTCGAACCTAGTCAAGATCCTAAAGGATACAGCTGCT
ATAGATCTCGAAACCCGTCAGAAGTTTGGAGTCTTAGATGTTGCGACCAAAAGATGGTTAATTAAACCTTTAG
CCAAGAATCACGCATGGGGCGTTATTGAAACACATGCTAGGAAGTACCACGTTGCACTTTTGGAGTATGATGA
GCATGGAGTGGTAACTTGCGACAGTTGGAGAAGGGTGGCCGTGAGTTCTGAGTCAATGGTTTATTCTGATAT
GGCAAAGCTCAGAACACTGAGGAGATTATTAAGGGATGGTGAGCCTCATGTCAGTAGTGCTAAAGTCGTCCT
AGTTGACGGTGTCCCGGGTTGTGGAAAGACAAAAGAGATTCTCTCAAAAGTAAATTTTGAGGAAGATCTAAT
CTTAGTACCGGGTAAGCAGGCTGCTGAAATGATAAAGAGGCGTGCTAATGCGTCAGGAATAATTCAAGCCAC
AAGAGATAATGTTCGTACTGTTGATTCATTTATAATGAATTACGGTAAAGGAACACGCTGTCAGTTCAAAAGG
TTATTTATCGACGAAGGTCTGATGTTGCACACTGGTTGTGTGAATTTTCTTGTTTCTATGTCTCTGTGCGAAATT
GCATATGTTTATGGAGACACACAACAAATTCCATACATCAACAGAGTATCCGGTTTTCCGTACCCTGCACATTT
TGCAAAAATAGAGGTTGATGAGGTGGAAACTCGCAGAACTACGCTGCGTTGTCCAGCCGACATTACCCACTAT
CTTAACAGAAGGTACGAAGGACATGTCATGTGTACATCGTCGGTTAAAAAGTCAGTTTCTCAGGAAATGGTGA
GCGGGGCCGCAATGATCAATCCTGTATCTAAGCCACTGAATGGGAAAGTTTTGACTTTCACTCAGTCTGATAA
AGAGGCGCTGCTTTCTCGAGGATATACGGACGTCCATACAGTACATGAGGTACAAGGTGAGACATATGCAGA
TGTGTCGTTGGTCAGATTGACTCCGACACCTGTATCTATCATCGCAGGAGATAGTCCGCACGTTCTCGTAGCTT
TGTCAAGGCATACCCAAACATTGAAGTATTACACCGTAGTGATGGATCCTCTTGTAAGTATAATTAGGGATTTA
GAAAAACTTAGTTCTTACTTGTTAGATATGTATAAAGTAGATGCAGGGACCCAATAGCAATTACAGGTAGACT
CCGTGTTTAAAGGTTCTAATCTTTTTGTTGCAGCACCAAAGACTGGAGATATCTCAGATATGCAATTTTACTAT
GATAAGTGTCTCCCAGGTAATAGCACCATGTTAAATAACTATGATGCTGTTACCATGAGGTTGACTGACATTTC
TCTTAATGTCAAAGATTGCATATTGGATTTCTCTAAGTCTGTGGCTGCACCGAAGGATCCGATCAAACCACTGA
TTCCAATGGTACGAACGGCGGCAGAAATGCCACGCCAGACTGGACTATTGGAAAATTTGGTGGCGATGATCA
AAAGAAACTTTAATTCACCGGAGTTATCAGGAATAATCGACATTGAGAATACTGCATCTTTAGTAGTAGATAA
ATTTTTTGATAGTTACTTGCTTAAAGAAAAAAGAAAACCAAATAAAAATGTTTCTTTATTTAGTAGAGAGTCTC
TTAATAGATGGTTAGAGAAGCAGGAGCAAGTGACCATTGGTCAGCTTGCAGATTTTGATTTTGTGGATCTTCC
TGCCGTTGATCAGTACAGGCATATGATTAAAGCGCAACCTAAGCAGAAGTTGGATACATCAATTCAAAGCGAA
TATCCGGCCTTGCAGACGATTGTGTATCATTCGAAAAAGATCAACGCAATCTTCGGTCCTTTGTTCAGTGAGCT
CACAAGGCAAATGCTCGAAAGCATAGACTCAAGTAAGTTTTTGTTCTTTACAAGGAAGACGCCAGCTCAAATT
GAGGATTTCTTCGGAGATCTCGATAGCCATGTCCCTATGGATATCTTGGAGTTGGATATTTCGAAGTATGACA
AATCTCAGAACGAGTTCCACTGTGCAGTAGAGTATGAAATATGGAGAAGACTTGGATTAGAAGATTTTCTGG

GAGAAGTTTGGAAACAAGGCCACAGGAAAACTACTCTTAAAGATTACACAGCTGGTATTAAAACGTGTTTATG
GTACCAGAGAAAGAGTGGGGACGTTACAACATTCATCGGTAATACGGTGATTATTGCTGCTTGTTTAGCTTCC
ATGTTGCCCATGGAGAAAATAATCAAAGGTGCATTTTGCGGAGATGACAGTTTACTATACTTCCCAAAAGGTT
GTGAGTTTCCTGACATACAGCATACAGCCAACCTTATGTGGAATTTCGAGGCTAAGCTATTCAGAAAGCAGTA
TGGTTATTTCTGTGGAAGGTACGTGATACATCATGACAGAGGGTGTATTGTTTATTATGACCCTTTGAAGTTGA
TTTCTAAACTTGGTGCTAAACACATCAAGGATTGGGATCACTTAGAAGAGTTCAGAAGATCCCTTTGTGATGTT
GCAAATTCGTTGAACAACTGTGCGTATTACACGCAGTTGGACGACGCTGTGAGTGAGGTCCATAAAACCGCAC
CCCCGGGTTCGTTTGTGTATAAAAGTTTAGTTAAATATCTGTCCGATAAGGTTCTTTTTAGAAGTTTGTTTATAG
ATGGCTCTTGTTAAGGGTAAAGTCAATATTAATGAGTTCATAGACTTGTCAAAATCAGAAAAAATTTCTTCCGTC
TATGTTCACACCTGTTAAGAGTGTCATGATCTCCAAGGTTGATAAGATATTGGTTCATGAAGATGAATCTTTGT
CCGAAGTCAATTTACTCAAAGGTGTAAAACTCATTGATGGTGGCTATGTACATCTTGCTGGTCTTGTGGTGACA
GGTGAATGGAATTTGCCAGATAATTGTCGTGGTGGTGTCAGTGTCTGTTTGGTCGATAAGAGAATGGAGAGA
GCGGACGAGGCAACTCTTGCTTCATACTATACCGCAGCGGCTAAGAAAAGGTTTCAGTTCAAAGTCGTTCCAA
ATTACAACATCACTACCAAGGACGCAGAAAAGGCAGTTTGGCAAGTACTAGTTAATATTAGAAATGTTAAAAT
TGCTGCGGGTTACTGTCCGCTGTCATTAGAATTTGTGTCAGTGTGTATTGTTTATAAAAATATTATAAAACTCG
GTTTGAGAGAGAAAATTACGAGCGTCACGGATGGAGGGCCCATGGAACTATCAGAAGAAGTTGTTGATGAG
TTCATTGAAGAAGTCCCGATGTCTGTAAGGCTTGCAAGATTTCGTTCGAAGACCGGAAAAAAGTTTAGTAGTA
AAAGTGAGAATAATAGTGGTAATAATAGGCCGAAACCAAACAAAAACCAAAGGAAGGAAAAGGGTTTAAAA
GTTAGGGTTGAGAAGGATAATTTAATTGATAATGAATTGGAGACTTACATCGCCGATTCAGATTCGTATTAAA
TATGTCTTACACAATCGCAACTCCATCGCAATTTGTGTTTTTGTCATCAGCATGGGCCGACCCTATAGAATTAAT
AAATTTATGTACTAATTCACTAGGTAATCAGTTCCAAACACAACAAGCTAGAACAACCGTTCAACGGCAATTTA
GCGAAGTGTGGAAACCTGTCCCTCAAGTCACTGTTAGGTTTCCTGACAGTGGTTTTAAGGTGTATAGGTACAA
TGCGGTACTAGATCCTCTAGTTACTGCTTTGTTAGGAGCTTTCGATACTAGAAATAGGATTATAGAAGTCGAAA
ATCAGGCGAACCCGACAACCGCCGAAACGTTAGACGCTACTCGTAGAGTAGATGACGCAACGGTGGCTATAA
GGAGCGCTATAAATAATTTAGTAGTAGAATTGGTCAAAGGAACAGGTTTGTACAATCAGAGCACATTTGAAA
GTGCATCCGGTTTACAATGGTCCTCTGCACCTGCATCTTGA

#OM718704

1

ATGGCATACACACAGACAGCTACCACATCCGCTTTGCTCGACACTGTCCGAGGTAACAATACCTTGGTCAACG
ATCTTGCGAAGCGGCGTCTTTATGACACAGCGGTCGACGAGTTCAACGCTCGTGATCGCAGGCCCAAAGTAA
ATTTTTCCAAAGTAATAAGTGAGGAACAGACGCTTATTGCTACTAGGGCATATCCAGAATTCCAGATAACCTTC
TATAATACGCAGAACGCCGTGCATTCGCTTGCCGGTGGACTACGATCCTTAGAACTGGAATATCTAATGATGC
AGATCCCGTACGGATCACTCACATATGATATAGGTGGGAATTTTGCATCTCATCTGTTCAAAGGACGGGCATA
TGTTCACTGCTGTATGCCCAATCTTGATGTCCGCGACATAATGCGGCACGAAGGCCAGAAAGACAGTATAGAA
TTATACCTTTCCAGGCTTGAGCGGGGCAACAAAGTTGTCCCAAATTTCCAAAAGGAAGCTTTTGACAGATACG
CTGAAACGCCAGACGAAGTTGTCTGTCACAGTACCTTCCAAACGTGTACGCACCAGCAGGTGGAAAACACAG
GCAGGGTGTATGCTATTGCATTGCACAGTATATACGATATACCTGCTGATGAATTCGGAGCGGCACTTTTAAG
GAAAAATGTCCATGTTTGTTACGCCGCCTTCCACTTTTCCGAGAATTTACTTCTCGAAGATTCACACGTCAACCT
TGACGAAATCAACGCGTGTTTTTCGCGTGATGGAGACAAGCTGACTTTTTCTTTCGCATCTGAGAGCACTTTAA
ATTATTGTCATAGTTATTCTAATATTTTAAAATACGTGTGCAGAACTTACTTCCCGGCATCTAATAGAGAGGTCT
ACATGAAGGAGTTTTTGGTCACCAGGGTTAACACCTGGTTTTGTAAGTTTTCTAGGATAGATACTTTTTTATTAT
ACAAGGGGGTAGCCCACAAAGGTGTAGATAGTGAGCAATTTTACAGCGCAATGGAAGATGCATGGCACTACA
AAAAGACTCTTGCAATGTGTAACAGCGAGAGGATTATTCTTGAAGATTCCTCATCGGTCAATTACTGGTTCCCA

AAAATGAGAGATATGGTCATAGTTCCTCTATTCGACATATCTCTCGACACCAGTAAAAGGACCCGCAAAGAAG
TCTTAGTGTCAAAGGATTTTGTATTCACAGTTTTAAATCACATTCGCACTTATCAAGCCAAGGCACTTACATACT
CCAATGTTTTATCCTTTGTCGAATCAATTCGTTCAAGGGTAATTATCAACGGAGTGACTGCCAGGTCTGAGTGG
GATGTTGACAAATCTCTTTTGCAATCCTTGTCCATGACATTTTTCTTGCATACTAAGCTTGCCGTTTTAAAAGAC
GAATTGTTAATCAGCAAGTTTAGTTTGGGGCCAAAATCAGTAAGCCAGCATGTATGGGATGAGATTTCCCTGG
CTTTTGGAAACGCATTTCCATCGATCAAGGAGAGACTGCTAAATCGGAAACTAATTAAAGTGTCGGGAGACGC
ATTAGAAATCAGGGTGCCTGATTTATATGTGACTTTTCACGATAGATTAGTGACTGAGTACAAAACATCGGTG
GATATGCCAGTGCTTGATATCAGAAAGAGAATGGAGGAGACTGAGGTTATGTACAATGCATTGTCTGAGCTA
TCTGTGCTCAAGGAGTCGGACAAGTTCGACGTTGATGTTTTTTCCCGGATGTGCCAGACTTTGGAGGTAGACC
CAATGACTGCAGCAAAGGTTATTGTGGCAGTGATGAGCAACGAGAGCGGACTGACTCTTACATTCGAACAGC
CAACTGAAGCAAATGTCGCATTGGCACTTAAAGATTCAGAAAAAGCCTCTGAGGGTGCACTAGTGGTTACTTC
TAGAGATGTTGAAGAACCGTCCATGAAGGGTTCAATGGCAAGAGGAGAGTTACAATTGGCCGGTCTGTCTGG
AGACCAACCAGAGTCTTCCTATACTCGGAACGAGGAAATAGAGTCATTAGAGCAATTCCACATGGCAACGGCT
AGTTCGTTAATTCGGAAACAGATGAGTTCGATTGTGTACACGGGCCCCATTAAAGTTCAGCAAATGAAAAACT
TTATTGATAGCCTGGTAGCATCACTCTCTGCTGCGGTGTCGAACCTAGTCAAGATCCTAAAGGATACAGCTGCT
ATAGATCTCGAAACCCGTCAGAAGTTTGGAGTCTTAGATGTTGCGACCAAAAGATGGTTAATTAAACCTTTAG
CCAAGAATCACGCATGGGGCGTTATTGAAACACATGCTAGGAAGTACCACGTTGCACTTTTGGAGTATGATGA
GCATGGAGTGGTAACTTGCGACAGTTGGAGAAGGGTGGCCGTGAGTTCTGAGTCAATGGTTTATTCTGATAT
GGCAAAGCTCAGAACACTGAGGAGATTATTAAGGGATGGTGAGCCTCATGTCAGCAGTGCTAAAGTCGTCCT
AGTTGACGGTGTCCCGGGTTGTGGAAAGACAAAAGAGATTCTCTCAAAAGTAAATTTTGAGGAAGATCTAAT
CTTAGTACCGGGTAAGCAGGCTGCTGAAATGATAAAGAGGCGTGCTAATGCGTCAGGAATAATTCAAGCCAC
AAGAGATAATGTTCGTACTGTTGATTCATTTATAATGAATTACGGTAAAGGAACACGCTGTCAGTTCAAAAGG
TTATTTATCGACGAAGGTCTGATGTTGCACACTGGTTGTGTGAATTTTCTTGTTTCTATGTCTCTGTGCGAAATT
GCATATGTTTATGGAGACACACAACAAATTCCATACATCAACAGAGTATCCGGTTTTCCGTACCCTGCACATTT
TGCAAAAATAGAGGTTGATGAGGTGGAAACTCGCAGAACTACGCTGCGTTGTCCAGCCGACATTACCCACTAT
CTTAACAGAAGGTACGAAGGACATGTCATGTGTACATCGTCGGTTAAAAAGTCAGTTTCTCAGGAAATGGTGA
GCGGGGCCGCAATGATCAATCCTGTATCTAAGCCACTGAATGGGAAAGTTTTGACTTTCACTCAGTCTGATAA
AGAGGCGCTGCTTTCTCGAGGATATACGGACGTCCATACAGTACATGAGGTACAAGGTGAGACATATGCAGA
TGTGTCGTTGGTCAGATTGACTCCGACACCTGTATCTATCATCGCAGGAGATAGTCCGCACGTTCTCGTAGCTT
TGTCAAGGCATACCCAAACATTGAAGTATTACACCGTAGTGATGGATCCTCTTGTAAGTATAATTAGGGATTTA
GAAAAACTTAGTTCTTACTTGTTAGATATGTATAAAGTAGATGCAGGGACCCAATAGCAATTACAGGTAGACT
CCGTGTTTAAAGGTTCTAATCTTTTTGTTGCAGCACCAAAGACTGGAGATATCTCAGATATGCAATTTTACTAT
GATAAGTGTCTCCCAGGTAATAGCACCATGTTAAATAACTATGATGCTGTTACCATGAGGTTGACTGACATTTC
TCTTAATGTCAAAGATTGCATATTGGATTTCTCTAAGTCTGTGGCTGCACCGAAGGATCCGATCAAACCACTGA
TTCCAATGGTACGAACGGCGGCAGAAATGCCACGCCAGACTGGACTATTGGAAAATTTGGTGGCGATGATCA
AAAGAAACTTTAATTCACCGGAGTTATCAGGAATAATCGACATTGAGAATACTGCATCTTTAGTAGTAGATAA
ATTTTTTGATAGTTACTTGCTTAAAGAAAAAAGAAAACCAAATAAAAATGTTTCTTTATTTAGTAGAGAGTCTC
TCAATAGATGGTTAGAGAAGCAGGAGCAAGTGACCATTGGTCAGCTTGCAGATTTTGATTTTGTGGATCTTCC
TGCCGTTGATCAGTACAGGCATATGATTAAAGCACAACCTAAGCAGAAGTTGGATACATCAATTCAAAGCGAA
TATCCGGCCTTGCAGACGATTGTGTATCATTCGAAAAAGATCAACGCAATCTTCGGTCCTTTGTTCAGTGAGCT
CACAAGGCAAATGCTCGAAAGCATAGACTCAAGTAAGTTTTTGTTCTTTACAAGGAAGACGCCAGCTCAAATT
GAGGATTTCTTCGGAGATCTCGATAGCCATGTCCCTATGGATATCTTGGAGTTGGATATTTCGAAGTATGACA
AATCTCAGAACGAGTTCCACTGTGCAGTAGAGTATGAAATATGGAGAAGACTTGGATTAGAAGATTTTCTGG
GAGAAGTTTGGAAACAAGGCCACAGGAAAACTACTCTTAAAGATTACACAGCTGGTATTAAAACGTGTTTATG
GTACCAGAGAAAGAGTGGGGACGTTACAACATTCATCGGTAATACGGTGATTATTGCTGCTTGTTTAGCTTCC
ATGTTGCCCATGGAGAAAATAATCAAAGGTGCATTTTGCGGAGATGACAGTTTACTATACTTCCCAAAAGGTT
GTGAGTTTCCTGACATACAGCACACAGCCAACCTTATGTGGAATTTCGAGGCTAAGCTATTCAGAAAGCAGTA
TGGTTATTTCTGTGGAAGGTACGTGATACATCATGACAGAGGGTGTATTGTTTATTATGACCCTTTGAAGTTGA

TTTCTAAACTTGGTGCTAAACACATCAAGGATTGGGATCACTTAGAAGAGTTCAGAAGATCCCTTTGTGATGTT
GCAAATTCGTTGAACAACTGTGCGTATTACACGCAGTTGGACGACGCTGTGAGTGAGGTCCATAAAACCGCAC
CCCCGGGTTCGTTTGTGTATAAAAGTTTAGTTAAATATCTGTCCGATAAGGTTCTTTTTAGAAGTTTGTTTATAG
ATGGCTCTTGTTAAGGGTAAAGTCAATATTAATGAGTTCATAGACTTGTCAAAATCAGAAAAATTTCTTCCGTC
TATGTTCACACCTGTTAAGAGTGTCATGATCTCCAAGGTTGATAAGATATTGGTTCATGAAGATGAATCTTTGT
CCGAAGTCAATTTACTCAAAGGTGTAAAACTCATTGATGGTGGCTATGTACATCTTGCTGGTCTTGTGGTGACA
GGTGAATGGAATTTGCCAGATAATTGTCGTGGTGGTGTCAGTGTCTGTTTGGTCGATAAGAGAATGGAGAGA
GCGGACGAGGCAACTCTTGCTTCATACTATACCGCAGCGGCTAAGAAAAGGTTTCAGTTCAAAGTCGTTCCAA
ATTACAACATCACTACCAAGGACGCAGAAAAGGCAGTTTGGCAAGTACTAGTTAATATTAGAAATGTTAAAAT
TGCTGCGGGTTACTGTCCGCTGTCATTAGAATTTGTGTCAGTGTGTATTGTTTATAAAAATATTATAAAACTCG
GTTTGAGAGAGAAAATTACGAGCGTCACGGATGGAGGGCCCATGGAACTATCAGAAGAAGTTGTTGATGAG
TTCATTGAAGAAGTCCCGATGTCTGTAAGGCTTGCAAAATTTCGTTCGAAGACCGGAAAAAAGTTTAGTAGTA
AAAGTGAGAATAATAGTGGTAATAATAGGCCGAAACCAAACAAAAACCAAAGGAAGGAAAAGGGTTTAAAA
GTTAGGGTTGAGAAGGATAATTTAATTGATAATGAATTGGAGACTTACATCGCCGATTCAGATTCGTATTAAA
TATGTCTTACACAATCGCAACTCCATCGCAATTTGTGTTTTTGTCATCAGCATGGGCCGACCCTATAGAATTAAT
AAATTTATGTACTAATTCACTAGGTAATCAGTTCCAAACACAACAAGCTAGAACAACCGTTCAACGGCAATTTA
GCGAAGTGTGGAAACCTGTCCCTCAAGTCACTGTTAGGTTTCCTGACAGTGGTTTTAAGGTGTATAGGTACAA
TGCGGTACTAGATCCTCTAGTTACTGCTTTGTTAGGAGCTTTCGATACTAGAAATAGGATTATAGAAGTCGAAA
ATCAGGCGAACCCGACAACCGCCGAAACGTTAGACGCTACTCGTAGAGTAGATGACGCAACGGTGGCTATAA
GGAGCGCTATAAATAATTTAGTAGTAGAATTGGTCAAAGGAACAGGTTTGTACAATCAGAGCACATTTGAAA
GTGCATCCGGTTTACAATGGTCCTCTGCACCTGCATCTTGA

#OM892671

1

ATGGCATACACACAGACAGCTACCACATCCGCTTTGCTCGACACTGTCCGAGGTAACAATACCTTGGTCAACG
ATCTTGCGAAGCGGCGTCTTTATGACACAGCGGTCGACGAGTTCAACGCTCGTGATCGCAGGCCCAAAGTAA
ATTTTTCCAAAGTAATAAGTGAGGAACAGACGCTTATTGCTACTAGGGCATATCCAGAATTCCAGATAACCTTC
TATAATACGCAGAACGCCGTGCATTCGCTTGCCGGTGGACTACGATCCTTAGAACTGGAATATCTAATGATGC
AGATCCCGTACGGATCACTCACATATGATATAGGTGGGAATTTTGCATCTCATCTGTTCAAAGGACGGGCATA
TGTTCACTGCTGTATGCCCAATCTTGATGTCCGCGACATAATGCGGCACGAAGGCCAGAAAGACAGTATAGAA
TTATACCTTTCCAGGCTTGAGCGGGGCAACAAAGTTGTCCCAAATTTCCAAAAGGAAGCTTTTGACAGATACG
CTGAAACGCCAGACGAAGTTGTCTGTCACAGTACCTTCCAAACGTGTACGCACCAGCAGGTGGAAAACACAG
GCAGGGTGTATGCTATTGCATTGCACAGTATATACGATATACCTGCTGATGAATTCGGAGCGGCACTTTTAAG
GAAAAATGTCCATGTTTGTTACGCCGCCTTCCACTTTTCCGAGAATTTACTTCTCGAAGATTCACACGTCAACCT
TGACGAAATCAACGCGTGTTTTTCGCGTGATGGAGACAAGCTGACTTTTTCTTTCGCATCTGAGAGCACTTTAA
ATTATTGTCATAGTTATTCTAATATTTTAAAATACGTGTGCAAAACTTACTTCCCGGCATCTAATAGAGAGGTCT
ACATGAAGGAGTTTTTGGTCACCAGGGTTAACACCTGGTTTTGTAAGTTTTCTAGGATAGATACTTTTTTATTAT
ACAAGGGGGTAGCCCACAAAGGTGTAAATAGTGAGCAATTTTACAGCGCAATGGAAGATGCATGGCACTACA
AAAAGACTCTTGCAATGTGTAACAGCGAGAGGATTCTTCTTGAAGATTCCTCATCGGTCAATTACTGGTTCCCA
AAAATGAGAGATATGGTCATAGTTCCTCTATTCGACATATCTCTCGACACCAGTAAAAGGACCCGCAAAGAAG
TCTTAGTGTCAAAGGATTTTGTATTCACAGTTTTAAATCACATTCGCACTTATCAAGCCAAGGCACTTACATACT
CCAATGTTTTATCCTTTGTCGAATCAATTCGTTCAAGGGTAATTATCAACGGAGTGACTGCCAGGTCTGAGTGG
GATGTTGACAAATCTCTTTTGCAATCCTTGTCCATGACATTTTTCTTGCATACTAAGCTTGCCGTTTTAAAAGAC
GAATTGTTAATCAGCAAGTTTAGTTTGGGGCCAAAATCAGTAAGCCAGCATGTATGGGATGAGATTTCCCTGG

CTTTTGGAAACGCATTTCCATCGATCAAGGAGAGACTGCTAAATCGGAAACTAATTAAAGTGTCGGGAGACGC
ATTAGAAATCAGGGTGCCTGATTTATATGTGACTTTTCACGATAGATTAGTGACTGAGTACAAAACATCGGTG
GATATGCCAGTGCTTGATATCAGAAAGAGAATGGAGGAGACTGAGGTTATGTACAATGCATTGTCTGAGCTA
TCTGTGCTCAAGGAGTCGGACAAGTTCGACGTTGATGTTTTTTCCCGGATGTGCCAGACTTTGGAGGTAGACC
CAATGACTGCAGCAAAGGTTATTGTGGCAGTGATGAGCAACGAGAGCGGACTGACTCTTACATTCGAACAGC
CAACTGAAGCAAATGTCGCATTGGCACTTAAAGATTCAGAAAAAGCCTCTGAGGGTGCACTAGTGGTTACTTC
TAGAGATGTTGAAGAACCATCCATGAAGGGTTCAATGGCAAGAGGAGAGTTACAATTGGCCGGTCTGTCTGG
AGACCAACCAGAGTCTTCCTATACTCGGAACGAGGAAATAGAGTCATTAGAGCAATTCCACATGGCAACGGCT
AGTTCGTTAATTCGGAAACAGATGAGTTCGATTGTGTACACGGGCCCCATTAAAGTTCAGCAAATGAAAAACT
TTATTGATAGCCTGGTAGCATCACTCTCTGCTGCGGTGTCGAACCTAGTCAAGATCCTAAAGGATACAGCTGCT
ATAGATCTCGAAACCCGTCAGAAGTTTGGAGTCTTAGATGTTGCGACCAAAAGATGGTTAATTAAACCTTTAG
CCAAGAATCACGCATGGGGCGTTATTGAAACACATGCTAGGAAGTACCACGTTGCACTTTTGGAGTATGATGA
GCATGGAGTGGTAACTTGCGACAGTTGGAGAAGGGTGGCCGTGAGTTCTGAGTCAATGGTTTATTCTGATAT
GGCAAAGCTCAGAACACTGAGGAGATTATTAAGGGATGGTGAGCCTCATGTCAGCAGTGCTAAAGTCGTCCT
AGTTGACGGTGTCCCGGGTTGTGGAAAGACAAAAGAGATTCTCTCGAAAGTAAATTTTGAGGAAGATCTAAT
CTTAGTACCGGGTAAGCAGGCTGCTGAAATGATAAAGAGGCGTGCTAATGCGTCAGGAATAATTCAAGCCAC
AAGAGATAATGTTCGTACTGTTGATTCATTTATAATGAATTACGGTAAAGGAACACGCTGTCAGTTCAAAAGG
TTATTTATCGACGAAGGTCTGATGTTGCACACTGGTTGTGTGAATTTTCTTGTTTCTATGTCTCTGTGCGAAATT
GCATATGTTTATGGAGACACACAACAAATTCCATACATCAACAGAGTATCCGGTTTTCCGTACCCTGCACATTT
TGCAAAAATAGAGGTTGATGAGGTGGAAACTCGCAGAACTACGCTGCGTTGTCCAGCCGACATTACCCACTAT
CTTAACAGAAGGTACGAAGGATATGTCATGTGTACATCGTCGGTTAAAAAGTCAGTTTCTCAGGAAATGGTGA
GCGGGGCCGCAATGATCAATCCTGTATCTAAGCCACTGAATGGGAAAGTTTTGACTTTCACTCAGTCTGATAA
AGAGGCGCTGCTTTCTCGAGGATATACGGACGTCCATACAGTACATGAGGTACAAGGTGAGACATATGCAGA
TGTGTCGTTGGTCAGATTGACTCCGACACCTGTATCTATCATCGCAGGAGATAGTCCGCACGTTCTCGTAGCTT
TGTCAAGGCATACCCAAACATTGAAGTATTACACCGTAGTGATGGATCCTCTTGTAAGTATAATTAGGGATTTA
GAAAAACTTAGTTCTTACTTGTTAGATATGTATAAAGTAGATGCAGGGACCCAATAGCAATTACAGGTAGACT
CCGTGTTTAAAGGTTCTAATCTTTTTGTTGCAGCACCAAAGACTGGAGATATCTCAGATATGCAATTTTACTAT
GATAAGTGTCTCCCAGGTAATAGCACCATGTTAAATAACTATGATGCTGTTACCATGAGGTTGACTGACATTTC
TCTTAATGTCAAAGATTGCATATTGGATTTCTCTAAGTCTGTGGCTGCACCGAAGGATCCGATCAAACCACTGA
TTCCAATGGTACGAACGGCGGCAGAAATGCCACGCCAGACTGGACTATTGGAAAATTTGGTGGCGATGATCA
AAAGAAACTTTAATTCACCGGAGTTATCGGGAATAATCGACATTGAGAATACTGCATCTTTAGTAGTAGATAA
ATTTTTTGATAGTTACTTGCTTAAAGAAAAAAGAAAACCAAATAAAAATGTTTCTTTATTTTGTAGAGAGTCTCT
CAATAGATGGTTAGAGAAGCAGGAGCAAGTGACCATTGGTCAGCTTGCAGATTTTGATTTTGTGGATCTTCCT
GCCGTTGATCAGTACAGGCATATGATTAAAGCGCAACCTAAGCAGAAGCTGGATACATCAATTCAAAGCGAAT
ATCCGGCCTTGCAGACGATTGTGTATCATTCGAAAAAGATCAACGCAATCTTCGGTCCTTTGTTCAGTGAGCTC
ACAAGGCAAATGCTCGAAAGCATAGACTCAAGTAAGTTTTTGTTCTTTACAAGGAAGACGCCAGCTCAAATTG
AGGATTTCTTCGGAGATCTCGATAGCCATGTCCCTATGGATATCTTGGAGTTGGATATTTCGAAGTATGACAAA
TCTCAGAACGAGTTCCACTGTGCAGTAGAGTATGAAATATGGAGAAGACTTGGATTAGAAGATTTTCTGGGA
GAAGTTTGGAAACAAGGCCACAGGAAAACTACTCTTAAAGATTACACAGCTGGTATTAAAACGTGTTTATGGT
ACCAGAGAAAGAGTGGGGACGTTACAACATTCATCGGTAATACGGTGATTATTGCTGCTTGTTTAGCTTCCAT
GTTGCCCATGGAGAAAATAATCAAAGGTGCATTTTGCGGAGATGACAGTTTACTATACTTCCCAAAAGGTTGT
GAGTTTCCTGACATACAGCATACAGCCAACCTTATGTGGAATTTCGAGGCTAAGCTATTCAGAAAGCAGTATG
GTTATTTCTGTGGAAGGTACGTGATACATCATGACAGAGGGTGTATTGTTTATTATGACCCTTTGAAGTTGATT
TCTAAACTTGGTGCTAAACACATCAAGGATTGGGATCACTTAGAAGAGTTCAGAAGATCCCTTTGTGATGTTG
CAAATTCGTTGAACAACTGTGCGTATTACACGCAGTTGGACGACGCTGTGAGTGAGGTCCATAAAACCGCACC
CCCGGGTTCGTTTGTGTATAAAAGTTTAGTTAAATATCTGTCCGATAAGGTTCTTTTTAGAAGTTTGTTTATAGA
TGGCTCTTGTTAAGGGTAAAGTCAATATTAATGAGTTCATAGACTTGTCAAAATCAGAAAAATTTCTTCCGTCT
ATGTTCACACCTGTTAAGAGTGTCATGATCTCCAAGGTTGATAAGATATTGGTTCATGAAGATGAATCTTTGTC

CGAAGTCAATTTACTCAAAGGTGTAAAACTCATTGATGGTGGCTATGTACATCTTGCTGGTCTTGTGGTGACA
GGTGAATGGAATTTGCCAGATAATTGTCGTGGTGGTGTCAGTGTCTGTTTGGTCGATAAGAGAATGGAGAGA
GCGGACGAGGCAACTCTTGCTTCATACTATACCGCAGCGGCTAAGAAAAGGTTTCAGTTCAAAGTCGTTCCAA
ATTACAACATCACTACCAAGGACGCAGAAAAGGCAGTTTGGCAAGTACTAGTTAATATTAGAAATGTTAAAAT
TGCTGCGGGTTACTGTCCGCTGTCATTAGAATTTGTGTCAGTGTGTATTGTTTATAAAAATATTATAAAACTCG
GTTTGAGAGAGAAAATTACGAGCGTCACGGATGGAGGGCCCATGGAACTATCAGAAGAAGTTGTTGATGAG
TTCATGGAAGAAGTCCCGATGTCTGTAAGGCTTGCAAAATTTCGTTCGAAGACCGGAAAAAAGTTTAGTAGTA
AAAGTGAGAATAATAGTGGTAATAATAGGCCGAAACCAAACAAAAACCAAAGGAAGGAAAAGGGTTTAAAA
GTTAGGGTTGAGAAGGATAATTTAATTGATAATGAATTGGAGACTTACATCGCCGATTCAGATTCGTATTAAA
TATGTCTTACACAATCGCAACTCCATCGCAATTTGTGTTTTTGTCATCAGCATGGGCCGACCCTATAGAATTAAT
AAATTTATGTACTAATTCACTAGGTAATCAGTTCCAAACACAACAAGCTAGAACAACCGTTCAACGGCAATTTA
GCGAAGTGTGGAAACCTGTCCCTCAAGTCACTGTTAGGTTTCCTGACAGTGGTTTTAAGGTGTATAGGTACAA
TGCGGTACTAGATCCTCTAGTTACTGCTTTGTTAGGAGCTTTCGATACTAGAAATAGGATTATAGAAGTCGAAA
ATCAGGCGAACCCGACAACCGCCGAAACGTTAGACGCTACTCGTAGAGTAGATGACGCAACGGTGGCTATAA
GGAGCGCTATAAATAATTTAGTAGTAGAATTGGTCAAAGGAACAGGTTTGTACAATCAGAGCACATTTGAAA
GTGCATCCGGTTTACAATGGTCCTCTGCACCTGCATCTTGA

#OM892672

1

ATGGCATACACACAGACAGCTACCACATCCGCTTTGCTCGACACTGTCCGAGGTAACAATACCTTGGTCAACG
ATCTTGCGAAGCGGCGTCTTTATGACACAGCGGTCGACGAGTTCAACGCTCGTGATCGCAGGCCCAAAGTAA
ATTTTTCCAAAGTAATAAGTGAGGAACAGACGCTTATTGCTACTAGGGCATATCCAGAATTCCAGATAACCTTC
TATAATACGCAGAACGCCGTGCATTCGCTTGCCGGTGGACTACGATCCTTAGAACTGGAATATCTAATGATGC
AGATCCCGTACGGATCACTCACATATGATATAGGTGGGAATTTTGCATCTCATCTGTTCAAAGGACGGGCATA
TGTTCACTGCTGTATGCCCAATCTTGATGTCCGCGACATAATGCGGCACGAAGGCCAGAAAGACAGTATAGAA
TTATACCTTTCCAGGCTTGAGCGGGGCAACAAAGTTGTCCCAAATTTCCAAAAGGAAGCTTTTGACAGATACG
CTGAAACGCCAGACGAAGTTGTCTGTCACAGTACCTTCCAAACGTGTACGCACCAGCAGGTGGAAAACACAG
GCAGGGTGTATGCTATTGCATTGCACAGTATATACGATATACCTGCTGATGAATTCGGAGCGGCACTTTTAAG
GAAAAATGTCCATGTTTGTTACGCCGCCTTCCACTTTTCCGAGAATTTACTTCTCGAAGATTCACACGTCAACCT
TGACGAAATCAACGCGTGTTTTTCGCGTGATGGAGACAAGCTGACTTTTTCTTTCGCATCTGAGAGCACTTTAA
ATTATTGTCATAGTTATTCTAATATTTTAAAATACGTGTGCAAAACTTACTTCCCGGCATCTAATAGAGAGGTCT
ACATGAAGGAGTTTTTGGTCACCAGGGTTAACACCTGGTTTTGTAAGTTTTCTAGGATAGATACTTTTTTATTAT
ACAAGGGGGTAGCCCACAAAGGTGTAAATAGTGAGCAATTTTACAGCGCAATGGAAGATGCATGGCACTACA
AAAAGACTCTTGCAATGTGTAACAGCGAGAGGATTCTTCTTGAAGATTCCTCATCGGTCAATTACTGGTTCCCA
AAAATGAGAGATATGGTCATAGTTCCTCTATTCGACATATCTCTCGACACCAGTAAAAGGACCCGCAAAGAAG
TCTTAGTGTCAAAGGATTTTGTATTCACAGTTTTAAATCACATTCGCACTTATCAAGCCAAGGCACTTACATACT
CCAATGTTTTATCCTTTGTCGAATCAATTCGTTCAAGGGTAATTATCAACGGAGTGACTGCCAGGTCTGAGTGG
GATGTTGACAAATCTCTTTTGCAATCCTTGTCCATGACATTTTTCTTGCATACTAAGCTTGCCGTTTTAAAAGAC
GAATTGTTAATCAGCAAGTTTAGTTTGGGGCCAAAATCAGTAAGCCAGCATGTATGGGATGAGATTTCACTGG
CTTTTGGAAACGCATTTCCATCGATCAAGGAGAGACTGCTAAATCGGAAACTAATTAAAGTGTCGGGAGACGC
ATTAGAAATCAGGGTGCCTGATTTATATGTGACTTTTCACGATAGATTAGTGACTGAGTACAAAACATCGGTG
GATATGCCAGTGCTTGATATCAGAAAGAGAATGGAGGAGACTGAGGTTATGTACAATGCATTGTCTGAGCTA
TCTGTGCTCAAGGAGTCGGACAAGTTCGACGTTGATGTTTTTTCCCGGATGTGCCAGACTTTGGAGGTAGACC
CAATGACTGCAGCAAAGGTTATTGTGGCAGTGATGAGCAACGAGAGCGGACTGACTCTTACATTCGAACAGC

CAACTGAAGCAAATGTCGCATTGGCACTTAAAGATTCAGAAAAAGCCTCTGAGGGTGCACTAGTGGTTACTTC
TAGAGATGTTGAAGAACCATCCATGAAGGGTTCAATGGCAAGAGGAGAGTTACAATTGGCCGGTCTGTCTGG
AGACCAACCAGAGTCTTCCTATACTCGGAACGAGGAAATAGAGTCATTAGAGCAATTCCACATGGCAACGGCT
AGTTCGTTAATTCGGAAACAGATGAGTTCGATTGTGTACACGGGCCCCATTAAAGTTCAGCAAATGAAAAACT
TTATTGATAGCCTGGTAGCATCACTCTCTGCTGCGGTGTCGAACCTAGTCAAGATCCTAAAGGATACAGCTGCT
ATAGATCTCGAAACCCGTCAGAAGTTTGGAGTCTTAGATGTTGCGACCAAAAGATGGTTAATTAAACCTTTAG
CCAAGAATCACGCATGGGGCGTTATTGAAACACATGCTAGGAAGTACCACGTTGCACTTTTGGAGTATGATGA
GCATGGAGTGGTAACTTGCGACAGTTGGAGAAGGGTGGCCGTGAGTTCTGAGTCAATGGTTTATTCTGATAT
GGCAAAGCTCAGAACACTGAGGAGATTATTAAGGGATGGTGAGCCTCATGTCAGCAGTGCTAAAGTCGTCCT
AGTTGACGGTGTCCCGGGTTGTGGAAAGACAAAAGAGATTCTCTCGAAAGTAAATTTTGAGGAAGATCTAAT
CTTAGTACCGGGTAAGCAGGCTGCTGAAATGATAAAGAGGCGTGCTAATGCGTCAGGAATAATTCAAGCCAC
AAGAGATAATGTTCGTACTGTTGATTCATTTATAATGAATTACGGTAAAGGAACACGCTGTCAGTTCAAAAGG
TTATTTATCGACGAAGGTCTGATGTTGCACACTGGTTGTGTGAATTTTCTTGTTTCTATGTCTCTGTGCGAAATT
GCATATGTTTATGGAGACACACAACAAATTCCATACATCAACAGAGTATCCGGTTTTCCGTACCCTGCACATTT
TGCAAAAATAGAGGTTGATGAGGTGGAAACTCGCAGAACTACGCTGCGTTGTCCAGCCGACATTACCCACTAT
CTTAACAGAAGGTACGAAGGACATGTCATGTGTACATCGTCGGTTAAAAAGTCAGTTTCTCAGGAAATGGTGA
GCGGGGCCGCAATGATCAATCCTGTATCTAAGCCACTGAATGGGAAAGTTTTGACTTTCACTCAGTCTGATAA
AGAGGCGCTGCTTTCTCGAGGATATACGGACGTCCATACAGTACATGAGGTACAAGGTGAGACATATGCAGA
TGTGTCGTTGGTCAGATTGACTCCGACACCTGTATCTATCATCGCAGGAGATAGTCCGCACGTTCTCGTAGCTT
TGTCAAGGCATACCCAAACATTGAAGTATTACACCGTAGTGATGGATCCTCTTGTAAGTATAATTAGGGATTTA
GAAAAACTTAGTTCTTACTTGTTAGATATGTATAAAGTAGATGCAGGGACCCAATAGCAATTACAGGTAGACT
CCGTGTTTAAAGGTTCTAATCTTTTTGTTGCAGCACCAAAGACTGGAGATATCTCAGATATGCAATTTTACTAT
GATAAGTGTCTCCCAGGTAATAGCACCATGTTAAATAACTATGATGCTGTTACCATGAGGTTGACTGACATTTC
TCTTAATGTCAAAGATTGCATATTGGATTTCTCTAAGTCTGTGGCTGCACCGAAGGATCCGATCAAACCACTGA
TTCCAATGGTACGAACGGCGGCAGAAATGCCACGCCAGACTGGACTATTGGAAAATTTGGTGGCGATGATAA
AAAGAAACTTTAATTCACCGGAGTTATCAGGAATAATCGACATTGAGAATACTGCATCTTTAGTAGTAGATAA
ATTTTTTGATAGTTACTTGCTTAAAGAAAAAAGAAAACCAAATAAAAATGTTTCTTTATTTTGTAGAGAGTCTCT
CAATAGATGGTTAGAGAAGCAGGAGCAAGTGACCATTGGTCAGCTTGCAGATTTTGATTTTGTGGATCTTCCT
GCCGTTGATCAGTACAGGCATATGATTAAAGCGCAACCTAAGCAGAAGCTGGATACATCAATTCAAAGCGAAT
ATCCGGCCTTGCAGACGATTGTGTATCATTCGAAAAAGATCAACGCAATCTTCGGTCCTTTGTTCAGTGAGCTC
ACAAGGCAAATGCTCGAAAGCATAGACTCAAGTAAGTTTTTGTTCTTTACAAGGAAGACGCCAGCTCAAATTG
AGGATTTCTTCGGAGATCTCGATAGCCATGTCCCTATGGATATCTTGGAGTTGGATATTTCGAAGTATGACAAA
TCTCAAAACGAGTTCCACTGTGCAGTAGAGTATGAAATATGGAGAAGACTTGGATTAGAAGATTTTCTGGGA
GAAGTTTGGAAACAAGGCCACAGAAAAACTACTCTTAAAGATTACACAGCTGGTATTAAAACGTGTTTATGGT
ACCAGAGAAAGAGTGGGGACGTTACAACATTCATCGGTAATACGGTGATTATTGCTGCTTGTTTAGCTTCCAT
GTTGCCCATGGAAAAAATAATCAAAGGTGCATTTTGCGGAGATGACAGTTTACTATACTTCCCAAAAGGTTGT
GAGTTTCCTGACATACAGCATACAGCCAACCTTATGTGGAATTTCGAGGCTAAGCTATTCAGAAAGCAGTATG
GTTATTTCTGTGGAAGGTACGTGATACATCATGACAGAGGGTGTATTGTTTATTATGACCCTTTGAAGTTGATT
TCTAAACTTGGTGCTAAACACATCAAGGATTGGGATCACTTAGAAGAGTTCAGAAGATCCCTTTGTGATGTTG
CAAATTCGTTGAACAACTGTGCGTATTACACGCAGTTGGACGACGCTGTGAGTGAGGTCCATAAAACCGCACC
CCCGGGTTCGTTTGTGTATAAAAGTTTAGTTAAATATCTGTCCGATAAGGTTCTTTTTAGAAGTTTGTTTATAGA
TGGCTCTTGTTAAGGGTAAAGTCAATATTAATGAGTTCATAGACTTGTCAAAATCAGAAAAAATTTCTTCCGTCT
ATGTTCACACCTGTTAAGAGTGTCATGATCTCCAAGGTTGATAAGATATTGGTTCATGAAGATGAATCTTTGTC
CGAAGTCAATTTACTCAAAGGTGTAAAACTCATTGATGGTGGCTATGTACATCTTGCTGGTCTTGTGGTGACA
GGTGAATGGAATTTGCCAGATAATTGTCGTGGTGGTGTCAGTGTCTGTTTGGTCGATAAGAGAATGGAGAGA
GCGGACGAGGCAACTCTTGCTTCATACTATACCGCAGCGGCTAAGAAAAGGTTTCAGTTCAAAGTCGTTCCAA
ATTACAACATCACTACCAAGGACGCAGAAAAGGCAGTTTGGCAAGTACTAGTTAATATTAGAAATGTTAAAAT
TGCTGCGGGTTACTGTCCGCTGTCATTAGAATTTGTGTCAGTGTGTATTGTTTATAAAAATATTATAAAACTCG

GTTTGAGAGAGAGAAAATTACGAGCGTCACGGATGGAGGGCCCATGGAACTATCAGAAGAAGTTGTTGATGAG
TTCATGGAAGAAGTCCCGATGTCTGTAAGGCTTGCAAAATTTCGTTCGAAGACCGGAAAAAAGTTTAGTAGTA
AAAGTGAGAATAATAGTGGTAATAATAGGCCGAAACCAAACAAAAACCAAAGGAAGGAAAAGGGTTTAAAA
GTTAGGGTTGAGAAGGATAATTTAATTGATAATGAATTGGAGACTTACATCGCCGATTCAGATTCGTATTAAA
TATGTCTTACACAATCGCAACTCCATCGCAATTTGTGTTTTTGTCATCAGCATGGGCCGACCCTATAGAATTAAT
AAATTTATGTACTAATTCACTAGGTAATCAGTTCCAAACACAACAAGCTAGAACAACCGTTCAACGGCAATTTA
GCGAAGTGTGGAAACCTGTCCCTCAAGTCACTGTTAGGTTTCCTGACAGTGGTTTTAAGGTGTATAGGTACAA
TGCGGTACTAGATCCTCTAGTTACTGCTTTGTTAGGAGCTTTCGATACTAGAAATAGGATTATAGAAGTCGAAA
ATCAGGCGAACCCGACAACCGCCGAAACGTTAGACGCTACTCGTAGAGTAGATGACGCAACGGTGGCTATAA
GGAGCGCTATAAATAATTTAGTAGTAGAATTGGTCAAAGGAACAGGTTTGTACAATCAGAGCACATTTGAAA
GTGCATCCGGTTTACAATGGTCCTCTGCACCTGCATCTTGA

#OM892674

1

ATGGCATACACACAGACAGCTACCACATCCGCTTTGCTCGACACTGTCCGAGGTAACAATACCTTGGTCAATG
ATCTTGCGAAGCGGCGTCTTTATGACACAGCGGTCGACGAGTTCAACGCTCGTGATCGCAGGCCCAAAGTAA
ATTTTTCCAAAGTAATAAGTGAGGAACAGACGCTTATTGCTACTAGGGCATATCCAGAATTCCAGATAACCTTC
TATAATACGCAGAACGCCGTGCATTCGCTTGCCGGTGGACTACGATCCTTAGAACTGGAATATCTAATGATGC
AGATCCCGTACGGATCACTCACATATGATATAGGTGGGAATTTTGCATCTCATCTGTTCAAAGGACGGGCATA
TGTTCACTGCTGTATGCCCAATCTTGATGTCCGCGACATAATGCGGCACGAAGGCCAGAAAGACAGTATAGAA
TTATACCTTTCCAGGCTTGAGCGGGGCAACAAAGTTGTCCCAAATTTCCAAAAGGAAGCTTTTGACAGATACG
CTGAAACGCCAGACGAAGTTGTCTGTCACAGTACCTTCCAAACGTGTACGCACCAGCAGGTGGAAAACACAG
GCAGGGTGTATGCTATTGCATTGCACAGTATATACGATATACCTGCTGATGAATTCGGAGCGGCACTTTTAAG
GAAAAATGTCCATGTTTGTTACGCCGCCTTCCACTTTTCCGAGAATTTACTTCTCGAAGATTCACACGTCAACCT
TGACGAAATCAACGCGTGTTTTTCGCGTGATGGAGACAAGCTGACTTTTTCTTTCGCATCTGAGAGCACTTTAA
ATTATTGTCATAGTTATTCTAATATTTTAAAATACGTGTGCAAAACTTACTTCCCGGCATCTAATAGAGAGGTCT
ACATGAAGGAGTTTTTAGTCACCAGGGTTAACACCTGGTTTTGTAAGTTTTCTAGGATAGATACTTTTTTATTAT
ACAAGGGGGTAGCCCACAAAGGTGTAAATAGTGAGCAATTTTACAGCGCAATGGAAGATGCATGGCACTACA
AAAAGACTCTTGCAATGTGTAACAGCGAGAGGATTCTTCTTGAAGATTCCTCATCGGTCAATTACTGGTTCCCA
AAAATGAGAGATATGGTCATAGTTCCTCTATTCGACATATCTCTCGACACCAGTAAAAGGACCCGCAAAGAAG
TCTTAGTGTCAAAGGATTTTGTATTCACAGTTTTAAATCACATTCGCACTTATCAAGCCAAGGCACTTACATACT
CCAATGTTTTATCCTTTGTCGAATCAATTCGTTCAAGGGTAATTATCAACGGAGTGACTGCCAGGTCTGAGTGG
GATGTTGACAAATCTCTTTTGCAATCCTTGTCCATGACATTTTTCTTGCATACTAAGCTTGCCGTTTTAAAAGAC
GAATTGTTAATCAGCAAGTTTAGTTTGGGGCCAAAATCAGTAAGCCAGCATGTATGGGATGAGATTTCCCTGG
CTTTTGGAAACGCATTTCCATCGATCAAGGAGAGACTGCTAAATCGGAAACTAATTAAAGTGTCGGGAGACGC
ATTAGAAATCAGGGTGCCTGATTTATATGTGACTTTTCACGATAGATTAGTGACTGAGTACAAAACATCGGTG
GATATGTCAGTGCTTGATATCAGAAAGAGAATGGAGGAGACTGAGGTTATGTACAATGCATTGTCTGAGCTA
TCTGTGCTCAAGGAGTCGGACAAGTTCGACGTTGATGTTTTTTCCCGGATGTGCCAGACTTTGGAGGTAGACC
CAATGACTGCAGCAAAGGTTATAGTGGCAGTGATGAGCAACGAGAGCGGACTGACTCTTACATTCGAACAGC
CAACTGAAGCAAATGTCGCATTGGCACTTAAAGATTCAGAAAAAGCCTCTGAGGGTGCACTAGTGGTTACTTC
TAGAGATGTTGAAGAACCATCCATGAAGGGTTCAATGGCAAGAGGAGAGTTACAATTGGCCGGTCTGTCTGG
AGACCAACCAGAGTCTTCCTATACTCGGAACGAGGAAATAGATTCATTAGAGCAATTCCACATGGCAACGGCT
AGTTCGTTAATTCGGAAACAGATGAGTTCGATTGTGTACACGGGCCCCATTAAAGTTCAGCAAATGAAAAACT
TTATTGATAGCCTGGTAGCATCACTCTCTGCTGCGGTGTCGAACCTAGTCAAGATCCTAAAGGATACAGCTGCT

ATAGATCTCGAAACCCGTCAGAAGTTTGGAGTCTTAGATGTTGCGACCAAAAGATGGTTAATTAAACCTTTAG
CCAAGAATCACGCATGGGGCGTTATTGAAACACATGCTAGGAAGTACCACGTTGCACTTTTGGAGTATGATGA
GCATGGAGTGGTAACTTGCGACAGTTGGAGAAGGGTGGCCGTGAGTTCTGAGTCAATGGTTTATTCTGATAT
GGCAAAGCTCAGAACACTGAGGAGATTATTAAGAGATGGTGAGCCTCATGTCAGCAGTGCTAAAGTCGTCCT
AGTTGACGGTGTCCCGGGTTGTGGAAAGACAAAAGAGATTCTCTCGAAAGTAAATTTTGAGGAAGATCTAAT
CTTAGTACCGGGTAAGCAGGCTGCTGAAATGATAAAGAGGCGTGCTAATGCGTCAGGAATAATTCAAGCCAC
AAGAGATAATGTTCGTACTGTTGATTCATTTATAATGAACTACGGTAAAGGAACACGCTGTCAGTTCAAAAGG
TTATTTATCGACGAAGGTCTGATGTTGCACACTGGTTGTGTGAATTTTCTTGTTTCTATGTCTCTGTGCGAAATT
GCATATGTTTATGGAGACACACAACAAATTCCATACATCAACAGAGTATCCGGTTTTCCGTACCCTGCACATTT
TGCAAAAATAGAGGTTGATGAGGTGGAAACTCGCAGAACTACGCTGCGTTGTCCAGCCGACATTACCCACTAT
CTTAACAGAAGGTACGAAGGACATGTCATGTGTACATCGTCGGTTAAAAAGTCAGTTTCTCAGGAAATGGTGA
GCGGGGCCGCAATGATCAATCCTGTATCTAAGCCATTGAATGGGAAAGTTTTGACTTTCACTCAGTCTGATAA
AGAGGCGCTGCTTTCTCGAGGATATACGGACGTCCATACAGTACATGAGGTACAAGGTGAGACATATGCAGA
TGTGTCGTTGGTCAGATTGACTCCGACACCTGTATCTATCATCGCAGGAGATAGTCCGCACGTTCTCGTAGCTT
TGTCAAGGCATACCCAAACATTGAAGTATTACACCGTAGTGATGGATCCTCTTGTAAGTATAATTAGGGATTTA
GAAAAACTTAGTTCTTACTTGTTAGATATGTATAAAGTAGATGCAGGGACCCAATAGCAATTACAGGTAGACT
CCGTGTTTAAAGGTTCTAATCTTTTTGTTGCAGCACCAAAGACTGGAGATATCTCAGATATGCAATTTTACTAT
GATAAGTGTCTCCCAGGTAATAGCACCATGTTAAATAACTATGATGCTGTTACCATGAGGTTGACTGACATTTC
TCTTAATGTCAAAGATTGCATATTGGATTTCTCTAAGTCTGTGGCTGCACCGAAGGATCCGATCAAACCACTGA
TTCCAATGGTACGAACGGCGGCAGAAATGCCACGCCAGACTGGACTATTGGAAAATTTGGTGGCGATGATCA
AAAGAAACTTTAATTCACCGGAGTTATCAGGAATAATCGACATTGAGAATACTGCATCTTTAGTAGTAGATAA
ATTTTTTGATAGTTACTTGCTTAAAGAAAAAAGAAAACCAAATAAAAATGTTTCTTTATTTTGTAGAGAGTCTCT
CAATAGATGGTTAGAGAAGCAGGAGCAAGTGACCATTGGTCAGCTTGCGGATTTTGATTTTGTGGATCTTCCT
GCCGTTGATCAGTACAGGCATATGATTAAAGCGCAACCTAAGCAGAAGCTGGATACATCAATTCAAAGCGAAT
ATCCGGCCTTGCAGACGATTGTGTATCATTCGAAAAAGATCAACGCAATCTTCGGTCCTTTGTTCAGTGAGCTC
ACAAGGCAAATGCTCGAAAGCATAGACTCAAGTAAGTTTTTGTTCTTTACAAGGAAGACGCCAGCTCAAATTG
AGGATTTCTTCGGAGATCTCGATAGCCATGTCCCTATGGATATCTTGGAGTTGGATATTTCGAAGTATGACAAA
TCTCAGAACGAGTTCCACTGTGCAGTAGAGTATGAAATATGGAGAAGACTTGGATTAGAAGATTTTCTGGGA
GAAGTTTGGAAACAAGGCCACAGAAAAACTACTCTTAAAGATTACACAGCTGGTATTAAAACGTGTTTATGGT
ACCAGAGAAAGAGTGGAGACGTTACAACATTCATCGGTAATACGGTGATTATTGCTGCATGTTTAGCTTCCAT
GTTGCCCATGGAGAAAATAATCAAAGGTGCATTTTGCGGAGATGACAGTTTACTATACTTCCCAAAAGGTTGT
GAGTTTCCTGACATACAGCATACAGCCAACCTTATGTGGAATTTCGAGGCTAAGCTATTCAGAAAGCAGTATG
GTTATTTCTGTGGAAGGTACGTGATACATCATGACAGAGGGTGTATTGTTTATTATGACCCTTTGAAGTTGATT
TCTAAACTTGGTGCTAAACACATCAAGGATTGGGATCACTTAGAAGAGTTCAGAAGATCCCTTTGTGATGTTG
CAATTTCGTTGAACAACTGTGCGTATTACACGCAGTTGGACGACGCTGTGAGTGAGGTCCATAAAACCGCACC
CCCGGGTTCGTTTGTTTATAAAAGTTTAGTTAAATATCTGTCCGATAAGGTTCTTTTTAGAAGTTTGTTTATAGA
TGGCTCTTGTTAAGGGTAAAGTCAATATTAATGAGTTCATAGACTTGTCAAAATCAGAAAAATTTCTTCCGTCT
ATGTTCACACCTGTTAAGAGTGTCATGATCTCCAAGGTTGATAAGATATTGGTTCATGAAGATGAATCTTTGTC
CGAAGTCAATTTACTCAAAGGTGTAAAACTCATTGATGGTGGCTATGTACATCTTGCTGGTCTTGTGGTGACA
GGTGAATGGAATTTGCCAGATAATTGTCGTGGTGGTGTCAGTGTCTGTTTGGTCGATAAGAGAATGGAGAGA
GCGGACGAGGCAACTCTTGCTTCATACTATACCGCAGCGGCTAAGAAAAGGTTTCAGTTCAAAGTCGTTCCAA
ATTACAACATCACTACCAAGGACGCAGAAAAGGCAGTTTGGCAAGTACTAGTTAATATTAGAAATGTTAAAAT
TGCTGCGGGTTACTGTCCGCTGTCATTAGAATTTGTGTCAGTGTGTATTGTTTATAAAAATATTATAAAACTCG
GTTTGAGAGAGAAAATTACGAGCGTCACGGATGGAGGGCCCATGGAACTATCAGAAGAAGTTGTTGATGAG
TTCATGGAAGAAGTCCCGATGTCTGTAAGGCTTGCAAAATTTCGTTCGAAGACCGGAAAAAAGTTTAGTAGTA
AAAGTGAGAATAATAGTGGTAATAATAGGCCGAAGCCAGGCAAAAACCAAAGGAAGGAAAAGGGTTTAAAA
GTTAGGGTTGAGAAAGATAATTTAATTGATAATGAATTGGAGACTTACGTCGCCGATTCAGATTCGTATTAAA
TATGTCTTACACAATCGCAACTCCATCGCAATTTGTGTTTTTGTCATCAGCATGGGCCGACCCTATAGAATTAAT

AAATTTATGTACTAATTCACTAGGTAATCAGTTCCAAACACAACAAGCTAGAACAACCGTTCAACGGCAATTTA
GCGAAGTGTGGAAACCTGTCCCTCAAGTCACTGTTAGGTTTCCTGACAGTGGTTTTAAGGTGTATAGGTACAA
TGCGGTACTAGATCCTCTAGTTACTGCTTTGTTAGGAGCTTTCGATACTAGAAATAGGATTATAGAAGTCGAAA
ATCAGGCGAACCCGACAACCGCCGAAACGTTAGACGCTACTCGTAGAGTAGATGACGCAACGGTGGCTATAA
GGAGCGCTATAAATAATTTAGTAGTAGAATTGGTCAAAGGAACAGGTTTGTACAATCAGAGCACATTTGAAA
GTGCATCCGGTTTACAATGGTCCCCTGCACCTGCATCTTGA

#OM892675

1

ATGGCATACACACAGACAGCTACCACATCCGCTTTGCTCGACACTGTCCGAGGTAACAATACCTTGGTCAACG
ATCTTGCGAAGCGGCGTCTTTATGACACAGCGGTCGACGAGTTCAACGCTCGTGATCGCAGGCCCAAAGTAA
ATTTTTCCAAAGTAATAAGTGAGGAACAGACGCTTATTGCTACTAGGGCATATCCAGAATTCCAGATAACCTTC
TATAATACGCAGAACGCCGTGCATTCGCTTGCCGGTGGACTACGATCCTTAGAACTGGAATATCTAATGATGC
AGATCCCGTACGGATCACTCACATATGATATAGGTGGGAATTTTGCATCTCATCTGTTCAAAGGACGGGCATA
TGTTCACTGCTGTATGCCCAATCTTGATGTCCGCGACATAATGCGGCACGAAGGCCAGAAAGACAGTATAGAA
TTATACCTTTCCAGGCTTGAACGGGGCAACAAAGTTGTCCCAAATTTCCAAAAGGAAGCTTTTGACAGATACG
CTGAAACGCCAGACGAAGTTGTCTGTCACAGTACCTTCCAAACGTGTACGCACCAGCAGGTGGAAAACACAG
GCAGGGTGTATGCTATTGCATTGCACAGTATATACGATATACCTGCTGATGAATTCGGAGCGGCACTTTTAAG
GAAAAATGTCCATGTTTGTTACGCCGCCTTCCACTTTTCCGAGAATTTACTTCTCGAAGATTCACACGTCAACCT
TGACGAAATCAACGCGTGTTTTTCGCGTGATGGAGACAAGCTGACTTTTTCTTTCGCATCTGAGAGCACTTTAA
ATTATTGTCATAGTTATTCTAATATTTTAAAATACGTGTGCAAAACTTACTTCCCGGCATCTAATAGAGAGGTCT
ACATGAAGGAGTTTTTGGTCACCAGGGTTAACACCTGGTTTTGTAAGTTTTCTAGAATAGATACTTTTTTATTAT
ACAAGGGGGTAGCCCACAAAGGTGTAAATAGTGAGCAATTTTACAGCGCAATGGAAGATGCATGGCACTACA
AAAAGACTCTTGCAATGTGTAACAGCGAGAGGATTCTTCTTGAAGATTCCTCATCGGTCAATTACTGGTTCCCA
AAAATGAGAGATATGGTCATAGTTCCTCTATTCGACATATCTCTCGACACCAGTAAAAGGACCCGCAAAGAAG
TCTTAGTGTCAAAGGATTTTGTATTCACAGTTTTAAATCACATTCGCACTTATCAAGCCAAGGCACTTACATACT
CCAATGTTTTATCCTTTGTCGAATCAATTCGTTCAAGGGTAATTATCAACGGAGTGACTGCCAGGTCTGAGTGG
GATGTTGACAAATCTCTTTTGCAATCCTTGTCCATGACATTTTTCTTGCATACTAAGCTTGCCGTTTTAAAAGAC
GAATTGTTAATCAGCAAGTTTAGTTTGGGGCCAAAATCAGTAAGCCAGCATGTATGGGATGAGATTTCCCTGG
CTTTTGGAAACGCATTTCCATCGATCAAGGAGAGACTGCTAAATCGGAAACTAATTAAAGTGTCGGGAGACGC
ATTAGAAATCAGGGTGCCTGATTTATATGTGACTTTTCACGATAGATTAGTGACTGAGTACAAAACATCGGTG
GATATGCCAGTGCTTGATATCAGAAAGAGAATGGAGGAGACTGAGGTTATGTACAATGCATTGTCTGAGCTA
TCTGTGCTCAAGGAGTCGGACAAGTTCGACGTTGATGTTTTTTCCCGGATGTGCCAGACTTTGGAGGTAGACC
CAATGACTGCAGCAAAGGTTATTGTGGCAGTGATGAGCAACGAGAGCGGACTGACTCTTACATTCGAACAGC
CAACTGAAGCAAATGTCGCATTGGCACTTAAAGATTCAGAAAAAGCCTCTGAGGGTGCACTAGTGGTTACTTC
TAGAGATGTTGAAGAACCATCCATGAAGGGTTCAATGGCAAGAGGAGAGTTACAATTGGCCGGTCTGTCTGG
AGACCAACCAGAGTCTTCCTATACTCGGAACGAGGAAATAGAGTCATTAGAGCAATTCCACATGGCAACGGCT
AGTTCGTTAATTCGGAAACAGATGAGTTCGATTGTGTACACGGGCCCCATTAAAGTTCAGCAAATGAAAAACT
TTATTGATAGCCTGGTAGCATCACTCTCTGCTGCGGTGTCGAACCTAGTCAAGATCCTAAAGGATACAGCTGCT
ATAGATCTCGAAACCCGTCAGAAGTTTGGAGTCTTAGATGTTGCGACCAAAAGATGGTTAATTAAACCTTTAG
CCAAGAATCACGCATGGGGCGTTATTGAAACACATGCTAGGAAGTACCACGTTGCACTTTTGGAGTATGATGA
GCATGGAGTGGTAACTTGCGACAGTTGGAGAAGGGTGGCCGTGAGTTCTGAGTCAATGGTTTATTCTGATAT
GGCAAAGCTCAGAACACTGAGGAGATTATTAAGGGATGGTGAGCCTCATGTCAGCAGTGCTAAAGTCGTCCT
AGTTGACGGTGTCCCGGGTTGTGGAAAGACAAAAGAGATTCTCTCGAAAGTAAATTTTGAGGAAGATCTAAT

CTTAGTACCGGGTAAGCAAGCTGCTGAAATGATAAAGAGGCGTGCTAATGCGTCAGGAATAATTCAAGCCAC
AAGAGATAATGTTCGTACTGTTGATTCATTTATAATGAATTACGGTAAAGGAACACGCTGTCAGTTCAAAAGG
TTATTTATCGACGAAGGTCTGATGTTGCACACTGGTTGTGTGAATTTTCTTGTTTCTATGTCTCTGTGCGAAATT
GCATATGTTTATGGAGACACACAACAAATTCCATACATCAACAGAGTATCCGGTTTTCCGTACCCTGCACATTT
TGCAAAAATAGAGGTTGATGAGGTGGAAACTCGCAGAACTACGCTGCGTTGTCCAGCCGACATTACCCACTAT
CTTAACAGAAGGTACGAAGGACATGTCATGTGTACATCGTCGGTTAAAAAGTCAGTTTCTCAGGAAATGGTGA
GCGGGGCCGCAATGATCAATCCTGTATCTAAGCCACTGAATGGGAAAGTTTTGACTTTCACTCAGTCTGATAA
AGAGGCGCTGCTTTCTCGAGGATATACGGACGTCCATACAGTACATGAGGTACAAGGTGAGACATATGCAGA
TGTGTCGTTGGTCAGATTGACTCCGACACCTGTATCTATCATCGCAGGAGATAGTCCGCACGTTCTCGTAGCTT
TGTCAAGGCATACCCAAACATTGAAGTATTACACCGTAGTGATGGATCCTCTTGTAAGTATAATTAGGGATTTA
GAAAAACTTAGTTCTTACTTGTTAGATATGTATAAAGTAGATGCAGGGACCCAATAGCAATTACAGGTAGACT
CCGTGTTTAAAGGTTCTAATCTTTTTGTTGCAGCACCAAAGACTGGAGATATCTCAGATATGCAATTTTACTAT
GATAAGTGTCTCCCAGGTAATAGCACCATGTTAAATAACTATGATGCTGTTACCATGAGGTTGACTGACATTTC
TCTTAATGTCAAAGATTGCATATTGGATTTCTCTAAGTCTGTGGCTGCACCGAAGGATCCGATCAAACCACTGA
TTCCAATGGTACGAACGGCGGCAGAAATGCCACGCCAGACTGGACTATTGGAAAATTTGGTGGCGATGATCA
AAAGAAACTTTAATTCACCGGAGTTATCAGGAATAATCGACATTGAGAATACTGCATCTTTAGTAGTAGATAA
ATTTTTTGATAGTTACTTGCTTAAAGAAAAAAGAAAACCAAATAAAAATGTTTCTTTATTTTGTAGAGAGTCTCT
CAATAGATGGTTAGAGAAGCAGGAGCAAGTGACCATTGGTCAGCTTGCAGATTTTGATTTTGTGGATCTTCCT
GCCGTTGATCAGTACAGGCATATGATTAAAGCGCAACCTAAGCAGAAGCTGGATACATCAATTCAAAGCGAAT
ATCCGGCCTTGCAGACGATTGTGTATCATTCGAAAAAGATCAACGCAATCTTCGGTCCTTTGTTCAGTGAGCTC
ACAAGGCAAATGCTCGAAAGCATAGACTCAAGTAAGTTTTTGTTCTTTACAAGGAAGACGCCAGCTCAAATTG
AGGATTTCTTCGGAGATCTCGATAGCCATGTCCCTATGGATATCTTGGAGTTGGATATTTCGAAGTATGACAAA
TCTCAAAACGAGTTCCACTGTGCAGTAGAGTATGAAATATGGAGAAGACTTGGATTAGAAGATTTTCTGGGA
GAAGTTTGGAAACAAGGCCACAGAAAAACTACTCTTAAAGATTACACAGCTGGTATTAAAACGTGTTTATGGT
ACCAGAGAAAGAGTGGGGACGTTACAACATTCATCGGTAATACGGTGATTATTGCTGCTTGTTTAGCTTCCAT
GTTGCCCATGGAGAAAATAATCAAAGGTGCATTTTGCGGAGATGACAGTTTACTATACTTCCCAAAAGGTTGT
GAGTTTCCTGACATACAGCATACAGCCAACCTTATGTGGAATTTCGAGGCTAAGCTATTCAGAAAGCAGTATG
GTTATTTCTGTGGAAGGTACGTGATACATCATGACAGAGGGTGTATTGTTTATTATGACCCTTTGAAGTTGATT
TCTAAACTTGGTGCTAAACACATCAAGGATTGGGATCACTTAGAAGAGTTCAGAAGATCCCTTTGTGATGTTG
CAAATTCGTTGAACAACTGTGCGTATTACACGCAGTTGGACGACGCTGTGAGTGAGGTCCATAAAACCGCACC
CCCGGGTTCGTTTGTGTATAAAAGTTTAGTTAAATATCTGTCCGATAAGGTTCTTTTTAGAAGTTTGTTTATAGA
TGGCTCTTGTTAAGGGTAAAGTCAATATTAATGAGTTCATAGACTTGTCAAAATCAGAAAAATTTCTTCCGTCT
ATGTTCACACCTGTTAAGAGTGTCATGATCTCCAAGGTTGATAAGATATTGGTTCATGAAGATGAATCTTTGTC
CGAAGTCAATTTACTCAAAGGTGTAAAACTCATTGATGGTGGCTATGTACATCTTGCTGGTCTCGTGGTGACA
GGTGAATGGAATTTGCCAGATAATTGTCGTGGTGGTGTCAGTGTCTGTTTGGTCGATAAGAGAATGGAGAGA
GCGGACGAGGCAACTCTTGCTTCATACTATACCGCAGCGGCTAAGAAAAGGTTTCAGTTCAAAGTCGTTCCAA
ATTACAACATCACTACCAAGGACGCAGAAAAGGCAGTTTGGCAAGTACTAGTTAATATTAGAAATGTTAAAAT
TGCTGCGGGTTACTGTCCGCTGTCATTAGAATTTGTGTCAGTGTGTATTGTTTATAAAAATATTATAAAACTCG
GTTTGAGAGAGAAAATTACGAGCGTCACGGATGGAGGGCCCATGGAACTATCAGAAGAAGTTGTTGATGAG
TTCATGGAAGAAGTCCCGATGTCTGTAAGGCTTGCAAAATTTCGTTCGAAGACCGGAAAAAAGTTTAGTAGTA
AAAGTGAGAATAATAGTGGTAATAATAGGCCGAAACCAAACAAAAACCAAAGGAAGGAAAAGGGTTTAAAA
GTTAGGGTTGAGAAGGATAATTTAATTGATAATGAATTGGAGACTTACATCGCCGATTCAGATTCGTATTAAA
TATGTCTTACACAATCGCAACTCCATCGCAATTTGTGTTTTTGTCATCAGCATGGGCCGACCCTATAGAATTAAT
AAATTTATGTACTAATTCACTAGGTAATCAGTTCCAAACACAACAAGCTAGAACAACCGTTCAACGGCAATTTA
GCGAAGTGTGGAAACCTGTCCCTCAAGTCACTGTTAGGTTTCCTGACAGTGGTTTTAAGGTGTATAGGTACAA
TGCGGTACTAGATCCTCTAGTTACTGCTTTGTTAGGAGCTTTCGATACTAGAAATAGGATTATAGAAGTCGAAA
ATCAGGCGAACCCGACAACCGCCGAAACGTTAGACGCTACTCGTAGAGTAGATGACGCAACGGTGGCTATAA

GGAGCGCTATAAATAATTTAGTAGTAGAATTGGTCAAAGGAACAGGTTTGTACAATCAGAGCACATTTGAAA
GTGCATCCGGTTTACAATGGTCCTCTGCACCTGCATCTTGA

#OM892676

1

ATGGCATACACACAGACAGCTACCACATCCGCTTTGCTCGACACTGTCCGAGGTAACAATACCTTGGTCAATG
ATCTTGCGAAGCGGCGTCTTTATGACACAGCGGTCGACGAGTTCAACGCTCGTGATCGCAGGCCCAAAGTAA
ATTTTTCCAAAGTAATAAGTGAGGAACAGACGCTTATTGCTACTAGGGCATATCCAGAATTCCAGATAACCTTC
TATAATACGCAGAACGCCGTGCATTCGCTTGCCGGTGGACTACGATCCTTAGAACTGGAATATCTAATGATGC
AGATCCCGTACGGATCACTCACATATGATATAGGTGGGAATTTTGCATCTCATCTGTTCAAAGGACGGGCATA
TGTTCACTGCTGTATGCCTAATCTTGATGTCCGCGACATAATGCGGCACGAAGGCCAGAAAGACAGTATAGAA
TTATACCTTTCCAGGCTTGAGCGGGGCAACAAAGTTGTCCCAAATTTCCAAAAGGAAGCTTTTGACAGATACG
CTGAAACGCCAGACGAAGTTGTCTGTCACAGTACCTTCCAAACGTGTACGCACCAGCAGGTGGAAAACACAG
GCAGGGTGTATGCTATTGCATTGCACAGTATATACGATATACCTGCTGATGAATTCGGAGCGGCACTTTTAAG
GAAAAATGTCCATGTTTGTTACGCCGCCTTCCACTTTTCCGAGAATTTACTTCTCGAAGATTCACACGTCAACCT
TGACGAAATCAACGCGTGTTTTTCGCGTGATGGAGACAAGCTGACTTTTTCTTTCGCATCTGAGAGCACTTTAA
ATTATTGTCATAGTTATTCTAATATTTTAAAATACGTGTGCAAAACTTACTTCCCGGCATCTAATAGAGAGGTCT
ACATGAAGGAGTTTTTAGTCACCAGGGTTAACACCTGGTTTTGTAAGTTTTCTAGGATAGATACTTTTTTATTAT
ACAAGGGGGTAGCCCACAAAGGTGTAAATAGTGAGCAATTTTACAGCGCAATGGAAGATGCATGGCACTACA
AAAAGACTCTTGCAATGTGTAACAGCGAGAGGATTCTTCTTGAAGATTCCTCATCGGTCAATTACTGGTTCCCA
AAAATGAGAGATATGGTCATAGTTCCTCTATTCGACATATCTCTCGACACCAGTAAAAGGACCCGCAAAGAAG
TCTTAGTGTCAAAGGATTTTGTATTCACAGTTTTAAATCACATTCGCACTTATCAAGCCAAGGCACTTACATACT
CCAATGTTTTATCCTTTGTCGAATCAATTCGTTCAAGGGTAATTATCAACGGAGTGACTGCCAGGTCTGAGTGG
GATGTTGACAAATCTCTTTTGCAATCCTTGTCCATGACATTTTTCTTGCATACTAAGCTTGCCGTTTTAAAAGAC
GAATTGTTAATCAGCAAGTTTAGTTTGGGGCCAAAATCAGTAAGCCAGCATGTATGGGATGAGATTTCCCTGG
CTTTTGGAAACGCATTTCCATCGATCAAGGAGAGACTGCTAAATCGGAAACTAATTAAAGTGTCGGGAGACGC
ATTAGAAATCAGGGTGCCTGATTTATATGTGACTTTTCACGATAGATTAGTGACTGAGTACAAAACATCGGTG
GATATGCCAGTGCTTGATATCAGAAAGAGAATGGAGGAGACTGAGGTTATGTACAATGCATTGTCTGAGCTA
TCTGTGCTCAAGGAGTCGGACAAGTTCGACGTTGATGTTTTTTCCCGGATGTGCCAGACTTTGGAGGTAGACC
CAATGACTGCAGCAAAGGTTATAGTGGCAGTGATGAGCAACGAGAGCGGACTGACTCTTACATTCGAACAGC
CAACTGAAGCAAATGTCGCATTGGCACTAAAAGATTCAGAAAAAGCCTCTGAGGGTGCACTAGTGGTTACTTC
TAGAGATGTTGAAGAACCATCCATGAAGGGTTCAATGGCAAGAGGAGAGTTACAATTGGCCGGTCTGTCTGG
AGACCAACCAGAGTCTTCCTATACTCGGAACGAGGAAATAGAGTCATTAGAGCAATTCCACATGGCAACGGCT
AGTTCGTTAATTCGGAAACAGATGAGTTCGATTGTGTACACGGGCCCCATTAAAGTTCAGCAAATGAAAAACT
TTATTGATAGCCTGGTAGCATCACTCTCTGCTGCGGTGTCGAACCTAGTCAAGATCCTAAAGGATACAGCTGCT
ATAGATCTCGAAACCCGTCAGAAGTTTGGAGTCTTAGATGTTGCGACCAAAAGATGGTTAATTAAACCTTTAG
CCAAAAATCACGCATGGGGCGTTATTGAAACACATGCTAGGAAGTACCACGTTGCACTTTTGGAGTATGATGA
GCATGGAGTGGTAACTTGCGACAGTTGGAGAAGGGTGGCCGTGAGTTCTGAGTCAATGGTTTATTCTGATAT
GGCAAAGCTCAGAACACTGAGGAGATTATTAAGAGATGGTGAGCCTCATGTCAGCAGTGCTAAAGTCGTCCT
AGTTGACGGTGTCCCGGGTTGTGGAAAGACAAAAGAGATTCTCTCGAAAGTAAATTTTGAGGAAGATCTAAT
CTTAGTACCGGGTAAGCAGGCTGCTGAAATGATAAAGAGGCGTGCTAATGCGTCAGGAATAATTCAAGCCAC
AAGAGATAATGTTCGTACTGTTGATTCATTTATAATGAATTACGGTAAAGGAACACGCTGTCAGTTCAAAAGG
TTATTTATCGACGAAGGTCTGATGTTGCACACTGGTTGTGTGAATTTTCTTGTTTCTATGTCTCTGTGCGAAATT
GCATATGTATATGGAGACACACAACAAATTCCATACATCAACAGAGTATCCGGTTTTCCGTACCCTGCACATTT

TGCAAAAATAGAGGTTGATGAGGTGGAAACACGCAGAACTACGCTGCGTTGTCCAGCCGACATTACCCACTA
TCTTAACAGAAGGTACGAAGGACATGTCATGTGTACATCGTCGGTTAAAAAGTCAGTTTCTCAGGAAATGGTG
AGCGGGGCCGCAATGATCAATCCTGTATCTAAGCCATTGAATGGGAAAGTTTTGACTTTCACTCAGTCTGATA
AAGAGGCGCTGCTTTCTCGAGGATATACGGACGTCCATACAGTACATGAGGTACAAGGTGAGACATATGCAG
ATGTGTCGTTGGTCAGATTGACTCCGACACCTGTATCTATCATCGCAGGAGATAGTCCGCACGTTCTCGTAGCT
TTGTCAAGGCATACCCAAACATTGAAGTATTACACTGTAGTGATGGATCCTCTTGTAAGTATAATTAGGGATTT
AGAAAAACTTAGTTCTTACTTGTTAGATATGTATAAAGTAGATGCAGGGACCCAATAGCAATTACAGGTAGAC
TCCGTGTTTAAAGGTTCTAATCTTTTTGTTGCAGCACCAAAGACTGGAGATATCTCAGATATGCAATTTTACTAT
GATAAGTGTCTCCCAGGTAATAGCACCATGTTAAATAACTATGATGCTGTTACCATGAGGTTGACTGACATTTC
TCTTAATGTCAAAGATTGCATATTGGATTTCTCTAAGTCTGTGGCTGCACCGAAGGATCCGATCAAACCACTGA
TTCCAATGGTACGAACGGCGGCAGAAATGCCACGCCAGACTGGACTATTGGAAAATTTGGTGGCGATGATCA
AAAGAAACTTTAATTCACCGGAGTTATCAGGAATAATCGACATTGAGAATACTGCATCTTTAGTAGTAGATAA
ATTTTTTGATAGTTACTTGCTTAAAGAAAAAAGAAAACCAAATAAAAATGTTTCTTTATTTTGTAGAGAGTCTCT
CAATAGATGGTTAGAGAAGCAGGAGCAAGTGACCATTGGTCAGCTTGCAGATTTTGATTTTGTGGATCTTCCT
GCCGTTGATCAGTACAGGCATATGATTAAAGCGCAACCTAAGCAGAAGCTGGATACATCAATTCAAAGCGAAT
ATCCGGCCTTGCAGACGATTGTGTATCATTCGAAAAAGATCAACGCAATCTTCGGTCCTTTGTTCAGTGAGCTC
ACAAGGCAAATGCTCGAAAGCATAGACTCAAGTAAGTTTTTGTTCTTTACAAGGAAGACGCCAGCTCAAATTG
AGGATTTCTTCGGAGATCTCGATAGCCATGTCCCTATGGATATCTTGGAGTTGGATATTTCAAAGTATGACAAA
TCTCAGAACGAGTTCCACTGTGCAGTAGAGTATGAAATATGGAGAAGACTTGGATTAGAAGATTTTCTGGGA
GAAGTTTGGAAACAAGGCCACAGAAAAACTACTCTTAAAGATTACACAGCTGGTATTAAAACGTGTTTATGGT
ACCAGAGAAAGAGTGGGGACGTTACAACATTCATCGGTAATACGGTGATTATTGCTGCTTGTTTAGCTTCCAT
GTTGCCCATGGAGAAAATAATCAAAGGTGCATTTTGCGGAGATGACAGTTTACTATACTTCCCAAAAGGTTGT
GAGTTTCCTGACATACAGCATACAGCTAACCTTATGTGGAATTTCGAGGCTAAGCTATTCAGAAAGCAGTATG
GTTATTTCTGTGGAAGGTACGTGATACATCATGACAGAGGGTGTATTGTTTATTATGACCCTTTGAAGTTGATT
TCTAAACTTGGTGCTAAACACATCAAGGATTGGGATCACTTAGAAGAGTTCAGAAGATCCCTTTGTGATGTTG
CAATTTCGTTGAACAACTGTGCGTATTACACGCAGTTGGACGACGCTGTGAGTGAGGTCCATAAAACCGCACC
CCCGGGTTCGTTTGTTTATAAAAGTTTAGTTAAATATCTGTCCGATAAGGTTCTTTTTAGAAGTTTGTTTATAGA
TGGCTCTTGTTAAGGGTAAAGTCAATATTAATGAGTTCATAGACTTGTCAAAATCAGAAAAATTTCTTCCGTCT
ATGTTCACACCTGTTAAGAGTGTCATGATCTCCAAGGTTGATAAGATATTGGTTCATGAAGATGAATCTTTGTC
CGAAGTCAATTTACTCAAAGGTGTAAAACTCATTGATGGTGGCTATGTACATCTTGCTGGTCTTGTAGTGACAG
GTGAATGGAATTTGCCAGATAATTGTCGTGGTGGTGTCAGTGTCTGTTTGGTCGATAAGAGAATGGAGAGAG
CGGACGAGGCAACTCTTGCTTCATACTATACCGCAGCGGCTAAGAAAAGGTTTCAGTTCAAAGTCGTACCAAA
TTACAACATCACTACCAAGGACGCAGAAAAGGCAGTTTGGCAAGTACTAGTTAATATTAGGAATGTTAAAATT
GCTGCGGGTTACTGTCCGCTGTCATTAGAATTTGTGTCAGTGTGTATTGTTTATAAAAATATTATAAAACTCGG
TTTGAGAGAGAAAATTACGAGCGTCACGGATGGAGGGCCCATGGAACTATCAGAAGAAGTTGTTGATGAGTT
CATGGAAGAAGTCCCGATGTCTGTAAGGCTTGCAAAATTTCGTTCGAAGACCGGGAAAAAGTTTAGTAGTAA
AAGTGAGAATAATAGTGGTAATAATAGGCCGAAACCAGACAAAAACCAAAGGAAGGAAAAGGGTTTAAAAG
TTAGGGTTGAGAAGGATAATTTAATTGATAATGAATTGGAGACTTACGTCGCCGATTCAGATTCGTATTAAAT
ATGTCTTACACAATCGCAACTCCATCGCAATTTGTGTTTTTGTCATCAGCATGGGCCGACCCTATAGAATTAATA
AATTTATGTACTAATTCACTAGGTAATCAGTTCCAAACACAACAAGCTAGAACAACCGTTCAACGGCAATTTAG
CGAAGTGTGGAAACCTGTCCCTCAAGTCACTGTTAGGTTTCCTGACAGTGGTTTTAAGGTGTATAGGTACAAT
GCGGTACTAGATCCTCTAGTTACTGCTTTGTTAGGAGCTTTCGATACTAGAAATAGGATTATAGAAGTCGAAA
ATCAGGCGAACCCGACAACCGCCGAAACGTTAGACGCTACTCGTAGAGTAGATGACGCAACGGTGGCTATAA
GGAGCGCTATGAATAATTTAGTAGTAGAATTGGTCAAAGGAACAGGTTTGTACAATCAGAGCACATTTGAAA
GTGCATCCGGTTTACAATGGTCCTCTGCACCTGCATCTTGA

#OM892678

1

ATGGCATACACACAGACAGCTACCACATCCGCTTTGCTCGACACTGTCCGAGGTAACAATACCTTGGTCAATG
ATCTTGCGAAGCGGCGTCTTTATGACACAGCGGTCGACGAGTTCAACGCTCGTGATCGCAGGCCCAAAGTAA
ATTTTTCCAAAGTAATAAGTGAGGAACAGACGCTTATTGCTACTAGGGCATATCCAGAATTCCAGATAACCTTC
TATAATACGCAGAACGCCGTGCATTCGCTTGCCGGTGGACTACGATCCTTAGAACTGGAATATCTAATGATGC
AGATCCCGTACGGATCACTCACATATGATATAGGTGGGAATTTTGCATCTCATCTGTTCAAAGGACGGGCATA
TGTTCACTGCTGTATGCCTAATCTTGATGTCCGCGACATAATGCGGCACGAAGGCCAGAAAGACAGTATAGAA
TTATACCTTTCCAGGCTTGAGCGGGGCAACAAAGTTGTCCCAAATTTCCAAAAGGAAGCTTTTGACAGATACG
CTGAAACGCCAGACGAAGTTGTCTGTCACAGTACCTTCCAAACGTGTACGCACCAGCAGGTGGAAAACACAG
GCAGGGTGTATGCTATTGCATTGCACAGTATATACGATATACCTGCTGATGAATTCGGAGCGGCACTTTTAAG
GAAAAATGTCCATGTTTGTTACGCCGCCTTCCACTTTTCCGAGAATTTACTTCTCGAAGATTCACACGTCAACCT
TGACGAAATCAACGCGTGTTTTTCGCGTGATGGAGACAAGCTGACTTTTTCTTTCGCATCTGAGAGCACTTTAA
ATTATTGTCATAGTTATTCTAATATTTTAAAATACGTGTGCAAAACTTACTTCCCGGCATCTAATAGAGAGGTCT
ACATGAAGGAGTTTTTAGTCACCAGGGTTAACACCTGGTTTTGTAAGTTTTCTAGGATAGATACTTTTTTATTAT
ACAAGGGGGTAGCCCACAAAGGTGTAAATAGTGAGCAATTTTACAGCGCAATGGAAGATGCATGGCACTACA
AAAAGACTCTTGCAATGTGTAACAGCGAGAGGATTCTTCTTGAAGATTCCTCATCGGTCAATTACTGGTTCCCA
AAAATGAGAGATATGGTCATAGTTCCTCTATTCGACATATCTCTCGACACCAGTAAAAGGACCCGCAAAGAAG
TCTTAGTGTCAAAGGATTTTGTATTCACAGTTTTAAATCACATTCGCACTTATCAAGCCAAGGCACTTACATACT
CCAATGTTTTATCCTTTGTCGAATCAATTCGTTCAAGGGTAATTATCAACGGAGTGACTGCCAGGTCTGAGTGG
GATGTTGACAAATCTCTTTTGCAATCCTTGTCCATGACATTTTTCTTGCATACTAAGCTTGCCGTTTTAAAAGAC
GAATTGTTAATCAGCAAGTTTAGTTTGGGGCCAAAATCAGTAAGCCAGCATGTATGGGATGAGATTTCCCTGG
CTTTTGGAAACGCATTTCCATCGATCAAGGAGAGACTGCTAAATCGGAAACTAATTAAAGTGTCGGGAGACGC
ATTAGAAATCAGGGTGCCTGATTTATATGTGACTTTTCACGATAGATTAGTGACTGAGTACAAAACATCGGTG
GATATGCCAGTGCTTGATATCAGAAAGAGAATGGAGGAGACTGAGGTTATGTACAATGCATTGTCTGAGCTA
TCTGTGCTCAAGGAGTCGGACAAGTTCGACGTTGATGTTTTTTCCCGGATGTGCCAGACTTTGGAGGTAGACC
CAATGACTGCAGCAAAGGTTATAGTGGCAGTGATGAGCAACGAGAGCGGACTGACTCTTACATTCGAACAGC
CAACTGAAGCAAATGTCGCATTGGCACTAAAAGATTCAGAAAAAGCCTCTGAGGGTGCACTAGTGGTTACTTC
TAGAGATGTTGAAGAACCATCCATGAAGGGTTCAATGGCAAGAGGAGAGTTACAATTGGCCGGTCTGTCTGG
AGACCAACCAGAGTCTTCCTATACTCGGAACGAGGAAATAGAGTCATTAGAGCAATTCCACATGGCAACGGCT
AGTTCGTTAATTCGGAAACAGATGAGTTCGATTGTGTACACGGGCCCCATTAAAGTTCAGCAAATGAAAAACT
TTATTGATAGCCTGGTAGCATCACTCTCTGCTGCGGTGTCGAACCTAGTCAAGATCCTAAAGGATACAGCTGCT
ATAGATCTCGAAACCCGTCAGAAGTTTGGAGTCTTAGATGTTGCGACCAAAAGATGGTTAATTAAACCTTTAG
CCAAAAATCACGCATGGGGCGTTATTGAAACACATGCTAGGAAGTACCACGTTGCACTTTTGGAGTATGATGA
GCATGGAGTGGTAACTTGCGACAGTTGGAGAAGGGTGGCCGTGAGTTCTGAGTCAATGGTTTATTCTGATAT
GGCAAAGCTCAGAACACTGAGGAGATTATTAAGAGATGGTGAGCCTCATGTCAGCAGTGCTAAAGTCGTCCT
AGTTGACGGTGTCCCGGGTTGTGGAAAGACAAAAGAGATTCTCTCGAAAGTAAATTTTGAGGAAGATCTAAT
CTTAGTACCGGGTAAGCAGGCTGCTGAAATGATAAAGAGGCGTGCTAATGCGTCAGGAATAATTCAAGCCAC
AAGAGATAATGTTCGTACTGTTGATTCATTTATAATGAATTACGGTAAAGGAACACGCTGTCAGTTCAAAAGG
TTATTTATCGACGAAGGTCTGATGTTGCACACTGGTTGTGTGAATTTTCTTGTTTCTATGTCTCTGTGCGAAATT
GCATATGTATATGGAGACACACAACAAATTCCATACATCAACAGAGTATCCGGTTTTCCGTACCCTGCACATTT
TGCAAAAATAGAGGTTGATGAGGTGGAAACACGCAGAACTACGCTGCGTTGTCCAGCCGACATTACCCACTA
TCTTAACAGAAGGTACGAAGGACATGTCATGTGTACATCGTCGGTTAAAAAGTCAGTTTCTCAGGAAATGGTG
AGCGGGGCCGCAATGATCAATCCTGTATCTAAGCCATTGAATGGGAAAGTTTTGACTTTCACTCAGTCTGATA
AAGAGGCGCTGCTTTCTCGAGGATATACGGACGTCCATACAGTACATGAGGTACAAGGTGAGACATATGCAG
ATGTGTCGTTGGTCAGATTGACTCCGACACCTGTATCTATCATCGCAGGAGATAGTCCGCACGTTCTCGTAGCT
TTGTCAAGGCATACCCAAACATTGAAGTATTACACTGTAGTGATGGATCCTCTTGTAAGTATAATTAGGGATTT

AGAAAAACTTAGTTCTTACTTGTTAGATATGTATAAAGTAGATGCAGGGACCCAATAGCAATTACAGGTAGAC
TCCGTGTTTAAAGGTTCTAATCTTTTTGTTGCAGCACCAAAGACTGGAGATATCTCAGATATGCAATTTTACTAT
GATAAGTGTCTCCCAGGTAATAGCACCATGTTAAATAACTATGATGCTGTTACCATGAGGTTGACTGACATTTC
TCTTAATGTCAAAGATTGCATATTGGATTTCTCTAAGTCTGTGGCTGCACCGAAGGATCCGATCAAACCACTGA
TTCCAATGGTACGAACGGCGGCAGAAATGCCACGCCAGACTGGACTATTGGAAAATTTGGTGGCGATGATCA
AAAGAAACTTTAATTCACCGGAGTTATCAGGAATAATCGACATTGAGAATACTGCATCTTTAGTAGTAGATAA
ATTTTTTGATAGTTACTTGCTTAAAGAAAAAAGAAAACCAAATAAAAATGTTTCTTTATTTTGTAGAGAGTCTCT
CAATAGATGGTTAGAGAAGCAGGAGCAAGTGACCATTGGTCAGCTTGCAGATTTTGATTTTGTGGATCTTCCT
GCCGTTGATCAGTACAGGCATATGATTAAAGCGCAACCTAAGCAGAAGCTGGATACATCAATTCAAAGCGAAT
ATCCGGCCTTGCAGACGATTGTGTATCATTCGAAAAAGATCAACGCAATCTTCGGTCCTTTGTTCAGTGAGCTC
ACAAGGCAAATGCTCGAAAGCATAGACTCAAGTAAGTTTTTGTTCTTTACAAGGAAGACGCCAGCTCAAATTG
AGGATTTCTTCGGAGATCTCGATAGCCATGTCCCTATGGATATCTTGGAGTTGGATATTTCAAAGTATGACAAA
TCTCAGAACGAGTTCCACTGTGCAGTAGAGTATGAAATATGGAGAAGACTTGGATTAGAAGATTTTCTGGGA
GAAGTTTGGAAACAAGGCCACAGAAAAACTACTCTTAAAGATTACACAGCTGGTATTAAAACGTGTTTATGGT
ACCAGAGAAAGAGTGGGGACGTTACAACATTCATCGGTAATACGGTGATTATTGCTGCTTGTTTAGCTTCCAT
GTTGCCCATGGAGAAAATAATCAAAGGTGCATTTTGCGGAGATGACAGTTTACTATACTTCCCAAAAGGTTGT
GAGTTTCCTGACATACAGCATACAGCTAACCTTATGTGGAATTTCGAGGCTAAGCTATTCAGAAAGCAGTATG
GTTATTTCTGTGGAAGGTACGTGATACATCATGACAGAGGGTGTATTGTTTATTATGACCCTTTGAAGTTGATT
TCTAAACTTGGTGCTAAACACATCAAGGATTGGGATCACTTAGAAGAGTTCAGAAGATCCCTTTGTGATGTTG
CAATTTCGTTGAACAACTGTGCGTATTACACGCAGTTGGACGACGCTGTGAGTGAGGTCCATAAAACCGCACC
CCCGGGTTCGTTTGTTTATAAAAGTTTAGTTAAATATCTGTCCGATAAGGTTCTTTTTAGAAGTTTGTTTATAGA
TGGCTCTTGTTAAGGGTAAAGTCAATATTAATGAGTTCATAGACTTGTCAAAATCAGAAAAATTTCTTCCGTCT
ATGTTCACACCTGTTAAGAGTGTCATGATCTCCAAGGTTGATAAGATATTGGTTCATGAAGATGAATCTTTGTC
CGAAGTCAATTTACTCAAAGGTGTAAAACTCATTGATGGTGGCTATGTACATCTTGCTGGTCTTGTAGTGACAG
GTGAATGGAATTTGCCAGATAATTGTCGTGGTGGTGTCAGTGTCTGTTTGGTCGATAAGAGAATGGAGAGAG
CGGACGAGGCAACTCTTGCTTCATACTATACCGCAGCGGCTAAGAAAAGGTTTCAGTTCAAAGTCGTACCAAA
TTACAACATCACTACCAAGGACGCAGAAAAGGCAGTTTGGCAAGTACTAGTTAATATTAGGAATGTTAAAATT
GCTGCGGGTTACTGTCCGCTGTCATTAGAATTTGTGTCAGTGTGTATTGTTTATAAAAATATTATAAAACTCGG
TTTGAGAGAGAAAATTACGAGCGTCACGGATGGAGGGCCCATGGAACTATCAGAAGAAGTTGTTGATGAGTT
CATGGAAGAAGTCCCGATGTCTGTAAGGCTTGCAAAATTTCGTTCGAAGACCGGGAAAAAGTTTAGTAGTAA
AAGTGAGAATAATAGTGGTAATAATAGGCCGAAACCAGACAAAAACCAAAGGAAGGAAAAGGGTTTAAAAG
TTAGGGTTGAGAAGGATAATTTAATTGATAATGAATTGGAGACTTACGTCGCCGATTCAGATTCGTATTAAAT
ATGTCTTACACAATCGCAACTCCATCGCAATTTGTGTTTTTGTCATCAGCATGGGCCGACCCTATAGAATTAATA
AATTTATGTACTAATTCACTAGGTAATCAGTTCCAAACACAACAAGCTAGAACAACCGTTCAACGGCAATTTAG
CGAAGTGTGGAAACCTGTCCCTCAAGTCACTGTTAGGTTTCCTGACAGTGGTTTTAAGGTGTATAGGTACAAT
GCGGTACTAGATCCTCTAGTTACTGCTTTGTTAGGAGCTTTCGATACTAGAAATAGGATTATAGAAGTCGAAA
ATCAGGCGAACCCGACAACCGCCGAAACGTTAGACGCTACTCGTAGAGTAGATGACGCAACGGTGGCTATAA
GGAGCGCTATGAATAATTTAGTAGTAGAATTGGTCAAAGGAACAGGTTTGTACAATCAGAGCACATTTGAAA
GTGCATCCGGTTTACAATGGTCCTCTGCACCTGCATCTTGA

#OM892679

1

ATGGCATACACACAGACAGCTACCACATCCGCTTTGCTCGACACTGTCCGAGGTAACAATACCTTGGTCAACG
ATCTTGCGAAGCGGCGTCTTTATGACACAGCGGTCGACGAGTTCAACGCTCGTGATCGCAGGCCCAAAGTAA

ATTTTTCCAAAGTAATAAGTGAGGAACAGACGCTTATTGCTACTAGGGCATATCCAGAATTCCAGATAACCTTC
TATAATACGCAGAACGCCGTGCATTCGCTTGCCGGTGGACTACGATCCTTAGAACTGGAATATCTAATGATGC
AGATCCCGTACGGATCACTCACATATGATATAGGTGGGAATTTTGCATCTCATCTGTTCAAAGGACGGGCATA
TGTTCACTGCTGTATGCCCAATCTTGATGTCCGCGACATAATGCGGCACGAAGGCCAGAAAGACAGTATAGAA
TTATACCTTTCCAGGCTTGAGCGGGGCAACAAAGTTGTCCCAAATTTCCAAAAGGAAGCTTTTGACAGATACG
CTGAAACGCCAGACGAAGTTGTCTGTCACAGTACCTTCCAAACGTGTACGCACCAGCAGGTGGAAAACACAG
GCAGGGTGTATGCTATTGCATTGCACAGTATATACGATATACCTGCTGATGAATTCGGAGCGGCACTTTTAAG
GAAAAATGTCCATGTTTGTTACGCCGCCTTCCACTTTTCCGAGAATTTACTTCTCGAAGATTCACACGTCAACCT
TGACGAAATCAACGCGTGTTTTTCGCGTGATGGAGACAAGCTGACTTTTTCTTTCGCATCTGAGAGCACTTTAA
ATTATTGTCATAGTTATTCTAATATTTTAAAATACGTGTGCAAAACTTACTTCCCGGCATCTAATAGAGAGGTCT
ACATGAAGGAGTTTTTGGTCACCAGGGTTAACACCTGGTTTTGTAAGTTTTCTAGGATAGATACTTTTTTATTAT
ACAAGGGGGTAGCCCACAAAGGTGTAAATAGTGAGCAATTTTACAGCGCAATGGAAGATGCATGGCACTACA
AAAAGACTCTTGCAATGTGTAACAGCGAGAGGATTCTTCTTGAAGATTCCTCATCGGTCAATTACTGGTTCCCA
AAAATGAGAGATATGGTCATAGTTCCTCTATTCGACATATCTCTCGACACCAGTAAAAGGACCCGCAAAGAAG
TCTTAGTGTCAAAGGATTTTGTATTCACAGTTTTAAATCACATTCGCACTTATCAAGCCAAGGCACTTACATACT
CCAATGTTTTATCCTTCGTCGAATCAATTCGTTCAAGGGTAATTATCAACGGAGTGACTGCCAGGTCTGAGTGG
GATGTTGACAAATCTCTTTTGCAATCCTTGTCCATGACATTTTTCTTGCATACTAAGCTTGCCGTTTTAAAAGAC
GAATTGTTAATCAGCAAGTTTAGTTTGGGGCCAAAATCAGTAAGCCAGCATGTATGGGATGAGATTTCCCTGG
CTTTTGGAAACGCATTTCCATCGATCAAGGAGAGACTGCTAAATCGGAAACTAATTAAAGTGTCGGGAGACGC
ATTAGAAATCAGGGTGCCTGATTTATATGTGACTTTTCACGATAGATTAGTGACTGAGTACAAAACATCGGTG
GATATGCCAGTGCTTGATATCAGAAAGAGAATGGAGGAGACTGAGGTTATGTACAATGCATTGTCTGAGCTA
TCTGTGCTCAAGGAGTCGGACAAGTTCGACGTTGATGTTTTTTCCCGGATGTGCCAGACTTTGGAGGTAGACC
CAATGACTGCAGCAAAGGTTATTGTGGCAGTGATGAGCAACGAGAGCGGACTGACTCTTACATTCGAACAGC
CAACTGAAGCAAATGTCGCATTGGCACTTAAAGATTCAGAAAAAGCCTCTGAGGGTGCACTAGTGGTTACTTC
TAGAGATGTTGAAGAACCATCCATGAAGGGTTCAATGGCAAGAGGAGAGTTACAATTGGCCGGTCTGTCTGG
AGACCAACCAGAGTCTTCCTATACTCGGAACGAGGAAATAGAGTCATTAGAGCAATTCCACATGGCAACGGCT
AGTTCGTTAATTCGGAAACAGATGAGTTCGATTGTGTACACGGGCCCCATTAAAGTTCAGCAAATGAAAAACT
TTATTGATAGCCTGGTAGCATCACTCTCTGCTGCGGTGTCGAACCTAGTCAAGATCCTAAAGGATACAGCTGCT
ATAGATCTCGAAACCCGTCAGAAGTTTGGAGTCTTAGATGTTGCGACCAAAAGATGGTTAATTAAACCTTTAG
CCAAGAATCACGCATGGGGCGTTATTGAAACACATGCTAGGAAGTACCACGTTGCACTTTTGGAGTATGATGA
GCATGGAGTGGTAACTTGCGACAGTTGGAGAAGGGTGGCCGTGAGTTCTGAGTCAATGGTTTATTCTGATAT
GGCAAAGCTCAGAACACTGAGGAGATTATTAAGGGATGGTGAGCCTCATGTCAGCAGTGCTAAAGTCGTCCT
AGTTGACGGTGTCCCGGGTTGTGGAAAGACAAAAGAGATTCTCTCGAAAGTAAATTTTGAGGAAGATCTAAT
CTTAGTACCGGGTAAGCAGGCTGCTGAAATGATAAAGAGGCGTGCTAATGCGTCAGGAATAATTCAAGCCAC
AAGAGATAATGTTCGTACTGTTGATTCATTTATAATGAATTACGGTAAAGGAACACGCTGTCAGTTCAAAAGG
TTATTTATCGACGAAGGTCTGATGTTGCACACTGGTTGTGTGAATTTTCTTGTTTCTATGTCTCTGTGCGAAATT
GCATATGTTTATGGAGACACACAACAAATTCCATACATCAACAGAGTATCCGGTTTTCCGTACCCTGCACATTT
TGGAAAAATAGAGGTTGATGAGGTGGAAACTCGCAGAACTACGCTGCGTTGTCCAGCCGACATTACCCACTA
TCTTAACAGAAGGTACGAAGGATATGTCATGTGTACATCGTCGGTTAAAAAGTCAGTTTCTCAGGAAATGGTG
AGCGGGGCCGCAATGATCAATCCTGTATCTAAGCCACTGAATGGGAAAGTTTTGACTTTCACTCAGTCTGATA
AAGAGGCGCTGCTTTCTCGAGGATATACGGACGTCCATACAGTACATGAGGTACAAGGTGAGACATATGCAG
ATGTGTCGTTGGTCAGATTGACTCCGACACCTGTATCTATCATCGCAGGAGATAGTCCGCACGTTCTCGTAGCT
TTGTCAAGGCATACCCAAACATTGAAGTATTACACCGTAGTGATGGATCCTCTTGTAAGTATAATTAGGGATTT
AGAAAAACTTAGTTCTTACTTGTTAGATATGTATAAAGTAGATGCAGGGACCCAATAGCAATTACTGGTAGAC
TCCGTGTTTAAAGGTTCTAATCTTTTTGTTGCAGCACCAAAGACTGGAGATATCTCAGATATGCAATTTTACTAT
GATAAGTGTCTCCCAGGTAATAGCACCATGTTAAATAACTATGATGCTGTTACCATGAGGTTGACTGACATTTC
TCTTAATGTCAAAGATTGCATATTGGATTTCTCTAAGTCTGTGGCTGCACCGAAGGATCCGATCAAACCACTGA
TTCCAATGGTACGAACGGCGGCAGAAATGCCACGCCAGACTGGACTATTGGAAAATTTGGTGGCGATGATCA

AAAGAAACTTTAATTCACCGGAGTTATCAGGAATAATCGACATTGAGAATACTGCATCTTTAGTAGTAGATAA
ATTTTTTGATAGTTACTTGCTTAAAGAAAAAAGAAAACCAAATAAAAATGTTTCTTTATTTTGTAGAGAGTCTCT
CAATAGATGGTTAGAGAAGCAGGAGCAAGTGACCATTGGTCAGCTTGCAGATTTTGATTTTGTGGATCTTCCT
GCCGTTGATCAGTACAGGCATATGATTAAAGCGCAACCTAAGCAGAAGCTGGATACATCAATTCAAAGCGAAT
ATCCGGCCTTGCAGACGATTGTGTATCATTCGAAAAAGATCAACGCAATCTTCGGTCCTTTGTTCAGTGAGCTC
ACAAGGCAAATGCTCGAAAGCATAGACTCAAGTAAGTTTTTGTTCTTTACAAGGAAGACGCCAGCTCAAATTG
AGGATTTCTTCGGAGATCTCGATAGCCATGTCCCTATGGATATCTTGGAGTTGGATATTTCGAAGTATGACAAA
TCTCAGAACGAGTTCCACTGTGCAGTAGAGTATGAAATATGGAGAAGACTTGGATTAGAAGATTTTCTGGGA
GAAGTTTGGAAACAAGGCCACAGGAAAACTACTCTTAAAGATTACACAGCTGGTATTAAAACGTGTTTATGGT
ACCAGAGAAAGAGTGGGGACGTTACAACATTCATCGGTAATACGGTGATTATTGCTGCTTGTTTAGCTTCCAT
GTTGCCCATGGAGAAAATAATCAAAGGTGCATTTTGCGGAGATGACAGTTTACTATACTTCCCAAAAGGTTGT
GAGTTTCCTGACATACAGCATACAGCCAACCTTATGTGGAATTTCGAGGCTAAGCTATTCAGAAAGCAGTATG
GTTATTTCTGTGGAAGGTACGTGATACATCATGACAGAGGGTGTATTGTTTATTATGACCCTTTGAAGTTGATT
TCTAAACTTGGTGCTAAACACATCAAGGATTGGGATCACTTAGAAGAGTTCAGAAGATCCCTTTGTGATGTTG
CAAATTCGTTGAACAACTGTGCGTATTACACGCAGTTGGACGACGCTGTGAGTGAGGTCCATAAAACCGCACC
CCCGGGTTCGTTTGTGTATAAAAGTTTAGTTAAATATCTGTCCGATAAGGTTCTTTTTAGAAGTTTGTTTATAGA
TGGCTCTTGTTAAGGGTAAAGTCAATATTAATGAGTTCATAGACTTGTCAAAATCAGAAAAATTTCTTCCGTCT
ATGTTCACACCTGTTAAGAGTGTCATGATCTCCAAGGTTGATAAGATATTGGTTCATGAAGATGAATCTTTGTC
CGAAGTCAATTTACTCAAAGGTGTAAAACTCATTGATGGTGGCTATGTACATCTTGCTGGTCTTGTGGTGACA
GGTGAATGGAATTTGCCAGATAATTGTCGTGGTGGTGTCAGTGTCTGTTTGGTCGATAAGAGAATGGAGAGA
GCGGACGAGGCAACTCTTGCTTCATACTATACCGCAGCGGCTAAGAAAAGGTTTCAGTTCAAAGTCGTTCCAA
ATTACAACATCACTACCAAGGACGCAGAAAAGACAGTTTGGCAAGTACTAGTTAATATTAGAAATGTTAAAAT
TGCTGCGGGTTACTGTCCGCTGTCATTAGAATTTGTGTCAGTGTGTATTGTTTATAAAAATATTATAAAACTCG
GTTTGAGAGAGAAAATTACGAGCGTCACGGATGGAGGGCCCATGGAACTATCAGAAGAAGTTGTTGATGAG
TTCATGGAAGAAGTCCCGATGTCTGTAAGGCTTGCAAAATTTCGTTCGAAGACCGGAAAAAAGTTTAGTAGTA
AAAGTGAGAATAATAGTGGTAATAATAGGCCGAAACCAAACAAAAACCAAAGGAAGGAAAAGGGTTTAAAA
GTTAGGGTTGAGAAGGATAATTTAATTGATAATGAATTGGAGACTTACATCGCCGATTCAGATTCGTATTAAA
TATGTCTTACACAATCGCAACTCCATCGCAATTTGTGTTTTTGTCATCAGCATGGGCCGACCCTATAGAATTAAT
AAATTTATGTACTAATTCACTAGGTAATCAGTTCCAAACACAACAAGCTAGAACAACCGTTCAACGGCAATTTA
GCGAAGTGTGGAAACCTGTCCCTCAAGTCACTGTTAGGTTTCCTGACAGTGGTTTTAAGGTGTATAGGTACAA
TGCGGTACTAGATCCTCTAGTTACTGCTTTGTTAGGAGCTTTCGATACTAGAAATAGGATTATAGAAGTCGAAA
ATCAGGCGAACCCGACAACCGCCGAAACGTTAGACGCTACTCGTAGAGTAGATGACGCAACGGTGGCTATAA
GGAGCGCTATAAATAATTTAGTAGTAGAATTGGTCAAAGGAACAGGTTTGTACAATCAGAGCACATTTGAAA
GTGCATCCGGTTTACAATGGTCCTCTGCACCTGCATCTTGA

#OM892682

1

ATGGCATACACACAGACAGCTACCACATCCGCTTTGCTCGACACTGTCCGAGGTAACAATACCTTGGTCAACG
ATCTTGCGAAGCGGCGTCTTTATGACACAGCGGTCGACGAGTTCAACGCTCGTGATCGCAGGCCCAAAGTAA
ATTTTTCCAAAGTAATAAGTGAGGAACAGACGCTTATTGCTACTAGGGCATATCCAGAATTCCAGATAACCTTC
TATAATACGCAGAACGCCGTGCATTCGCTTGCCGGTGGACTACGATCCTTAGAACTGGAATATCTAATGATGC
AGATCCCGTACGGATCACTCACATATGATATAGGTGGGAATTTTGCATCTCATCTGTTCAAAGGACGGGCATA
TGTTCACTGCTGTATGCCCAATCTTGATGTCCGCGACATAATGCGGCACGAAGGCCAGAAAGACAGTATAGAA
TTATACCTTTCCAGGCTTGAGCGGGGCAACAAAGTTGTCCCAAATTTCCAAAAGGAAGCTTTTGACAGATACG

CTGAAACGCCAGACGAAGTTGTCTGTCACAGTACCTTCCAAACGTGTACGCACCAGCAGGTGGAAAACACAG
GCAGGGTGTATGCTATTGCATTGCACAGTATATACGATATACCTGCTGATGAATTCGGAGCGGCACTTTTAAG
GAAAAATGTCCATGTTTGTTACGCCGCCTTCCACTTTTCCGAGAATTTACTTCTCGAAGATTCACACGTCAACCT
TGACGAAATCAACGCGTGTTTTTCGCGTGATGGAGACAAGCTGACTTTTTCTTTCGCATCTGAGAGCACTTTAA
ATTATTGTCATAGTTATTCTAATATTTTAAAATACGTGTGCAAAACTTACTTCCCGGCATCTAATAGAGAGGTCT
ACATGAAGGAGTTTTTGGTCACCAGGGTTAACACCTGGTTTTGTAAGTTTTCTAGGATAGATACTTTTTTATTAT
ACAAGGGGGTAGCCCACAAAGGTGTAAATAGTGAGCAATTTTACAGCGCAATGGAAGATGCATGGCACTACA
AAAAGACTCTTGCAATGTGTAACAGCGAGAGGATTCTTCTTGAAGATTCCTCATCGGTCAATTACTGGTTCCCA
AAAATGAGAGATATGGTCATAGTTCCTCTATTCGACATATCTCTCGACACCAGTAAAAGGACCCGCAAAGAAG
TCTTAGTGTCAAAGGATTTTGTATTCACAGTTCTAAATCACATTCGCACTTATCAAGCCAAGGCACTTACATACT
CCAATGTTTTATCCTTTGTCGAATCAATTCGTTCAAGGGTAATTATCAACGGAGTGACTGCCAGGTCTGAGTGG
GATGTTGACAAATCTCTTTTGCAATCCTTGTCCATGACATTTTTCTTGCATACTAAGCTTGCCGTTTTAAAAGAC
GAATTGTTAATCAGCAAGTTTAGTTTGGGGCCAAAATCAGTAAGCCAGCATGTATGGGATGAGATTTCCCTGG
CTTTTGGAAACGCATTTCCATCGATCAAGGAGAGACTGCTAAATCGGAAACTAATTAAAGTGTCGGGAGACGC
ATTAGAAATCAGGGTGCCTGATTTATATGTGACTTTTCACGATAGATTAGTGACTGAGTACAAAACATCGGTG
GATATGCCAGTGCTTGATATCAGAAAGAGAATGGAGGAGACTGAGGTTATGTACAATGCATTGTCTGAGCTA
TCTGTGCTCAAGGAGTCGGACAAGTTCGACGTTGATGTTTTTTCCCGGATGTGCCAGACTTTGGAGGTAGACC
CAATGACTGCAGCAAAGGTTATTGTGGCAGTGATGAGCAACGAGAGCGGACTGACTCTCACATTCGAACAGC
CAACTGAAGCAAATGTCGCATTGGCACTTAAAGATTCAGAAAAAGCCTCTGAGGGTGCACTAGTGGTTACTTC
TAGAGATGTTGAAGAACCATCCATGAAGGGTTCAATGGCAAGAGGAGAGTTACAATTGGCCGGTCTGTCTGG
AGACCAACCAGAGTCTTCCTATACTCGGAACGAGGAAATAGAGTCATTAGAGCAATTCCACATGGCAACGGCT
AGTTCGTTAATTCGGAAACAGATGAGTTCGATTGTGTACACGGGCCCCATTAAAGTTCAGCAAATGAAAAACT
TTATTGATAGCCTGGTAGCATCACTCTCTGCTGCGGTGTCGAACCTAGTCAAGATCCTAAAGGATACAGCTGCT
ATAGATCTCGAAACCCGTCAGAAGTTTGGAGTCTTAGATGTTGCGACCAAAAGATGGTTAATTAAACCTTTAG
CCAAGAATCACGCATGGGGCGTTATTGAAACACATGCTAGGAAGTACCACGTTGCACTTTTGGAGTATGATGA
GCATGGAGTGGTAACTTGCGACAGTTGGAGAAGGGTGGCCGTGAGTTCTGAGTCAATGGTTTATTCTGATAT
GGCAAAGCTCAGAACACTGAGGAGATTATTAAGAGATGGTGAGCCTCATGTCAGCAGTGCTAAAGTCGTCCT
AGTTGACGGTGTCCCGGGTTGTGGAAAGACAAAAGAGATTCTCTCGAAAGTAAATTTTGAGGAAGATCTAAT
CTTAGTACCGGGTAAGCAGGCTGCTGAAATGATAAAGAGGCGTGCTAATGCGTCAGGAATAATTCAAGCCAC
AAGAGATAATGTTCGTACTGTTGATTCATTTATAATGAATTACGGTAAAGGAACACGCTGTCAGTTCAAAAGG
TTATTTATCGACGAAGGTCTGATGTTGCACACTGGTTGTGTGAATTTTCTTGTTTCTATGTCTCTGTGCGAAATT
GCATATGTTTATGGAGACACACAACAAATTCCATACATCAACAGAGTATCCGGTTTTCCGTACCCTGCACATTT
TGCAAAAATAGAGGTTGATGAGGTGGAAACTCGCAGAACTACGCTGCGTTGTCCAGCCGACATTACCCACTAT
CTTAACAGAAGGTACGAAGGACATGTCATGTGTACATCGTCGGTTAAAAAGTCAGTTTCTCAGGAAATGGTGA
GCGGGGCCGCAATGATCAATCCTGTATCTAAGCCATTGAATGGGAAAGTTTTGACTTTCACTCAGTCTGACAA
AGAGGCGCTGCTTTCTCGAGGATATACGGACGTCCATACAGTACATGAGGTACAAGGTGAGACATATGCAGA
TGTGTCGTTGGTCAGATTGACTCCGACACCTGTATCTATTATCGCAGGAGATAGTCCGCACGTTCTCGTAGCTT
TGTCAAGGCATACCCAAACATTGAAGTATTACACCGTAGTGATGGATCCTCTTGTAAGTATAATTAGGGATTTA
GAAAAACTTAGTTCTTACTTGTTAGATATGTATAAAGTAGATGCAGGGACCCAATAGCAATTACAGGTAGACT
CCGTGTTTAAAGGTTCTAATCTTTTTGTTGCAGCACCAAAGACTGGAGATATCTCAGATATGCAATTTTACTAT
GATAAGTGTCTCCCAGGTAATAGCACCATGTTAAATAACTATGATGCTGTTACCATGAGGTTGACTGACATTTC
TCTTAATGTCAAAGATTGCATATTGGATTTCTCTAAGTCTGTGGCTGCACCGAAGGATCCGATCAAACCACTGA
TTCCAATGGTACGAACGGCGGCAGAAATGCCACGCCAGACTGGACTATTGGAAAATTTGGTGGCGATGATCA
AAAGAAACTTTAATTCACCGGAGTTATCAGGAATAATCGACATTGAGAATACTGCATCTTTAGTAGTAGATAA
ATTTTTTGATAGTTACTTGCTTAAAGAAAAAAGAAAACCAAATAAAAATGTTTCTTTATTTTGTAGAGAGTCTCT
CAATAGATGGTTAGAGAAGCAGGAGCAAGTGACCATTGGTCAGCTTGCAGATTTTGATTTTGTGGATCTTCCT
GCCGTTGATCAGTACAGGCATATGATTAAAGCGCAACCTAAGCAGAAGCTGGATACATCAATTCAAAGCGAAT
ATCCGGCCTTGCAGACGATTGTGTATCATTCGAAAAAGATCAACGCAATCTTCGGTCCTTTGTTCAGTGAGCTC

ACAAGGCAAATGCTCGAAAGCATAGACTCAAGTAAGTTTTTGTTCTTTACAAGGAAGACGCCAGCTCAAATTG
AGGATTTCTTCGGAGATCTCGATAGCCATGTCCCTATGGATATCTTGGAGTTGGATATTTCGAAGTATGACAAA
TCTCAGAACGAGTTCCACTGTGCAGTAGAGTATGAAATATGGAGAAGACTTGGATTAGAAGATTTTCTGGGA
GAAGTTTGGAAACAAGGCCACAGGAAAACTACTCTTAAAGATTACACAGCTGGTATTAAAACGTGTTTATGGT
ACCAGAGAAAGAGTGGGGACGTTACAACATTCATCGGTAATACGGTGATTATTGCTGCTTGTTTAGCTTCCAT
GTTGCCCATGGAGAAAATAATCAAAGGTGCATTTTGCGGAGATGACAGTTTACTATACTTCCCAAAAGGTTGT
GAGTTTCCTGACATACAGCATACAGCCAACCTTATGTGGAATTTCGAGGCTAAGCTATTCAGAAAGCAGTATG
GTTATTTCTGTGGAAGGTACGTGATACATCATGACAGAGGGTGTATTGTTTATTATGACCCTTTGAAGTTGATT
TCTAAACTTGGTGCTAAACACATCAAGGATTGGGATCACTTAGAAGAGTTCAGAAGATCCCTTTGTGATGTTG
CAAATTCGTTGAACAACTGTGCGTATTACACGCAGTTGGACGACGCTGTGAGTGAGGTCCATAAAACCGCACC
CCCGGGTTCGTTTGTATATAAAAGTTTAGTTAAATATCTGTCCGATAAGGTTCTTTTTAGAAGTTTGTTTATAGA
TGGCTCTTGTTAAGGGTAAAGTCAATATTAATGAGTTCATAGACTTGTCAAAATCAGAAAAATTTCTTCCGTCT
ATGTTCACACCTGTTAAGAGTGTCATGATCTCCAAGGTTGATAAGATATTGGTTCATGAAGATGAATCTTTGTC
CGAAGTCAATTTACTCAAAGGTGTAAAACTCATTGATGGTGGCTATGTACATCTTGCTGGTCTTGTGGTGACA
GGTGAATGGAATTTGCCAGATAATTGTCGTGGTGGTGTCAGTGTCTGTTTGGTCGATAAGAGAATGGAGAGA
GCGGACGAGGCAACTCTTGCTTCATACTATACCGCAGCGGCTAAGAAAAGGTTTCAGTTCAAAGTCGTTCCAA
ATTACAACATCACTACCAAGGACGCAGAAAAGGCAGTTTGGCAAGTACTAGTTAATATTAGAAATGTTAAAAT
TGCTGCGGGTTACTGTCCGCTGTCATTAGAATTTGTGTCAGTGTGTATTGTTTATAAAAATATTATAAAACTCG
GTTTGAGAGAGAAAATTACGAGCGTCACGGATGGAGGGCCCATGGAACTATCAGAAGAAGTTGTTGATGAG
TTCATGGAAGAAGTCCCGATGTCTGTAAGGCTTGCAAAATTTCGTGCGAAGACCGGAAAAAAGTTTAGTAGTA
AAAGTGAGAATAATAGTGGTAATAATAGGCCGAAACCAAACAAAAACCAAAGGAAGGAAAAGGGTTTAAAA
ATTAGGGTTGAGAAGGATAATTTAATTGATAATGAATTGGAGACTTACGTCGCCGATTCAGATTCGTATTAAA
TATGTCTTACACAATCGCAACTCCATCGCAATTTGTGTTTTTGTCATCAGCATGGGCCGACCCTATAGAATTAAT
AAATTTATGTACTAATTCACTAGGTAATCAGTTCCAAACACAACAAGCTAGAACAACCGTTCAACGGCAATTTA
GCGAAGTGTGGAAACCTGTCCCTCAAGTCACTGTTAGGTTTCCTGACAGTGGTTTTAAGGTGTATAGGTACAA
TGCGGTACTAGATCCTCTAGTTACTGCTTTGTTAGGAGCTTTCGATACTAGAAATAGGATTATAGAAGTCGAAA
ATCAGGCGAACCCGACAACCGCCGAAACGTTAGACGCTACTCGTAGAGTAGATGACGCAACGGTGGCTATAA
GGAGCGCTATAAATAATTTAGTAGTAGAATTGGTCAAAGGAACAGGTTTGTACAATCAGAGCACATTTGAAA
GTGCATCCGGTTTACAATGGTCCTCTGCACCTGCATCTTGA

#OM892683

1

ATGGCATACACACAGACAGCTACCACATCCGCTTTGCTCGACACTGTCCGAGGTAACAATACCTTGGTCAACG
ATCTTGCGAAGCGGCGTCTTTATGACACAGCGGTCGACGAGTTCAACGCTCGTGATCGCAGGCCCAAAGTAA
ATTTTTCCAAAGTAATAAGTGAGGAACAGACGCTTATTGCTACTAGGGCATATCCAGAATTCCAGATAACCTTC
TATAATACGCAGAACGCCGTGCATTCGCTTGCCGGTGGACTACGATCCTTAGAACTGGAATATCTAATGATGC
AGATCCCGTACGGATCACTCACATATGATATAGGTGGGAATTTTGCATCTCATCTGTTCAAAGGACGGGCATA
TGTTCACTGCTGTATGCCCAATCTTGATGTCCGCGACATAATGCGGCACGAAGGCCAGAAAGACAGTATAGAA
TTATACCTTTCCAGGCTTGAGCGGGGCAACAAAGTTGTCCCAAATTTCCAAAAGGAAGCTTTTGACAGATACG
CTGAAACGCCAGACGAAGTTGTCTGTCACAGTACCTTCCAAACGTGTACGCACCAGCAGGTGGAAAACACAG
GCAGGGTGTATGCTATTGCATTGCACAGTATATACGATATACCTGCTGATGAATTCGGAGCGGCACTTTTAAG
AAAAAATGTCCATGTTTGTTACGCCGCCTTCCACTTTTCCGAGAATTTACTTCTCGAAGATTCACACGTCAACCT
TGACGAAATCAACGCGTGTTTTTCGCGTGATGGAGACAAGCTGACTTTTTCTTTCGCATCTGAGAGCACTTTAA
ATTATTGTCATAGTTATTCTAATATTTTAAAATACGTGTGCAAAACTTACTTCCCGGCATCTAATAGAGAGGTCT

ACATGAAGGAGTTTTTGGTCACCAGGGTTAACACCTGGTTTTGTAAGTTTTCTAGGATAGATACTTTTTTATTAT
ACAAGGGGGTAGCCCACAAAGGTGTAAATAGTGAGCAATTTTACAGCGCAATGGAAGATGCATGGCACTACA
AAAAGACTCTTGCAATGTGTAACAGCGAGAGGATTCTTCTTGAAGATTCCTCATCGGTCAATTACTGGTTCCCA
AAAATGAGAGATATGGTCATAGTTCCTCTATTCGACATATCTCTCGACACCAGTAAAAGGACCCGCAAAGAAG
TCTTAGTGTCAAAGGATTTTGTATTCACAGTTCTAAATCACATTCGCACTTATCAAGCCAAGGCACTTACATACT
CCAATGTTTTATCCTTTGTCGAATCAATTCGTTCAAGGGTAATTATCAACGGAGTGACTGCCAGGTCTGAGTGG
GATGTTGACAAATCTCTTTTGCAATCCTTGTCCATGACATTTTTCTTGCATACTAAGCTTGCCGTTTTAAAAGAC
GAATTGTTAATCAGCAAGTTTAGTTTGGGGCCAAAATCAGTAAGCCAGCATGTATGGGATGAGATTTCCCTGG
CTTTTGGAAACGCATTTCCATCGATCAAGGAGAGACTGCTAAATCGGAAACTAATTAAAGTGTCGGGAGACGC
ATTAGAAATCAGGGTGCCTGATTTATATGTGACTTTTCACGATAGATTAGTGACTGAGTACAAAACATCGGTG
GATATGCCAGTGCTTGATATCAGAAAGAGAATGGAGGAGACTGAGGTTATGTACAATGCATTGTCTGAGCTA
TCTGTGCTCAAGGAGTCGGACAAGTTCGACGTTGATGTTTTTTCCCGGATGTGCCAGACTTTGGAGGTAGACC
CAATGACTGCAGCAAAGGTTATTGTGGCAGTGATGAGCAACGAGAGCGGACTGACTCTCACATTCGAACAGC
CAACTGAAGCAAATGTCGCATTGGCACTTAAAGATTCAGAAAAAGCCTCTGAGGGTGCACTAGTGGTTACTTC
TAGAGATGTTGAAGAACCATCCATGAAGGGTTCAATGGCAAGAGGAGAGTTACAATTGGCCGGTCTGTCTGG
AGACCAACCAGAGTCTTCCTATACTCGGAACGAGGAAATAGAGTCATTAGAGCAATTCCACATGGCAACGGCT
AGTTCGTTAATTCGGAAACAGATGAGTTCGATTGTGTACACGGGCCCCATTAAAGTTCAGCAAATGAAAAACT
TTATTGATAGCCTGGTAGCATCACTCTCTGCTGCGGTGTCGAACCTAGTCAAGATCCTAAAGGATACAGCTGCT
ATAGATCTCGAAACCCGTCAGAAGTTTGGAGTCTTAGATGTTGCGACCAAAAGATGGTTAATTAAACCTTTAG
CCAAGAATCACGCATGGGGCGTTATTGAAACACATGCTAGGAAGTACCACGTTGCACTTTTGGAGTATGATGA
GCATGGAGTGGTAACTTGCGACAGTTGGAGAAGGGTGGCCGTGAGTTCTGAGTCAATGGTTTATTCTGATAT
GGCAAAGCTCAGAACACTGAGGAGATTATTAAGAGATGGTGAGCCTCATGTCAGCAGTGCTAAAGTCGTCCT
AGTTGACGGTGTCCCGGGTTGTGGAAAGACAAAAGAGATTCTCTCGAAAGTAAATTTTGAGGAAGATCTAAT
CTTAGTACCGGGTAAGCAGGCTGCTGAAATGATAAAGAGGCGTGCTAATGCGTCAGGAATAATTCAAGCCAC
AAGAGATAATGTTCGTACTGTTGATTCATTTATAATGAATTACGGTAAAGGAACACGCTGTCAGTTCAAAAGG
TTATTTATCGACGAAGGTCTGATGTTGCACACTGGTTGTGTGAATTTTCTTGTTTCTATGTCTCTGTGCGAAATT
GCATATGTTTATGGAGACACACAACAAATTCCATACATCAACAGAGTATCCGGTTTTCCGTACCCTGCACATTT
TGCAAAAATAGAGGTTGATGAGGTAGAAACTCGCAGAACTACGCTGCGTTGTCCAGCCGACATTACCCACTAT
CTTAACAGAAGGTACGAAGGACATGTCATGTGTACATCGTCGGTTAAAAAGTCAGTTTCTCAGGAAATGGTGA
GCGGGGCCGCAATGATCAATCCTGTATCTAAGCCATTGAATGGGAAAGTTTTGACTTTCACTCAGTCTGACAA
AGAGGCGCTGCTTTCTCGAGGATATACGGACGTCCATACAGTACATGAGGTACAAGGTGAGACATATGCAGA
TGTGTCGTTGGTCAGATTGACTCCGACACCTGTATCTATTATCGCAGGAGATAGTCCGCACGTTCTCGTAGCTT
TGTCAAGGCATACCCAAACATTGAAGTATTACACCGTAGTGATGGATCCTCTTGTAAGTATAATTAGGGATTTA
GAAAAACTTAGTTCTTACTTGTTAGATATGTATAAAGTAGATGCAGGGACCCAATAGCAATTACAGGTAGACT
CCGTGTTTAAAGGTTCTAATCTTTTTGTTGCAGCACCAAAGACTGGAGATATCTCAGATATGCAATTTTACTAT
GATAAGTGTCTCCCAGGTAATAGCACCATGTTAAATAACTATGATGCTGTTACCATGAGGTTGACTGACATTTC
TCTTAATGTCAAAGATTGCATATTGGATTTCTCTAAGTCTGTGGCTGCACCGAAGGATCCGATCAAACCACTGA
TTCCAATGGTACGAACGGCGGCAGAAATGCCACGCCAGACTGGACTATTGGAAAATTTGGTGGCGATGATCA
AAAGAAACTTTAATTCACCGGAGTTATCAGGAATAATCGACATTGAGAATACTGCATCTTTAGTAGTAGATAA
ATTTTTTGATAGTTACTTGCTTAAAGAAAAAAGAAAACCAAATAAAAATGTTTCTTTATTTTGTAGAGAGTCTCT
CAATAGATGGTTAGAGAAGCAGGAGCAAGTGACCATTGGTCAGCTTGCAGATTTTGATTTTGTGGATCTTCCT
GCCGTTGATCAGTACAGGCATATGATTAAAGCGCAACCTAAGCAGAAGCTGGATACATCAATTCAAAGCGAAT
ATCCGGCCTTGCAGACGATTGTGTATCATTCGAAAAAGATCAACGCAATCTTCGGTCCTTTGTTCAGTGAGCTC
ACAAGGCAAATGCTCGAAAGCATAGACTCAAGTAAGTTTTTGTTCTTTACAAGGAAGACGCCAGCTCAAATTG
AGGATTTCTTCGGAGATCTCGATAGCCATGTCCCTATGGATATCTTGGAGTTGGATATTTCGAAGTATGACAAA
TCTCAGAACGAGTTCCACTGTGCAGTAGAGTATGAAATATGGAGAAGACTTGGATTAGAAGATTTTCTGGGA
GAAGTTTGGAAACAAGGCCACAGGAAAACTACTCTTAAAGATTACACAGCTGGTATTAAAACGTGTTTATGGT
ACCAGAGAAAGAGTGGGGACGTTACAACATTCATCGGTAATACGGTGATTATTGCTGCTTGTTTAGCTTCCAT

GTTGCCCATGGAGAAAATAATCAAAGGTGCATTTTGCGGAGATGACAGTTTACTATACTTCCCAAAAGGTTGT
GAGTTTCCTGACATACAGCATACAGCCAACCTTATGTGGAATTTCGAGGCTAAGCTATTCAGAAAGCAGTATG
GTTATTTCTGTGGAAGGTACGTGATACATCATGACAGAGGGTGTATTGTTTATTATGACCCTTTGAAGTTGATT
TCTAAACTTGGTGCTAAACACATCAAGGATTGGGATCACTTAGAAGAGTTCAGAAGATCCCTTTGTGATGTTG
CAAATTCGTTGAACAACTGTGCGTATTACACGCAGTTGGACGACGCTGTGAGTGAGGTCCATAAAACCGCACC
CCCGGGTTCGTTTGTATATAAAAGTTTAGTTAAATATCTGTCCGATAAGGTTCTTTTTAGAAGTTTGTTTATAGA
TGGCTCTTGTTAAGGGTAAAGTCAATATTAATGAGTTCATAGACTTGTCAAAATCAGAAAAATTTCTTCCGTCT
ATGTTCACACCTGTTAAGAGTGTCATGATCTCCAAGGTTGATAAGATATTGGTTCATGAAGATGAATCTTTGTC
CGAAGTCAATTTACTCAAAGGTGTAAAACTCATTGATGGTGGCTATGTACATCTTGCTGGTCTTGTGGTGACA
GGTGAATGGAATTTGCCAGATAATTGTCGTGGTGGTGTCAGTGTCTGTTTGGTCGATAAGAGAATGGAGAGA
GCGGACGAGGCAACTCTTGCTTCATACTATACCGCAGCGGCTAAGAAAAGGTTTCAGTTCAAAGTCGTTCCAA
ATTACAACATCACTACCAAGGACGCAGAAAAGGCAGTTTGGCAAGTACTAGTTAATATTAGAAATGTTAAAAT
TGCTGCGGGTTACTGTCCGCTGTCATTAGAATTTGTGTCAGTGTGTATTGTTTATAAAAATATTATAAAACTCG
GTTTGAGAGAGAAAATTACGAGCGTCACGGATGGAGGGCCCATGGAACTATCAGAAGAAGTTGTTGATGAG
TTCATGGAAGAAGTCCCGATGTCTGTAAGGCTTGCAAAATTTCGTGCGAAGACCGGAAAAAAGTTTAGTAGTA
AAAGTGAGAATAATAGTGGTAATAATAGGCCGAAACCAAACAAAAACCAAAGGAAGGAAAAGGGTTTAAAA
ATTAGGGTTGAGAAGGATAATTTAATTGATAATGAATTGGAGACTTACGTCGCCGATTCAGATTCGTATTAAA
TATGTCTTACACAATCGCAACTCCATCGCAATTTGTGTTTTTGTCATCAGCATGGGCCGACCCTATAGAATTAAT
AAATTTATGTACTAATTCACTAGGTAATCAGTTCCAAACACAACAAGCTAGAACAACCGTTCAACGGCAATTTA
GCGAAGTGTGGAAACCTGTCCCTCAAGTCACTGTTAGGTTTCCTGACAGTGGTTTTAAGGTGTATAGGTACAA
TGCGGTACTAGATCCTCTAGTTACTGCTTTGTTAGGAGCTTTCGATACTAGAAATAGGATTATAGAAGTCGAAA
ATCAGGCGAACCCGACAACCGCCGAAACGTTAGACGCTACTCGTAGAGTAGATGACGCAACGGTGGCTATAA
GGAGCGCTATAAATAATTTAGTAGTAGAATTGGTCAAAGGAACAGGTTTGTACAATCAGAGCACATTTGAAA
GTGCATCCGGTTTACAATGGTCCTCTGCACCTGCATCTTGA

#OM892686

1

ATGGCATACACACAGACAGCTACCACATCCGCTTTGCTCGACACTGTCCGAGGTAACAATACCTTGGTCAACG
ATCTTGCGAAGCGGCGTCTTTATGACACAGCGGTCGACGAGTTCAACGCTCGTGATCGCAGGCCCAAAGTAA
ATTTTTCCAAAGTAATAAGTGAGGAACAGACGCTTATTGCTACTAGGGCATATCCAGAATTCCAGATAACCTTC
TATAATACGCAGAACGCCGTGCATTCGCTTGCCGGTGGACTACGATCCTTAGAACTGGAATATCTAATGATGC
AGATCCCGTACGGATCACTCACATATGATATAGGTGGGAATTTTGCATCTCATCTGTTCAAAGGACGGGCATA
TGTTCACTGCTGTATGCCCAATCTTGATGTCCGCGACATAATGCGGCACGAAGGCCAGAAAGACAGTATAGAA
TTATACCTTTCCAGGCTTGAGCGGGGCAACAAAGTTGTCCCAAATTTCCAAAAGGAAGCTTTTGACAGATACG
CTGAAACGCCAGACGAAGTTGTCTGTCACAGTACCTTCCAAACGTGTACGCACCAGCAGGTGGAAAACACAG
GCAGGGTGTATGCTATTGCATTGCACAGTATATACGATATACCTGCTGATGAATTCGGAGCGGCACTTTTAAG
GAAAAATGTCCATGTTTGTTACGCCGCCTTCCACTTTTCCGAGAATTTACTTCTCGAAGATTCACACGTCAACCT
TGACGAAATCAACGCGTGTTTTTCGCGTGATGGAGACAAGCTGACTTTTTCTTTCGCATCTGAGAGCACTTTAA
ATTATTGTCATAGTTATTCTAATATTTTAAAATACGTGTGCAAAACTTACTTCCCGGCATCTAATAGAGAGGTCT
ACATGAAGGAGTTTTTGGTCACCAGGGTTAACACCTGGTTTTGTAAGTTTTCTAGGATAGATACTTTTTTATTAT
ACAAGGGGGTAGCCCACAAAGGTGTAAATAGTGAGCAATTTTACAGCGCAATGGAAGATGCATGGCACTACA
AAAAGACTCTTGCAATGTGTAACAGCGAGAGGATTCTTCTTGAAGATTCCTCATCGGTCAATTACTGGTTCCCA
AAAATGAGAGATATGGTCATAGTTCCTCTATTCGACATATCTCTCGACACCAGTAAAAGGACCCGCAAAGAAG
TCTTAGTGTCAAAGGATTTTGTATTCACAGTTTTAAATCACATTCGCACTTATCAAGCCAAGGCACTTACATACT

CCAATGTTTTATCCTTTGTCGAATCAATTCGTTCAAGGGTAATTATCAACGGAGTGACTGCCAGGTCTGAGTGG
GATGTTGACAAATCTCTTTTGCAATCCTTGTCCATGACATTTTTCTTGCATACTAAGCTTGCCGTTTTAAAAGAC
GAATTGTTAATCAGCAAGTTTAGTTTGGGGCCAAAATCAGTAAGCCAGCATGTATGGGATGAGATTTCCCTGG
CTTTTGGAAACGCATTTCCATCGATCAAGGAGAGACTGCTAAATCGGAAACTAATTAAAGTGTCGGGAGACGC
ATTAGAAATCAGGGTGCCTGATTTATATGTGACTTTTCACGATAGATTAGTGACTGAGTACAAAACATCGGTG
GATATGCCAGTGCTTGATATCAGAAAGAGAATGGAGGAGACTGAGGTTATGTACAATGCATTGTCTGAGCTA
TCTGTGCTCAAGGAGTCGGACAAGTTCGACGTTGATGTTTTTTCCCGGATGTGCCAGACTTTGGAGGTAGACC
CAATGACTGCAGCAAAGGTTATTGTGGCAGTGATGAGCAACGAGAGCGGACTGACTCTTACATTCGAACAGC
CAACTGAAGCAAACGTCGCATTGGCACTTAAAGATTCAGAAAAAGCCTCTGAGGGTGCACTAGTGGTTACTTC
TAGAGATGTTGAAGAACCATCCATGAAGGGTTCAATGGCAAGAGGAGAGTTACAATTGGCCGGTCTGTCTGG
AGACCAACCAGAGTCTTCCTATACTCGGAACGAGGAAATAGAGTCATTAGAGCAATTCCACATGGCAACGGCT
AGTTCGTTAATTCGGAAACAGATGAGTTCGATTGTGTACACGGGCCCCATTAAAGTTCAGCAAATGAAAAACT
TTATTGATAGCCTGGTAGCATCACTCTCTGCTGCGGTGTCGAACCTAGTCAAGATCCTAAAGGATACAGCTGCT
ATAGATCTCGAAACCCGTCAGAAGTTTGGAGTCTTAGATGTTGCGACCAAAAGATGGTTAATTAAACCTTTAG
CCAAGAATCACGCATGGGGCGTTATTGAAACACATGCTAGGAAGTACCACGTTGCACTTTTGGAGTATGATGA
GCATGGAGTGGTAACTTGCGACAGTTGGAGAAGGGTGGCCGTGAGTTCTGAGTCAATGGTTTATTCTGATAT
GGCAAAGCTCAGAACACTGAGGAGATTACTAAGGGATGGTGAGCCTCATGTCAGCAGTGCTAAAGTCGTCCT
AGTTGACGGTGTCCCGGGTTGTGGAAAGACAAAAGAGATTCTCTCGAAAGTAAATTTTGAGGAAGATCTAAT
CTTAGTACCGGGTAAGCAGGCTGCTGAAATGATAAAGAGGCGTGCTAATGCGTCAGGAATAATTCAAGCCAC
AAGAGATAATGTTCGTACTGTTGATTCATTTATAATGAATTACGGTAAAGGAACACGCTGTCAGTTCAAAAGG
TTATTTATCGACGAAGGTCTGATGTTGCACACTGGTTGTGTGAATTTTCTTGTTTCTATGTCTCTGTGCGAAATT
GCATATGTTTATGGAGACACACAACAAATTCCATACATCAACAGAGTATCCGGTTTTCCGTACCCTGCACATTT
TGCAAAAATAGAGGTTGATGAGGTGGAAACTCGCAGAACTACGCTGCGTTGTCCAGCCGACATTACCCACTAT
CTTAACAGAAGGTACGAAGGACATGTCATGTGTACATCGTCGGTTAAAAAGTCAGTTTCTCAGGAAATGGTGA
GCGGGGCCGCAATGATCAATCCTGTATCTAAGCCACTGAATGGGAAAGTTTTGACTTTCACTCAGTCTGATAA
AGAGGCGCTGCTTTCTCGAGGATATACGGACGTCCATACAGTACATGAGGTACAAGGTGAGACATATGCAGA
TGTGTCGTTGGTCAGATTGACTCCGACACCTGTATCTATCATCGCAGGAGATAGTCCGCACGTTCTCGTAGCTT
TGTCAAGGCATACCCAAACATTGAAGTATTACACCGTAGTGATGGATCCTCTTGTAAGTATAATTAGGGATTTA
GAAAAACTTAGTTCTTACTTGTTAGATATGTATAAAGTAGATGCAGGGACCCAATAGCAATTACAGGTAGACT
CCGTGTTTAAAGGTTCTAATCTTTTTGTTGCAGCACCAAAGACTGGAGATATCTCAGATATGCAATTTTACTAT
GATAAGTGTCTCCCAGGTAATAGCACCATGTTAAATAACTATGATGCTGTTACCATGAGGTTGACTGACATTTC
TCTTAATGTCAAAGATTGCATATTGGATTTCTCTAAGTCTGTGGCTGCACCGAAGGATCCGATCAAACCACTGA
TTCCAATGGTACGAACGGCGGCAGAAATGCCACGCCAGACTGGACTATTGGAAAATTTGGTGGCGATGATCA
AAAGAAACTTTAATTCACCGGAGTTATCAGGAACAATCGACATTGAGAATACTGCATCTTTAGTAGTAGATAA
ATTTTTTGATAGTTACTTGCTTAAAGAAAAAAGAAAACCAAATAAAAATGTTTCTTTATTTTGTAGAGAGTCTCT
CAATAGATGGTTAGAGAAGCAGGAGCAAGTGACCATTGGTCAGCTTGCAGATTTTGATTTTGTAGATCTTCCT
GCCGTTGATCAGTACAGACATATGATTAAAGCGCAACCTAAGCAGAAGCTGGATACATCAATTCAAAGCGAAT
ATCCGGCCTTGCAGACGATTGTGTATCATTCGAAAAAGATCAACGCAATCTTCGGTCCTTTGTTCAGTGAGCTC
ACAAGGCAAATGCTTGAAAGCATAGACTCAAGTAAGTTTTTGTTCTTTACAAGGAAGACGCCAGCTCAAATTG
AGGATTTCTTCGGAGATCTCGATAGCCATGTCCCTATGGATATCTTGGAGTTGGATATTTCGAAGTATGACAAA
TCTCAGAACGAGTTCCACTGTGCAGTAGAGTATGAAATATGGAGAAGACTTGGATTAGAAGATTTTCTGGGA
GAAGTTTGGAAACAAGGCCACAGGAAAACTACTCTTAAAGATTACACAGCTGGTATTAAAACGTGTTTATGGT
ACCAGAGAAAGAGTGGGGACGTTACAACATTCATCGGTAATACGGTGATTATTGCTGCTTGTTTAGCTTCCAT
GTTGCCCATGGAGAAAATAATCAAAGGTGCATTTTGCGGAGATGACAGTTTACTATACTTCCCAAAAGGTTGT
GAGTTTCCTGACATACAGCATACAGCCAACCTTATGTGGAATTTCGAGGCTAAGCTATTCAGAAAGCAGTATG
GTTATTTCTGTGGAAGGTACGTGATACATCATGACAGAGGGTGTATTGTTTATTATGACCCTTTGAAGTTGATT
TCTAAACTTGGTGCTAAACACATCAAGGATTGGGATCACTTAGAAGAGTTCAGAAGATCCCTTTGTGATGTTG
CAAATTCGTTGAACAACTGTGCGTATTACACGCAGTTGGACGACGCTGTGAGTGAGGTCCATAAAACCGCACC

CCCGGGTTCGTTTGTGTATAAAAGTTTAGTTAAATATCTGTCCGATAAGGTTCTTTTTAGAAGTTTGTTTATAGA
TGGCTCTTGTTAAGGGTAAAGTCAATATTAATGAGTTCATAGACTTGTCAAAATCAGAAAAATTTCTTCCGTCT
ATGTTCACACCTGTTAAGAGTGTCATGATCTCCAAGGTTGATAAGATATTGGTTCATGAAGATGAATCTTTGTC
CGAAGTCAATTTACTCAAAGGTGTAAAACTCATTGATGGTGGCTATGTACATCTTGCTGGTCTTGTGGTGACA
GGTGAATGGAATTTGCCAGATAATTGTCGTGGTGGTGTCAGTGTCTGTTTGGTCGATAAGAGAATGGAGAGA
GCGGACGAGGCAACTCTTGCTTCATACTATACCGCAGCGGCTAAGAAAAGGTTTCAGTTCAAAGTCGTTCCAA
ATTACAACATCACTACCAAGGACGCAGAAAAGGCAGTTTGGCAAGTACTAGTTAATATTAGAAATGTTAAAAT
TGCTGCGGGTTACTGTCCGCTGTCATTAGAATTTGTGTCAGTGTGTATTGTTTATAAAAATATTATAAAACTCG
GTTTGAGAGAGAAAATTACGAGCGTCACGGATGGAGGGCCCATGGAACTATCAGAAGAAGTTGTTGATGAG
TTCATTGAAGAAGTCCCGATGTCTGTAAGGCTTGCAAAATTTCGTTCGAAGACCGGAAAAAAGTTTAGTAGTA
AAAGTGAGAATAATAGTGGTAATAATAGGCCGAAACCAAACAAAAACCAAAGGAAGGAAAAGGGTTTAAAA
GTTAGGGTTGAGAAGGATAATTTAATTGATAATGAATTGGAGACTTACATCGCCGATTCAGATTCGTATTAAA
TATGTCTTACACAATCGCAACTCCATCGCAATTTGTGTTTTTGTCATCAGCATGGGCCGACCCTATAGAATTAAT
AAATTTATGTACTAATTCACTAGGTAATCAGTTCCAAACACAACAAGCTAGAACAACCGTTCAACGGCAATTTA
GCGAAGTGTGGAAACCTGTCCCTCAAGTCACTGTTAGGTTTCCTGACAGTGGTTTTAAGGTGTATAGGTACAA
TGCGGTACTAGATCCTCTAGTTACTGCTTTGTTAGGAGCTTTCGATACTAGAAATAGGATTATAGAAGTCGAAA
ATCAGGCGAACCCGACAACCGCCGAAACGTTAGACGCTACTCGTAGAGTAGATGACGCAACGGTGGCTATAA
GGAGCGCTATAAATAATTTAGTAGTAGAATTGGTCAAAGGAACAGGTTTGTACAATCAGAGCACATTTGAAA
GTGCATCCGGTTTACAATGGTCCTCTGCACCTGCATCTTGA

#OP557566

1

ATGGCATACACACAGACAGCTACCACATCCGCTTTGCTCGACACTGTCCGAGGTAACAATACCTTGGTCAACG
ATCTTGCGAAGCGGcGTcTTTATGACACAGCGGTCGACGAGTTCAACGCTCGTGATCGCAGGCCCAAAGTAAA
TTTTTCCAAAGTAATAAGTGAGGAACAGACGCTTATTGCTACTAGGGCATATCCAGAATTCCAGATAaCCTTCT
ATAATACGCAGAACGCCGTGCATTCGcTTGCCGGTGGACTACGATCCTTAGAACTGGAATATCTAATGAtGCA
GATCCCGTACGGATCACTCACAtATGATATAGGTGGGAATTTTGCATCTCATcTGTTCAAAGGACGGGCATATG
TTCACTGCTGTATGCCCAATcTTGATGTCCGCGACATAATGCGGCaCGAAGGCCAGAAAgACAGTATAGAATTA
TACCTTTCCAGGcTtGAGCGGGGCAACAAAGTTGTCCCAAATTTCCAAAAGGAAGCTTTTGACaGATACGCTGA
AACgCCAGACGAAGTTGTcTGTCaCAGTaCCTTCCAAACGtGTACgCaCCAGCAGGtGGAAAACaCAGGCaGGG
tGTaTGcTATTGCATTGCACAGTATATACGATATACCTGCTGATGAATTCGGAGcGGCACTTTTAAGGAAAAAT
GTCCATGTTTGTTACgCCgCCTTCCaCTTTTCCGAGAATTTACTTCTCGAAgATTCaCaCGTCAACCTTGACGAAA
TCAACGcGtGTTTTTCGCGTGATGGAGACAAGCtGACTTTTTCTTTCGCATCTGaGAGCaCTTTAAATTATTGTCA
TAGTTATTcTAATATTTTAAAATACGTGTGCAAAACTTACTTCCCGGCATCTAATAGAGAGGTCTACAtGAAGG
AGTTTTTGGTCACCAGGGTTAACACCtGGTTTTGTAAGTTTTCTAGGATAgATACTTTTTTaTTATACAAGGGGG
TAGCCCACAAAGGTGTAAAtAGtGAGCAATTTTACAGCGCAATGGAAGATGCATGGCACTACAAAAAGACTCT
TGCAATGTGTAACAGCGAGAGGATTCTTCTTGAAGATTCCTCATCGGTCAATTACTGGTTCCCAAAAATGAGA
GATATGGTCATAGTTCCTCTATTCGACATATCTCTCGACACCAGTAAAAGGACCCGCAAAGAAGTCTTAGTGTC
AAAGGATTTTGTATTCACAGTTTTAAATCACATTCGCACTTATCAAGCCAAGGCACTTACATACTCCAATGTTTT
ATCCTTTGTCGAATCAATTCGTTCAAGGGTAATTATCAACGGAGTGACTGCCAGGTCTGAGTGGGATGTTGAC
AAATCTCTTTTGCAATCCTTGTCCATGACATTTTTCTTGCATACTAAGCTTGCCGTTTTAAAAGACGAATTGTTA
ATCAGCAAGTTTAGTTTGGGGCCAAAATCAGTAAGCCAGCATGTATGGGATGAGATTTCCCTGGCTTTTGGAA
ACGCATTTCCATCGATCAAGGAGAGACTGCTAAATCGGAAACTAATTAAAGTGTCGGGAGACGCATTAGAAA
TCAGAGTGCCTGATTTATATGTGACTTTTCACGATAGATTAGTGGCTGAGTACAAAACATCGGTGGATATGCC

AGTGCTTGATATCAGAAAGAGAATGGAGGAGACTGAGGTTATGTACAATGCATTGTCTGAGCTATCTGTGCTC
AAGGAATCGGACAAGTTCGACGTTGATGTTTTTTCCCGGATGTGCCAGACTTTGGAGGTAGACCCAATGACTG
CAGCAAAGGTTATTGTGGCAGTGATGAGCAACGAGAGCGGACTGACTCTTACATTCGAACAGCCAACTGAAG
CAAATGTCGCATTGGCACTTAAAGATTCAGAAAAAGCCTCTGAGGGTGCACTAGTGGTTACTTCTAGAGATGT
TGAAGAACCATCCATGAAGGGTTCAATGGCAAGAGGAGAGTTACAATTGGCCGGTCTGTCTGGAGACCAACC
AGAGTCTTCCTATACTCGGAACGAGGAAATAGAGTCATTAGAGCAATTCCACATGGCAACGGCTAGTTCGTTA
ATTCGGAAACAGATGAGTTCGATTGTGTACACGGGCCCCACTAAAGTTCAGCAAATGAAAAACTTTATTGATA
GCCTGGTAGCATCACTCTCTGCTGCGGTGTCGAACCTAGTCAAGATCCTAAAGGATACAGCTGCTATAGATCT
CGAAACCCGTCAGAAGTTTGGAGTCTTAGATGTTGCGACCAAAAGATGGTTAATTAAACCTTTAgCCAAgAATC
aCGCaTGGGGcGTTaTTGAAACACATGCTAGGAAGTACCACGTTGCAcTTTTGGAGTATGATGAGCATGGAGt
GGtAACTTGCgACAGTtGgAgAAGGGtGGCCGtGAGTTCTGAGTCAATGGTTTATTCTGATATGGCAAAgCTCAg
AACACTGAGGAGATTATTAAGAGATGGTGagCCTCATGTCAGCAGTGCTAAAGTCGTCCTAGTTGACGGTGTC
CCGGGTTGTGGAAAGACAAAAGAGATTCTCTCGAAagTAAATTTTGAGGAAGATCTAATCTTAGTACcGGGTA
AGCAGGCTGCTGAAATGATAAAGAGGCGTGCTAATGCGTCAGGAATAATTCAAGCCACAAGAGATAATGTTC
GTACTGTTGATTCATTTATAATGAATTACGGTAAAGGAACACGCTGTCAGTTCAAAAGGTTATTTATCGACGAA
GGTCTGATGTTGCACACTGGTTGTGTGAATTTTCTTGTTTCTATGTCTCTGTGCGAAATTGCATATGTTTATGGA
GACACACAACAAATTCCATACATCAACAGAGTATCCGGTTTTCCGTACCCTGCACATTTTGCAAAAATAGAGGT
TGATGAGGTGGAAACTCGCAGAACTACGCTGCGTTGTCCAGCCGACATTACCCACTATCTTAACAGAAGGTAC
GAAGGATATGTCATGTGTACATCGTCGGTTAAAAAGTCAGTTTCTCAGGAAATGGTGAGCGGGGCCGCAATG
ATCAATCCTGTATCTAGGCCATTGAATGGGAAAGTTTTGACTTTCACTCAGTCTGATAAAGAGGCGCTGCTTTC
TCGAGGATATACGGACGTCCATACAGTACATGAGGTACAAGGTGAGACATATGCAGATGTGTCGTTGGTCAG
ATTGACTCCGACACCTATATCTATCATCGCAGGAGATAGTCCGCACGTTCTCGTAGCTTTGTCAAGGCATACCC
AAACATTGAAGTATTACACCGTAGTGATGGATCCTCTTGTAAGTATAATTAGGGATTTAGAAAAACTTAGTTCT
TACTTGTTAGATATGTATAAAGTAGATGCAGGGACCCAATAGCAATTACAGGTAGACTCCGTGTTTAAAGGTT
CTAATCTTTTTGTTGCAGCACCAAAGACTGGAGATATCTCAGATATGCAATTTTACTATGATAAGTGTCTCCCA
GGTAATAGCACCATGTTAAATAACTATGATGCTGTTACCATGAGGTTGACTGACATTTCTCTTAATGTCAAAGA
TTGCATATTGGATTTCTCTAAGTCTGTGGCTGCACCGAAGGATCCGATCAAACCACTGATTCCAATGGTACGAA
CGGCGGCAGAAATGCCACGCCAGACTGGACTATTGGAAAATTTGGTGGCGATGATCAAAAGAAACTTTAATT
CACCGGAGTTATCAGGAATAATCGACATTGAGAATACTGCATCTTTAGTAGTAGATAAATTTTTTGATAGTTAC
TTGCTTAAAGAAAAAAGAAAACCAAATAAAAATGTTTCTTTATTTTGTAGAGAGTCTCTCAATAGATGGTTAGA
GAAGCAGGAGCAAGTGACCATTGGTCAGCTTGCAGATTTTGATTTTGTGGATCTTCCTGCCGTTGATCAGTAC
AGGCATATGATTAAAGCGCAACCTAAGCAGAAGCTGGATACATCAATTCAAAGCGAATATCCGGCCTTGCAG
ACGATTGTGTATCATTCGAAAAAGATCAACGCAATCTTCGGTCCTTTGTTCAGTGAGCTCACAAGGCAAATGCT
CGAAAGCATAGACTCAAGTAAGTTTTTGTTCTTTACAAGGAAGACGCCAGCTCAAATTGAGGATTTCTTCGGA
GATCTCGATAGCCATGTCCCTATGGATATCTTGGAGTTGGATATTTCGAAGTATGACAAATCTCAGAACGAGTT
CCACTGTGCAGTAGAGTATGAAATATGGAGAAGACTTGGATTAGAAGATTTTCTGGGAGAAGTTTGGAAACA
AGGCCACAGGAAAACTACTCTTAAAGATTACACAGCTGGTATTAAAACGTGTTTATGGTACCAGAGAAAGAGT
GGGGACGTTACAACATTCATCGGTAATACGGTGATTATTGCTGCTTGTTTAGCTTCCATGTTGCCCATGGAGAA
AATAATCAAAGGTGCATTTTGCGGAGATGACAGTTTACTATACTTCCCAAAAGGTTGTGAGTTTCCTGACATAC
AGCATACAGCCAACCTTATGTGGAATTTCGAGGCTAAGCTATTCAGAAAGCAGTATGGTTATTTCTGTGGAAG
GTACGTGATACATCATGACAGAGGGTGTATTGTTTATTATGACCCTTTGAAGTTGATTTCTAAACTTGGTGCTA
AACACATCAAGGATTGGGATCACTTAGAAGAGTTCAGAAGATCCCTTTGTGATGTTGCAAATTCGTTGAACAA
CTGTGCGTATTACACGCAGTTGGACGACGCTGTGAGTGAGGTCCATAAAACCGCACCCCCGGGTTCGTTTGTA
TATAAAAGTTTAGTTAAATATCTGTCCGATAAGGTTCTTTTTAGAAGTTTGTTTATAGATGGCTCTTGTTAAGG
GTAAAATCAATATTAATGAGTTCATAGACTTGTCAAAATCAAAAAAATTTCTTCCGTCTATGTTCACACCTGTTA
AGAGTGTCATGATCTCCAAGGTTGATAAGATATTGGTTCATGAAGATGAATCTTTGTCCGAAGTCAATTTACTC
AAAGGTGTAAAACTCATTGATGGTGGCTATGTACATCTTGCTGGTCTTGTGGTGACAGGTGAATGGAATTTGC
CAGATAATTGTCGTGGTGGTGTCAGTGTCTGTTTGGTCGATAAGAGAATGGAGAGAGCGGACGAGGCAACTC

TTGCTTCATACTATACCGCAGCGGCTAAGAAAAGGTTTCAGTTCAAAGTCGTTCCAAATTACAACATCACTACC
AAGGACGCAGAAAAGACAGTTTGGCAAGTACTAGTTAATATTAGAAATGTTAAAATTGCTGCGGGTTACTGTC
CGCTGTCATTAGAATTTGTGTCAGTGTGTATTGTTTATAAAAATATTATAAAACTCGGTTTGAGAGAGAAAATT
ACGAGCGTCACGGATGGAGGGCCCATGGAACTATCAGAAGAAGTTGTTGATGAGTTCATGGAAGAAGTCCC
GATGTCTGTAAGGCTTGCAAAATTTCGTTCGAAGACCGGAAAAAAGTTTAGTAGTAAAAGTGAGAATAATAG
TGGTAATAATAGGCCGAAACCAGACAAAAACCAAAGGAAGAAAAAGGGTTTAAAAGTTAGGGTTGAGAAGG
ATAATTTAATTGATAATGAATTGGAGACTTACGTCGCCGATTCAGATTCGTATTAAATATGTCTTACACAATCG
CAACTCCATCGCAATTTGTGTTTTTGTCATCAGCATGGGCAGACCCTATAGAATTAATAAATTTATGTACTAATT
CACTAGGTAATCAGTTCCAAACACAACAAGCTAGAACAACCGTTCAACGGCAATTTAGCGAAGTGTGGAAACC
TGTCCCTCAAGTCACTGTTAGGTTTCCTGACAGTGGTTTTAAGGTGTATAGGTACAATGCGGTACTAGATCCTC
TAGTTACTGCTTTGTTAGGAGCTTTCGATACTAGAAATAGGATTATAGAAGTCGAAAATCAGGCGAACCCGAC
AACCGCCGAAACGTTAGACGCTACTCGTAGAGTAGATGACGCAACGGTGGCTATAAGGAGCGCTATAAATAA
TTTAGTAGTAGAATTGGTCAAAGGAACAGGTTTGTACAATCAGAGCACATTTGAAAGTGCATCCGGTTTACAA
TGGTCCTCTGCACCTGCATCTTGA
